# Synthesis of Highly Enantioenriched Sulfonimidoyl Fluorides and Sulfonimidamides by Stereospecific Sulfur–Fluorine Exchange (SuFEx) Reaction†

**DOI:** 10.1002/chem.202002265

**Published:** 2020-09-11

**Authors:** Stephanie Greed, Edward L. Briggs, Fahima I. M. Idiris, Andrew J. P. White, Ulrich Lücking, James A. Bull

**Affiliations:** ^1^ Department of Chemistry Imperial College London Molecular Sciences Research Hub White City Campus Wood Lane London W12 0BZ UK; ^2^ Bayer AG Pharmaceuticals Division Drug Discovery Müllerstr. 178 13353 Berlin Germany

**Keywords:** chirality, SuFEx reactions, sulfonimidamides, sulfur, synthetic methods

## Abstract

Sulfonimidamides present exciting opportunities as chiral isosteres of sulfonamides, with potential for additional directional interactions. Here, we present the first modular enantioselective synthesis of sulfonimidamides, including the first stereoselective synthesis of enantioenriched sulfonimidoyl fluorides, and studies on their reactivity. A new route to sulfonimidoyl fluorides is presented from solid bench‐stable, N‐Boc‐sulfinamide (Boc=*tert*‐butyloxycarbonyl) salt building blocks. Enantioenriched arylsulfonimidoyl fluorides are shown to be readily racemised by fluoride ions. Conditions are developed, which trap fluoride and enable the stereospecific reaction of sulfonimidoyl fluorides with primary and secondary amines (100 % *es*, *es*=enantiospecificity) generating sulfonimidamides with up to 99 % *ee*. Aryl and alkyl sulfonimidoyl fluoride reagents are suitable for mild late stage functionalisation reactions, exemplified by coupling with a selection of complex amines in marketed drugs.

## Graphical abstract

**S–F exchange**: Stereocontrolled synthesis provides highly enantioenriched sulfonimidoyl fluorides for SuFEx chemistry. Enantiospecific S−N bond formation is achieved with inversion by preventing fluoride promoted racemisation. A diverse array of sulfonimidamides is synthesised by using primary and secondary amines including complex amine‐containing drugs.

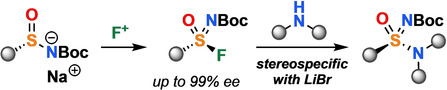

Directional interactions are crucial to the development of active ingredients in pharmaceutical and agrochemical products. Inclusion of chiral moieties can increase complementarity and hence potency and selectivity of a compound for a biological target.^1^ In contrast to sulfones and sulfonamides, their chiral aza‐analogues, that is, sulfoximines and sulfonimidamides (Figure 1 a), have been underrepresented in the life sciences despite their beneficial chemical properties.^2^, ^3^ They have high chemical and metabolic stability, and can improve physicochemical properties of a molecule, such as increased solubility, with the introduction of both hydrogen‐bond donor and acceptor capabilities for NH derivatives.^4^, ^5^ It is notable that sulfoximines have appeared in several new S^VI^ clinical candidates as single enantiomers (Figure 1 b).^6^, ^7^, ^8^ Sulfonimidamides are less developed in drug discovery, but present similar potential advantages.^9^ However, to date there are no general methods available to prepare these in enantioenriched form.

**Figure 1:**
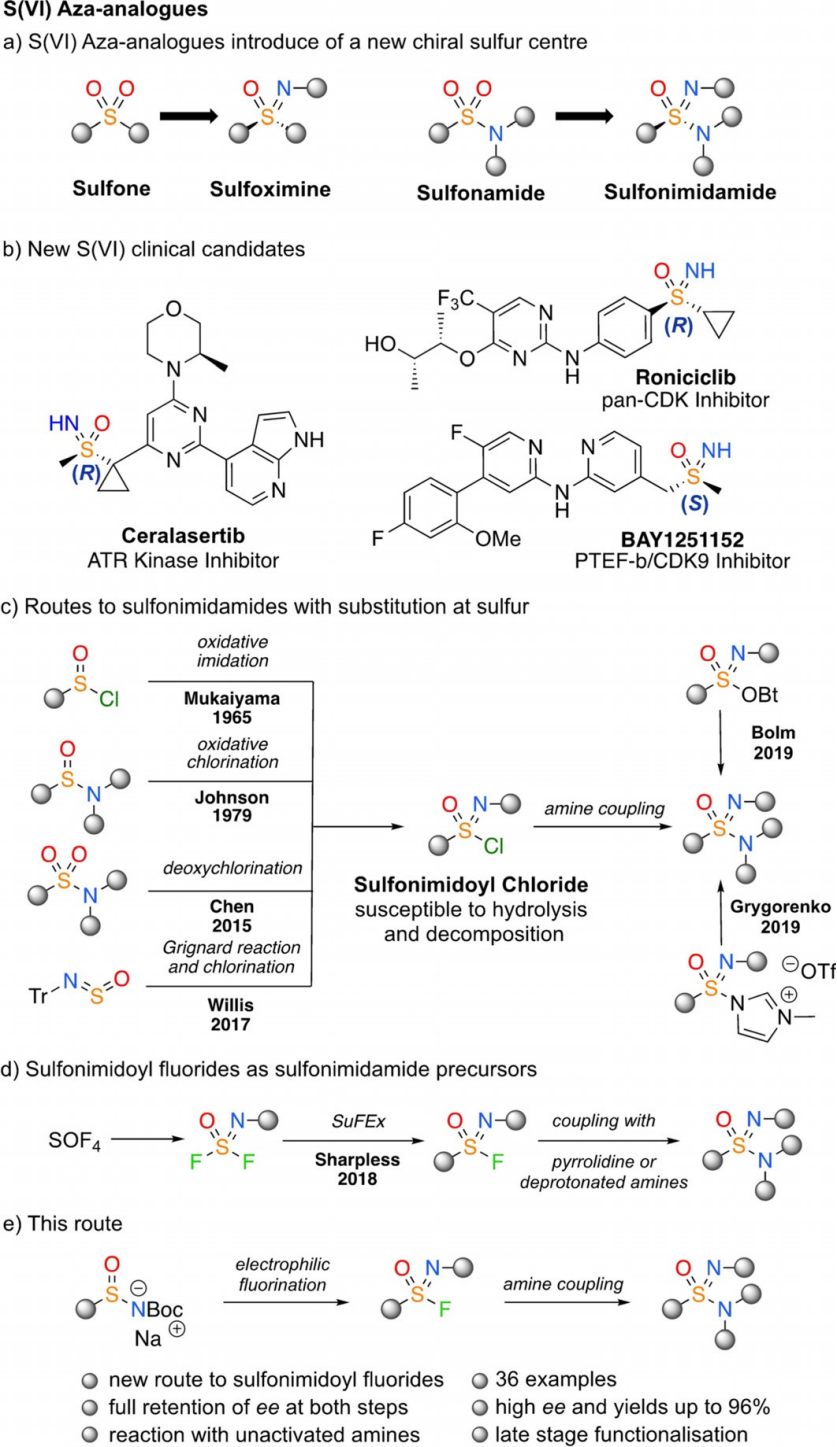
Structure of sulfoximine and sulfonimidamide groups and S^VI^ clinical candidates with asymmetric sulfur centres. Synthetic routes to access sulfonimidamides typically rely on sulfonimidoyl chloride precursors, alternative sulfonimidoyl‐X species, and this work as a new way to access enantioenriched sulfonimidoyl fluorides and sulfonimidamides.

Similarly, sulfonyl fluorides have been developed by Sharpless as click reagents in sulfur–fluorine exchange (SuFEx) reactions, and are used increasingly as biological probes.^10^, ^11^ To date, application of the chiral sulfonimidoyl fluoride derivatives as biological probes is limited,10a, 11c but presents interesting potential for improved specific directional interactions.

Methods for the synthesis of sulfonimidamides have developed significantly in recent years including powerful NH transfer methods.^12^ A valuable disconnection for divergent synthesis is formation of the S−N bond, by coupling an electrophilic S‐source with amines.^13^, ^14^ Several methods proceed via the sulfonimidoyl chloride, including recent powerful methods developed by Chen and Gibson^15^ and Willis et al. (Figure 1 c).^16^ However, sulfonimidoyl chlorides are typically unstable and decompose under basic, aqueous or reductive conditions, requiring in situ generation and amine coupling.^5^ Alternative sulfonimidoyl reagents have been developed as more stable sulfonimidamide precursors.^17^, ^18^, ^19^ Sharpless has used SOF_4_ gas to generate stable *N*‐aryl sulfonimidoyl fluoride reagents which were reacted with pyrrolidine or lithium amide reagents (Figure 1 d).^20^ It is notable that none of the methods described to date have been amenable to the preparation of enantioenriched derivatives.^21^

Enantioenriched sulfonimidoyl fluorides have been unknown until very recently. Zuilhof isolated the first example of an enantioenriched sulfonimidoyl fluoride, which was generated by the reaction of a racemic sulfonimidoyl chloride with KF, followed by separation of the enantiomers by chiral HPLC.^22^ Notably these reagents reacted with phenols in the presence of DBU, without requiring silylation, but underwent racemisation ascribed to the base. Using sodium phenolate provided enantioenriched sulfonimidates in a rapid reaction.

Here, we describe a method to prepare highly enantioenriched sulfonimidoyl fluorides from bench‐stable sulfinamide salts, and their use in the synthesis of a diverse range of enantioenriched sulfonimidamides by stereospecific SuFEx reaction (Figure 1 e). The first stereocontrolled synthesis of sulfonimidoyl fluorides is reported. Fluoride ions are demonstrated to cause racemisation of the sulfonimidoyl fluorides, which is avoided by fluoride trapping. The mild coupling reagents allow the use of neutral primary and secondary amines, and the methodology is exemplified in the functionalisation of amine containing drug compounds, showing the potential for its use to rapidly prepare novel chemical entities and diverse chemical libraries.^23^ A readily removed N‐Boc (Boc=*tert*‐butyloxycarbonyl) protecting group on the imide nitrogen is employed, which also increased the electrophilicity of the sulfonimidoyl fluoride.

By analogy with sulfonyl fluorides, we envisaged a new route to sulfonimidoyl fluorides through fluorination of sulfinamide salts.^24^, ^25^ Initially, racemic *p*‐tolyl sulfinamide salt **1 a** was prepared from the corresponding N‐Boc‐sulfoximine by elimination of acrylate (see the Supporting Information). Pleasingly, sulfonimidoyl fluoride **2 a** was formed in high yield and excellent purity using Selectfluor, after a simple aqueous workup (Scheme 1 a, Procedure A). Furthermore, we developed conditions for the reaction **2 a** with 11 examples of primary and secondary amines (Procedure B; for example, piperidine, 77 % yield **3 a** in Scheme 1 a. See Supporting Information for further examples).

**Scheme 1:**
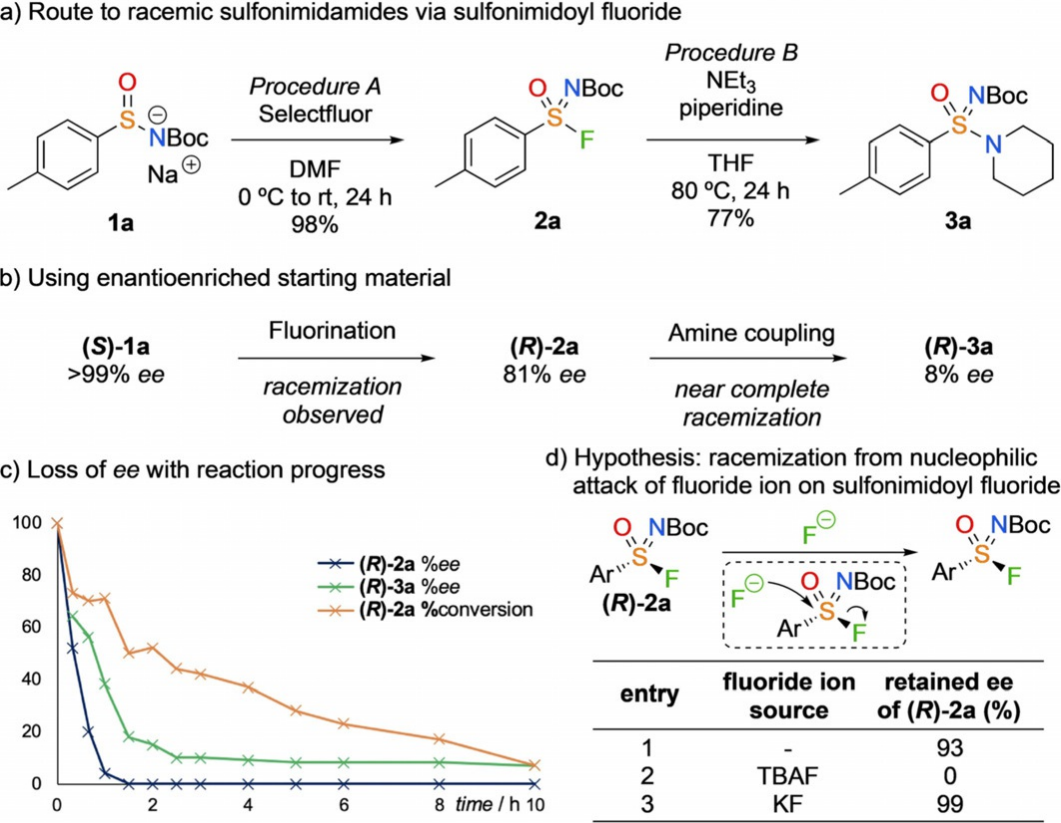
a) New route to sulfonimidamides via sulfonimidoyl fluorides was developed, however racemisation was observed in both fluorination and amine coupling steps, understood to be from sulfonimidoyl fluoride racemisation. For details of procedures A and B, see the Supporting Information. b) Loss of *ee* using enantioenriched materials under the initially developed conditions. c) *ee* and conversion with time for the reaction of enantioenriched **(*R*)*‐*2 a** and piperidine under procedure B. d) Reactions were performed on a 0.1 mmol scale in THF (0.3 m) at RT for 3 h. Retained *ee* given by % *ee*_**(*R*)‐2 a** product_/% *ee*_**(*R*)‐2 a** SM_ (SM=starting material).

Although this route demonstrated the proof of concept, a key aim for this project was in the development of an efficient strategy to enantioenriched sulfonimidamides. The corresponding enantioenriched salt **(*S*)‐1 a** was formed from commercial (*S*)‐(+)‐*p*‐toluenesulfinamide **(*S*)‐4** (Table 1; **(*S*)‐4** to **(*S*)‐1 a**). However, when using the enantioenriched sulfinamide salt, a significant loss of *ee* occurred in both fluorination and coupling steps (Scheme 1 b). Monitoring the reduction of *ee* over the course of the SuFEx reaction showed sulfonimidoyl fluoride **(*R*)‐2 a** rapidly racemised (Scheme 1 c, see the Supporting Information for additional data). Sulfonimidamide **(*R*)‐3 a** retained a low *ee* and was proven to be configurationally stable under the reaction conditions.

**Table 1:** Optimisation of amine coupling for retention of *ee* and yield.

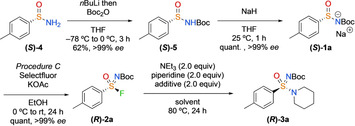						
Entry	Solvent	Additive	Yield [%]^[a]^			[% *es*]^[c]^
			**(*R*)‐2 a**	**(*R*)‐3 a**	total^[b]^	
1	THF	–	30	52	82	8
2	THF	TMS‐Cl	75	1	76	n.d.
3	THF	H_2_O	6	77	83	26
4	THF	KBr	33	44	77	13
5	THF	LiCl	11	31	42	>99
6	THF	LiBr	19	56	75	>99
7	EtOH	–	–	33	33	45
8	*i*PrOH	–	6	66	72	29
9	MeCN	–	9	73	82	28
10	MeCN	LiBr	–	96	96	>99
11	MeCN	LiI	–	87	87	>99

Optimisation of reaction conditions for yield and *ee* of **(*R*)‐3 a**. Reactions were performed using 0.1 mmol **(*R*)‐2 a**. [a] Yields determined by ^1^H NMR spectroscopy by using 1,3,5‐trimethoxybenzene as internal standard. [b] Sum of preceding two columns. [c] *es*=enantiospecificity, given by % *ee*_**(*R*)‐3 a**_/% *ee*_**(*R*)‐2 a**_. For details on Procedure C, see the Supporting Information.

We proposed that degenerate nucleophilic attack of fluoride ions in solution on the sulfonimidoyl fluoride centre was causing the racemisation (Scheme 1 d). To explore this hypothesis, two fluoride ion sources, tetra‐*n*‐butylammonium fluoride (TBAF) and KF, were added to the sulfonimidoyl fluoride **(*R*)‐2 a** in THF at RT for 3 h. In the absence of a soluble fluoride ion source (Figure 1 d, entry 1), a small amount of racemisation occurred, presumably as a result of elimination of fluoride ions from the starting material. A soluble fluoride source (TBAF, Figure 1 d, entry 2) caused the complete racemisation of the sulfonimidoyl fluoride whereas the highly insoluble, inorganic KF did not release fluoride ions into solution and may, in fact, have complexed with F^−^ present to prevent racemisation (Figure 1 d, entry 3).^26^, ^27^

This directed us to examine fluoride trapping strategies (see the Supporting Information). Firstly, in the fluorination step, changing the solvent to ethanol, being polar and protic, resulted in full preservation of *ee*. However, the yield of the fluorination was much reduced, presumably from the protonation of the sulfinamide salt causing reduced nucleophilicity of the sulfur centre. The introduction of potassium acetate as a soluble inorganic base resulted in an increased yield for this step with no loss of *ee* (Table 1, **(*S*)‐1 a** to **(*R*)‐2 a**).

Preventing racemisation of the sulfonimidoyl fluoride in the amine coupling step required more extensive optimisation (Table 1, **(*R*)‐2 a**‐**(*R*)‐3 a**). A selection of trapping additives was examined (Table 1, entries 2–6). A typical organic fluoride ion scavenger, TMS‐Cl (TMS=trimethylsilyl) shut down the reactivity and resulted in almost complete recovery of starting material. The addition of water resulted in an increased yield, however, there was only a small increase in *es* observed. Soluble inorganic salts were investigated to precipitate insoluble fluoride salts.^26^ Adding KBr was not beneficial, whereas LiCl gave complete preservation of *ee*. The use of more soluble LiBr resulted in complete preservation of *ee* and an increase in yield. Changing the solvent had a lesser effect on *ee* than in the fluorination step and alcohol solvents caused a significant amount of sulfonimidate formation (Table 1, entries 7 and 8). However, changing the solvent to MeCN resulted in an increased yield (Entry 9), and when combined with LiBr resulted in the formation of an enantioenriched sulfonimidamide in excellent yield (Table 1, entry 10).

With the optimised conditions in hand, the amine scope of the reaction was explored (Scheme 2). Pleasingly, both primary and secondary amines were suitable in this reaction with complete enantiospecificity in all cases. Aliphatic, benzylic, and allylic amines were all coupled in good yields (**(*R*)‐3 b**–**(*R*)‐3 g**). Acyclic and cyclic secondary amines reacted in excellent yields (**(*R*)‐3 h**–**(*R*)‐3 l**). Ketones and *gem*‐difluoro substituents were all well tolerated on the amine substrate (**(*R*)‐3 m–(*R*)‐3 n**). Chemoselective reactivity was observed with 4‐piperidinol to form sulfonimidamide **(*R*)‐3 o** without significant competing sulfonimidate formation. Finally, more highly functionalised piperidines and piperazine heterocycles were coupled in good yield under the mild conditions (**(*R*)‐3 p–(*R*)‐3 s**), including the drug desipramine. Treating **(*R*)‐2 a** with both enantiomers of α‐methylbenzylamine gave different single diastereoisomer products (**(*R*)‐3 t** and **(*R*)‐3 u**).

**Scheme 2:**
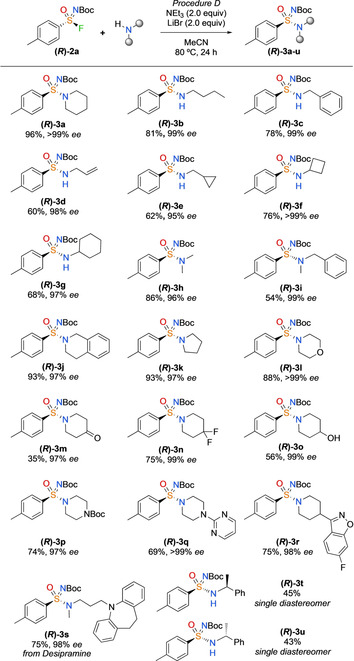
Amine scope using the *p*‐tolyl sulfinamide salt. Reactions performed on 0.25 mmol scale. Coupling reaction performed by using **(*R*)‐2 a** (95–99 % *ee*) with no loss of *ee* in this step.

Sulfonimidamide **(*R*)‐3 h** was determined to be (*R*)‐stereochemistry by single crystal X‐ray diffraction analysis (Scheme 3). This indicates inversion in the substitution reaction. Nucleophilic substitution with inversion at the sulfur centre has precedent with sulfonimidoyl chlorides and sulfonimidates,^14^, ^21^, ^28^ and more recently in the nucleophilic attack of phenols on sulfonimidoyl fluorides.^22^ Fluorination is presumed to occur with retention of the configuration similar to prior studies on chlorination (*S* changing to *R* due to priority change).^29^

**Scheme 3:**
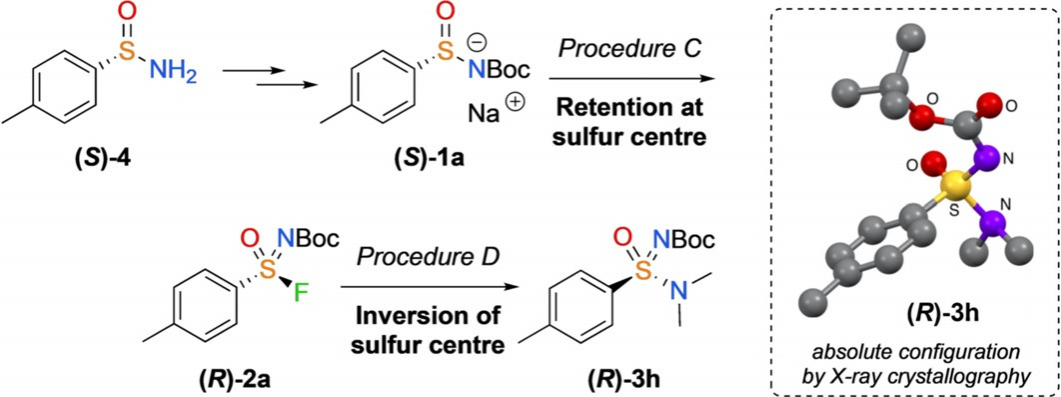
From the commercially available (*S*)‐enantiomer of the sulfinamide salt building block, the fluorination–amine coupling sequence was proven to occur with overall inversion of the sulfur centre from crystal structure analysis, rationalised to occur in the amine coupling step. Deposition Number 1991431 contains the supplementary crystallographic data for this paper. These data are provided free of charge by the joint Cambridge Crystallographic Data Centre and Fachinformationszentrum Karlsruhe Access Structures service www.ccdc.cam.ac.uk/structures.

To exemplify a general approach to enantioenriched sulfinamides, we targeted 4‐bromophenylsulfonimidoyl fluoride **(*R*)‐2 b**, exploiting enantioselective sulfoxide oxidation as the asymmetric step (Scheme 4).

**Scheme 4:**
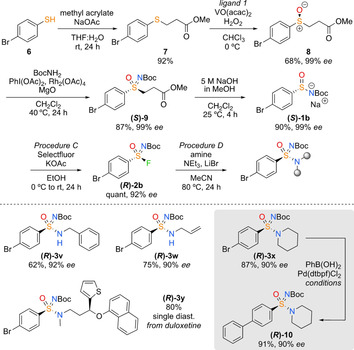
Preparation of enantioenriched 4‐bromophenyl sulfonimidamides. Ligand 1=(*S*)‐(−)‐2‐(*N*‐3,5‐diiodosalicyliden)amino‐3,3‐dimethyl‐1‐butanol. Stereochemistry of **8** assigned based on prior literature.^30^ Suzuki conditions=PhB(OH)_2_ (1.5 equiv), K_2_CO_3_ (2 equiv), [Pd(dtbpf)Cl_2_] (dtbpf =1,1′‐bis(di‐*tert*‐butylphosphino)ferrocene) (10 mol %), MeCN/H_2_O (1:1, 0.2 m), 80 °C, 2 h.

Catalytic enantioselective oxidation of sulfide **7** gave sulfoxide **8** in 68 % yield and 99 % *ee* (*S*).^30^ Rh‐catalysed N‐Boc transfer to form the sulfoximine **(*S*)‐9**,^31^ which has been shown to occur with retention of *ee*, and elimination of methyl acrylate gave the bench‐stable sulfinamide salt **(*S*)‐1 b**. The elimination occurred with preservation of *ee*, as indicated by reprotonation of a sample of the salt. This provides a new approach to enantioenriched sulfinamide salts. Treatment of **(*S*)‐1 b** with Selectfluor gave sulfonimidoyl fluoride **(*R*)‐2 b** with 92 % *ee*. Reaction of **(*R*)‐2 b** with amines gave high yields and maintained high *ee* (**(*R*)‐3 v**–**(*R*)‐3 x**). Using the enantiopure drug compound duloxetine yielded a single diastereoisomer product **(*R*)‐3 y**. Moreover, the 4‐bromophenyl substituent of **(*R*)‐3 x** was shown to be a suitable handle for further derivatisation, as exemplified in a Suzuki–Miyaura cross‐coupling to give enantioenriched biphenyl derivative **(*R*)‐10**.

Moreover, additional racemic aryl and alkyl sulfinamide salts **1 c**–**1 h** were prepared (see the Supporting Information). These were converted to new sulfonimidoyl fluorides **2 c**–**2 h** with Selectfluor and coupled with piperidine to form a collection of N‐Boc‐protected sulfonimidamides (**3 z**–**3 aj**, Scheme 5). Electron‐rich methoxyphenyl (**3 ab**), and electron‐poor pyridine derivatives (**3 ac**) were successfully employed. Alkyl sulfinamide salts were also successfully converted to piperidine sulfonimidamides **3 ad** and **3 ae**. Procedure A (DMF) was more suitable for the fluorination step with the alkyl derivatives. The SuFEx reaction with methylsulfonimidoyl fluoride **2 h** was demonstrated with several amines, including the marketed drugs primaquine, desipramine and amoxapine (**3 ah**–**3 aj**).

**Scheme 5:**
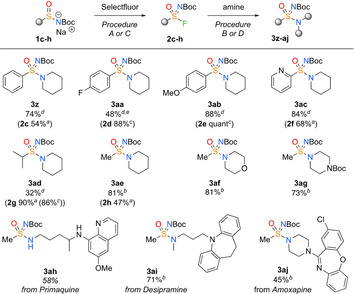
Scope of varying sulfinamide salt building blocks in fluorination and SuFEx amine coupling reactions. For reaction conditions and equivalents, see the Supporting Information. a) Procedure A; b) Procedure B; c) Procedure C; d) Procedure D. e) Competing S_N_Ar reactivity gave reduced yield of **3 aa**.

Finally, the N‐Boc protecting‐group was readily removed on both tertiary and secondary enantioenriched sulfonimidamide substrates with trifluoroacetic acid (TFA) in CH_2_Cl_2_ (Scheme 6). Treating **(*R*)‐3 a** and **(*R*)‐3 f** with TFA effected deprotection with no racemisation to give NH‐sulfonimidamides **(*R*)‐11** and **(*R*)‐12**.

**Scheme 6:**
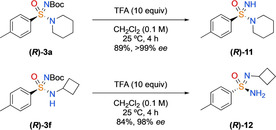
N‐Boc deprotection of primary and secondary sulfonimidamide. Sample of **(*R*)‐3 f** used had 98 % *ee*, with N‐Boc deprotection occurring with complete preservation of *ee*.

In conclusion, we have reported a new, practical method to access sulfonimidamides from bench‐stable sulfinamide salt building blocks by a SuFEx reaction of sulfonimidoyl fluorides. We describe the first facile route to enantioenriched sulfonimidamides, which are currently underrepresented in the life sciences. Moreover, the stereocontrolled synthesis of enantioenriched sulfonimidoyl fluorides is reported for the first time. Similar to the achiral sulfonyl fluorides, we see great potential for enantioenriched sulfonimidoyl fluorides as novel warheads for chemical biology and molecular pharmacology. Our synthetic methodology has a broad substrate scope of sulfinamide salt starting materials and both primary and secondary amines are suitable coupling partners for the SuFEx reaction to access a diverse array of sulfonimidamides. The methodology can be applied to the late stage functionalisation of drug molecules all in good to excellent yields, which has the potential to accelerate the preparation of novel chemical entities and diverse chemical libraries.

## Conflict of interest

The authors declare no conflict of interest.

## Supporting information

As a service to our authors and readers, this journal provides supporting information supplied by the authors. Such materials are peer reviewed and may be re‐organized for online delivery, but are not copy‐edited or typeset. Technical support issues arising from supporting information (other than missing files) should be addressed to the authors.

Supplementary
