# Supplementary material for: Synthesis of Highly Enantioenriched Sulfonimidoyl Fluorides and Sulfonimidamides by Stereospecific Sulfur–Fluorine Exchange (SuFEx) Reaction
Source: Chemistry. 2020 Sep 11;26(55):12533–8. doi: 10.1002/chem.202002265 (PMC7590120; doi:10.1002/chem.202002265)
Supplement: Supplementary file 1 — Supplementary [file CHEM-26-12533-s001.pdf]

Chemistry–A European Journal

Supporting Information

Synthesis of Highly Enantioenriched Sulfonimidoyl Fluorides and Sulfonimidamides by Stereospecific Sulfur–Fluorine Exchange (SuFEx) Reaction**

Stephanie Greed,[a] Edward L. Briggs,[a] Fahima I. M. Idiris,[a] Andrew J. P. White,[a] Ulrich Lücking,[b] and James A. Bull*[a]

Table of Contents

Extended References from Manuscript 3

General Experimental Conditions 4

Structures of Additional Compounds in SI 5

General Procedures 6

General Procedure A: Synthesis of racemic sulfonimidoyl fluorides 6

General Procedure B: Synthesis of racemic sulfonimidamides 6

General Procedure C: Synthesis of enantioenriched sulfonimidoyl fluorides 6

General Procedure D: Synthesis of enantioenriched sulfonimidamides 6

Initial route to racemic sulfonimidamides using Procedures A and B 7

Synthesis of enantioenriched sulfinamide salt ((S)-1a) 8

Applying the racemic conditions to enantioenriched starting materials 9

Determination of *ee* and conversion along the timescale of the reaction 10

Sulfonimidoyl Fluoride Racemisation in the Presence of Fluoride Ions 11

Optimisation of Synthesis of Enantioenriched Sulfonimidoyl Fluoride (R)-2a 12

Optimisation of Synthesis of Enantioenriched Sulfonimidamide (R)-3a 13

Experimental and Characterisation Data: Amine scope with (R)-2a (Scheme 2) 14

Crystal Structure Data for (R)-3h 26

Preparation of enantioenriched 4-bromophenyl derivatives (Scheme 4) 27

Experimental Data for Racemic Sulfinamide Salts (S4b-f) 33

Synthesis of sulfinamide salts 33

Synthesis of Sulfonimidoyl Fluorides (2c-2h) 42

Synthesis of Racemic Sulfonimidamides (3z-aj) 45

Experimental Procedures for NBoc-deprotection 49

X-Ray Crystallography Supplementary Data 51

References 52

^{1}H and ^{13}C-NMR Spectra *53*

HPLC Data *148*

List of Supplementary Figures

Figure S1: The crystal structure of (*R*)-3h……..**26**

Figure S2: The crystal structure of (*R*)-3h (50% probability ellipsoids)…….**51**

Extended References from Manuscript

[2b] F. Sehgelmeble, J. Janson, C. Ray, S. Rosqvist, S. Gustavsson, L. I. Nilsson, A. Minidis, J. Holenz, D. Rotticci, J. Lundkvist and P. I. Arvidsson, *ChemMedChem*, **2012**, *7*, 396.

[6a] A. Min, S.-A. Im, H. Jang, S. Kim, M. Lee, D. K. Kim, Y. Yang, H.-J. Kim, K.-H. Lee, J. W. Kim, T.-Y Kim, D.-Y. Oh, J. Brown, M. J. O'Connor, Y.-J. Bang, *Mol. Cancer Ther.,* **2017**, *16*, 566.

[6b] K. M. Foote, J. W. M. Nissink, T. McGuire, P. Turner, S. Guichard, J. W. T. Yates, A. Lau, K. Blades, D. Heathcote, R. Odedra, G. Wilkinson, Z. Wilson, C. M. Wood and P. J. Jewsbury, *J. Med. Chem.*, **2018**, *61*, 9889.

[7a] U. Lücking, R. Jautelat, M. Krüger, T. Brumby, P. Lienau, M. Schäfer, H. Briem, J. Schulze, A. Hillisch, A. Reichel, A. M. Wengner and G. Siemeister, *ChemMedChem*, **2013**, *8*, 1067.

[7b] U. Lücking, A. Scholz, P. Lienau, G. Siemeister, D. Kosemund, R. Bohlmann, H. Briem, I. Terebesi, K. Meyer, K. Prelle, K. Denner, Ulf Bömer, M. Schäfer, K. Eis, R. Valencia, S. Ince, F. von Nussbaum, D. Mumberg, K. Ziegelbauer, B. Klebl, A. Choidas, P. Nussbaumer, M. Baumann, C. Schultz-Fademrecht, G. Rühter, J. Eickhoff, M. Brands, *ChemMedChem*, **2017**, *12*, 1776

[8] U. T. Luecking, A. Scholz, D. Kosemund, R. Bohlmann, H. Briem, P. Lienau, G. Siemeister, I. Terebesi, K. Meyer, K. Prelle, R. Valencia, S. Ince, F. von Nussbaum, D. Mumberg, K. Ziegelbauer and M. Brands, in *Cancer Chemistry*, American Association for Cancer Research, **2017**, *77*, 984.

General Experimental Conditions

All non-aqueous reactions were run under an inert atmosphere (argon) with flame-dried glassware, using standard techniques. Anhydrous solvents were obtained by filtration through drying columns (THF, CH_2Cl_2, DMF, MeCN, EtOH and toluene) or used as supplied. Reactions for the scope optimisation were carried out in sealed Biotage microwave vials.

Flash chromatography was performed using 230–400 mesh silica, with the indicated solvent system according to standard techniques. Analytical thin-layer chromatography (TLC) was performed on precoated, glassbacked silica gel plates. Visualization of the developed chromatogram was performed by UV absorbance (254 nm) and stained with aqueous potassium permanganate solution or a ninhydrin solution in reagent stain.

Nuclear magnetic resonance spectra were recorded on 400 MHz spectrometers. The frequency used to record the NMR spectra is given in each assignment and spectrum (^{1}H NMR at 400 MHz; ^{13}C NMR at 101 MHz; ^{19}F NMR at 377 MHz). Chemical shifts for ^{1}H NMR spectra are recorded in parts per million with the residual protic solvent resonance as the internal standard (chloroform: δ = 7.26 ppm, D_2O: δ = 4.79 ppm). Data is reported as follows: chemical shift (multiplicity [s = singlet, d = doublet, t = triplet, q = quartet, quint. = quintet, m = multiplet and br = broad], coupling constant (in Hz), integration and assignment). ^{13}C NMR spectra were recorded with complete proton decoupling. Chemical shifts are reported in parts per million with the residual protic solvent resonance as the internal standard ($^{13}CDCl_3$: δ = 77.2 ppm). Assignments of ^{1}H and ^{13}C spectra were based upon the analysis of δ_H and J values, as well as DEPT, COSY and HSQC experiments where appropriate. For clarity NMR spectra are displayed as follows unless this would obscure signals: ^{1}H NMR spectra are displayed between 10.0 ppm and 0.0 ppm; ^{13}C NMR spectra are displayed between 210 ppm and 0 ppm.

IR spectra were recorded as solids or neat liquids on an Agilent Cary 630 FTIR spectrometer and are reported in wavenumbers (cm^{-1}) to the nearest integer.

High-resolution mass spectrometry (HRMS) analyses were performed using electrospray ion source (ESI). This was performed using a Waters LCT Premier equipped with an ESI source operated in positive ion mode. The software used was MassLynx 4.1. This software does not account for the electron and all the calibrations/references are calculated accordingly, i.e. $[M+H]^+$ is detected and the mass is calibrated to output [M+H]. In the cases where this software is used we report the HRMS as [M+H].

All melting points were determined in open glass capillaries and are uncorrected.

Reagents: Commercial reagents were used as supplied or purified by standard techniques where necessary.

Observed optical rotation (α’) was measured at the indicated temperature (T °C) and were converted to the corresponding specific rotations $[\alpha]^{T}_{D}$ in deg cm^2 g^{-1} , concentration (c) in g per 100 mL.

HPLC analyses were carried out on an Agilent 1260 Infinity Series system, employing Daicel Chiracel columns.

Structures of Additional Compounds in SI

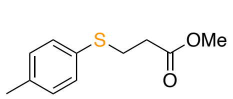
S1a

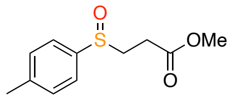
S2a

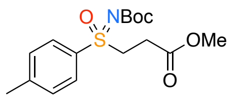
S3a

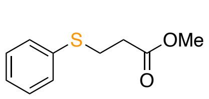
S1b

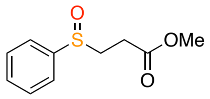
S2b

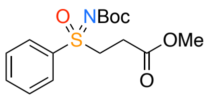
S3b

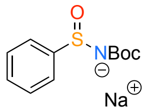
S4b

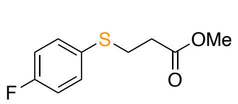
S1c

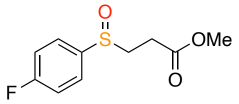
S2c

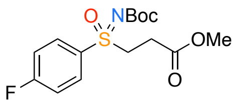
S3c

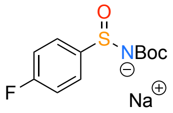
S4c

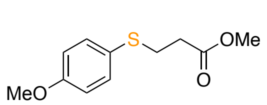
S1d

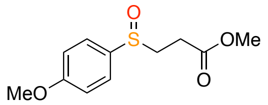
S2d

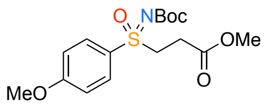
S3d

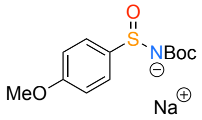
S4d

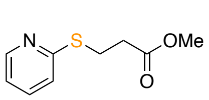
S1e

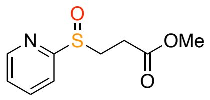
S2e

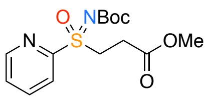
S3e

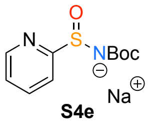
S4e

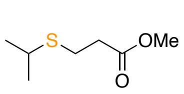
S1f

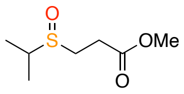
S2f

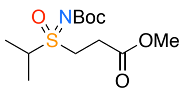
S3f

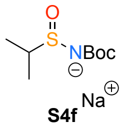
S4f

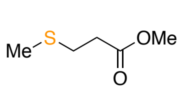
S1g

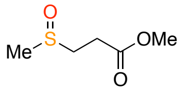
S2g

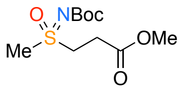
S3g

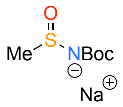
S4g

General Procedures

General Procedure A: Synthesis of racemic sulfonimidoyl fluorides

Selectfluor (1.32 g, 3.75 mmol, 1.5 equiv) was added to a solution of sulfinamide salt **1a-b, S4b-g** (2.5 mmol, 1 equiv) in DMF (13 mL, 0.2 M) at 0 °C and warmed to 25 °C for 18 h. H_2O (25 mL) was added and the aqueous mixture extracted with EtOAc (3 × 25 mL). The combined organic layers were dried (Na_2SO_4), filtered and the solvent removed under reduced pressure to give the racemic sulfonimidoyl fluorides **2a-h** which was typically used with no further purification.

General Procedure B: Synthesis of racemic sulfonimidamides

Amine (0.50 mmol, 2.0 equiv) and triethylamine (70 μL, 0.50 mmol, 2.0 equiv) were added to a stirred solution sulfonimidoyl fluoride **2a-h** (0.25 mmol, 1 equiv) in THF (0.83 mL, 0.3 M) at RT and warmed to 80 °C for 24 h. The solvent was removed under reduced pressure. The resulting residue was then purified by silica flash column chromatography as described for each entry to yield the sulfonimidamides **3a-3aj**.

General Procedure C: Synthesis of enantioenriched sulfonimidoyl fluorides

Selectfluor (0.71 g, 2.0 mmol, 2 equiv) were added to a stirred solution of sulfinamide salt **1a-b, S4b-g** (1.0 mmol, 1 equiv) and potassium acetate (0.20 g, 2.0 mmol, 2.0 equiv) in ethanol (5 mL, 0.2 M) at 0 ºC and slowly warmed to RT over 24 h. The reaction mixture was quenched with water (10 mL) and diluted with CH_2Cl_2 (10 mL). The mixture was extracted with CH_2Cl_2 (3 × 40 mL) and the combined organic layers were dried (Na_2SO_4), filtered and concentrated under reduced pressure. Typically, no further purification was required giving sulfonimidoyl fluoride **2a-h**.

General Procedure D: Synthesis of enantioenriched sulfonimidamides

Amine (0.50 mmol, 2.0 equiv) and triethylamine (70 μL, 0.50 mmol, 2.0 equiv) were added to a stirred solution of sulfonimidoyl fluoride **2a-h** (0.25 mmol, 1 equiv) and LiBr (43 mg, 0.50 mmol, 2.0 equiv) in MeCN (0.83 mL, 0.3 M) at RT and warmed to 80 °C for 24 h. The solvent was removed under reduced pressure. The resulting residue was then purified by silica flash column chromatography as described for each entry to yield the sulfonimidamides **3a-aj**.

Initial route to racemic sulfonimidamides using Procedures A and B

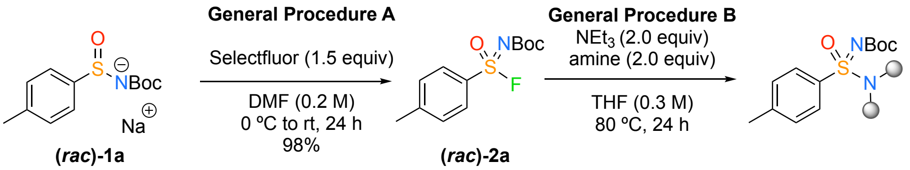

(*rac*)-1a **(*rac*)-2a**

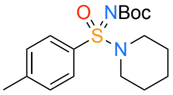

(*rac*)-3a: 77%

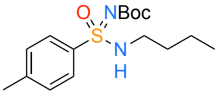

(*rac*)-3b: 65%

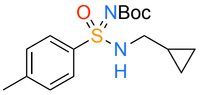

(*rac*)-3e: 42%

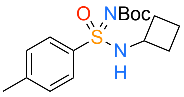

(*rac*)-3f: 54%

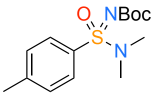

(*rac*)-3h: 74%

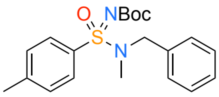

(*rac*)-3i: 61%

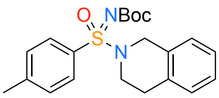

(*rac*)-3j: 68%

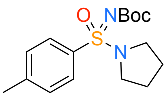

(*rac*)-3k: 64%

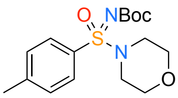

(*rac*)-3l: 78%

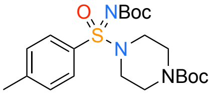

(*rac*)-3p: 74%

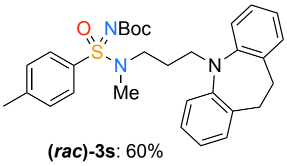

(*rac*)-3s: 60%

Reactions were performed on a 0.25 mmol scale. In each case, the racemic material was remade in their enantioenriched form using General Procedures B & D. The experimental and analytical data are given later in the Amine Scope section of the SI (p S14)

Synthesis of enantioenriched sulfinamide salt ((S)-1a)

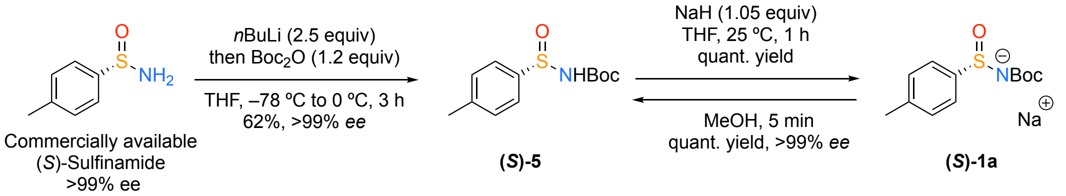

tert-Butyl (*p*-tolylsulfinyl)carbamate ((*S*)-5)

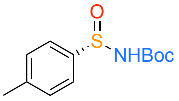

Prepared according to a literature procedure.[1] *n*-BuLi (1.52 M in hexanes, 10.6 mL, 16.1 mmol, 2.5 equiv) was added dropwise to a stirred solution of (*S*)-*p*-toluenesulfinamide (1.0 g, 6.4 mmol, 1 equiv) in THF (8 mL, 0.8 M) at –78 ºC. The mixture was stirred for 10 min followed by the addition of di-*tert*-butyl carbamate (1.70 g, 7.8 mmol, 1.2 equiv) in THF (5 mL, 1.5 M) and warmed to rt for 3 h. At 0 ºC, the reaction mixture was quenched with NH_4Cl solution (sat. aq., 10 mL) and diluted with CH_2Cl_2 (10 mL). The mixture was extracted with CH_2Cl_2 (5 × 15 mL) and the combined organic layers were dried (Na_2SO_4), filtered and concentrated under reduced pressure. Purification by recrystallisation (3:1 hexane/EtOAc) gave sulfinamide **(*S*)-5** as a white solid (1.03 g, 62%, >99% *ee*). mp = 90–92 °C. IR (film)/cm^{-1} 3116, 3064, 2971, 2922, 2814, 1703 (C=O), 1595, 1490, 1331, 1156, 1100, 898, 809. ^{1}H NMR (400 MHz, $CDCl_3$) δ 7.59 (d, *J* = 7.9 Hz, 2H, 2 × Ar–H), 7.32 (d, *J* = 7.9 Hz, 2H, 2 × Ar–H), 2.41 (s, 3H, Ar–CH_3), 1.49 (s, 9H, $C(CH_3)_3$). ^{13}C NMR (101 MHz, $CDCl_3$) δ 152.7 (C=O), 142.5 (Ar–C_q), 140.7 (Ar–C_q), 130.1 (2 × Ar–C), 124.8 (2 × Ar–C), 83.6 (*C*$(CH_3)_3$), 28.2 (C(*C*$H_3)_3$), 21.5 (Ar–CH_3). $[\alpha]^{21}_D$ = +80 (c 0.1, $CHCl_3$). HPLC Conditions: Chiralpak IB column, 98:2 *n*hexane:*i*PrOH, flow rate: 1 mL min^{-1}, 35 °C, UV detection wavelength: 260 nm, **((*S*)-5)** retention time: 22 min. Analytical data (NMR) in agreement with those reported in the literature.[2]

(*rac*)-5 HPLC Conditions: Chiralpak IB column, 98:2 *n*hexane:*i*PrOH, flow rate: 1 mL min^{-1}, 35 °C, UV detection wavelength: 260 nm, retention times: 22 & 24 min.

Sodium (*tert*-butoxycarbonyl)(*p*-tolylsulfinyl)amide ((*S*)-1a)

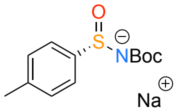

NaH (60% in oil, 52 mg, 1.23 mmol, 1.05 equiv) was added portionwise to sulfinamide **(*S*)-5** (300 mg, 1.23 mmol, 1 equiv) in THF (13 mL, 0.1 M) and stirred for 1 h at rt. The reaction mixture was quenched with MeOH (0.1 mL, 0.1 mmol, 0.05 equiv) and concentrated under reduced pressure. The precipitate was collected by filtration and washed with hexane to give sulfinamide salt **(*S*)-1a** (340 mg, 1.23 mmol, quant, >99% *ee*) as a white solid. mp = 233–234 °C. IR (film)/cm^{-1} 3086, 3049, 2922, 2960, 1642 (C=O), 1580, 1480, 1241, 1152, 1021, 798. ^{1}H NMR (400 MHz, D_2O) δ 7.54 (d, *J* = 8.2 Hz, 2H, 2 × Ar–H), 7.35 (d, *J* = 8.2 Hz, 2H, 2 × Ar–H), 2.37 (s, 3H, Ar–CH_3), 1.41 (s, 9H, $C(CH_3)_3$). ^{13}C NMR (101 MHz, D_2O) δ 165.9 (C=O), 143.2 (Ar–C_q), 141.7 (Ar–C_q), 129.6 (2 × Ar–C), 124.7 (2 × Ar–C), 79.6 (*C*$(CH_3)_3$), 27.8 (C(*C*$H_3)_3$), 20.5 (Ar–CH_3). HRMS (ESI) m/z Calcd for $C_{12}H_{16}NO_3S$ $[M]^-$: 254.0844; Found: 254.0851. $[\alpha]^{21}_D$ = +56 (c 1.0, H_2O).

Determination of *ee* from reprotonation. The minimum MeOH (~0.1 mL) was added to a sample of **(*S*)-1a** (~1 mg) until completely dissolved. An aliquot was removed and diluted with hexane for HPLC analysis of sulfinamide **(*S*)-5**.

Applying the racemic conditions to enantioenriched starting materials

When General Procedures A and B were applied to enantioenriched material, racemisation occurs at both steps in the synthesis.

***tert*-Butyl (fluoro(oxo)(*p*-tolyl)-λ^6-sulfaneylidene)carbamate ((*R*)-2a)**

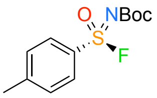

Reaction performed according to General Procedure A. Selectfluor (1.15 g, 3.25 mmol, 1.5 equiv) was added to a solution of sulfinamide salt **(*S*)-1a** (600 mg, 2.16 mmol, >99% *ee*, 1 equiv) in DMF (10.8 mL) at 0 °C and warmed to 25 °C for 18 h. H_2O (25 mL) was added and the aqueous mixture extracted with EtOAc (3 × 25 mL). The combined organic layers were dried ($MgSO_4$), filtered and the solvent removed under reduced pressure to give sulfonimidoyl fluoride **(*R*)-2a** (585 mg, 98%, 81% *ee*) as a colourless oil. IR (film)/cm^{-1} 2982, 2933, 1700 (C=O), 1595, 1454, 1327, 1141, 1096, 813, 678. ^{1}H NMR (400 MHz, $CDCl_3$) δ 7.99 (d, *J* = 8.4 Hz, 2H, 2 × Ar–H), 7.40 (d, *J* = 8.4 Hz, 2H, 2 × Ar–H), 2.48 (s, 3H, Ar–CH_3), 1.53 (s, 9H, $C(CH_3)_3$). ^{13}C NMR (101 MHz, $CDCl_3$) δ 152.7 (C=O), 147.1 (Ar–C_q), 130.8 (d, *J* = 20.9 Hz, Ar–C_q), 130.2 (2 × Ar–C), 128.3 (2 × Ar–C), 82.7 ($\mathit{C}(CH_3)_3$), 28.0 ($C(\mathit{C}H_3)_3$), 21.9 (Ar–CH_3). ^{19}F NMR (377 MHz, $CDCl_3$) δ 68.8. HRMS (ESI) m/z Calcd for $C_{12}H_{17}NO_3SF$ $[M+H]^+$: 274.0913; Found: 274.0924.

(*R*)-2a $[\alpha]^{21}_D$ = +9 (c 5.0, $CHCl_3$). HPLC Conditions: Chiralpak IA column, 99:1 *n*hexane:*i*PrOH, flow rate: 1 mL min^{-1}, 35 °C, UV detection wavelength: 260 nm, retention time: 13 & 14 min.

***tert*-Butyl (oxo(piperidin-1-yl)(*p*-tolyl)-λ^6-sulfaneylidene)carbamate ((*R*)-3a)**

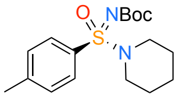

Reaction performed according to General Procedure B. Piperidine (49 μL, 0.50 mmol) and triethylamine (70 μL, 0.50 mmol) were added to a stirred solution of sulfonimidoyl fluoride **(*R*)-2a** (68.3 mg, 0.25 mmol) in THF (0.83 mL) at RT and warmed to 80 °C for 24 h. The solvent was removed under reduced pressure. Purification by flash column chromatography (SiO_2, 15% EtOAc/pentane) afforded sulfonimidamide **(*R*)-3a** (64.8 mg, 77%, 8% *ee*) as a white solid; mp = 137–139 °C. R_f 0.40 (15% EtOAc in pentane). IR (film)/cm^{-1} 2974, 2937, 2855, 1677 (C=O), 1595, 1454, 1364, 1275, 1156, 1092, 932, 816. ^{1}H NMR (400 MHz, $CDCl_3$) δ 7.73 (d, *J* = 8.3 Hz, 2H, 2 × Ar–H), 7.31 (d, *J* = 8.3 Hz, 2H, 2 × Ar–H), 3.10–3.06 (m, 4H, 2 × NCH_2), 2.42 (s, 3H, Ar–CH_3), 1.66–1.59 (m, 4H, 2 × $NCH_2C\mathit{H}_2$), 1.45–1.41 (m, 2H, $NCH_2CH_2C\mathit{H}_2$), 1.46–1.42 (s, 9H, $C(CH_3)_3$). ^{13}C NMR (101 MHz, $CDCl_3$) δ 156.8 (C=O), 143.8 (Ar–C_q), 133.4 (Ar–C_q), 129.8 (2 × Ar–C), 127.9 (2 × Ar–C), 80.1 ($\mathit{C}(CH_3)_3$), 46.7 (2 × NCH_2), 28.2 ($C(\mathit{C}H_3)_3$), 25.3 (2 × $NCH_2\mathit{C}H_2$), 23.7 ($NCH_2CH_2\mathit{C}H_2$), 21.6 (Ar–CH_3). HRMS (SI) *m/z* Calcd for $C_{17}H_{27}N_2O_3S$ $[M+H]^+$: 339.1742; Found: 339.1728.

(*R*)-3a $[\alpha]^{21}_D$ = –18 (c 0.5, $CHCl_3$). HPLC Conditions: Chiralpak IA column, 95:5 *n*hexane:*i*PrOH, flow rate: 1 mL min^{-1}, 35 °C, UV detection wavelength: 270 nm, retention time: 17 & 21 min.

Determination of *ee* and conversion along the timescale of the reaction

Sulfonimidoyl fluoride **(*R*)-2a** (273 mg, 1 mmol) was subjected to General Procedure B with the initial addition of 1,3,5-trimethoxybenzene (169 mg, 1 mmol). Aliquots (~50 μL) were removed from the sealed reaction vial at the timepoints given below and the sample was split and concentrated for preparation of ^{1}H-NMR and HPLC samples. Yields were determined using 1,3,5-trimethoxybenzene as the internal standard in the ^{1}H-NMR. %*ee* of the crude samples were obtained by HPLC analysis on both the **(*R*)-2a** and **(*R*)-3a** column conditions.

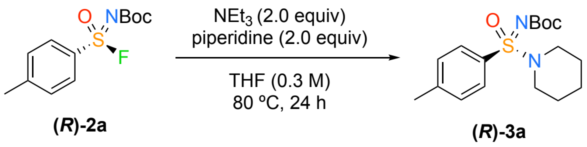

Time (h)	**(*R*)-2a** Yield (%)	**(*R*)-2a** %*ee*	**(*R*)-3a** Yield (%)	**(*R*)-3a** %*ee*
0.00	100	100	0	-
0.33	73	52	8	64
0.67	70	20	13	56
1	71	4	14	38
1.5	50	0	29	18
2	52	0	27	15
2	44	0	37	10
3	42	0	35	10
4	37	0	43	9
5	28	0	45	8
6	23	0	53	8
8	17	0	58	8
10	7	0	69	7
24	0	0	72	6
48	0	0	73	6

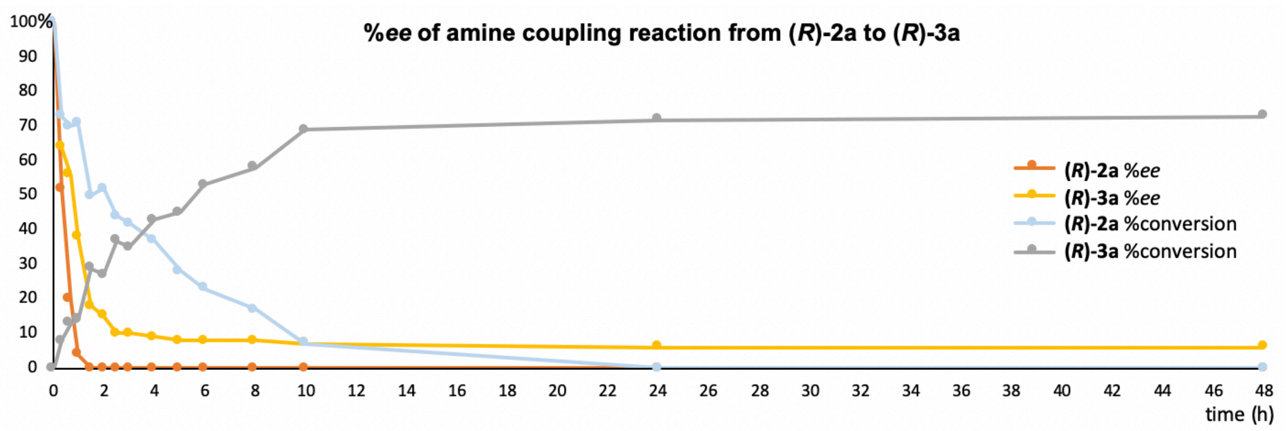

Sulfonimidoyl Fluoride Racemisation in the Presence of Fluoride Ions

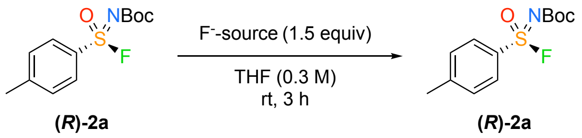

The fluoride source (0.15 mmol, 1.5 equiv) was added to **(*R*)-2a** (27.3 mg, 0.1 mmol, 1.0 equiv) in THF (0.33 mL, 0.3 M) at rt and stirred for 3 h. The reaction mixture was then filtered, concentrated under reduced pressure and dissolved in minimal amounts of hexane (~2 mL). An aliquot was removed for HPLC analysis to determine the *ee* of the returned **(*R*)-2a**.

HPLC Conditions: Chiralpak IA column, 99:1 nhexane:iPrOH, flow rate: 1 mL min-1, 35 °C, UV detection wavelength: 260 nm, retention time: 13 & 14 min.

**Entry**	**Fluoride Ion Source**	**Retained *ee* of (*R*)-2a (%)**
1	-	93
2	TBAF	0
3	KF	99

Retained *ee* given by %*ee*$_{(R)\text{-}2a\ \text{product}}$/%*ee*$_{(R)\text{-}2a\ \text{Starting material}}$

Optimisation of Synthesis of Enantioenriched Sulfonimidoyl Fluoride (*R*)-2a

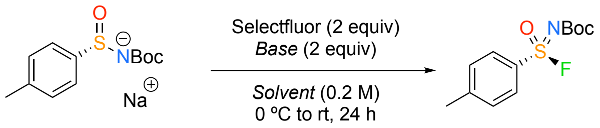

Entry	Solvent	Base	Yield (%)[a]			(*R*)-2a ee (%)
			Protonated (S)-1a	(*R*)-2a	Total[b]	
1	DMF	-	-	74	74	81-95
2	THF	-	19	30	49	68
3	MeCN	-	14	59	73	79
4	Et_2O	-	16	52	68	85-99
5	*i*PrOH	-	19	59	78	79
6	CH_2Cl_2	-	22	59	81	68
7	Hexane	-	22	57	79	44
8	EtOH	-	23	46	69	>99
9	EtOH	K_2CO_3	14	58	72	n.d.
10	EtOH	NaOAc	9	71	80	>99
11	EtOH	KOAc	2	80	82	>99
12	EtOH	NEt_3	72	trace	72	n.d.
13[c]	EtOH	KOAc	-	[98]	98	>99

Reactions performed on a 0.1 mmol scale. [a]Conversion determined by ^{1}H-NMR spectroscopy using 1,3,5-trimethoxybenzene as an internal standard. Isolated yield in parenthesis. [b]Sum of two preceding columns. [c]Reaction performed on a 1.2 mmol scale.

Optimisation of Synthesis of Enantioenriched Sulfonimidamide (*R*)-3a

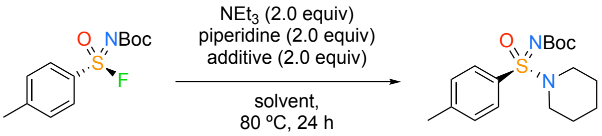

Entry	Solvent	Additive	Yield (%)[a]			% *es*[c]
			(*R*)-2a	(*R*)-3a	Total[b]	
1	THF	-	30	52	82	8
2	THF	TMS-Cl	75	1	76	n.d.
3	THF	KBr	33	44	77	13
4	THF	LiCl	11	31	42	>99
5	THF	H_2O	6	77	83	26
6	THF	LiBr	19	56	75	>99
7	EtOH	-	-	33	33	45
8	*t*BuOH	-	18	54	72	38
9	*i*PrOH	-	6	66	72	29
10	MeCN	-	9	73	82	28
11	MeCN	LiBr	-	96	96	>99
12	MeCN	LiI	-	87	87	>99
13	MeCN	Li_2CO_3	-	60	60	96

Reactions performed on a 0.1 mmol scale. [a]Conversion determined by ^{1}H-NMR spectroscopy using 1,3,5-trimethoxybenzene as an internal standard. Isolated yield in parenthesis. [b]Sum of two preceding columns. [c]*es*, enantiospecificity, given by $\%ee_{(R)\text{-}3a}/\ \%ee_{(R)\text{-}2a}$

Experimental and Characterisation Data: Amine scope with (*R*)-2a (Scheme 2)

tert-Butyl (fluoro(oxo)(*p*-tolyl)-λ^6-sulfaneylidene)carbamate ((*R*)-2a)

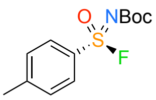

Reaction performed according to General Procedure C. Selectfluor (0.71 g, 2.0 mmol, 2 equiv) were added to a stirred solution of sulfinamide salt **(*S*)-1a** (0.29 g, 1.0 mmol, 1 equiv) and potassium acetate (0.20 g, 2.0 mmol, 2.0 equiv) in ethanol (5 mL, 0.2 M) at 0 ºC and slowly warmed to RT over 24 h. The reaction mixture was quenched with water (10 mL) and diluted with CH_2Cl_2 (10 mL). The mixture was extracted with CH_2Cl_2 (3 × 40 mL) and the combined organic layers were dried (Na_2SO_4), filtered and concentrated under reduced pressure. No further purification was required giving sulfonimidoyl fluoride **(*R*)-2a** (0.29 g, quant., >99% *ee*) as a colourless viscous oil. IR (film)/cm^{-1} 2982, 2933, 1700 (C=O), 1595, 1454, 1327, 1141, 1096, 813, 678. ^{1}H NMR (400 MHz, $CDCl_3$) δ 7.99 (d, *J* = 8.4 Hz, 2H, 2 × Ar–H), 7.40 (d, *J* = 8.4 Hz, 2H, 2 × Ar–H), 2.48 (s, 3H, Ar–CH_3), 1.53 (s, 9H, $C(CH_3)_3$). ^{13}C NMR (101 MHz, $CDCl_3$) δ 152.7 (C=O), 147.1 (Ar–C_q), 130.8 (d, *J* = 20.9 Hz, Ar–C_q), 130.2 (2 × Ar–C), 128.3 (2 × Ar–C), 82.7 ($\mathit{C}(CH_3)_3$), 28.0 ($C(\mathit{C}H_3)_3$), 21.9 (Ar–CH_3). ^{19}F NMR (377 MHz, $CDCl_3$) δ 68.8. HRMS (ESI) m/z Calcd for $C_{12}H_{17}NO_3SF$ $[M+H]^+$: 274.0913; Found: 274.0924. $[\alpha]^{21}_D$ = +9 (c 5.0, $CHCl_3$). HPLC Conditions: Chiralpak IA column, 99:1 *n*hexane:*i*PrOH, flow rate: 1 mL min^{-1}, 35 °C, UV detection wavelength: 260 nm, retention time: 13 & 14 min.

Synthesis of racemic sample for HPLC analysis performed according to General Procedure A, see p. S10: Selectfluor (533 g, 1.51 mmol, 1.5 equiv) was added to a solution of sulfinamide salt **(*rac*)-1a** (250 mg, 1.00 mmol) in DMF (5.00 mL) at 0 °C and warmed to 25 °C for 16 h. H_2O (10 mL) was added and the aqueous mixture extracted with EtOAc (3 × 15 mL). The combined organic layers were dried ($MgSO_4$), filtered and the solvent removed under reduced pressure to give sulfonimidoyl fluoride **(*rac*)-2a** (116 mg, 48%) as a colourless oil with characterisation data in accordance with the above.

tert-Butyl (oxo(piperidin-1-yl)(*p*-tolyl)-λ^6-sulfaneylidene)carbamate ((*R*)-3a)

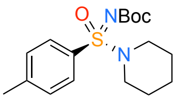

Reaction performed according to General Procedure D. Piperidine (50 μL, 0.50 mmol, 2.0 equiv) and triethylamine (70 μL, 0.50 mmol, 2.0 equiv) were added to a stirred solution of sulfonimidoyl fluoride **(*R*)-2a** (69 mg, 0.25 mmol, 1 equiv) and flame-dried LiBr (43 mg, 0.50 mmol, 2 equiv) in MeCN (0.83 mL, 0.3 M) at RT and warmed to 80 °C for 24 h. The solvent was removed under reduced pressure. Purification by flash column chromatography (SiO_2, 20% EtOAc/pentane) afforded sulfonimidamide **(*R*)-3a** (81.3 mg, 96%, >99% *ee*) as a white solid. mp = 137–139 °C. R_f 0.40 (15% EtOAc in pentane). IR (film)/cm^{-1} 2974, 2937, 2855, 1677 (C=O), 1595, 1454, 1364, 1275, 1156, 1092, 932, 816. ^{1}H NMR (400 MHz, $CDCl_3$) δ 7.72 (d, *J* = 8.3 Hz, 2H, 2 × Ar–H), 7.30 (d, *J* = 8.3 Hz, 2H, 2 × Ar–H), 3.07 (m, 4H, 2 × NCH_2), 2.41 (s, 3H, Ar–CH_3), 1.61 (p, *J* = 5.7 Hz, 4H, 2 × $NCH_2\mathit{CH_2}$), 1.49–1.41 (m, 2H, $NCH_2CH_2\mathit{CH_2}$), 1.39 (s, 9H, $C(CH_3)_3$). ^{13}C NMR (101 MHz, $CDCl_3$) δ 156.9 (C=O), 143.8 (Ar–C_q), 133.4 (Ar–C_q), 129.8 (2 × Ar–C), 127.9 (2 × Ar–C), 80.1 ($\mathit{C}(CH_3)_3$), 46.7 (2 × NCH_2), 28.2 ($C(\mathit{C}H_3)_3$), 25.3 (2 × $NCH_2\mathit{C}H_2$), 23.7 ($NCH_2CH_2\mathit{C}H_2$), 21.6 (Ar–CH_3). HRMS (ESI) *m/z* Calcd for $C_{17}H_{27}N_2O_3S$ $[M+H]^+$: 339.1742; Found: 339.1728. $[\alpha]^{21}_D$ = –18 (c 0.5, $CHCl_3$). HPLC Conditions: Chiralpak IA column, 95:5 *n*hexane:*i*PrOH, flow rate: 1 mL min^{-1}, 35 °C, UV detection wavelength: 270 nm, **((*R*)-3a)** retention time: 21 min.

Synthesis of racemic sample for HPLC analysis prepared according to General Procedure B, see p. S10, to afford sulfonimidamide **(*rac*)-3a** (64.8 mg, 77%) as a white solid with characterisation data in accordance with the above. HPLC Conditions: Chiralpak IA column, 95:5 *n*hexane:*i*PrOH, flow rate: 1 mL min^{-1}, 35 °C, UV detection wavelength: 270 nm, **((*rac*)-3a)** retention time: 17 & 21 min.

***tert*-Butyl (*R*)-((butylamino)(oxo)(*p*-tolyl)-λ^6-sulfaneylidene)carbamate ((*R*)-3b)**

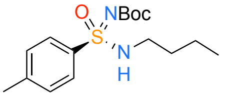

Reaction performed according to General Procedure D. Butylamine (50 μL, 0.50 mmol, 2.0 equiv) and triethylamine (70 μL, 0.50 mmol, 2.0 equiv) were added to a stirred solution of sulfonimidoyl fluoride **(*R*)-2a** (69 mg, 0.25 mmol, 1 equiv) and flame-dried LiBr (43 mg, 0.50 mmol, 2 equiv) in MeCN (0.83 mL, 0.3 M) at RT and warmed to 80 °C for 24 h. The solvent was removed under reduced pressure. Purification by flash column chromatography (SiO_2, 5% Et_2O in CH_2Cl_2) afforded sulfonimidamide **(*R*)-3b** (66.2 mg, 81%, 99% *ee*) as a white solid. mp = 135–137 °C. R_f 0.32 (5% Et_2O in CH_2Cl_2). IR (film)/cm^{-1} 3068, 2960, 2930, 2870, 1681 (C=O), 1454, 1275, 1163, 1118, 902, 813. ^{1}H NMR (400 MHz, $CDCl_3$) δ 7.82 (d, *J* = 8.3 Hz, 2H, 2 × Ar–H), 7.30 (d, *J* = 8.1 Hz, 2H, 2 × Ar–H), 6.87 (s, 1H, NH), 2.98–2.93 (m, 1H, NC*H*H), 2.77–2.69 (m, 1H, NCH*H*), 2.42 (s, 3H, Ar–CH_3), 1.51–1.42 (m, 2H, NCH_2CH_2), 1.35 (s, 9H, $C(CH_3)_3$), 1.28–1.22 (m, 2H, CH_2CH_3), 0.83 (t, *J* = 7.3 Hz, 3H, CH_2CH_3). ^{13}C NMR (101 MHz, $CDCl_3$) δ 157.1 (C=O), 143.8 (Ar–C_q), 135.8 (Ar–C_q), 129.7 (2 × Ar–C), 128.1 (2 × Ar–C), 80.1 ($C(CH_3)_3$), 41.0 (NCH_2), 31.4 (NCH_2CH_2), 28.1 ($C(CH_3)_3$), 21.6 (Ar–CH_3), 19.8 (CH_2CH_3), 13.6 (CH_2CH_3). HRMS (ESI) *m/z* Calcd for $C_{16}H_{27}N_2O_3S$ $[M+H]^+$: 327.1742; Found: 327.1739. $[\alpha]^{21}_D$ = +42 (c 1.0, $CHCl_3$). HPLC conditions: Chiralpak IA column, 97:3 *n*hexane:*i*PrOH, flow rate: 1 mL min^{-1}, 35 °C, UV detection wavelength: 254 nm, **((*R*)-3b)** retention time: 21 min.

Synthesis of racemic sample for HPLC analysis prepared according to General Procedure B to afford sulfonimidamide **(*rac*)-3b** (53.0 mg, 65%) as a white solid with characterisation data in accordance with the above. HPLC conditions: Chiralpak IA column, 97:3 *n*hexane:*i*PrOH, flow rate: 1 mL min^{-1}, 35 °C, UV detection wavelength: 254 nm, **(*rac*)-3b** retention times: 21 & 23 min.

***tert*-Butyl (*R*)-((benzylamino)(oxo)(*p*-tolyl)-λ^6-sulfaneylidene)carbamate ((*R*)-3c)**

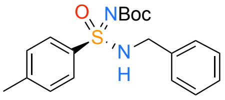

Reaction performed according to General Procedure D. Benzylamine (50 μL, 0.50 mmol, 2.0 equiv) and triethylamine (70 μL, 0.50 mmol, 2.0 equiv) were added to a stirred solution of sulfonimidoyl fluoride **(*R*)-2a** (69 mg, 0.25 mmol, 1 equiv) and flame-dried LiBr (43 mg, 0.50 mmol, 2 equiv) in MeCN (0.83 mL, 0.3 M) at RT and warmed to 80 °C for 24 h. The solvent was removed under reduced pressure. Purification by flash column chromatography (SiO_2, 5% Et_2O in CH_2Cl_2) afforded sulfonimidamide **(*R*)-3c** (70.2 mg, 78%, 99% *ee*) as a white solid. mp = 62–63 °C. R_f 0.39 (10% Et_2O in CH_2Cl_2). IR (film)/cm^{-1} 3064, 2978, 2930, 2840, 1677, 1454, 1249, 1152, 1115, 1059, 906, 865, 787, 731, 697, 671. ^{1}H NMR (400 MHz, $CDCl_3$) δ 7.84 (d, *J* = 8.4 Hz, 2H, 2 × Ar–H), 7.33–7.29 (m, 2H, 2 × Ar–H), 7.28–7.26 (m, 2H, 2 × Ar–H), 7.25–7.19 (m, 3H, 3 × Ar–H), 6.43 (s, 1H, NH), 4.22 (d, *J* = 13.8 Hz, 1H, NHC*H*H), 3.94 (d, *J* = 13.8 Hz, 1H, NHCH*H*), 2.43 (s, 3H, Ar–CH_3), 1.40 (s, 9H, $C(CH_3)_3$). ^{13}C NMR (101 MHz, $CDCl_3$) δ 157.8 (C=O), 144.6 (Ar–C_q), 136.3 (Ar–C_q), 136.0 (Ar–C_q), 130.3 (2 × Ar–C), 129.2 (2 × Ar–C), 128.5 (2 × Ar–C), 128.45 (2 × Ar–C), 128.42 (Ar–C), 81.0 ($C(CH_3)_3$), 46.1 ($NHCH_2$), 28.6 ($C(CH_3)_3$), 22.0 (Ar–CH_3). HRMS (ESI) m/z Calcd for $C_{19}H_{25}N_2O_3S$ $[M+H]^+$: 361.1586; Found: 361.1593. $[\alpha]^{23}_D$ = +88 (c 0.5, $CHCl_3$). HPLC Conditions:

Chiralpak IA column, 97:3 *n*hexane:*i*PrOH, flow rate: 1 mL min^{-1}, 35 °C, UV detection wavelength: 250 nm. **((*R*)-3c)** Retention time: 41 min.

Synthesis of racemic sample for HPLC analysis prepared according to General Procedure D on a 0.1 mmol scale to afford the sulfonimidamide **(*rac*)-3c** (~10 mg) with characterisation data in accordance with the above. HPLC Conditions: Chiralpak IA column, 97:3 *n*hexane:*i*PrOH, flow rate: 1 mL min^{-1}, 35 °C, UV detection wavelength: 250 nm. **((*R*)-3c)** Retention times: 37 & 42 min.

***tert*-Butyl (*R*)-((allylamino)(oxo)(*p*-tolyl)-λ^6-sulfaneylidene)carbamate ((*R*)-3d)**

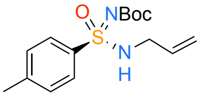

Reaction performed according to General Procedure D. Allylamine (37 μL, 0.50 mmol, 2.0 equiv) and triethylamine (70 μL, 0.50 mmol, 2.0 equiv) were added to a stirred solution of sulfonimidoyl fluoride **(*R*)-2a** (69 mg, 0.25 mmol, 1 equiv) and flame-dried LiBr (43 mg, 0.50 mmol, 2 equiv) in MeCN (0.83 mL, 0.3 M) at RT and warmed to 80 °C for 24 h. The solvent was removed under reduced pressure. Purification by flash column chromatography (SiO_2, 5% Et_2O in CH_2Cl_2) afforded sulfonimidamide **(*R*)-3d** (45.9 mg, 60%, 98% *ee*) as a white solid. mp = 95–96 °C. R_f 0.21 (10% Et_2O in CH_2Cl_2). IR (film)/cm^{-1} 3071, 2978, 2930, 1681, 1595, 1453, 1278, 1159, 1118, 1092, 1062, 924, 813. ^{1}H NMR (400 MHz, $CDCl_3$) δ 7.83 (d, *J* = 8.4 Hz, 2H, 2 × Ar–H), 7.31 (d, *J* = 7.9 Hz, 2H, 2 × Ar–H), 5.74 (ddt, *J* = 17.1, 10.3, 5.8 Hz, 1H, NCH_2CH), 5.21 (dd, *J* = 17.1, 1.3 Hz, 1H, $NHCH_2CHCH$H), 5.11 (dd, *J* = 10.2, 1.3 Hz, 1H, $NHCH_2CHCHH$), 3.62 (ddt, *J* = 15.0, 5.6, 1.6 Hz, 1H, NHC*H*H), 3.42 (ddt, *J* = 14.9, 6.0, 1.5 Hz, 1H, NHCH*H*), 2.42 (s, 3H, Ar–CH_3), 1.37 (s, 9H, $C(CH_3)_3$). ^{13}C NMR (101 MHz, $CDCl_3$) δ 157.2 (C=O), 144.1 (Ar–C_q), 135.8 (Ar–C_q), 132.8 ($NHCH_2CH=CH_2$), 129.9 (2 × Ar–C), 128.1 (2 × Ar–C), 118.0 ($NHCH_2CH=CH_2$), 80.5 ($C(CH_3)_3$), 44.1 ($NHCH_2$), 28.2 ($C(CH_3)_3$), 21.7 (Ar–CH_3). HRMS (ESI) *m/z* Calcd for $C_{20}H_{28}N_5O_3S$ [M+H]$^+$: 418.1913; Found: 418.1899. $[\alpha]^{23}_D$ = +40 (c 0.5, $CHCl_3$). HPLC conditions: Chiralpak IA column, 97:3 *n*hexane:*i*PrOH, flow rate: 1 mL min^{-1}, 35 °C, UV detection wavelength: 270 nm, **(*R*)-3d** retention times 23 min.

Synthesis of racemic sample for HPLC analysis prepared according to General Procedure D on a 0.1 mmol scale to afford the sulfonimidamide **(*rac*)-3d** (~10 mg) with characterisation data in accordance with the above. HPLC conditions: Chiralpak IA column, 97:3 *n*hexane:*i*PrOH, flow rate: 1 mL min^{-1}, 35 °C, UV detection wavelength: 270 nm, **(*rac*)-3d** retention times 23 & 26 min.

***tert*-Butyl (*R*)-(((cyclopropylmethyl)amino)(oxo)(*p*-tolyl)-λ^6-sulfaneylidene)carbamate ((*R*)-3e)**

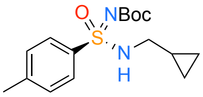

Reaction performed according to General Procedure D. Cyclopropylmethanamine (44 μL, 0.50 mmol, 2.0 equiv) and triethylamine (70 μL, 0.50 mmol, 2.0 equiv) were added to a stirred solution of sulfonimidoyl fluoride **(*R*)-2a** (69 mg, 0.25 mmol, 1 equiv) and flame-dried LiBr (43 mg, 0.50 mmol, 2 equiv) in MeCN (0.83 mL, 0.3 M) at RT and warmed to 80 °C for 24 h. The solvent was removed under reduced pressure. Purification by flash column chromatography (SiO_2, 5% Et_2O in CH_2Cl_2) afforded sulfonimidamide **(*R*)-3e** (50.0 mg, 62%, 95% *ee*) as a white solid. mp = 124–126 °C. R_f 0.26 (5% Et_2O in CH_2Cl_2). IR (film)/cm^{-1} 3086, 2974, 2926, 2873, 1674 (C=O), 1595, 1454, 1252, 1156, 1111, 1044, 809, 731. ^{1}H NMR (400 MHz, $CDCl_3$) δ 7.83 (d, *J* = 8.1 Hz, 2H, 2 × Ar–H), 7.30 (d, *J* = 8.1 Hz, 2H, 2 × Ar–H), 6.56 (s, 1H, NH), 2.89 (dd, *J* = 12.8, 7.0 Hz, 1H, NHC*H*H), 2.62 (dd, *J* = 12.8, 7.3 Hz, 1H, NHCH*H*), 2.42 (s, 3H, Ar–CH_3), 1.37 (s, 9H, $C(CH_3)_3$), 0.95–0.86 (m, 1H, $NHCH_2CH$), 0.50–0.41 (m, 2H, 2 × CHC*H*H), 0.16–0.05 (m, 2H, 2 × CHCH*H*). ^{13}C NMR (101 MHz, $CDCl_3$) δ 157.2 (C=O), 143.9 (Ar–C_q), 135.9 (Ar–C_q), 129.8 (2 × Ar–C),

128.1 (2 × Ar–C), 80.3 (*C*(CH_3)$_3$), 46.6 (NCH_2), 28.2 (C(*C*H_3)$_3$), 21.7 (Ar–CH_3), 10.7 (NCH_2*C*H), 3.9 (1 × CH*C*H_2), 3.6 (1 × CH*C*H_2). HRMS (ESI) *m/z* Calcd for $C_{16}H_{25}N_2O_3S$ $[M+H]^+$: 325.1586; Found: 325.1590. $[\alpha]^{21}_D$ = +29 (c 1.0, $CHCl_3$). HPLC conditions: Chiralpak IA column, 97:3 *n*hexane:*i*PrOH, flow rate: 1 mL min^{-1}, 35 °C, UV detection wavelength: 280 nm, **(*R*)-3e** retention time 26 min.

Synthesis of racemic sample for HPLC analysis prepared according to General Procedure B to afford sulfonimidamide **(*rac*)-3e** (34.1 mg, 65%) as a yellow solid with characterisation data in accordance with the above. HPLC conditions: Chiralpak IA column, 97:3 *n*hexane:*i*PrOH, flow rate: 1 mL min^{-1}, 35 °C, UV detection wavelength: 280 nm, **(*rac*)-3e** retention times 26 & 28 min.

***tert*-Butyl (*R*)-((cyclobutylamino)(oxo)(*p*-tolyl)-λ^6-sulfaneylidene)carbamate ((*R*)-3f)**

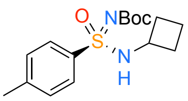

Reaction performed according to General Procedure D. Cyclobutylamine (43 µL, 0.50 mmol, 2.0 equiv) and triethylamine (70 µL, 0.50 mmol, 2.0 equiv) were added to a stirred solution of sulfonimidoyl fluoride **(*R*)-2a** (69 mg, 0.25 mmol, 1 equiv) and flame-dried LiBr (43 mg, 0.50 mmol, 2 equiv) in MeCN (0.83 mL, 0.3 M) at RT and warmed to 80 °C for 24 h. The solvent was removed under reduced pressure. Purification by flash column chromatography (SiO_2, 5% Et_2O in CH_2Cl_2) afforded sulfonimidamide **(*R*)-3f** (61.9 mg, 76%, >99% *ee*) as a white solid. mp = 131–133 °C. R_f 0.18 (5% Et_2O in CH_2Cl_2). IR (film)/cm^{-1} 3068, 2978, 2870, 1674, 1595, 1450, 1390, 1275, 1245, 1141, 1096, 973, 906, 857, 731. ^1H NMR (400 MHz, $CDCl_3$) δ 7.82 (d, *J* = 8.3 Hz, 2H, 2 × Ar–H), 7.29 (d, *J* = 8.1 Hz, 2H, 2 × Ar–H), 6.77 (s, 1H, NH), 3.75–3.59 (m, 1H, NHC*H*), 2.41 (s, 3H, Ar–CH_3), 2.30–2.22 (m, 1H, 1 × NHCHC*H*H), 2.11–2.00 (m, 1H, 1 × NHCHC*H*H), 1.89–1.77 (m, 2H, 2 × NHCHCH*H*), 1.66–1.49 (m, 2H, NHCHCH_2C*H*$_2$), 1.36 (s, 9H, C(CH_3)$_3$). ^{13}C NMR (101 MHz, $CDCl_3$) δ 157.3 (C=O), 143.9 (Ar–C_q), 136.7 (Ar–C_q), 129.7 (2 × Ar–C), 128.0 (2 × Ar–C), 80.4 (*C*(CH_3)$_3$), 46.9 (NHCH), 32.0 (1 × NHCH*C*H_2), 31.3 (1 × NHCH*C*H_2), 28.2 (C(*C*H_3)$_3$), 21.7 (Ar–CH_3), 15.4 (NHCHCH_2*C*H_2). HRMS (ESI) *m/z* Calcd for $C_{16}H_{25}N_2O_3S$ $[M+H]^+$: 325.1586; Found: 325.1587. $[\alpha]^{21}_D$ = +48 (c 0.8, $CHCl_3$). HPLC Conditions: Chiralpak IA column, 95:5 *n*hexane:*i*PrOH, flow rate: 1 mL min^{-1}, 35 °C, UV detection wavelength: 260 nm, **(*R*)-3f** retention time: 15 min.

Synthesis of racemic sample for HPLC analysis prepared according to General Procedure B to afford sulfonimidamide **(*rac*)-3f** (44.0 mg, 54%) as a white solid with characterisation data in accordance with the above. HPLC Conditions: Chiralpak IA column, 95:5 *n*hexane:*i*PrOH, flow rate: 1 mL min^{-1}, 35 °C, UV detection wavelength: 260 nm, **(*rac*)-3f** retention times: 15 & 18 min.

***tert*-Butyl (*R*)-((cyclohexylamino)(oxo)(*p*-tolyl)-λ^6-sulfaneylidene)carbamate ((*R*)-3g)**

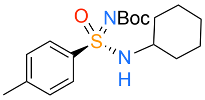

Reaction performed according to General Procedure D. Cyclohexylamine (57 µL, 0.50 mmol, 2.0 equiv) and triethylamine (70 µL, 0.50 mmol, 2.0 equiv) were added to a stirred solution of sulfonimidoyl fluoride **(*R*)-2a** (69 mg, 0.25 mmol, 1 equiv) and flame-dried LiBr (43 mg, 0.50 mmol, 2 equiv) in MeCN (0.83 mL, 0.3 M) at RT and warmed to 80 °C for 24 h. The solvent was removed under reduced pressure. Purification by flash column chromatography (SiO_2, 5% Et_2O in CH_2Cl_2) afforded sulfonimidamide **(*R*)-3g** (60.8mg, 68%, 97% *ee*) as a white solid. mp = 124–125 °C. R_f 0.32 (10% Et_2O in CH_2Cl_2). IR (film)/cm^{-1} 2933, 2855, 1741, 1684, 1453, 1368, 1278, 1162 1096, 1021, 931, 909, 861, 813, 671. ^1H NMR (400 MHz, $CDCl_3$) δ 7.83 (d, *J* = 8.4 Hz, 2H, 2 × Ar–H), 7.30 (d, *J* = 7.8 Hz, 2H, 2 × Ar–H),

6.03 (d, J = 7.5 Hz, 1H, NH), 3.19–3.02 (m, 1H, NHC*H*), 2.43 (s, 3H, Ar–CH_3), 2.04–1.97 (m, 1H, 1 × NHCHC*H*H), 1.76–1.63 (m, 1H, 1 × NHCHC*H*H), 1.55–1.45 (m, 2H, 2 × NHCHCH*H*), 1.40 (s, 9H, $C(CH_3)_3$), 1.36–1.22 (m, 2H, 2 × $NHCHCH_2C$*H*H), 1.20–1.04 (m, 4H, 2 × $NCHCH_2CH$*H* & $NCHCH_2CH_2C$*H_2*). ^{13}C NMR (101 MHz, $CDCl_3$) δ 157.5 (C=O), 143.9 (Ar–C_q), 137.2 (Ar–C_q), 129.8 (2 × Ar–C), 128.0 (2 × Ar–C), 80.4 (*C*$(CH_3)_3$), 51.6 (NHCH), 34.6 (1 × NHCH*CH_2*), 33.4 (1 × NHCH*CH_2*), 28.3 (C(*CH_3*)$_3$), 25.3 (2 × $NCHCH_2$*CH_2*), 24.7 ($NCHCH_2CH_2$*CH_2*), 21.7 (Ar–CH_3). HRMS (ESI) m/z Calcd for $C_{18}H_{29}N_2O_3S$ $[M+H]^+$: 353.1899; Found: 353.1906. $[\alpha]^{23}_D$ = +42 (c 0.5, $CHCl_3$). HPLC conditions: Chiralpak IF column, 95:5 *n*hexane:*i*PrOH, flow rate: 1 mL min^{-1}, 35 °C, UV detection wavelength: 254 nm, **(*R*)-3g** retention times 24 min.

Synthesis of racemic sample for HPLC analysis prepared according to General Procedure D on a 0.1 mmol scale to afford the sulfonimidamide **(*rac*)-3g** (~10 mg) with characterisation data in accordance with the above. HPLC conditions: Chiralpak IF column, 95:5 *n*hexane:*i*PrOH, flow rate: 1 mL min^{-1}, 35 °C, UV detection wavelength: 254 nm, **(*rac*)-3g** retention times 19 & 24 min.

***tert*-Butyl (*R*)-((dimethylamino)(oxo)(*p*-tolyl)-λ[6]-sulfaneylidene)carbamate ((*R*)-3h)**

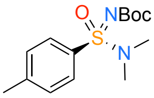

Reaction performed according to General Procedure D. Dimethylamine hydrochloride (41 mg, 0.50 mmol, 2.0 equiv) and triethylamine (140 μL, 1.00 mmol, 4.0 equiv) were added to a stirred solution of sulfonimidoyl fluoride **(*R*)-2a** (69 mg, 0.25 mmol, 1 equiv) and flame-dried LiBr (43 mg, 0.50 mmol, 2 equiv) in MeCN (0.83 mL, 0.3 M) at RT and warmed to 80 °C for 24 h. The solvent was removed under reduced pressure. Purification by flash column chromatography (SiO_2, 20% EtOAc/pentane) afforded sulfonimidamide **(*R*)-3g** (64.2 mg, 86%, 96% *ee*) as a white solid. mp = 115–116 °C. R_f 0.16 (20% EtOAc in pentane). IR (film)/cm^{-1} 3027, 2974, 2922, 2878, 1692, 1592, 1476, 1390, 1275, 1156, 1040, 943, 820, 775. ^{1}H NMR (400 MHz, $CDCl_3$) δ 7.75 (d, J = 8.3 Hz, 2H, 2 × Ar–H), 7.32 (d, J = 8.2 Hz, 2H, 2 × Ar–H), 2.75 (s, 6H, $N(CH_3)_2$), 2.42 (s, 3H, Ar–CH_3), 1.40 (s, 9H, $C(CH_3)_3$). ^{13}C NMR (101 MHz, $CDCl_3$) δ 156.7 (C=O), 144.0 (Ar–C_q), 132.6 (Ar–C_q), 129.8 (2 × Ar–C), 127.9 (2 × Ar–C), 80.3 (*C*$(CH_3)_3$), 37.8 ($N(CH_3)_2$), 28.2 (C(*CH_3*)$_3$), 21.6 (Ar–CH_3). HRMS (ESI) m/z Calcd for $C_{14}H_{23}N_2O_3S$ $[M+H]^+$: 299.1429; Found: 299.1437. $[\alpha]^{21}_D$ = –28 (c 1.0, $CHCl_3$). HPLC Conditions: Chiralpak IA column, 97:3 *n*hexane:*i*PrOH, flow rate: 1 mL min^{-1}, 35 °C, UV detection wavelength: 250 nm, **(*R*)-3g** retention time: 25 min.

Synthesis of racemic sample for HPLC analysis prepared according to General Procedure B to afford sulfonimidamide **(*rac*)-3g** (56.9 mg, 74%) as a colourless oil with characterisation data in accordance with the above. HPLC Conditions: Chiralpak IA column, 97:3 *n*hexane:*i*PrOH, flow rate: 1 mL min^{-1}, 35 °C, UV detection wavelength: 250 nm, **(*rac*)-3g** retention times: 23 & 25 min.

***tert*-Butyl (*R*)-((benzyl(methyl)amino)(oxo)(*p*-tolyl)-λ[6]-sulfaneylidene)carbamate ((*R*)-3i)**

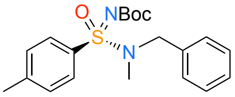

Reaction performed according to General Procedure D. *N*-methyl benzylamine (65 μL, 0.50 mmol, 2.0 equiv) and triethylamine (70 μL, 0.50 mmol, 2.0 equiv) were added to a stirred solution of sulfonimidoyl fluoride **(*R*)-2a** (69 mg, 0.25 mmol, 1 equiv) and flame-dried LiBr (43 mg, 0.50 mmol, 2 equiv) in MeCN (0.83 mL, 0.3 M) at RT and warmed to 80 °C for 24 h. The solvent was removed under reduced pressure. Purification by flash column chromatography (SiO_2, 5% Et_2O in CH_2Cl_2) afforded sulfonimidamide **(*R*)-3i** (50.4 mg, 54%, 99% *ee*) as a white solid. mp = 95–97 °C. R_f = 0.28 (5% Et_2O in CH_2Cl_2). IR (film)/cm^{-1} 2971, 2926, 1666, 1450, 1282, 1248, 1148, 1085, 992, 936, 895, 816, 753. ^{1}H NMR (400 MHz,

$CDCl_3$) δ 7.87 (d, *J* = 8.1 Hz, 2H, 2 × Ar–H), 7.38 (d, *J* = 8.1 Hz, 2H, 2 × Ar–H), 7.36–7.27 (m, 5H, 5 × Ar–H), 4.41 (d, *J* = 14.1 Hz, 1H, NC*H*H), 4.17 (d, *J* = 14.0 Hz, 1H, NCH*H*), 2.67 (s, 3H, NCH_3), 2.47 (s, 3H, Ar–CH_3), 1.47 (s, 9H, $C(CH_3)_3$). ^{13}C NMR (101 MHz, $CDCl_3$) δ 157.0 (C=O), 144.4 (Ar–C_q), 136.1 (Ar–C_q), 134.5 (Ar–C_q), 130.2 (2 × Ar–C), 129.1 (2 × Ar–C), 128.9 (2 × Ar–C), 128.3 (Ar–C), 128.2 (2 × Ar–C), 80.7 (*C*$(CH_3)_3$), 54.2 (NCH_2), 34.7 (NCH_3), 28.6 ($C(CH_3)_3$), 22.0 (Ar–CH_3). HRMS (APCI) m/z Calcd for $C_{20}H_{27}N_2O_3S$ $[M+H]^+$: 375.1737; Found: 375.1735. $[\alpha]^{23}_D$ = –12 (c 1, $CHCl_3$). HPLC Conditions: Chiralpak IA column, 95:5 *n*hexane:*i*PrOH, flow rate: 1 mL min^{-1}, 35 °C, UV detection wavelength: 250 nm. **(*R*)-3i** retention time: 33 min.

Synthesis of racemic sample for HPLC analysis prepared according to General Procedure B to afford sulfonimidamide **(*rac*)-3i** (56.9 mg, 61%) as a colourless oil with characterisation data in accordance with the above. HPLC Conditions: Chiralpak IA column, 95:5 *n*hexane:*i*PrOH, flow rate: 1 mL min^{-1}, 35 °C, UV detection wavelength: 250 nm. **(*rac*)-3i** retention times: 21 & 33 min.

***tert*-Butyl (*R*)-((3,4-dihydroisoquinolin-2(1*H*)-yl)(oxo)(*p*-tolyl)-λ^6-sulfaneylidene)carbamate ((*R*)-3j)**

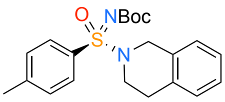

Reaction performed according to General Procedure D. 1,2,3,4-tetrahydroisoquinoline (63 μL, 0.50 mmol, 2.0 equiv) and triethylamine (70 μL, 0.50 mmol, 2.0 equiv) were added to a stirred solution of sulfonimidoyl fluoride **(*R*)-2a** (69 mg, 0.25 mmol, 1 equiv) and flame-dried LiBr (43 mg, 0.50 mmol, 2 equiv) in MeCN (0.83 mL, 0.3 M) at RT and warmed to 80 °C for 24 h. The solvent was removed under reduced pressure. Purification by flash column chromatography (SiO_2, 20% EtOAc in pentane) afforded sulfonimidamide **(*R*)-3j** (90.3 mg, 93%, 97% *ee*) as a pale-yellow oil. R_f 0.36 (20% EtOAc in pentane). IR (film)/cm^{-1} 2974, 2930, 1670 (C=O), 1595, 1495, 1364, 1249, 1152, 951, 895, 727. ^1H NMR (400 MHz, $CDCl_3$) δ 7.82 (d, *J* = 8.4 Hz, 2H, 2 × Ar–H), 7.31 (d, *J* = 8.1 Hz, 2H, 2 × Ar–H), 7.14–7.10 (m, 2H, 2 × Ar–H), 7.08–7.00 (m, 2H, 2 × Ar–H), 4.41–4.32 (m, 2H, NCH_2), 3.55 (dt, *J* = 11.7, 5.7 Hz, 1H, NC*H*H), 3.40 (dt, *J* = 17.7, 5.9 Hz, 1H, NCH*H*), 2.92–2.88 (m, 2H, NCH_2CH_2), 2.40 (s, 3H, Ar–CH_3), 1.40 (s, 9H, $C(CH_3)_3$). ^{13}C NMR (101 MHz, $CDCl_3$) δ 156.6 (C=O), 144.1 (Ar–C_q), 133.5 (Ar–C_q), 133.2 (Ar–C_q), 131.8 (Ar–C_q), 129.8 (2 × Ar–C), 128.8 (Ar–C), 127.9 (2 × Ar–C), 126.8 (Ar–C), 126.4 (Ar–C), 126.4 (Ar–C), 80.4 (*C*$(CH_3)_3$), 47.4 (NCH_2), 43.5 (NCH_2CH_2), 29.0 (NCH_2CH_2), 28.1 ($C(CH_3)_3$), 21.6 (Ar–CH_3). HRMS (ESI) *m/z* Calcd for $C_{21}H_{27}N_2O_3S$ $[M+H]^+$: 387.1742; Found: 387.1747. $[\alpha]^{23}_D$ = 0 (c 1.0, $CHCl_3$). HPLC Conditions Chiralpak IA column, 95:5 *n*hexane:*i*PrOH, flow rate: 1 mL min^{-1}, 35 °C, UV detection wavelength: 250 nm, **(*R*)-3j** retention times: 33 min.

Synthesis of racemic sample for HPLC analysis prepared according to General Procedure B to afford sulfonimidamide **(*rac*)-3j** (65.3 mg, 68%) as a colourless oil with characterisation data in accordance with the above. HPLC Conditions Chiralpak IA column, 95:5 *n*hexane:*i*PrOH, flow rate: 1 mL min^{-1}, 35 °C, UV detection wavelength: 250 nm, **(*rac*)-3j** retention times: 26 & 33 min.

***tert*-Butyl (*R*)-(oxo(pyrrolidin-1-yl)(*p*-tolyl)-λ^6-sulfaneylidene)carbamate ((*R*)-3k)**

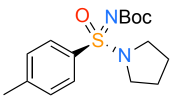

Reaction performed according to General Procedure D. Pyrrolidine (42 μL, 0.50 mmol, 2.0 equiv) and triethylamine (70 μL, 0.50 mmol, 2.0 equiv) were added to a stirred solution of sulfonimidoyl fluoride **(*R*)-2a** (69 mg, 0.25 mmol, 1 equiv) and flame-dried LiBr (43 mg, 0.50 mmol, 2 equiv) in MeCN (0.83 mL, 0.3 M) at RT and warmed to 80 °C for 24 h. The solvent was removed

under reduced pressure. Purification by flash column chromatography (SiO_2, 5% Et_2O in CH_2Cl_2) afforded sulfonimidamide **(*R*)-3k** (75.7 mg, 93%, 97% *ee*) as a white solid. mp = 129–131 °C. R_f 0.25 (5% Et_2O in CH_2Cl_2). IR (film)/cm^{-1} 3068, 2975, 2926, 2866, 1674 (C=O), 1595, 1457, 1275, 1156, 1059, 887, 727. ^1H NMR (400 MHz, $CDCl_3$) δ 7.78 (d, *J* = 8.3 Hz, 2H, 2 × Ar–H), 7.29 (d, *J* = 8.3 Hz, 2H, 2 × Ar–H), 3.32–3.19 (m, 4H, 2 × NCH_2), 2.40 (s, 3H, Ar–CH_3), 1.82–1.76 (m, 4H, 2 × NCH_2CH_2), 1.38 (s, 9H, $C(CH_3)_3$). ^{13}C NMR (101 MHz, $CDCl_3$) δ 156.9 (C=O), 143.7 (Ar–C_q), 134.3 (Ar–C_q), 129.8 (2 × Ar–C), 127.7 (2 × Ar–C), 80.0 ($C(CH_3)_3$), 47.9 (2 × NCH_2), 28.1 ($C(CH_3)_3$), 25.4 (2 × NCH_2CH_2), 21.6 (Ar–CH_3). HRMS (ESI) *m/z* Calcd for $C_{16}H_{25}N_2O_3S$ $[M+H]^+$: 325.1586; Found: 325.1594. $[\alpha]^{21}_D$ = –9 (c 1.0, $CHCl_3$). HPLC conditions: Chiralpak IA column, 95:5 *n*hexane:*i*PrOH, flow rate: 1 mL min^{-1}, 35 °C, UV detection wavelength: 260 nm, **(*R*)-3k** retention times: 20 min.

Synthesis of racemic sample for HPLC analysis prepared according to General Procedure B to afford sulfonimidamide **(*rac*)-3k** (51.5 mg, 64%) as a white solid with characterisation data in accordance with the above. HPLC conditions: Chiralpak IA column, 95:5 *n*hexane:*i*PrOH, flow rate: 1 mL min^{-1}, 35 °C, UV detection wavelength: 250 nm, **(*rac*)-3k** retention times: 19 & 20 min.

***tert*-Butyl (*R*)-(morpholino(oxo)(*p*-tolyl)-λ⁶-sulfaneylidene)carbamate ((*R*)-3l)**

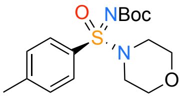

Reaction performed according to General Procedure D. Morpholine (50 μL, 0.50 mmol, 2.0 equiv) and triethylamine (70 μL, 0.50 mmol, 2.0 equiv) were added to a stirred solution of sulfonimidoyl fluoride **(*R*)-2a** (69 mg, 0.25 mmol, 1 equiv) and LiBr (43 mg, 0.50 mmol, 2 equiv) in MeCN (0.83 mL, 0.3 M) at RT and warmed to 80 °C for 24 h. The solvent was removed under reduced pressure. Purification by flash column chromatography (SiO_2, 5% Et_2O in CH_2Cl_2) afforded sulfonimidamide **(*R*)-3l** (74.9 mg, 88%, >99% *ee*) as a white solid. R_f 0.21 (5% Et_2O in CH_2Cl_2). mp = 136–138 °C. IR (film)/cm^{-1} 2974, 2859, 1677 (C=O), 1595, 1464, 1278, 1159, 1115, 939, 816. ^1H NMR (400 MHz, $CDCl_3$) δ 7.72 (d, *J* = 8.3 Hz, 2H, 2 × Ar–H), 7.33 (d, *J* = 8.1 Hz, 2H, 2 × Ar–H), 3.72–3.70 (m, 4H, 2 × OCH_2), 3.09–3.08 (m, 4H, 2 × NCH_2), 2.43 (s, 3H, Ar–/CH_3), 1.39 (s, 9H, $C(CH_3)_3$). ^{13}C NMR (101 MHz, $CDCl_3$) δ 156.5 (C=O), 144.4 (Ar–C_q), 132.1 Ar–C_q), 130.0 (2 × Ar–C), 128.0 (2 × Ar–C), 80.5 ($C(CH_3)_3$), 66.2 (2 × OCH_2), 45.8 (2 × NCH_2), 28.1 ($C(CH_3)_3$)), 21.7 (Ar–CH_3). HRMS (ESI) *m/z* Calcd for $C_{16}H_{25}N_2O_4S$ $[M+H]^+$: 341.1535; Found: 341.1540. $[\alpha]^{21}_D$ = –11 (c 1.0, $CHCl_3$). HPLC Conditions: Chiralpak IA column, 95:5 *n*hexane:*i*PrOH, flow rate: 1 mL min^{-1}, 35 °C, UV detection wavelength: 250 nm, **(*R*)-3l** retention time: 31 min.

Synthesis of racemic sample for HPLC analysis prepared according to General Procedure B to afford sulfonimidamide **(*rac*)-3l** (66.2 mg, 78%) as a white solid with characterisation data in accordance with the above. HPLC Conditions: Chiralpak IA column, 95:5 *n*hexane:*i*PrOH, flow rate: 1 mL min^{-1}, 35 °C, UV detection wavelength: 250 nm, **(*rac*)-3l** retention time: 25 & 31 min.

***tert*-Butyl (*R*)-(oxo(4-oxopiperidin-1-yl)(*p*-tolyl)-λ⁶-sulfaneylidene)carbamate ((*R*)-3m)**

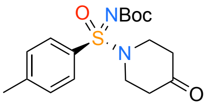

Reaction performed according to General Procedure D. 4-piperidone hydrochloride salt (78 mg, 0.50 mmol, 2.0 equiv) and triethylamine (140 μL, 1.00 mmol, 4.0 equiv) were added to a stirred solution of sulfonimidoyl fluoride **(*R*)-2a** (69 mg, 0.25 mmol, 1 equiv) and flame-dried LiBr (43 mg, 0.50 mmol, 2 equiv) in MeCN (0.83 mL, 0.3 M) at RT and warmed to 80 °C for 24 h.

The solvent was removed under reduced pressure. Purification by flash column chromatography (SiO_2, 5% Et_2O in CH_2Cl_2) afforded sulfonimidamide **(*R*)-3m** (30.4 mg, 35%, 97% *ee*) as a white solid. mp = 145–146 °C. R_f 0.28 (10% Et_2O in CH_2Cl_2). IR (film)/cm^{-1} 2974, 2926, 2870, 1718, 1670, 1595, 1368, 1341, 1274, 1252, 1156, 1111, 924, 816, 764, 708, 667. ^{1}H NMR (400 MHz, $CDCl_3$) δ 7.79 (d, *J* = 8.4 Hz, 2H, 2 × Ar–H), 7.34 (d, *J* = 7.7 Hz, 2H, 2 × Ar–H), 3.57–3.46 (m, 4H, 2 × NCH_2), 2.53 (t, *J* = 6.2 Hz, 4H, 2 × NCH_2CH_2), 2.44 (s, 3H, Ar–CH_3), 1.42 (s, 9H, $C(CH_3)_3$). ^{13}C NMR (101 MHz, $CDCl_3$) δ 206.2 (C=O), 156.9 (C=O), 144.9 (Ar–C_q), 134.0 (Ar–C_q), 130.5 (2 × Ar–C), 128.1 (2 × Ar–C), 81.1 ($C(CH_3)_3$), 45.9 (NCH_2), 41.1 (NCH_2CH_2), 28.5 ($C(CH_3)_3$), 22.0 (Ar–CH_3). HRMS (ESI) m/z Calcd for $C_{17}H_{25}N_2O_4S$ $[M+H]^+$: 353.1535; Found: 353.1527. $[\alpha]^{23}_D$ = 0 (c 0.2, $CHCl_3$). HPLC Conditions: Chiralpak IA column, 95:5 *n*hexane:*i*PrOH, flow rate: 1 mL min^{-1}, 35 °C, UV detection wavelength: 254 nm. **(*R*)-3m** retention times: 43 min.

Synthesis of racemic sample for HPLC analysis prepared according to General Procedure D on a 0.1 mmol scale to afford the sulfonimidamide **(*rac*)-3m** (~10 mg) with characterisation data in accordance with the above. HPLC Conditions: Chiralpak IA column, 95:5 *n*hexane:*i*PrOH, flow rate: 1 mL min^{-1}, 35 °C, UV detection wavelength: 254 nm. **(*rac*)-3m** retention times: 37 & 43 min.

tert-Butyl (*R*)-((4,4-difluoropiperidin-1-yl)(oxo)(*p*-tolyl)-λ^6-sulfaneylidene)carbamate ((*R*)-3n)

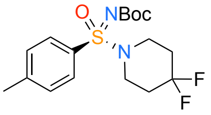

Reaction performed according to General Procedure D. 4,4-difluoropiperidine (61 mg, 0.50 mmol, 2.0 equiv) and triethylamine (70 µL, 0.50 mmol, 2.0 equiv) were added to a stirred solution of sulfonimidoyl fluoride **(*R*)-2a** (69 mg, 0.25 mmol, 1 equiv) and flame-dried LiBr (43 mg, 0.50 mmol, 2 equiv) in MeCN (0.83 mL, 0.3 M) at RT and warmed to 80 °C for 24 h. The solvent was removed under reduced pressure. Purification by flash column chromatography (SiO_2, 5% Et_2O in CH_2Cl_2) afforded sulfonimidamide **(*R*)-3n** (70.0 mg, 75%, 99% *ee*) as a white solid. mp = 152–154 °C. R_f 0.80 (5% Et_2O in CH_2Cl_2). IR (film)/cm^{-1} 2978, 2870, 1670, 1364, 1249, 1148, 1118, 1036, 996, 913, 865, 816, 768, 708. ^{1}H NMR (400 MHz, $CDCl_3$) δ 7.75 (d, *J* = 8.4 Hz, 2H, 2 × Ar–H), 7.34 (d, *J* = 7.7 Hz, 2H, 2 × Ar–H), 3.29 (dd, *J* = 12.8, 6.5 Hz, 4H, 2 × NCH_2), 2.44 (s, 3H, Ar–CH_3), 2.06 (td, *J* = 13.4, 6.5 Hz, 4H, 2 × NCH_2CH_2), 1.41 (s, 9H, $C(CH_3)_3$). ^{13}C NMR (101 MHz, $CDCl_3$) δ 156.8 (C=O), 144.8 (Ar–C_q), 133.8 (Ar–C_q), 130.4 (2 × Ar–C), 128.0 (2 × Ar–C), 81.0 ($C(CH_3)_3$), 43.4 (t, *J* = 5.7 Hz, NCH_2), 34.0 (t, *J* = 23.9 Hz, (NCH_2CH_2), 28.6 ($C(CH_3)_3$), 22.0 (Ar–CH_3). ^{19}F NMR (377 MHz, $CDCl_3$) δ -99.2. HRMS (ESI) m/z Calcd for $C_{17}H_{25}F_2N_2O_3S$ $[M+H]^+$: 375.1554; Found: 375.1548. $[\alpha]^{23}_D$ = –6 (c 1, $CHCl_3$). HPLC conditions: Chiralpak IB column, 95:5 *n*hexane:*i*PrOH, flow rate: 1 mL min^{-1}, 35 °C, UV detection wavelength: 250 nm, **(*R*)-3n** retention times 10 min.

Synthesis of racemic sample for HPLC analysis prepared according to General Procedure D on a 0.1 mmol scale to afford the sulfonimidamide **(*rac*)-3n** (~10 mg) with characterisation data in accordance with the above. HPLC conditions: Chiralpak IB column, 95:5 *n*hexane:*i*PrOH, flow rate: 1 mL min^{-1}, 35 °C, UV detection wavelength: 250 nm, **(*rac*)-3n** retention times 10 & 12 min.

tert-Butyl (*R*)-((4-hydroxypiperidin-1-yl)(oxo)(*p*-tolyl)-λ^6-sulfaneylidene)carbamate ((*R*)-3o)

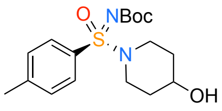

Reaction performed according to General Procedure D. Triethylamine (70 µL, 0.50 mmol, 2.0 equiv) were added to a stirred solution of sulfonimidoyl fluoride **(*R*)-2a** (69 mg, 0.25 mmol, 1 equiv), 4-piperidinol (51 mg, 0.50 mmol, 2.0 equiv) and flame-dried LiBr (43 mg, 0.50 mmol, 2 equiv) in MeCN (0.83 mL, 0.3 M) at RT and warmed to 80 °C for 24 h. The solvent was

removed under reduced pressure. Purification by flash column chromatography (SiO_2, 5% Et_2O in CH_2Cl_2) afforded sulfonimidamide ***(R)*-3o** (50.0 mg, 56%, 99% *ee*) as a viscous oil. R_f 0.11 (5% MeOH in CH_2Cl_2). IR (film)/cm^{-1} 3433, 2978, 2930, 2866, 1670, 1454, 1388, 1252, 1156, 1088, 1036, 917, 865, 813, 731, 667. ^{1}H NMR (400 MHz, $CDCl_3$) δ 7.70 (d, *J* = 8.4 Hz, 2H, 2 × Ar–H), 7.30 (d, *J* = 7.8 Hz, 2H. 2 × Ar–C), 3.75 (tt, *J* = 7.5, 3.7 Hz, 1H, C*H*OH), 3.45–3.29 (m, 2H, 2 × NC*H*H), 2.97–2.91 (m, 2H, 2 × NCH*H*), 2.41 (s, 3H, Ar–CH_3), 2.08 (bs, 1H, OH), 1.93–1.82 (m, 2H, 2 × NCH_2C*H*H), 1.67–1.53 (m, 2H, 2 × NCH_2CH*H*), 1.37 (s, 9H, $C(CH_3)_3$). ^{13}C NMR (101 MHz, $CDCl_3$) δ 157.1 (C=O), 144.3 (Ar–C_q), 133.7 (Ar–C_q), 130.2 (2 × Ar–C), 128.1 (2 × Ar–C), 80.6 (*C*$(CH_3)_3$), 66.3 (CHOH), 43.3 (NCH_2), 33.7 (NCH_2*C*H_2), 28.5 (C(*C*$H_3)_3$), 21.9 (Ar–CH_3). HRMS (ESI) m/z Calcd for $C_{17}H_{27}N_2O_4S$ $[M+H]^+$: 355.1692; Found: 355.1691. $[\alpha]^{23}_D$ = –4 (c 1, $CHCl_3$). HPLC conditions: Chiralpak IA column, 85:15 *n*hexane:*i*PrOH, flow rate: 1 mL min^{-1}, 35 °C, UV detection wavelength: 254 nm, ***(R)*-3o** retention time: 13 min.

Synthesis of racemic sample for HPLC analysis prepared according to General Procedure D on a 0.1 mmol scale to afford the sulfonimidamide ***(rac)*-3o** (~10 mg) with characterisation data in accordance with the above. HPLC conditions: Chiralpak IA column, 85:15 *n*hexane:*i*PrOH, flow rate: 1 mL min^{-1}, 35 °C, UV detection wavelength: 254 nm, ***(rac)*-3o** retention times: 11 & 13 min.

***tert*-Butyl (*R*)-4-(*N*-(*tert*-butoxycarbonyl)-4-methylphenylsulfonimidoyl)piperazine-1-carboxylate ((*R*)-3p)**

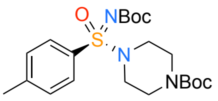

Reaction performed according to General Procedure D. 1-Boc piperazine (93 mg, 0.50 mmol, 2.0 equiv) and triethylamine (70 μL, 0.50 mmol, 2.0 equiv) were added to a stirred solution of sulfonimidoyl fluoride ***(R)*-2a** (69 mg, 0.25 mmol, 1 equiv) and LiBr (43 mg, 0.50 mmol, 2 equiv) in MeCN (0.83 mL, 0.3 M) at RT and warmed to 80 °C for 24 h. The solvent was removed under reduced pressure. Purification by flash column chromatography (SiO_2, 20% EtOAc/pentane) afforded sulfonimidamide ***(R)*-3p** (79.0 mg, 74%, 97% *ee*) as a white solid. mp = 136–138 °C. R_f 0.26 (20% EtOAc in pentane). IR (film)/cm^{-1} 2974, 2930, 2863, 1692, 1595, 1464, 1364, 1249, 1159, 1126, 932, 865, 731. ^{1}H NMR (400 MHz, $CDCl_3$) δ 7.70 (d, *J* = 8.3 Hz, 2H, 2 × Ar–H), 7.32 (d, *J* = 8.3 Hz, 2H, 2 × Ar–H), 3.47 (t, *J* = 5.1 Hz, 4H, 2 × NCH_2), 3.09–3.01 (m, 4H, 2 × NCH_2), 2.41 (s, 3H, Ar–CH_3), 1.39 (s, 9H, $C(CH_3)_3$), 1.38 (s, 9H, $C(CH_3)_3$). ^{13}C NMR (101 MHz, $CDCl_3$) δ 156.5 (C=O), 154.3 (C=O), 144.3 (Ar–C_q), 132.6 (Ar–C_q), 130.0 (2 × Ar–C), 127.9 (2 × Ar–C), 80.5 (*C*$(CH_3)_3$), 80.5 (*C*$(CH_3)_3$), 45.7 (4 × NCH_2), 28.4 (C(*C*$H_3)_3$), 28.1 (C(*C*$H_3)_3$), 21.6 ($ArCH_3$). HRMS (ESI) m/z Calcd for $C_{21}H_{34}N_3O_5S$ $[M+H]^+$: 440.2219; Found: 440.2227. $[\alpha]^{21}_D$ = –6 (c 1.0, $CHCl_3$). HPLC Conditions: Chiralpak IA column, 95:5 *n*hexane:*i*PrOH, flow rate: 1 mL min^{-1}, 35 °C, UV detection wavelength: 250 nm, ***(R)*-3p** retention time: 30 min.

Synthesis of racemic sample for HPLC analysis prepared according to General Procedure B to afford sulfonimidamide ***(rac)*-3p** (81.2 mg, 74%) as a white solid with characterisation data in accordance with the above. HPLC Conditions: Chiralpak IA column, 95:5 *n*hexane:*i*PrOH, flow rate: 1 mL min^{-1}, 35 °C, UV detection wavelength: 250 nm, ***(rac)*-3p** retention times: 27 & 30 min.

tert-Butyl (R)-(oxo(4-(pyrimidin-2-yl)piperazin-1-yl)(p-tolyl)-λ^6-sulfaneylidene)carbamate ((R)-3q)

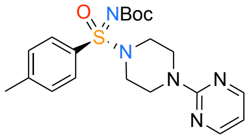

Reaction performed according to General Procedure D. 1-(2-pyrimidyl)piperazine (70 μL, 0.50 mmol, 2.0 equiv) and triethylamine (70 μL, 0.50 mmol, 2.0 equiv) were added to a stirred solution of sulfonimidoyl fluoride **(R)-2a** (69 mg, 0.25 mmol, 1 equiv) and flame-dried LiBr (43 mg, 0.50 mmol, 2 equiv) in MeCN (0.83 mL, 0.3 M) at RT and warmed to 80 °C for 24 h. The solvent was removed under reduced pressure. Purification by flash column chromatography (SiO_2, 5% Et_2O in CH_2Cl_2) afforded sulfonimidamide **(R)-3q** (72.1 mg, 69%, >99% *ee*) as a white solid. mp = 193–196 °C. R_f 0.25 (10% Et_2O in CH_2Cl_2). IR (film)/cm^{-1} 3019, 2978, 2874, 1740, 1681, 1588, 1551, 1491, 1450, 1364, 1260, 1159, 954, 913. ^{1}H NMR (400 MHz, $CDCl_3$) δ 8.29 (d, *J* = 4.7 Hz, 2H, 2 × Ar–H), 7.76 (d, *J* = 8.4 Hz, 2H, 2 × Ar–H), 7.35 (d, *J* = 8.0 Hz, 2H, 2 × Ar–H), 6.52 (t, *J* = 4.7 Hz, 1H, Ar–H), 3.94 (dd, *J* = 6.0, 4.3 Hz, 4H, 2 × NCH_2), 3.19 (m, 4H, NCH_2), 2.44 (s, 3H, Ar–CH_3), 1.43 (s, 9H, $C(CH_3)_3$). ^{13}C NMR (101 MHz, $CDCl_3$) δ161.3 (Ar–C_q), 157.8 (C=O), 156.6 (2 × Ar–C), 144.2 (Ar–C_q), 132.6 (Ar–C_q), 130.0 (2 × Ar–C), 127.9 (2 × Ar–C), 110.6 (Ar–C), 80.5 ($C(CH_3)_3$), 45.8 (2 × NCH_2), 43.2 (2 × NCH_2), 28.2 ($C(CH_3)_3$), 21.6 (Ar–CH_3). HRMS (ESI) m/z Calcd for $C_{20}H_{28}N_5O_3S$ $[M+H]^+$: 418.1913; Found: 418.1905. $[\alpha]^{23}_D$ = 0 (c 1, $CHCl_3$). HPLC Conditions: Chiralpak IB column, 97:3 *n*hexane:*i*PrOH, flow rate: 1 mL min^{-1}, 35 °C, UV detection wavelength: 250 nm. **(R)-3q** retention times: 33 min.

Synthesis of racemic sample for HPLC analysis prepared according to General Procedure D on a 0.1 mmol scale to afford the sulfonimidamide **(rac)-3q** (~10 mg) with characterisation data in accordance with the above. HPLC Conditions: Chiralpak IB column, 97:3 *n*hexane:*i*PrOH, flow rate: 1 mL min^{-1}, 35 °C, UV detection wavelength: 250 nm. **(rac)-3q** retention times: 33 & 38 min.

tert-Butyl (R)-((4-(6-fluorobenzo[d]isoxazol-3-yl)piperidin-1-yl)(oxo)(p-tolyl)-λ^6-sulfaneylidene)carbamate ((R)-3r)

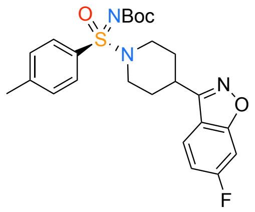

Reaction performed according to General Procedure D. 6-Fluoro-3-(4-piperidinyl)benzisoxazole (110 mg, 0.50 mmol, 2.0 equiv) and triethylamine (70 μL, 0.50 mmol, 2.0 equiv) were added to a stirred solution of sulfonimidoyl fluoride **(R)-2a** (69 mg, 0.25 mmol, 1 equiv) and flame-dried LiBr (43 mg, 0.50 mmol, 2 equiv) in MeCN (0.83 mL, 0.3 M) at RT and warmed to 80 °C for 24 h. The solvent was removed under reduced pressure. Purification by flash column chromatography (SiO_2, 5% Et_2O in CH_2Cl_2) afforded sulfonimidamide **(R)-3r** (88.4 mg, 75%, 98% *ee*) as a pale-yellow oil. R_f 0.27 (5% Et_2O in CH_2Cl_2). IR (film)/cm^{-1} 2974, 2930, 2855, 1737, 1670, 1614, 1446, 1271, 1148, 1111, 1044, 924, 839, 796, 731, 667. ^{1}H NMR (400 MHz, $CDCl_3$) δ 7.80 (d, *J* = 8.4 Hz, 2H, 2 × Ar–H), 7.61 (dd, *J* = 8.8, 5.1 Hz, 1H, Ar–H), 7.35 (d, *J* = 8.0 Hz, 2H, 2 × Ar–H), 7.24 (dd, *J* = 8.4, 2.1 Hz, 1H, Ar–H), 7.06 (td, *J* = 8.8, 2.1 Hz, 1H, Ar–H), 4.06 (dd, *J* = 12.2, 1.9 Hz, 1H, NC*H*H), 3.87 (dd, *J* = 12.1, 1.9 Hz, 1H, NC*H*H), 3.12 (p, *J* = 7.7 Hz, 1H, NCH_2CH_2CH), 2.90–2.79 (m, 1H, NCH*H*), 2.72 (ddd, *J* = 12.1, 8.3, 6.1 Hz, 1H, NCH*H*), 2.45 (s, 3H, Ar–CH_3), 2.12 (tt, *J* = 8.8, 4.6 Hz, 4H, 2 × NCH_2CH_2), 1.43 (s, 9H, $C(CH_3)_3$). ^{13}C NMR (101 MHz, $CDCl_3$) δ 164.8 (d, *J* = 280 Hz, Ar–C_q), 164.3 (Ar–C_q), 160.4 (C=N), 156.6 (C=O), 134.0 (Ar–C_q), 130.3 (2 × Ar–C), 128.2 (2 × Ar–C), 125.5 (Ar–C_q), 122.7 (*J* = 11 Hz, Ar–C), 117.3 (Ar–C_q), 113.2 (d, *J* = 25 Hz, Ar–C), 97.9 (d, *J* = 27 Hz, Ar–C), 80.8 ($C(CH_3)_3$), 46.8 (NCH_2), 45.3 (NCH_2). 34.1 (NCH_2CH_2CH), 30.3 (1 × NCH_2CH_2), 30.0 (1 × NCH_2CH_2), 28.6 ($C(CH_3)_3$, 22.0 (Ar–CH_3). ^{19}F NMR (377 MHz, $CDCl_3$) δ 109.0. HRMS (ESI) m/z Calcd for $C_{24}H_{29}N_3O_4SF$ $[M+H]^+$: 474.1863; Found:

474.1861. $[\alpha]^{23}_D$ = +20 (c 0.5, $CHCl_3$). HPLC conditions: Chiralpak IA column, 90:10 *n*hexane:*i*PrOH, flow rate: 1 mL min^{-1}, 35 °C, UV detection wavelength: 250 nm. **(*R*)-3r** retention time: 36 min.

Synthesis of racemic sample for HPLC analysis prepared according to General Procedure D on a 0.1 mmol scale to afford the sulfonimidamide **(*rac*)-3r** (~10 mg) with characterisation data in accordance with the above. HPLC conditions: Chiralpak IA column, 90:10 *n*hexane:*i*PrOH, flow rate: 1 mL min^{-1}, 35 °C, UV detection wavelength: 250 nm. **(*rac*)-3r** retention times: 23 & 36 min.

***tert*-Butyl (*R*)-(((3-(10,11-dihydro-5*H*-dibenzo[*b*,*f*]azepin-5-yl)propyl)(methyl)amino)(oxo)(*p*-tolyl)-λ^6-sulfaneylidene)carbamate ((*R*)-3s)**

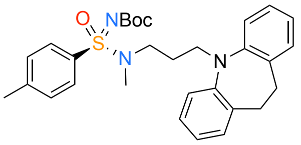

Prepared according to General Procedure D. Desipramine hydrochloride (151 mg, 0.50 mmol, 2.0 equiv) and triethylamine (140 µL, 1.00 mmol, 4.0 equiv) were added to a stirred solution of sulfonimidoyl fluoride **(*R*)-2a** (69 mg, 0.25 mmol, 1 equiv) and LiBr (43 mg, 0.50 mmol, 2 equiv) in MeCN (0.83 mL, 0.3 M) at RT and warmed to 80 °C for 24 h. The solvent was removed under reduced pressure. Purification by flash column chromatography (SiO_2, 20% Et_2O in CH_2Cl_2) afforded sulfonimidamide **(*R*)-3s** (96.8 mg, 75%, 98% *ee*) as a colourless oil. R_f 0.17 (20% EtOAc in pentane). IR (film)/cm^{-1} 3060, 2974, 2922, 1670, 1595, 1487, 1454, 1390, 1249, 1152, 910, 865, 731. ^1H NMR (400 MHz, $CDCl_3$) δ 7.67 (d, *J* = 8.3 Hz, 2H, 2 × Ar–H), 7.22 (d, *J* = 8.3 Hz, 2H, 2 × Ar–H), 7.15–7.10 (m, 4H, 4 × Ar–C), 7.08 (d, *J* = 7.4 Hz, 2H, 2 × Ar–H), 6.95–6.91 (m, 2H, 2 × Ar–H), 3.72 (t, *J* = 6.6 Hz, 2H, NCH_2), 3.29–3.17 (m, 2H, NCH_2), 3.15 (s, 4H, 2 × Ar–CH_2), 2.69 (s, 3H, NCH_3), 2.40 (s, 3H, Ar–CH_3), 1.79–1.74 (m, 2H, NCH_2CH_2), 1.42 (s, 9H, $C(CH_3)_3$). ^{13}C NMR (101 MHz, $CDCl_3$) δ 156.7 (C=O), 148.1 (2 × Ar–C_q), 143.7 (Ar–C_q), 134.7 (Ar–C_q), 134.4 (2 × Ar–C_q), 130.0 (2 × Ar–C), 129.7 (2 × Ar–C), 127.6 (2 × Ar–C), 126.5 (2 × Ar–C), 122.7 (2 × Ar–C), 119.9 (2 × Ar–C), 80.1 ($C(CH_3)_3$), 47.8 (NCH_2), 47.6 (NCH_2), 34.7 (NCH_3), 32.2 (2 × Ar–CH_2), 28.2 ($C(CH_3)_3$), 25.9 (NCH_2CH_2), 21.6 (Ar–CH_3). HRMS (ESI) m/z Calcd for $C_{30}H_{38}N_3O_3S$ $[M+H]^+$: 520.2634; Found: 520.2622. $[\alpha]^{21}_D$ = +8 (c 1.0, $CHCl_3$). HPLC conditions: Chiralpak IA column, 95:5 *n*hexane:*i*PrOH, flow rate: 1 mL min^{-1}, 35 °C, UV detection wavelength: 290 nm, **(*R*)-3s** retention time: 30 min.

Synthesis of racemic sample for HPLC analysis prepared according to General Procedure B to afford sulfonimidamide **(*rac*)-3s** (77.7 mg, 60%) as a colourless oil with characterisation data in accordance with the above. HPLC conditions: Chiralpak IA column, 95:5 *n*hexane:*i*PrOH, flow rate: 1 mL min^{-1}, 35 °C, UV detection wavelength: 290 nm, **(*rac*)-3s** retention times: 22 & 30 min.

***tert*-Butyl ((*R*)-oxo(((*S*)-1-phenylethyl)amino)(*p*-tolyl)-λ^6-sulfaneylidene)carbamate ((*R*)-3t)**

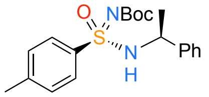

Prepared according to General Procedure D. (*S*)-1-phenylethan-1-amine (64 µL, 0.50 mmol, 2.0 equiv) and triethylamine (70 µL, 0.50 mmol, 2.0 equiv) were added to a stirred solution of sulfonimidoyl fluoride **(*R*)-2a** (69 mg, 0.25 mmol, 1 equiv) and LiBr (43 mg, 0.50 mmol, 2 equiv) in MeCN (0.83 mL, 0.3 M) at RT and warmed to 80 °C for 24 h. The solvent was removed under reduced pressure. Purification by flash column chromatography (SiO_2, 5% Et_2O in CH_2Cl_2) afforded sulfonimidamide **(*R*)-3t** (42 mg, 45%) as a single diastereomer as a colourless oil. R_f 0.24 (5% Et_2O in CH_2Cl_2). IR (film)/cm^{-1} 3083, 2978, 1673, 1453, 1367, 1278, 1162, 1118. ^1H NMR (400 MHz, $CDCl_3$) δ 7.58

(d, *J* = 8.4 Hz, 2H, 2 × Ar–H), 7.15–7.07 (m, 5H, 5 × Ar–C), 7.03–6.96 (m, 2H, 2 × Ar–H), 6.55 (d, *J* = 4.7 Hz, 1H, NH), 4.47–4.44 (m, 1H, NHC*H*), 2.34 (s, 3H, Ar–CH_3), 1.57 (d, *J* = 6.8 Hz, 3H, CHCH_3), 1.41 (s, 9H, $C(CH_3)_3$). ^{13}C NMR (101 MHz, $CDCl_3$) δ 157.3 (C=O), 143.5 (Ar–C_q), 141.3 (Ar–C_q), 136.0 (Ar–C_q), 129.3 (2 × Ar–C), 128.4 (2 × Ar–C), 127.8 (2 × Ar–C), 127.4 (Ar–C), 126.2 (2 × Ar–C), 80.4 (*C*$(CH_3)_3$), 52.6 (NCH), 28.1 (C(*C*$H_3)_3$), 24.1 (CH(*C*H_3)), 21.4 (Ar–CH_3). HRMS (APCI +p) m/z: Calcd for $C_{20}H_{27}N_2O_3S$ $[M+H]^+$: 375.1737; Found: 375.1737. $[\alpha]^{23}_D$ = +4 (c 1.0, $CDCl_3$).

***tert*-Butyl ((*R*)-oxo(((*R*)-1-phenylethyl)amino)(*p*-tolyl)-λ⁶-sulfaneylidene)carbamate ((*R*)-3u)**

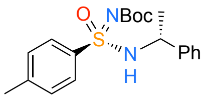

Prepared according to General Procedure D. (*R*)-1-phenylethan-1-amine (64 μL, 0.50 mmol, 2.0 equiv) and triethylamine (70 μL, 0.50 mmol, 2.0 equiv) were added to a stirred solution of sulfonimidoyl fluoride **(*R*)-2a** (69 mg, 0.25 mmol, 1 equiv) and LiBr (43 mg, 0.50 mmol, 2 equiv) in MeCN (0.83 mL, 0.3 M) at RT and warmed to 80 °C for 24 h. The solvent was removed under reduced pressure. Purification by flash column chromatography (SiO_2, 5% Et_2O in CH_2Cl_2) afforded sulfonimidamide **(*R*)-3u** (40 mg, 43%) as a single diastereomer as a colourless oil. R_f 0.28 (5% Et_2O in CH_2Cl_2). IR (film)/cm^{-1} 3063, 2978, 1677, 1453, 1367, 1274, 1159, 1118, 969, 909. ^{1}H NMR (400 MHz, $CDCl_3$) δ 7.86 (d, *J* = 8.4 Hz, 2H, 2 × Ar–H), 7.34–7.25 (m, 7H, 7 × Ar–C), 6.58 (s, 1H, NH), 4.43 (q, *J* = 6.9 Hz, 1H, NHC*H*), 2.45 (s, 3H, Ar–CH_3), 1.39 (s, 9H, $C(CH_3)_3$), 1.30 (d, *J* = 6.9 Hz, 3H, CHCH_3). ^{13}C NMR (101 MHz, $CDCl_3$) δ 157.0 (C=O), 143.9 (Ar–C_q), 142.3 (Ar–C_q), 136.8 (Ar–C_q), 129.6 (2 × Ar–C), 128.6 (2 × Ar–C), 127.9 (2 × Ar–C), 127.6 (Ar–C), 126.2 (2 × Ar–C), 80.3 (*C*$(CH_3)_3$), 52.4 (NHCH), 28.1 (C(*C*$H_3)_3$), 23.0 (CH*C*H_3), 21.5 (Ar–CH_3). HRMS (APCI +p) m/z: Calcd for $C_{20}H_{27}N_2O_3S$ $[M+H]^+$: 375.1737; Found: 375.1725. $[\alpha]^{23}_D$ = +39 (c 1.0, $CDCl_3$).

Crystal Structure Data for (*R*)-3h

(*R*)-3h

The absolute structure of **(*R*)-3h** was unambiguously determined by use of the Flack parameter [*x* = –0.035(17)].

Figures

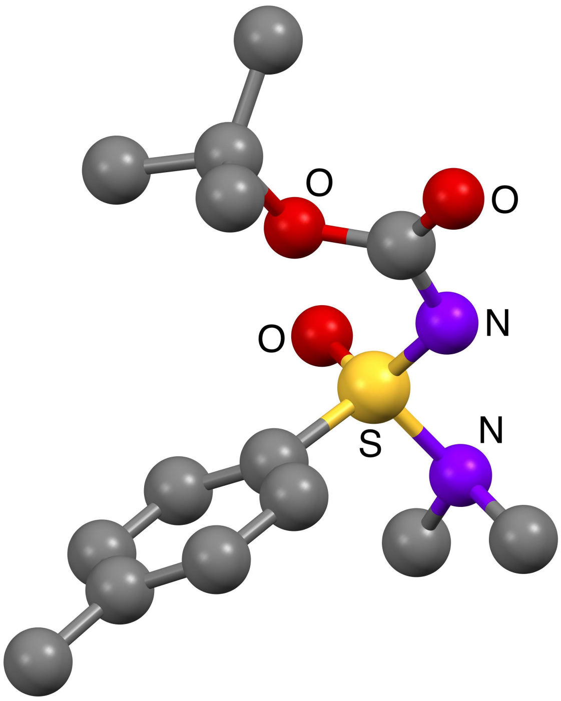

Figure S 1: The crystal structure of (R)-3h.

Preparation of enantioenriched 4-bromophenyl derivatives (Scheme 4)

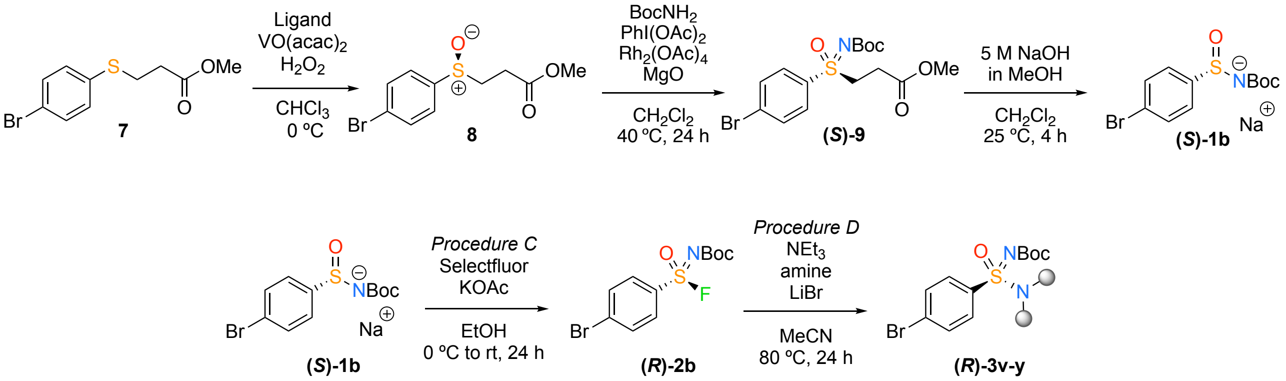

Methyl 3-((4-bromophenyl)thio)propanoate (7)

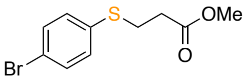

Methyl acrylate (2.00 mL, 22.0 mmol, 1.1 equiv) and sodium acetate (247 mg, 3.0 mmol, 0.15 equiv) were added to 4-bromobenzenethiol (3.78 g, 20.0 mmol, 1 equiv) in THF:H_2O (1:1, 67 mL) and stirred at 25 °C for 18 h. Aqueous $NaHCO_3$ (50 mL) was added and the aqueous mixture was extracted with EtOAc (3 × 60 mL) and washed with brine (60 mL). The combined organic layers were dried (Na_2SO_4), filtered, and concentrated under reduced pressure to give sulfide **7** (5.08, 92%) as a white solid. mp = 52–54 °C. R_f 0.18 (5% Et_2O in pentane). IR (film)/cm^{-1} 2997, 2950, 2844, 1737, 1474, 1435, 1359, 1245, 1217, 1195, 1172, 1092, 1008, 811. ^{1}H NMR (400 MHz, $CDCl_3$) δ 7.42 (d, *J* = 8.6 Hz, 2H, 2 × Ar–H), 7.23 (d, *J* = 8.6 Hz, 2H, 2 × Ar–H), 3.68 (s, 3H, OCH_3), 3.15 (t, *J* = 7.4 Hz, 2H, SCH_2), 2.62 (t, *J* = 7.4 Hz, 2H, SCH_2CH_2). ^{13}C NMR (101 MHz, $CDCl_3$) δ 172.5 (C=O), 134.6 (Ar–C_q), 132.2 (2 × Ar–C), 131.8 (2 × Ar–C), 120.8 (Ar–C_q), 52.0 (OCH_3), 34.2 (SCH_2), 29.3 (SCH_2CH_2). Analytical data (NMR) in agreement with those reported in the literature.[3]

Methyl 3-((4-bromophenyl)sulfinyl)propanoate (8)

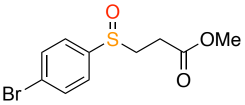

Prepared according to a literature procedure.[4,5] A solution of VO(acac)$_2$ (16 mg, 0.06 mmol, 1 mol%) in $CHCl_3$ (1.5 mL) was added dropwise to a solution of (*S*,*E*)-2-(((1-hydroxy-3,3-dimethylbutan-2-yl)imino)methyl)-4,6-diiodophenol (42 mg, 0.09 mmol, 1.5 mol%) in $CHCl_3$ (1.5 mL) and stirred at 25 °C for 30 min. Sulfide **7** (1.65 g, 6.0 mmol, 1.0 equiv) and $CHCl_3$ (3 mL) were added and the reaction mixture cooled to 0 °C. H_2O_2 (30% in H_2O, 736 µL, 7.2 mmol, 1.2 equiv) was added dropwise and the mixture left to vigorously stir at 0 °C for 72 h. Sat. aq. $Na_2S_2O_3$ (30 mL) was added and the aqueous mixture was extracted with $CHCl_3$ (3 × 30 mL) and washed with brine (40 mL). The combined organic layers were dried (Na_2SO_4), filtered, and concentrated under reduced pressure. Purification by flash column chromatography (40% EtOAc in pentane) afforded sulfoxide **(*S*)-8** (1.19 g, 68%, 99% *ee*) as a white solid. mp = 79–80 °C. R_f 0.23 (40% EtOAc in pentane). IR (film)/cm^{-1} 3050, 2997, 2948, 2916, 2846, 1728, 1571, 1472, 1435, 1415, 1388, 1239, 1170, 1131, 1060, 1034, 1005, 893, 826, 762, 732, 718. ^{1}H NMR (400 MHz, $CDCl_3$) δ 7.68 (d, *J* = 8.5 Hz, 2H, 2 × Ar–H), 7.49 (d, *J* = 8.6 Hz, 2H, 2 × Ar–H), 3.67 (s, 3H, OCH_3), 3.23 (ddd, *J* = 13.2, 8.2, 6.8 Hz, 1H, SC*H*H), 2.94 (ddd, *J* = 13.4, 8.0, 5.7 Hz, 1H, SCH*H*), 2.84 (ddd, *J* = 17.2, 8.1, 6.8 Hz, 1H, SCH_2C*H*H), 2.56 (ddd, *J* = 17.2, 8.2, 5.7 Hz, 1H, SCH_2CH*H*). ^{13}C NMR (101 MHz, $CDCl_3$) δ 171.6 (C=O), 142.2 (Ar–C_q), 132.7 (2 × Ar–C), 125.8 (Ar–C_q), 125.8 (2 × Ar–C), 52.3 (SCH_2), 51.3 (OCH_3), 26.1 (SCH_2CH_2). HRMS (Voltage EI+) *m/z* Calcd for $C_{10}H_{11}O_3SBr$ $[M+H]^+$: 289.9612;

Found: 289.9607. $[\alpha]^{23}_{D}$ = –98 (c 1.0, $CHCl_3$). HPLC conditions: Chiralpak IA column, 90:10 *n*hexane:*i*PrOH, flow rate: 1 mL min^{-1}, 35 °C, UV detection wavelength: 250 nm, **(*S*)-8** retention time: 19 min.

Synthesis of racemic sample for HPLC analysis prepared by *m*CPBA oxidation to afford sulfoxide **(*rac*)-8** (5.09 g, 87%) as a white solid with characterisation data in accordance with the above. HPLC conditions: Chiralpak IA column, 90:10 *n*hexane:*i*PrOH, flow rate: 1 mL min^{-1}, 35 °C, UV detection wavelength: 250 nm, **(*rac*)-8** retention times: 19 & 21 min.

Methyl 3-(4-bromo-*N*-(*tert*-butoxycarbonyl)phenylsulfonimidoyl)propanoate ((*S*)-9)

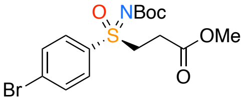

Prepared according to a literature procedure.[6] Magnesium oxide (659 mg, 16.4 mmol, 4 equiv), *tert*-butyl carbamate (720 mg, 6.2 mmol, 1.5 equiv), $PhI(OAc)_2$ (1.98 g, 6.2 mmol, 1.5 equiv) and $Rh_2(OAc)_4$ (45 mg, 0.10 mmol, 2.5 mol%) were added to a stirred solution of sulfoxide **(*S*)-8** (1.19 g, 4.1 mmol, 1 equiv) in CH_2Cl_2 (40 mL, 0.1 M) at RT and warmed to 40 °C for 18 h. At rt, the reaction mixture was filtered through celite and concentrated under reduced pressure. Purification by flash column chromatography (25% EtOAc in pentane) afforded sulfoximine **(*S*)-9** (1.45 g, 87%, 99% *ee*) as a white solid. mp = 83–84 °C. R_f 0.15 (25% EtOAc in pentane). mp = 100–101 °C. IR (film)/cm^{-1} 2978, 1740 (C=O), 1699, 1669, 1390, 1274, 1252, 1155, 1110. ^1H NMR (400 MHz, $CDCl_3$) δ 7.82–7.78 (m, 2H, 2 × Ar–H), 7.77–7.73 (m, 2H, 2 × Ar–H), 3.76–3.65 (m, 1H, SC*H*H), 3.63 (s, 3H, OCH_3), 3.58 (dd, *J* = 6.3, 5.5 Hz, 1H, SCH*H*), 2.81 (qdd, *J* = 17.3, 8.9, 6.2 Hz, 2H, SCH_2CH_2), 1.38 (s, 9H, $C(CH_3)_3$). ^{13}C NMR (101 MHz, $CDCl_3$) δ 170.0 (C=O), 157.3 (C=O), 136.0 (Ar–C_q), 133.0 (Ar–C_q), 129.6 (2 × Ar–C), 129.4 (2 × Ar–C), 81.0 ($C(CH_3)_3$), 52.4 (OCH_3), 51.6 (SCH_2), 27.9 ($C(CH_3)_3$), 27.1 (SCH_2CH_2). HRMS (ESI) m/z: Calcd for $C_{15}H_{21}NO_5S_{81}Br$ $[M+H]^+$: 408.0303; Found: 408.0296. $[\alpha]^{23}_{D}$ = +44 (c 1.0, $CHCl_3$). HPLC conditions: Chiralpak IA column, 93:7 *n*hexane:*i*PrOH, flow rate: 1 mL min^{-1}, 35 °C, UV detection wavelength: 250 nm, **(*R*)-9** retention time: 22 min.

Synthesis of racemic sample for HPLC analysis prepared by using the above procedure to afford sulfoximine **(*rac*)-9** (6.24 g, 95%) as a white solid with characterisation data in accordance with the above. HPLC conditions: Chiralpak IA column, 93:7 *n*hexane:*i*PrOH, flow rate: 1 mL min^{-1}, 35 °C, UV detection wavelength: 250 nm, **(*rac*)-9** retention times: 22 & 37 min.

Sodium ((4-bromophenyl)sulfinyl)(*tert*-butoxycarbonyl)amide ((*S*)-1b)

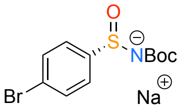

5 M NaOH in MeOH (718 µL, 3.6 mmol, 1.05 equiv) was added to sulfoximine **(*S*)-9** (1.39 g, 3.4 mmol, 1.0 equiv) at 25 °C in CH_2Cl_2 (25 mL, 0.1 M) and the reaction was stirred for 1 h. The suspension was then filtered to collect the white precipitate, which was washed with CH_2Cl_2 (100 mL). Excess solvent was then removed under reduced pressure to afford sulfinamide salt **(*S*)-1b** as a white solid (1.05 g, 90%, 99% *ee*). mp = 227–229 °C. IR (film)/cm^{-1} 2981, 1640, 1468, 1390, 1271, 1162, 998, 834, 760; ^1H NMR (400 MHz, D_2O) δ 7.71–7.65 (m, 2H, 2 × Ar–H), 7.56–7.50 (m, 2H, 2 × Ar–H), 1.40 (s, 9H, $C(CH_3)_3$). ^{13}C NMR (101 MHz, D_2O) δ 165.9 (C=O), 145.6 (Ar–C_q), 132.0 (2 × Ar–C), 126.5 (2 × Ar–C), 124.6 (Ar–C_q), 79.7 ($C(CH_3)_3$), 27.7 ($C(CH_3)_3$). HRMS (ESI) m/z: Calcd for $C_{11}H_{13}NO_3SBr$ $[M]^-$: 317.9800; Found: 317.9806. Further characterisation was carried out after washing ~20 mg with sat. aq. NH_4Cl solution. Sulfinamide: $[\alpha]^{23}_{D}$ = +88 (c 1.0, $CDCl_3$). HPLC conditions: Chiralpak IA column, 95:5 *n*hexane:*i*PrOH, flow rate: 1 mL min^{-1}, 35 °C, UV detection wavelength: 250 nm, **(*S*)-1b** retention time: 20 min.

Synthesis of racemic sample for HPLC analysis prepared by using the above procedure to afford sulfinamide salt ***(rac)*-1b** (786 mg, 89%) as a white solid with characterisation data in accordance with the above. HPLC conditions: Chiralpak IA column, 95:5 *n*hexane:*i*PrOH, flow rate: 1 mL min^{-1}, 35 °C, UV detection wavelength: 250 nm, ***(rac)*-1b** retention times: 18 & 20 min.

***tert*-Butyl ((4-bromophenyl)fluoro(oxo)-λ^6-sulfaneylidene)carbamate ((*R*)-2b)**

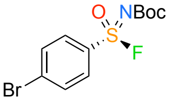

Prepared according to General Procedure C. Selectfluor (850 mg, 2.4 mmol, 2.0 equiv) was added to a stirred solution of sulfinamide salt **(*S*)-1b** (411 mg, 1.2 mmol, 1.0 equiv) and potassium acetate (235 mg, 2.4 mmol, 2.0 equiv) in ethanol (6.0 mL, 0.2 M) at 0 ºC and slowly warmed to RT over 24 h. The reaction mixture was quenched with water (10 mL) and diluted with CH_2Cl_2 (10 mL). The mixture was extracted with CH_2Cl_2 (5 × 15 mL) and the combined organic layers were dried (Na_2SO_4), filtered and concentrated under reduced pressure. No further purification was required giving sulfonimidoyl fluoride **(*R*)-2b** (175 mg, quant, 92% *ee*) as a colourless oil. IR (film)/cm^{-1} 3093, 2982, 1707, 1573, 1331, 1252, 1148, 1069, 1010, 909, 857, 756. ^{1}H NMR (400 MHz, $CDCl_3$) δ 8.01–7.94 (m, 2H, 2 × Ar–H), 7.79–7.74 (m, 2H, 2 × Ar–H), 1.53 (s, 9H, $C(CH_3)_3$). ^{13}C NMR (101 MHz, $CDCl_3$) δ 151.7 (C=O), 132.5 (2 × Ar–C), 132.3 (Ar–C_q), 130.8 (Ar–C_q), 129.2 (2 × Ar–C), 82.7 (*C*(CH_3)$_3$), 27.5 (C(CH_3)$_3$). ^{19}F NMR (377 MHz, $CDCl_3$) δ 69.8. $[\alpha]^{23}_D$ = –15 (c 1.7, $CDCl_3$). HPLC conditions: Chiralpak IA column, 98:2 *n*hexane:*i*PrOH, flow rate: 1 mL min^{-1}, 35 °C, UV detection wavelength: 250 nm, **(*R*)-2b** retention time: 11 min.

Synthesis of racemic sample for HPLC analysis prepared by using the above procedure to afford sulfonimidoyl fluoride ***(rac)*-2b** (175 mg, quant.) as a colourless oil with characterisation data in accordance with the above. HPLC conditions: Chiralpak IA column, 98:2 *n*hexane:*i*PrOH, flow rate: 1 mL min^{-1}, 35 °C, UV detection wavelength: 250 nm, ***(rac)*-2b** retention times: 10 & 11 min.

***tert*-Butyl (*R*)-((benzylamino)(4-bromophenyl)(oxo)-λ^6-sulfaneylidene)carbamate ((*R*)-3v)**

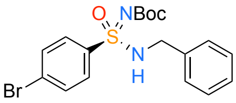

Prepared according to General Procedure D. Benzylamine (55 µL, 0.50 mmol, 2.0 equiv) and triethylamine (70 µL, 0.50 mmol, 2.0 equiv) were added to a stirred solution of sulfonimidoyl fluoride **(*R*)-2b** (85 mg, 0.25 mmol, 1.0 equiv) and LiBr (43 mg, 0.50 mmol, 2.0 equiv) in MeCN (0.83 mL, 0.3 M) at RT and heated to 80 °C for 24 h. The solvent was removed under reduced pressure. Purification by flash column chromatography (SiO_2, 2% Et_2O in CH_2Cl_2) afforded sulfonimidamide **(*R*)-3v** (66 mg, 62%, 92% *ee*) as a white solid. R_f = 0.23 (2% Et_2O in CH_2Cl_2); mp = 154–156 °C. IR (film)/cm^{-1} 3086, 1684, 1572, 1282, 1159, 1088, 909; ^{1}H NMR (400 MHz, $CDCl_3$) δ 7.79–7.75 (m, 2H, 2 × Ar–H), 7.63–7.60 (m, 2H, 2 × Ar–H), 7.30–7.25 (m, 3H, 3 × Ar–C), 7.23–7.17 (m, 2H, 2 × Ar–H), 6.89 (s, 1H, NH), 4.25 (d, *J* = 14.1 Hz, 1H, NHC*H*H), 3.99 (d, *J* = 14.1 Hz, 1H, NHCH*H*), 1.38 (s, 9H, $C(CH_3)_3$). ^{13}C NMR (101 MHz, $CDCl_3$) δ 156.8 (C=O), 138.0 (Ar-C_q), 135.6 (Ar-C_q), 132.3 (2 × Ar–C), 129.4 (2 × Ar–C), 128.7 (2 × Ar–C), 128.2 (Ar–C_q), 128.0 (3 × Ar–C), 80.9 (*C*(CH_3)$_3$), 45.5 (NH*C*H_2), 28.0 (C(CH_3)$_3$). HRMS (ESI) m/z: Calcd for $C_{18}H_{22}N_2O_3SBr$ $[M+H]^+$: 425.0535; Found: 425.0531. $[\alpha]^{23}_D$ = +6 (c 0.5, CH_2Cl_2). HPLC conditions: Chiralpak ID column, 90:10 *n*hexane:*i*PrOH, flow rate: 1 mL min^{-1}, 35 °C, UV detection wavelength: 254 nm, **(*R*)-3v** retention time: 15 min.

Synthesis of racemic sample for HPLC analysis prepared by using the above procedure to afford sulfonimidamide **(*rac*)-3v** (20 mg, 47%) as a white solid with characterisation data in accordance with the above. HPLC conditions: Chiralpak ID column, 90:10 *n*hexane:*i*PrOH, flow rate: 1 mL min^{-1}, 35 °C, UV detection wavelength: 254 nm, **(*rac*)-3v** retention times: 15 & 20 min.

***tert*-Butyl (*R*)-((allylamino)(4-bromophenyl)(oxo)-λ^6-sulfaneylidene)carbamate ((*R*)-3w)**

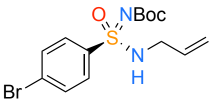

Prepared according to General Procedure D. Allylamine (38 µL, 0.50 mmol, 2.0 equiv) and triethylamine (70 µL, 0.50 mmol, 2.0 equiv) were added to a stirred solution of sulfonimidoyl fluoride **(*R*)-2b** (85 mg, 0.25 mmol, 1.0 equiv) and LiBr (43 mg, 0.50 mmol, 2.0 equiv) in MeCN (0.83 mL, 0.3 M) at RT and heated to 80 °C for 24 h. The solvent was removed under reduced pressure. Purification by flash column chromatography (SiO_2, 5% Et_2O in CH_2Cl_2) afforded sulfonimidamide **(*R*)-3w** (70 mg, 75%, 90% *ee*) as a white solid. R_f = 0.31 (5% Et_2O in CH_2Cl_2); mp = 86–88 °C. IR (film)/cm^{-1} 3243, 2981, 1677, 1572, 1390, 1282, 1159, 1088, 905; ^{1}H NMR (400 MHz, $CDCl_3$) δ 7.84–7.81 (m, 2H, 2 × Ar–H), 7.70–7.66 (m, 2H, 2 × Ar–H), 6.36 (s, 1H, NH), 5.73 (dddd, *J* = 17.1, 10.2, 6.1, 5.5 Hz, 1H, NCH_2CH), 5.22 (dq, *J* = 17.1, 1.5 Hz, 1H, NCH_2CHCHH), 5.15 (dq, *J* = 10.2, 1.3 Hz, 1H, NCH_2CHCHH), 3.68 (dd, *J* = 15.1, 5.5 Hz, 1H, NC*H*H), 3.46 (dd, *J* = 15.0, 6.1 Hz, 1H, NCH*H*), 1.41 (s, 9H, $C(CH_3)_3$). ^{13}C NMR (101 MHz, $CDCl_3$) δ 156.7 (C=O), 138.0 (Ar–C_q), 132.4 (2 × Ar–C), 132.3 (NCH_2CH), 129.5 (2 × Ar–C), 128.2 (Ar–C_q), 118.2 (NCH_2CHCH_2), 80.8 ($C(CH_3)_3$), 43.9 (NCH_2), 28.0 ($C(CH_3)_3$). HRMS (ESI) m/z: Calcd for $C_{14}H_{20}N_2O_3SBr$ $[M+H]^+$: 375.0378; Found: 375.0380. $[\alpha]^{23}_D$ = +8 (c 0.5, CH_2Cl_2). HPLC conditions: Chiralpak IA column, 97:3 *n*hexane:*i*PrOH, flow rate: 1 mL min^{-1}, 35 °C, UV detection wavelength: 250 nm, **(*R*)-3w** retention time: 20 min.

Synthesis of racemic sample for HPLC analysis prepared by using the above procedure to afford sulfonimidamide **(*rac*)-3w** (18 mg, 48%) as a white solid with characterisation data in accordance with the above. HPLC conditions: Chiralpak IA column, 97:3 *n*hexane:*i*PrOH, flow rate: 1 mL min^{-1}, 35 °C, UV detection wavelength: 250 nm, **(*rac*)-3w** retention times: 20 & 29 min.

***tert*-Butyl ((4-bromophenyl)(oxo)(piperidin-1-yl)-λ^6-sulfaneylidene)carbamate ((*R*)-3x)**

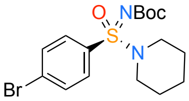

Prepared according to General Procedure D. Piperidine (50 µL, 0.50 mmol, 2.0 equiv) and triethylamine (70 µL, 0.50 mmol, 2.0 equiv) were added to a stirred solution of sulfonimidoyl fluoride **(*R*)-2b** (85 mg, 0.25 mmol, 1.0 equiv) and LiBr (43 mg, 0.50 mmol, 2.0 equiv) in MeCN (0.83 mL, 0.3 M) at RT and heated to 80 °C for 24 h. The solvent was removed under reduced pressure. Purification by flash column chromatography (SiO_2, 2% Et_2O in CH_2Cl_2) afforded sulfonimidamide **(*R*)-3x** (88 mg 87%, 90% *ee*) as a white solid. R_f = 0.36 (2% Et_2O in CH_2Cl_2); mp = 171–172 °C. IR (film)/cm^{-1} 2974, 2937, 2855, 1674, 1572, 1275, 1151, 931, 745; ^{1}H NMR (400 MHz, $CDCl_3$) δ 7.75–7.71 (m, 2H, 2 × Ar–H), 7.69–7.65 (m, 2H, 2 × Ar–H), 3.20–3.05 (m, 4H, 2 × NCH_2), 1.67–1.59 (m, 4H, 2 × NCH_2CH_2), 1.51–1.44 (m, 2H, $NCH_2CH_2CH_2$), 1.42 (s, 9H, $C(CH_3)_3$). ^{13}C NMR (101 MHz, $CDCl_3$) δ 156.4 (C=O), 135.8 (Ar–C_q), 132.3 (2 × Ar–C), 129.2 (2 × Ar–C), 127.9 (Ar–C_q), 80.4 ($C(CH_3)_3$), 46.6 (2 × NCH_2), 28.0 ($C(CH_3)_3$), 25.2 (2 × NCH_2CH_2), 23.5 ($NCH_2CH_2CH_2$). HRMS (ESI) m/z: Calcd for $C_{16}H_{24}N_2O_3SBr$ $[M+H]^+$: 403.0691; Found: 403.0703. $[\alpha]^{23}_D$ = –8 (c 0.5, CH_2Cl_2). HPLC conditions: Chiralpak IA column, 95:5 *n*hexane:*i*PrOH, flow rate: 1 mL min^{-1}, 35 °C, UV detection wavelength: 250 nm, **(*R*)-3x** retention time: 24 min.

Synthesis of racemic sample for HPLC analysis prepared by using the above procedure to afford sulfonimidamide **(*rac*)-3x** (~20mg, 50%) as a white solid with characterisation data in accordance with the above. HPLC conditions: Chiralpak IA column, 95:5 *n*hexane:*i*PrOH, flow rate: 1 mL min^{-1}, 35 °C, UV detection wavelength: 250 nm, **(*rac*)-3x** retention times: 16 & 24 min.

***tert*-Butyl ((*R*)-(4-bromophenyl)(methyl((*S*)-3-(naphthalen-1-yloxy)-3-(thiophen-2-yl)propyl)amino)(oxo)-λ⁶-sulfaneylidene)carbamate ((*R*)-3y)**

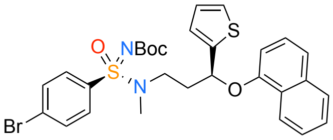

Prepared according to General Procedure D. Duloxetine Hydrochloride (167 mg, 0.50 mmol, 2.0 equiv) and triethylamine (140 µL, 1.0 mmol, 4.0 equiv) were added to a stirred solution of sulfonimidoyl fluoride **(*R*)-2a** (85 mg, 0.25 mmol, 1.0 equiv) and LiBr (43 mg, 0.50 mmol, 2.0 equiv) in MeCN (0.83 mL, 0.3 M) at RT and heated to 80 °C for 24 h. The solvent was removed under reduced pressure. Purification by flash column chromatography (SiO_2, 5% Et_2O in CH_2Cl_2) followed by (SiO_2, 10% acetone in pentane) afforded sulfonimidamide **(*R*)-3y** (124 mg, 80%) as a single diastereomer as a white solid. R_f 0.15 (10% acetone in pentane). mp = 57–60 °C. IR (film)/cm^{-1} 2978, 1744, 1673, 1572, 1461, 1394, 1263, 1151, 1088, 775. ^{1}H NMR (400 MHz, $CDCl_3$) δ 8.27 (dd, *J* = 6.9, 3.0 Hz, 1H, Ar–H), 7.82–7.76 (m, 1H, Ar–H), 7.70 (d, *J* = 8.6 Hz, 2H, 2 × Ar–H), 7.56–7.46 (m, 4H, 4 × Ar–C), 7.41 (d, *J* = 8.2 Hz, 1H, Ar–H), 7.32–7.24 (m, 1H, Ar–H), 7.22 (dd, *J* = 5.1, 1.2 Hz, 1H, Ar–H), 7.10 (d, *J* = 3.4 Hz, 1H, Ar–H), 6.94 (dd, *J* = 5.0, 3.5 Hz, 1H, Ar–H), 6.83 (d, *J* = 7.7 Hz, 1H, Ar–H), 5.74 (dd, *J* = 8.3, 4.3 Hz, 1H, OC*H*Ar), 3.52 (ddd, *J* = 13.7, 8.2, 5.3 Hz, 1H, NC*H*H), 3.37 (dt, *J* = 14.2, 7.4 Hz, 1H, NCH*H*), 2.87 (s, 3H, NCH_3), 2.51 (dtd, *J* = 13.5, 8.0, 5.2 Hz, 1H, NCH_2C*H*H), 2.40–2.28 (m, 1H, NCH_2CH*H*), 1.45 (s, 9H, $C(CH_3)_3$). ^{13}C NMR (101 MHz, $CDCl_3$) δ 156.3 (C=O), 152.8 (Ar–C_q), 144.3 (Ar–C_q), 136.6 (Ar–C_q), 134.5 (Ar–C_q), 132.3 (2 × Ar–C), 128.9 (2 × Ar–C), 128.0 (Ar–C_q), 127.6 (Ar–C), 126.7 (Ar–C), 126.3 (Ar–C), 125.9 (Ar–C_q), 125.7 (Ar–C), 125.3 (Ar–C), 124.98 (Ar–C), 124.97 (Ar–C), 121.8 (Ar–C), 120.9 (Ar–C), 107.0 (Ar–C), 80.5 (*C*$(CH_3)_3$), 73.4 (O*C*HAr), 47.0 (NCH_2), 37.4 (NCH_3), 35.4 (NCH_2*C*H_2), 28.1 (C(*C*H_3)$_3$). HRMS (ESI) m/z: Calcd for $C_{29}H_{31}N_2O_4S_2BrNa$ $[M+Na]^+$: 637.0815; Found: 637.0806. $[\alpha]^{23}_D$ = +10 (c 0.5, CH_2Cl_2).

***tert*-Butyl (*R*)-([1,1'-biphenyl]-4-yl(oxo)(piperidin-1-yl)-λ⁶-sulfaneylidene)carbamate ((*R*)-10)**

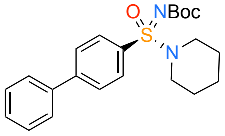

Prepared according to a literature procedure.[7] Sulfonimidamide **(*R*)-3x** (40 mg, 0.10 mmol, 1 equiv), phenylboronic acid (18 mg, 0.15 mmol, 1.5 equiv), potassium carbonate (28 mg, 0.20 mmol, 2 equiv) and 1,1'-bis(di-tert-butylphosphino)ferrocene palladium dichloride (6.5 mg, 0.01 mmol, 10 mol%) was added to a glass vial with MeCN:H_2O (1:1, 500 µL, 0.2 M) and was degassed before being stirred and heated to 80 °C for 2 h. The reaction mixture was quenched with aq. sat. NH_4Cl (10 mL) and diluted with CH_2Cl_2 (10 mL). The mixture was extracted with CH_2Cl_2 (3 × 10 mL) and the combined organic layers were dried (Na_2SO_4), filtered and concentrated under reduced pressure. Purification by flash column chromatography (SiO_2, 3% Et_2O in CH_2Cl_2) afforded sulfonimidamide **(*R*)-10** (37 mg, 91%, 90% *ee*) as a colourless oil. R_f 0.38 (3% Et_2O in CH_2Cl_2). IR (film)/cm^{-1} 2974, 2937, 2855, 1669, 1595, 1453, 1394, 1248, 1148, 928, 760. ^{1}H NMR (400 MHz, $CDCl_3$) δ 7.95–7.90 (m, 2H, 2 × Ar–H), 7.76–7.70 (m, 2H, 2 × Ar–H), 7.64–7.60 (m, 2H, 2 × Ar–H), 7.52–7.46 (m, 2H, 2 × Ar–H), 7.45–7.39 (m, 1H, Ar–H), 3.17 (q, *J* = 4.9 Hz, 4H, 2 × NCH_2), 1.67 (p, *J* = 5.6 Hz, 4H, 2 × NCH_2C*H*$_2$), 1.52–1.46 (m, 2H, NCH_2CH_2C*H*$_2$), 1.43 (s, 9H, $C(CH_3)_3$). ^{13}C NMR (101 MHz, $CDCl_3$) δ 156.7 (C=O), 145.7 (Ar–C_q), 139.2 (Ar–

C_q), 135.1 (Ar–C_q), 129.0 (2 × Ar–C), 128.5 (Ar–C), 128.2 (2 × Ar–C), 127.6 (2 × Ar–C), 127.3 (2 × Ar–C), 80.1 ($C(CH_3)_3$), 46.6 (2 × NCH_2), 28.1 ($C(CH_3)_3$), 25.2 (2 × NCH_2CH_2), 23.6 ($NCH_2CH_2CH_2$). $[\alpha]^{23}_{D}$ = –7 (c 1.0, $CDCl_3$). HPLC conditions: Chiralpak IA column, 95:5 *n*hexane:*i*PrOH, flow rate: 1 mL min^{-1}, 35 °C, UV detection wavelength: 250 nm, **(*R*)-10** retention time: 32 min.

Synthesis of racemic sample for HPLC analysis prepared by using the above procedure to afford sulfonimidamide **(*rac*)-10** (~10 mg) as a colourless oil with characterisation data in accordance with the above. HPLC conditions: Chiralpak IA column, 95:5 *n*hexane:*i*PrOH, flow rate: 1 mL min^{-1}, 35 °C, UV detection wavelength: 250 nm, **(*rac*)-10** retention times: 27 & 32 min.

Experimental Data for Racemic Sulfinamide Salts (S4b-f)

Synthesis of sulfinamide salts

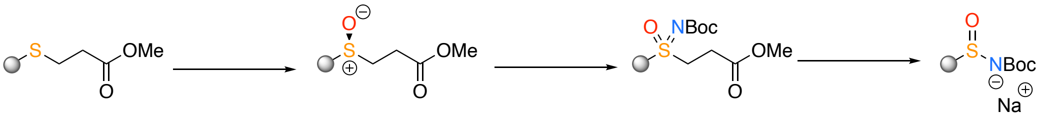

<u>Thiol Alkylation</u>

Methyl 3-(tolylthio)propanoate (S1a)

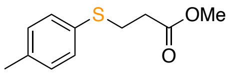

Methyl acrylate (2.00 mL, 22.1 mmol) and sodium acetate (247 mg, 3.02 mmol) were added to 4-methylbenzene-1-thiol (2.50 g, 20.1 mmol) in THF:H_2O (1:1, 67 mL) and stirred at 25 °C for 18 h. Aqueous $NaHCO_3$ (50 mL) was added and the aqueous mixture was extracted with EtOAc (3 × 60 mL) and washed with brine (60 mL). The combined organic layers were dried (Na_2SO_4), filtered, and concentrated under reduced pressure to give sulfide **S1a** (4.25, quant.) as a colourless oil. IR (film)/cm^{-1} 3019, 2952, 1737 (C=O), 1491, 1435, 1353, 1193, 1241, 1092, 1017, 980, 805. ^{1}H NMR (400 MHz, $CDCl_3$) δ 7.29 (d, J = 8.1 Hz, 2H, 2 × Ar–H), 7.11 (d, J = 7.9 Hz, 2H, 2 × Ar–H), 3.67 (s, 3H, OCH_3), 3.11 (t, J = 7.4 Hz, 2H, SCH_2), 2.60 (t, J = 7.4 Hz, 2H, SCH_2CH_2), 2.32 (s, 3H, Ar–CH_3). ^{13}C NMR (101 MHz, $CDCl_3$) δ 172.3 (C=O), 136.9 (Ar–C_q), 131.3 (Ar–C_q), 131.1 (2 × Ar–C), 129.8 (2 × Ar–C), 51.8 (OCH_3), 34.3 (SCH_2), 29.8 (SCH_2CH_2), 21.1 (Ar–CH_3). Analytical data (NMR) in agreement with those reported in the literature.[8]

Methyl 3-(phenylthio)propanoate (S1b)

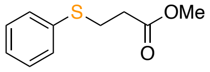

Methyl acrylate (269 µL, 3.0 mmol, 1.0 equiv) and sodium acetate (37 mg, 0.45 mmol, 0.15 equiv) were added to benzenethiol (306 µL, 3.0 mmol, 1.0 equiv) in THF:H_2O (1:1, 10 mL) and stirred at 25 °C for 18 h. Aqueous $NaHCO_3$ (50 mL) was added and the aqueous mixture was extracted with EtOAc (3 × 15 mL). The combined organic layers were dried (Na_2SO_4), filtered, and concentrated under reduced pressure. Purification by flash column chromatography (5% EtOAc in pentane) afforded sulfide **S1b** (498 mg, 72%) as a colourless oil. R_f 0.25 (5% EtOAc in pentane). IR (film)/cm^{-1} 2951, 1733 (C=O), 1583, 1481, 1437, 1356, 1243, 1171, 1024, 737, 690. ^{1}H NMR (400 MHz, $CDCl_3$) δ 7.39–7.35 (m, 2H, 2 × Ar–H), 7.35–7.25 (m, 2H, 2 × Ar–H), 7.24–7.18 (m, 1H, Ar–H), 3.68 (s, 3H, OCH_3), 3.17 (t, *J* = 7.4 Hz, 2H, SCH_2), 2.63 (t, *J* = 7.4 Hz, 2H, SCH_2CH_2). ^{13}C NMR (101 MHz, $CDCl_3$) δ 172.2 (C=O), 135.1 (Ar–C_q), 130.1 (Ar–C), 129.0 (2 × Ar–C), 126.6 (2 × Ar–C), 51.9 (OCH_3), 34.2 (SCH_2), 29.1 (SCH_2CH_2). Analytical data (NMR) in agreement with those reported in the literature.[9]

Methyl 3-((4-fluorophenyl)thio)propanoate (S1c)

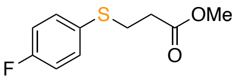

Methyl acrylate (1.81 mL, 20 mmol, 1.0 equiv) and sodium acetate (247 mg, 3.00 mmol, 0.15 equiv) were added to the 4-fluorobenzenethiol (2.13 mL, 20 mmol, 1 equiv) in THF:H_2O (67 mL, 1:1) and stirred at 25 °C and monitored by TLC. Aqueous $NaHCO_3$ (50 mL) was added and the aqueous mixture was extracted with EtOAc (3 × 60 mL). The combined organic layers were dried ($MgSO_4$), filtered, and concentrated under reduced pressure to give sulfide **S1c** (3.59 g, 84%) as a yellow oil which was

used without further purification. IR (film)/cm^{-1} 2952, 1733 (C=O), 1588, 1491, 1435, 1357, 1219, 1156, 1088, 980, 820. ^{1}H NMR (400 MHz, $CDCl_3$) δ 7.39–7.36 (m, 2H, 2 × Ar–H), 7.02–6.98 (m, 2H, 2 × Ar–H), 3.66 (s, 3H, OCH_3), 3.09 (t, *J* = 7.4 Hz, 2H, SCH_2), 2.58 (t, *J* = 7.4 Hz, 2H, SCH_2CH_2).^{13}C NMR (101 MHz, $CDCl_3$) δ 172.2 (C=O), 162.3 (d, $^{1}J_{C\text{-}F}$ = 247 Hz, Ar–C_q), 133.5 (d, $^{3}J_{C\text{-}F}$ = 8.1 Hz, 2 × Ar–C), 130.1 (d, $^{4}J_{C\text{-}F}$ = 3.1 Hz, Ar–C_q), 116.3 (d, $^{2}J_{C\text{-}F}$ = 21.9 Hz, 2 × Ar–C), 51.9 (OCH_3), 34.3 (SCH_2), 30.5 (SCH_2CH_2). ^{19}F NMR (377 MHz, $CDCl_3$) δ -114.6– -114.7 (m, 1F, Ar–F). Analytical data (NMR) in agreement with those reported in the literature.[10]

Methyl 3-((4-methoxyphenyl)thio)propanoate (S1d)

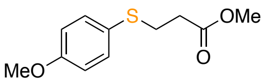

Methyl acrylate (1.81 mL, 20 mmol, 1.0 equiv) and sodium acetate (247 mg, 3.00 mmol, 0.15 equiv) were added to the 4-methoxybenzenethiol (2.13 mL, 20 mmol, 1 equiv) in THF:H_2O (67 mL, 1:1) and stirred at 25 °C and monitored by TLC. Aqueous $NaHCO_3$ (50 mL) was added and the aqueous mixture was extracted with EtOAc (3 × 60 mL). The combined organic layers were dried ($MgSO_4$), filtered, and concentrated under reduced pressure to give the corresponding sulfide **S1d** (3.76 g, 83%) as an orange oil which was used without further purification. IR (film)/cm^{-1} 3001, 2952, 2837, 1733 (C=O), 1592, 1491, 1461, 1357, 1241, 1170, 1029, 980, 824. ^{1}H NMR (400 MHz, $CDCl_3$) δ 7.40–7.33 (m, 2H, 2 × Ar–H), 6.86–6.81 (m, 2H, 2 × Ar–H), 3.78 (s, 3H, OCH_3), 3.65 (s, 3H, OCH_3), 3.03 (t, *J* = 7.4 Hz, 2H, SCH_2), 2.56 (t, *J* = 7.4 Hz, 2H, SCH_2CH_2). ^{13}C NMR (101 MHz, $CDCl_3$) δ 172.3 (C=O), 159.4 (Ar–C_q), 134.2 (2 × Ar–C), 125.1 (2 × Ar–C), 114.7 (Ar–C_q), 55.3 (Ar–OCH_3), 51.7 ($COOCH_3$), 34.4 (SCH_2), 31.1 (SCH_2CH_2). Analytical data (NMR) in agreement with those reported in the literature.[11]

Methyl 3-(pyridin-2-ylthio)propanoate (S1e)

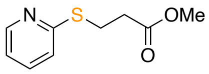

Prepared according to a literature procedure.[12] Methyl 3-bromopropionate (2.6 mL, 24 mmol, 1.2 equiv) was added to a stirred solution of 2-mercaptopyridine (2.22 g, 20 mmol, 1 equiv) and NEt_3 (4.16 mL, 30 mmol, 1.5 equiv) in acetonitrile (10 mL, 2 M) and heated under reflux to 85 ºC for 24 h. At rt the reaction mixture was concentrated under reduced pressure. Purification by flash column chromatography (10% EtOAc in pentane) afforded sulfide **S1e** (3.11 g, 15.8 mmol, 79%) as a pale-yellow oil. R_f 0.33 (10% EtOAc in pentane). IR (film)/cm^{-1} 3049, 2997, 2952, 1737 (C=O), 1580, 1454, 1412, 1357, 1249, 1108, 984, 760, 723. ^{1}H NMR (400 MHz, $CDCl_3$) δ 8.41 (ddd, *J* = 5.0, 1.9, 1.0 Hz, 1H, Ar–H), 7.46 (ddd, *J* = 8.1, 7.3, 1.9 Hz, 1H, Ar–H), 7.16 (dt, *J* = 8.1, 1.1 Hz, 1H, Ar–H), 6.97 (ddd, *J* = 7.3, 4.9, 1.1 Hz, 1H, Ar–H), 3.70 (s, 3H, OCH_3), 3.43 (t, *J* = 7.1 Hz, 2H, SCH_2), 2.79 (t, *J* = 7.1 Hz, 2H, SCH_2CH_2). ^{13}C NMR (101 MHz, $CDCl_3$) δ 172.7 (C=O), 158.3 (Ar–C_q), 149.6 (Ar–C), 136.0 (Ar–C), 122.5 (Ar–C), 119.6 (Ar–C), 51.9 (OCH_3), 34.7 (SCH_2), 25.0 (SCH_2CH_2). Analytical data (NMR) in agreement with those reported in the literature.[12]

Methyl 3-(isopropylthio)propanoate (S1f)

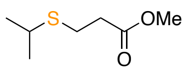

2-iodopropane (3.6 mL, 36 mmol, 2 equiv) was added to methyl 3-sulfanylpropanoate (2.0 mL, 18 mmol, 1 equiv) and K_2CO_3 (2.99 g, 22 mmol, 1.2 equiv) in acetone (90 mL, 0.2 M) at 25 °C and stirred for 24 h. The reaction mixture was quenched with 1 M K_2CO_3 (50 mL) and extracted with CH_2Cl_2 (3 × 100 mL). The combined organic layers were dried (Na_2SO_4), filtered, and concentrated under reduced pressure. Purification by flash column chromatography (SiO_2, 10% Et_2O in pentane) afforded sulfide **S1f** (5.09 g, 84%) as a colourless oil. R_f 0.30 (10% Et_2O in pentane). IR (film)/cm^{-1} 2960, 2930, 2870, 1737

(C=O), 1439, 1357, 1244, 1170, 1051, 1021, 980. ^{1}H NMR (400 MHz, $CDCl_3$) δ 3.69 (s, 3H, OCH_3), 2.94 (p, J = 6.7 Hz, 1H, SCH), 2.80 (td, J = 7.4, 0.7 Hz, 2H, SCH_2), 2.63–2.56 (m, 2H, SCH_2CH_2), 1.26 (d, J = 6.7 Hz, 6H, CH(C*H*$_3$)$_2$). ^{13}C NMR (101 MHz, $CDCl_3$) δ 172.6 (C=O), 51.9 (OCH_3), 35.1 (SCH), 34.9 (SCH_2), 25.6 (SCH_2*C*H_2), 23.4 (CH(*C*H_3)$_2$). HRMS (ESI) m/z Calcd for $C_7H_{15}O_2S$ $[M+H]^+$: 163.0793; Found: 163.0791.

Methyl 3-(methylthio)propanoate (S1g)

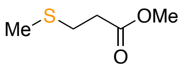

Iodomethane (5.6 mL, 90 mmol, 2 equiv) was added to methyl 3-sulfanylpropanoate (5.0 mL, 45 mmol, 1 equiv) and K_2CO_3 (7.46 g, 54 mmol, 1.2 equiv) in acetone (200 mL, 0.2 M) at 25 °C and stirred for 24 h. The reaction mixture was quenched with 1 M K_2CO_3 (100 mL) and extracted with CH_2Cl_2 (3 × 200 mL). The combined organic layers were dried (Na_2SO_4), filtered, and concentrated under reduced pressure. Purification by flash column chromatography (SiO_2, 5% Et_2O in pentane) afforded sulfide **S1g** (5.09 g, 84%) as a colourless oil. **Note: due to the volatility of the product, removal of solvent was carried out at pressures no less than 200 mbar at 25 °C** R_f 0.25 (5% Et_2O in pentane). IR (film)/cm^{-1} 2952, 2918, 1733 (C=O), 1435, 1357, 1245, 1144, 1021. ^{1}H NMR (400 MHz, $CDCl_3$) δ 3.70 (s, 3H, OCH_3), 2.78–2.75 (m, 2H, SCH_2), 2.64–2.61 (m, 2H, SCH_2C*H*$_2$), 2.12 (s, 3H, SCH_3). ^{13}C NMR (101 MHz, $CDCl_3$) δ 172.6 (C=O), 51.9 (OCH_3), 34.4 (SCH_2), 29.2 (SCH_2*C*H_2), 15.6 (SCH_3). Analytical data (NMR) in agreement with those reported in the literature.[13]

Sulfide Oxidation

Methyl 3-(*p*-tolylsulfinyl)propanoate (S2a)

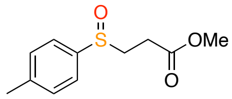

*m*CPBA (3.30 g, 19.1 mmol, 1 equiv) was added portionwise to sulfide **S1a** (4.02 g, 19.1 mmol, 1 equiv) in CH_2Cl_2 (100 mL, 0.2 M) at 0 °C and stirred for 2 hr. The reaction mixture was quenched with 1 M K_2CO_3 (100 mL) and the aqueous mixture was extracted with CH_2Cl_2 (3 × 80 mL). The combined organic layers were dried (Na_2SO_4), filtered, and concentrated under reduced pressure to afford sulfoxide **S2a** (3.97 g, 92%) as a pale-yellow oil. IR (film)/cm^{-1} 2952, 2922, 1733 (C=O), 1595, 1494, 1439, 1358, 1234, 1170, 1085, 1040, 977, 809. ^{1}H NMR (400 MHz, $CDCl_3$) δ 7.49 (d, J = 8.2 Hz, 2H, 2 × Ar–H), 7.36–7.28 (m, 2H, 2 × Ar–H), 3.65 (s, 3H, OCH_3), 3.20 (ddd, J = 13.3, 8.6, 6.7 Hz, 1H, SC*H*H), 2.94 (ddd, J = 13.3, 8.5, 5.7 Hz, 1H, SCH*H*), 2.81 (ddd, J = 17.2, 8.5, 6.6 Hz, 1H, SCH_2C*H*H), 2.53 (ddd, J = 17.2, 8.6, 5.7 Hz, 1H, SCH_2CH*H*), 2.41 (s, 3H, Ar–CH_3). ^{13}C NMR (101 MHz, $CDCl_3$) δ 171.8 (C=O), 141.8 (Ar–C_q), 139.7 (Ar–C_q), 130.1 (2 × Ar–C), 124.2 (2 × Ar–C), 52.2 (OCH_3), 51.3 (SCH_2), 26.1 (SCH_2CH_2), 21.5 (Ar–CH_3). Analytical data (NMR) in agreement with those reported in the literature.[14]

Methyl 3-(phenylsulfinyl)propanoate (S2b)

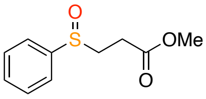

*m*CPBA (131 mg, 0.76 mmol, 1 equiv) was added portionwise to sulfide **S1b** (150 mg, 0.76 mmol, 1 equiv) in CH_2Cl_2 (5 mL, 0.2 M) at 0 ˚C and stirred for 2 h. The reaction mixture was quenched with 3 M KOH (15 mL) and the aqueous mixture was extracted with CH_2Cl_2 (3 × 15 mL). The combined organic layers were dried (Na_2SO_4), filtered, and concentrated under reduced pressure. Purification by flash column chromatography (50% EtOAc in pentane) afforded sulfoxide **S2b** (113 mg, 70%) as a colourless oil. R_f 0.17 (50% EtOAc in pentane). IR (film)/cm^{-1} 3316, 3054, 2949, 2901, 1721 (C=O), 1591, 1440, 1197, 1183, 819, 756. ^{1}H NMR (400 MHz, $CDCl_3$) δ 7.67–7.60 (m, 2H, 2 × Ar–H), 7.58–7.48 (m, 3H, 3 × Ar–H), 3.67 (s, 3H, OCH_3), 3.25 (ddd, *J* = 13.4, 8.5, 6.7 Hz, 1H, SC*H*H), 2.97 (ddd, *J* =

13.3, 8.3, 5.7 Hz, 1H, SCH*H*), 2.85 (ddd, *J* = 17.2, 8.4, 6.7 Hz, 1H, SCH_2*C*HH), 2.57 (ddd, *J* = 17.2, 8.5, 5.7 Hz, 1H, SCH_2CH*H*). ^{13}C NMR (101 MHz, $CDCl_3$) δ 171.8 (C=O), 143.0 (Ar–C_q), 131.3 (Ar–C), 129.4 (2 × Ar–C), 124.1 (2 × Ar–C), 52.3 (OCH_3), 51.2 (SCH_2), 26.1 (SCH_2CH_2). Analytical data (NMR) in agreement with those reported in the literature.[15]

Methyl 3-((4-fluorophenyl)sulfinyl)propanoate (S2c)

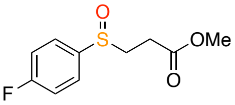

*m*CPBA (2.83 g, 16.4 mmol, 1 equiv) was added portionwise to sulfide **S1c** (3.50 g, 16.4 mmol, 1 equiv) in CH_2Cl_2 (80 mL, 0.2 M) at 0 °C and stirred for 4 hr. The reaction mixture was quenched with 1 M K_2CO_3 (100 mL) and the aqueous mixture was extracted with CH_2Cl_2 (3 × 80 mL). The combined organic layers were dried (Na_2SO_4), filtered, and concentrated under reduced pressure to afford sulfoxide **S2c** (3.76 g, quant.) as a pale-yellow oil. R_f 0.20 (50% EtOAc in pentane). IR (film)/cm^{-1} 2997, 2952, 1773 (C=O), 1588, 1491, 1439, 1357, 1219, 1174, 1219, 1044, 977, 835, 749. ^{1}H NMR (400 MHz, $CDCl_3$) δ 7.70–7.53 (m, 2H, 2 × Ar–H), 7.24–7.19 (m, *J* = 8.5 Hz, 2H, 2 × Ar–H), 3.64 (s, 3H, OCH_3), 3.25–3.15 (m, 1H, SC*H*H), 2.93 (ddd, *J* = 13.7, 7.9, 6.0 Hz, 1H, SCH*H*), 2.85–2.77 (m, 1H, SCH_2C*H*H), 2.59–2.50 (m, 1H, SCH_2CH*H*). ^{13}C NMR (101 MHz, $CDCl_3$) δ 171.6 (C=O), 164.5 (d, $^1J_{C\text{-}F}$ = 251.9 Hz, Ar–F), 138.5 (Ar-C_q), 126.4 (d, $^3J_{C\text{-}F}$ = 8.8 Hz, 2 × Ar–C), 116.8 (d, $^2J_{C\text{-}F}$ = 22.6 Hz, 2 × Ar–C), 52.3 (OCH_3), 51.5 (SCH_2), 26.0 (SCH_2*C*H_2). ^{19}F{^{1}H} NMR (377 MHz, $CDCl_3$) δ -108.3 (1F, Ar–F). HRMS (ESI) *m/z* Calcd for $C_{10}H_{12}O_3SF$ $[M+H]^+$: 231.0491; Found: 231.0481.

Methyl 3-((4-methoxyphenyl)sulfinyl)propanoate (S2d)

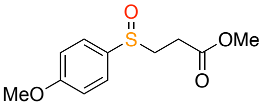

*m*CPBA (2.83 g, 16.4 mmol, 1 equiv) was added portionwise to sulfide **S1d** (3.70 g, 16.4 mmol, 1 equiv) in CH_2Cl_2 (80 mL, 0.2 M) at 0 °C and stirred for 4 hr. The reaction mixture was quenched with 1 M K_2CO_3 (100 mL) and the aqueous mixture was extracted with CH_2Cl_2 (3 × 80 mL). The combined organic layers were dried (Na_2SO_4), filtered, and concentrated under reduced pressure to afford sulfoxide **S2d** (3.91 g, 99%) as a yellow oil. R_f 0.14 (50% EtOAc in pentane). IR (film)/cm^{-1} 2952, 2840, 1733 (C=O), 1595, 1495, 1461, 1357, 1245, 1170, 1025, 828, 753. ^{1}H NMR (400 MHz, $CDCl_3$) δ 7.45–7.42 (m, 2H, 2 × Ar–H), 6.93–6.90 (m, 2H, 2 × Ar–H), 3.73 (s, 3H, OCH_3), 3.53 (s, 3H, OCH_3), 3.06 (ddd, *J* = 13.4, 8.5, 6.8 Hz, 1H, SC*H*H), 2.90–2.81 (m, 1H, SCH*H*), 2.67 (ddd, *J* = 17.2, 8.5, 6.8 Hz, 1H, SCH_2C*H*H), 2.43 (ddd, *J* = 17.2, 8.5, 5.9 Hz, 1H, SCH_2CH*H*). ^{13}C NMR (101 MHz, $CDCl_3$) δ 171.4 (C=O), 161.9 (Ar–C_q), 133.5 (Ar–C_q), 125.7 (2 × Ar–C), 114.7 (2 × Ar–C), 55.4 (Ar–OCH_3), 51.9 (COO*C*H_3), 51.03 (SCH_2), 25.89 (SCH_2*C*H_2). HRMS (ESI) *m/z* Calcd for $C_{11}H_{15}O_4S$ $[M+H]^+$: 243.0691; Found: 243.0691.

Methyl 3-(pyridin-2-ylsulfinyl)propanoate (S2e)

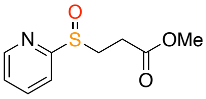

*m*CPBA (2.62 g, 15.2 mmol, 1 equiv) was added portionwise to sulfide **S1e** (3.00 g, 15.2 mmol, 1 equiv) in CH_2Cl_2 (80 mL, 0.2 M) at 0 °C and stirred for 4 hr. The reaction mixture was quenched with 1 M K_2CO_3 (100 mL) and the aqueous mixture was extracted with CH_2Cl_2 (3 × 80 mL). The combined organic layers were dried (Na_2SO_4), filtered, and concentrated under reduced pressure to afford sulfoxide **S2e** (1.97 g, 9.3 mmol, 61%) as a pale yellow oil. R_f 0.26 (2% MeOH in CH_2Cl_2). IR (film)/cm^{-1} 3049, 2993, 2952, 1733 (C=O), 1576, 1424, 1356, 1238, 1174, 1084, 1036, 771. ^{1}H NMR (400 MHz, $CDCl_3$) δ 8.64 (ddd, *J* = 4.7, 1.7, 1.0 Hz, 1H, Ar–H), 8.00–7.87 (m, 2H, 2 × Ar–H), 7.39 (ddd, *J* = 7.1, 4.7, 1.7 Hz, 1H, Ar–H), 3.65 (s, 3H, OCH_3), 3.50 (ddd, *J* = 13.6, 9.3, 6.1 Hz, 1H, SC*H*H), 3.20

(ddd, J = 13.7, 9.2, 5.9 Hz, 1H, SCH*H*), 2.86 (ddd, J = 17.1, 9.2, 6.1 Hz, 1H, SCH_2C*H*H), 2.45 (ddd, J = 17.0, 9.3, 5.9 Hz, 1H, SCH_2CH*H*). ^{13}C NMR (101 MHz, $CDCl_3$) δ 171.7 (C=O), 163.9 (Ar–C_q), 150.0 (Ar–C), 138.1 (Ar–C), 124.9 (Ar–C), 120.4 (Ar–C), 52.3 (CH_3), 48.1 (SCH_2), 25.6 (SCH_2CH_2). Analytical data (NMR) in agreement with those reported in the literature.[12]

Methyl 3-(isopropylsulfinyl)propanoate (S2f)

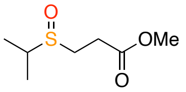

mCPBA (2.56 g, 14.8 mmol, 1 equiv) was added portionwise to sulfide **S1f** (2.40 g, 14.8 mmol, 1 equiv) in CH_2Cl_2 (75 mL, 0.2 M) at 0 °C and stirred for 2 hr. The reaction mixture was quenched with 1 M K_2CO_3 (100 mL) and the aqueous mixture was extracted with CH_2Cl_2 (3 × 80 mL). The combined organic layers were dried (Na_2SO_4), filtered, and concentrated under reduced pressure to afford sulfoxide **S2f** (1.63 g, 62%) as a pale-yellow oil. R_f 0.11 (60% EtOAc in pentane). IR (film)/cm^{-1} 2960, 2874, 1737 (C=O), 1439, 1364, 1234, 1040, 977, 828. ^{1}H NMR (400 MHz, $CDCl_3$) δ 3.73 (s, 3H, OCH_3), 3.03–2.91 (m, 1H, SC*H*(CH_3)$_2$), 2.91–2.74 (m, 4H, 2 × CH_2), 1.33 (d, J = 6.9 Hz, 3H, 1 × CH(C*H*$_3$)$_2$), 1.28 (d, J = 6.9 Hz, 3H, 1 × CH(C*H*$_3$)$_2$). ^{13}C NMR (101 MHz, $CDCl_3$) δ 172.2 (C=O), 52.3 (OCH_3), 51.0 (SCH_2), 43.6 (*C*H(CH_3)$_2$), 27.4 (SCH_2CH_2), 15.9 (1 × CH(*C*H$_3$)$_2$), 15.0 (1 × CH(*C*H$_3$)$_2$). HRMS (APCI) m/z Calcd for $C_7H_{14}O_3S$ [M+H]$^+$: 179.0742; Found: 179.0736.

Methyl 3-(methanesulfinyl)propanoate (S2g)

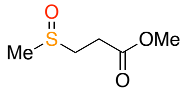

mCPBA (4.49 g, 26.0 mmol, 1 equiv) was added portionwise to sulfide **S1g** (3.47 g, 26.0 mmol, 1 equivl) in CH_2Cl_2 (130 mL, 0.2 M) at 0 °C and stirred for 1 hr. The reaction mixture was quenched with 1 M K_2CO_3 (100 mL) and the aqueous mixture was extracted with CH_2Cl_2 (3 × 80 mL). The combined organic layers were dried (Na_2SO_4), filtered, and concentrated under reduced pressure to afford sulfoxide **S2g** (3.09 g, 79%) as a colourless oil. **Note: volatility was observed under pressures of >1 mbar** IR (film)/cm^{-1} 3000, 2956, 1730 (C=O), 1439, 1364, 1241, 1178, 1029, 943, 827. ^{1}H NMR (400 MHz, $CDCl_3$) δ 3.72 (s, 3H, OCH_3), 3.06 (ddd, J = 13.0, 8.2, 7.1 Hz, 1H, SC*H*H), 2.94–2.87 (m, 1H, SCH*H*), 2.87–2.80 (m, 2H, SCH_2C*H*$_2$), 2.59 (s, 3H, SCH_3). ^{13}C NMR (101 MHz, $CDCl_3$) δ 171.9 (C=O), 52.4 (OCH_3), 49.0 (SCH_2), 38.9 (SCH_2CH_2), 26.9 (SCH_3). Analytical data (NMR) in agreement with those reported in the literature.[13]

<u>NBoc-transfer</u>

Methyl 3-(N-(tert-butoxycarbonyl)-4-methylphenylsulfonimidoyl)propanoate (S3a)

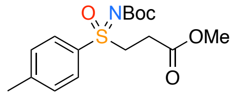

Prepared according to a literature procedure.[6] Magnesium oxide (3.24 g, 80.4 mmol, 4 equiv), *tert*-butyl carbamate (3.53 g, 30.2 mmol, 1.5 equiv), PhI(OAc)$_2$ (9.71 g, 30.2 mmol, 1.5 equiv) and $Rh_2(OAc)_4$ (0.22 g, 0.5 mmol, 2.5 mol%) were added to a stirred solution of sulfoxide **S2a** (4.20 g, 20.1 mmol, 1 equiv) in CH_2Cl_2 (200 mL, 0.1 M) at RT and warmed to 40 °C for 18 h. At RT the reaction mixture was filtered through celite and concentrated under reduced pressure. Purification by flash column chromatography (50% EtOAc in pentane) afforded sulfoximine **S3a** (4.39 g, 19.4 mmol, 97%) as a white solid. mp = 83–84 °C. R_f 0.34 (50% EtOAc in pentane). IR (film)/cm^{-1} 2978, 1740 (C=O), 1670 (C=O), 1439, 1364, 1274, 1252, 1156, 894 ^{1}H NMR (400 MHz, $CDCl_3$) δ 7.80 (d, J = 8.4 Hz, 2H, 2 × Ar–H), 7.37 (d, J = 8.4 Hz, 2H, 2 × Ar–H), 3.69 (ddd, J = 14.3, 9.5, 6.0 Hz, 1H, SC*H*H), 3.61 (s, 3H, OCH_3), 3.55 (ddd, J = 14.2, 9.3, 6.0 Hz, 1H, SCH*H*), 2.89–2.67 (m, 2H, SCH_2C*H*$_2$), 2.46 (s, 3H, Ar–CH_3), 1.37 (s, 9H, C(CH_3)$_3$). ^{13}C NMR (101 MHz, $CDCl_3$) δ 170.3 (C=O), 157.7 (C=O), 145.2 (Ar–C_q), 133.8 (Ar–C_q), 130.5 (2 ×

Ar–C), 128.3 (2 × Ar–C), 80.8 ($C(CH_3)_3$), 52.5 (OCH_3), 51.9 (Ar–CH_3), 28.1 ($C(CH_3)_3$), 27.4 (CH_2), 21.8 (CH_2). HRMS (ESI) *m/z* Calcd for $C_{16}H_{25}NO_5S$ $[M+H]^+$: 342.1370; Found: 342.1375.

Methyl 3-(*N*-(*tert*-butoxycarbonyl)phenylsulfonimidoyl)propanoate (S3b)

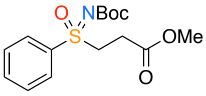

Prepared according to a literature procedure.[6] Magnesium oxide (71 mg, 1.8 mmol, 4 equiv), *tert*-butyl carbamate (77 mg, 0.66 mmol, 1.5 equiv), $PhI(OAc)_2$ (213 mg, 0.66 mmol, 1.5 equiv) and $Rh_2(OAc)_4$ (4.9 mg, 0.011 mmol, 2.5 mol%) were added to a stirred solution of sulfoxide **S2b** (94 mg, 0.44 mmol, 1 equiv) in CH_2Cl_2 (4.4 mL, 0.1 M) at RT and warmed to 40 °C for 18 h. At RT the reaction mixture was filtered through celite and concentrated under reduced pressure. Purification by flash column chromatography (50% EtOAc in pentane) afforded sulfoximine **S3b** (114 mg, 80%) as a colourless oil. R_f 0.43 (50% EtOAc in pentane). IR (film)/cm^{-1} 2987, 2940, 1738 (C=O), 1666, 1447, 1408, 1367, 1215, 1153, 1133, 872, 740. ^1H NMR (400 MHz, $CDCl_3$) δ 8.00–7.93 (m, 2H, 2 × Ar–H), 7.73–7.67 (m, 1H, Ar–H), 7.65–7.58 (m, 2H, 2 × Ar–H), 3.73 (ddd, *J* = 14.4, 9.4, 6.0 Hz, 1H, SC*H*H), 3.63 (s, 3H, OCH_3), 3.61–3.54 (m, 1H, SCH*H*), 2.92–2.72 (m, 2H, SCH_2CH_2), 1.37 (s, 9H, $C(CH_3)_3$). ^{13}C NMR (101 MHz, $CDCl_3$) δ 170.3 (C=O), 157.5 (C=O), 137.1 (Ar–C_q), 134.1 (Ar–C), 129.8 (2 × Ar–C), 128.2 (2 × Ar–C), 80.9 ($C(CH_3)_3$), 52.5 (OCH_3), 51.8 (CH_2), 28.1 ($C(CH_3)_3$), 27.4 (CH_2). HRMS (ESI) *m/z* Calcd for $C_{15}H_{22}NO_5S$ $[M+H]^+$: 328.1219; Found: 328.1213.

Methyl 3-(*N*-(*tert*-butoxycarbonyl)-4-fluorophenylsulfonimidoyl)propanoate (S3c)

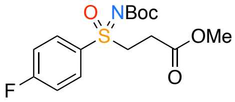

Prepared according to a literature procedure.[6] Magnesium oxide (2.63 g, 65.2 mmol, 4 equiv), *tert*-butyl carbamate (2.87 g, 24.5 mmol, 1.5 equiv), $PhI(OAc)_2$ (7.87 g, 24.5 mmol, 1.5 equiv) and $Rh_2(OAc)_4$ (0.18 g, 0.4 mmol, 2.5 mol%) were added to a stirred solution of sulfoxide **S2c** (3.76 g, 16.3 mmol, 1 equiv) in CH_2Cl_2 (162 mL, 0.1 M) at RT and warmed to 40 °C for 18 h. At RT the reaction mixture was filtered through celite and concentrated under reduced pressure. Purification by flash column chromatography (30% EtOAc in pentane) afforded sulfoximine **S3c** (3.63 g, 64%) as a viscous yellow oil. R_f 0.22 (30% EtOAc in pentane). IR (film)/cm^{-1} 2978, 1737 (C=O), 1666 (C=O), 1588, 1491, 1364, 1275, 1226, 1144, 895, 835, 731. ^1H NMR (400 MHz, $CDCl_3$) δ 7.96–7.89 (m, 2H, 2 × Ar–H), 7.29–7.23 (m, 2H, 2 × Ar–H), 3.74–3.65 (m, 1H, SC*H*H), 3.60 (s, 3H, OCH_3), 3.59–3.53 (m, 1H, SCH*H*), 2.78 (ddd, *J* = 15.5, 8.9, 6.3 Hz, 2H, SCH_2CH_2), 1.35 (s, 9H,$C(CH_3)_3$).^{13}C NMR (101 MHz, $CDCl_3$) δ 170.1 (C=O), 166.0 (d, $^1J_{C-F}$ = 257.2 Hz, Ar–F), 157.3 (C=O), 132.7 (Ar–C_q), 131.0 (d, $^3J_{C-F}$ = 9.6 Hz, 2 × Ar–C), 117.1 (d, $^2J_{C-F}$ = 22.8 Hz, 2 × Ar–C), 80.9 ($C(CH_3)_3$), 52.4 (OCH_3), 51.8 (CH_2), 28.0 (CH_2), 27.2 ($C(CH_3)_3$). $^{19}F\{^1H\}$ NMR (377 MHz, $CDCl_3$) δ -103.0 (1F, Ar–F). HRMS (ESI) *m/z* Calcd for $C_{15}H_{21}NO_5SF$ $[M+H]^+$: 346.1124; Found: 346.1130.

Methyl 3-(*N*-(tert-butoxycarbonyl)-4-methoxyphenylsulfonimidoyl)propanoate (S3d)

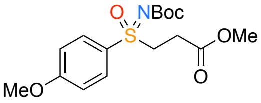

Prepared according to a literature procedure.[6] Magnesium oxide (2.60 g, 64.4 mmol, 4 equiv), *tert*-butyl carbamate (2.84 g, 24.2 mmol, 1.5 equiv), $PhI(OAc)_2$ (7.80 g, 24.2 mmol, 1.5 equiv) and $Rh_2(OAc)_4$ (0.18 g, 0.4 mmol, 2.5 mol%) were added to a stirred solution of sulfoxide **S2d** (3.90 g, 16.1 mmol, 1 equiv) in CH_2Cl_2 (161 mL, 0.1 M) at RT and warmed to 40 °C for 18 h. At RT the reaction mixture was filtered through celite and concentrated under reduced pressure. Purification by flash column chromatography (60% Et_2O in Hexane) afforded sulfoximine **S3d** (3.62 g, 63%) as a pale pink solid. mp = 93–95 °C. R_f 0.12 (60% Et_2O in Hexane). IR (film)/cm^{-1} 2974, 1737 (C=O),

1666 (C=O), 1592, 1498, 1364, 1245, 1148, 1107, 891, 731. ^{1}H NMR (400 MHz, $CDCl_3$) δ 7.83 (d, *J* = 8.8 Hz, 2H, 2 × Ar–H), 7.02 (d, *J* = 8.8, 2H, 2 × Ar–H), 3.87 (s, 3H, OCH_3), 3.69–3.63 (m, 1H, SC*H*H), 3.59 (s, 3H, OCH_3), 3.57–3.51 (m, 1H, SCH*H*), 2.79–2.71 (m, 2H, SCH_2CH_2), 1.36 (s, 9H, $C(CH_3)_3$). ^{13}C NMR (101 MHz, $CDCl_3$) δ 170.3 (C=O), 164.1 (Ar–C_q), 157.7 (C=O), 130.4 (2 × Ar–C), 127.6 (Ar–C_q), 115.0 (2 × Ar–C), 80.6 ($C(CH_3)_3$), 55.9 (OCH_3), 52.4 (OCH_3), 52.0 (CH_2), 28.1 (CH_2), 27.5 ($C(CH_3)_3$). HRMS (ESI) *m/z* Calcd for $C_{16}H_{24}NO_6S$ $[M+H]^+$ 358.1324; Found: 358.1340.

Methyl 3-(*N*-(*tert*-butoxycarbonyl)pyridine-2-sulfonimidoyl)propanoate (S3e)

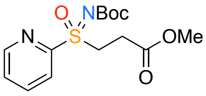

Prepared according to a literature procedure.[6] Magnesium oxide (1.48 g, 36.8 mmol, 4 equiv), *tert*-butyl carbamate (1.62 g, 13.8 mmol, 1.5 equiv), $PhI(OAc)_2$ (4.44 g, 13.8 mmol, 1.5 equiv) and $Rh_2(OAc)_4$ (0.10 g, 0.2 mmol, 2.5 mol%) were added to a stirred solution of sulfoxide **S2e** (1.97 g, 9.2 mmol, 1 equiv) in CH_2Cl_2 (92 mL, 0.1 M) at RT and warmed to 40 °C for 18 h. At RT the reaction mixture was filtered through celite and concentrated under reduced pressure. Purification by flash column chromatography (1% MeOH in CH_2Cl_2) afforded sulfoximine **S3e** (0.97 g, 64%) as a pale yellow oil. R_f 0.14 (1% MeOH in CH_2Cl_2). IR (film)/cm^{-1} 3056, 2978, 1737 (C=O), 1700, 1662 (C=O), 1580, 1429, 1364, 1275, 1230, 1148, 895, 864, 764. ^{1}H NMR (400 MHz, $CDCl_3$) δ 8.71 (ddd, *J* = 4.7, 1.7, 0.8 Hz, 1H, Ar–H), 8.20 (dt, *J* = 7.9, 1.0 Hz, 1H, Ar–H), 7.98 (td, *J* = 7.8, 1.7 Hz, 1H, Ar–H), 7.55 (ddd, *J* = 7.7, 4.7, 1.1 Hz, 1H, Ar–H), 3.98–3.79 (m, 2H, SCH_2CH_2), 3.62 (s, 3H, OCH_3), 2.91 (ddd, *J* = 17.3, 8.9, 6.3 Hz, 1H, SC*H*H), 2.76 (ddd, *J* = 17.3, 9.1, 6.3 Hz, 1H, SCH*H*), 1.33 (s, 9H, $C(CH_3)_3$). ^{13}C NMR (101 MHz, $CDCl_3$) δ 170.3 (C=O), 157.5 (C=O), 155.8 (Ar–C_q), 150.3 (Ar–C), 138.3 (Ar–C), 127.5 (Ar–C), 124.4 (Ar–C), 80.8 ($C(CH_3)_3$), 52.4 (OCH_3), 47.8 (CH_2), 28.0 ($C(CH_3)_3$), 27.2 (CH_2). HRMS (ESI) *m/z* Calcd for $C_{14}H_{21}N_2O_5S$ $[M+H]^+$ 329.1171; Found: 329.1168.

Methyl 3-(*N*-(*tert*-butoxycarbonyl)propan-2-ylsulfonimidoyl)propanoate (S3f)

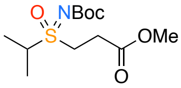

Prepared according to a literature procedure.[6] Magnesium oxide (1.45 g, 36.4 mmol, 4 equiv), *tert*-butyl carbamate (1.60 g, 13.7 mmol, 1.5 equiv), $PhI(OAc)_2$ (4.40 g, 13.7 mmol, 1.5 equiv) and $Rh_2(OAc)_4$ (0.10 g, 0.2 mmol, 2.5 mol%) were added to a stirred solution of sulfoxide **S2f** (1.62 g, 9.1 mmol, 1 equiv) in CH_2Cl_2 (91 mL, 0.1 M) at RT and warmed to 40 °C for 18 h. At RT, the reaction mixture was filtered through celite and concentrated under reduced pressure. Purification by flash column chromatography (50% EtOAc in pentane) afforded sulfoximine **S3f** (1.15 g, 43%) as a white solid. mp = 93–94 °C. R_f 0.30 (50% EtOAc in pentane). IR (film)/cm^{-1} 2982, 1737 (C=O), 1655 (C=O), 1364, 1279, 1249, 1156, 1092, 1054, 891, 848. ^{1}H NMR (400 MHz, $CDCl_3$) δ 3.79–3.69 (m, 4H, $CH(CH_3)_3$ and OCH_3), 3.69–3.51 (m, 2H, SCH_2), 2.98 (ddd, *J* = 8.1, 6.8, 4.0 Hz, 2H, SCH_2CH_2), 1.52–1.44 (m, 15H, $C(CH_3)_3$ and $CH(CH_3)_2$). ^{13}C NMR (101 MHz, $CDCl_3$) δ 171.2 (C=O), 158.8 (C=O), 80.5 ($C(CH_3)_3$), 54.3 ($CH(CH_3)_2$), 52.6 (OCH_3), 43.2 (CH_2), 28.3 ($C(CH_3)_3$), 27.2 (CH_2), 15.8 (1 × $CH(CH_2)_2$), 15.7 (1 × $CH(CH_2)_2$). HRMS (ESI) *m/z* Calcd for $C_{12}H_{24}NO_5S$ $[M+H]^+$ 294.1375; Found: 294.1381.

Methyl 3-(*N*-(*tert*-butoxycarbonyl)-*S*-methylsulfonimidoyl)propanoate (S3g)

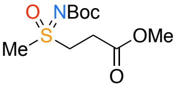

Prepared according to a literature procedure.[6] Magnesium oxide (2.58 g, 64 mmol, 4 equiv), *tert*-butyl carbamate (2.81 g, 24 mmol, 1.5 equiv), $PhI(OAc)_2$ (7.73 g, 24 mmol,

1.5 equiv) and $Rh_2(OAc)_4$ (0.18 g, 0.4 mmol, 2.5 mol%) were added to a stirred solution of sulfoxide **S2g** (2.46 g, 16 mmol, 1 equiv) in CH_2Cl_2 (160 mL, 0.1 M) at RT and warmed to 40 °C for 18 h. At RT the reaction mixture was filtered through celite and concentrated under reduced pressure. Purification by flash column chromatography (50% EtOAc in pentane) afforded sulfoximine **S3g** (3.39 g, 14.0 mmol, 87%) as a pale-yellow oil. R_f 0.28 (50% EtOAc in pentane). IR (film)/cm^{-1} 2978, 2933, 1737 (C=O), 1655 (C=O), 1439, 1364, 1275, 1250, 1152, 992, 861,790. ^{1}H NMR (400 MHz, $CDCl_3$) δ 3.78 (dd, J = 14.3, 7.1 Hz, 1H, SC*H*H), 3.74 (s, 3H, OCH_3), 3.65 (dt, J = 14.4, 7.2 Hz, 1H, SCH*H*), 3.24 (s, 3H, SCH_3), 3.10–2.86 (m, 2H, SCH_2CH_2), 1.48 (s, 9H, $C(CH_3)_3$). ^{13}C NMR (101 MHz, $CDCl_3$) δ 170.9 (C=O), 158.5 (C=O), 80.9 ($C(CH_3)_3$), 52.7 (OCH_3), 49.5 (CH_2), 40.8 (CH_2), 28.3 ($C(CH_3)_3$), 27.7 (SCH_3). HRMS (ESI) m/z Calcd for $C_{10}H_{19}NO_5S$ $[M+H]^+$: 266.1060; Found: 266.1062.

Elimination to Sulfinamide Salt

Sodium (*tert*-butoxycarbonyl)(*p*-tolylsulfinyl)amide ((*rac*)-1a)

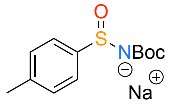

NaH (60% in oil, 526 mg, 13.1 mmol) was added to sulfoximine **S3a** (4.28 g, 12.5 mmol) in THF (125 mL) at 25 °C and stirred for 3 h. The reaction was quenched with MeOH (25 μL) and concentrated under reduced pressure. The precipitate was collected by filtration and washed with hexane to give sulfinamide salt **(*rac*)-1a** (3.46 g, quant. yield) as a white solid. The data was identical to that shown for **(*S*)-1a** above.

Sodium (*tert*-butoxycarbonyl)(phenylsulfinyl)amide (S4b)

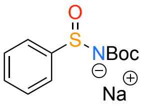

NaH (60 % in oil, 137 mg, 3.44 mmol, 1.05 equiv) was added to a stirred solution of sulfoximine **S3b** (1.07 g, 3.27 mmol, 1.0 equiv) in anhydrous THF (33 mL, 0.1 M) at 25 °C and stirred for 1 h. MeOH (100 mL) was added and the solvent removed under reduced pressure. The resulting precipitate was washed with hexane (50 mL) and collected by filtration to afford the sulfinamide salt **S4b** as a white solid (852 mg, 99%). mp = 238–240 °C. IR (film)/cm^{-1} 3344, 2978, 1631 (C=O), 1582, 1268, 1155, 1004, 993, 829, 747, 697. ^{1}H NMR (400 MHz, D_2O) δ 7.63 (dd, *J* = 6.7, 3.0 Hz, 2H, 2 × Ar–H), 7.54–7.47 (m, 3H, 3 × Ar–H), 1.39 (s, 9H, $C(CH_3)_3$). ^{13}C NMR (101 MHz, D_2O) δ 165.9 (C=O), 146.1 (Ar–C_q), 130.9 (Ar–C), 129.0 (2 × Ar–C), 124.6 (2 × Ar–C), 79.6 ($C(CH_3)_3$), 27.7 ($C(CH_3)_3$). HRMS (ESI) *m/z*: Calcd for $C_{11}H_{14}NO_3S$ $[M]^-$: 240.0694; Found: 240.0704.

Sodium (*tert*-butoxycarbonyl)((4-fluorophenyl)sulfinyl)amide (S4c)

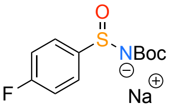

NaH (60% in oil, 442 mg, 11.0 mmol, 1.05 equiv) was added to sulfoximine **S3c** (3.60 g, 10.5 mmol, 1 equiv) in THF (100 mL) at 25 °C and stirred for 3 h. The reaction was quenched with MeOH (25 μL) and concentrated under reduced pressure. The precipitate was collected by filtration and washed with hexane to give sulfinamide salt **S4c** (1.91 g, 65%) as a white solid. mp = 162–164 °C. IR (film)/cm-1 2982, 2933, 1644 (C=O), 1588, 1484, 1453, 1394, 1275, 1219, 1167, 999, 895, 831, 794, 761. ^{1}H NMR (400 MHz, D_2O) δ 7.72–7.62 (m, 2H, 2 × Ar–H), 7.25 (t, J = 8.9 Hz, 2H, 2 × Ar–H), 1.41 (s, 9H, $C(CH_3)_3$). ^{13}C NMR (101 MHz, D_2O) δ 165.7 (C=O), 163.9 (d, $^{1}J_{C-F}$ = 247.8 Hz, Ar–C_q), 127.1 (d, $^{3}J_{C-F}$ = 9.3 Hz, 2 × Ar–C), 126.5 (Ar–C_q), 116.1 (d, $^{2}J_{C-F}$ = 23.5 Hz, 2 × Ar–C), 79.8 ($C(CH_3)_3$), 27.8 ($C(CH_3)_3$). ^{19}F NMR (377 MHz, CD_3OD) δ -114.03 (1F, Ar–F). HRMS (ESI) m/z Calcd for $C_{11}H_{13}FNO_3S$ $[M]^-$: 258.0600; Found: 258.0594.

Sodium (*tert*-butoxycarbonyl)((4-methoxyphenyl)sulfinyl)amide (S4d)

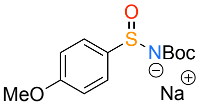

NaH (60% in oil, 425 mg, 10.6 mmol, 1.05 equiv) was added to sulfoximine **S3d** (3.61 g, 10.1 mmol, 1 equiv) in THF (100 mL) at 25 °C and stirred for 3 h. The reaction was quenched with MeOH (25 μL) and concentrated under reduced pressure. The precipitate was collected by filtration and washed with hexane to give sulfinamide salt **S4d** (2.69 g, 91%) as a white solid. mp = 219–220 °C. IR (film)/cm^{-1} 2982, 2933, 1633 (C=O), 1595, 1491, 1457, 1252, 1159, 1081, 1033, 999, 832. ^{1}H NMR (400 MHz, D_2O) δ 7.61 (d, *J* = 8.9 Hz, 2H, 2 × Ar–H), 7.09 (d, *J* = 8.9 Hz, 2H, 2 × Ar–H), 3.86 (s, 3H, OCH_3), 1.41 (s, 9H, $C(CH_3)_3$). ^{13}C NMR (101 MHz, D_2O) δ 165.6 (C=O), 160.9 ($Ar–C_q$), 138.3 ($Ar–C_q$), 126.6 (2 × Ar–C), 114.5 (2 × Ar–C), 79.6 ($\mathit{C}(CH_3)_3$), 55.5 (OCH_3), 27.8 ($C(\mathit{C}H_3)_3$). HRMS (ESI) m/z Calcd for $C_{12}H_{16}NO_4S$ $[M]^-$: 270.0800; Found: 270.0804.

Sodium (*tert*-butoxycarbonyl)(pyridin-2-ylsulfinyl)amide (S4e)

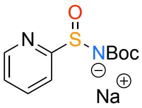

NaH (60% in oil, 370 mg, 9.2 mmol, 1.05 equiv) was added to sulfoximine **S3e** (3.02 g, 8.8 mmol, 1 equiv) in THF (90 mL) at 25 °C and stirred for 24 h. The reaction was quenched with MeOH (25 μL) and concentrated under reduced pressure. The precipitate was collected by filtration and washed with hexane to give sulfinamide salt **S4e** (2.64 g, quant) as a white solid. mp = 191–194 °C. IR (film)/cm^{-1} 3015, 2978, 2933, 1629 (C=O), 1573, 1454, 1368, 1290, 1156, 1085, 999, 835, 798, 760. ^{1}H NMR (400 MHz, D_2O) δ 8.53 (ddd, *J* = 4.9, 1.8, 0.9 Hz, 1H, Ar–H), 8.02 (td, *J* = 7.8, 1.7 Hz, 1H, Ar–H), 7.87 (dt, *J* = 8.0, 1.1 Hz, 1H, Ar–H), 7.52 (ddd, *J* = 7.7, 4.9, 1.2 Hz, 1H, Ar–H), 1.41 (s, 9H, $C(CH_3)_3$). ^{13}C NMR (101 MHz, D_2O) δ 166.2 (C=O), 164.9 ($Ar–C_q$), 148.9 (Ar–C), 139.3 (Ar–C), 125.7 (Ar–C), 119.6 (Ar–C), 79.9 ($\mathit{C}(CH_3)_3$), 27.7 ($C(\mathit{C}H_3)_3$).HRMS (ESI) m/z Calcd for $C_{10}H_{13}N_2O_3S$ $[M]^-$: 241.0647; Found: 241.0641.

Sodium (*tert*-butoxycarbonyl)(isopropylsulfinyl)amide (S4f)

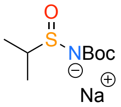

NaH (60% in oil, 164 mg, 4.1 mmol, 1.05 equiv) was added to sulfoximine **S3f** (1.15 g, 3.9 mmol, 1 equiv) in THF (40 mL) at 25 °C and stirred for 24 h. The reaction was quenched with MeOH (25 μL) and concentrated under reduced pressure. The precipitate was collected by filtration and washed with hexane to give sulfinamide salt **S4f** (1.06 g, quant.) as a white solid. mp = 199–200 °C. IR (film)/cm^{-1} 2930, 2986, 2870, 1606 (C=O), 1464, 1297, 1256, 1170, 1074, 988, 905, 746, 664. ^{1}H NMR (400 MHz, CD_3OD) δ 2.51–2.44 (m, 1H, SCH), 1.39 (s, 9H, $C(CH_3)_3$), 1.17 (d, *J* = 7.0 Hz, 3H, 1 × $CH(C\mathit{H}_3)_2$), 1.11 (d, *J* = 7.0 Hz, 3H, 1 × $CH(C\mathit{H}_3)_2$). ^{13}C NMR (101 MHz, MeOD) δ 167.2 (C=O), 78.4 ($\mathit{C}(CH_3)_3$), 54.1 ($\mathit{C}H(CH_3)_2$), 29.3 ($C(\mathit{C}H_3)_3$), 16.7 (1 × $CH(\mathit{C}H_3)_2$), 16.3 (1 × $CH(\mathit{C}H_3)_2$). HRMS (ESI) m/z Calcd for $C_8H_{16}NO_3S$ $[M]^-$: 206.0851; Found: 206.0847.

Sodium (*tert*-butoxycarbonyl)(methylsulfinyl)amide (S4g)

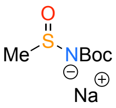

NaH (60% in oil, 0.34 g, 8.4 mmol, 1.05 equiv) was added to sulfoximine **S3g** (2.16 g, 8.0 mmol, 1 equiv) in THF (0.1 M) at 25 °C and stirred for 2 h. The reaction was quenched with MeOH (20 μL) and concentrated under reduced pressure. The precipitate was collected by filtration and washed with hexane to give sulfinamide salt **S4g** (1.44 g, 7.2 mmol, 90%) as a white solid. mp = 183–188 °C. IR (film)/cm^{-1} 2974, 2930, 1610 (C=O), 1364, 1286, 1163, 999, 835, 756. ^{1}H NMR (400 MHz, D_2O) δ 2.40 (s,

3H, SCH_3), 1.38 (s, 9H, ($C(CH_3)_3$). ^{13}C NMR (101 MHz, D_2O) δ 79.4 (*C*(CH_3)$_3$), 40.6 (SCH_3), 27.7 (C(CH_3)$_3$). HRMS (ESI) m/z Calcd for $C_6H_{12}NO_3S$ $[M]^-$: 178.0538; Found: 178.0542.

Synthesis of Sulfonimidoyl Fluorides (2c-2h)

tert-Butyl (fluoro(oxo)(phenyl)-λ^6-sulfaneylidene)carbamate (2c)

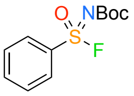

Prepared according to General Procedure A. Selectfluor (81 mg, 0.23 mmol, 1.5 equiv) was added to a solution of sulfinamide salt **S4b** (40 mg, 0.15 mmol) in DMF (0.75 mL, 0.2 M) at 0 °C and warmed to 25 °C for 18 h. H_2O (25 mL) was added and the aqueous mixture extracted with EtOAc (3 × 25 mL). The combined organic layers were dried (Na_2SO_4), filtered and the solvent removed under reduced pressure. Purification by flash column chromatography (SiO_2, 10% EtOAc in pentane) afforded sulfonimidoyl fluoride **2c** (21 mg, 54%) as a colourless oil. R_f 0.51 (20% EtOAc in pentane). IR (film)/cm^{-1} 2980, 1726 (C=O), 1700, 1325, 1249, 1140, 1095, 744, 697. ^{1}H NMR (400 MHz, $CDCl_3$) δ 8.16–8.07 (m, 2H, 2 × Ar–H), 7.81–7.73 (m, 1H, Ar–H), 7.68–7.57 (m, 2H, 2 × Ar–H), 1.54 (s, 9H, C(CH_3)$_3$). ^{13}C NMR (101 MHz, $CDCl_3$) δ 152.3 (d, J = 2.8 Hz, C=O), 135.4 (Ar–C), 133.9 (d, J = 21.4 Hz, Ar–C_q), 129.5 (2 × Ar–C), 128.1 (2 × Ar–C), 82.7 (*C*(CH_3)$_3$), 27.9 (C(CH_3)$_3$). ^{19}F NMR (377 MHz, $CDCl_3$) δ 68.54. Analytical data (NMR) in agreement with those reported in the literature.[16]

tert-Butyl (fluoro(4-fluorophenyl)(oxo)-λ^6-sulfaneylidene)carbamate (2d)

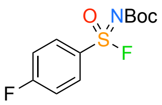

Prepared according to General Procedure C. Selectfluor (0.71 g, 2.0 mmol, 2 equiv) was added to a stirred solution of sulfinamide salt **S4c** (0.28 g, 1.0 mmol, 1 equiv) and potassium acetate (0.20 g, 2.0 mmol, 2.0 equiv) in ethanol (5 mL, 0.2 M) at 0 ºC and slowly warmed to RT over 24 h. The reaction mixture was quenched with water (10 mL) and diluted with CH_2Cl_2 (10 mL). The mixture was extracted with CH_2Cl_2 (3 × 40 mL) and the combined organic layers were dried (Na_2SO_4), filtered and concentrated under reduced pressure. No further purification was required giving sulfonimidoyl fluoride **2d** (0.25 g, 88%) as a colourless viscous oil. IR (film)/cm^{-1} 3109, 2981, 2937, 1700, 1588, 1495, 1371, 1271, 1238, 1141, 1014, 910, 839, 731, 682. ^{1}H NMR (400 MHz, $CDCl_3$) δ 8.21–8.13 (m, 2H, 2 × Ar–H), 7.32 (dd, J = 9.2, 7.9 Hz, 2H, 2 × Ar–H), 1.55 (s, 9H, C(CH_3)$_3$). ^{13}C NMR (101 MHz, $CDCl_3$) δ 166.9 (d, $^1J_{C–F}$ 259.9 Hz, Ar–C_q), 152.6 (C=O), 131.7 (d, $^3J_{C–F}$ = 10.1 Hz, 2 × Ar–C), 131.1 (Ar–C_q), 117.5 (d, $^2J_{C–F}$ = 25.5 Hz, 2 × Ar–C), 83.0 (*C*(CH_3)$_3$), 28.0 (C(CH_3)$_3$).^{19}F NMR (377 MHz, $CDCl_3$) δ 70.1 (S–F), -99.5 (Ar–F).

tert-Butyl (fluoro(4-methoxyphenyl)(oxo)-λ^6-sulfaneylidene)carbamate (2e)

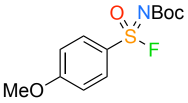

Prepared according to General Procedure C. Selectfluor (0.71 g, 2.0 mmol, 2 equiv) were added to a stirred solution of sulfinamide salt **S4d** (0.29 g, 1.0 mmol, 1 equiv) and potassium acetate (0.20 g, 2.0 mmol, 2.0 equiv) in ethanol (5 mL, 0.2 M) at 0 ºC and slowly warmed to RT over 24 h. The reaction mixture was quenched with water (10 mL) and diluted with CH_2Cl_2 (10 mL). The mixture was extracted with CH_2Cl_2 (3 × 40 mL) and the combined organic layers were dried (Na_2SO_4), filtered and concentrated under reduced pressure. No further purification was required giving sulfonimidoyl fluoride **2e** (0.29 g, quant.) as a colourless viscous oil. IR (film)/cm^{-1} 2982, 2937, 1700, 1595, 1498, 1461, 1320, 1245, 1141, 1096, 1021, 910, 835, 805, 708. ^{1}H NMR (400 MHz, $CDCl_3$) δ 8.04 (d, J = 9.1 Hz, 2H, 2 × Ar–H), 7.04 (d, J = 9.1 Hz, 2H, 2 × Ar–H), 3.91 (s, 3H, OCH_3), 1.52 (s, 9H, C(CH_3)$_3$). ^{13}C NMR (101 MHz, $CDCl_3$) δ

165.3 (Ar–C_q), 152.8 (C=O), 130.8 (2 × Ar–C), 114.9 (2 × Ar–C), 110.1 (Ar–C_q), 82.6 ($C(CH_3)_3$), 56.1 (OCH_3), 28.1 ($C(CH_3)_3$). ^{19}F NMR (377 MHz, $CDCl_3$) δ 69.9.

tert-Butyl (fluoro(oxo)(pyridin-2-yl)-λ^6-sulfaneylidene)carbamate (2f)

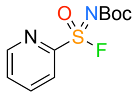

Prepared according to General Procedure A. Selectfluor (527 mg, 1.5 mmol, 1.5 equiv) was added to a stirred solution of sulfinamide salt **S4e** (0.26 g, 1.0 mmol, 1 equiv) in DMF (5 mL, 0.2 M) at 0 ºC and slowly warmed to RT over 24 h. The reaction mixture was quenched with water (10 mL) and diluted with CH_2Cl_2 (10 mL). The mixture was extracted with EtOAc (5 × 15 mL) and the combined organic layers were dried (Na_2SO_4), filtered and concentrated under reduced pressure. Purification by column chromatography (SiO_2, 100% CH_2Cl_2) gave sulfonimidoyl fluoride **2f** (177 mg, 68%) as an amorphous solid. R_f 0.19 (20% EtOAc in pentane). IR (film)/cm^{-1} 3094, 2982, 2937, 1726, 1481, 1580, 1491, 1368, 1334, 1275, 1252, 1144, 1043, 991, 973, 857, 760.^{1}H NMR (400 MHz, $CDCl_3$) δ 8.83 (d, J = 5.5 Hz, 1H, Ar–H), 8.23 (d, J = 8.0 Hz, 1H, Ar–H), 8.03 (t, J = 7.8 Hz, 1H, Ar–H), 7.71–7.65 (m, 1H, Ar–H), 1.52 (s, 9H, $C(CH_3)_3$).^{13}C NMR (101 MHz, $CDCl_3$) δ 150.8 (Ar–C), 138.7 (Ar–C), 129.1 (Ar–C), 124.0 (Ar–C), 83.2 ($C(CH_3)_3$), 27.98 ($C(CH_3)_3$). ^{19}F NMR (377 MHz, $CDCl_3$) δ 58.0.

tert-Butyl (fluoro(isopropyl)(oxo)-λ^6-sulfaneylidene)carbamate (2g)

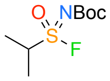

Prepared according to General Procedure C. Selectfluor (0.71 g, 2.0 mmol, 2 equiv) was added to a stirred solution of sulfinamide salt **S4f** (0.26 g, 1.0 mmol, 1 equiv) and potassium acetate (0.20 g, 2.0 mmol, 2.0 equiv) in ethanol (5 mL, 0.2 M) at 0 ºC and slowly warmed to RT over 24 h. The reaction mixture was quenched with water (10 mL) and diluted with CH_2Cl_2 (10 mL). The mixture was extracted with CH_2Cl_2 (5 × 15 mL) and the combined organic layers were dried (Na_2SO_4), filtered and concentrated under reduced pressure. No further purification was required giving sulfonimidoyl fluoride **2g** (0.19 g, 0.86 mmol, 86%) as a white solid.

Alternatively, prepared according to General Procedure A. Selectfluor (0.71 g, 2.0 mmol, 2 equiv) was added to a stirred solution of sulfinamide salt **S4f** (0.26 g, 1.0 mmol, 1 equiv) in DMF (5 mL, 0.2 M) at 0 ºC and slowly warmed to RT over 24 h. The reaction mixture was quenched with water (10 mL) and diluted with CH_2Cl_2 (10 mL). The mixture was extracted with CH_2Cl_2 (5 × 15 mL) and the combined organic layers were dried (Na_2SO_4), filtered and concentrated under reduced pressure. No further purification was required giving sulfonimidoyl fluoride **2g** (0.20 g, 0.90 mmol, 90%) as a white solid.

mp = 62–65 ºC. IR (film)/cm^{-1} 3440, 3388, 3340, 1737, 1670, 1621, 1488, 1454, 1379, 1215, 752. ^{1}H NMR (400 MHz, $CDCl_3$) δ 3.72 (hept, J = 6.8 Hz, 1H, SCH), 1.56 (m, 6H, $CH(CH_3)_2$), 1.50 (s, 9H, $C(CH_3)_3$).^{13}C NMR (101 MHz, $CDCl_3$) δ 153.1 (C=O), 82.6 ($C(CH_3)_3$), 55.4 (d, J = 12.6 Hz, $CH(CH_3)_2$), 28.1 ($C(CH_3)_3$), 16.7 (1 × $CH(CH_3)_2$), 16.5 (1 × $CH(CH_3)_2$). ^{19}F NMR (377 MHz, $CDCl_3$) δ 41.3

tert-Butyl (fluoro(methyl)(oxo)-λ^6-sulfaneylidene)carbamate (2h)

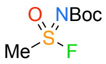

Prepared according to General Procedure A. Selectfluor (1.32 g, 3.74 mmol, 1.5 equiv) was added to a solution of sulfinamide salt **S4g** (500 mg, 2.49 mmol, 1 equiv) in DMF (13 mL, 0.2 M) at 0 °C and warmed to 25 °C for 18 h. H_2O (25 mL) was added and the aqueous mixture extracted with EtOAc (3 ×

25 mL). The combined organic layers were dried ($MgSO_4$), filtered and the solvent removed under reduced pressure to give sulfonimidoyl fluoride **2h** (233 mg, 47%) as a colorless oil. IR (film)/cm^{-1} 2937, 2981, 1692 (C=O), 1319, 1252, 1141, 984, 909, 857, 782. ^{1}H NMR (400 MHz, $CDCl_3$) δ 3.43 (d, $^3J_{H\text{-}F}$ = 4.8 Hz, 3H, SCH_3), 1.50 (s, 9H, $C(CH_3)_3$). ^{13}C NMR (101 MHz, $CDCl_3$) δ 82.9 ($C(CH_3)_3$), 39.6 (d, $^2J_{C\text{-}F}$ = 19.5 Hz, SCH_3) 28.0 ($C(CH_3)_3$). ^{19}F NMR (377 MHz, $CDCl_3$) δ 62.66.

Synthesis of Racemic Sulfonimidamides (3z-aj)

tert-Butyl (oxo(phenyl)(piperidin-1-yl)-λ^6-sulfaneylidene)carbamate (3z)

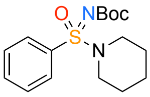

Prepared according to General Procedure D. Piperidine (50 μL, 0.50 mmol, 2.0 equiv) and triethylamine (70 μL, 0.50 mmol, 2.0 equiv) were added to a stirred solution of sulfonimidoyl fluoride **2c** (65 mg, 0.25 mmol, 1.0 equiv) and LiBr (43 mg, 0.50 mmol, 2.0 equiv) in MeCN (0.83 mL, 0.3 M) at RT and heated to 80 °C for 24 h. The solvent was removed under reduced pressure. Purification by flash column chromatography (SiO_2, 1% Et_2O in CH_2Cl_2) afforded sulfonimidamide **3z** (60 mg, 74%) as a white solid. R_f = 0.12 (1% Et_2O in CH_2Cl_2); mp = 95–97 °C. IR (film)/cm^{-1} 2974, 2937, 2855, 1673, 1446, 1364, 1249, 1148, 928. ^{1}H NMR (400 MHz, $CDCl_3$) δ 7.88–7.82 (m, 2H, 2 × Ar–H), 7.62–7.56 (m, 1H, Ar–H), 7.55–7.49 (m, 2H, 2 × Ar–H), 3.11 (td, *J* = 5.0, 3.2 Hz, 4H, 2 × NCH_2), 1.63 (p, *J* = 5.8 Hz, 4H, 2 × NCH_2CH_2), 1.48–1.41 (m, 2H, $NCH_2CH_2CH_2$), 1.39 (s, 9H, $C(CH_3)_3$). ^{13}C NMR (101 MHz, $CDCl_3$) δ 156.5 (C=O), 136.5 (Ar–C_q), 132.7 (Ar–C), 128.9 (2 × Ar–C), 127.6 (2 × Ar–C), 80.1 ($C(CH_3)_3$), 46.6 (2 × NCH_2), 28.0 ($C(CH_3)_3$), 25.2 (2 × NCH_2CH_2), 23.5 ($NCH_2CH_2CH_2$). HRMS (ESI) m/z: Calcd for $C_{16}H_{25}N_2O_3S$ $[M+H]^+$: 325.1586; Found: 325.1596.

tert-Butyl ((4-fluorophenyl)(oxo)(piperidin-1-yl)-λ^6-sulfaneylidene)carbamate (3aa)

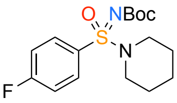

Prepared according to General Procedure D. Piperidine (50 μL, 0.50 mmol, 2.0 equiv) and triethylamine (70 μL, 0.50 mmol, 2.0 equiv) were added to a stirred solution of sulfonimidoyl fluoride **2d** (69 mg, 0.25 mmol, 1 equiv) and LiBr (43 mg, 0.50 mmol, 2 equiv) in MeCN (0.83 mL, 0.3 M) at RT and heated to 80 °C for 24 h. The solvent was removed under reduced pressure. Purification by flash column chromatography (SiO_2, 5% Et_2O in CH_2Cl_2) afforded sulfonimidamide **3aa** (41.5 mg, 48%) as a white solid. mp = 137–138 °C. R_f 0.48 (5% Et_2O in CH_2Cl_2). IR (film)/cm^{-1} 3071, 2975, 2937, 2855, 1674, 1588, 1491, 1365, 1275, 1152, 1096, 932, 865, 821. ^{1}H NMR (400 MHz, $CDCl_3$) δ 7.93–7.84 (m, 2H, 2 × Ar–H), 7.24–7.15 (m, 2H, 2 × Ar–H), 3.12 (m, 4H, 2 × NCH_2), 1.64 (p, *J* = 5.7 Hz, 2H, 2 × NCH_2CH_2), 1.52–1.43 (m, 2H, $NCH_2CH_2CH_2$), 1.42 (s, 9H, $C(CH_3)_3$). ^{13}C NMR (101 MHz, $CDCl_3$) δ 165.3 (d, $^1J_{C-F}$ = 258 Hz, Ar–C_q), 156.6 (C=O), 132.9 (Ar–C_q), 130.6 (d, $^3J_{C-F}$ = 8.9 Hz, 2 × Ar–C), 116.4 (d, $^2J_{C-F}$ = 22.5 Hz, 2 × Ar–C), 80.4 ($C(CH_3)_3$), 46.8 (2 × NCH_2), 28.2 ($C(CH_3)_3$), 25.4 (NCH_2CH_2), 23.7 ($NCH_2CH_2CH_2$). ^{19}F NMR (377 MHz, $CDCl_3$) δ -105.2. HRMS (ESI) m/z Calcd for $C_{16}H_{24}FN_2O_3S$ $[M+H]^+$: 343.1492; Found: 343.1489.

tert-Butyl ((4-methoxyphenyl)(oxo)(piperidin-1-yl)-λ^6-sulfaneylidene)carbamate (3ab)

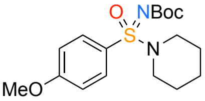

Prepared according to General Procedure D. Piperidine (50 μL, 0.50 mmol, 2.0 equiv) and triethylamine (70 μL, 0.50 mmol, 2.0 equiv) were added to a stirred solution of sulfonimidoyl fluoride **2e** (72 mg, 0.25 mmol, 1 equiv) and flame-dried LiBr (43 mg, 0.50 mmol, 2 equiv) in MeCN (0.83 mL, 0.3 M) at RT and heated to 80 °C for 24 h. The solvent was removed under reduced pressure. Purification by flash column chromatography (SiO_2, 5% Et_2O in CH_2Cl_2) afforded sulfonimidamide **3ab** (77.8 mg, 88%) as a white solid. mp = 115–117 °C. R_f 0.45 (5% Et_2O in CH_2Cl_2). IR (film)/cm^{-1} 2975, 2937, 2851, 1670, 1595, 1495, 1454, 1364, 1245, 1148, 1092, 1047, 924, 835, 805, 723. ^{1}H NMR (400 MHz, $CDCl_3$) δ 7.80 (d, *J* = 8.9 Hz, 2H, 2 × Ar–H), 6.98 (d, *J* = 9.0 Hz, 2H, 2 × Ar–H), 3.87 (s, 3H, OCH_3), 3.09 (m, 4H, 2 × NCH_2), 1.63 (p, *J* = 5.6 Hz, 4H, 2 × NCH_2CH_2), 1.49–1.43 (m, 2H, $NCH_2CH_2CH_2$), 1.42 (s, 9H, $C(CH_3)_3$). ^{13}C NMR (101 MHz, $CDCl_3$) δ 163.2 (Ar–C_q), 156.9 (C=O), 130.1 (2 × Ar–C), 127.8 (Ar–C_q), 114.3 (2 × Ar–C),

80.1 ($C(CH_3)_3$), 55.8 (OCH_3), 46.7 (2 × NCH_2), 28.3 ($C(CH_3)_3$), 25.4 (2 × NCH_2CH_2), 23.8 ($NCH_2CH_2CH_2$). HRMS (ESI) m/z Calcd for $C_{17}H_{27}N_2O_4S$ $[M+H]^+$: 355.1692; Found: 355.1689.

***tert*-Butyl (oxo(piperidin-1-yl)(pyridin-2-yl)-λ⁶-sulfaneylidene)carbamate (3ac)**

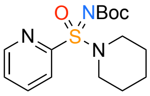

Prepared according to General Procedure D. Piperidine (50 μL, 0.50 mmol, 2.0 equiv) and triethylamine (70 μL, 0.50 mmol, 2.0 equiv) were added to a stirred solution of sulfonimidoyl fluoride **2f** (65 mg, 0.25 mmol, 1 equiv) and flame-dried LiBr (43 mg, 0.50 mmol, 2 equiv) in MeCN (0.83 mL, 0.3 M) at RT and heated to 80 °C for 24 h. The solvent was removed under reduced pressure. Purification by flash column chromatography (SiO_2, 5% Et_2O in CH_2Cl_2) afforded sulfonimidamide **3ac** (69 mg, 84%) as a white solid. R_f 0.14 (2% Et_2O in CH_2Cl_2). mp = 123–124 °C. IR (film)/cm^{-1} 2974, 2940, 2855, 1700, 1364, 1278, 1151, 1051, 939. ^{1}H NMR (400 MHz, $CDCl_3$) δ 8.69 (d, *J* = 4.7 Hz, 1H, Ar–H), 8.08 (d, *J* = 7.9 Hz, 1H, Ar–H), 7.91 (t, *J* = 7.7 Hz, 1H, Ar–H), 7.48 (t, *J* = 7.6 Hz, 1H, Ar–H), 3.38–3.34 (m, 4H, 2 × NCH_2), 1.67–1.62 (m, 4H, 2 × NCH_2CH_2), 1.55–1.46 (m, 2H, $NCH_2CH_2CH_2$), 1.39 (s, 9H, $C(CH_3)_3$). ^{13}C NMR (101 MHz, $CDCl_3$) δ 156.8 (C=O), 156.5 (Ar–C_q), 149.6 (Ar–C), 137.8 (Ar–C), 126.4 (Ar–C), 124.0 (Ar–C), 80.1 ($C(CH_3)_3$), 47.3 (2 × NCH_2), 28.0 ($C(CH_3)_3$), 25.4 (2 × NCH_2CH_2), 23.7 ($NCH_2CH_2CH_2$). HRMS (APCI +p) m/z: Calcd for $C_{15}H_{24}N_3O_3S$ $[M+H]^+$:326.1533; Found: 326.1542.

***tert*-Butyl (isopropyl(oxo)(piperidin-1-yl)-λ⁶-sulfaneylidene)carbamate (3ad)**

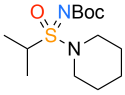

Prepared according to General Procedure D. Piperidine (50 μL, 0.50 mmol, 2.0 equiv) and triethylamine (70 μL, 0.50 mmol, 2.0 equiv) were added to a stirred solution of sulfonimidoyl fluoride **2g** (56 mg, 0.25 mmol, 1 equiv) and flame-dried LiBr (43 mg, 0.50 mmol, 2 equiv) in MeCN (0.83 mL, 0.3 M) at RT and heated to 80 °C for 24 h. The solvent was removed under reduced pressure. Purification by flash column chromatography (SiO_2, 20% EtOAc in pentane) afforded sulfonimidamide **3ad** (23 mg, 32%) as a white solid. R_f 0.25 (1% Et_2O in CH_2Cl_2). mp = 72–74 °C. IR (film)/cm^{-1} 2929, 2855, 1666, 1453, 1278, 1237, 1162, 1043, 939. ^{1}H NMR (400 MHz, $CDCl_3$) δ 3.44–3.22 (m, 5H, $CH(CH_3)_2$ + 2 × NCH_2), 1.68–1.60 (m, 6H, 2 × NCH_2CH_2 and $NCH_2CH_2CH_2$), 1.49 (s, 9H, $C(CH_3)_3$), 1.42 (d, *J* = 6.8 Hz, 3H, 1 × $CH(CH_3)_2$), 1.32 (d, *J* = 6.9 Hz, 3H, 1 × $CH(CH_3)_2$). ^{13}C NMR (101 MHz, $CDCl_3$) δ 157.6 (C=O), 79.7 ($C(CH_3)_3$), 54.5 ($CHCH_3$), 47.4 (2 × NCH_2), 28.2 ($C(CH_3)_3$), 25.8 (2 × NCH_2CH_2), 23.9 ($NCH_2CH_2CH_2$), 16.2 (1 × $CH(CH_3)_2$), 15.7 (1 × $CH(CH_3)_2$). HRMS (ESI) m/z: Calcd for $C_{13}H_{27}N_2O_3S$ $[M+H]^+$: 291.1742; Found: 291.1732.

***tert*-Butyl (methyl(oxo)(piperidin-1-yl)-λ⁶-sulfaneylidene)carbamate (3ae)**

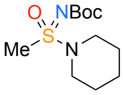

Prepared according to General Procedure B. Piperidine (50 μL, 0.50 mmol, 2.0 equiv) and triethylamine (70 μL, 0.50 mmol, 2.0 equiv) were added to a stirred solution of sulfonimidoyl fluoride **2h** (50 mg, 0.25 mmol, 1 equiv) in THF (0.83 mL, 0.3 M) at RT and warmed to 80 °C for 24 h. The solvent was removed under reduced pressure. Purification by flash column chromatography (SiO_2, 50% EtOAc/pentane) afforded sulfonimidamide **3ae** (53.4 mg, 81%) as a white solid. mp = 63—64 °C. R_f 0.29 (50% EtOAc in pentane). IR (film)/cm^{-1} 2930, 2855, 1662 (C=O), 1595, 1457, 1249, 1279, 1204, 1148, 1066, 981, 924, 820. ^{1}H NMR (400 MHz, $CDCl_3$) δ 3.34–3.18 (m, 4H, 2 × NCH_2), 2.97 (s, 3H, SCH_3), 1.70–1.60 (m, 4H, 2 × NCH_2CH_2), 1.59–1.51 (m, 2H, $NCH_2CH_2CH_2$), 1.45 (s, 9H, $C(CH_3)_3$). ^{13}C NMR (101 MHz, $CDCl_3$) δ 156.9 (C=O), 80.1 ($C(CH_3)_3$), 46.8 (2 × NCH_2), 38.7 (SCH_3), 28.2 ($C(CH_3)_3$), 25.6 (2 × NCH_2CH_2), 23.8 ($NCH_2CH_2CH_2$). HRMS (APCI) m/z Calcd for $C_{11}H_{22}N_2O_3S$ $[M+H]^+$: 263.1424; Found: 263.1421.

tert-Butyl (methyl(morpholino)(oxo)-λ⁶-sulfaneylidene)carbamate (3af)

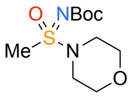

Prepared according to General Procedure B. Morpholine (44 μL, 0.50 mmol, 2.0 equiv) and triethylamine (70 μL, 0.50 mmol, 2.0 equiv) were added to a stirred solution of sulfonimidoyl fluoride **2h** (50 mg, 0.25 mmol, 1 equiv) in THF (0.83 mL, 0.3 M) at RT and warmed to 80 °C for 24 h. The solvent was removed under reduced pressure. Purification by flash column chromatography (SiO_2, 50% EtOAc/pentane) afforded sulfonimidamide **3af** (53.7 mg, 81%) as a white solid. mp = 96–97 °C. R_f 0.31 (50% EtOAc in pentane). IR (film)/cm^{-1} 2863, 2974, 2930, 1666 (C=O), 1453, 1368, 1279, 1245, 1156, 1111, 1069, 939, 865, 790, 723. ^{1}H NMR (400 MHz, $CDCl_3$) δ 3.76 (t, *J* = 4.6 Hz, 4H, 2 × OCH_2), 3.35–3.20 (m, 4H, 2 × NCH_2), 3.01 (s, 3H, SCH_3), 1.45 (s, 9H, $C(CH_3)_3$). ^{13}C NMR (101 MHz, $CDCl_3$) δ 156.4 (C=O), 80.3 (*C*$(CH_3)_3$), 66.2 (2 × OCH_2), 45.9 (2 × NCH_2), 37.9 (SCH_3), 27.9 ($C(CH_3)_3$). HRMS (ESI) m/z Calcd for $C_{10}H_{20}N_2O_4S$ $[M+H]^+$: 265.1230; Found: 265.1222.

tert-Butyl 4-(N-(tert-butoxycarbonyl)-S-methylsulfonimidoyl)piperazine-1-carboxylate (3ag)

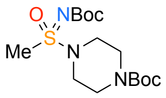

Prepared according to General Procedure B. 1-Boc piperazine (93 mg, 0.50 mmol, 2.0 equiv) and triethylamine (70 μL, 0.50 mmol, 2.0 equiv) were added to a stirred solution of sulfonimidoyl fluoride **2h** (50 mg, 0.25 mmol, 1 equiv) in THF (0.83 mL, 0.3 M) at RT and warmed to 80 °C for 24 h. The solvent was removed under reduced pressure. Purification by flash column chromatography (SiO_2, 60% EtOAc/pentane) afforded sulfonimidamide **3ag** (66.3 mg, 73%) as a white solid. mp = 126–127 °C. R_f 0.19 (60% EtOAc in pentane). IR (film)/cm-1 2978, 2933, 2870, 1696 (C=O), 1457, 1420, 1368, 1282, 1249, 1163, 1125, 691, 931. ^{1}H NMR (400 MHz, $CDCl_3$) δ 3.54 (bs, 4H, 2 × NCH_2), 3.29 (tq, *J* = 12.1, 6.6, 5.8 Hz, 4H, 2 × NCH_2), 3.02 (s, 3H, SCH_3), 1.47 (s, 9H, $C(CH_3)_3$), 1.46 (s, 9H, $C(CH_3)_3$). ^{13}C NMR (101 MHz, $CDCl_3$) δ 156.6 (C=O), 154.3 (C=O), 80.6 (*C*$(CH_3)_3$), 80.5 (*C*$(CH_3)_3$), 45.8 (2 × NCH_2), 38.7 (2 × NCH_2), 28.4 ($C(CH_3)_3$), 28.2 ($C(CH_3)_3$). HRMS (ESI) m/z Calcd for $C_{15}H_{29}N_3O_5SNa$ $[M+Na]^+$: 363.1726; Found: 363.1730.

tert-Butyl (((4-((6-methoxyquinolin-8-yl)amino)pentyl)amino)(methyl)(oxo)-λ⁶-sulfaneylidene)carbamate (3ah)

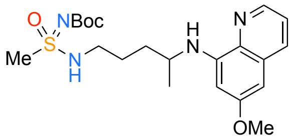

Prepared according to General Procedure B. Primaquine bisphosphate (227 mg, 0.50 mmol, 2.0 equiv) and triethylamine (210 μL, 1.50 mmol, 6.0 equiv) were added to a stirred solution of sulfonimidoyl fluoride **2h** (49 mg, 0.25 mmol, 1 equiv) in THF (0.83 mL, 0.3 M) at RT and warmed to 80 °C for 24 h. The solvent was removed under reduced pressure. Purification by flash column chromatography (SiO_2, 5% Et_2O in CH_2Cl_2) afforded sulfonimidamide **3ah** (49 mg, 58%) as a 1:1 mixture of diastereomers as a colourless oil. R_f 0.14 (10% Et_2O in CH_2Cl_2). IR (film)/cm^{-1} 2970, 2933, 1666, 1617, 1520, 1386, 1282, 1162, 977, 824, 790. ^{1}H NMR (400 MHz, $CDCl_3$) δ 8.53 (dd, *J* = 4.3, 1.7 Hz, 2H, Ar–H_{a+b}), 7.92 (dd, *J* = 8.3, 1.7 Hz, 2H, Ar–H_{a+b}), 7.31 (dd, *J* = 8.2, 4.2 Hz, 2H, Ar–H_{a+b}), 6.34 (d, *J* = 2.5 Hz, 2H, Ar–H_{a+b}), 6.28 (t, *J* = 2.4 Hz, 2H, Ar–H_{a+b}), 5.99 (d, *J* = 8.5 Hz, 2H, NH_{a+b}), 5.70 (s, 2H, NH_{a+b}), 3.89 (s, 6H, $OCH_{3(a+b)}$), 3.68–3.59 (m, 2H, NCH_{a+b}), 3.16 (s, 4H, $NCH_{2(a+b)}$), 3.11 (d, *J* = 1.6 Hz, 6H, $SCH_{3(a+b)}$), 1.79–1.67 (m, 8H, 2 × $CH_{2(a+b)}$), 1.46 (s, 18H, $C(CH_{3(a+b)})_3$), 1.31 (d, *J* = 6.4 Hz, 6H, $CH_{3(a+b)}$). ^{13}C NMR (101 MHz, $CDCl_3$) δ 159.3 $(Ar–C_q)_{a+b}$, 157.7 $(C=O)_{a+b}$, 144.8 $(Ar–C_q)_{a+b}$, 144.3 $(Ar–C)_{a+b}$, 135.2 $(Ar–C_q)_{a+b}$, 134.8 $(Ar–C)_{a+b}$, 129.8 $(Ar–C_q)_{a+b}$, 121.9 $(Ar–C)_{a+b}$, 96.8 $(Ar–C)_{a+b}$, 91.7 $(Ar–C)_{a+b}$, 80.4 $(C(CH_3)_3)_{a+b}$, 55.2 $(OCH_3)_{a+b}$, 47.7 $(NCH_2)_{a/b}$, 47.6 $(NCH_2)_{a/b}$, 42.4 $(NCH)_{a/b}$, 42.3 $(NCH)_{a/b}$, 41.0 $(SCH_3)_{a+b}$,

33.6 $(CH_2)_{a/b}$, 33.5 $(CH_2)_{a/b}$, 28.1 $(C(CH_3)_3)_{a+b}$, 26.5 $(CH_2)_{a/b}$, 26.4 $(CH_2)_{a/b}$, 20.6 $(CHCH_3)_{a+b}$. HRMS (ESI) m/z: Calcd for $C_{21}H_{33}N_4O_4S$ $[M+H]^+$: 437.2223; Found: 437.2225.

tert-Butyl (((3-(10,11-dihydro-5*H*-dibenzo[*b*,*f*]azepin-5-yl)propyl)(methyl)amino)(methyl)(oxo)-λ⁶-sulfaneylidene)carbamate (3ai)

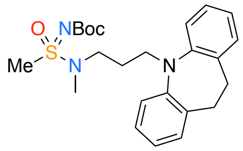

Prepared according to General Procedure B. Desipramine hydrochloride (151 mg, 0.50 mmol, 2.0 equiv) and triethylamine (210 μL, 1.50 mmol, 6.0 equiv) were added to a stirred solution of sulfonimidoyl fluoride **2h** (49 mg, 0.25 mmol, 1 equiv) in THF (0.83 mL, 0.3 M) at RT and warmed to 80 °C for 24 h. The solvent was removed under reduced pressure. Purification by flash column chromatography (SiO_2, 5% Et_2O in CH_2Cl_2) afforded sulfonimidamide **3ai** (79.2 mg, 71%) as a colourless oil. R_f 0.35 (50% EtOAc in pentane). IR (film)/cm^{-1} 3060, 2974, 2930, 1666 (C=O) 1595, 1487, 1274, 1244, 1156, 910, 862, 731. ^1H NMR (400 MHz, $CDCl_3$) δ 7.15–7.09 (m 6H, 6 × Ar–H), 6.96–6.87 (m, 2H, 2 × Ar–H), 3.81 (t, *J* = 6.5 Hz, 2H, NCH_2), 3.36 (dt, *J* = 14.2, 7.2 Hz, 1H, NC*H*H), 3.24 (dt, *J* = 14.1, 7.1 Hz, 1H, NCH*H*), 3.17 (s, 4H, 2 × Ar–CH_2), 2.81 (s, 3H, SCH_3), 2.77 (s, 3H, NCH_3), 1.86 (dtd, *J* = 8.4, 7.4, 6.7, 4.2 Hz, 2H, NCH_2CH_2), 1.46 (s, 9H, $C(CH_3)_3$). ^{13}C NMR (101 MHz, $CDCl_3$) δ 156.8 (C=O), 148.0 (2 × Ar–C_q), 134.4 (2 × Ar–C_q), 130.1 (2 × Ar–C), 126.6 (2 × Ar–C), 122.9 (2 × Ar–C), 119.9 (2 × Ar–C), 80.2 ($C(CH_3)_3$), 47.9 (NCH_2), 47.5 (NCH_2), 39.5 (SCH_3), 34.9 (NCH_3), 32.3 (2 × Ar–CH_2), 28.3 ($C(CH_3)_3$), 26.3 (NCH_2CH_2). HRMS (ESI) m/z Calcd for $C_{24}H_{33}N_3O_3S$ $[M+H]^+$: 444.2330; Found: 444.2321.

tert-Butyl ((4-(2-chlorodibenzo[*b*,*f*][1,4]oxazepin-11-yl)piperazin-1-yl)(methyl)(oxo)-λ⁶-sulfaneylidene)carbamate (3aj)

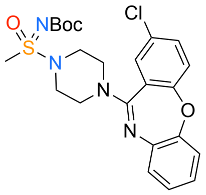

Prepared according to General Procedure B. Amoxipine (157 mg, 0.50 mmol, 2.0 equiv) and triethylamine (70 μL, 1.00 mmol, 2.0 equiv) were added to a stirred solution of sulfonimidoyl fluoride **2h** (49 mg, 0.25 mmol, 1 equiv) in THF (0.83 mL, 0.3 M) at RT and warmed to 80 °C for 24 h. The solvent was removed under reduced pressure. Purification by flash column chromatography (SiO_2, 5% Et_2O in CH_2Cl_2) afforded sulfonimidamide **3aj** (45 mg, 45%) as a colourless oil. R_f 0.26 (5% Et_2O in CH_2Cl_2). IR (film)/cm^{-1} 2978, 2929, 1669, 1602, 1558, 1472, 1282, 1252, 1162, 954. ^1H NMR (400 MHz, $CDCl_3$) δ 7.42 (dd, *J* = 8.7, 2.6 Hz, 1H, Ar–H), 7.31 (d, *J* = 2.6 Hz, 1H, Ar–H), 7.20 (d, *J* = 8.6 Hz, 1H, Ar–H), 7.17–7.08 (m, 3H, 3 × Ar–H), 7.06–7.00 (m, 1H, Ar–H), 3.65 (s, 4H, 2 × NCH_2), 3.45 (s, 4H, 2 × NCH_2), 3.06 (s, 3H, SCH_3), 1.49 (s, 9H, $C(CH_3)_3$)). ^{13}C NMR (101 MHz, $CDCl_3$) δ 159.3 (Ar–C_q), 158.4 (Ar–C_q), 156.5 (C=O), 151.7(Ar–C_q), 139.6 (Ar–C_q), 132.9 (Ar–C), 130.5 (Ar–C_q), 128.7 (Ar–C), 127.1 (Ar–C), 125.8 (Ar–C), 125.1 (Ar–C), 124.6 (Ar–C_q), 122.8 (Ar–C), 120.1 (Ar–C), 80.5 ($C(CH_3)_3$), 47.1 (2 × NCH_2), 45.5 (2 × NCH_2), 38.4 (SCH_3), 28.1 ($C(CH_3)_3$). HRMS (ESI) m/z: Calcd for $C_{23}H_{28}N_4O_4SCl$ $[M+H]^+$: 491.1520; Found: 491.1511.

Experimental Procedures for NBoc-deprotection

(*R*)-1-(4-Methylphenylsulfonimidoyl)piperidine ((*R*)-11)

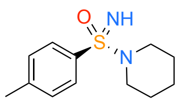

Trifluoroacetic acid (46 µL, 0.6 mmol, 10 equiv) was added to sulfonimidamide **(*R*)-3a** (20 mg, 0.06 mmol, 1 equiv) in CH_2Cl_2 (0.3 mL, 0.2 M) at 0 ºC, and stirred at RT for 4 h. The reaction mixture was quenched with $NaHCO_3$ (5 mL), water (10 mL) was added and then diluted with CH_2Cl_2 (10 mL). The aqueous layer was extracted with CH_2Cl_2 (3 × 20 mL), and the combined organic layers were dried (Na_2SO_4), filtered and concentrated under reduced pressure. Purification by flash column chromatography (SiO_2, 30% EtOAc in pentane) afforded NH-sulfonimidamide **(*R*)-11** (12.5 mg, 89%, >99% *ee*) as a white solid. mp = 83–84 °C. R_f 0.10 (30% EtOAc in pentane). IR (film)/cm^{-1} 3280, 2937, 2851, 1595, 1491, 1454, 1252, 1133, 1072, 1043, 977, 917, 816, 701. ^{1}H NMR (400 MHz, $CDCl_3$) δ 7.75 (d, *J* = 8.4 Hz, 2H, 2 × Ar–H), 7.29 (d, *J* = 8.1 Hz, 2H, 2 × Ar–H), 2.96 (t, *J* = 5.5 Hz, 4H, 2 × NCH_2), 2.42 (s, 3H, Ar–CH_3), 1.66–1.56 (m, 4H, 2 × NCH_2CH_2), 1.41–1.31 (m, 2H, $NCH_2CH_2CH_2$). ^{13}C NMR (101 MHz, $CDCl_3$) δ 142.9 (Ar–C_q), 133.3 (Ar–C_q), 129.4 (2 × Ar–C), 128.2 (2 × Ar–C), 48.1 (2 × NCH_2), 25.8 (2 × NCH_2CH_2), 23.8 ($NCH_2CH_2CH_2$), 21.6 (Ar–CH_3). Characterisation data (NMR) in accordance with literature.[17] $[\alpha]^{23}_D$ = –8 (c 0.5, $CHCl_3$). HPLC Conditions: Chiralpak IA column, 95:5 *n*hexane:*i*PrOH, flow rate: 1 mL min^{-1}, 35 °C, UV detection wavelength: 250 nm. **(*R*)-11** retention time: 44 min.

Synthesis of racemic sample for HPLC analysis prepared according to the above procedure to afford sulfonimidamide **(*rac*)-11** as a white solid with characterisation data in accordance with the above. HPLC Conditions: Chiralpak IA column, 95:5 *n*hexane:*i*PrOH, flow rate: 1 mL min^{-1}, 35 °C, UV detection wavelength: 250 nm, **(*rac*)-11** retention times: 28 & 44 min.

(*R*)-*N'*-Cyclobutyl-4-methylbenzenesulfonimidamide ((*R*)-12)

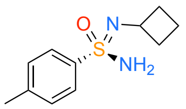

Trifluoroacetic acid (54 µL, 0.8 mmol, 10 equiv) was added to sulfonimidamide **(*R*)-3f** (23 mg, 0.08 mmol, 1 equiv) in CH_2Cl_2 (700 µL, 0.1 M) at 0 ºC, and stirred at RT for 4 h. The reaction mixture was quenched with $NaHCO_3$ (5 mL), water (10 mL) was added and then diluted with CH_2Cl_2 (10 mL). The aqueous layer was extracted with CH_2Cl_2 (3 × 20 mL), and the combined organic layers were dried (Na_2SO_4), filtered and concentrated under reduced pressure. Purification by flash column chromatography (SiO_2, 20% Et_2O in CH_2Cl_2) afforded NH-sulfonimidamide **(*R*)-12** (13 mg, 84%, 98% *ee*) as a colourless oil. R_f 0.19 (20% Et_2O in CH_2Cl_2). IR (film)/cm^{-1} 3254, 2974, 2944, 2870, 1446, 1244, 1133, 1010, 816. ^{1}H NMR (400 MHz, $CDCl_3$) δ 7.86 (d, *J* = 8.4 Hz, 2H, 2 × Ar–H), 7.28 (d, *J* = 8.0 Hz, 2H, 2 × Ar–H), 3.84–3.74 (m, 1H, NCH), 2.42 (s, 3H, $ArCH_3$), 2.15–1.97 (m, 2H, 2 × NCHC*H*H), 1.79–1.51 (m, 4H, 2 × NCHCH*H* + CH_2). ^{13}C NMR (101 MHz, $CDCl_3$) δ 142.8 (Ar–C_q), 139.0 (Ar–C_q), 129.5 (2 × Ar–C), 127.2 (2 × Ar–C), 48.6 (NCH), 31.9 (1 × $NCHCH_2$), 31.8 (1 × $NCHCH_2$), 21.4 (Ar–CH_3), 15.0 ($NCHCH_2CH_2$). HRMS (ESI) m/z: Calcd for $C_{11}H_{17}N_2OS$ $[M+H]^+$: 225.1062; Found: 225.1062. $[\alpha]^{23}_D$ = –46 (c 0.13, $CHCl_3$). HPLC Conditions: Chiralpak IA column, 85:15 *n*hexane:*i*PrOH, flow rate: 1 mL min^{-1}, 35 °C, UV detection wavelength: 250 nm. **(*R*)-12** retention time: 11 min.

Synthesis of racemic sample for HPLC analysis prepared according to the above procedure to afford sulfonimidamide **(*rac*)-12** as a colourless oil with characterisation data in accordance with the above. HPLC

Conditions: Chiralpak IA column, 85:15 *n*hexane:*i*PrOH, flow rate: 1 mL min^{-1}, 35 °C, UV detection wavelength: 250 nm, **(*rac*)-12** retention times: 11 & 16 min.

X-Ray Crystallography Supplementary Data

Manuscript: Synthesis of Highly Enantioenriched Sulfonimidamides by Stereospecific SuFEx Reaction of Sulfonimidoyl Fluorides with Amines

Authors: Stephanie Greed, Edward L. Briggs, Fahima I.M. Idiris, Andrew J.P. White, Ulrich Lücking, and James A. Bull

The X-ray crystal structure of (*R*)-3h

Crystal data for **(*R*)-3h**: $C_{14}H_{22}N_2O_3S$, *M* = 298.39, triclinic, *P*1 (no. 1), *a* = 6.2313(4), *b* = 8.2793(5), *c* = 8.8044(6) Å, α = 117.174(7), β = 100.944(5), γ = 93.245(5)°, *V* = 391.37(5) Å^3, *Z* = 1, D_c = 1.266 g cm^{-3}, μ(Cu-Kα) = 1.917 mm^{-1}, *T* = 173 K, colourless blocks, Agilent Xcalibur PX Ultra A diffractometer; 2737 independent measured reflections (R_{int} = 0.0246), F^2 refinement,[X1,X2] R_1(obs) = 0.0300, wR_2(all) = 0.0798, 2681 independent observed absorption-corrected reflections [$|F_o| > 4\sigma(|F_o|)$, completeness to θ_{full}(67.7°) = 99.9%], 188 parameters. The absolute structure of **(*R*)-3h** was unambiguously determined by use of the Flack parameter [*x* = –0.035(17)]. CCDC 1991431.

References

[X1] SHELXTL v5.1, Bruker AXS, Madison, WI, 1998.

[X2] SHELX-2013, G.M. Sheldrick, *Acta Cryst.*, 2015, **C71**, 3-8.

Figures

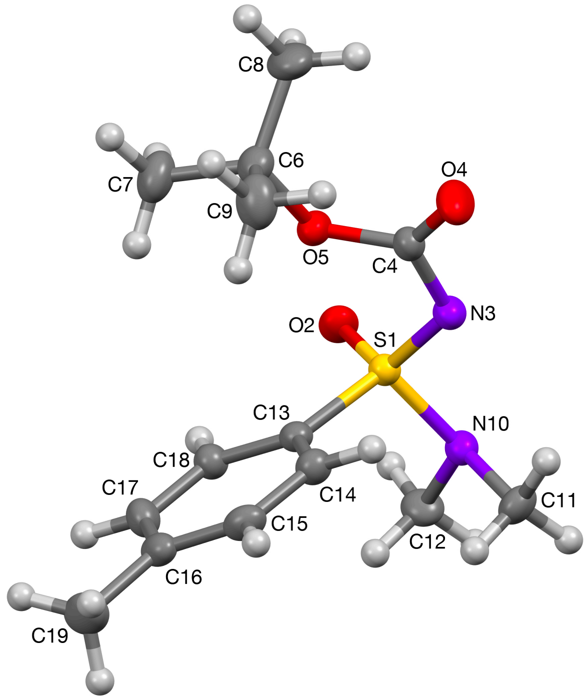

Figure S 2: The crystal structure of (R)-3h (50% probability ellipsoids).

References

[1] C. Worch, I. Atodiresei, G. Raabe, C. Bolm, *Chem. Eur. J.* **2010**, *16*, 677–683.

[2] M. Steurer, C. Bolm, *J. Org. Chem.* **2010**, *75*, 3301–3310.

[3] C. B. W. Phippen, J. K. Beattie, C. S. P. McErlean, *Chem. Commun.* **2010**, *46*, 8234–8236.

[4] C. Drago, E.-J. Walker, L. Caggiano, R. F. W. Jackson, *Org. Synth.* **2009**, *86*, 121–129.

[5] C. Drago, L. Caggiano, R. F. W. Jackson, *Angew. Chem. Int. Ed.* **2005**, *44*, 7221–7223.

[6] M. Zenzola, R. Doran, R. Luisi, J. A. Bull, *J. Org. Chem.* **2015**, *80*, 6391–6399.

[7] N. Pemberton, H. Graden, E. Evertsson, E. Bratt, M. Lepistö, P. Johannesson, P. H. Svensson, *ACS Med. Chem. Lett.* **2012**, *3*, 574–578.

[8] C. H. Rosa, M. L. B. Peixoto, G. R. Rosa, B. Godoi, F. Z. Galetto, M. G. M. D'Oca, M. Godoi, *Tetrahedron Lett.* **2017**, *58*, 3777–3781.

[9] J. Li, X. Bi, H. Wang, J. Xiao, *Phosphorus, Sulfur Silicon Relat. Elem.* **2014**, *189*, 1873–1881.

[10] W. Guo, G. Lv, J. Chen, W. Gao, J. Ding, H. Wu, *Tetrahedron* **2010**, *66*, 2297–2300.

[11] F. Rajabi, S. Razavi, R. Luque, *Green Chem.* **2010**, *12*, 786–789.

[12] L. Zong, X. Ban, C. W. Kee, C.-H. Tan, *Angew. Chem. Int. Ed.* **2014**, *53*, 11849–11853.

[13] N. P. Crouch, R. M. Adlington, J. E. Baldwin, M.-H. Lee, C. H. MacKinnon, *Tetrahedron* **1997**, *53*, 6993–7010.

[14] H. Cui, W. Wei, D. Yang, Y. Zhang, H. Zhao, L. Wang, H. Wang, *Green Chem.* **2017**, *19*, 3520–3524.

[15] H. L. Yue, M. Klussmann, *Synlett* **2016**, *27*, 2505–2509.

[16] H. Mukherjee, J. Debreczeni, J. Breed, S. Tentarelli, B. Aquila, J. E. Dowling, A. Whitty, N. P. Grimster, *Org. Biomol. Chem.* **2017**, *15*, 9685–9695.

[17] F. Izzo, M. Schäfer, R. Stockman, U. Lücking, *Chem. Eur. J.* **2017**, *23*, 15189–15193.

^{1}H and ^{13}C-NMR Spectra

tert-Butyl (p-tolylsulfinyl)carbamate ((S)-5)

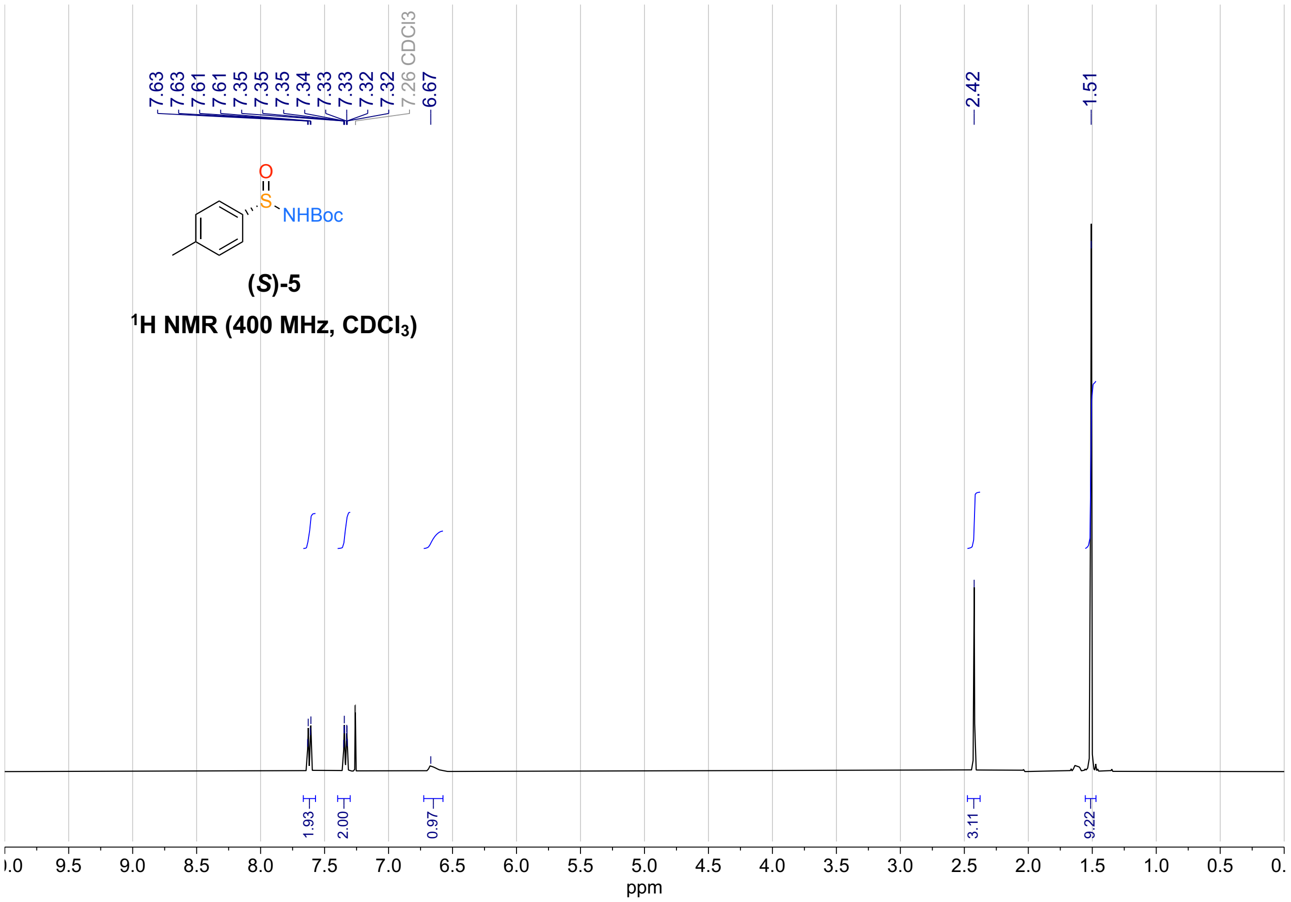

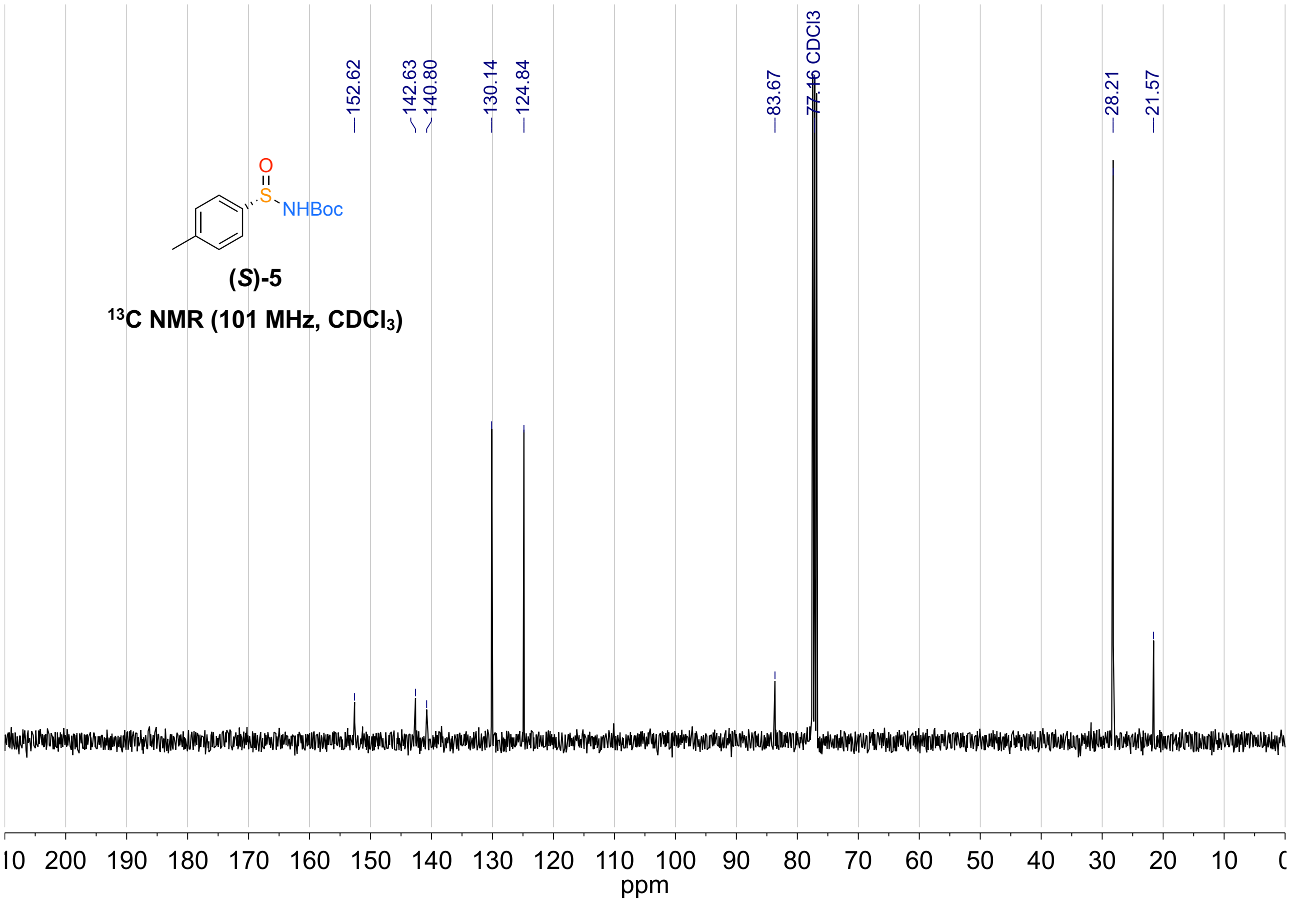

Sodium (*tert*-butoxycarbonyl)(*p*-tolylsulfinyl)amide ((*S*)-1a)

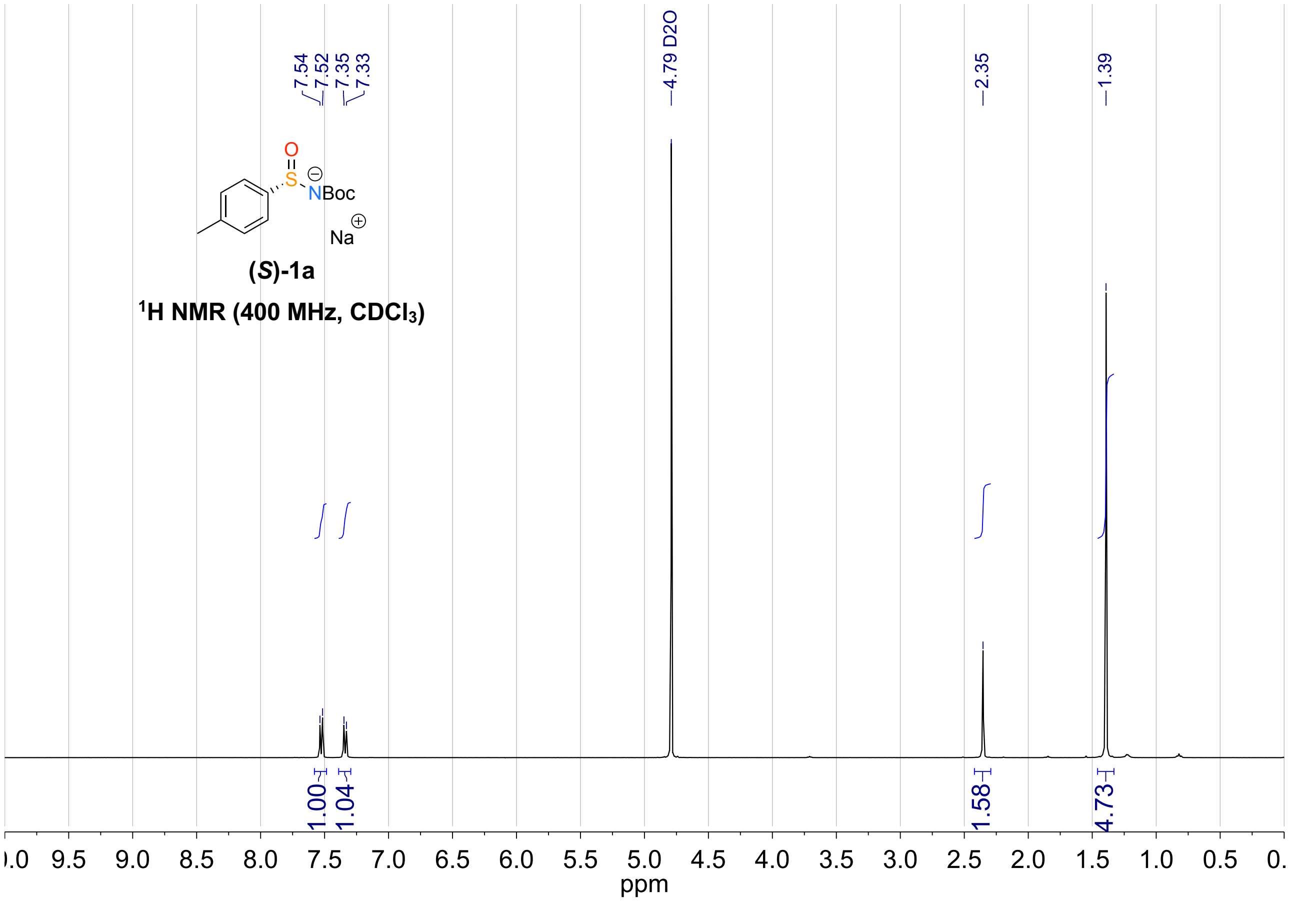

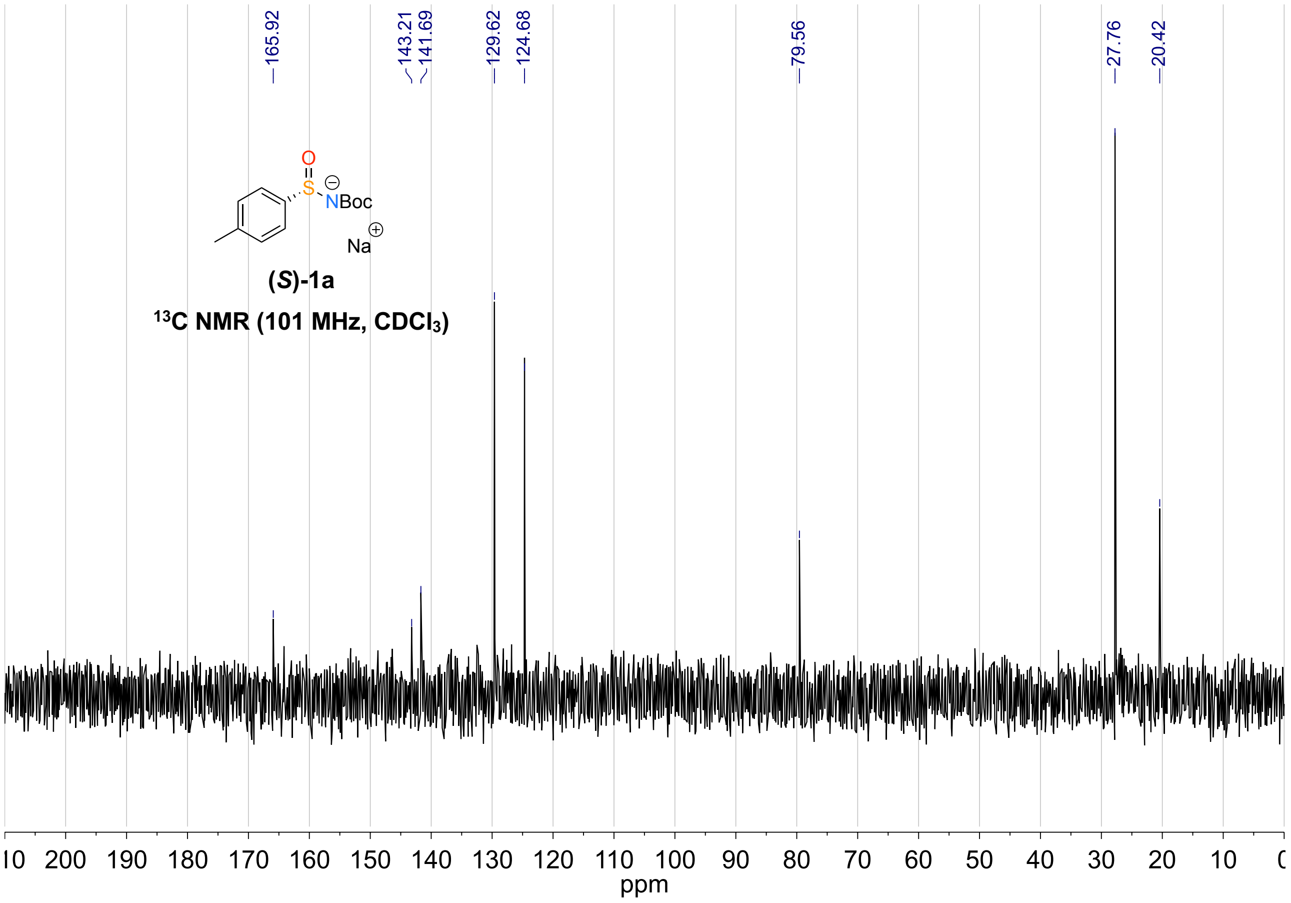

tert-Butyl (fluoro(oxo)(p-tolyl)-λ⁶-sulfaneylidene)carbamate ((R)-2a)

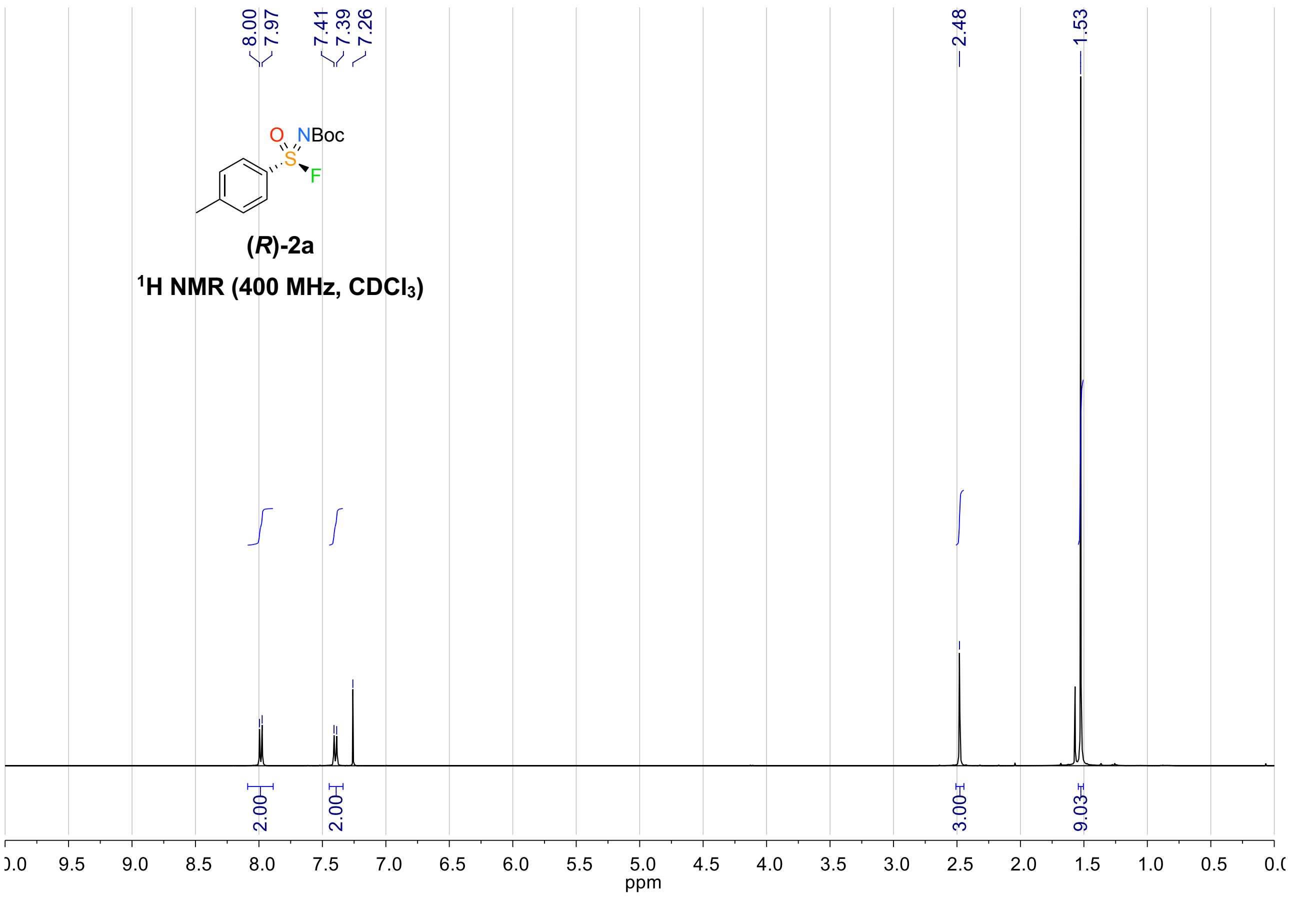

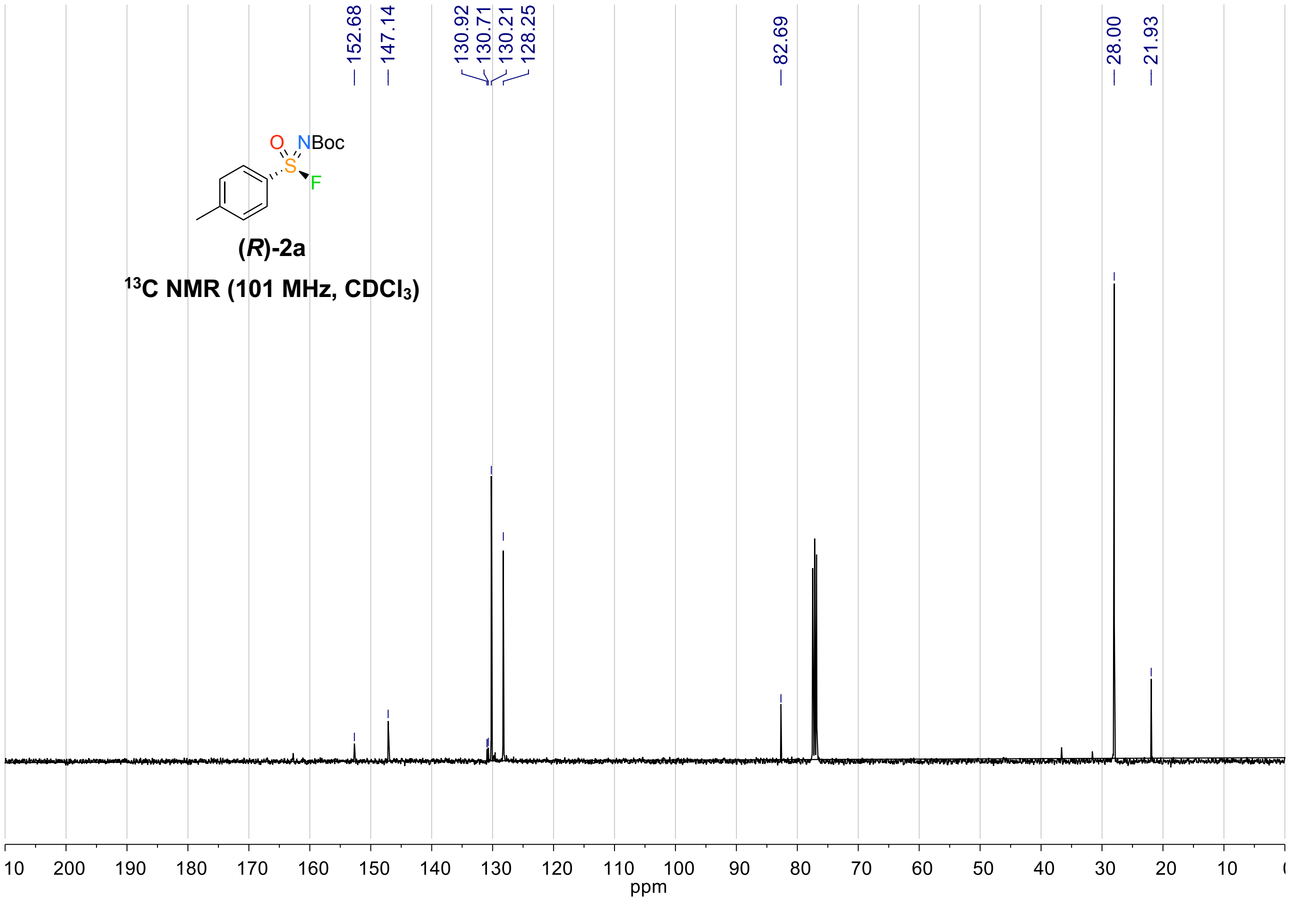

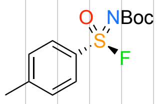

(*R*)-2a

^{19}F NMR (377 MHz, $CDCl_3$)

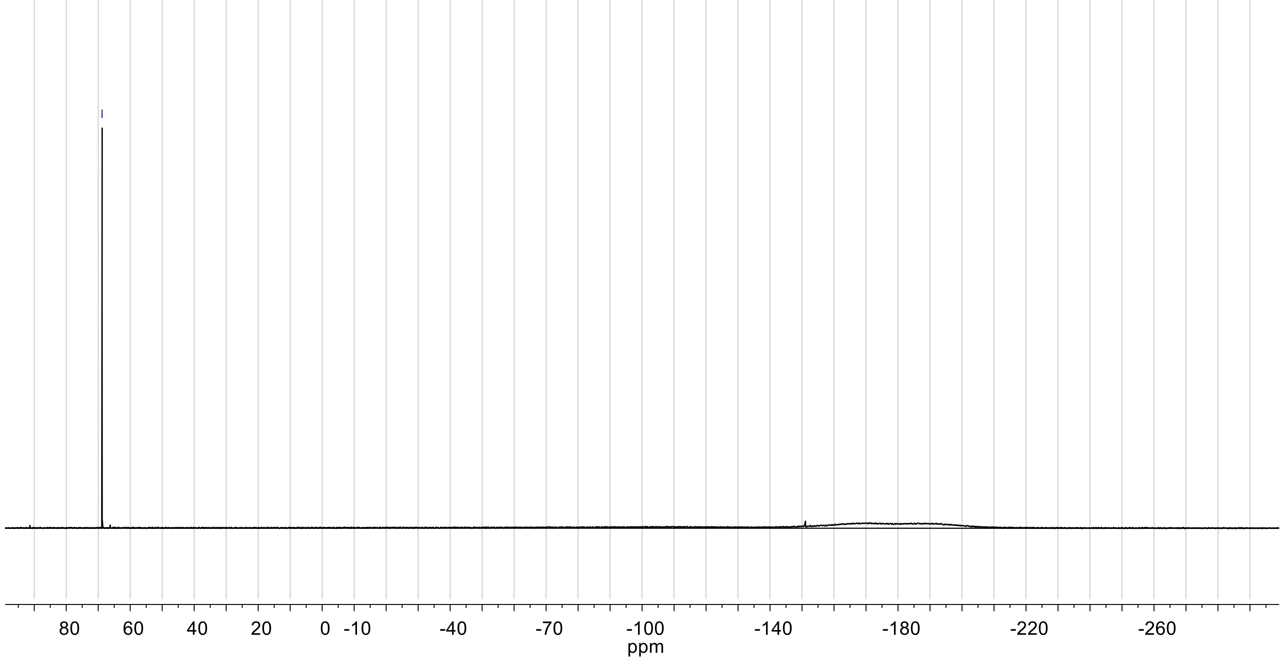

tert-Butyl (oxo(piperidin-1-yl)(p-tolyl)-λ⁶-sulfaneylidene)carbamate ((R)-3a)

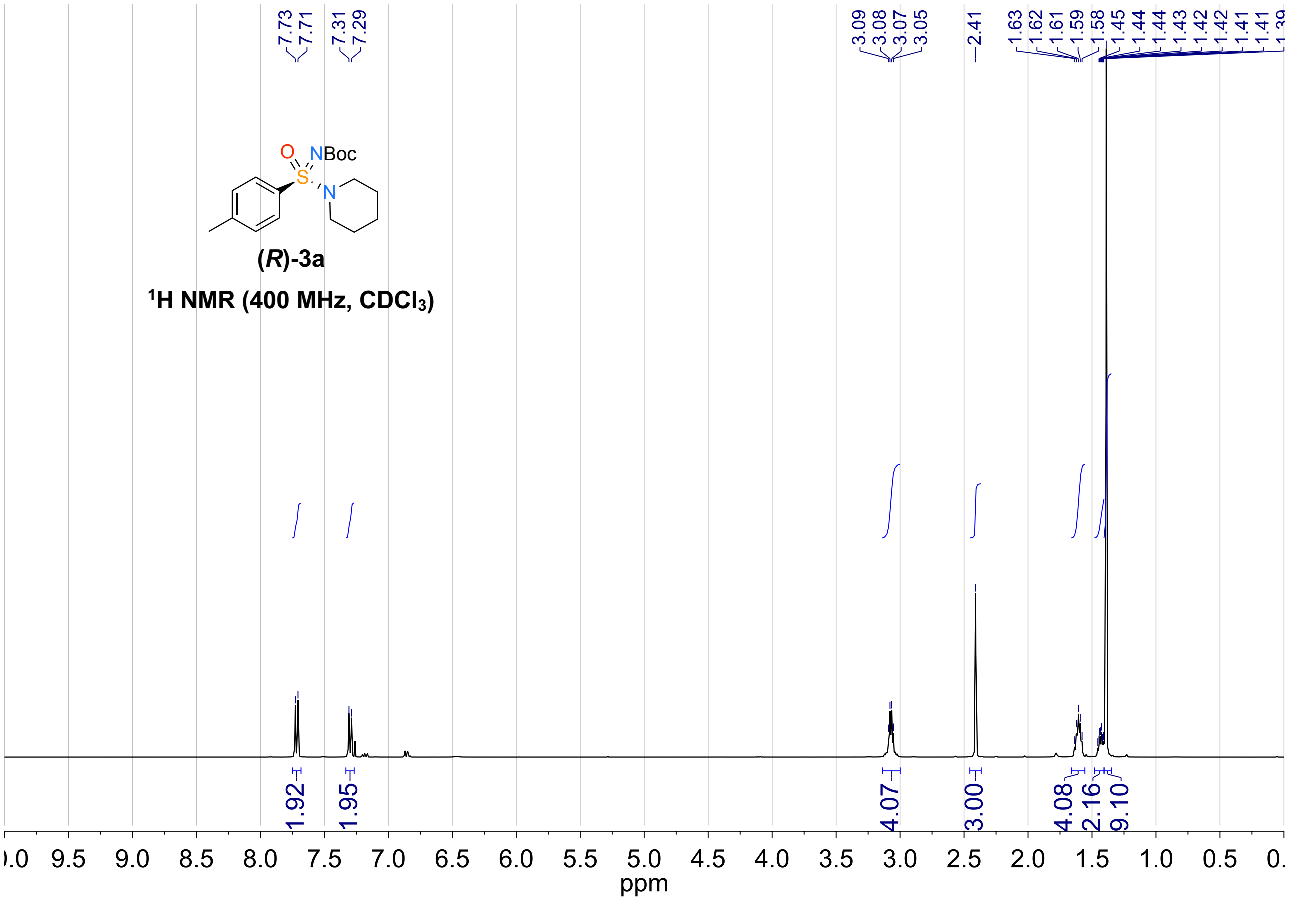

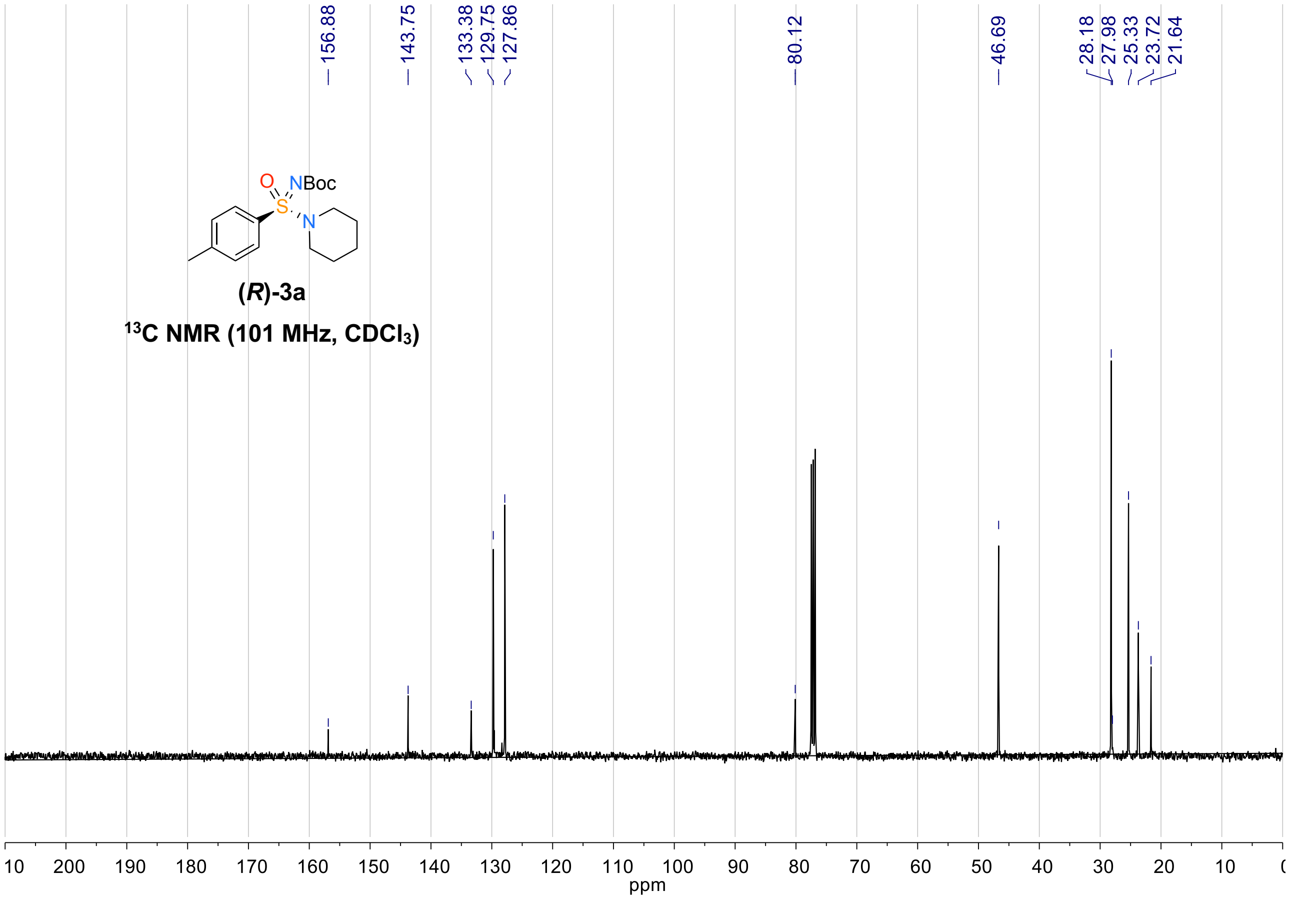

tert-Butyl (R)-((butylamino)(oxo)(p-tolyl)-λ⁶-sulfaneylidene)carbamate ((R)-3b)

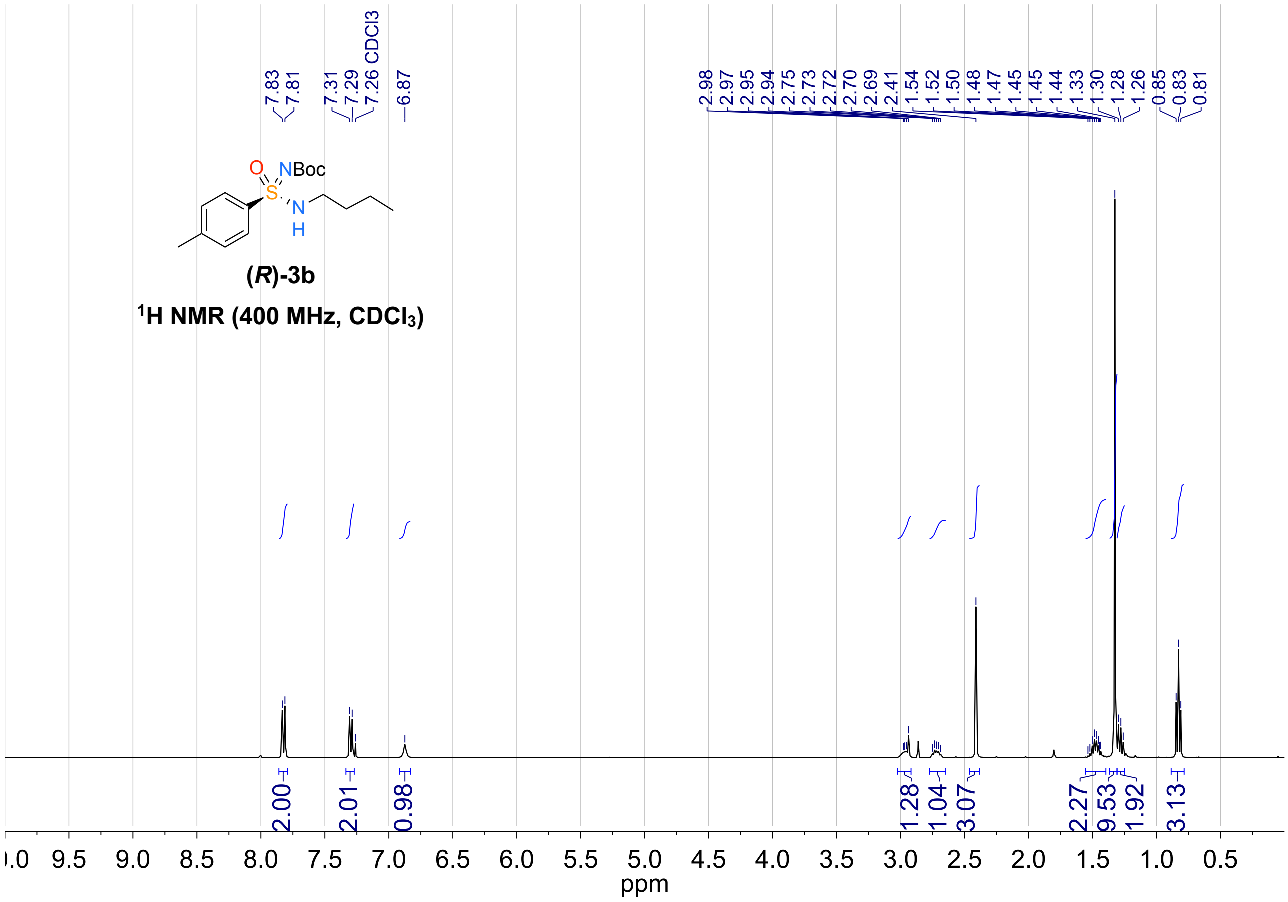

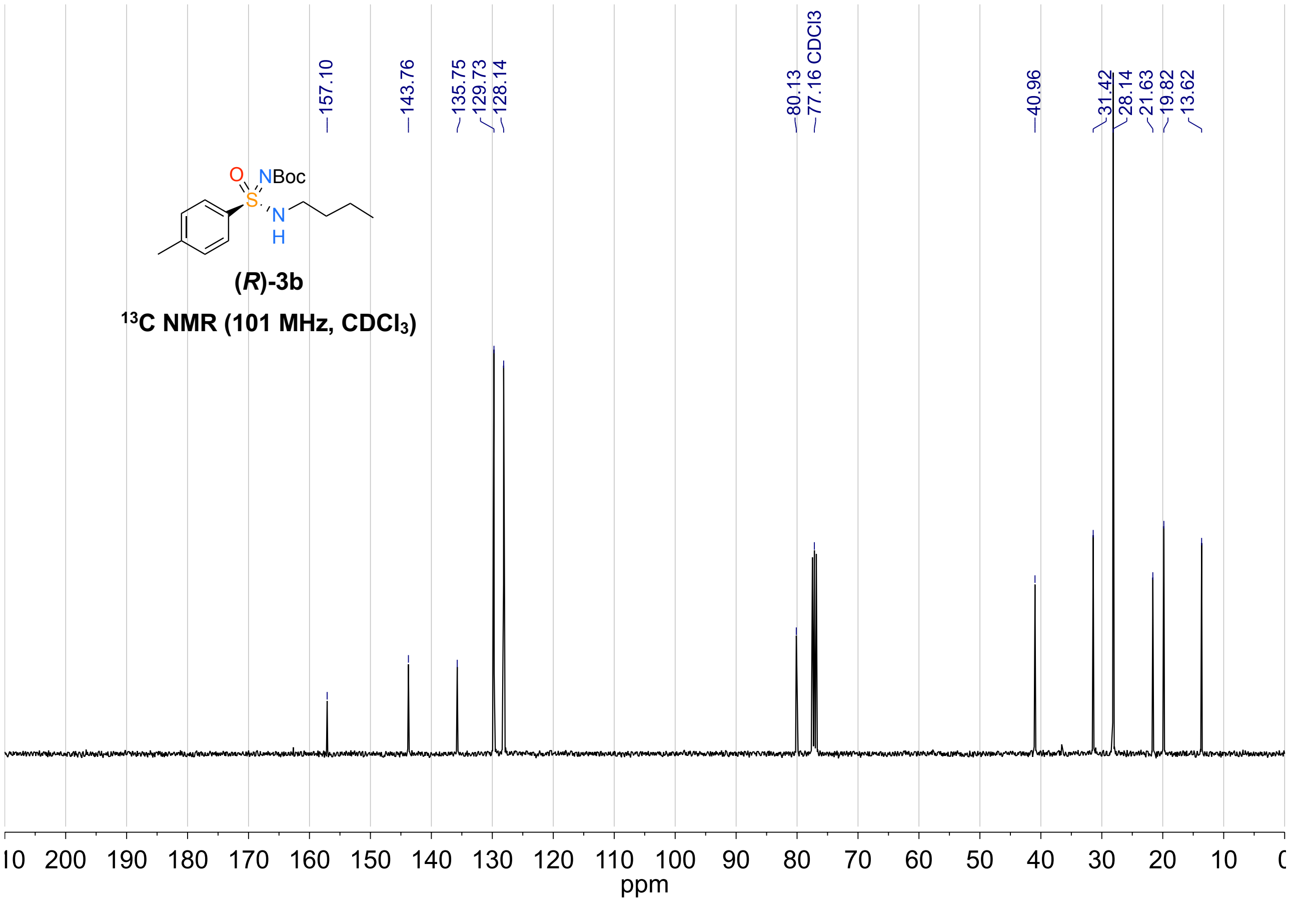

tert-Butyl (_R_)-((benzylamino)(oxo)(_p_-tolyl)-λ⁶-sulfaneylidene)carbamate ((_R_)-3c)

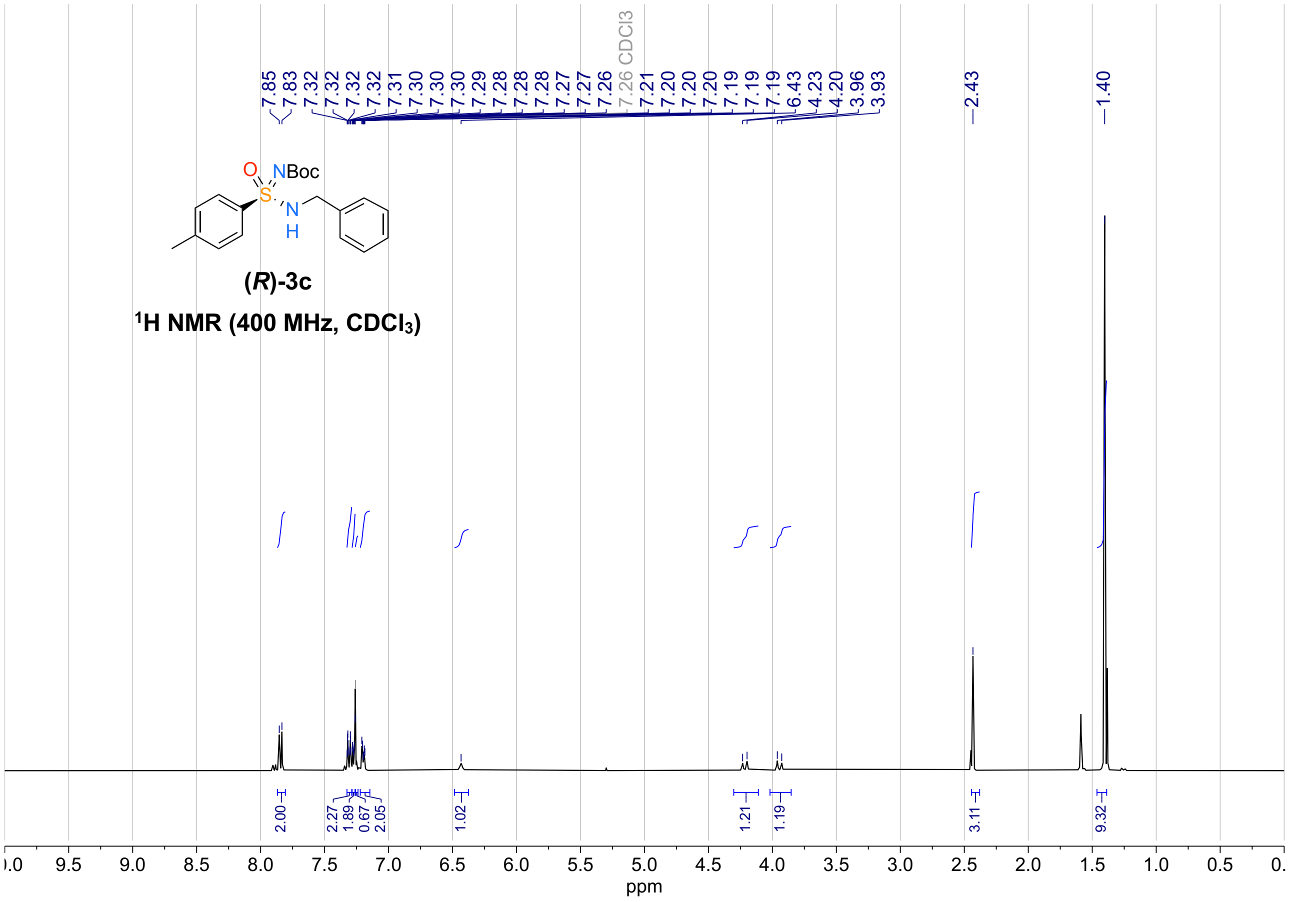

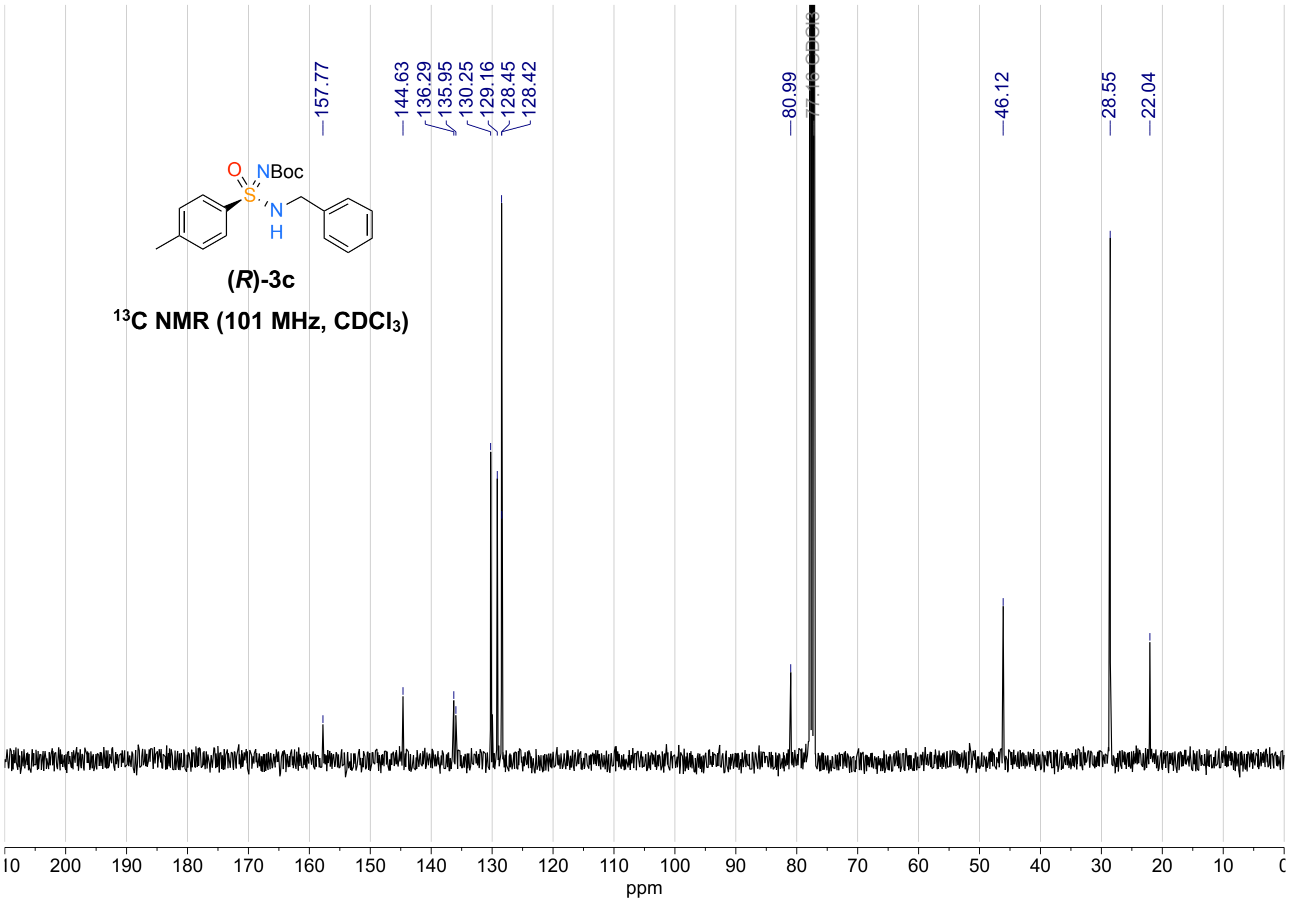

tert-Butyl (R)-((allylamino)(oxo)(p-tolyl)-λ^6-sulfaneylidene)carbamate ((R)-3d)

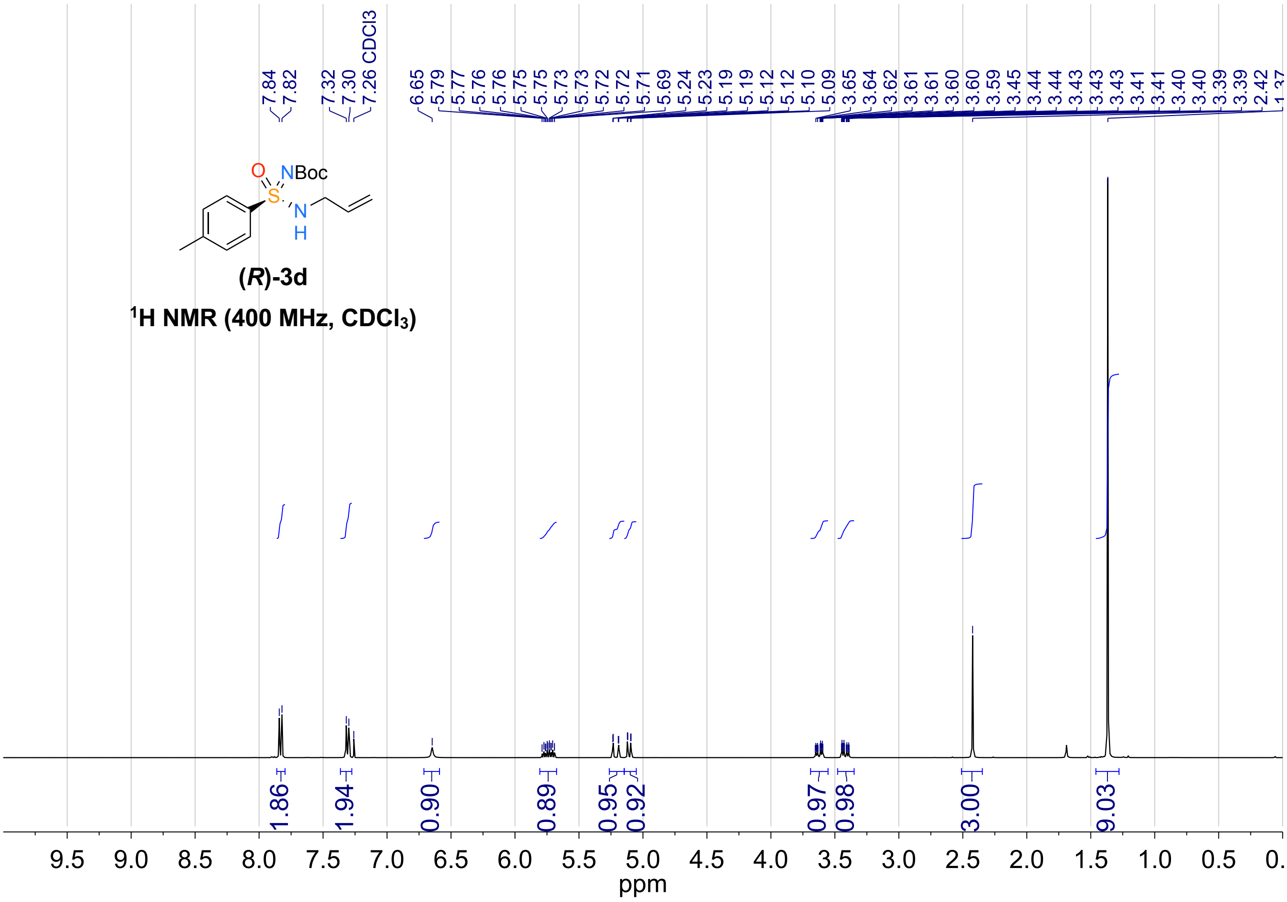

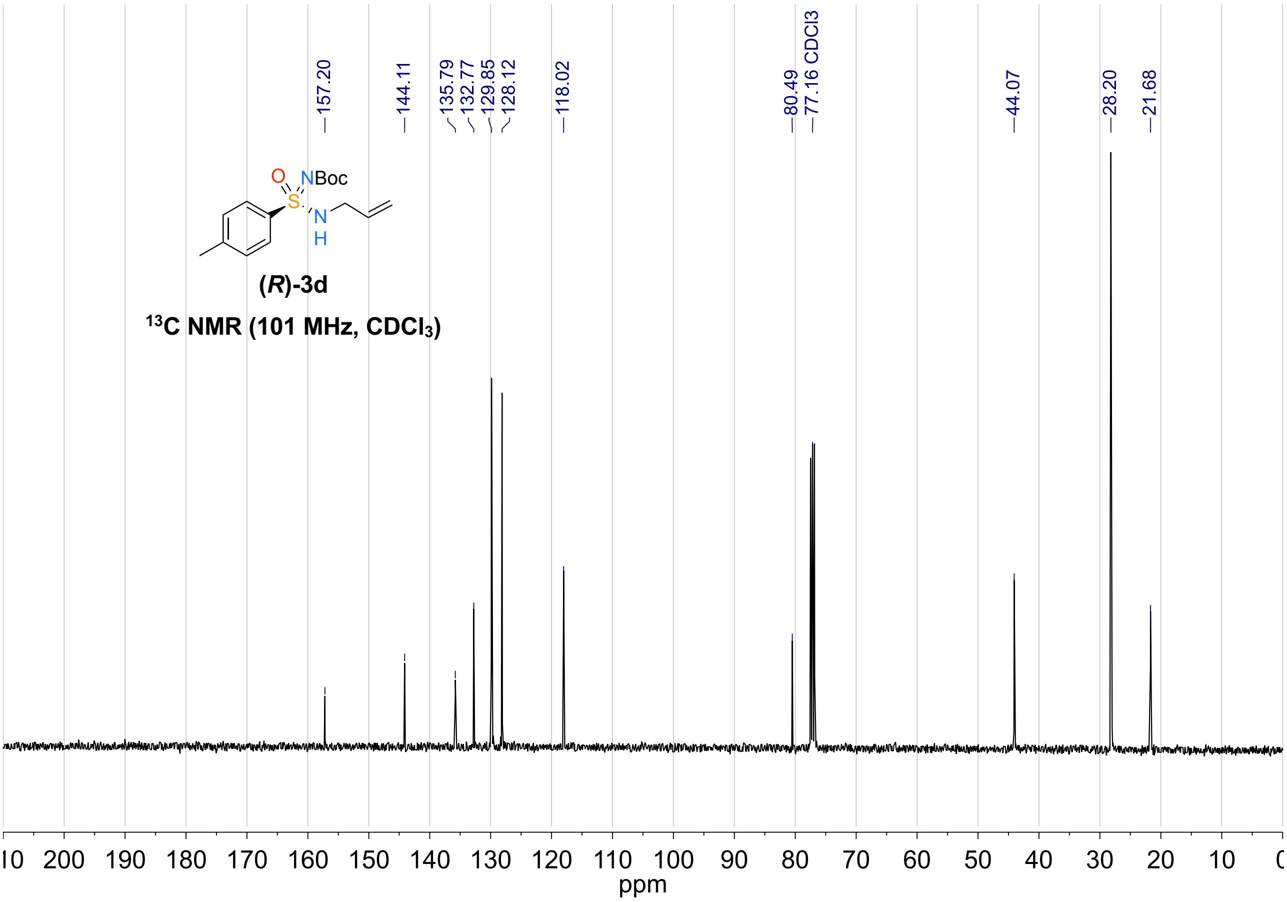

tert-Butyl (R)-(((cyclopropylmethyl)amino)(oxo)(p-tolyl)-λ⁶-sulfaneylidene)carbamate ((R)-3e)

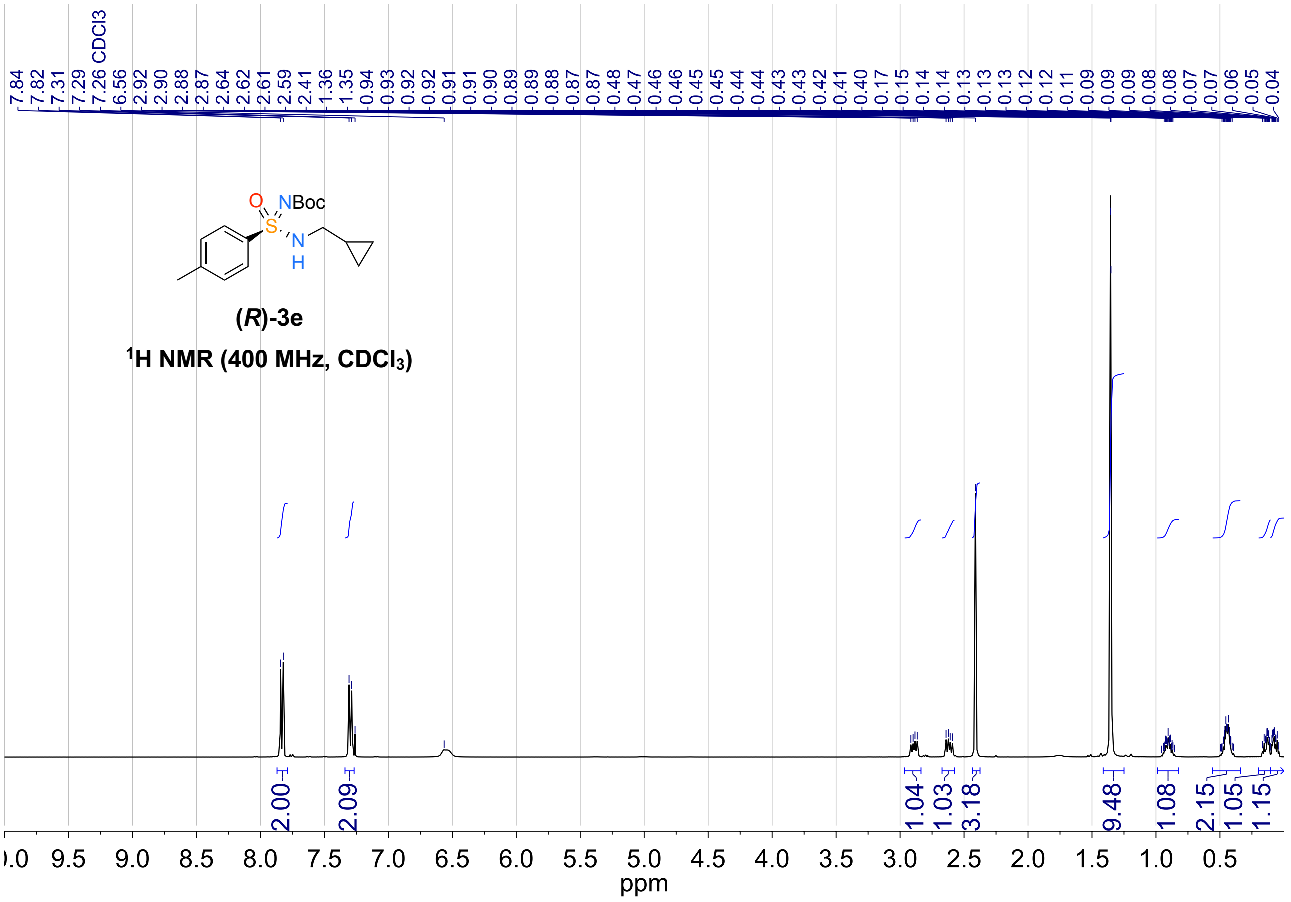

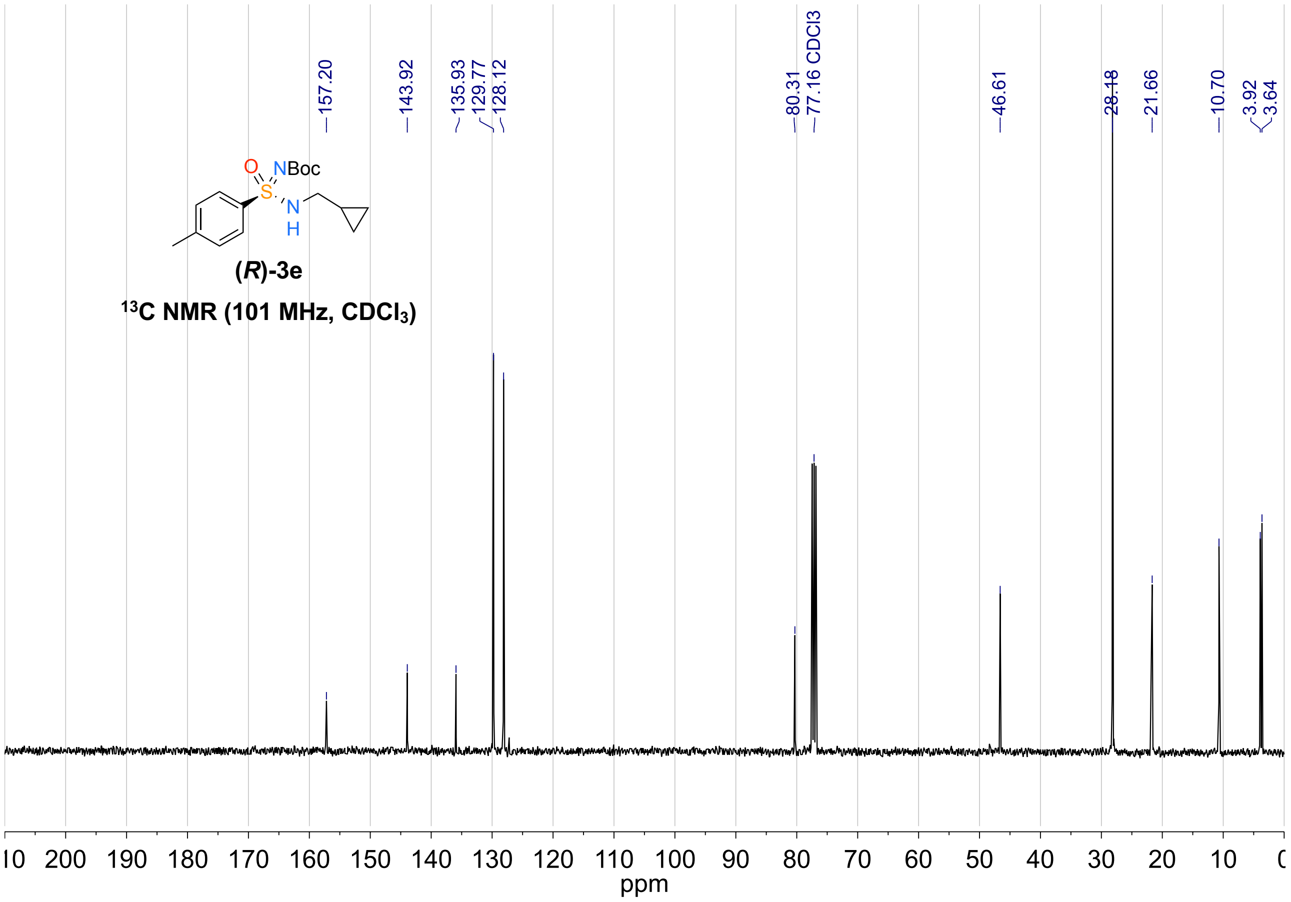

tert-Butyl (R)-((cyclobutylamino)(oxo)(p-tolyl)-λ⁶-sulfaneylidene)carbamate ((R)-3f)

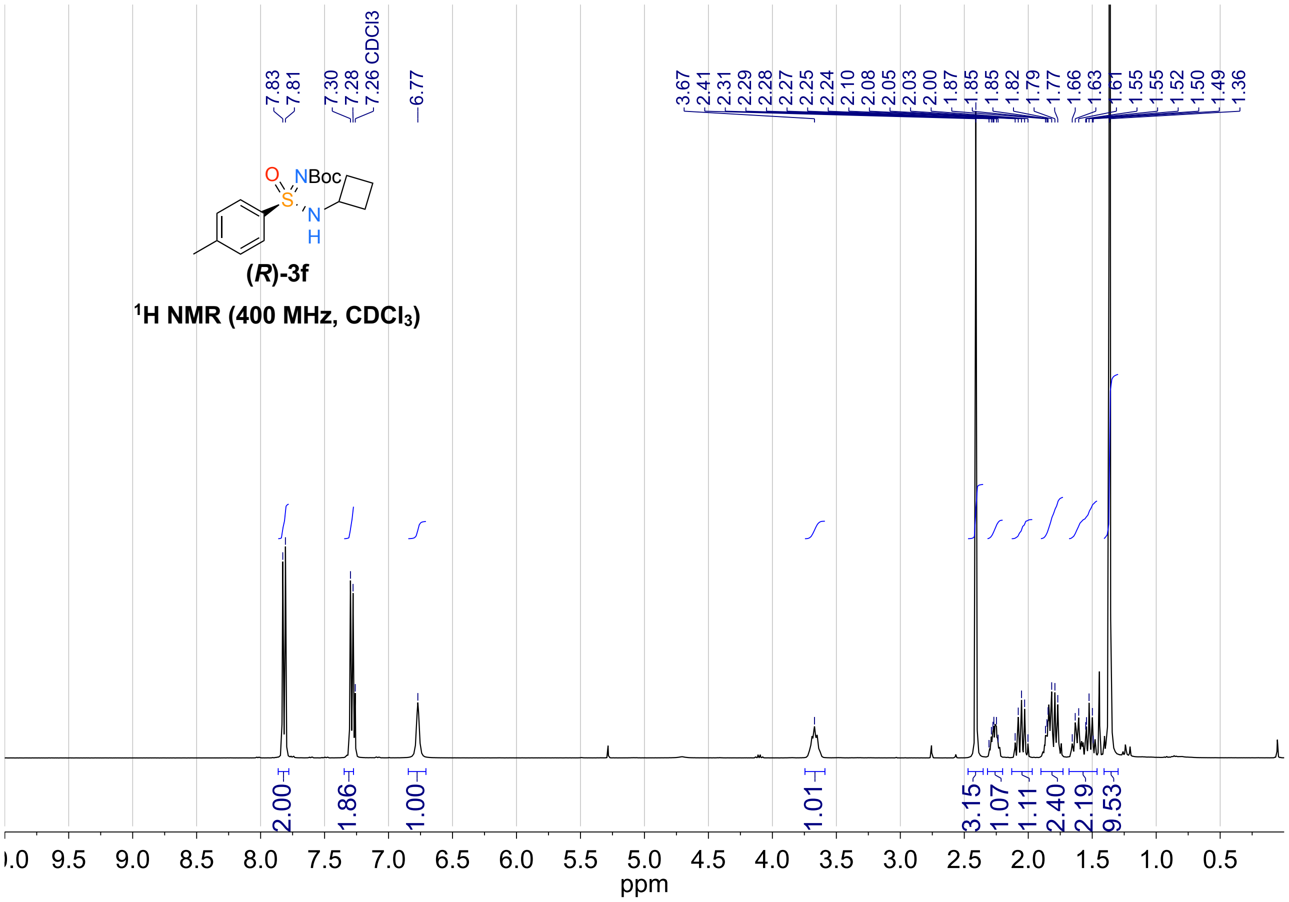

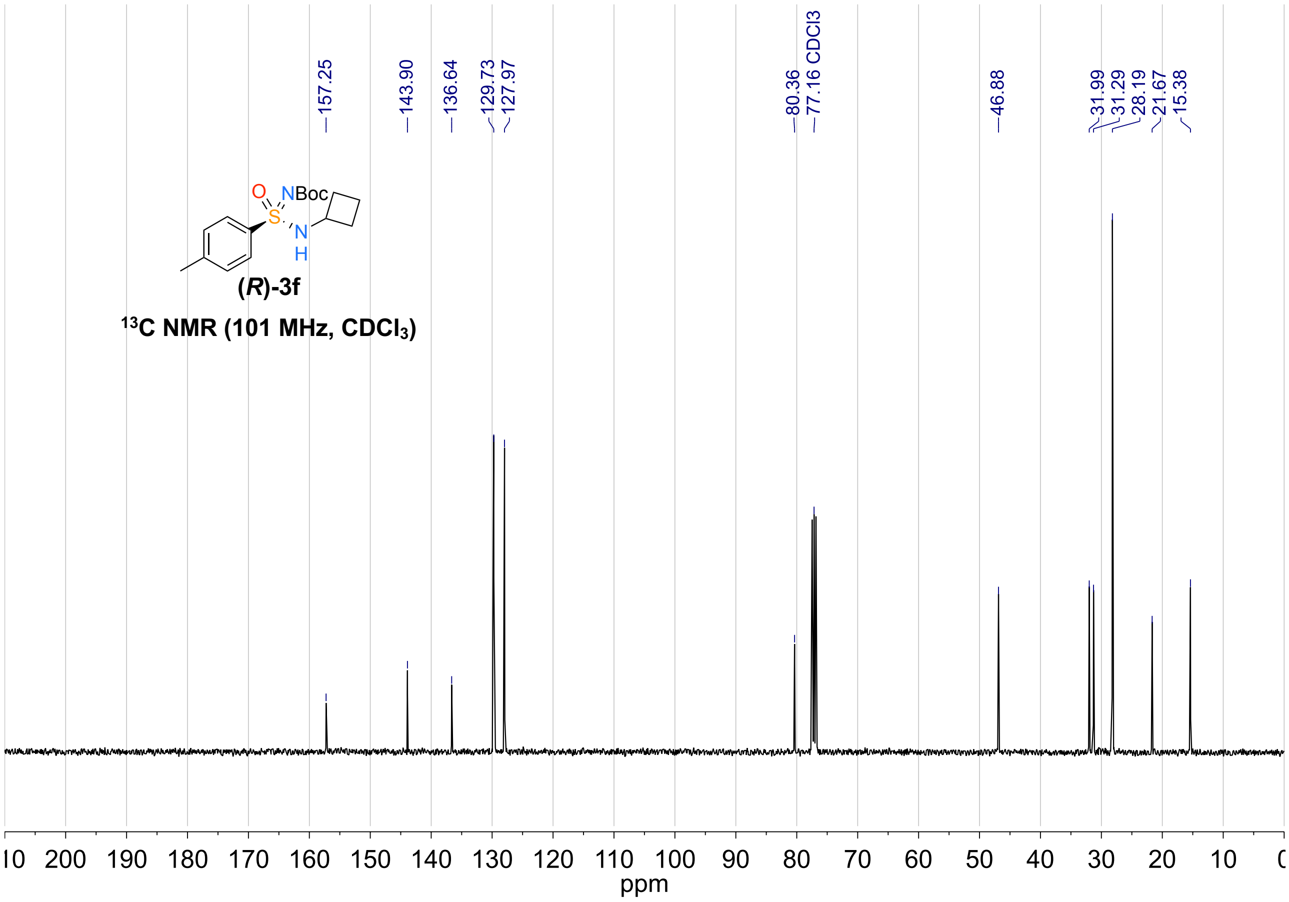

tert-Butyl (R)-((cyclohexylamino)(oxo)(p-tolyl)-λ6-sulfaneylidene)carbamate ((R)-3g)

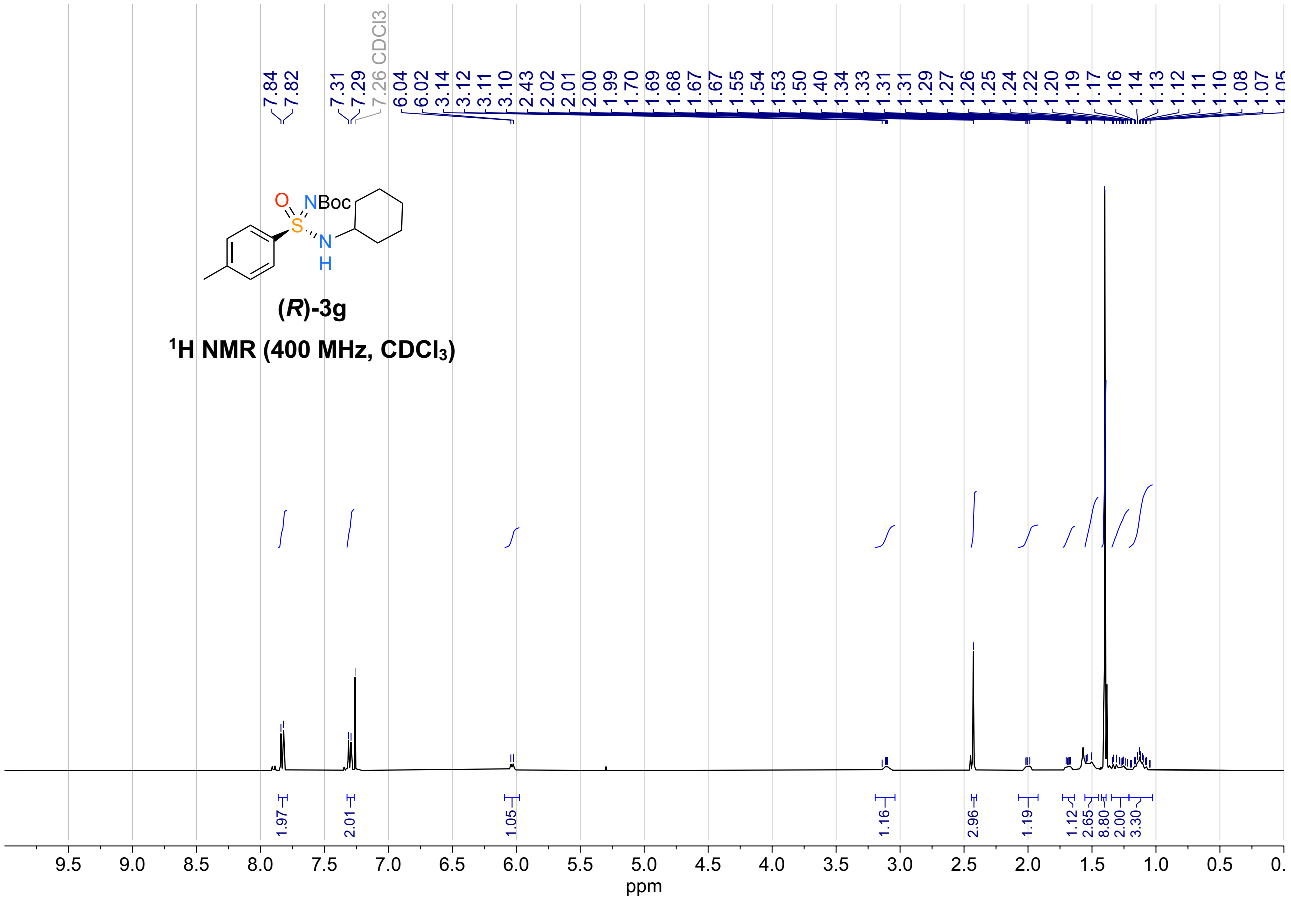

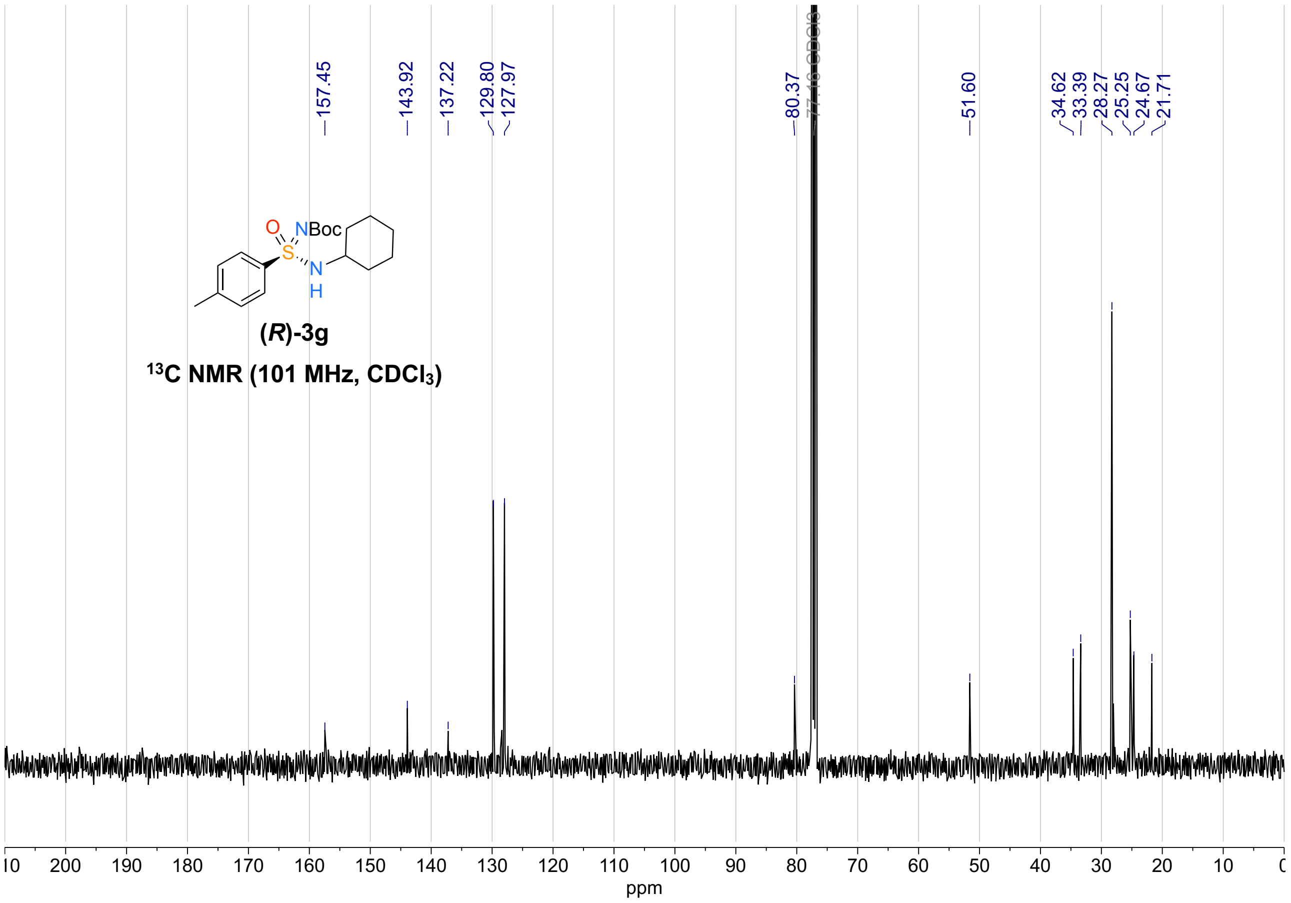

tert-Butyl (R)-((dimethylamino)(oxo)(p-tolyl)-λ^6-sulfaneylidene)carbamate ((R)-3h)

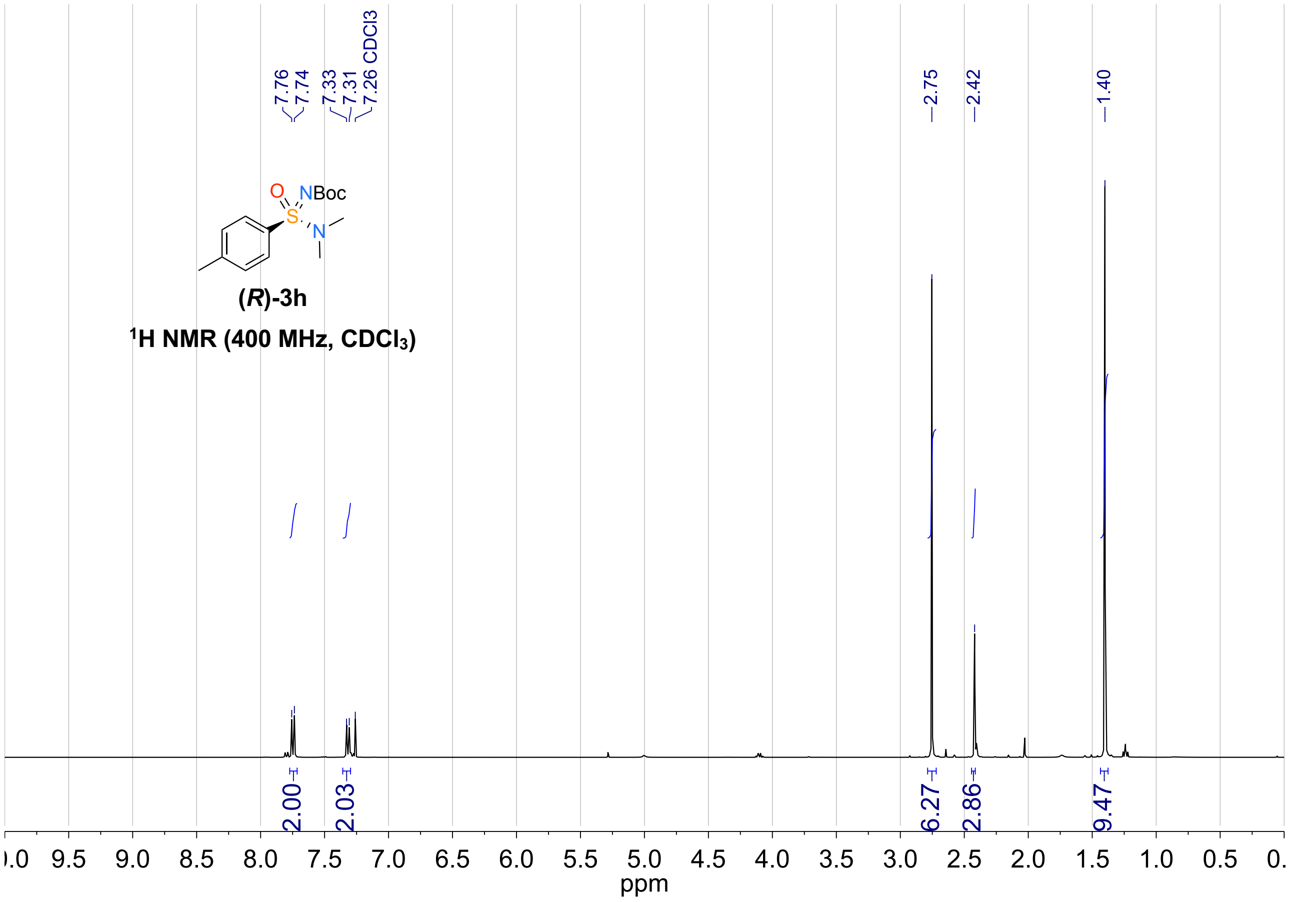

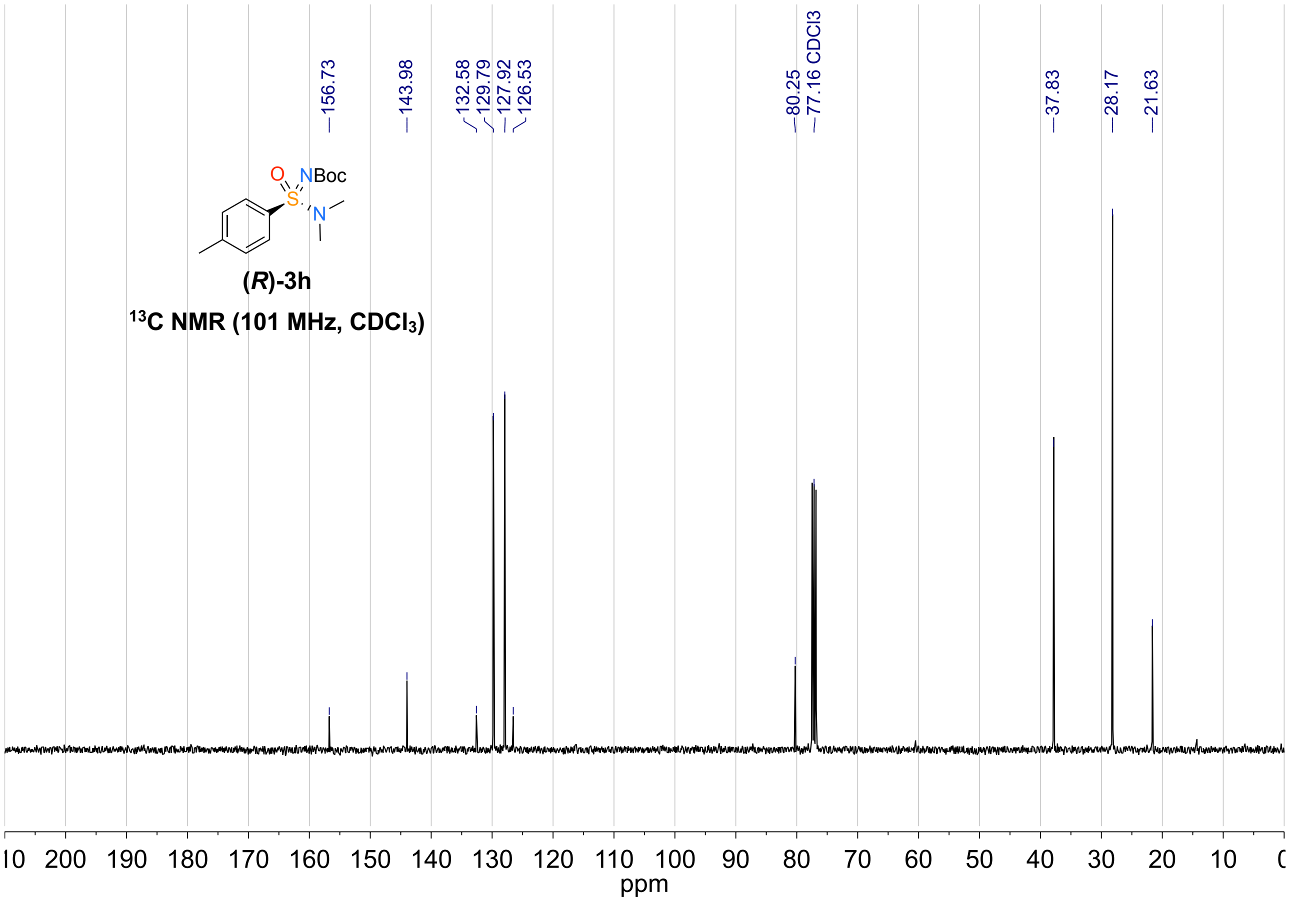

tert-Butyl (_R_)-((benzyl(methyl)amino)(oxo)(_p_-tolyl)-λ⁶-sulfaneylidene)carbamate ((_R_)-3i)

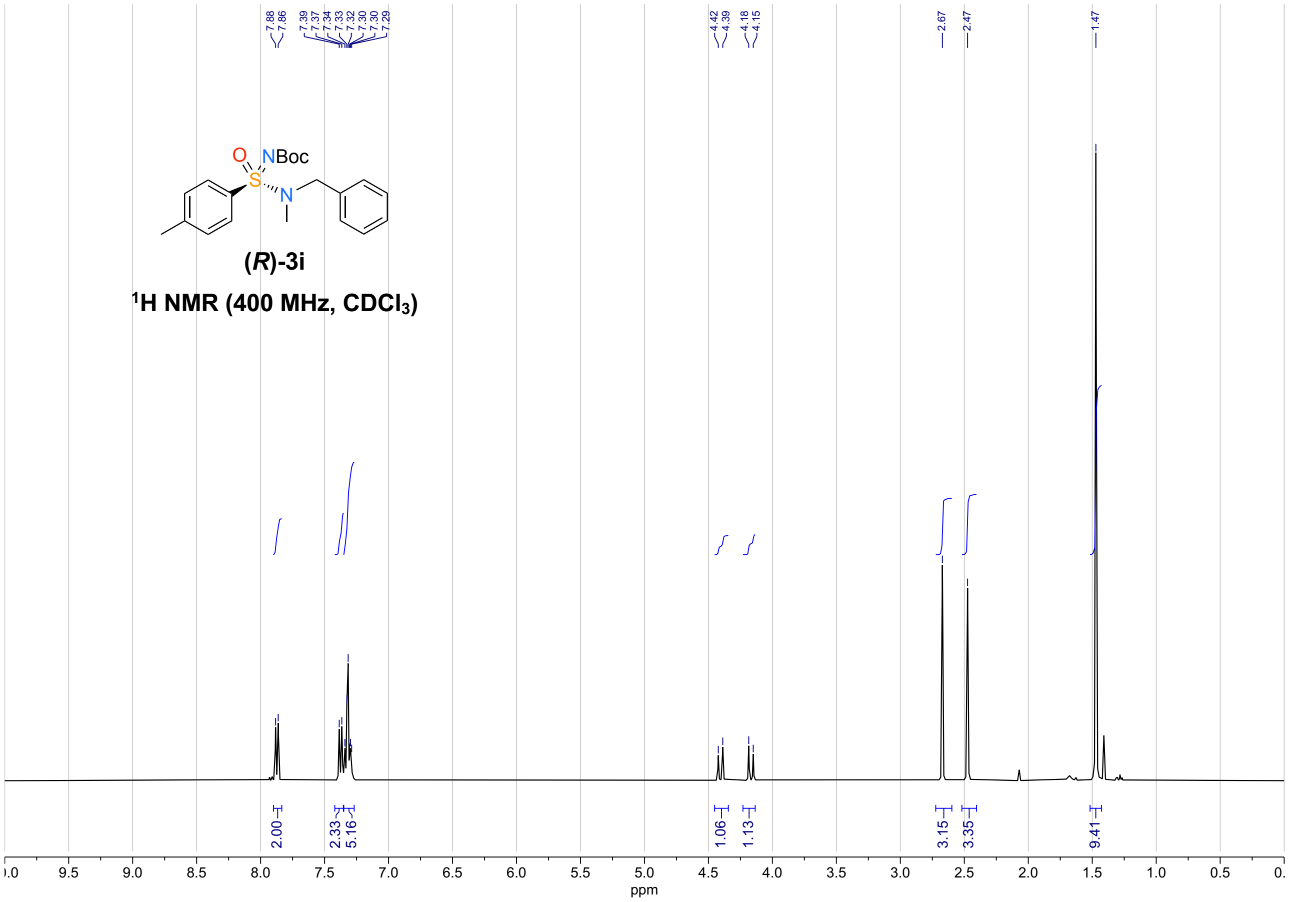

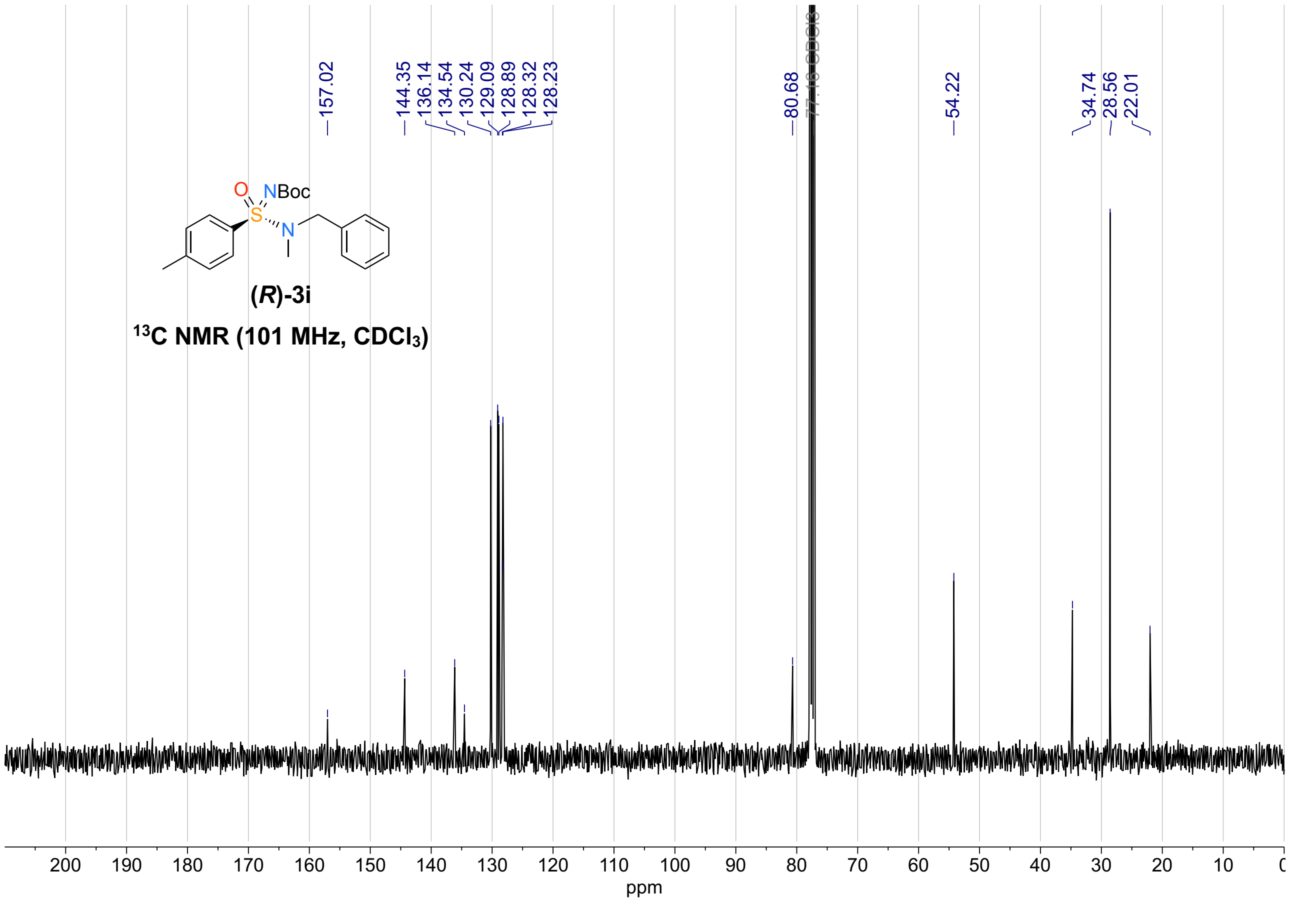

tert-Butyl (_R_)-((3,4-dihydroisoquinolin-2(1_H_)-yl)(oxo)(_p_-tolyl)-λ^6-sulfaneylidene)carbamate ((_R_)-3j)

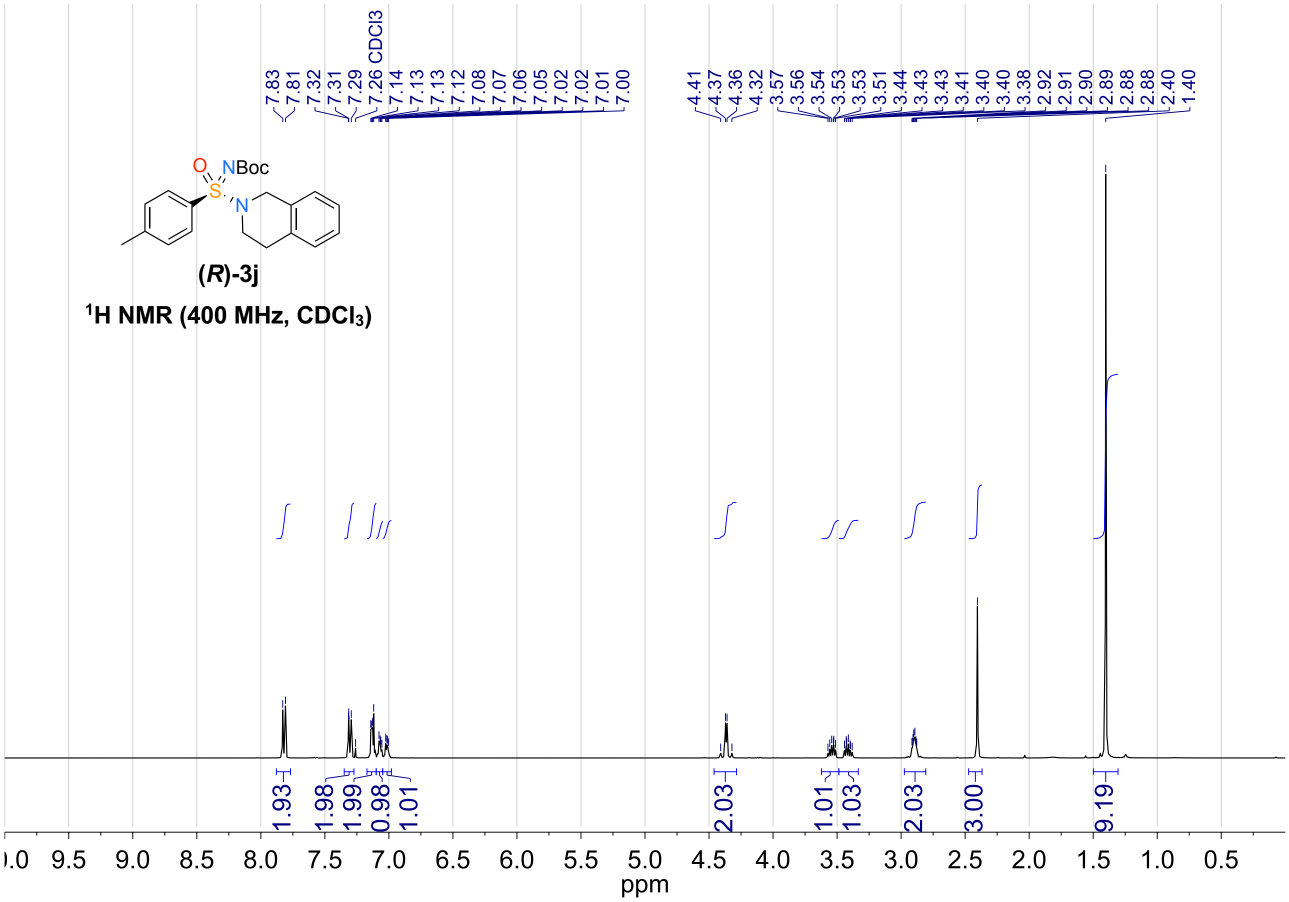

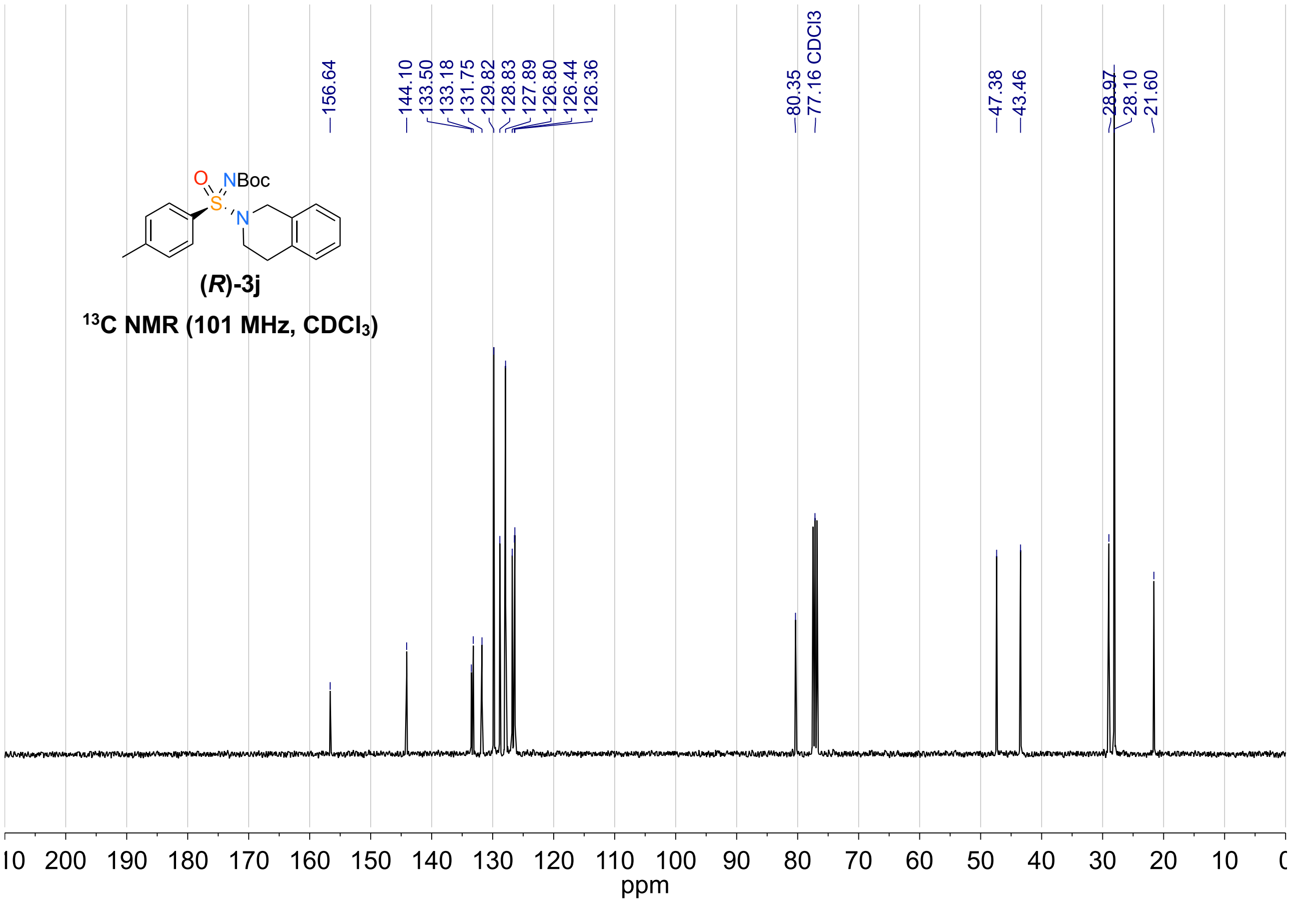

tert-Butyl (R)-(oxo(pyrrolidin-1-yl)(p-tolyl)-λ⁶-sulfaneylidene)carbamate ((R)-3k)

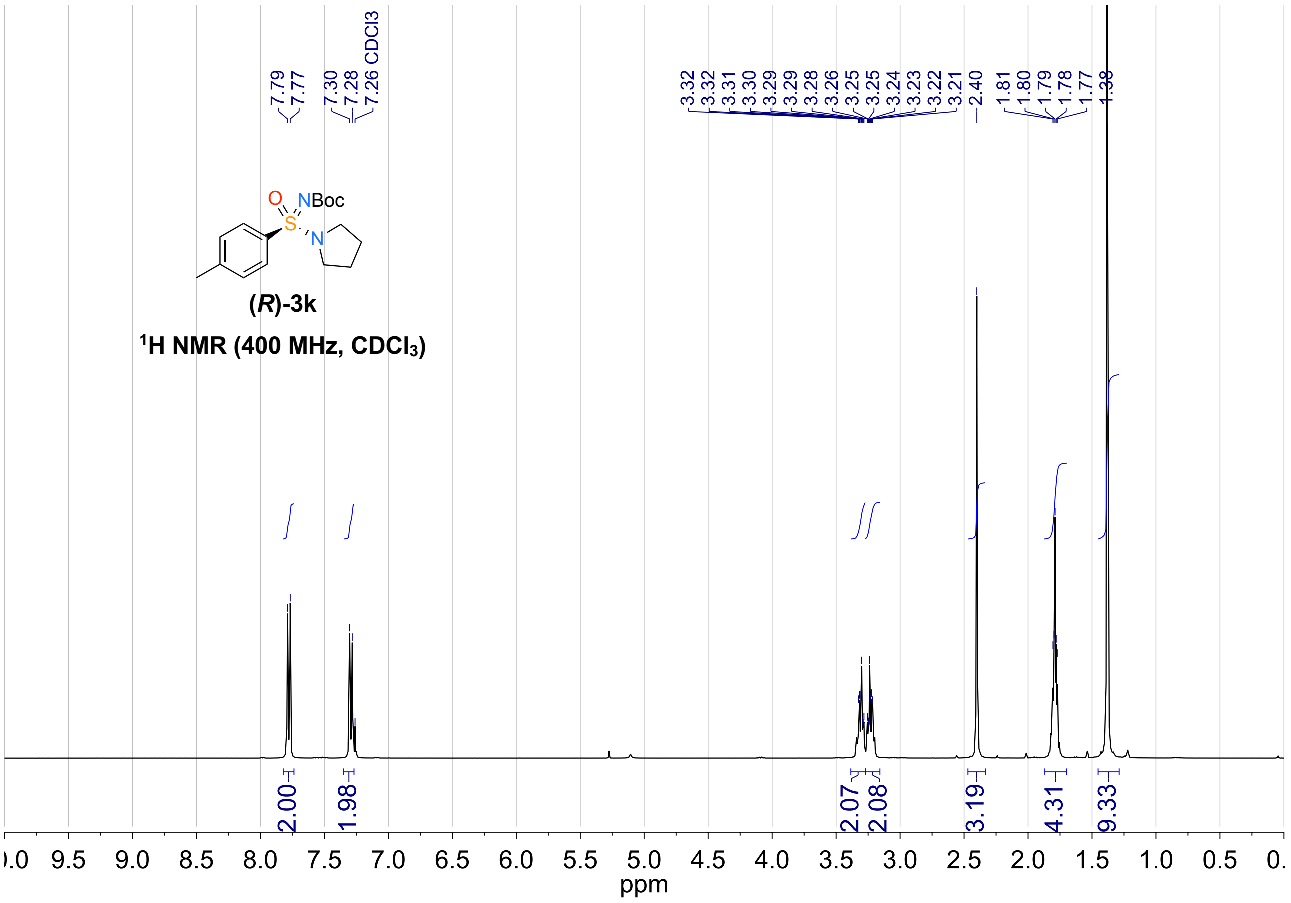

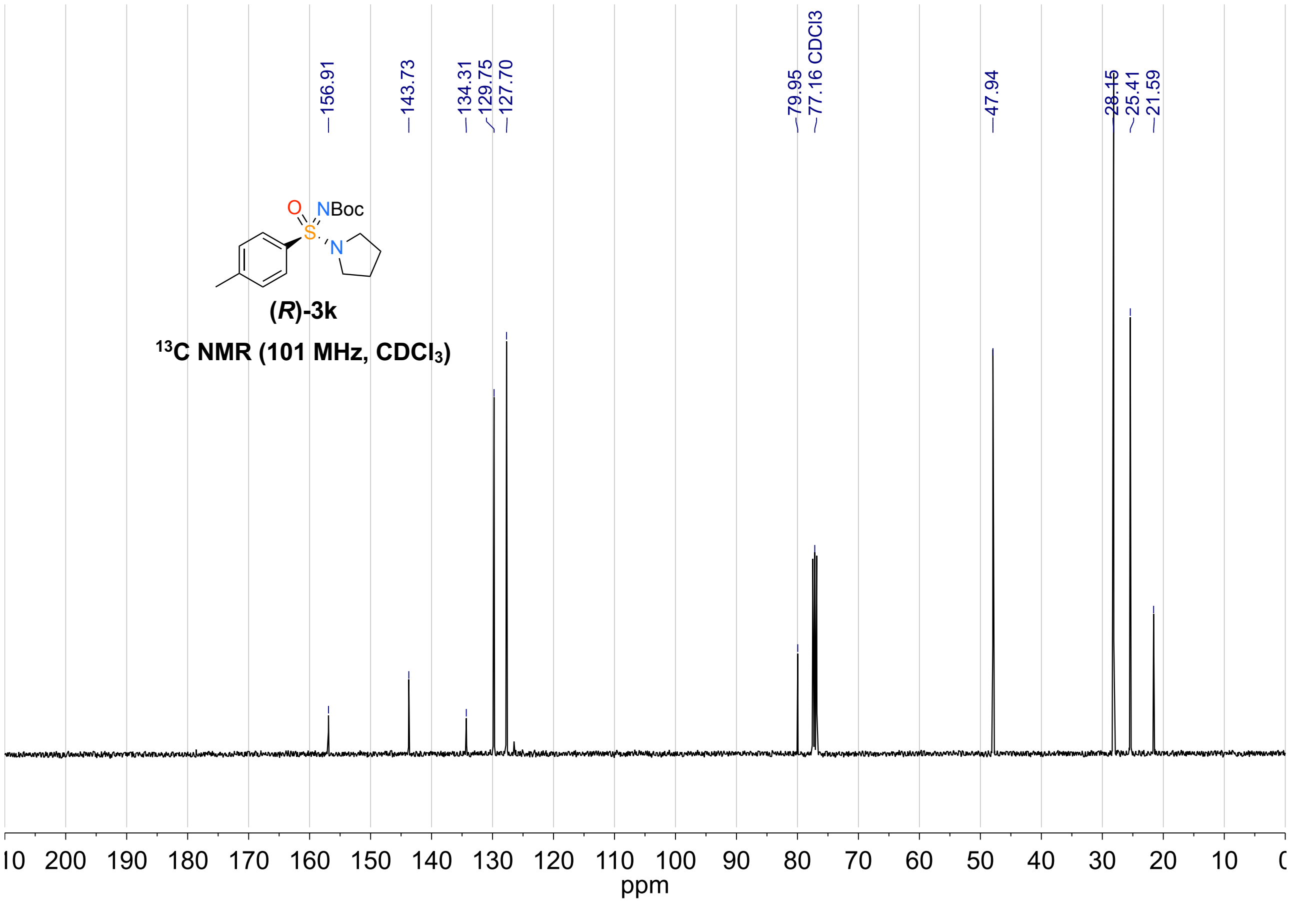

tert-Butyl (R)-(morpholino(oxo)(p-tolyl)-λ^6-sulfaneylidene)carbamate ((R)-3l)

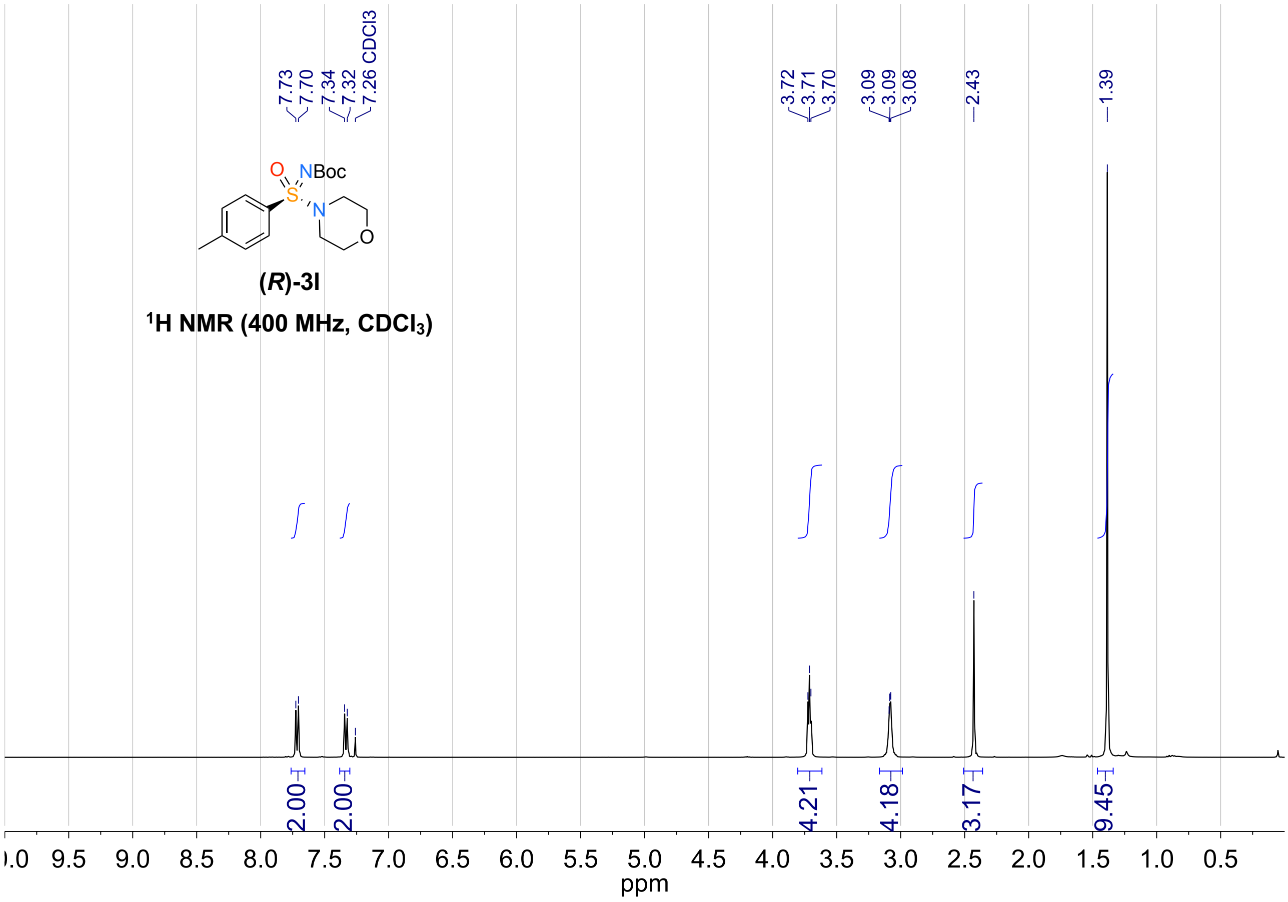

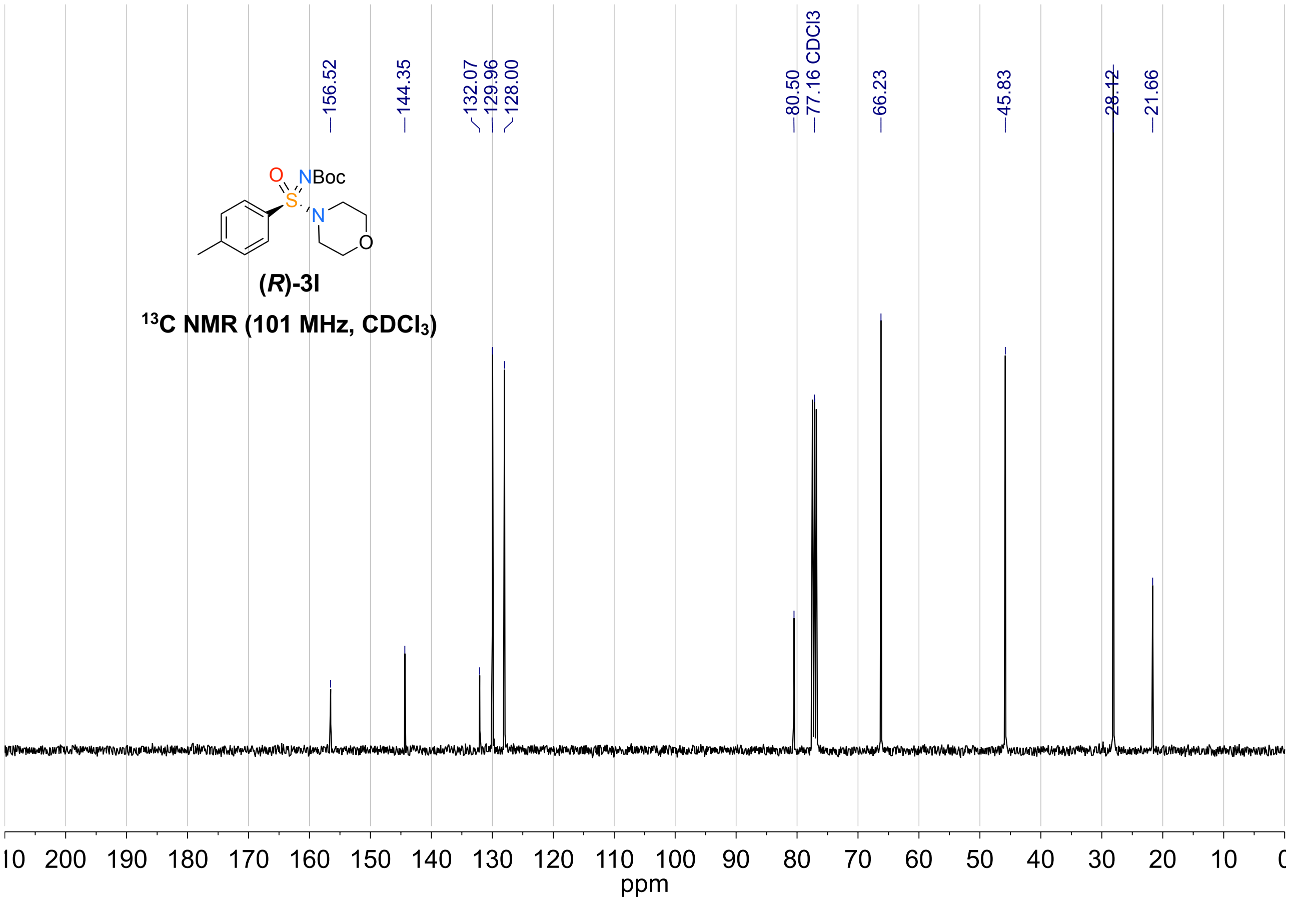

tert-Butyl (R)-(oxo(4-oxopiperidin-1-yl)(p-tolyl)-λ⁶-sulfaneylidene)carbamate ((R)-3m)

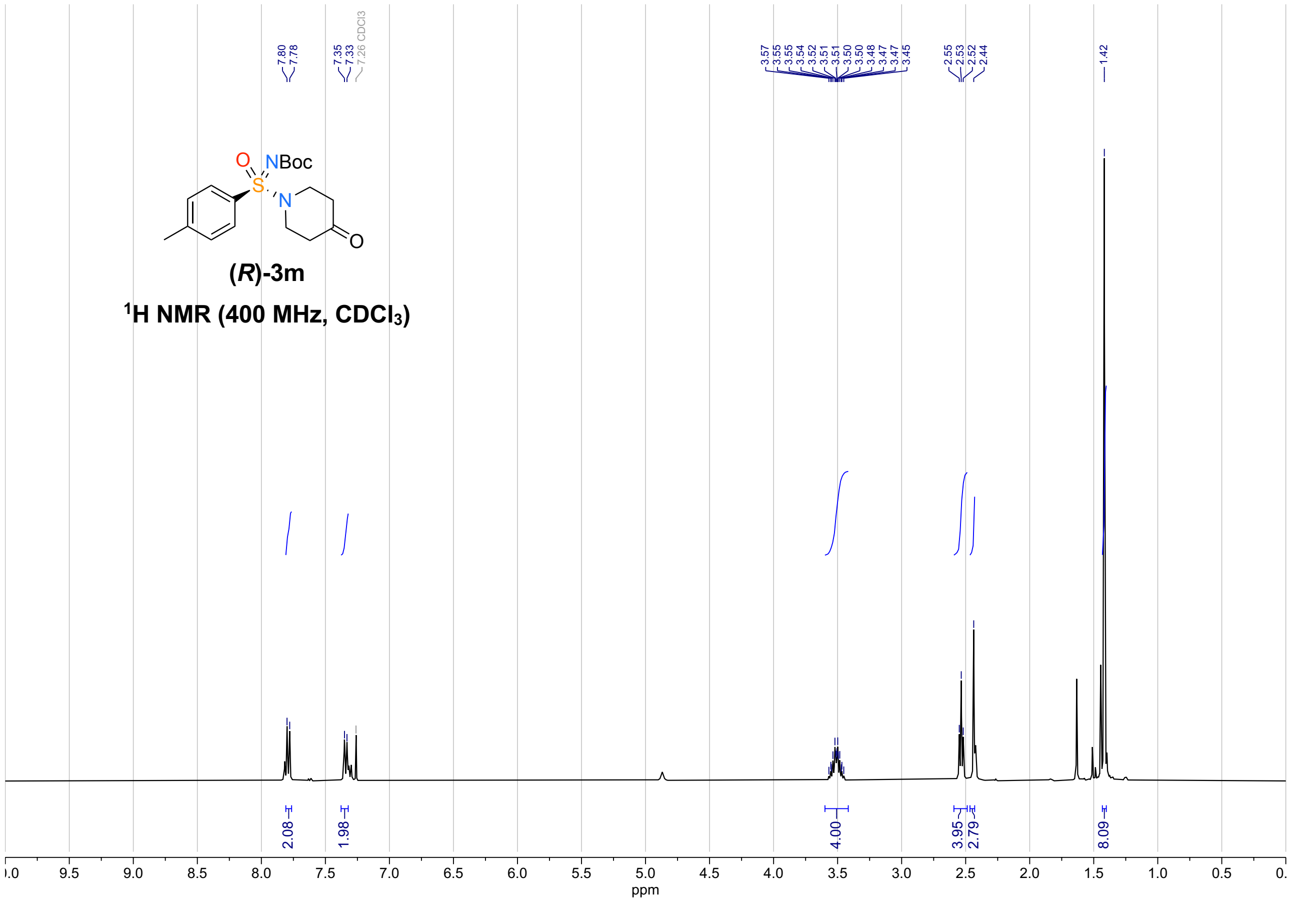

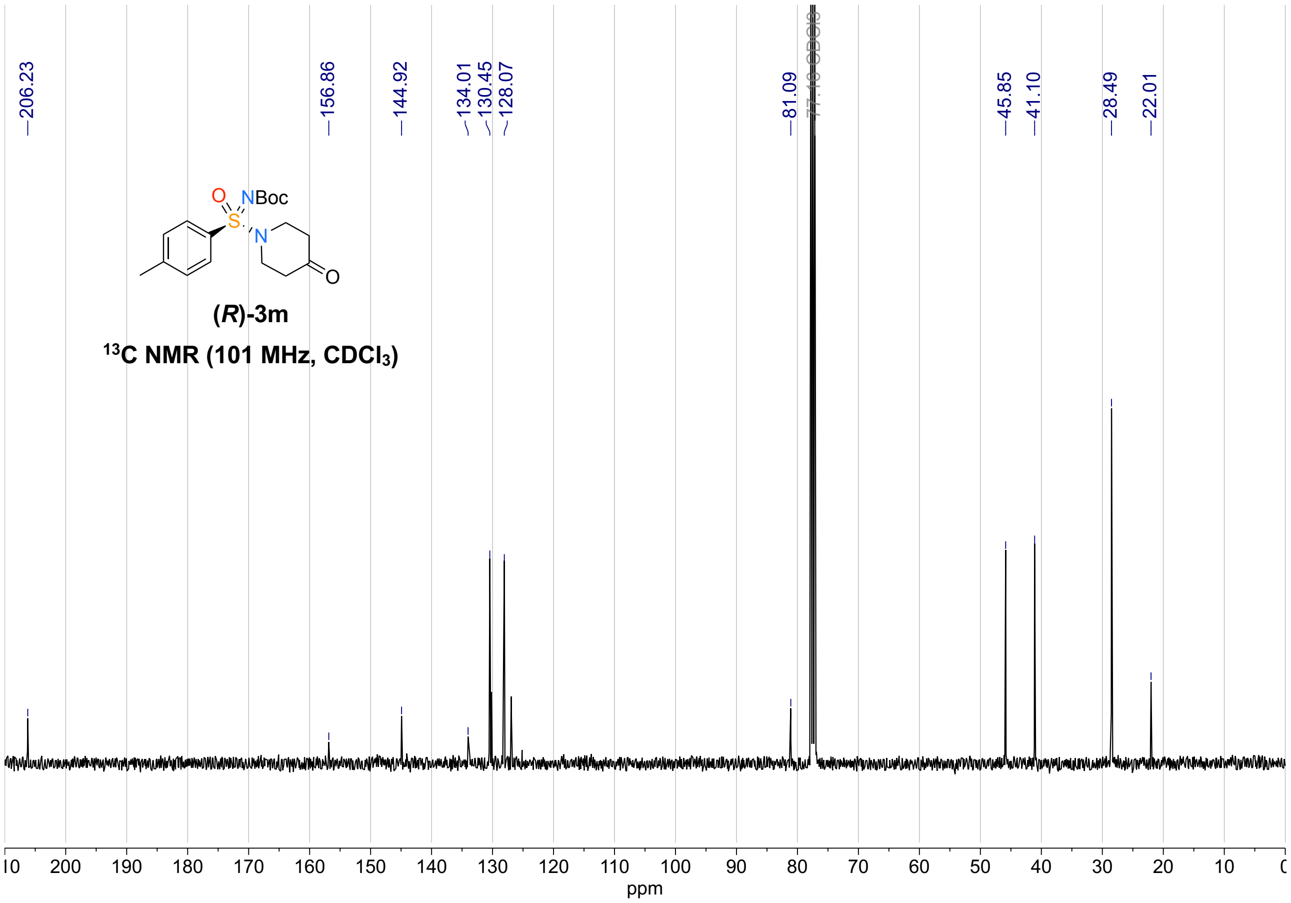

tert-Butyl (*R*)-((4,4-difluoropiperidin-1-yl)(oxo)(*p*-tolyl)-λ^6-sulfaneylidene)carbamate ((*R*)-3n)

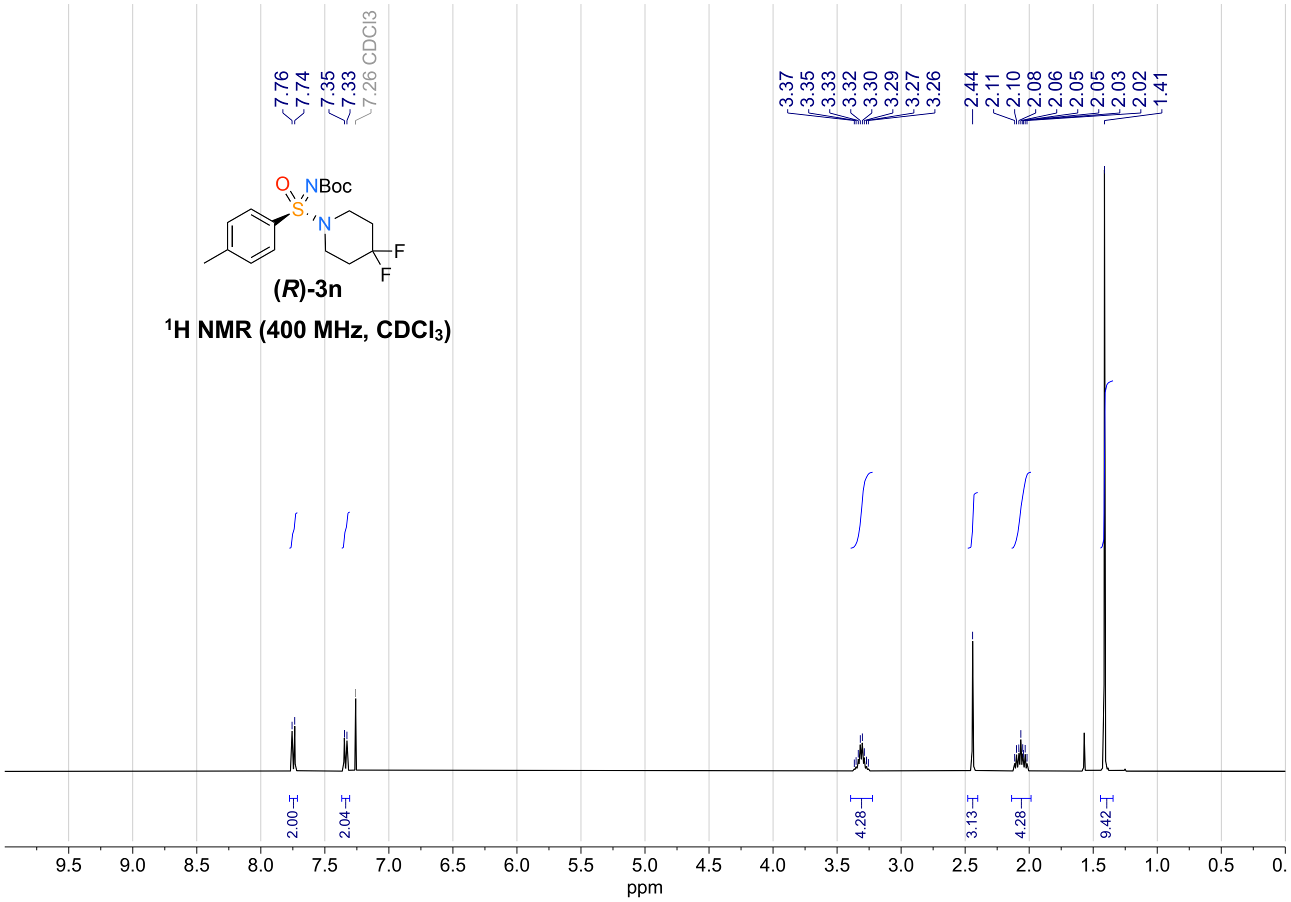

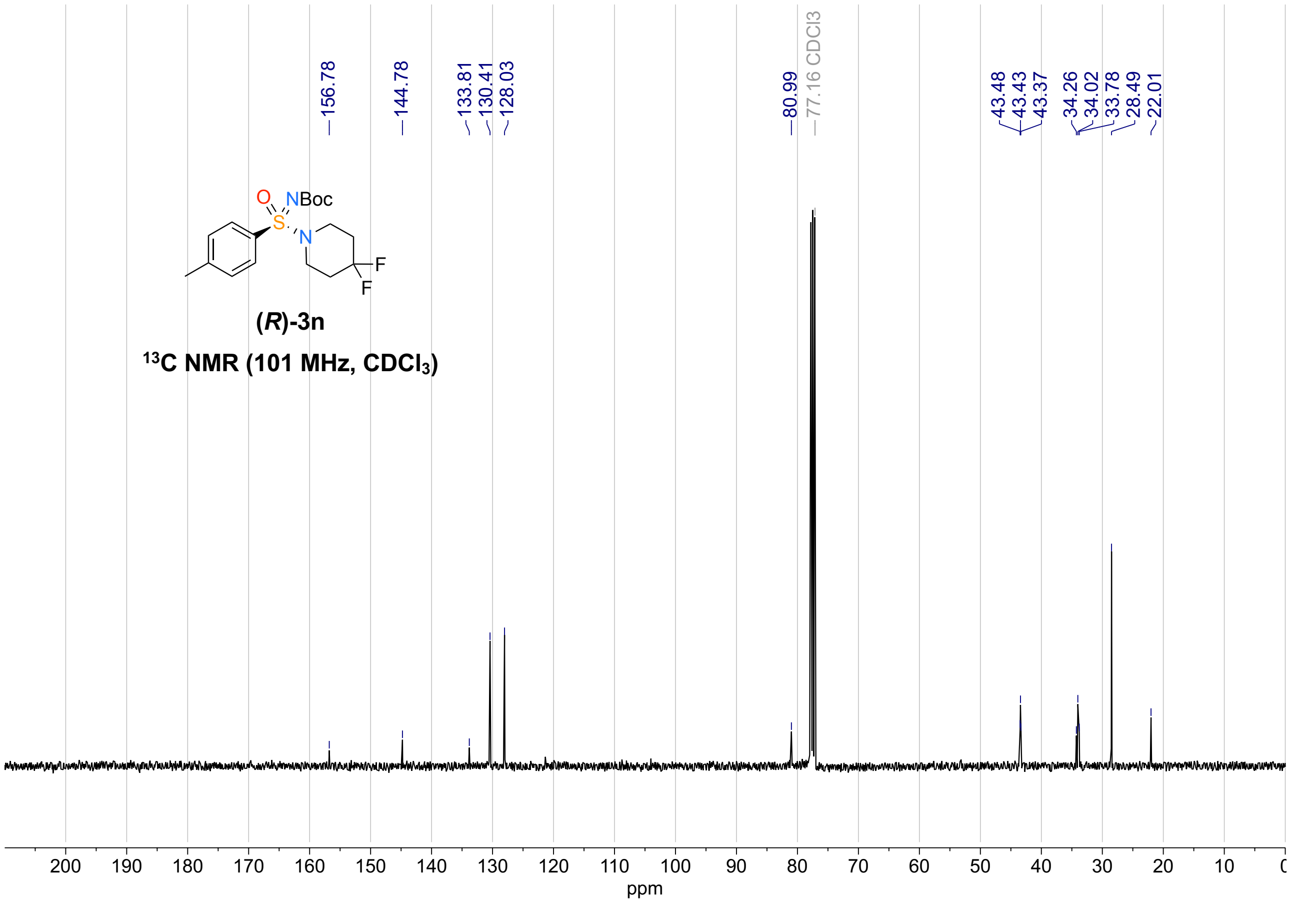

(*R*)-3n

^{19}F NMR (377 MHz, $CDCl_3$)

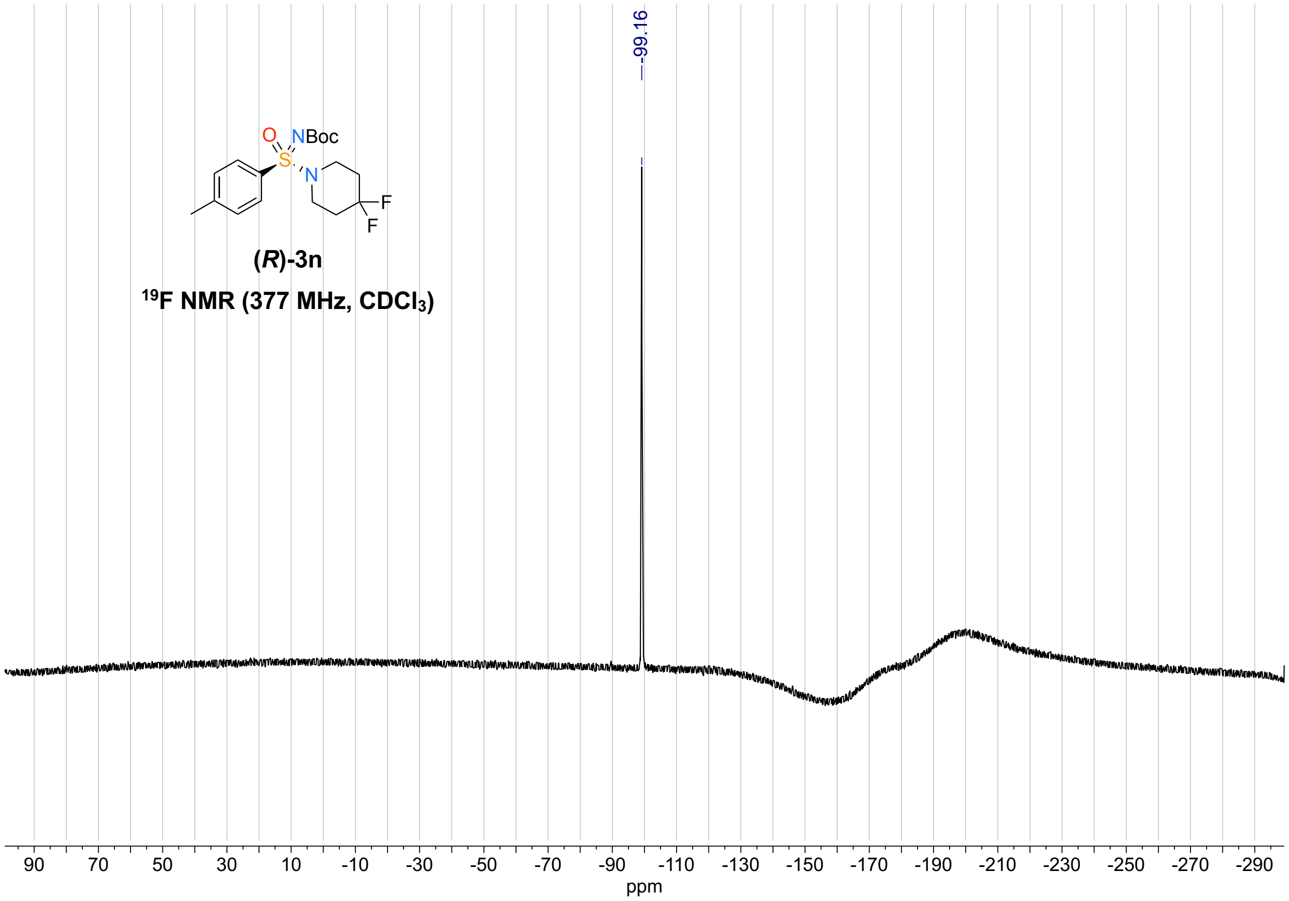

tert-Butyl (R)-((4-hydroxypiperidin-1-yl)(oxo)(p-tolyl)-λ⁶-sulfaneylidene)carbamate ((R)-3o)

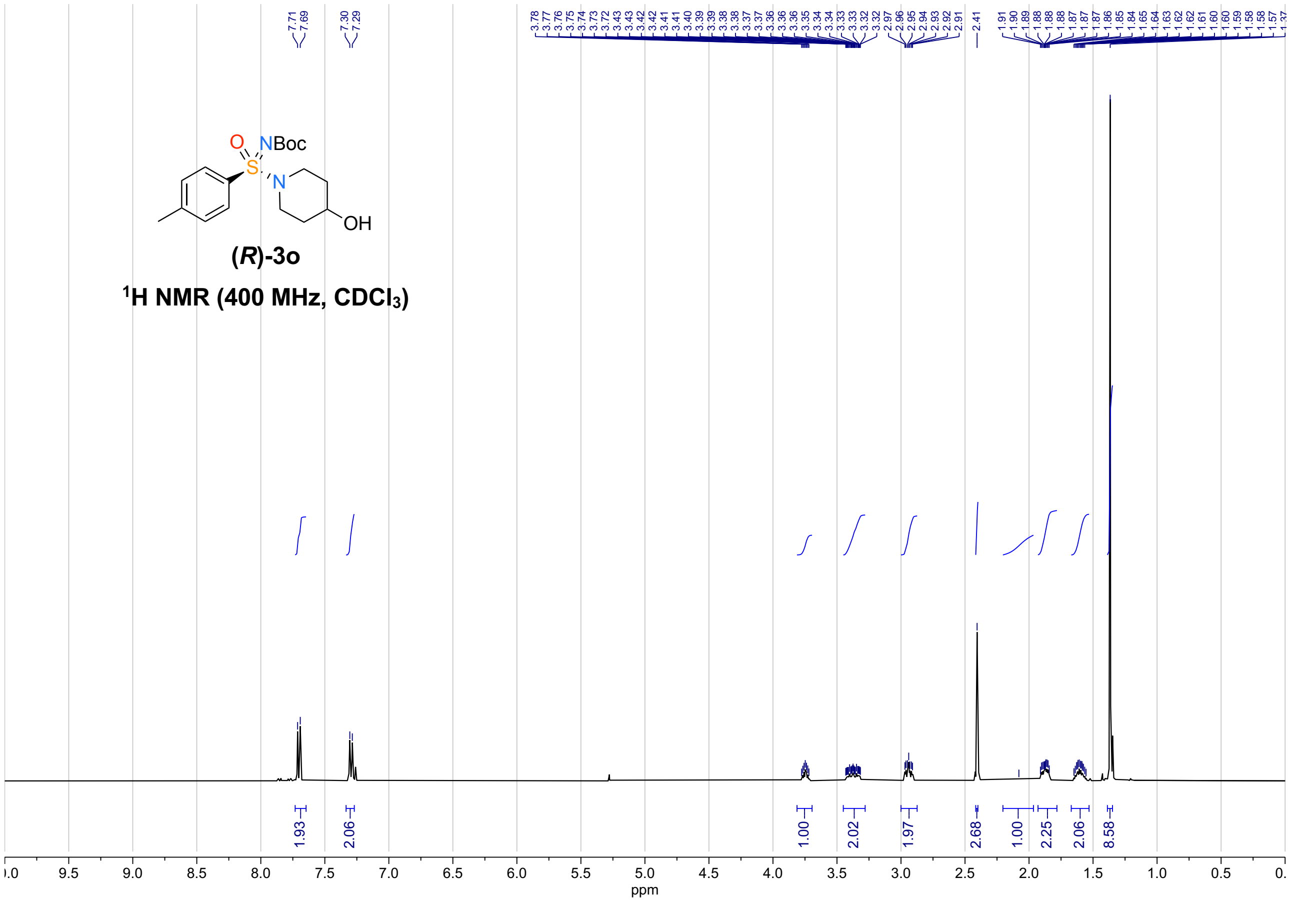

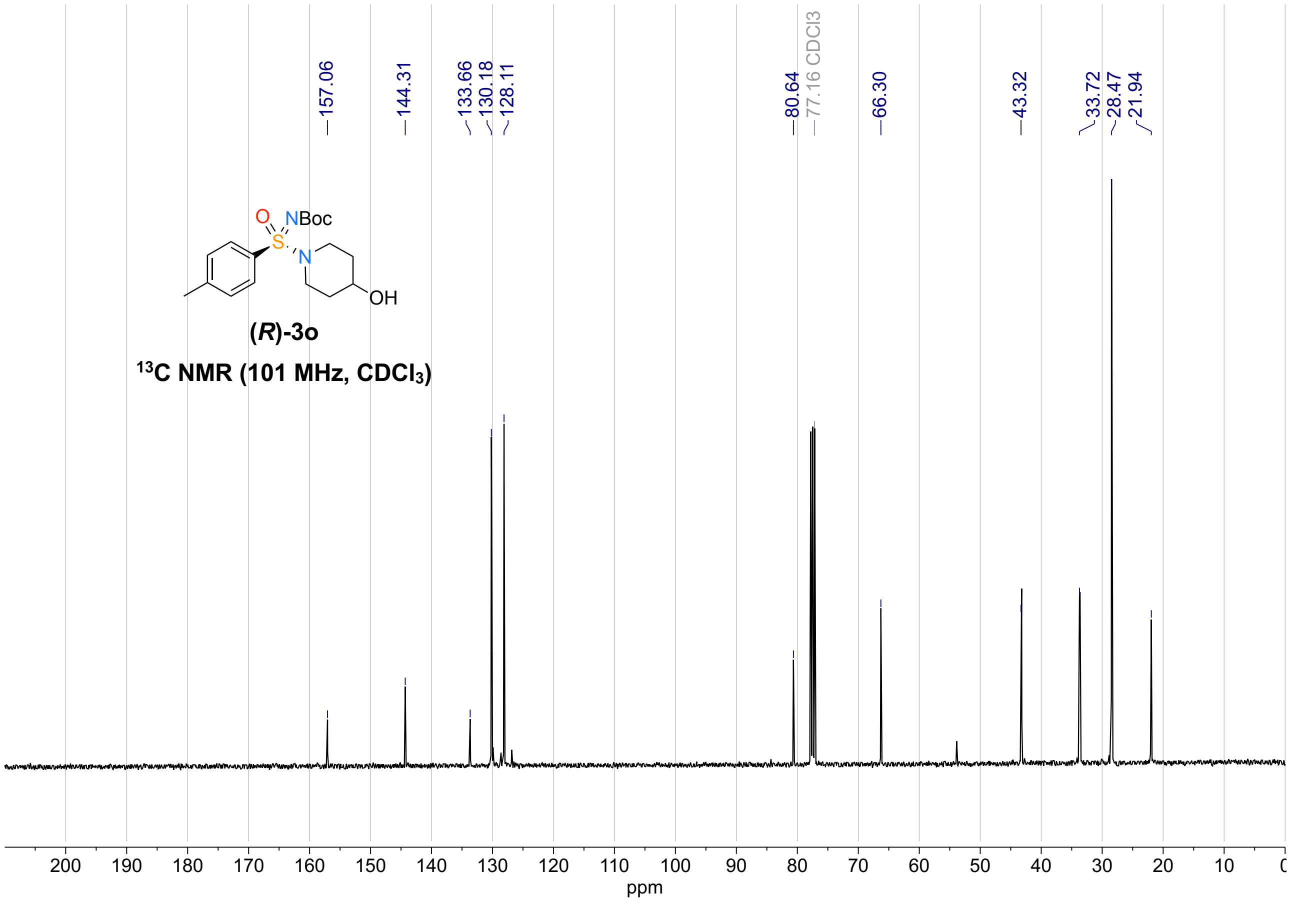

tert-Butyl (R)-4-(N-(tert-butoxycarbonyl)-4-methylphenylsulfonimidoyl)piperazine-1-carboxylate ((R)-3p)

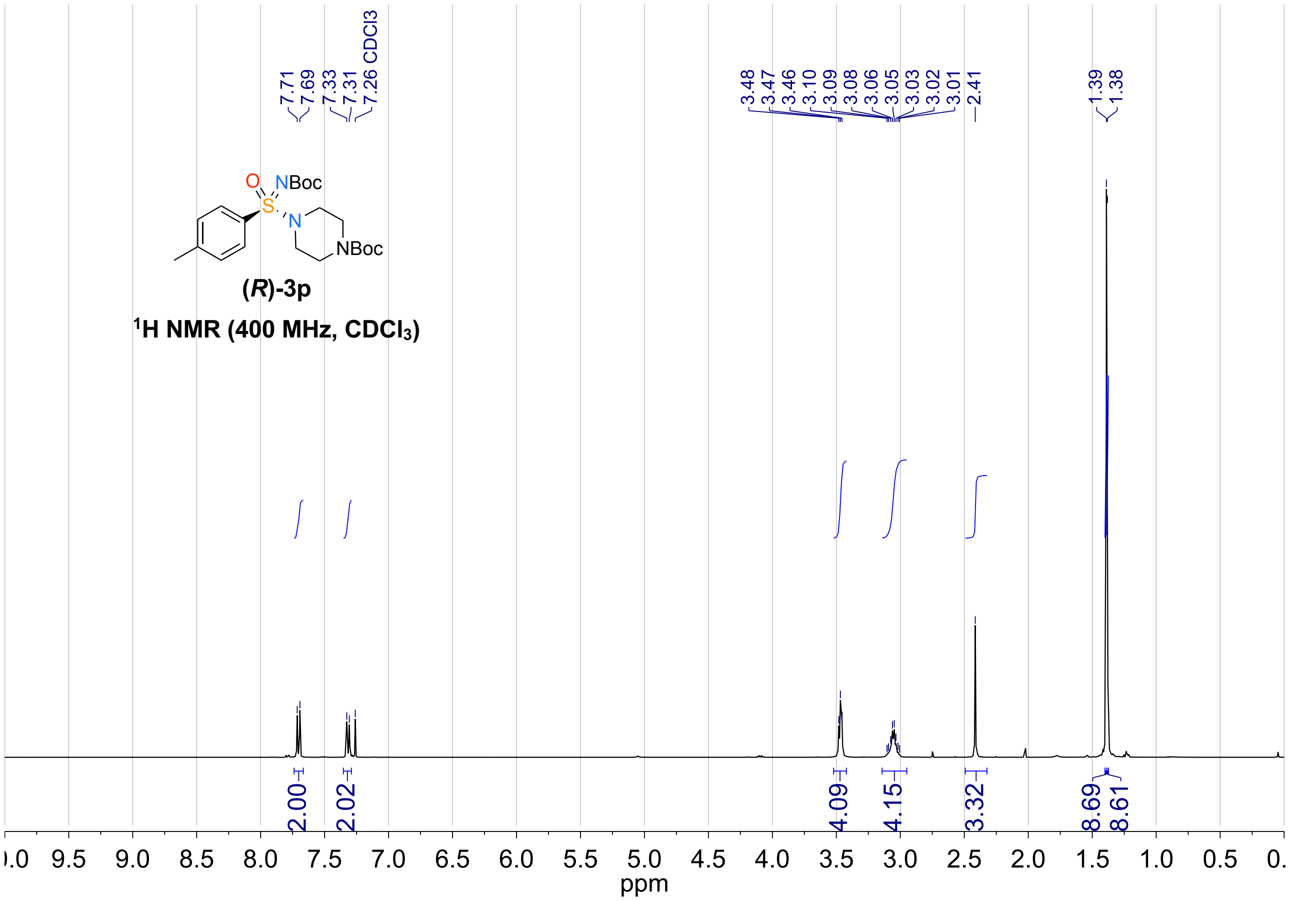

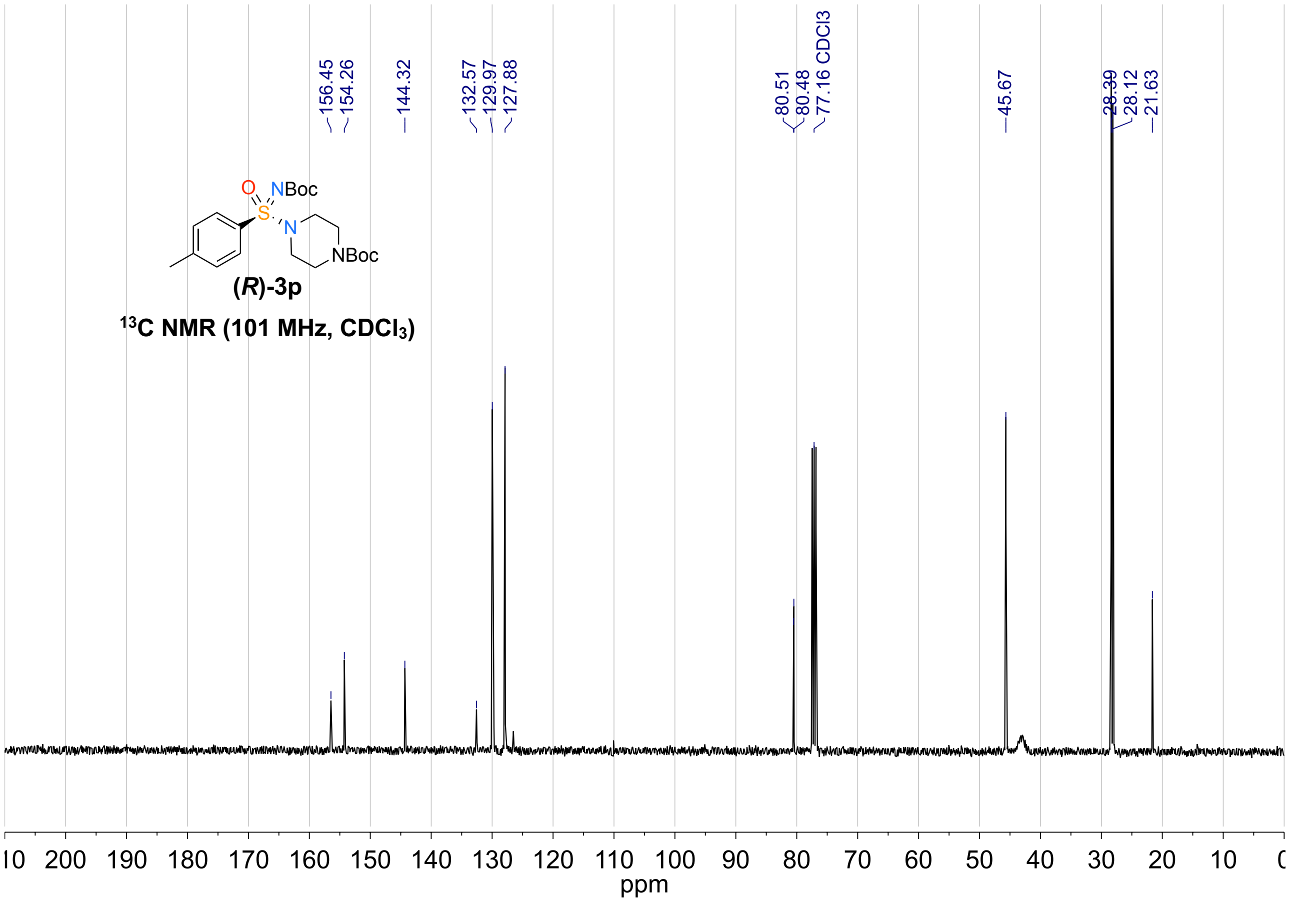

tert-Butyl (*R*)-(oxo(4-(pyrimidin-2-yl)piperazin-1-yl)(*p*-tolyl)-λ⁶-sulfaneylidene)carbamate ((*R*)-3q)

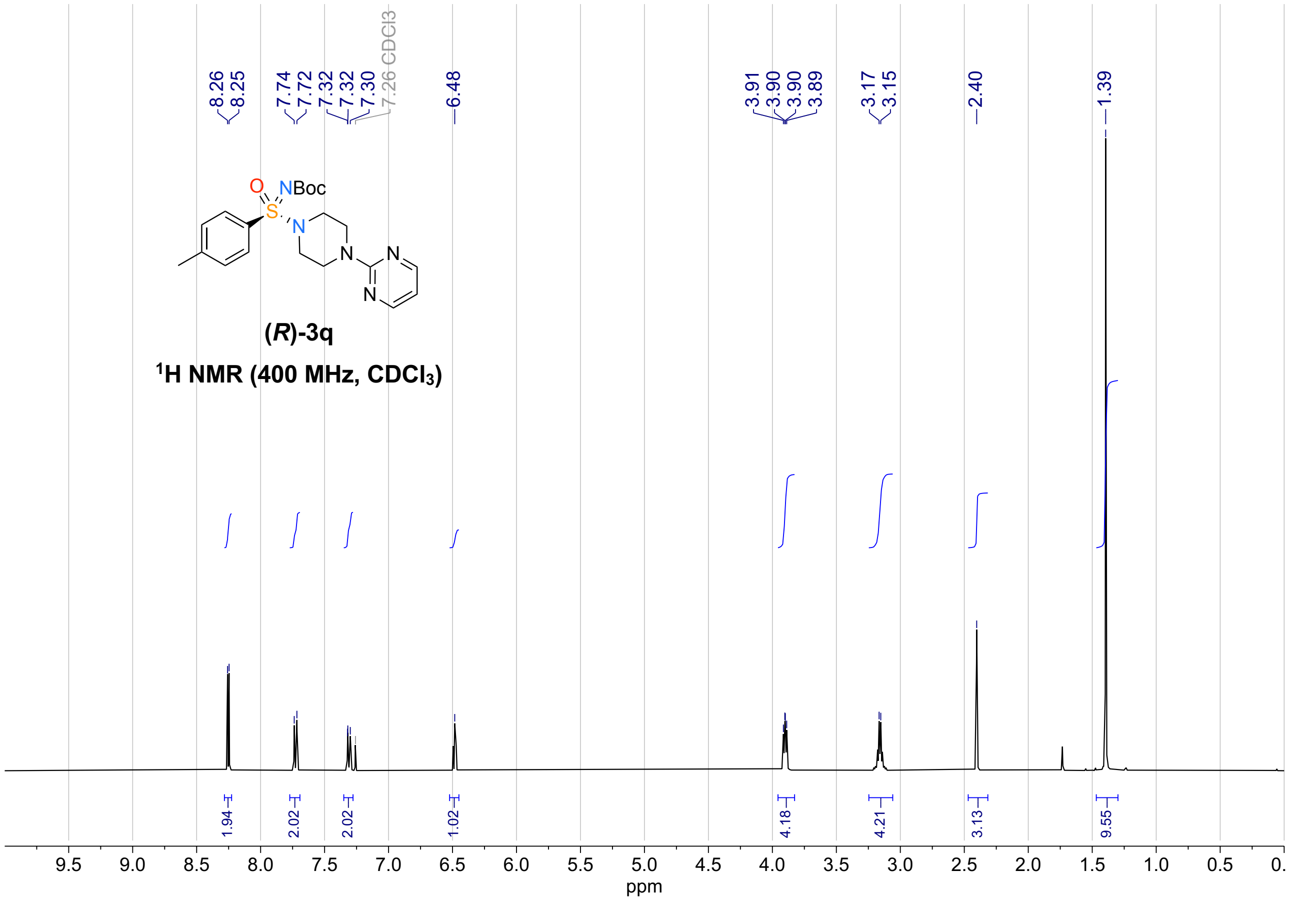

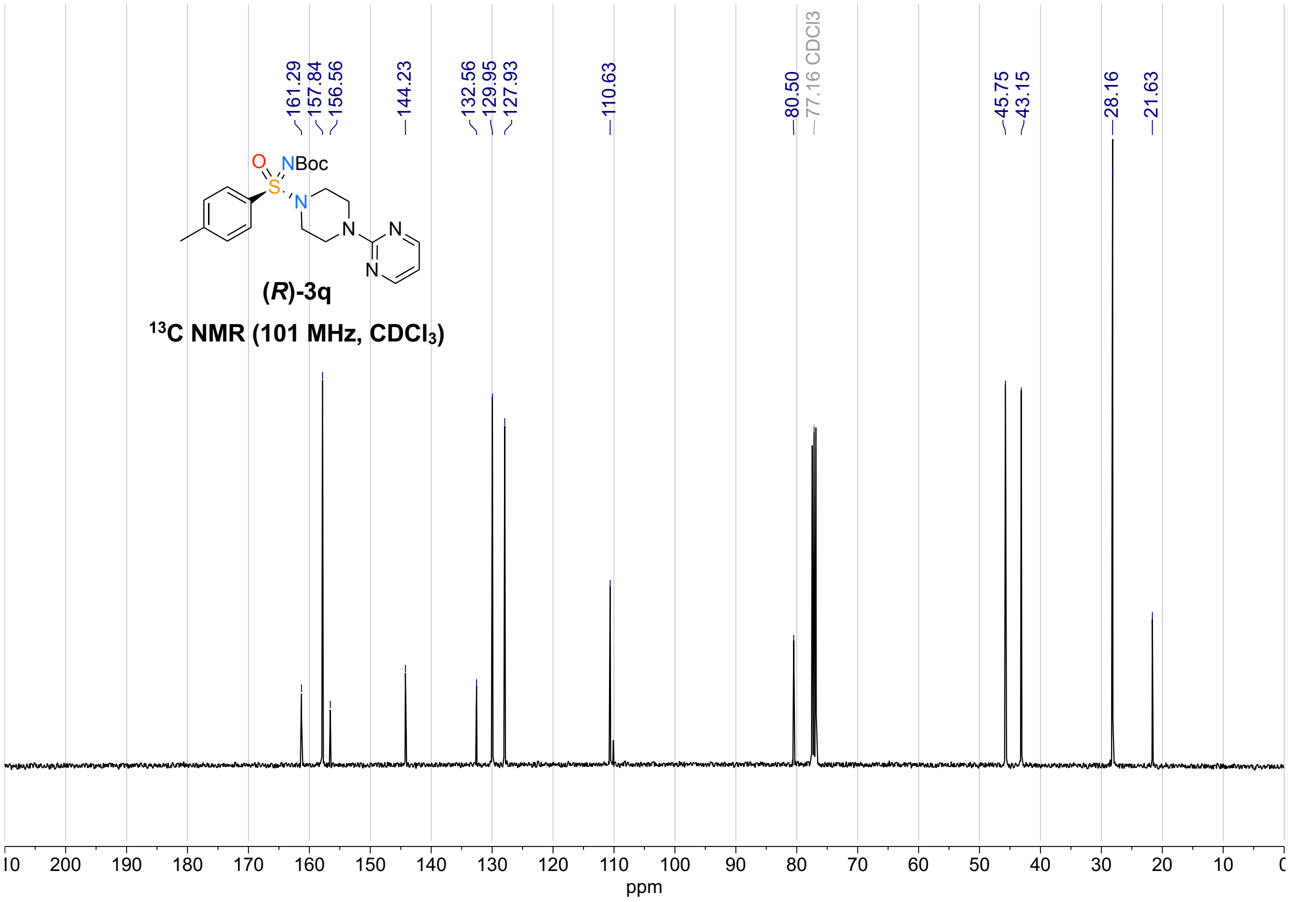

tert-Butyl (_R_)-((4-(6-fluorobenzo[_d_]isoxazol-3-yl)piperidin-1-yl)(oxo)(_p_-tolyl)-λ[6]-sulfaneylidene)carbamate ((_R_)-3r)

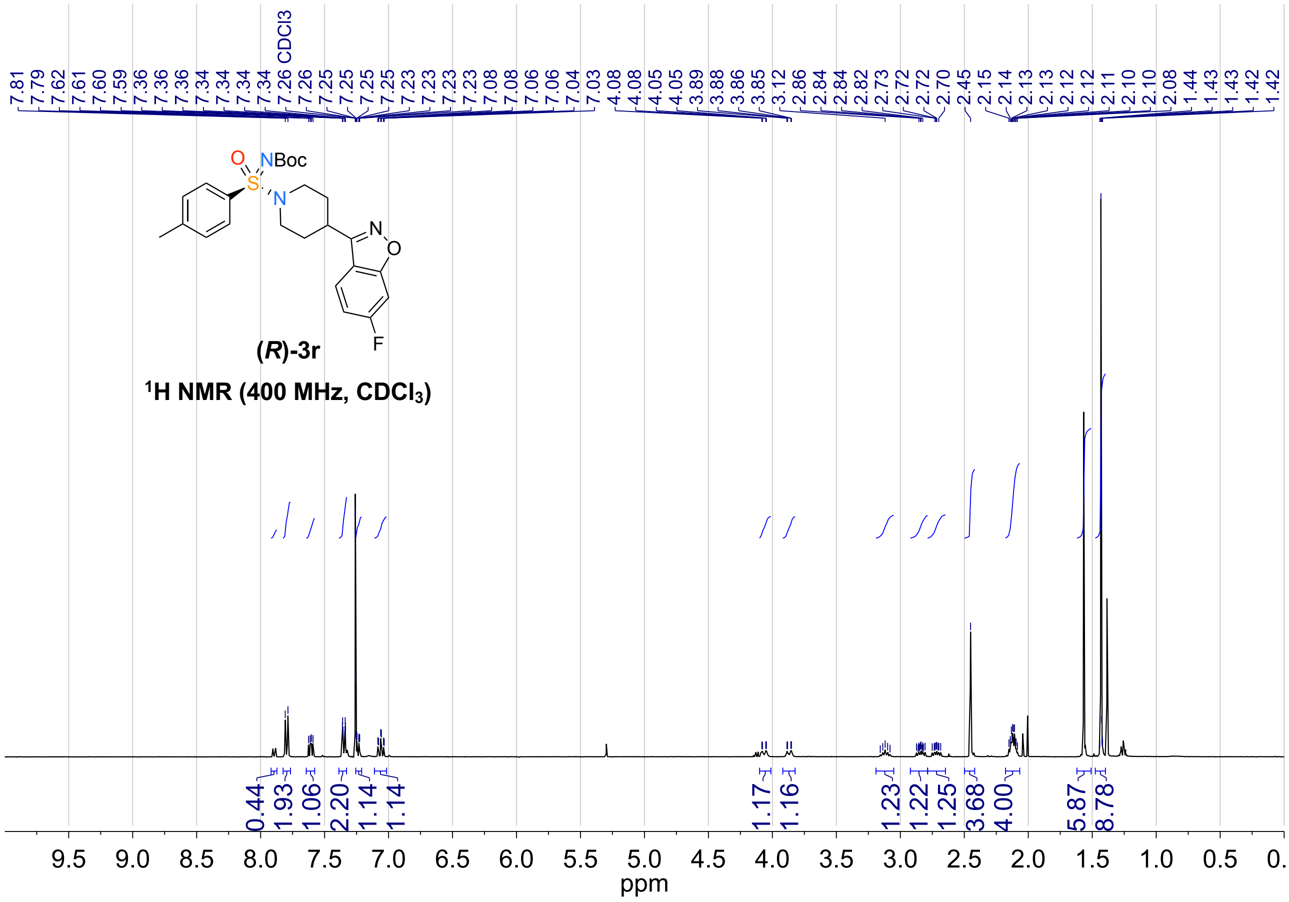

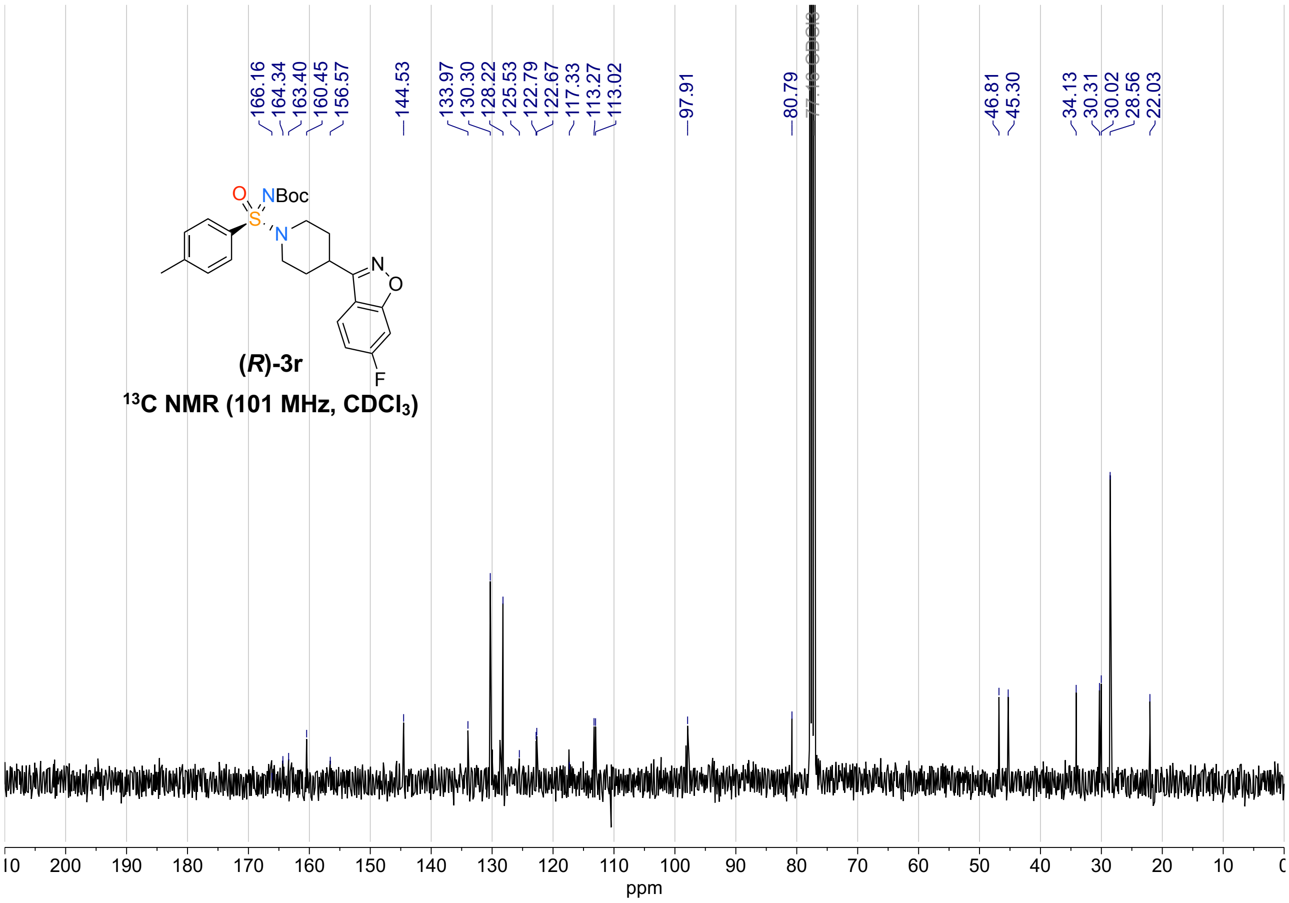

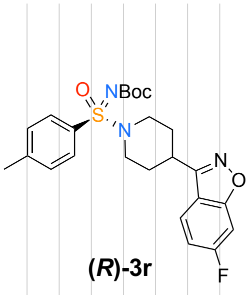

(*R*)-3r

^{19}F NMR (377 MHz, $CDCl_3$)

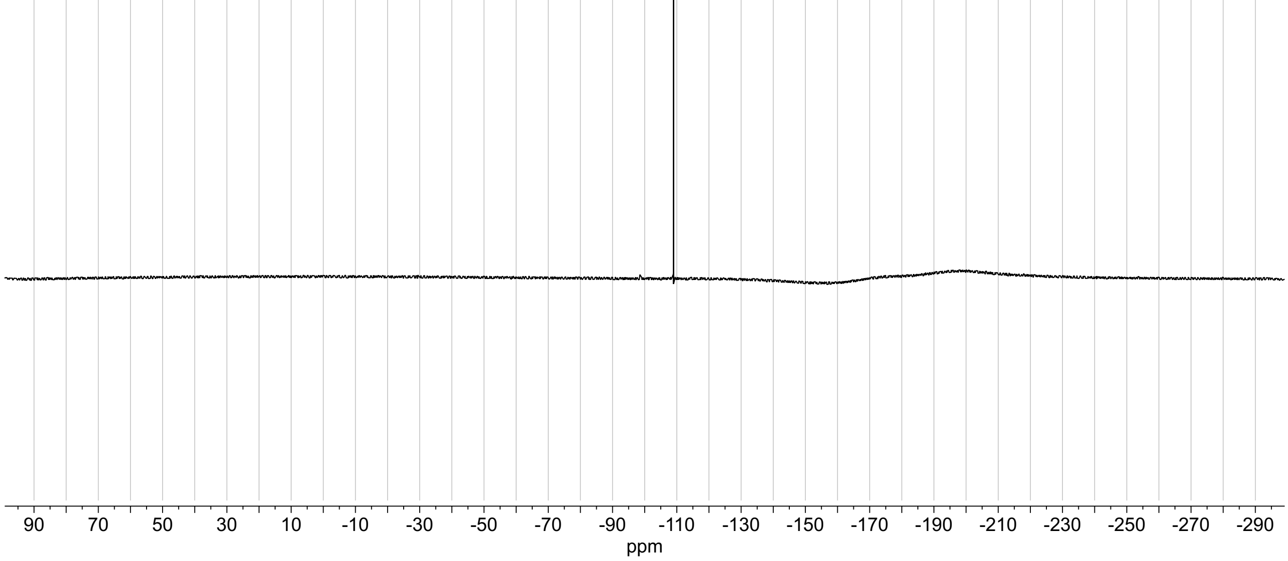

tert-Butyl (_R_)-(((3-(10,11-dihydro-5_H_-dibenzo[_b_,_f_]azepin-5-yl)propyl)(methyl)amino)(oxo)(_p_-tolyl)-λ^6-sulfaneylidene)carbamate ((_R_)-3s)

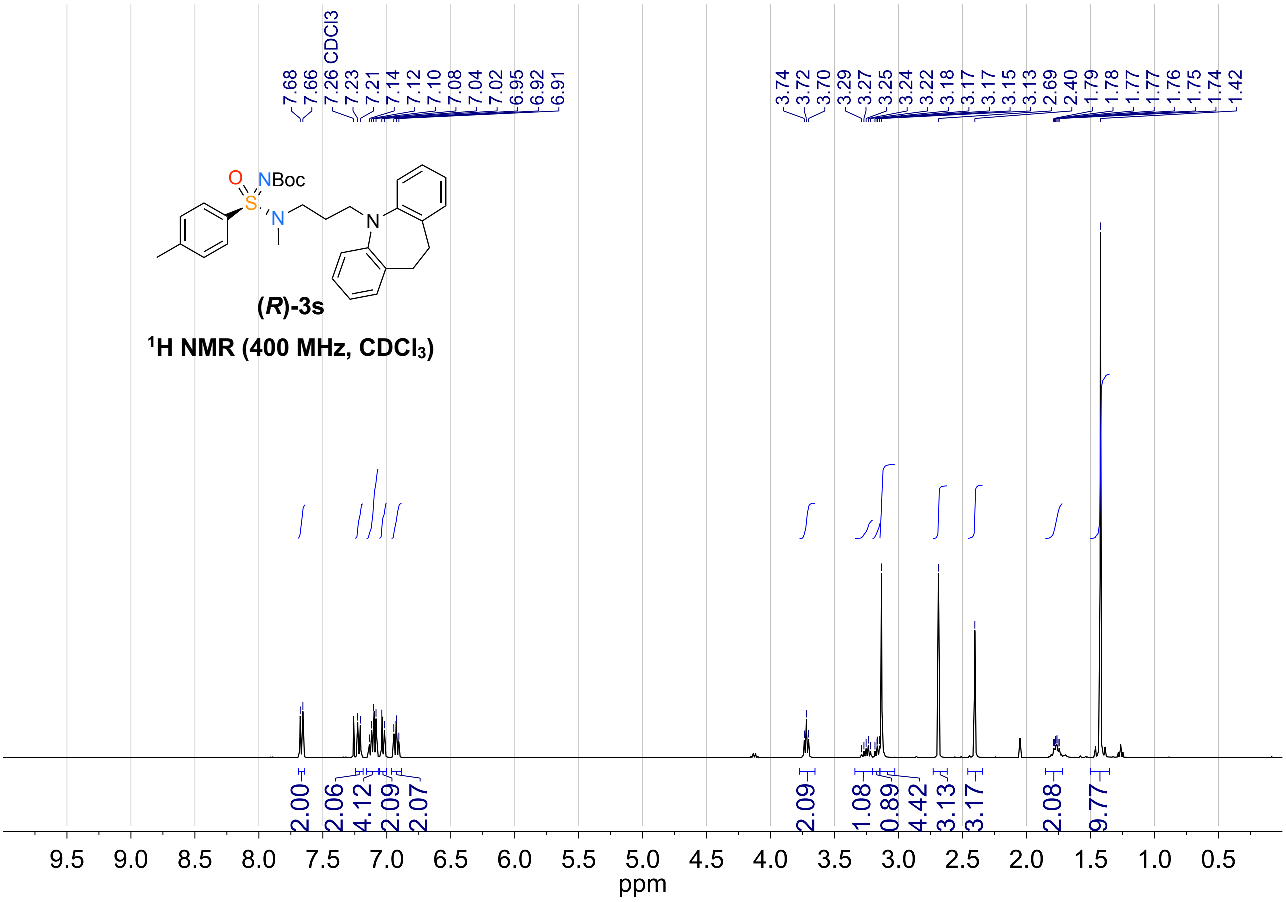

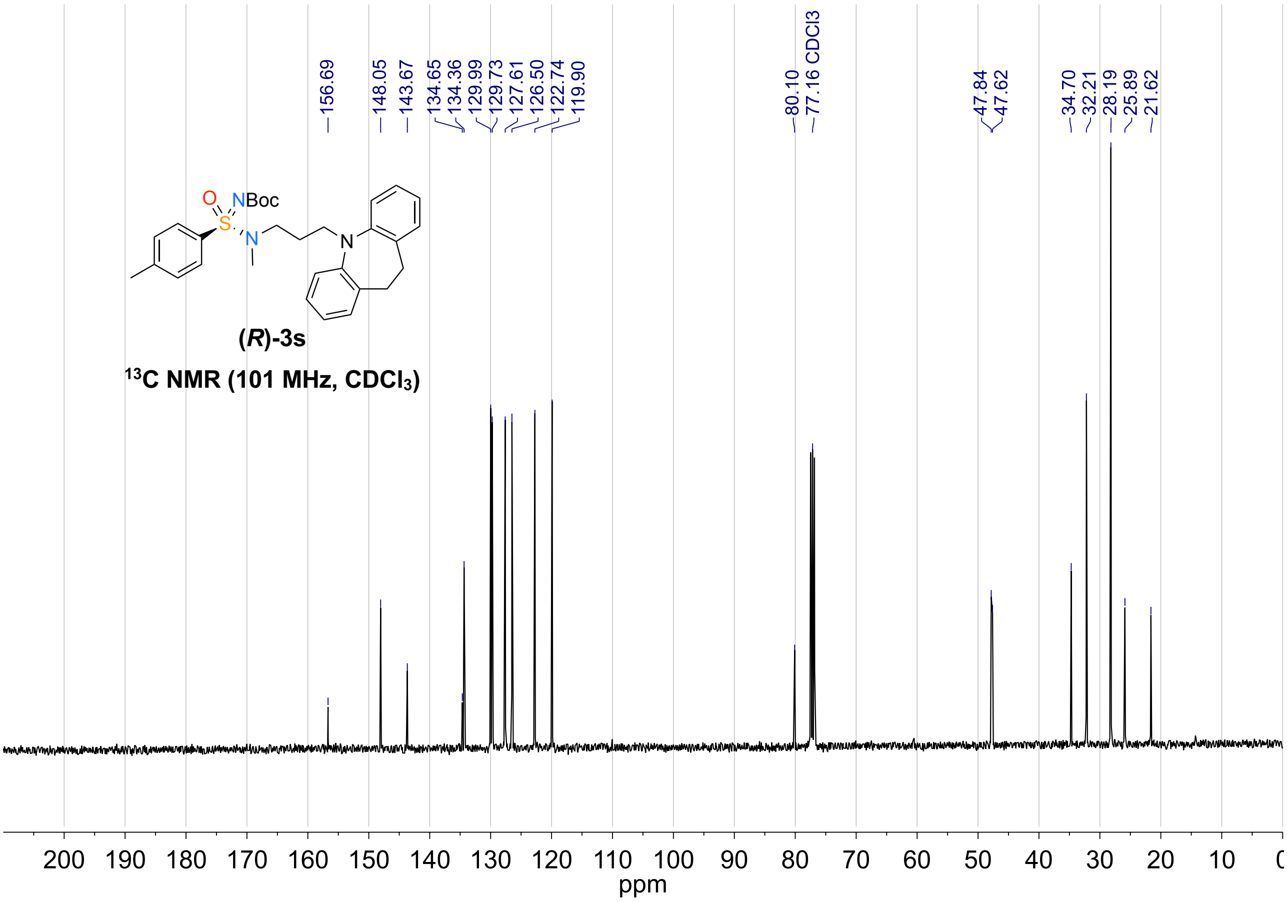

tert-Butyl ((_R_)-oxo(((_S_)-1-phenylethyl)amino)(_p_-tolyl)-λ^6^-sulfaneylidene)carbamate ((_R_)-3t)

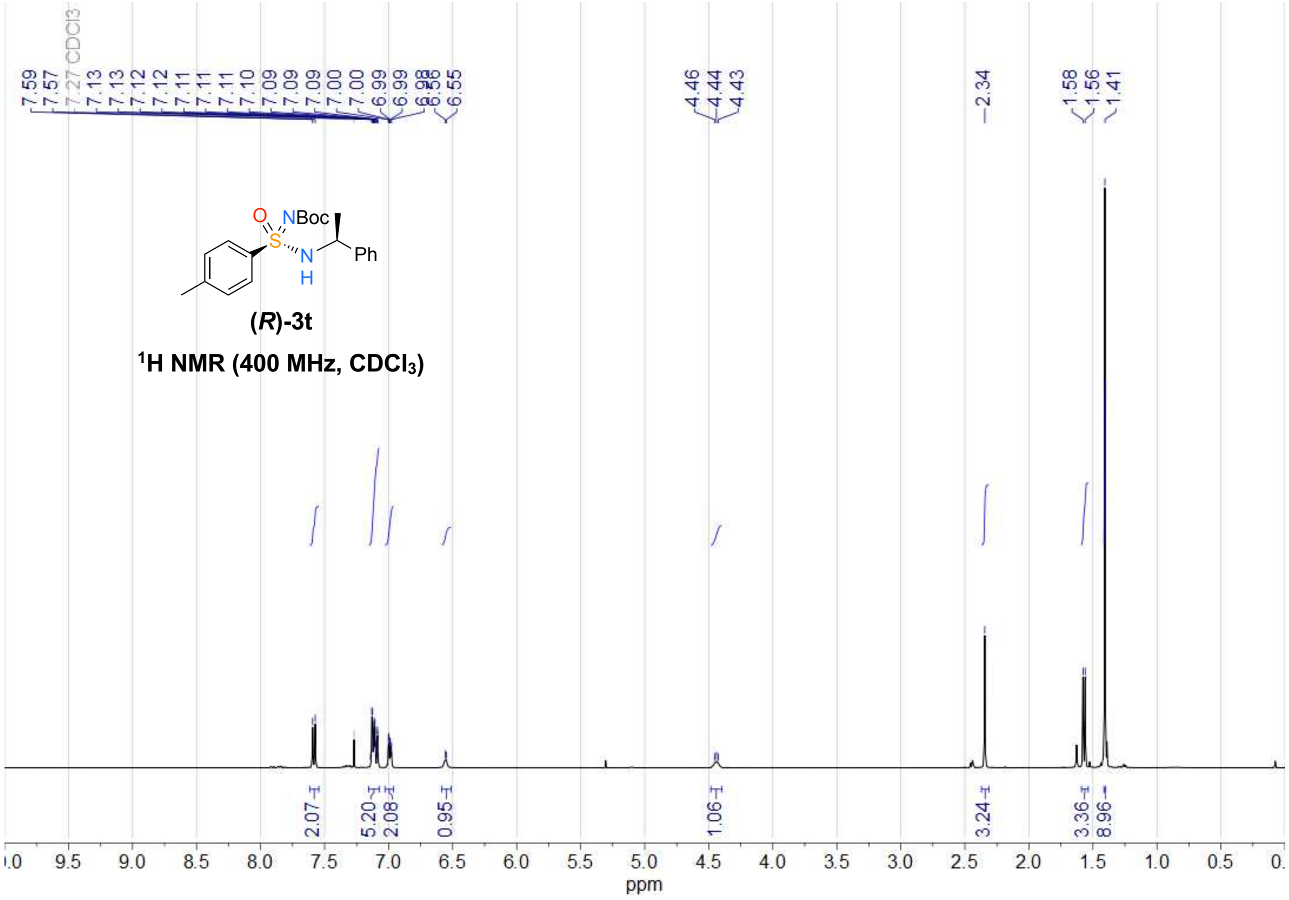

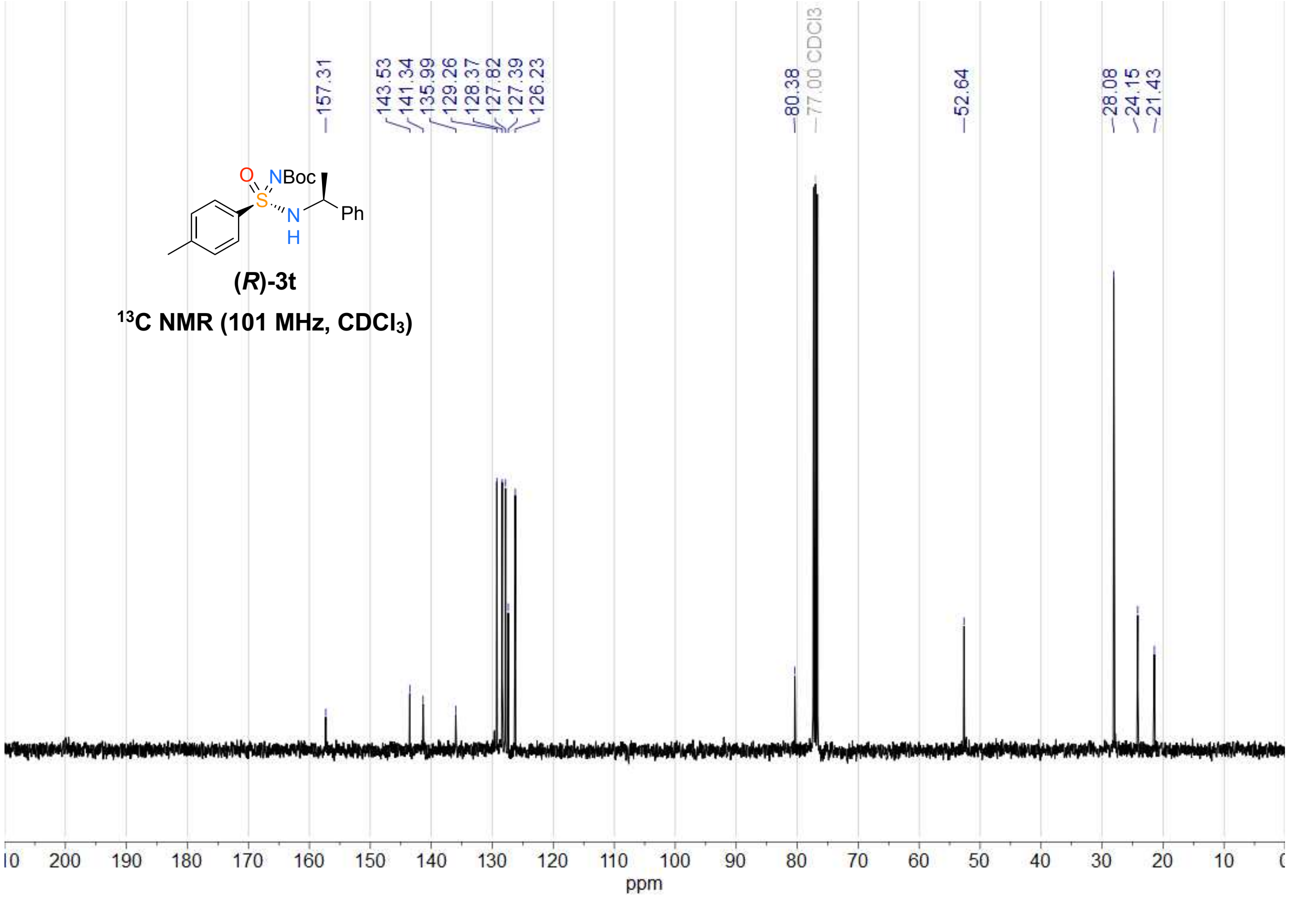

tert-Butyl ((*R*)-oxo(((*R*)-1-phenylethyl)amino)(*p*-tolyl)-λ⁶-sulfaneylidene)carbamate ((*R*)-3u)

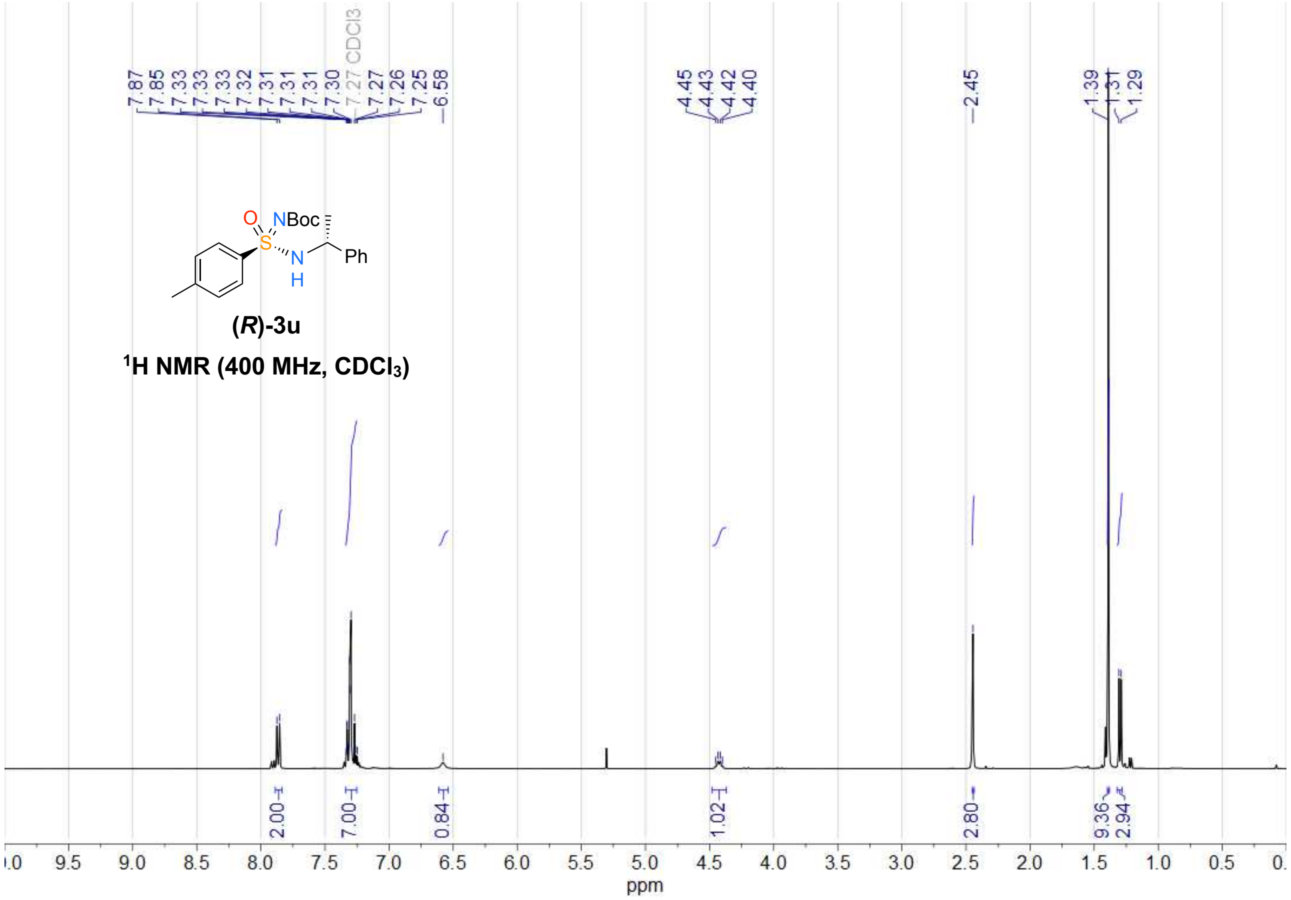

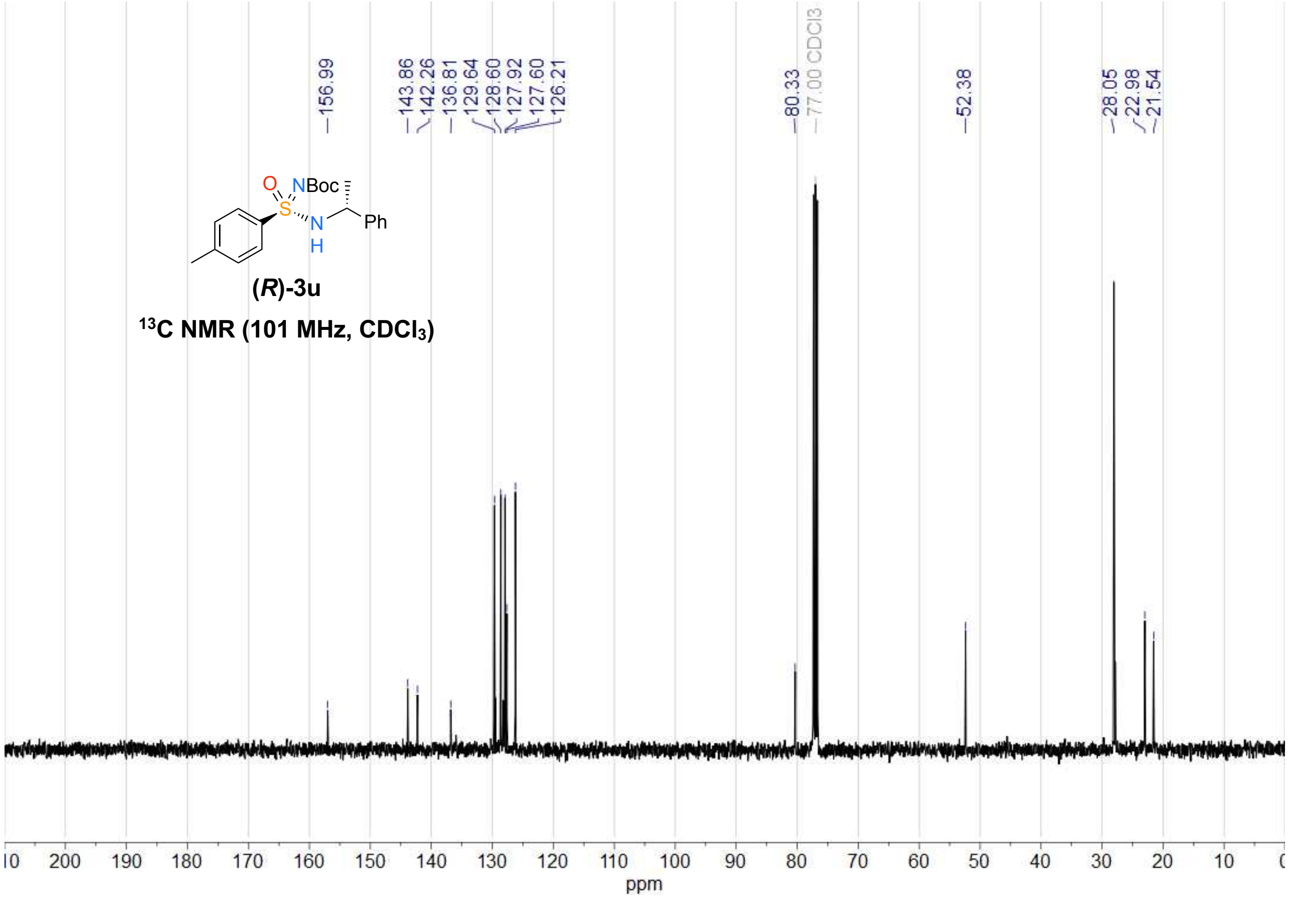

Methyl 3-((4-bromophenyl)thio)propanoate (7)

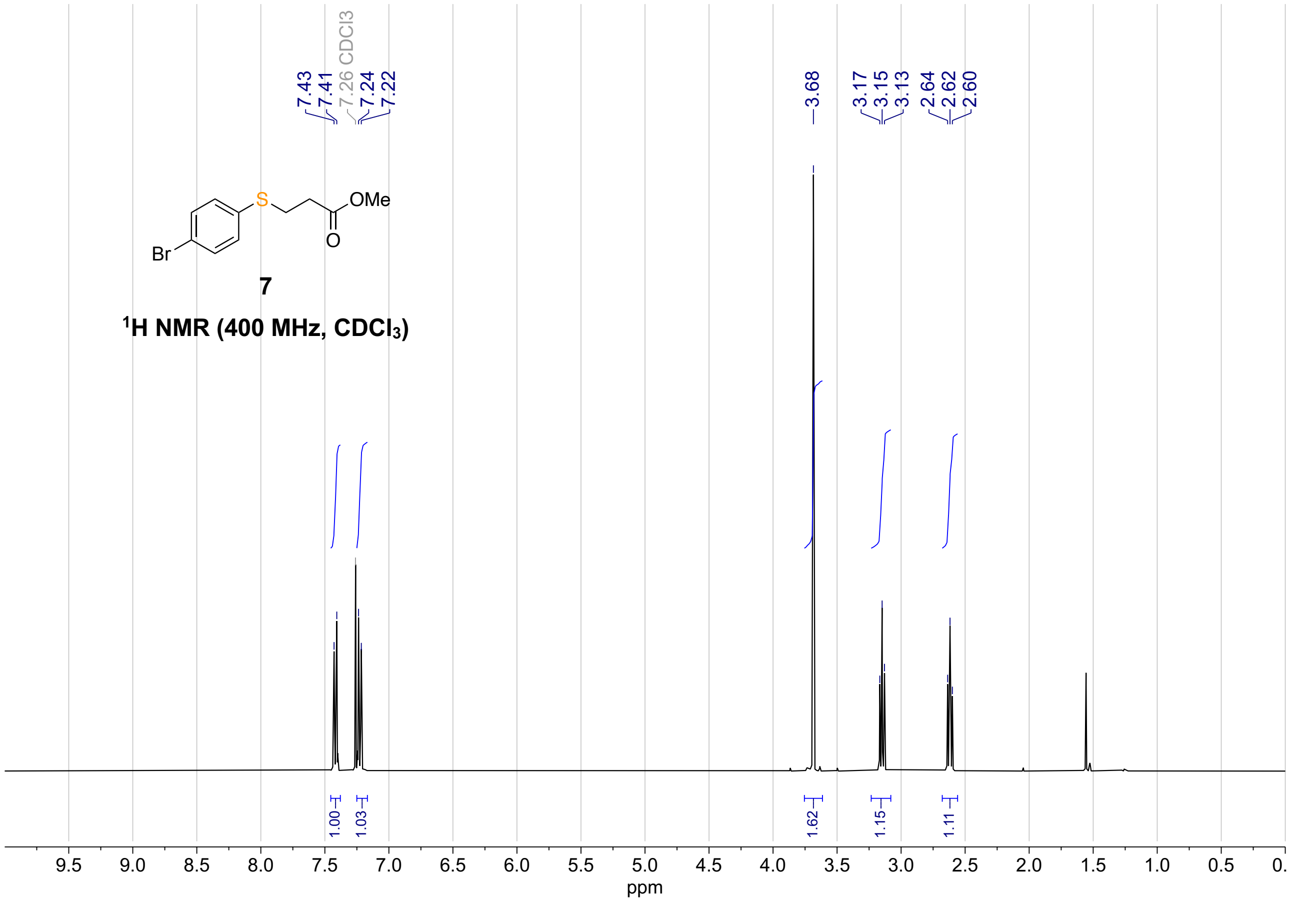

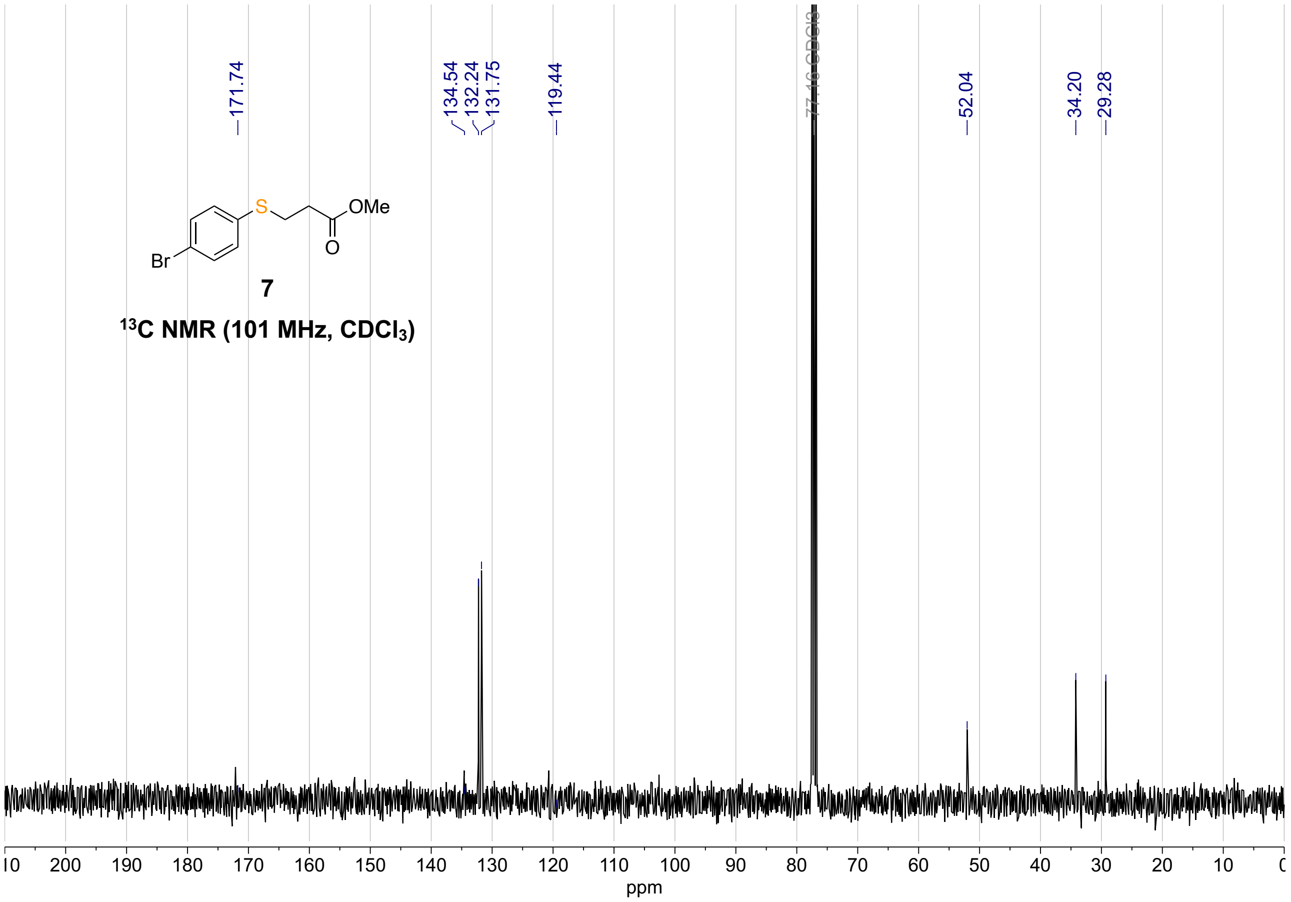

Methyl 3-((4-bromophenyl)sulfinyl)propanoate (8)

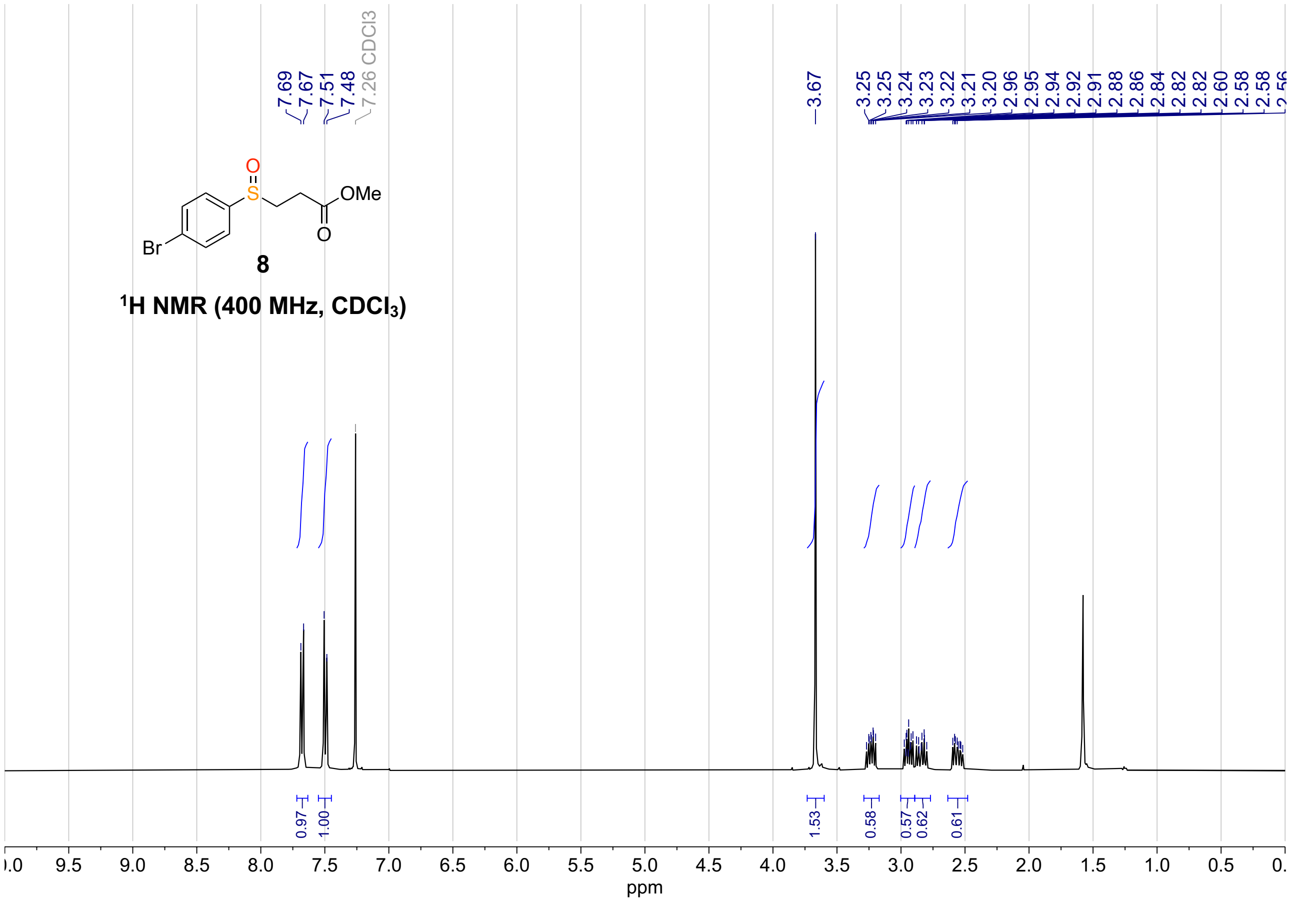

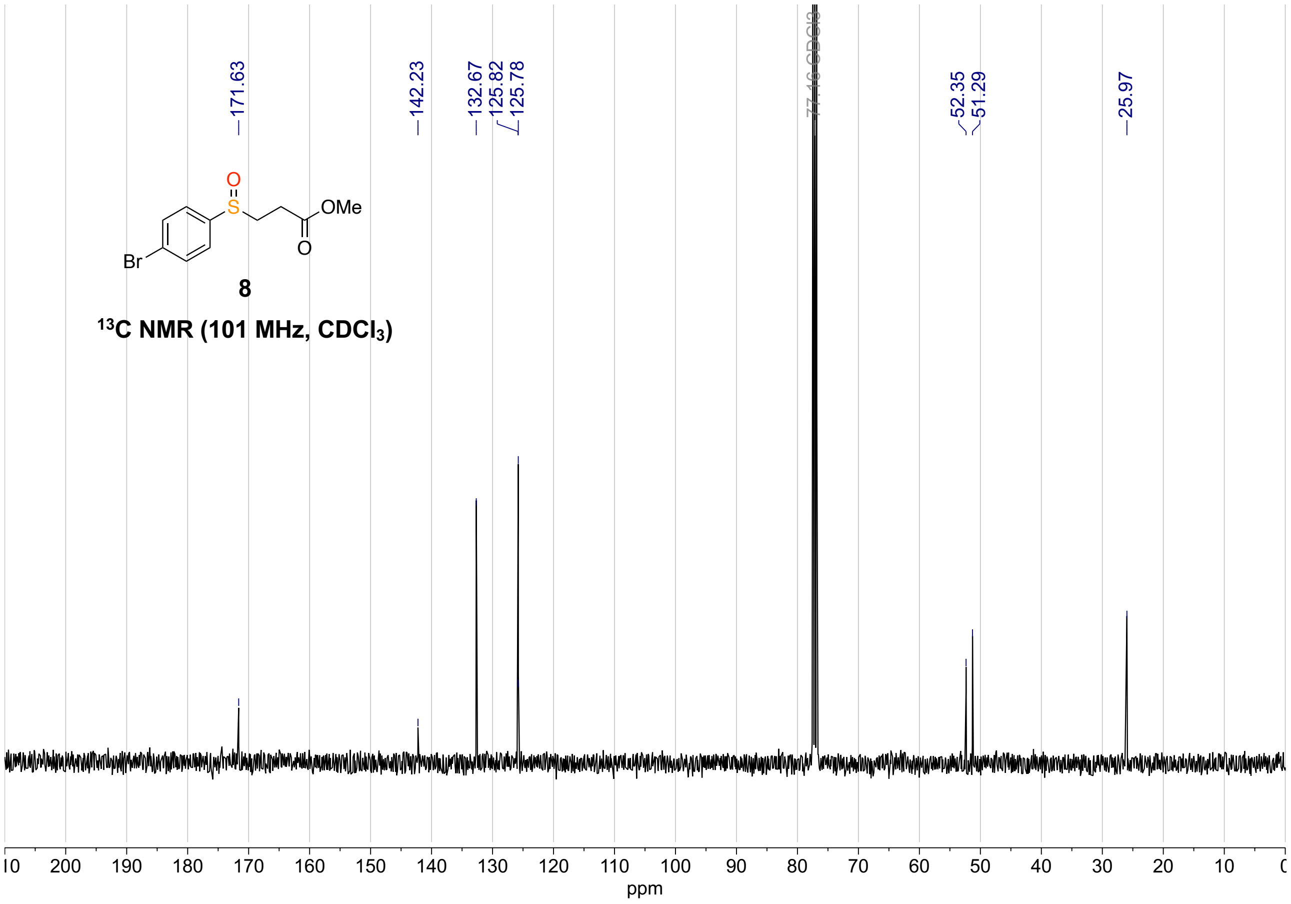

Methyl 3-(4-bromo-*N*-(*tert*-butoxycarbonyl)phenylsulfonimidoyl)propanoate ((*S*)-9)

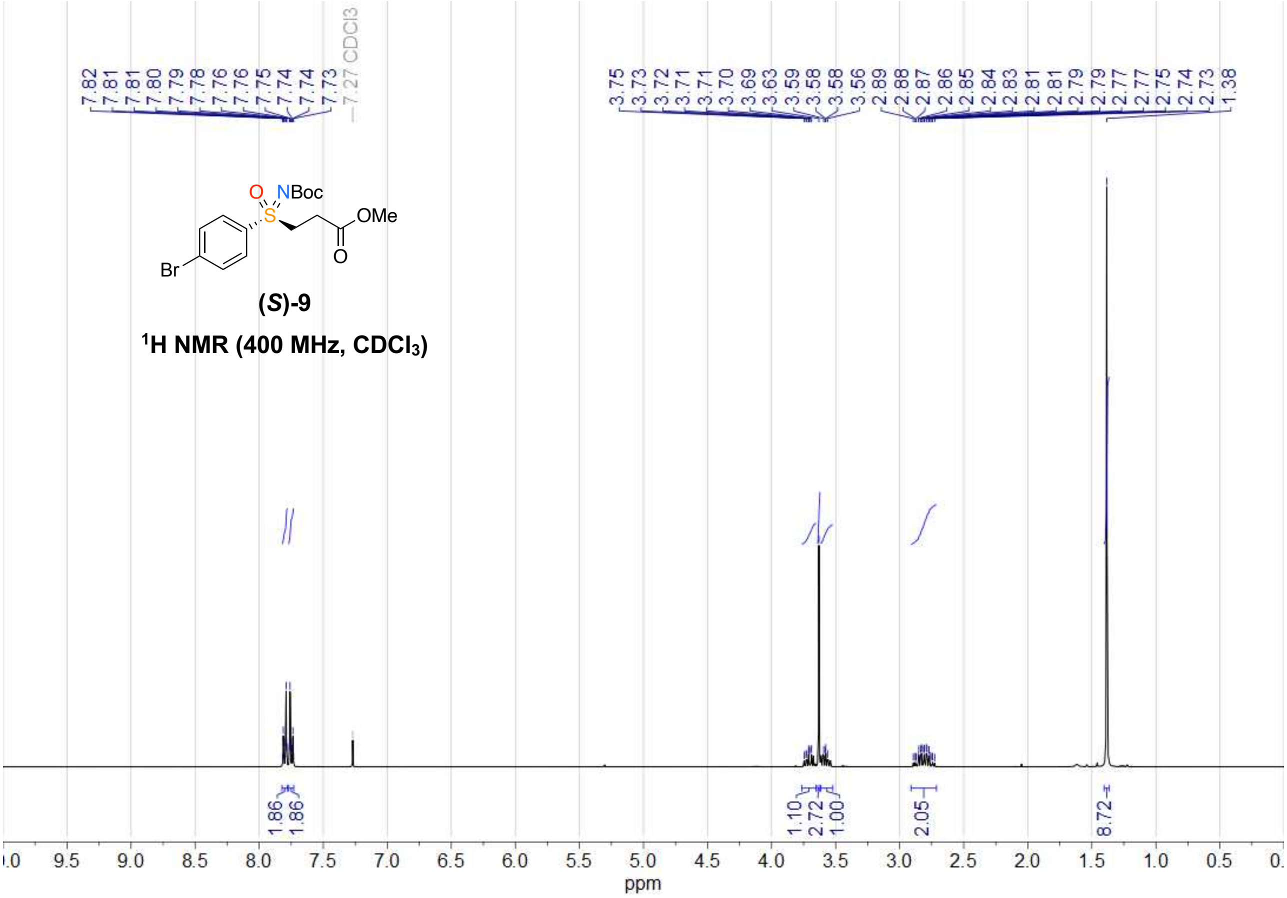

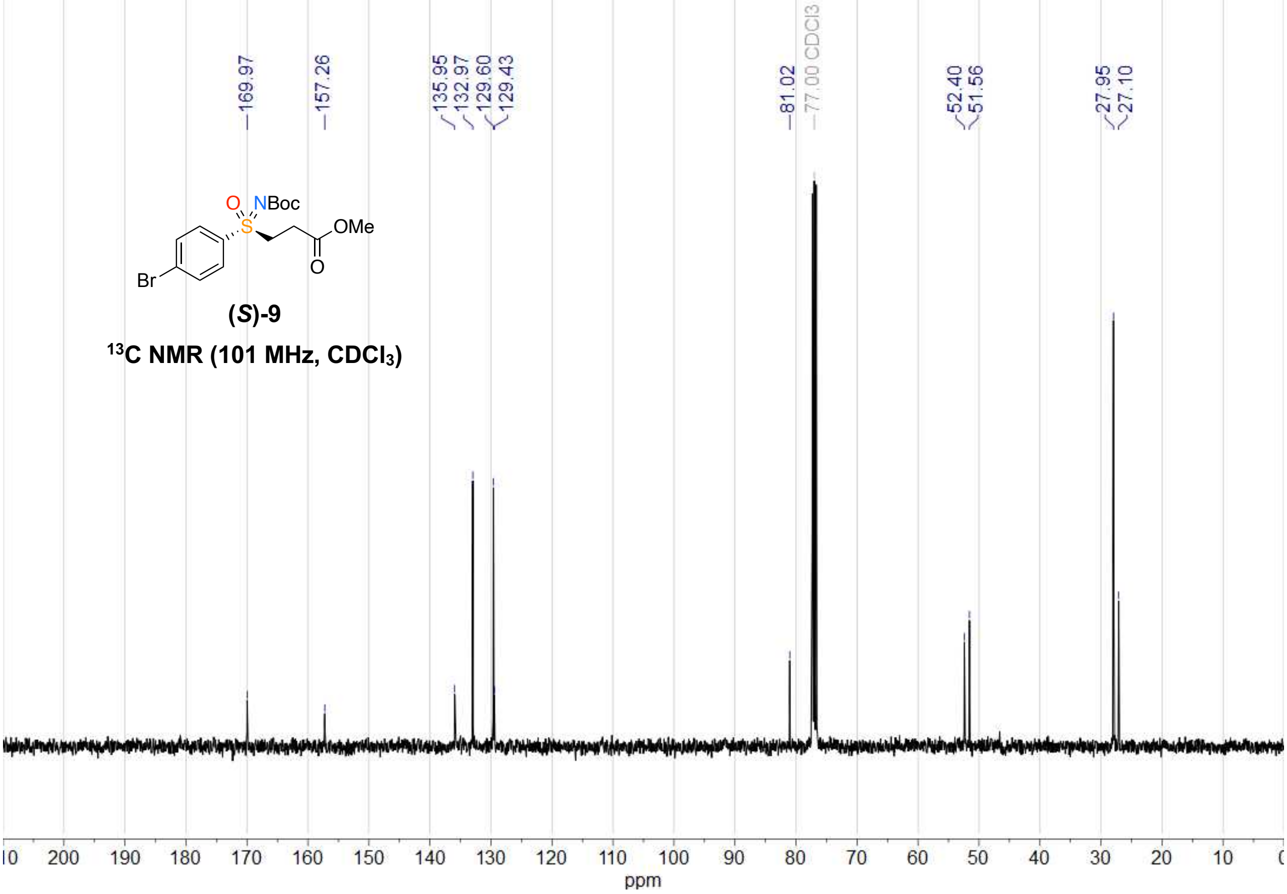

Sodium ((4-bromophenyl)sulfinyl)(*tert*-butoxycarbonyl)amide ((*S*)-1b)

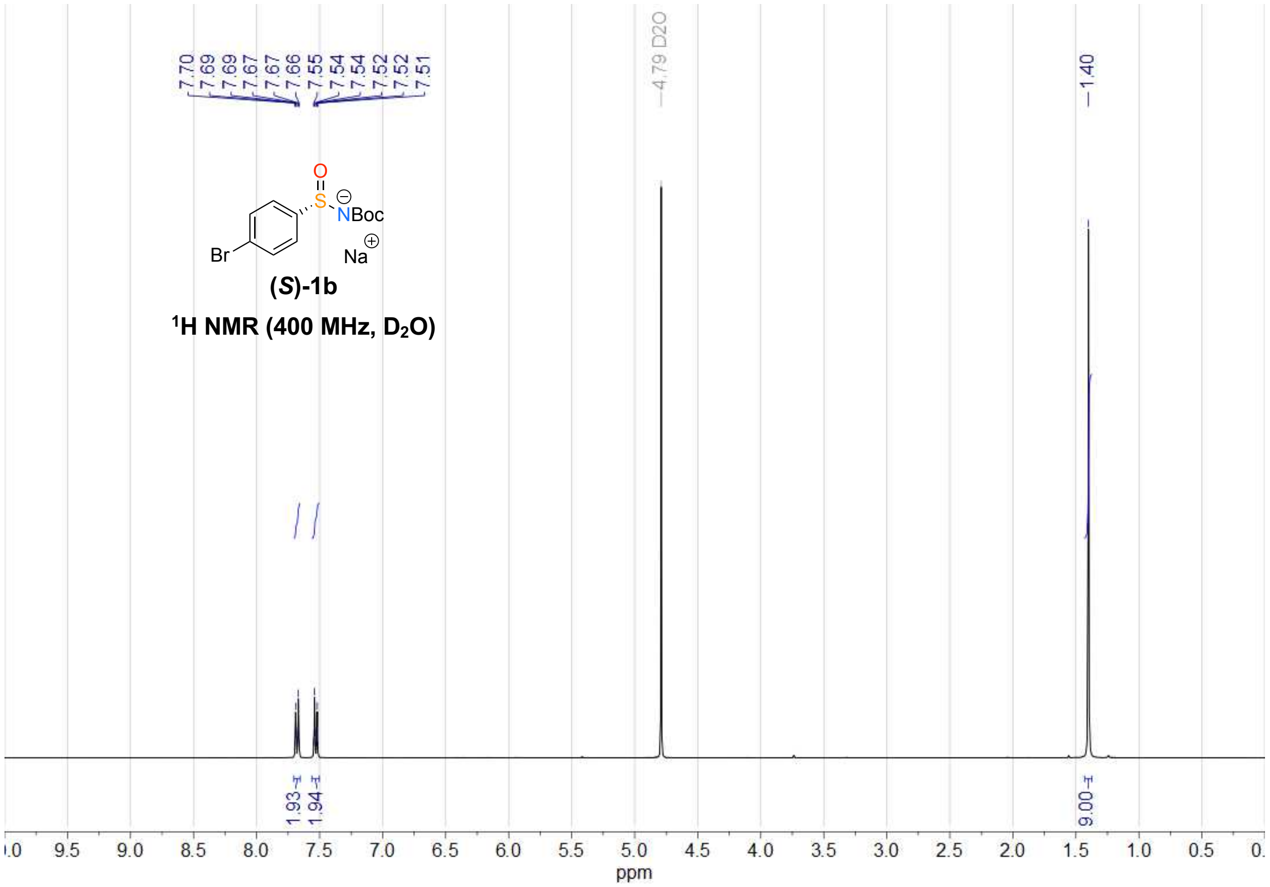

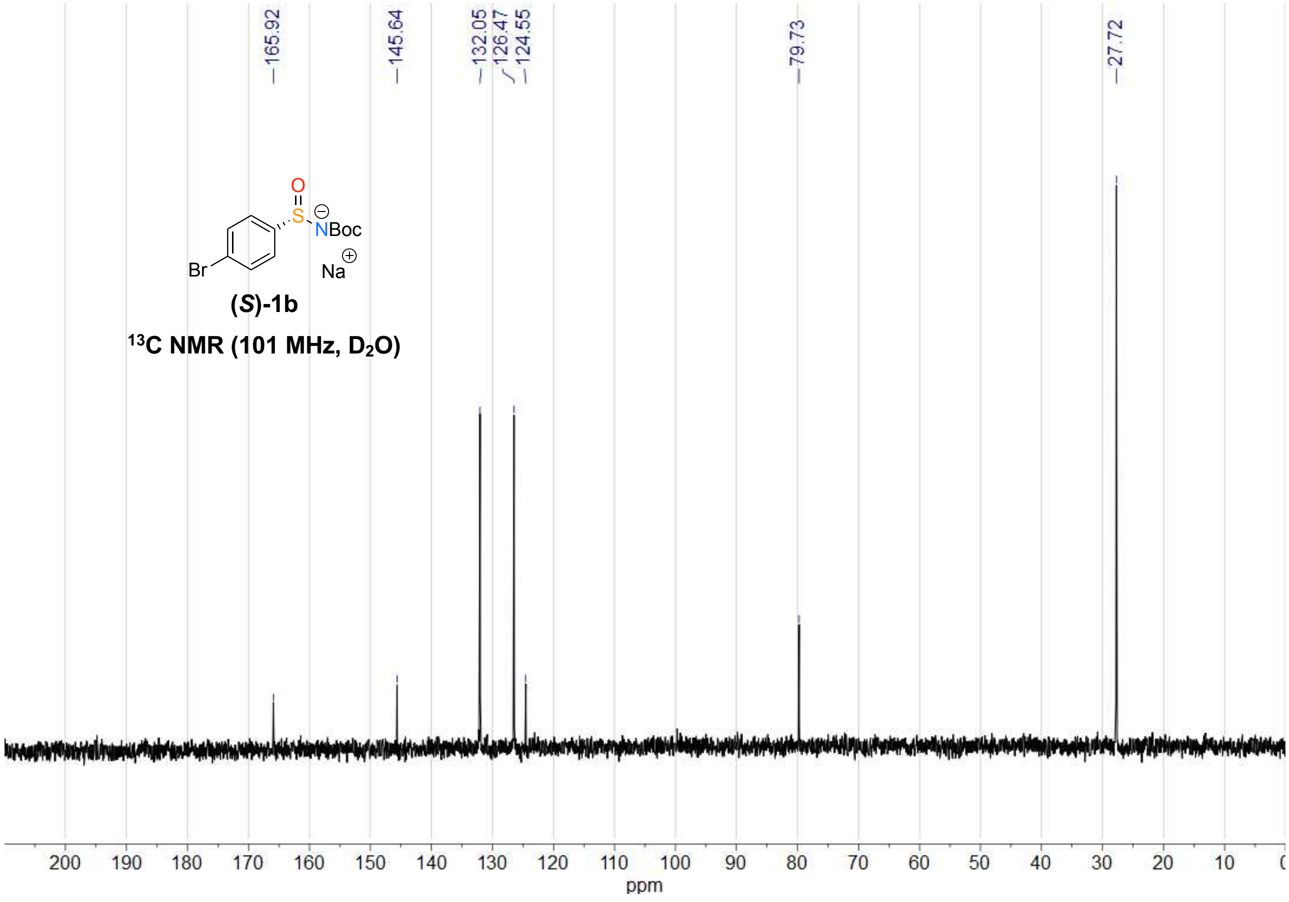

tert-Butyl ((4-bromophenyl)fluoro(oxo)-λ^6-sulfaneylidene)carbamate ((*R*)-2b)

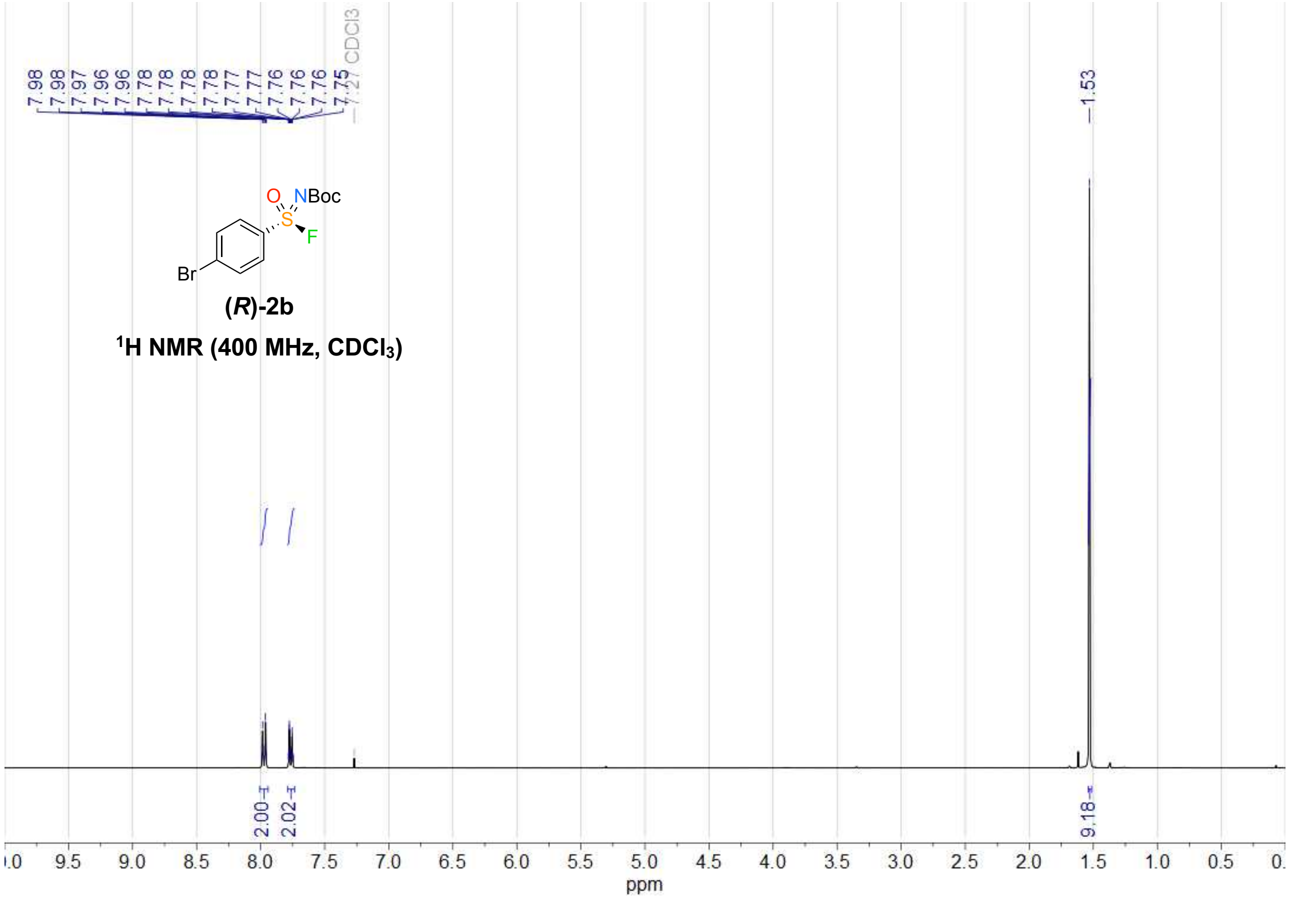

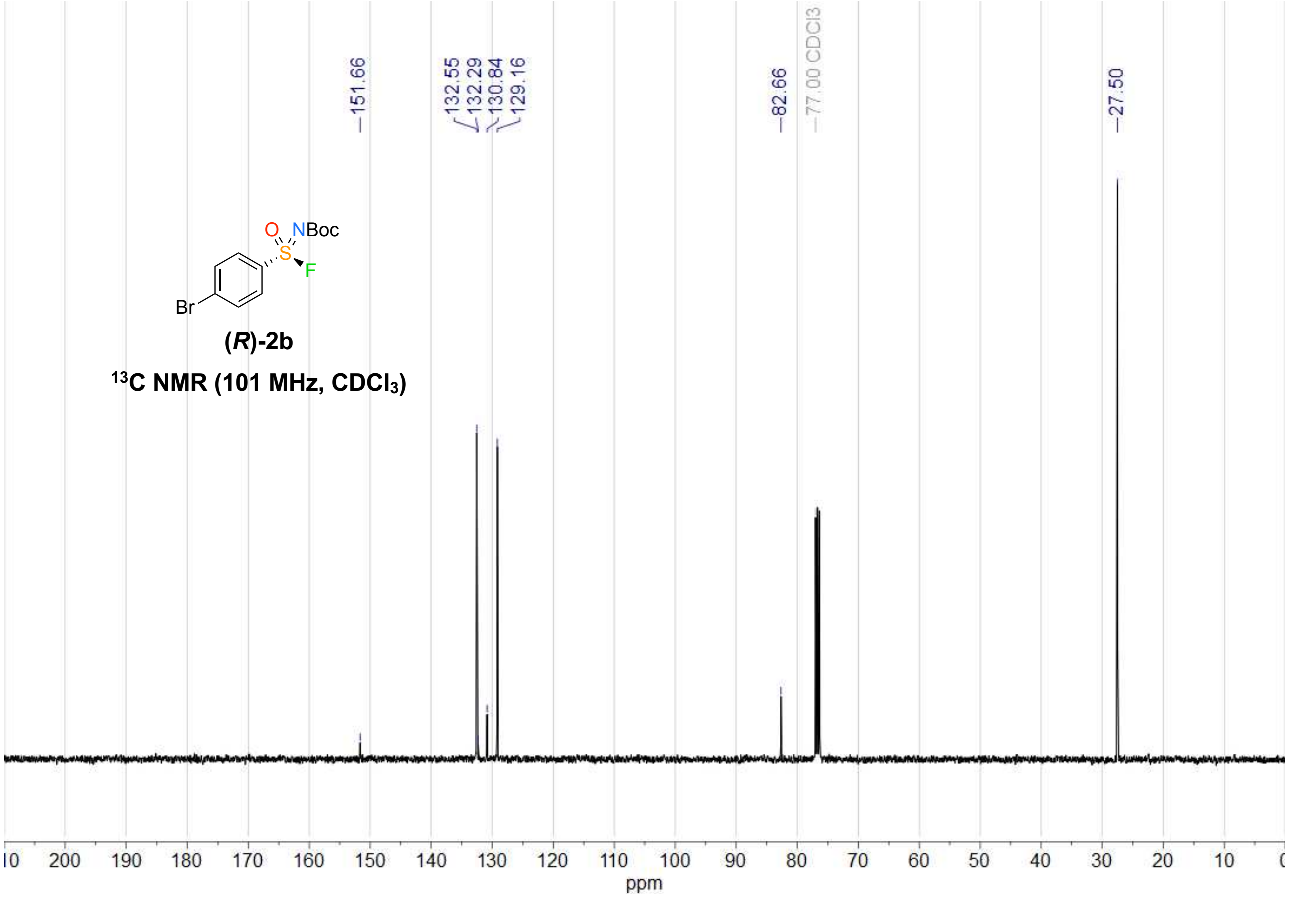

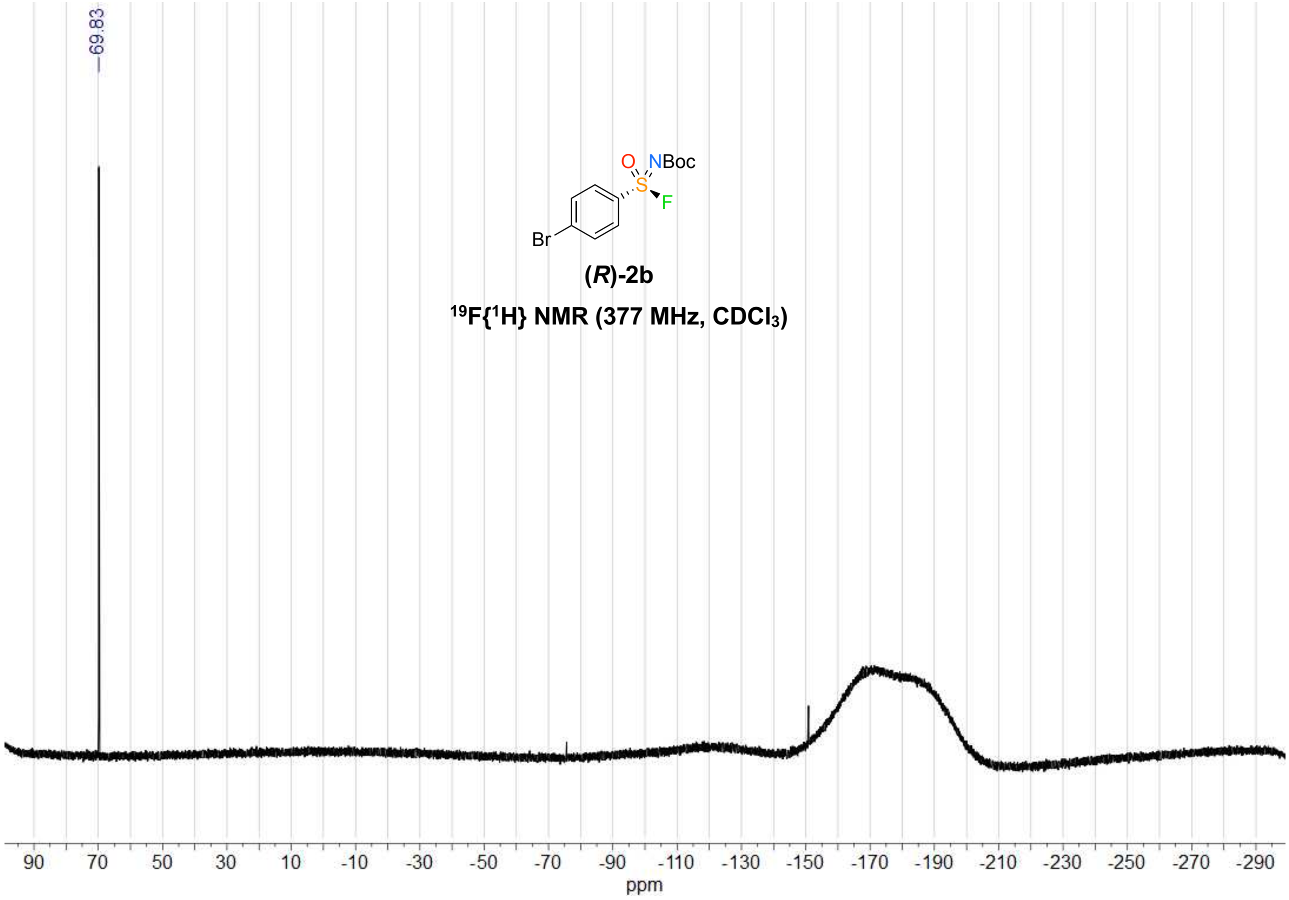

69.83
O NBoc
S
F
Br
(*R*)-2b
$^{19}F\{^{1}H\}$ NMR (377 MHz, $CDCl_3$)
90 70 50 30 10 -10 -30 -50 -70 -90 -110 -130 -150 -170 -190 -210 -230 -250 -270 -290
ppm

tert-Butyl (R)-((benzylamino)(4-bromophenyl)(oxo)-λ⁶-sulfaneylidene)carbamate ((R)-3v)

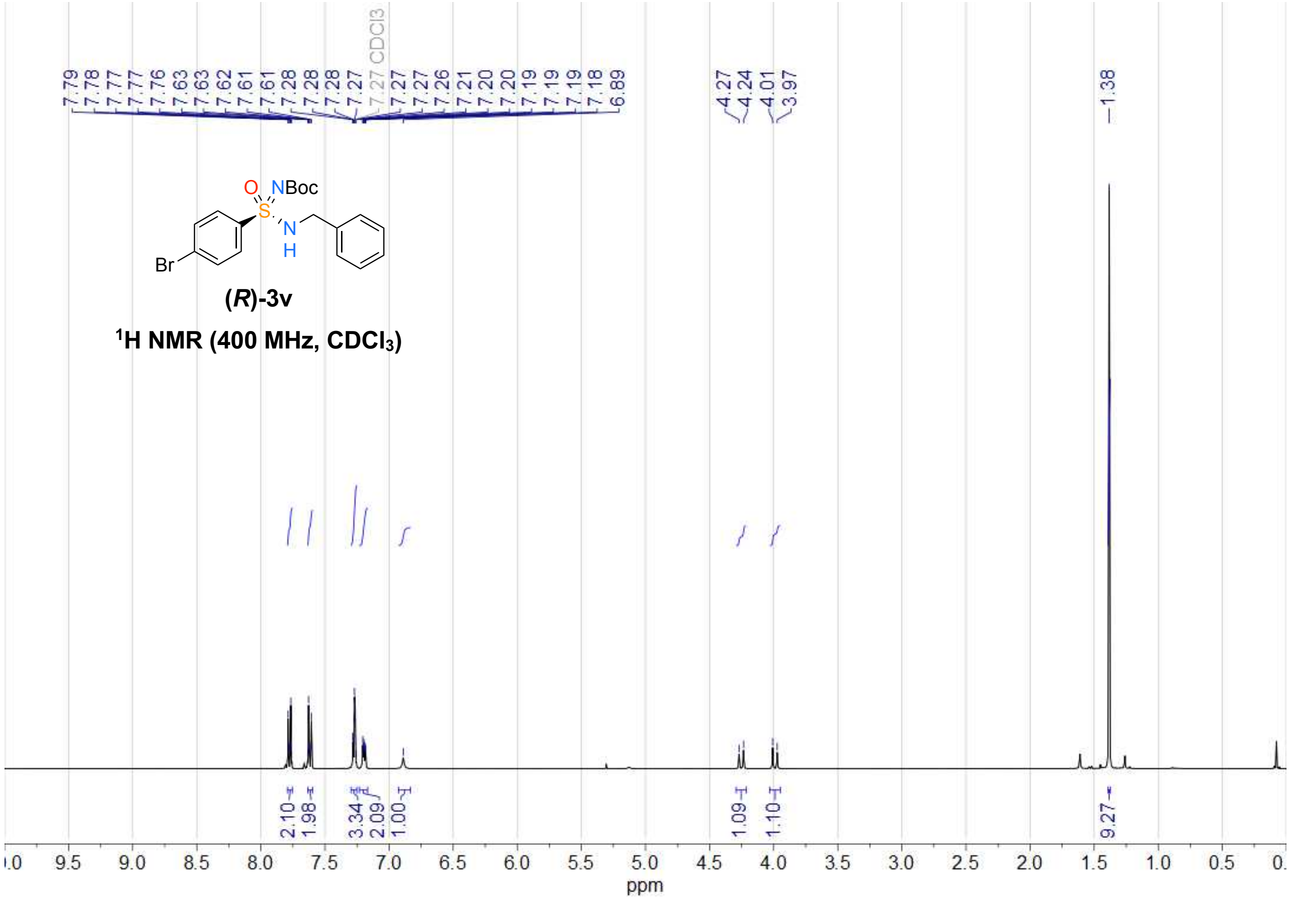

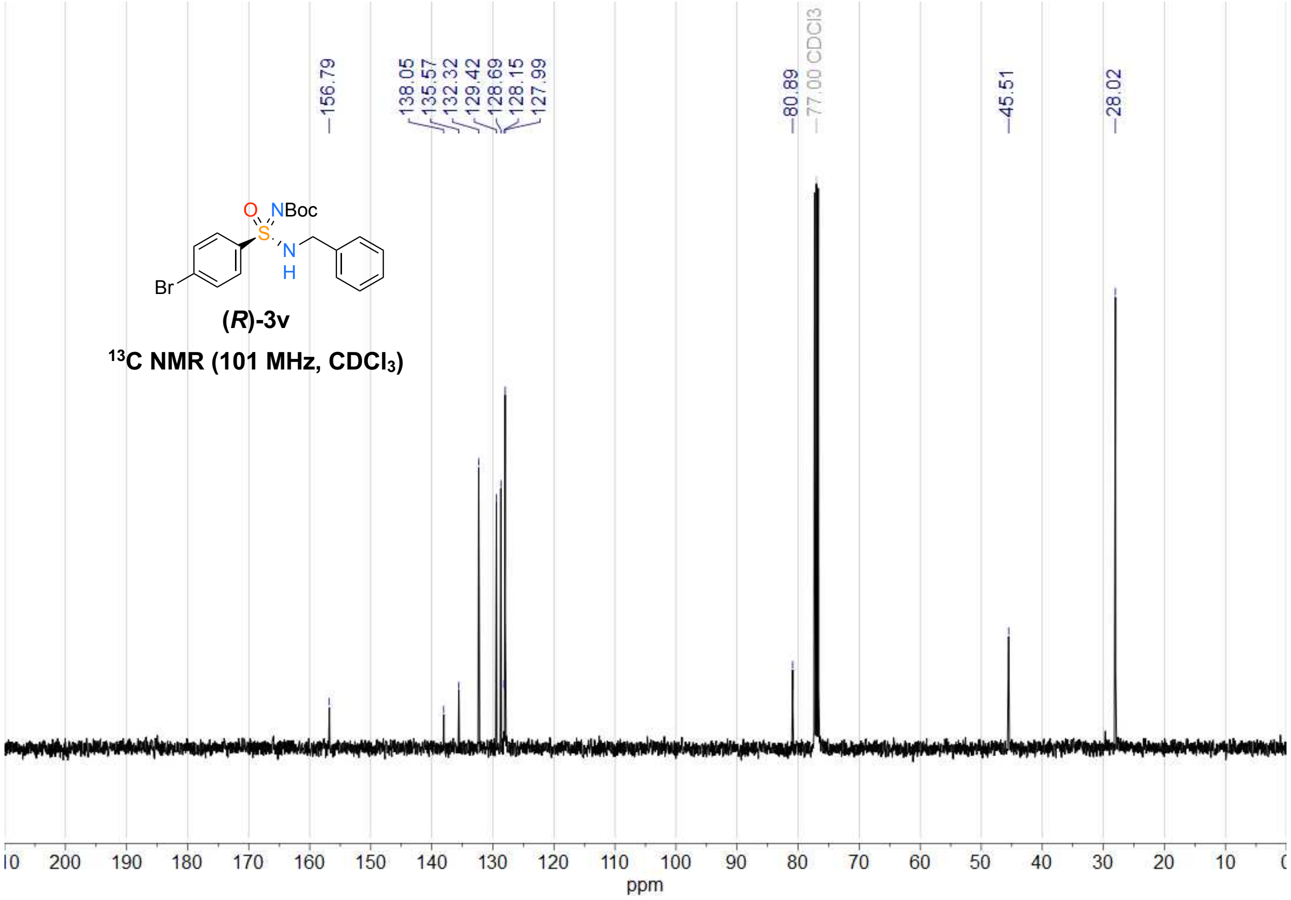

tert-Butyl (R)-((allylamino)(4-bromophenyl)(oxo)-λ^6-sulfaneylidene)carbamate ((R)-3w)

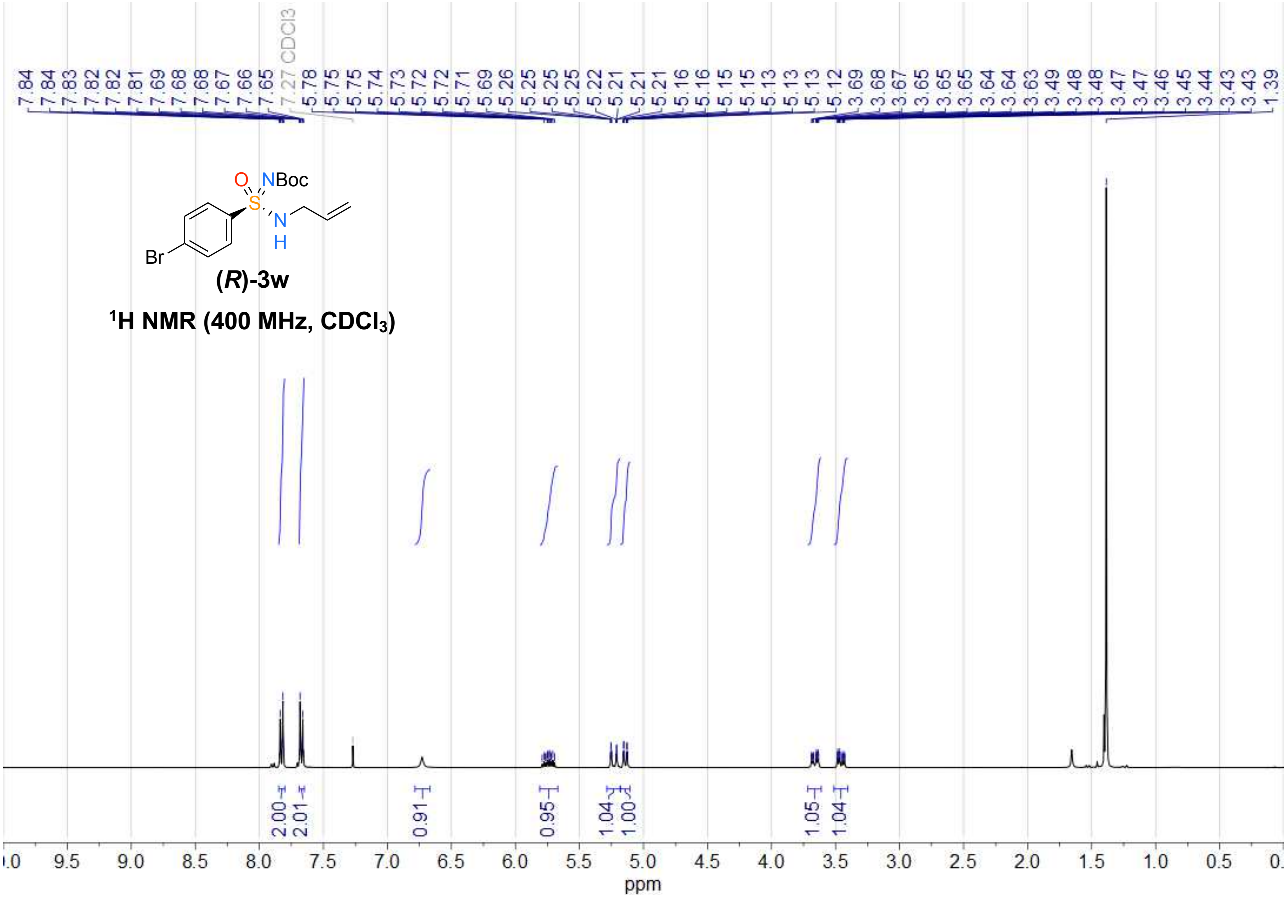

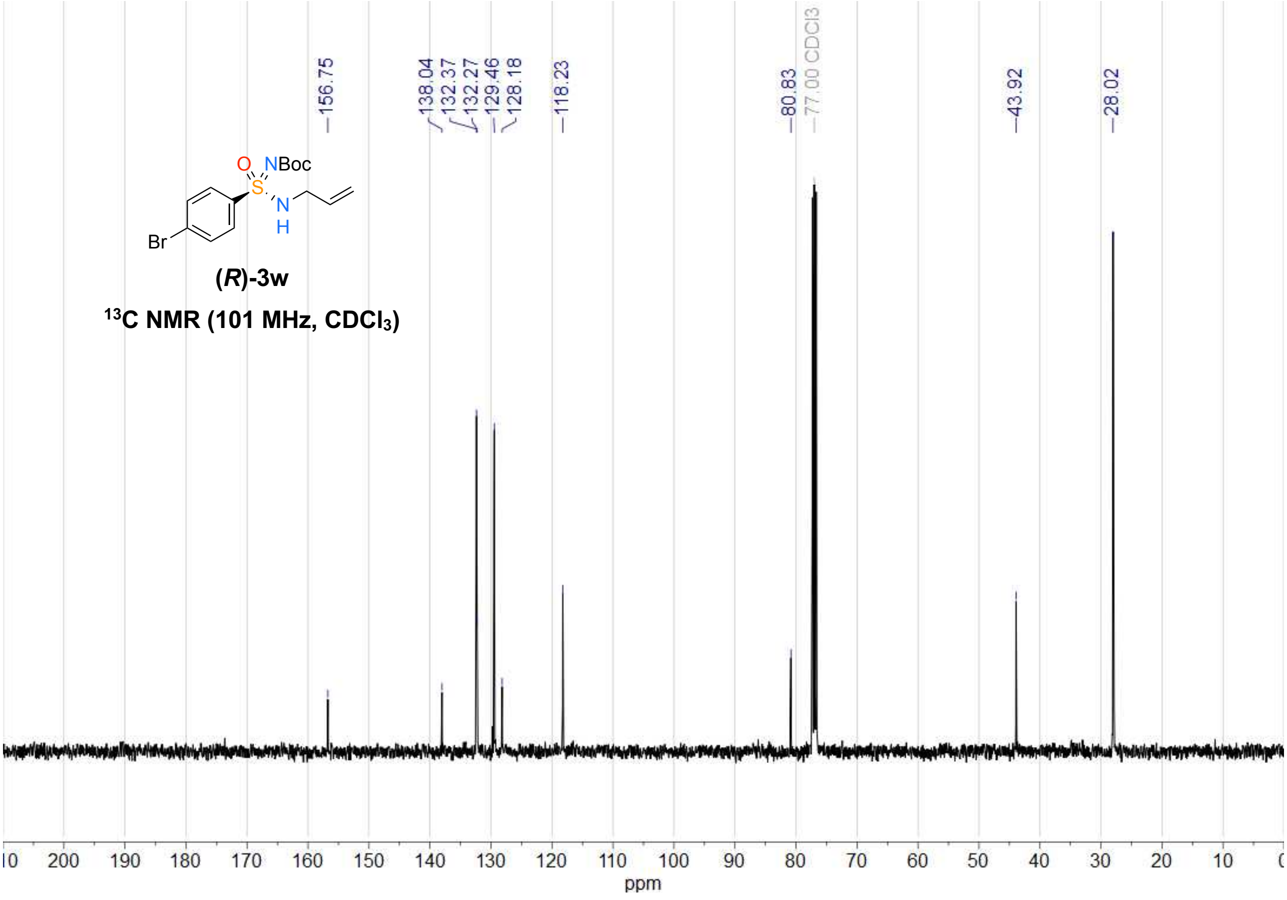

***tert*-Butyl ((4-bromophenyl)(oxo)(piperidin-1-yl)-λ^6-sulfaneylidene)carbamate ((*R*)-3x)**

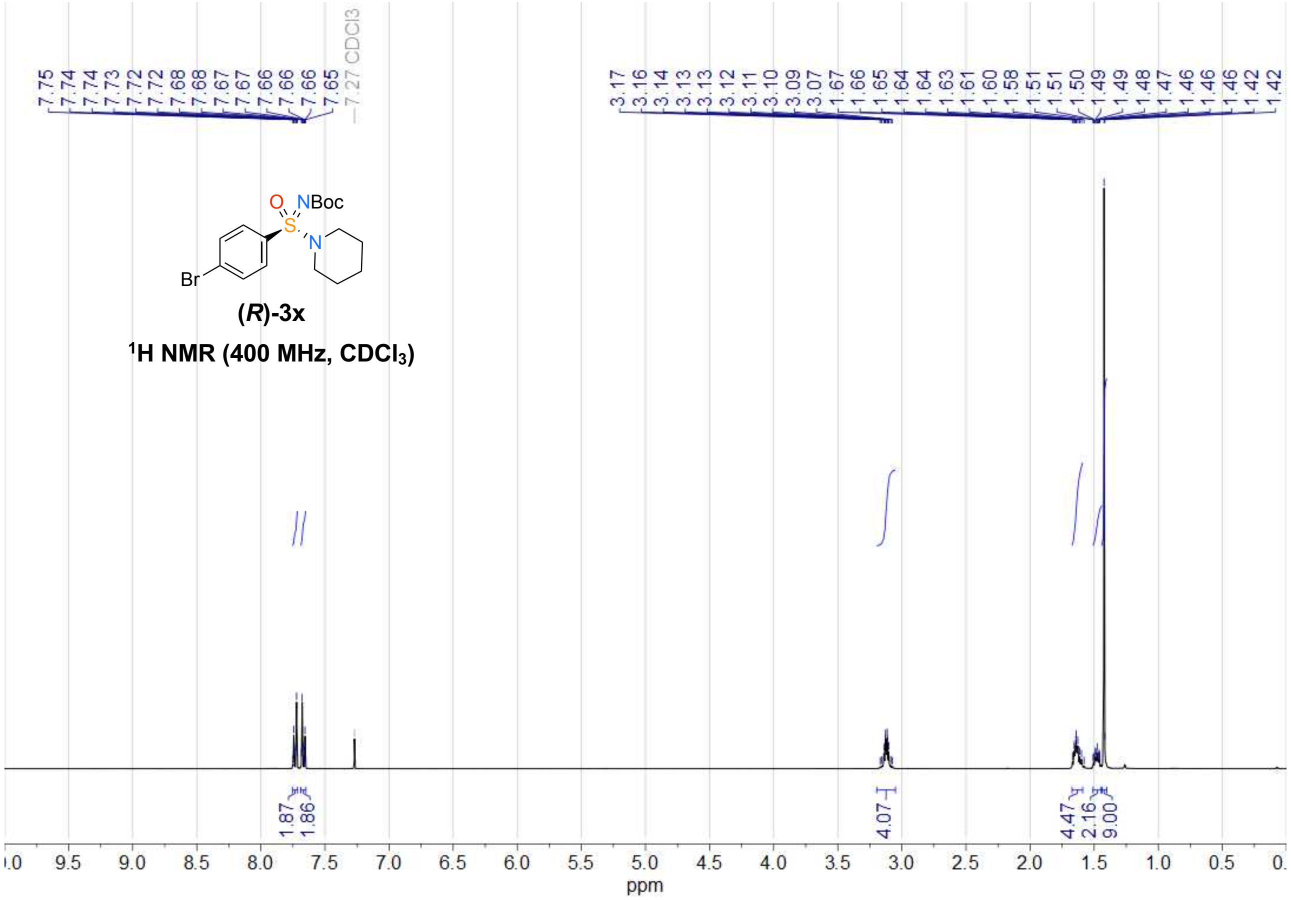

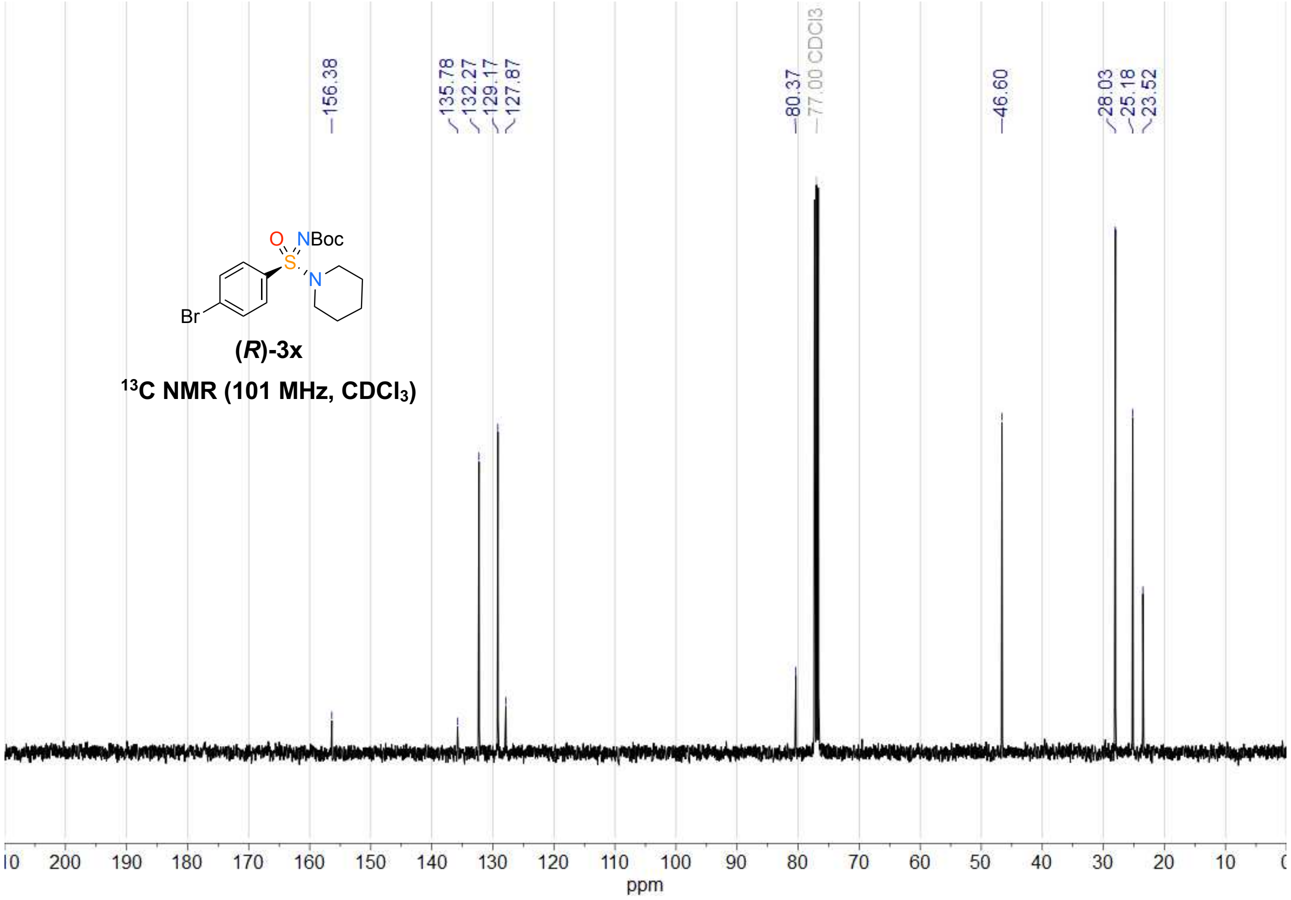

tert-Butyl ((R)-(4-bromophenyl)(methyl((S)-3-(naphthalen-1-yloxy)-3-(thiophen-2-yl)propyl)amino)(oxo)-λ⁶-sulfaneylidene)carbamate ((R)-3y)

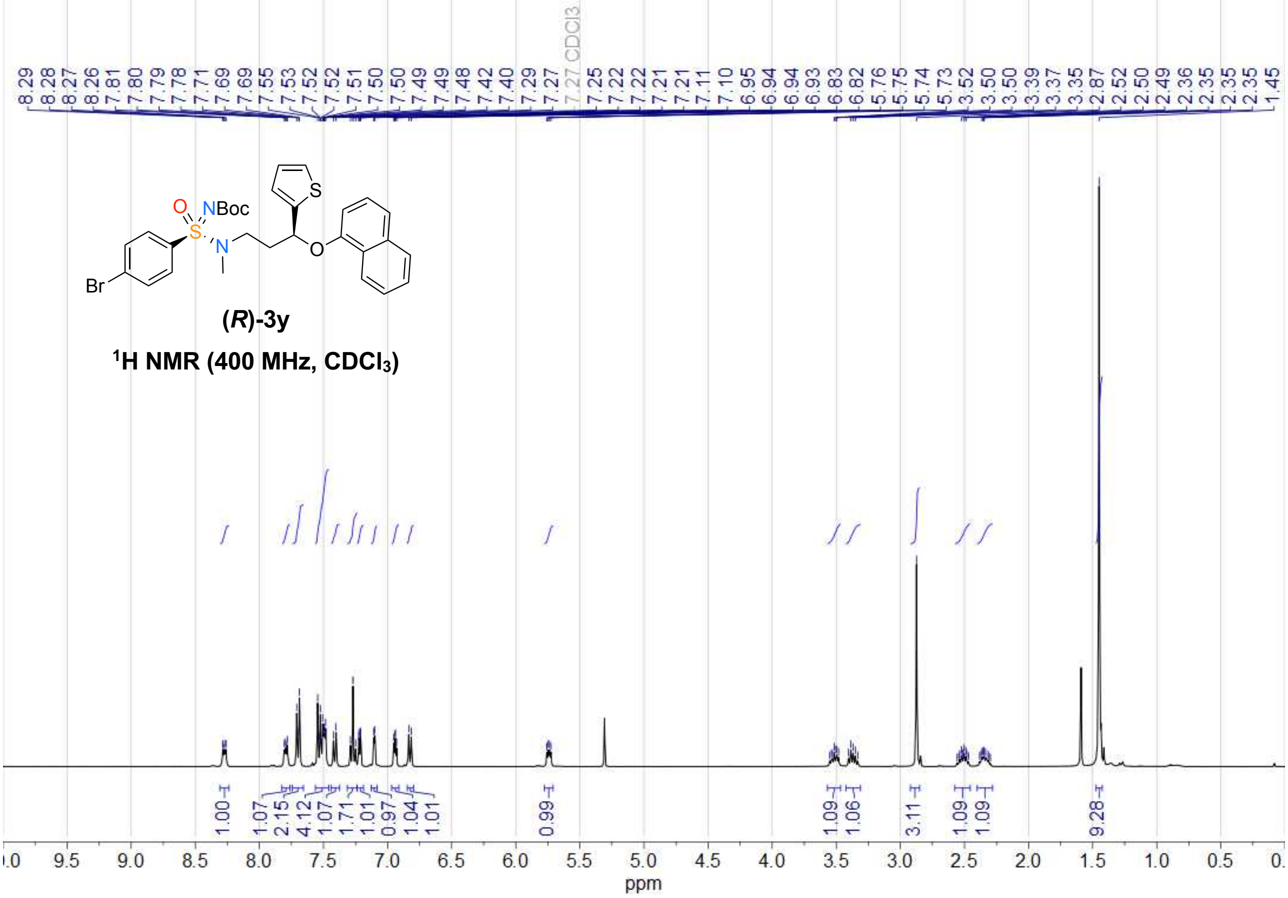

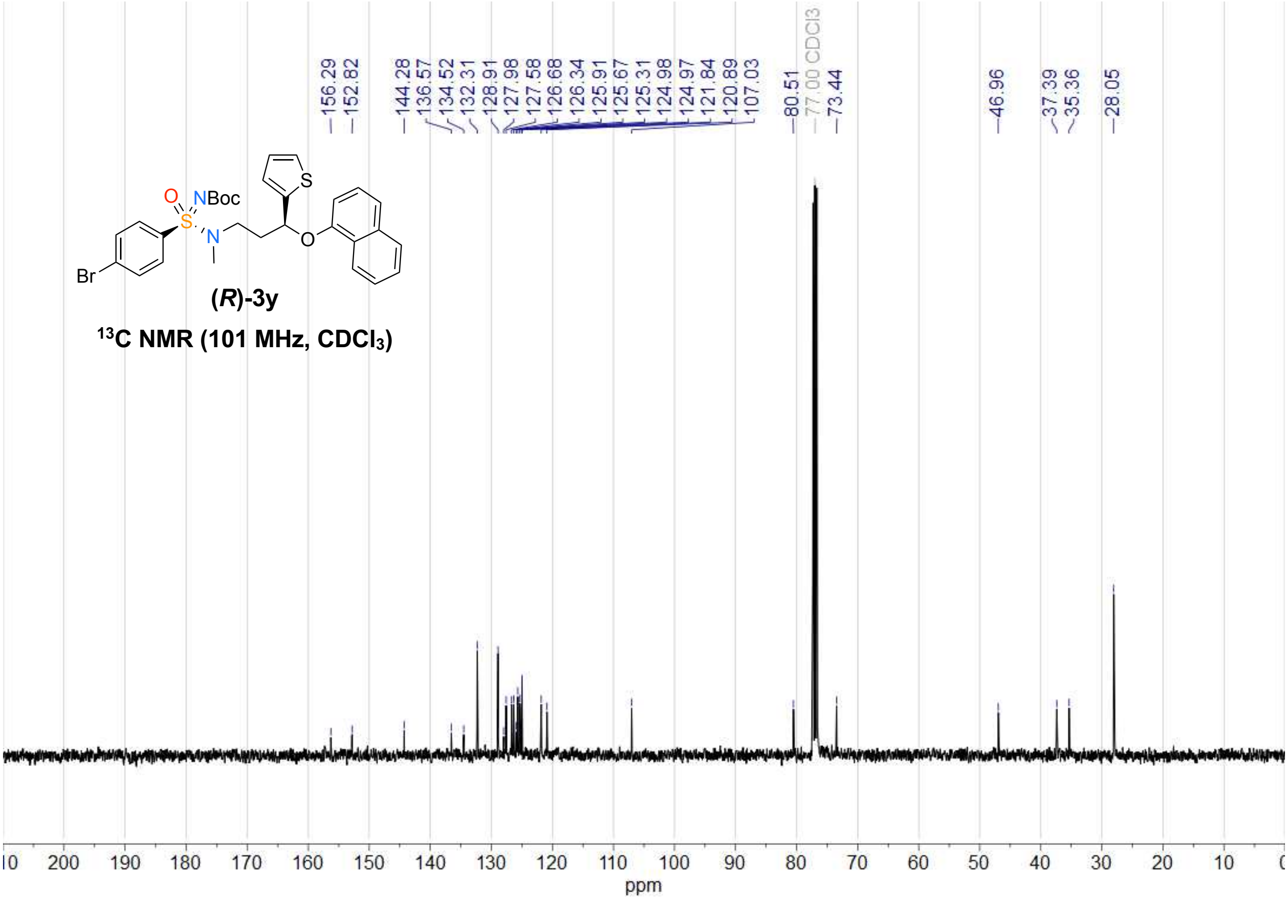

tert-Butyl (R)-([1,1'-biphenyl]-4-yl(oxo)(piperidin-1-yl)-λ6-sulfaneylidene)carbamate ((R)-10)

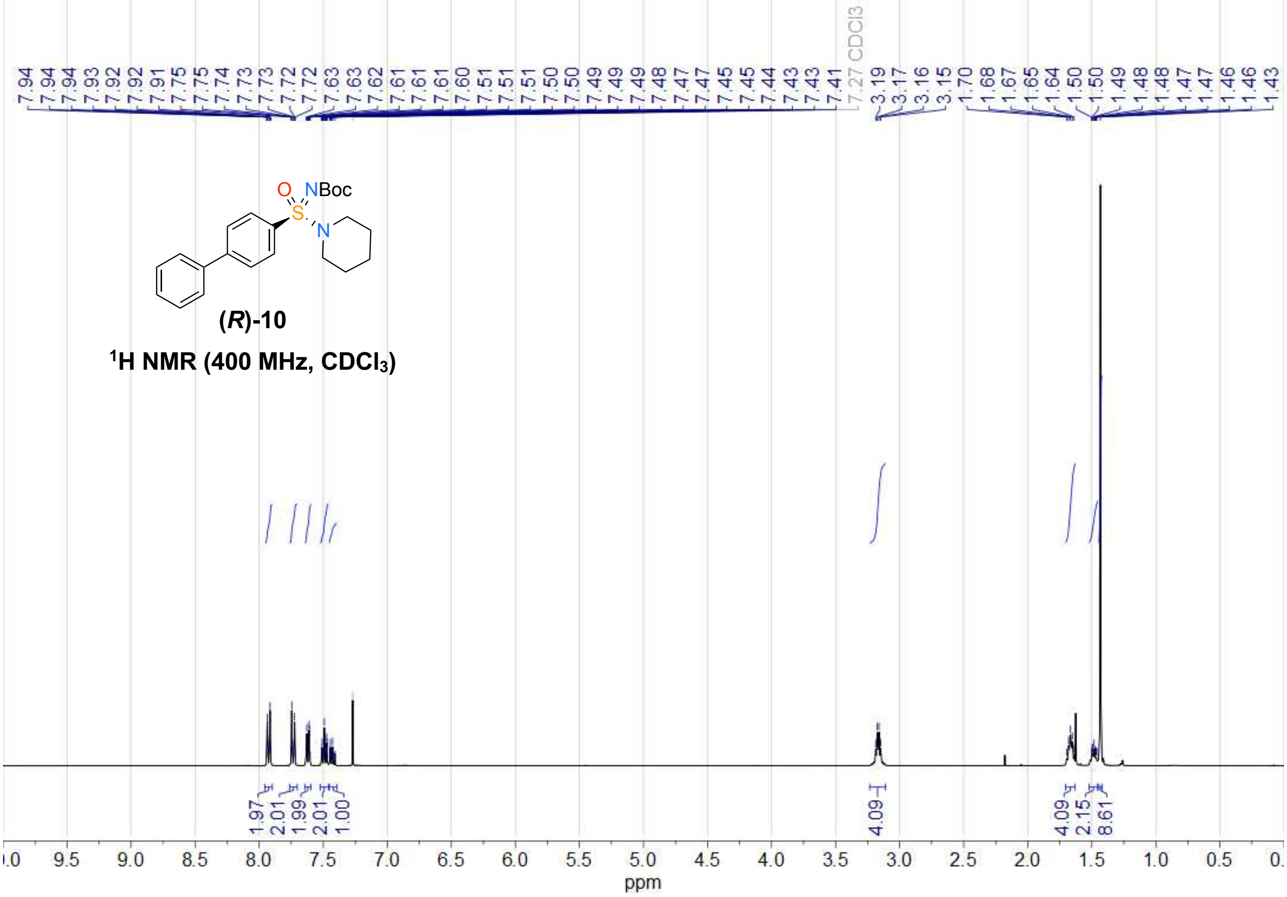

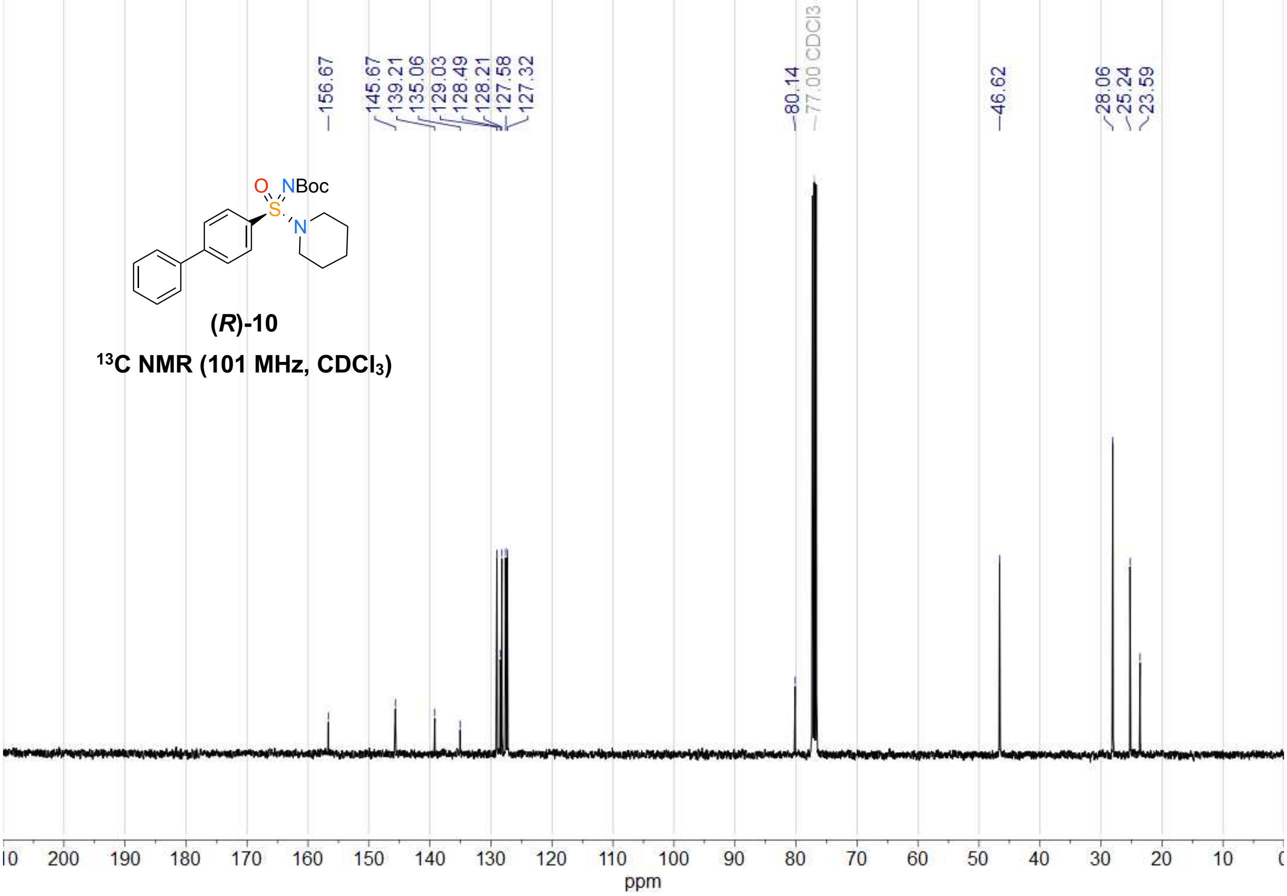

Methyl 3-(tolylthio)propanoate (S1a)

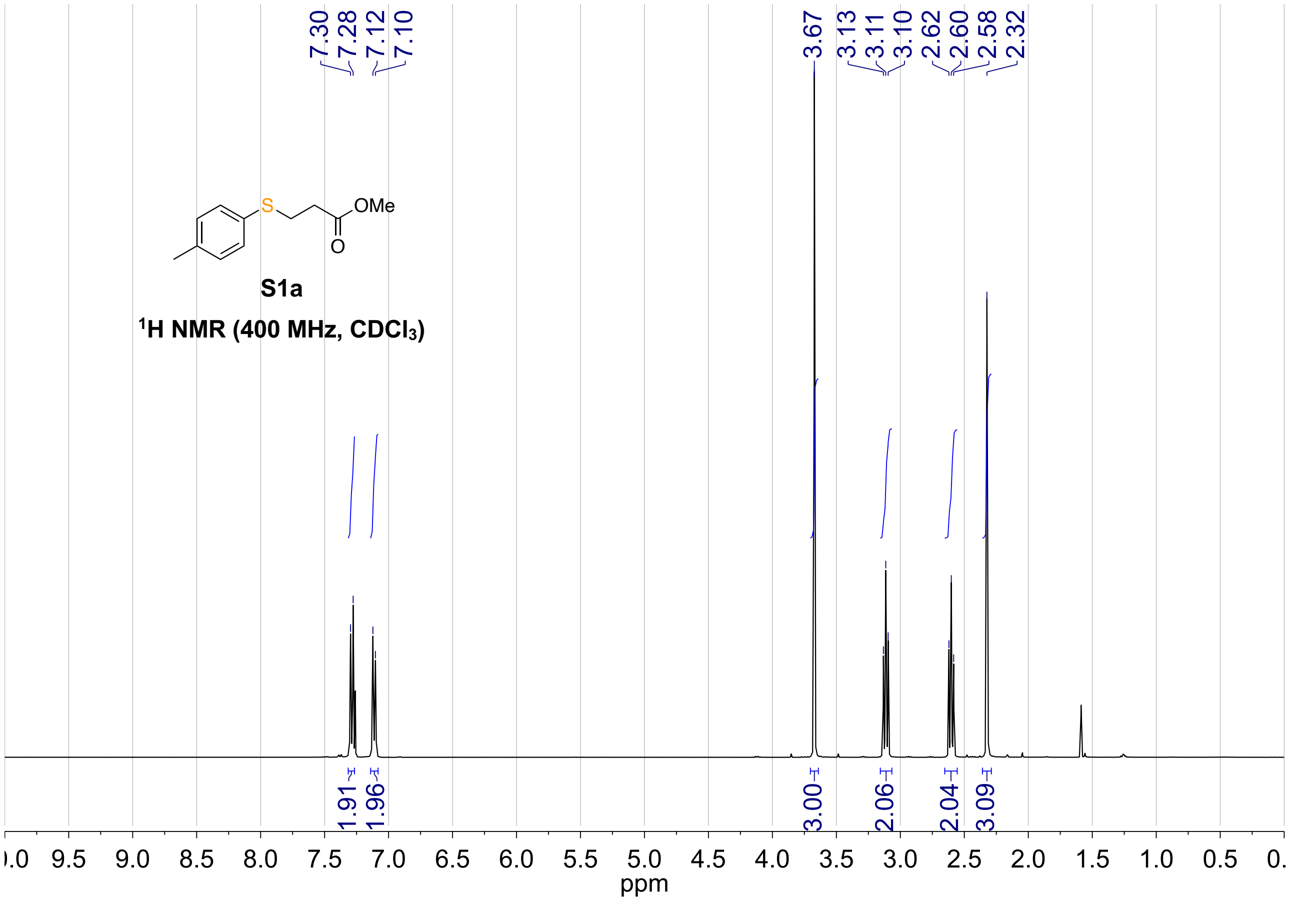

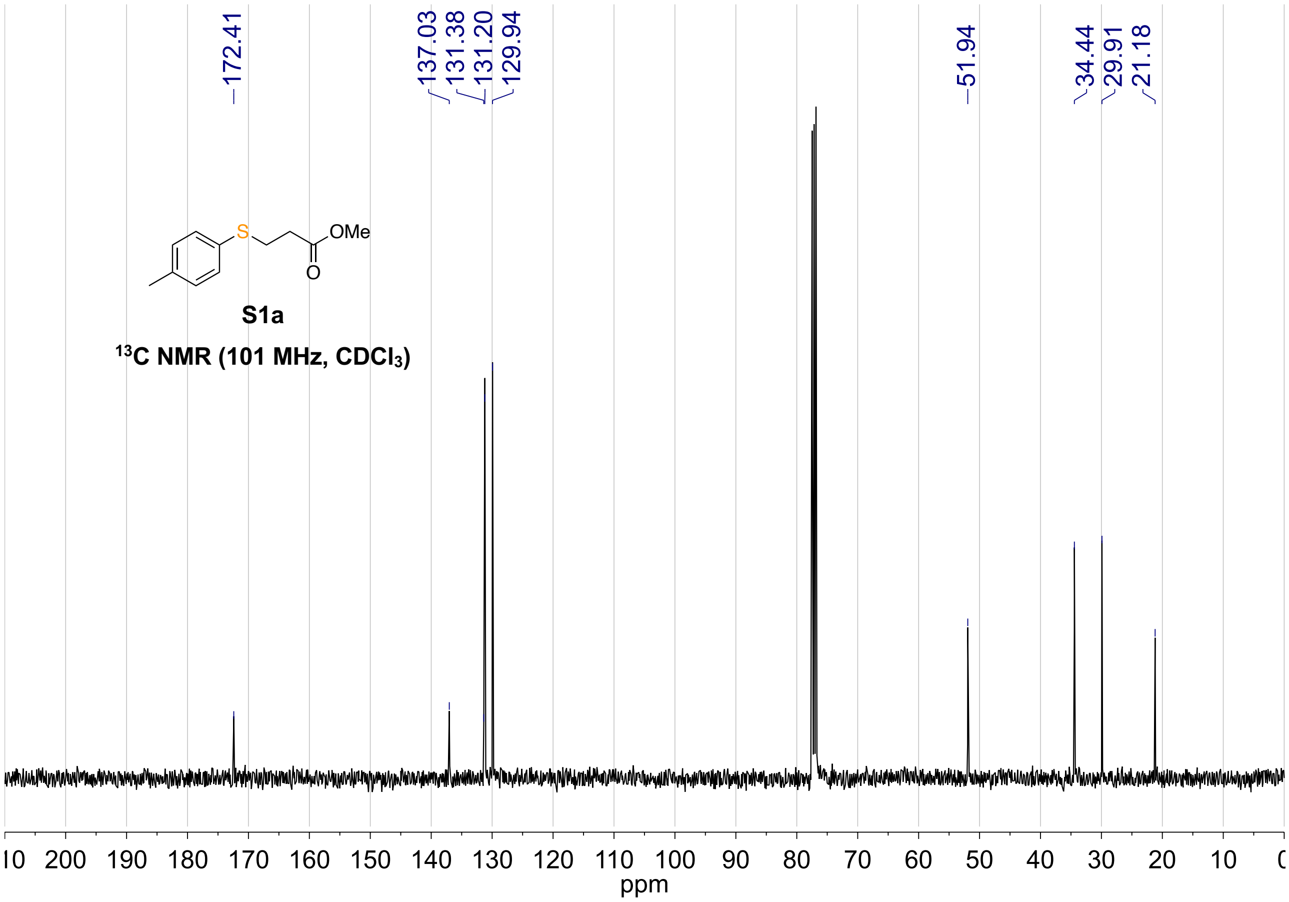

Methyl 3-(phenylthio)propanoate (S1b)

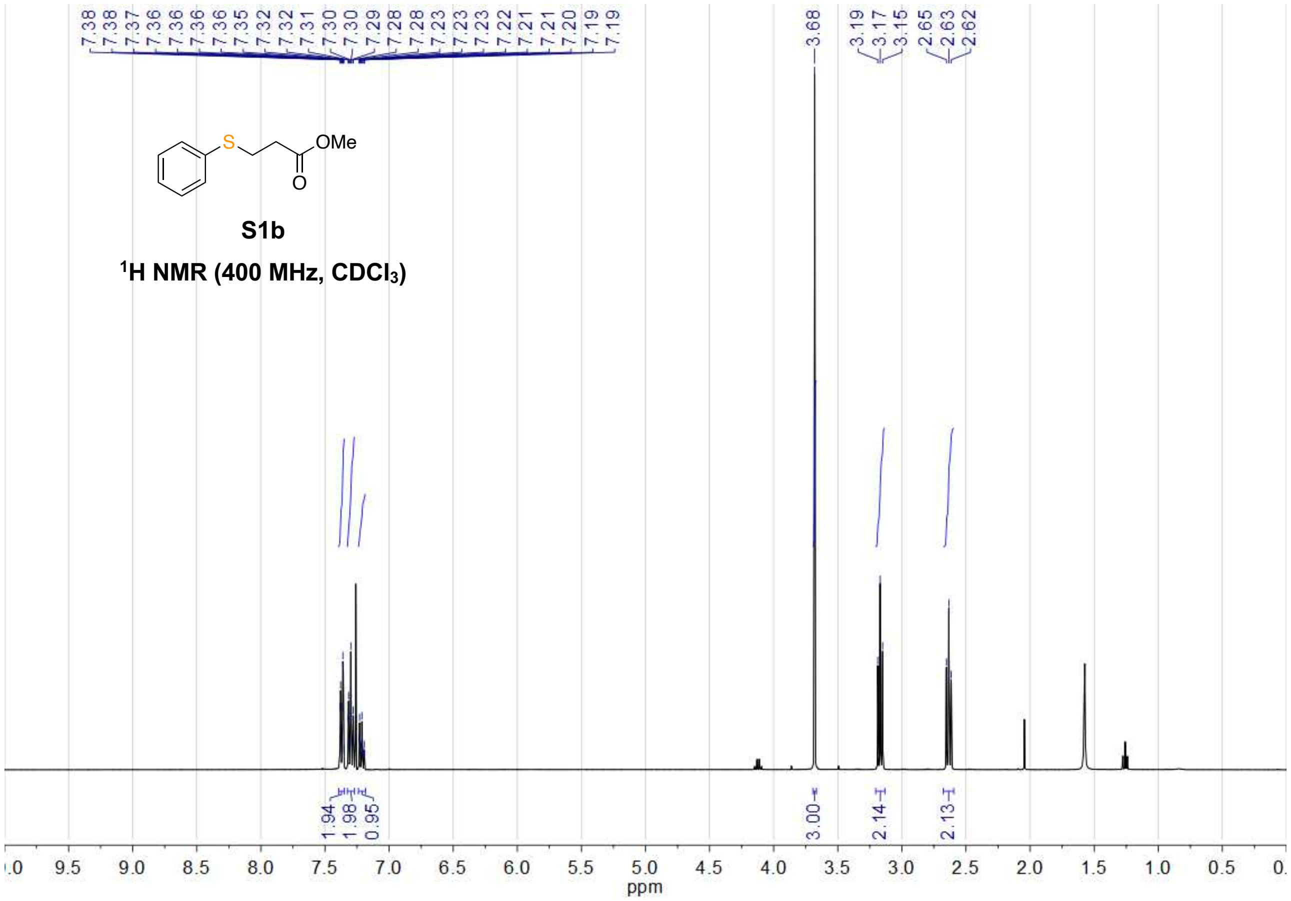

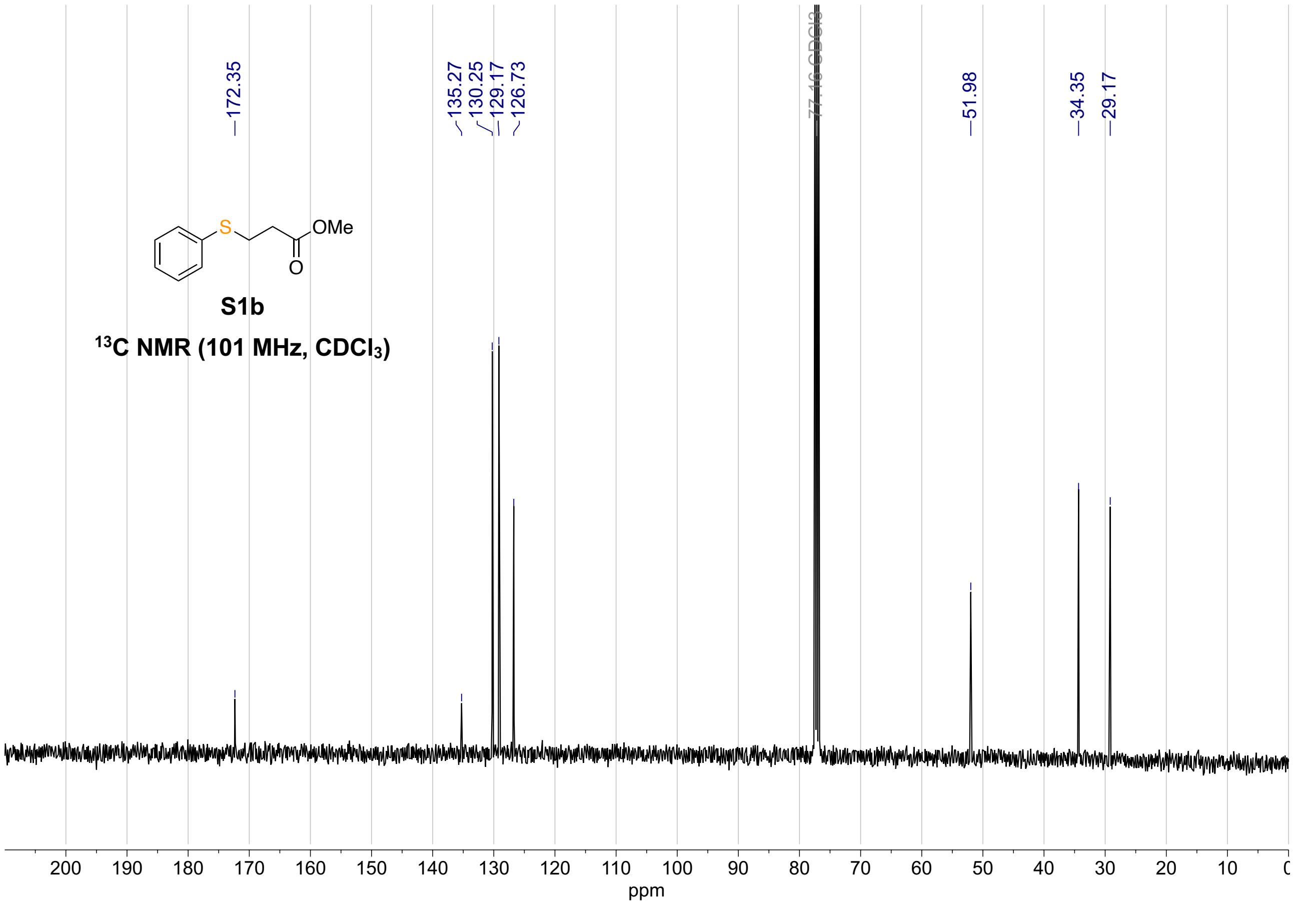

Methyl 3-((4-fluorophenyl)thio)propanoate (S1c)

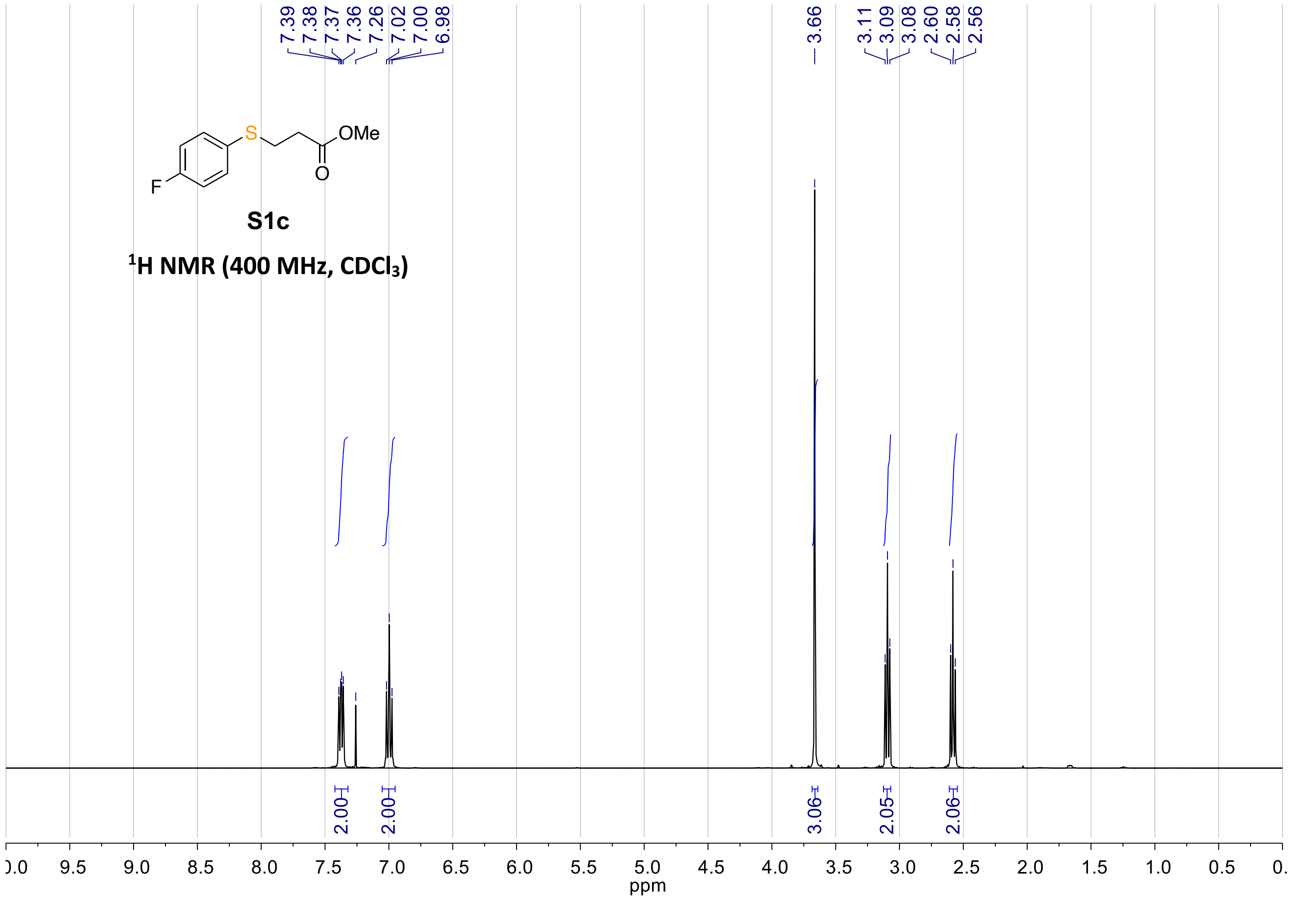

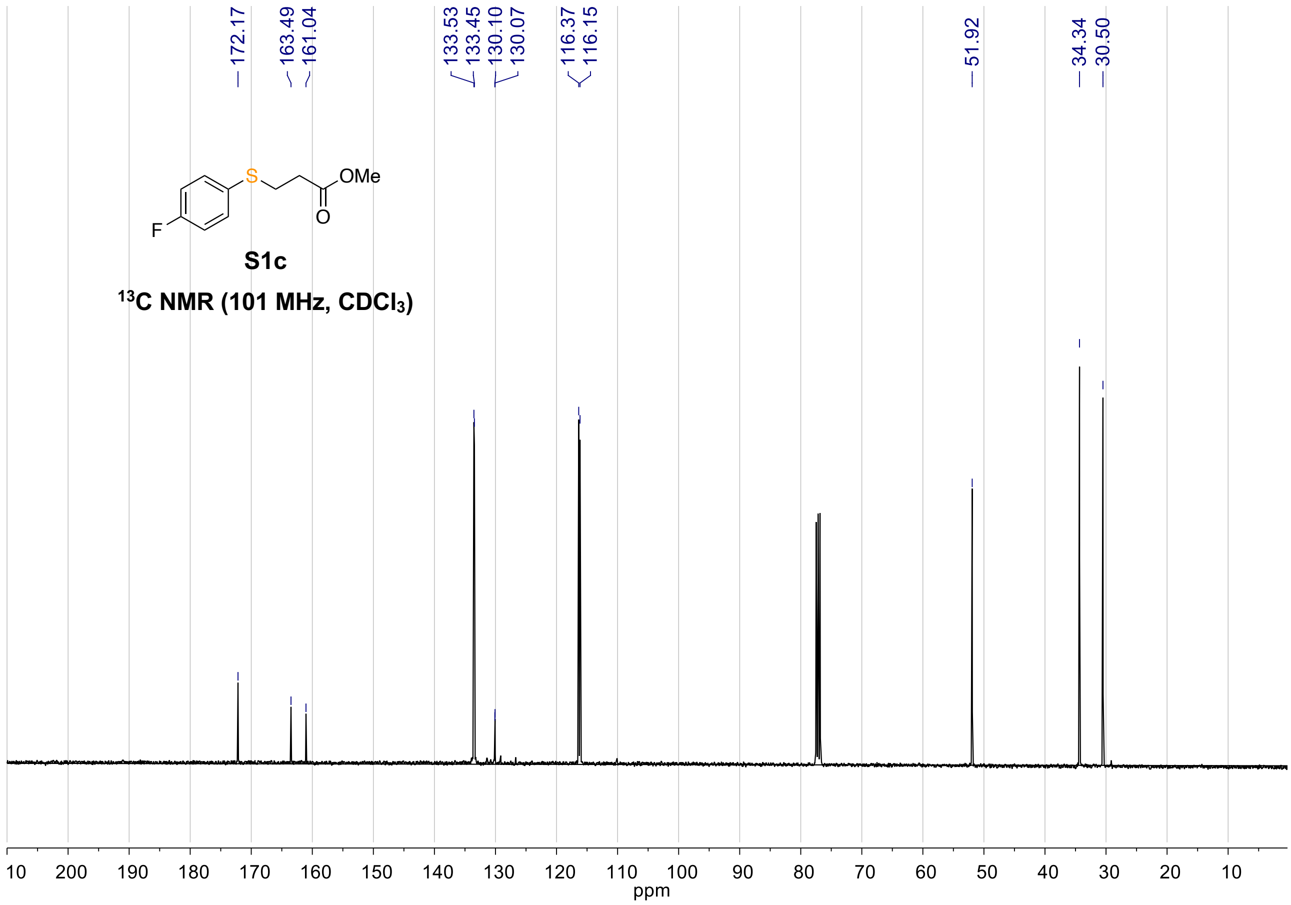

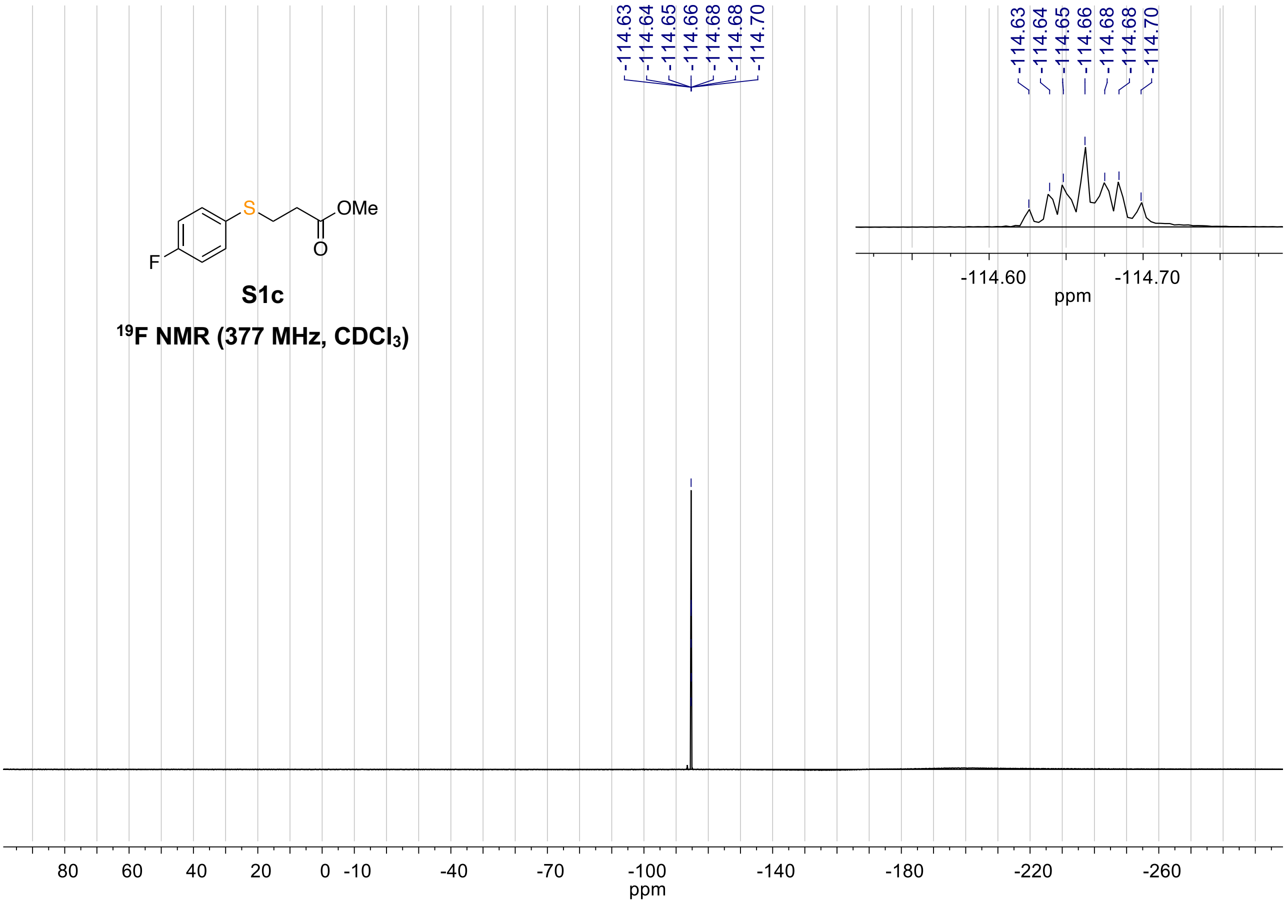

S1c

^{19}F NMR (377 MHz, $CDCl_3$)

Methyl 3-((4-methoxyphenyl)thio)propanoate (S1d)

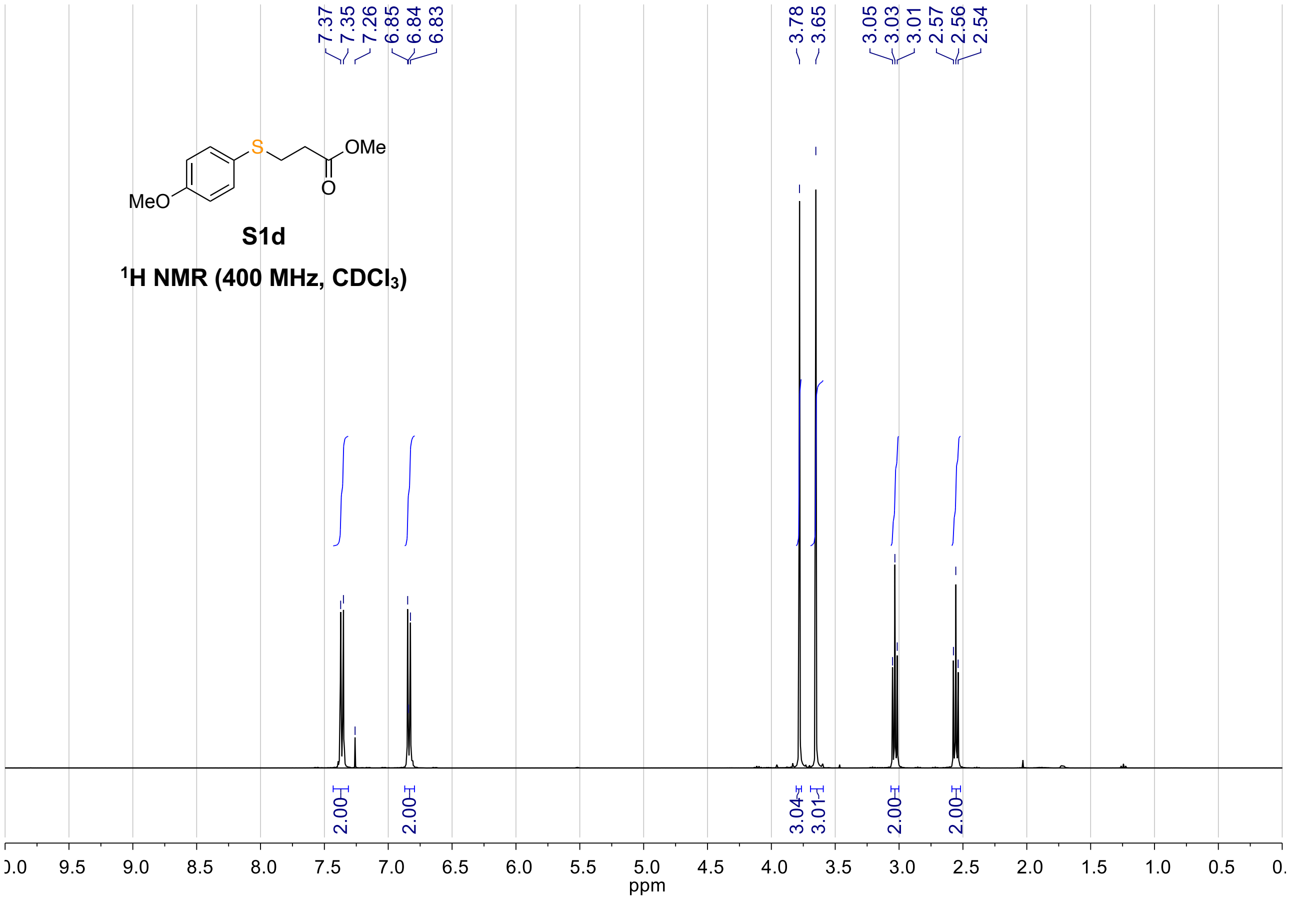

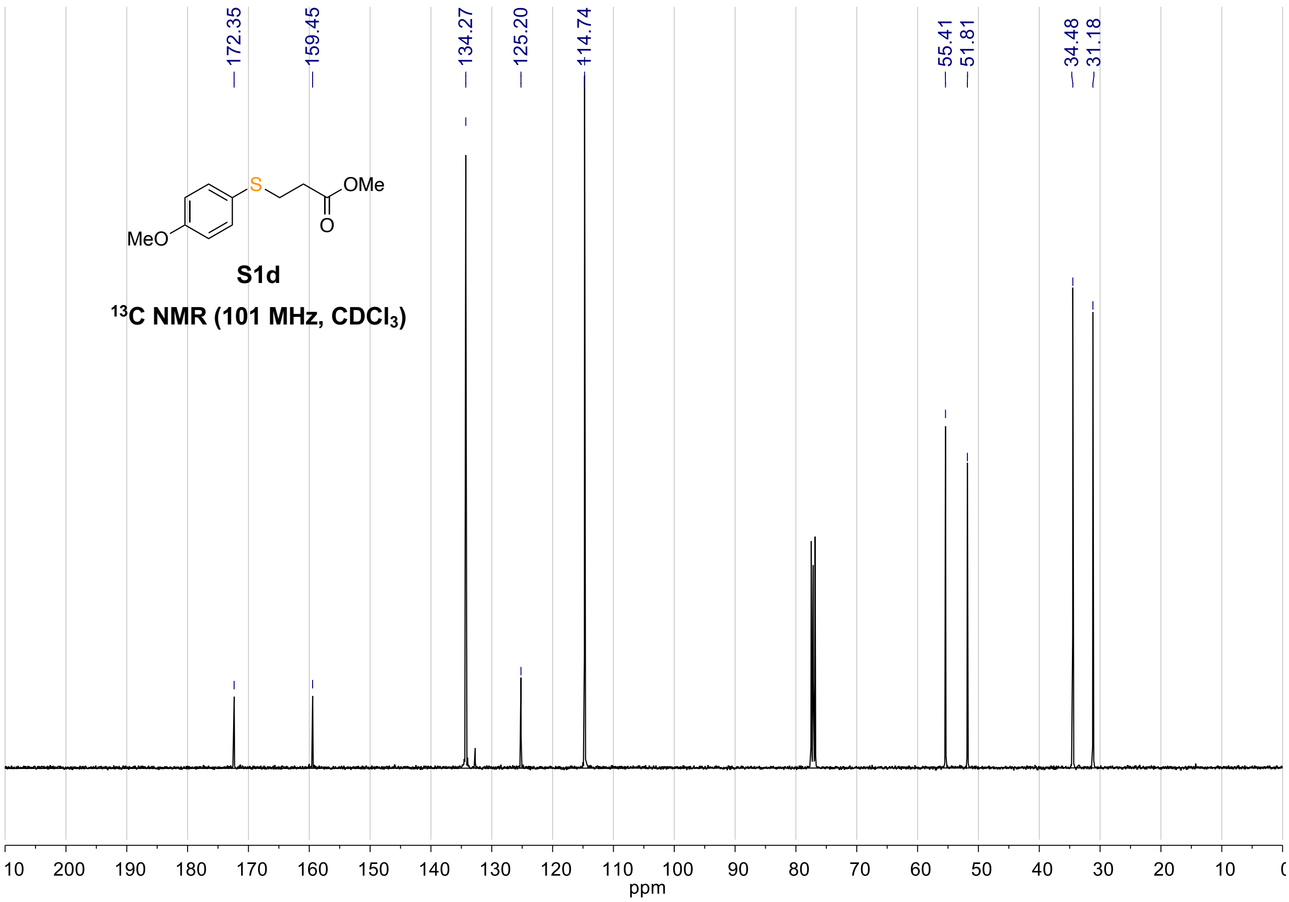

Methyl 3-(pyridin-2-ylthio)propanoate (S1e)

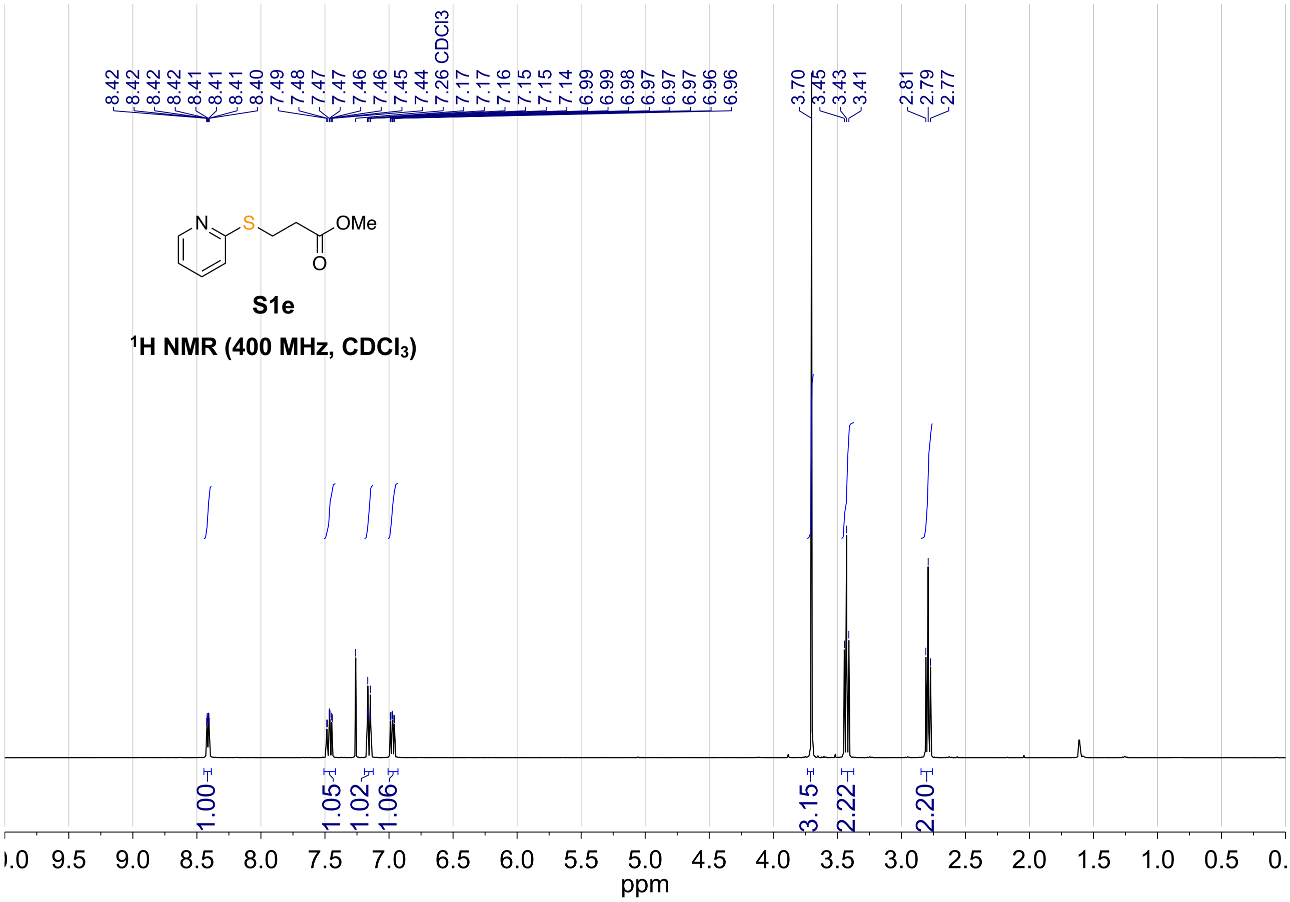

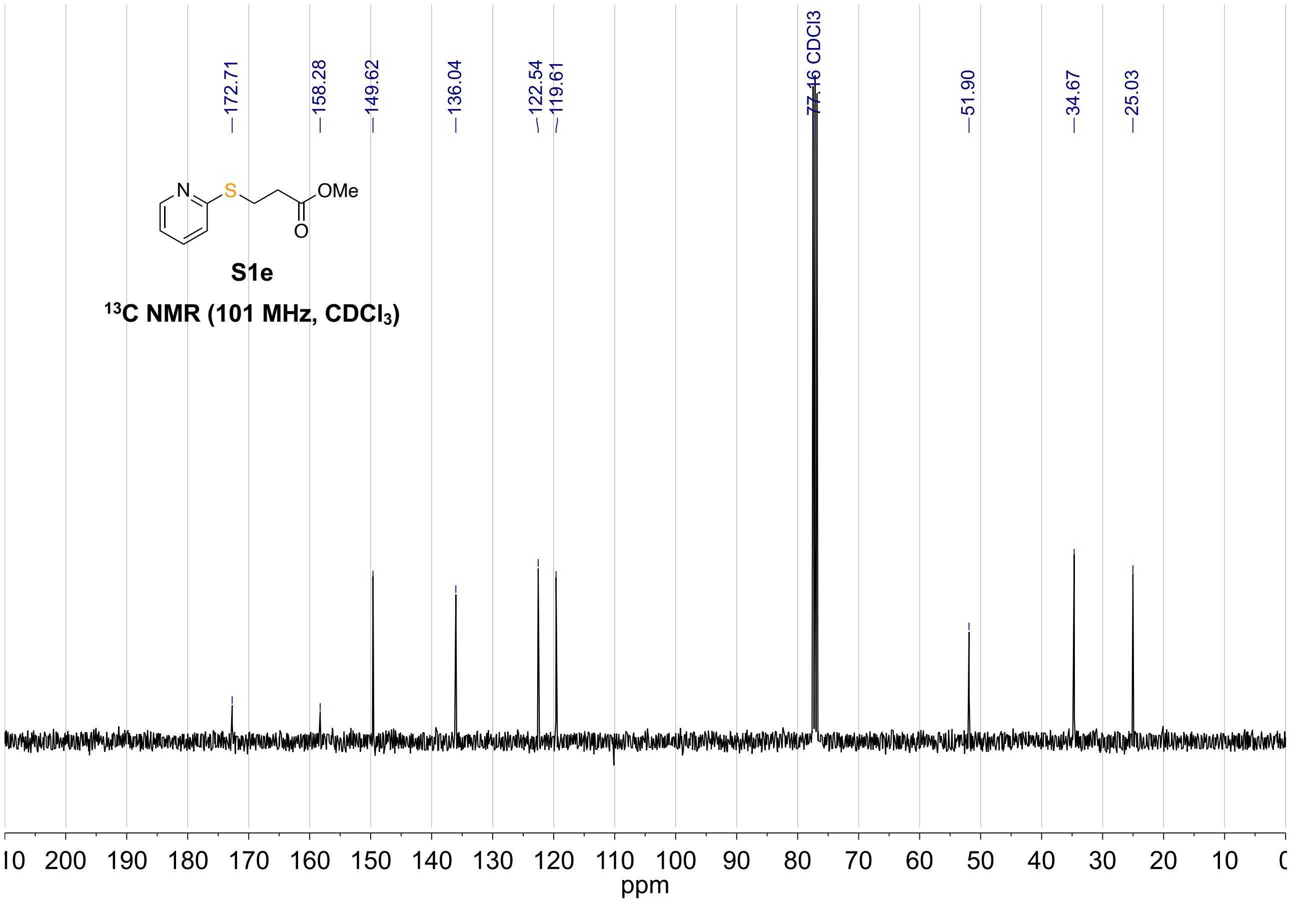

Methyl 3-(isopropylthio)propanoate (S1f)

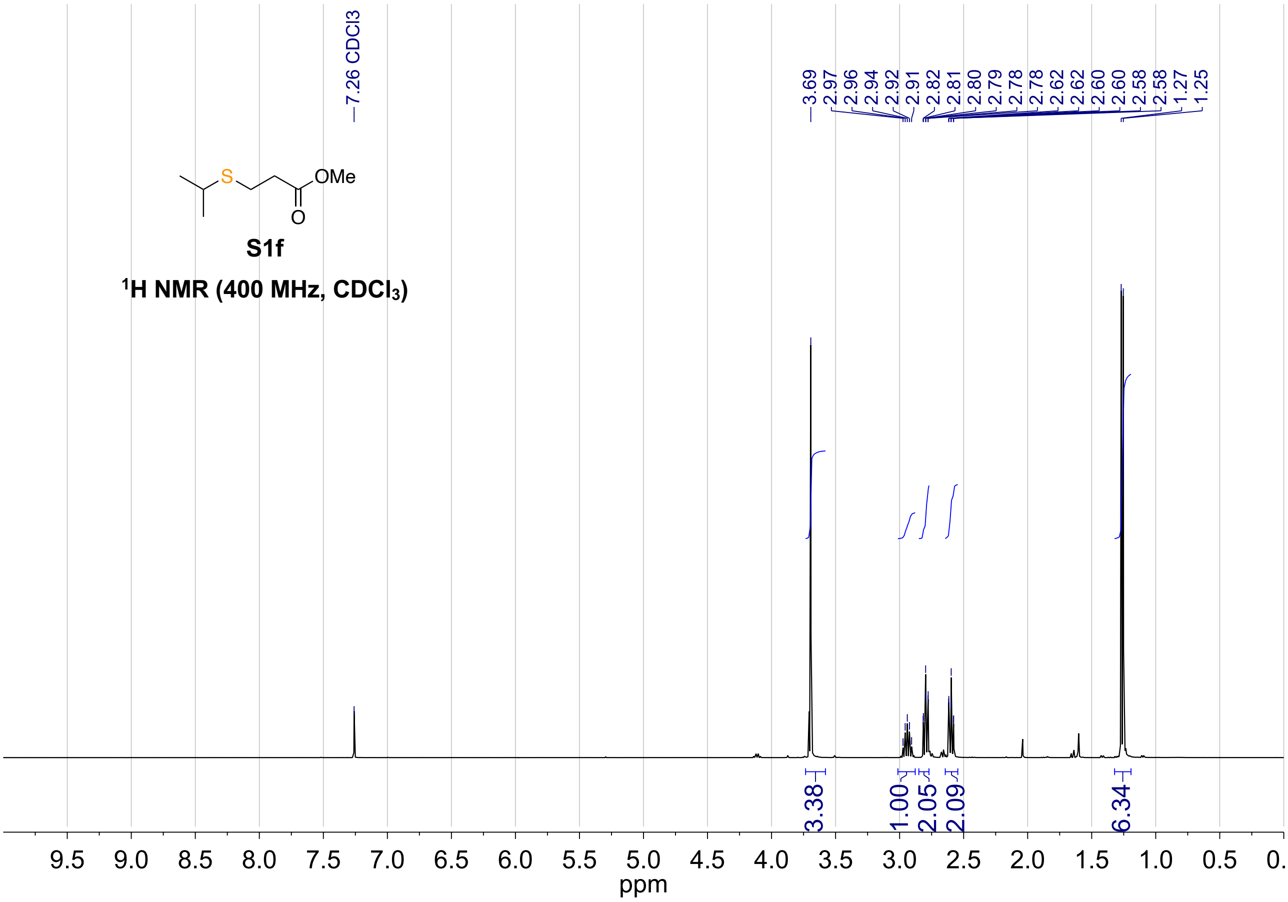

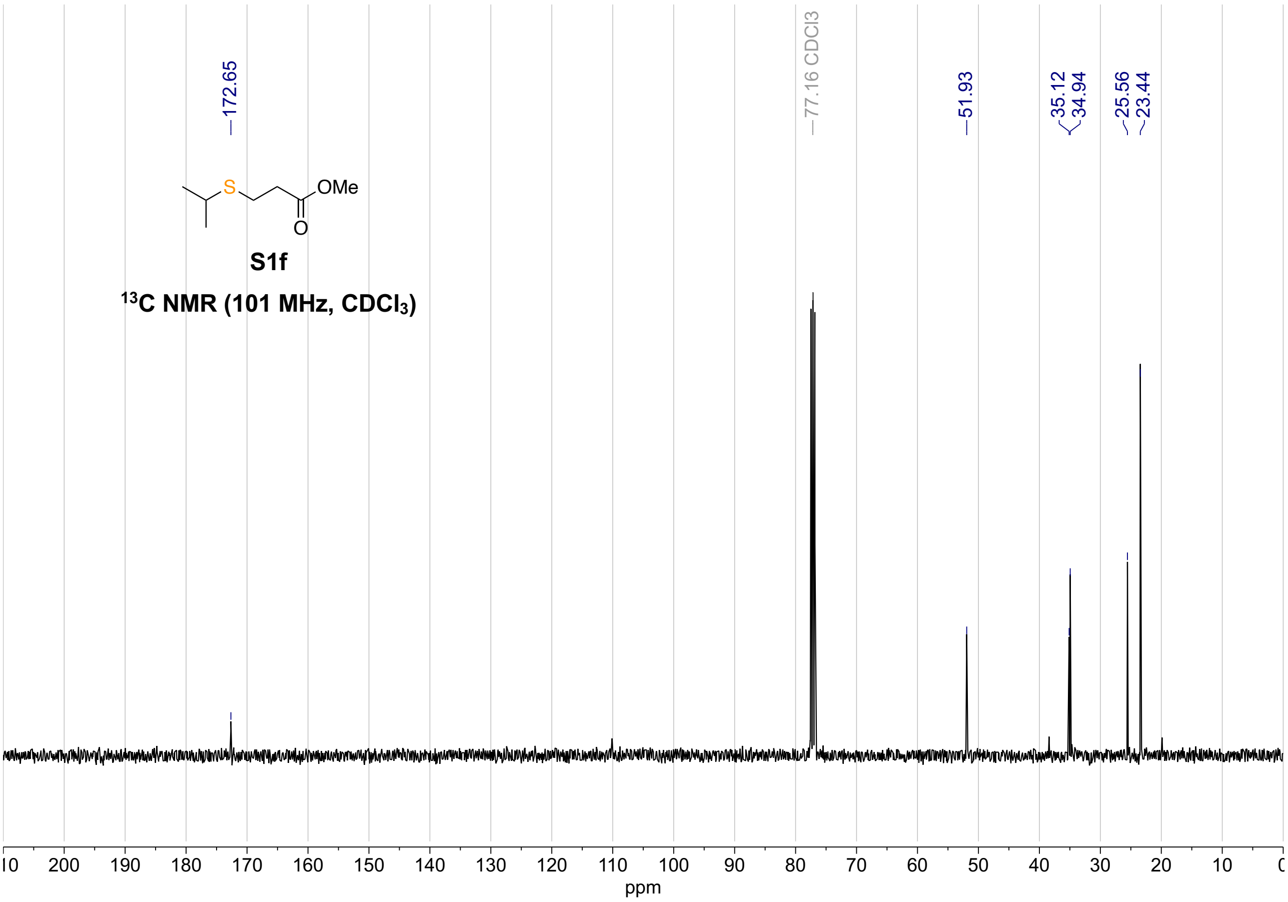

Methyl 3-(methylthio)propanoate (S1g)

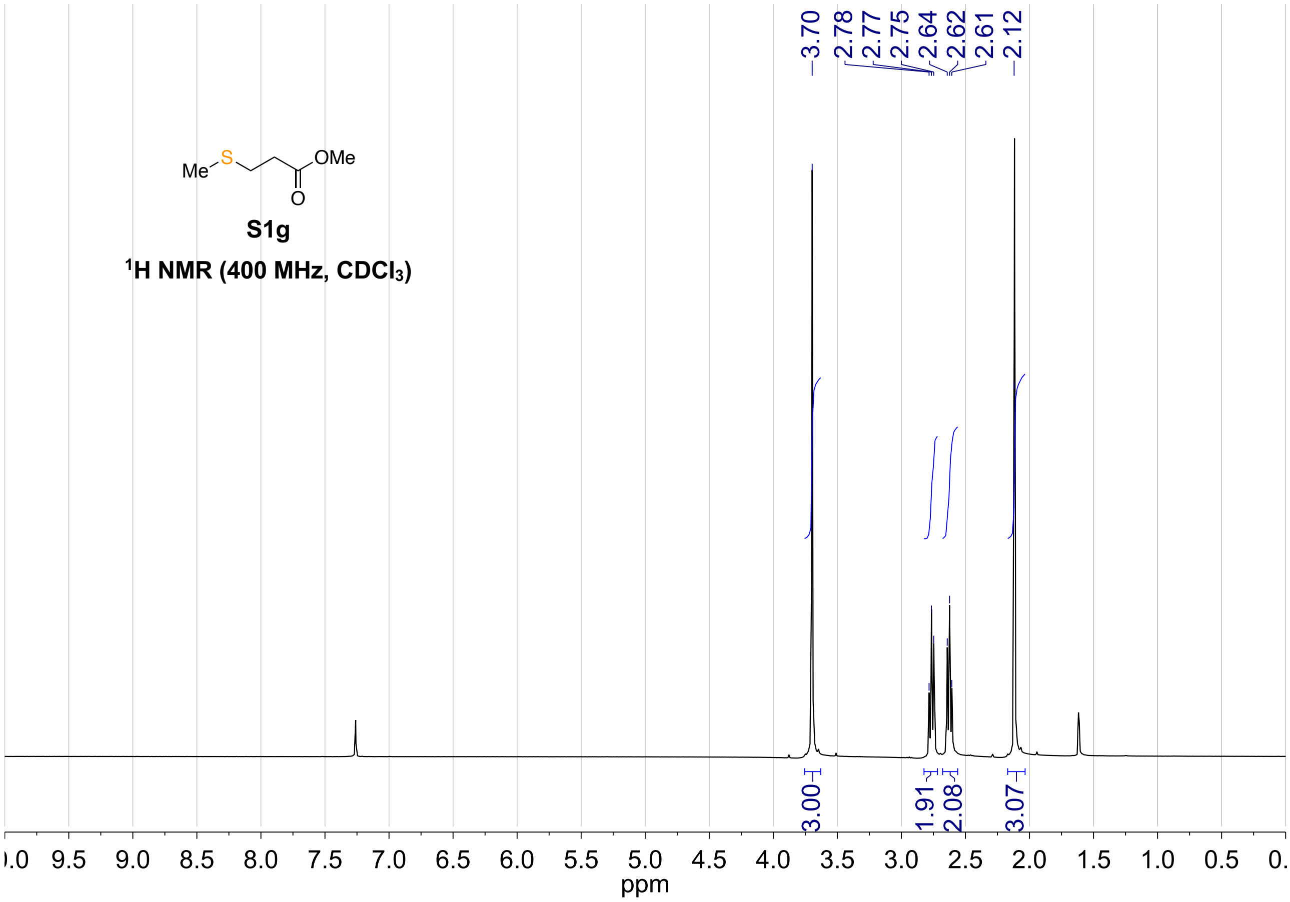

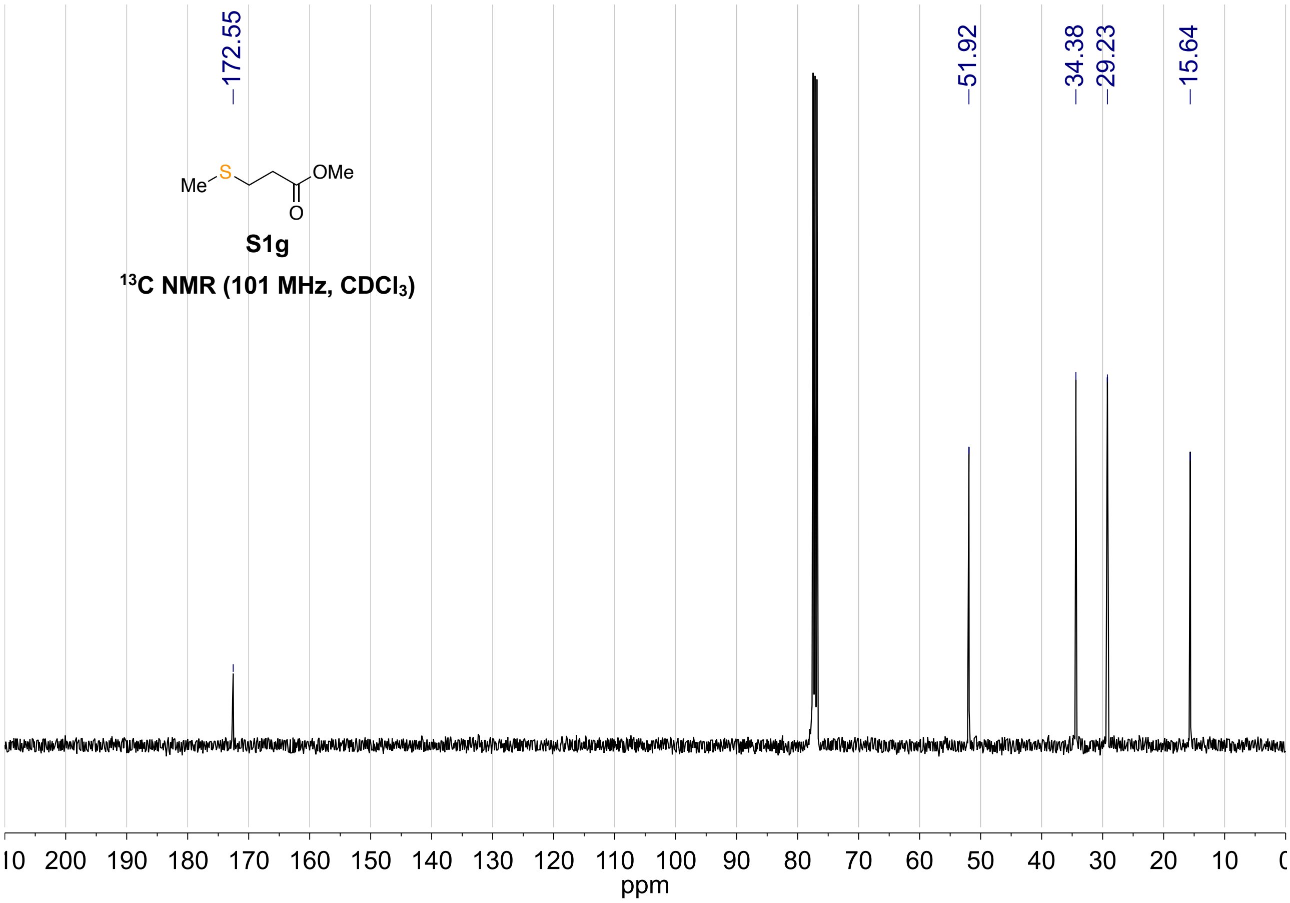

Methyl 3-(*p*-tolylsulfinyl)propanoate (S2a)

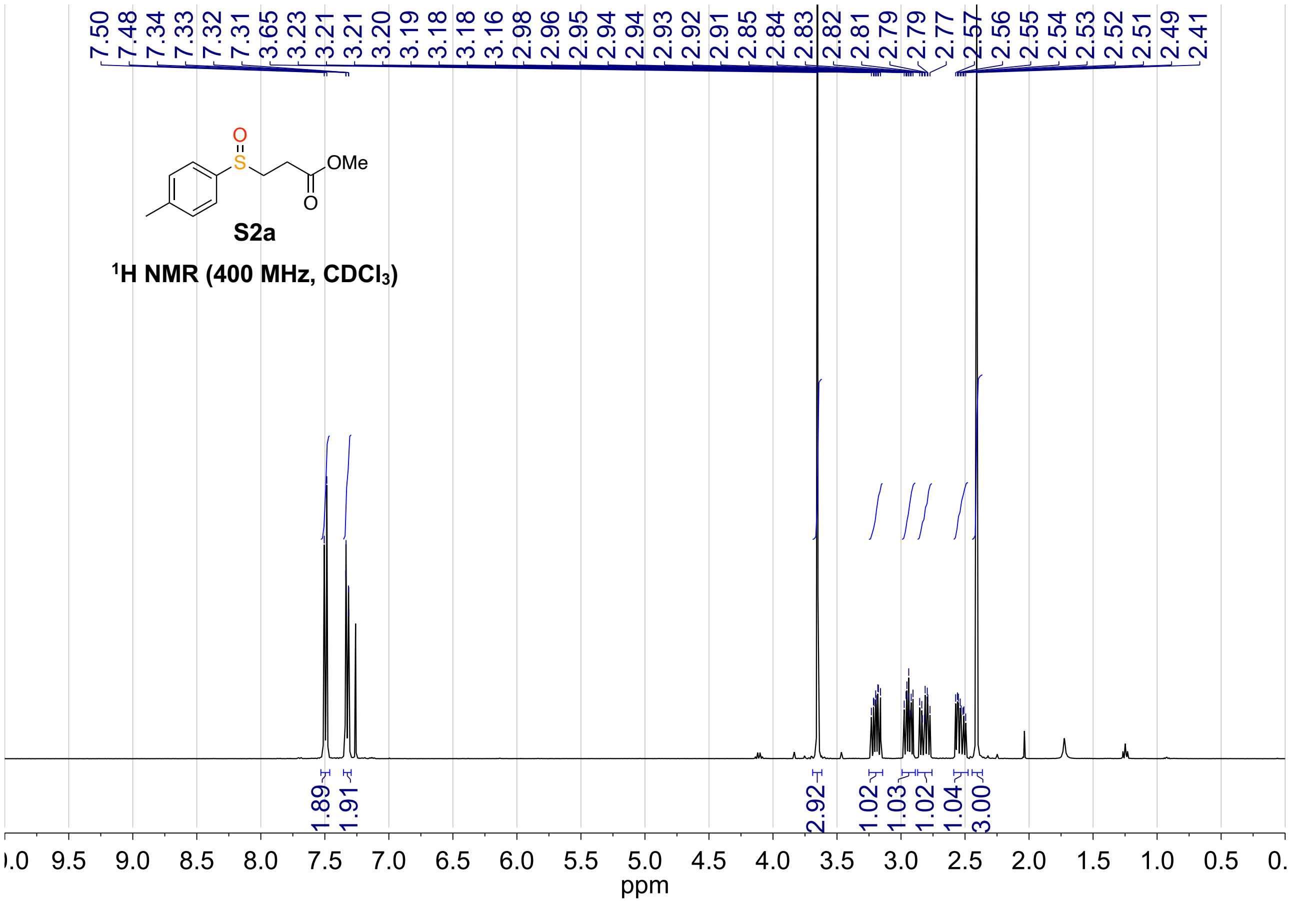

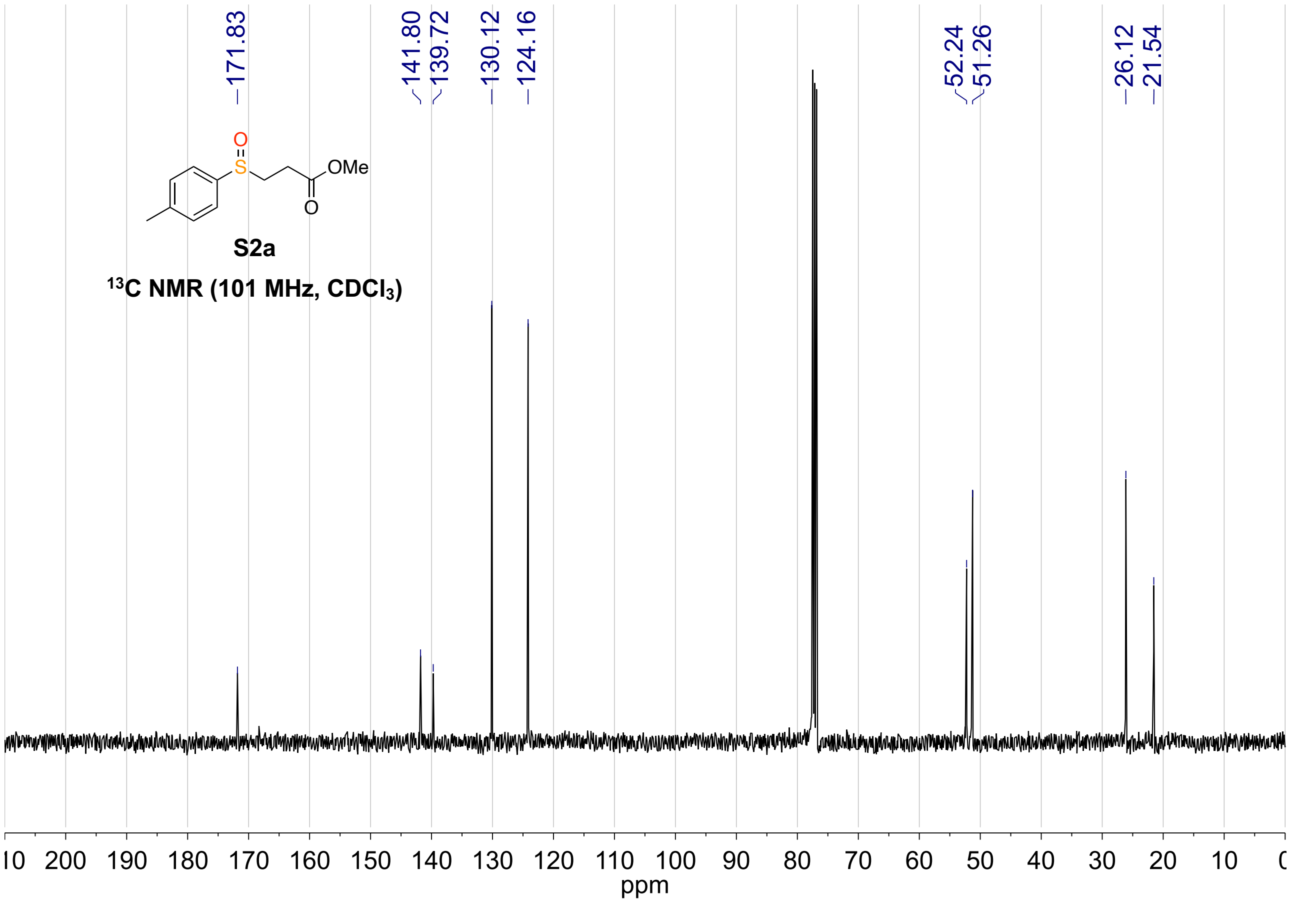

Methyl 3-(phenylsulfinyl)propanoate (S2b)

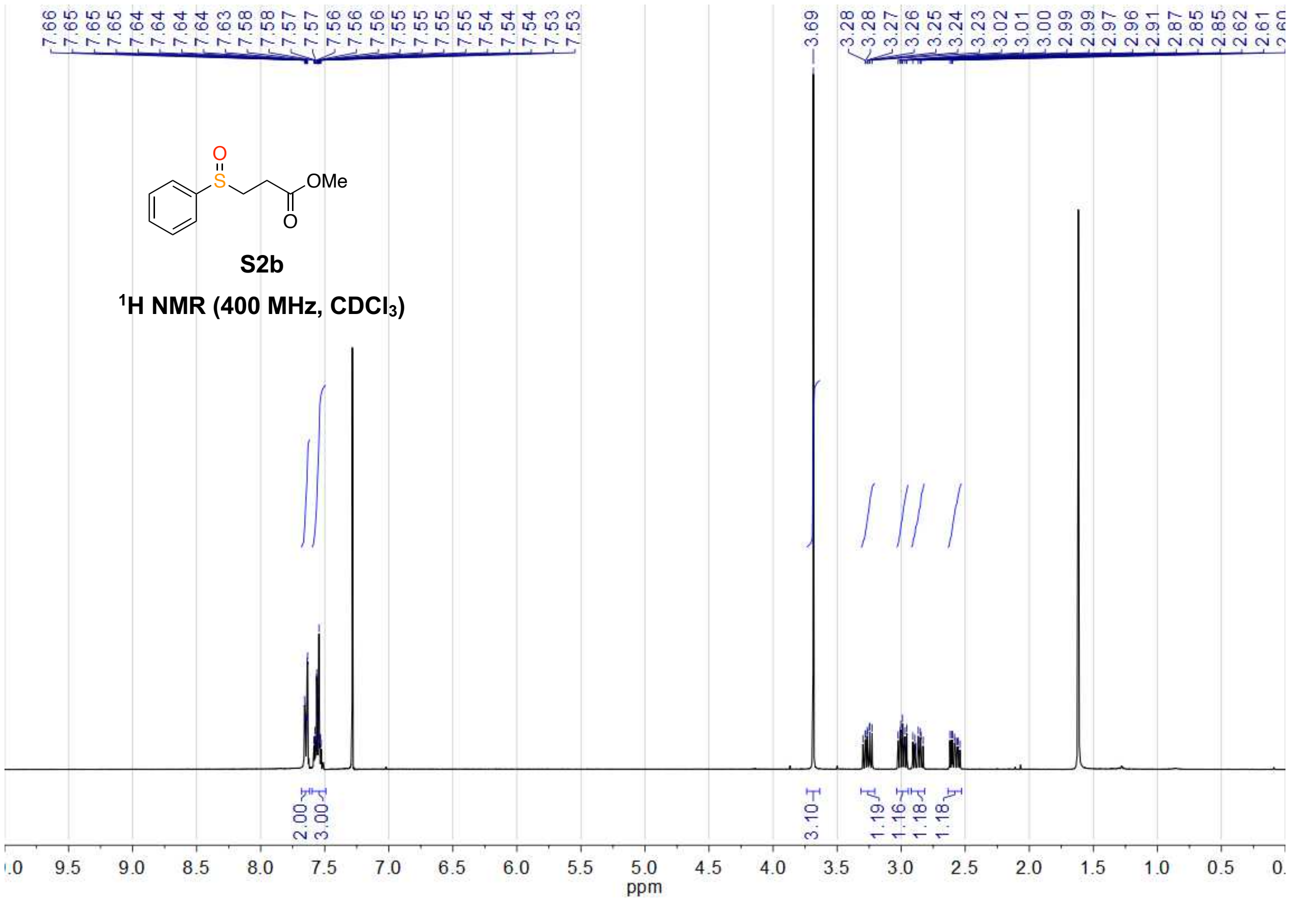

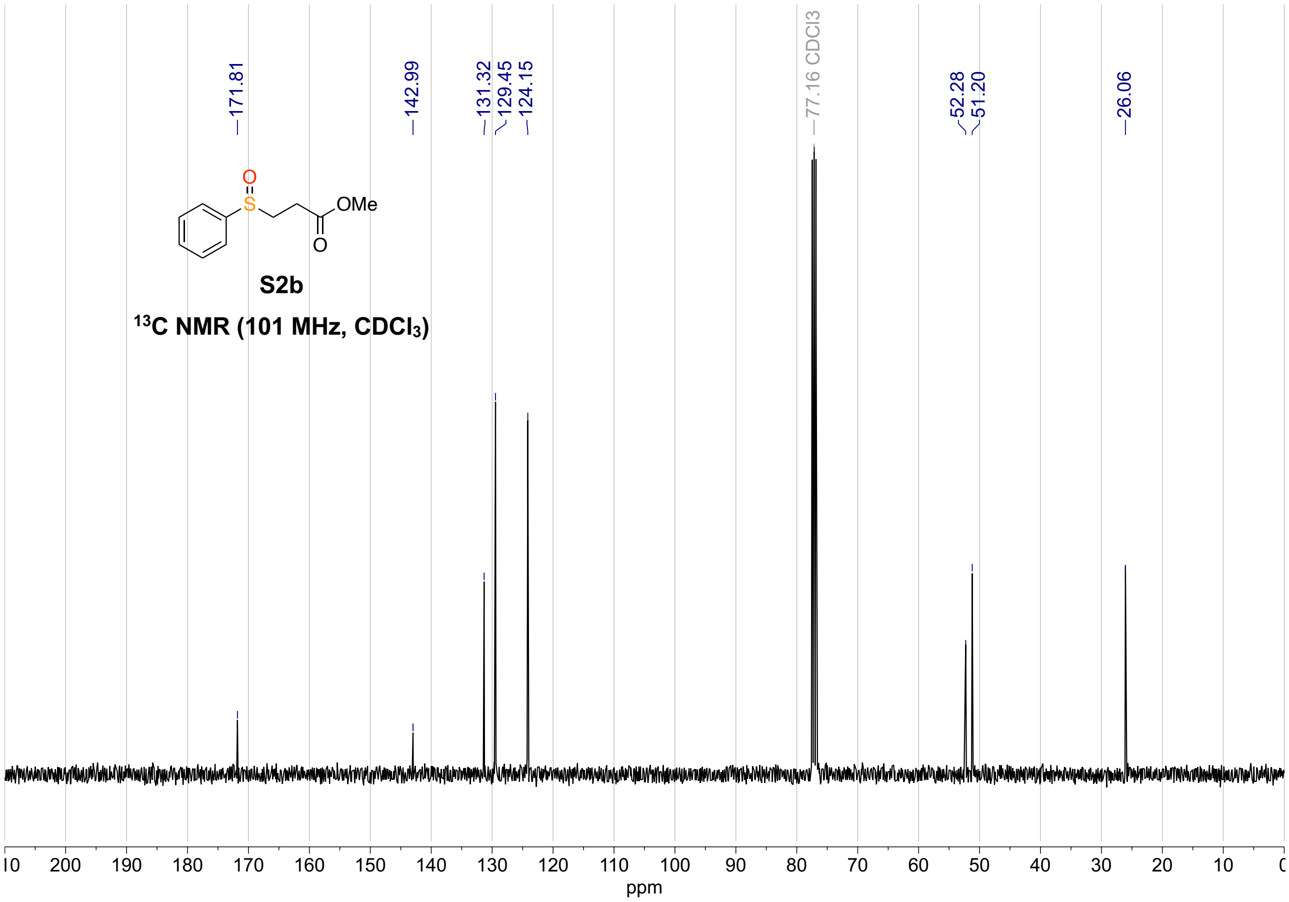

Methyl 3-((4-fluorophenyl)sulfinyl)propanoate (S2c)

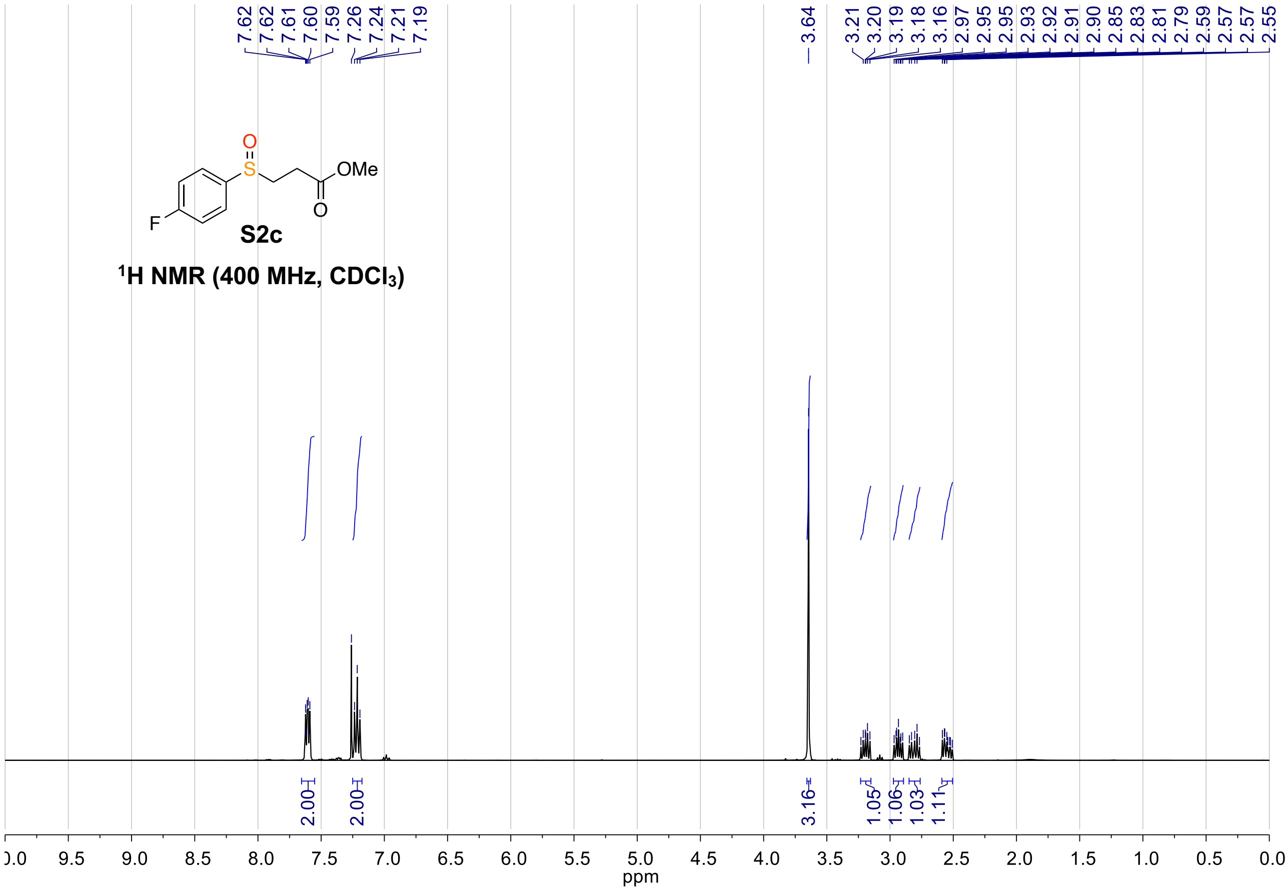

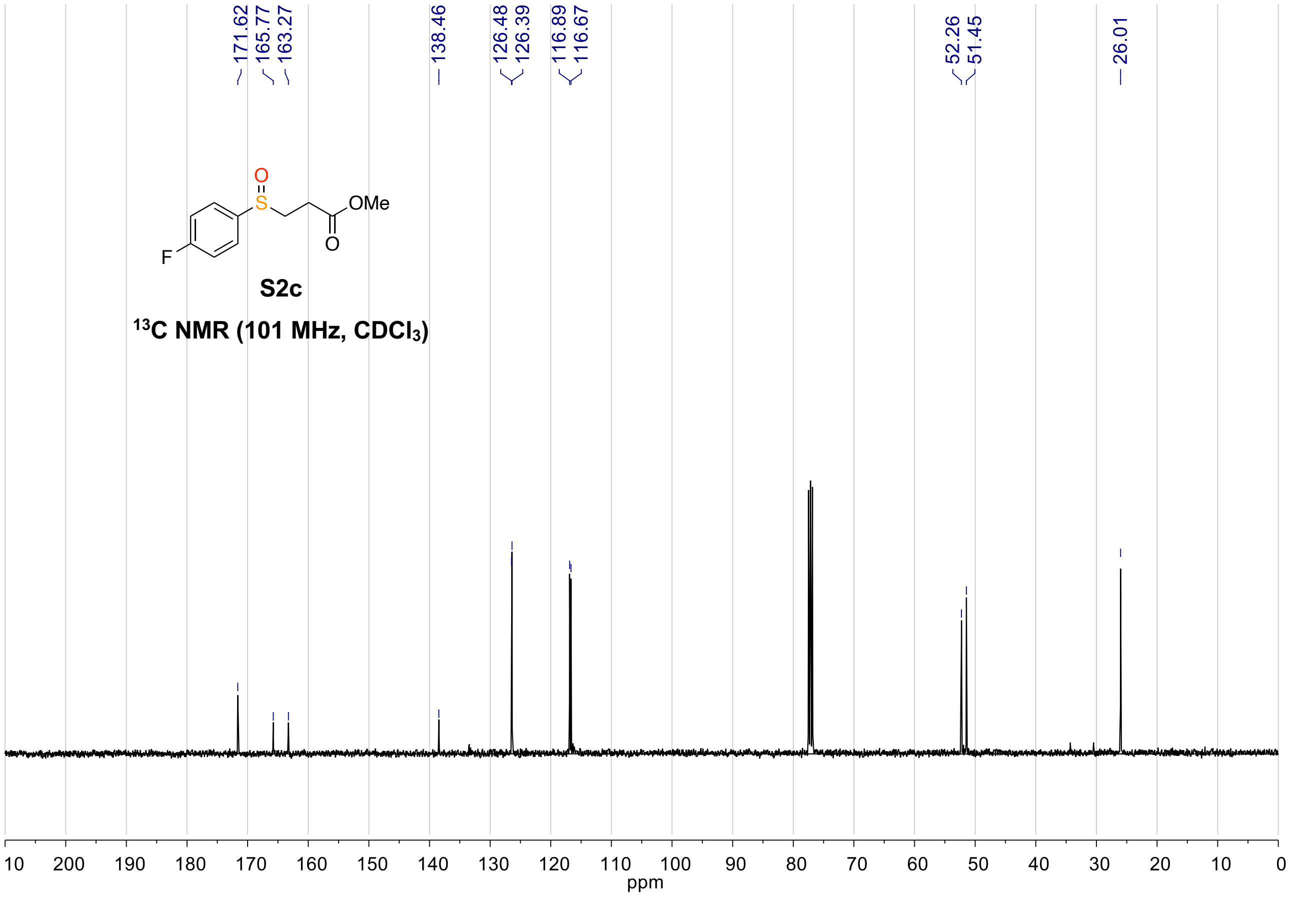

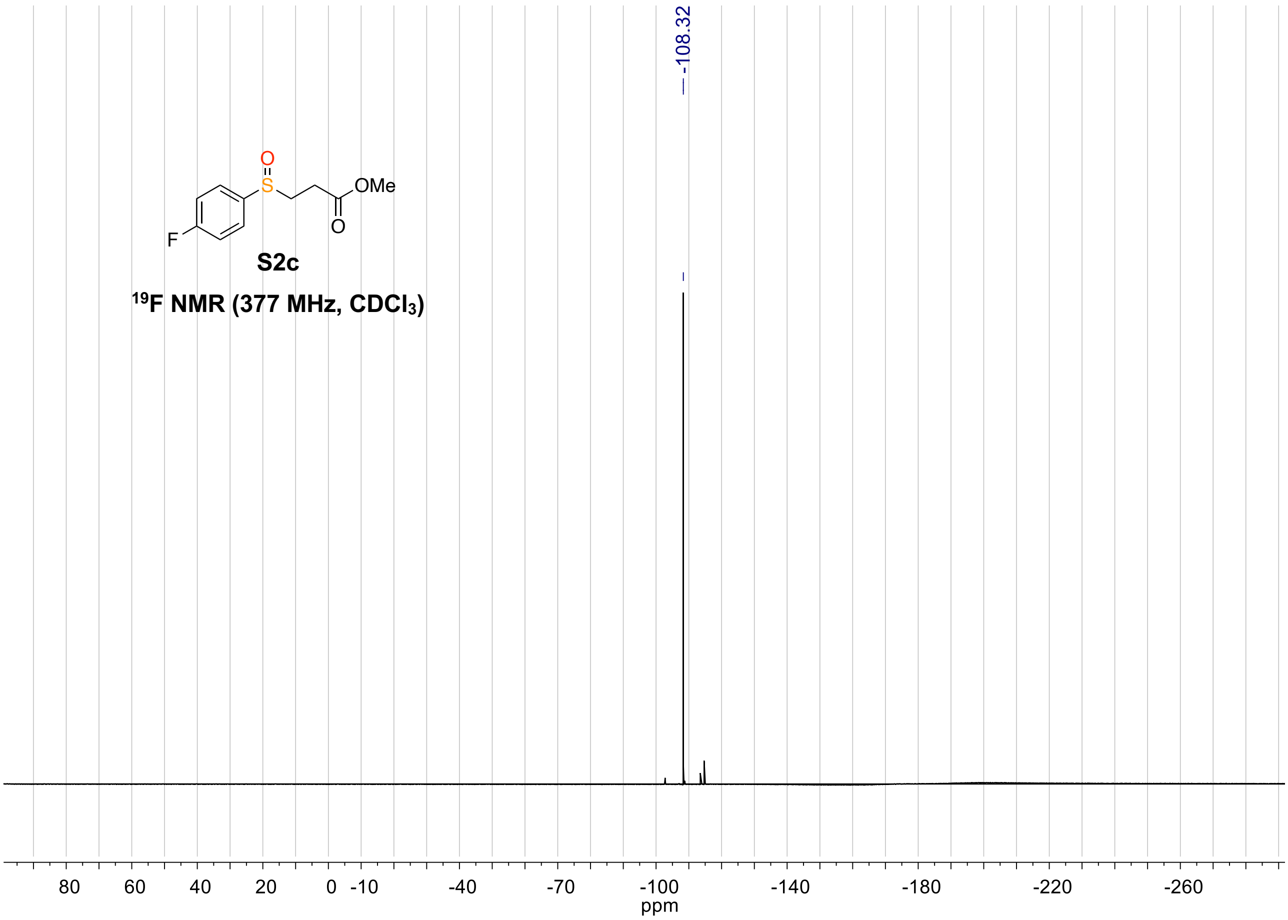

S2c

^{19}F NMR (377 MHz, $CDCl_3$)

Methyl 3-((4-methoxyphenyl)sulfinyl)propanoate (S2d)

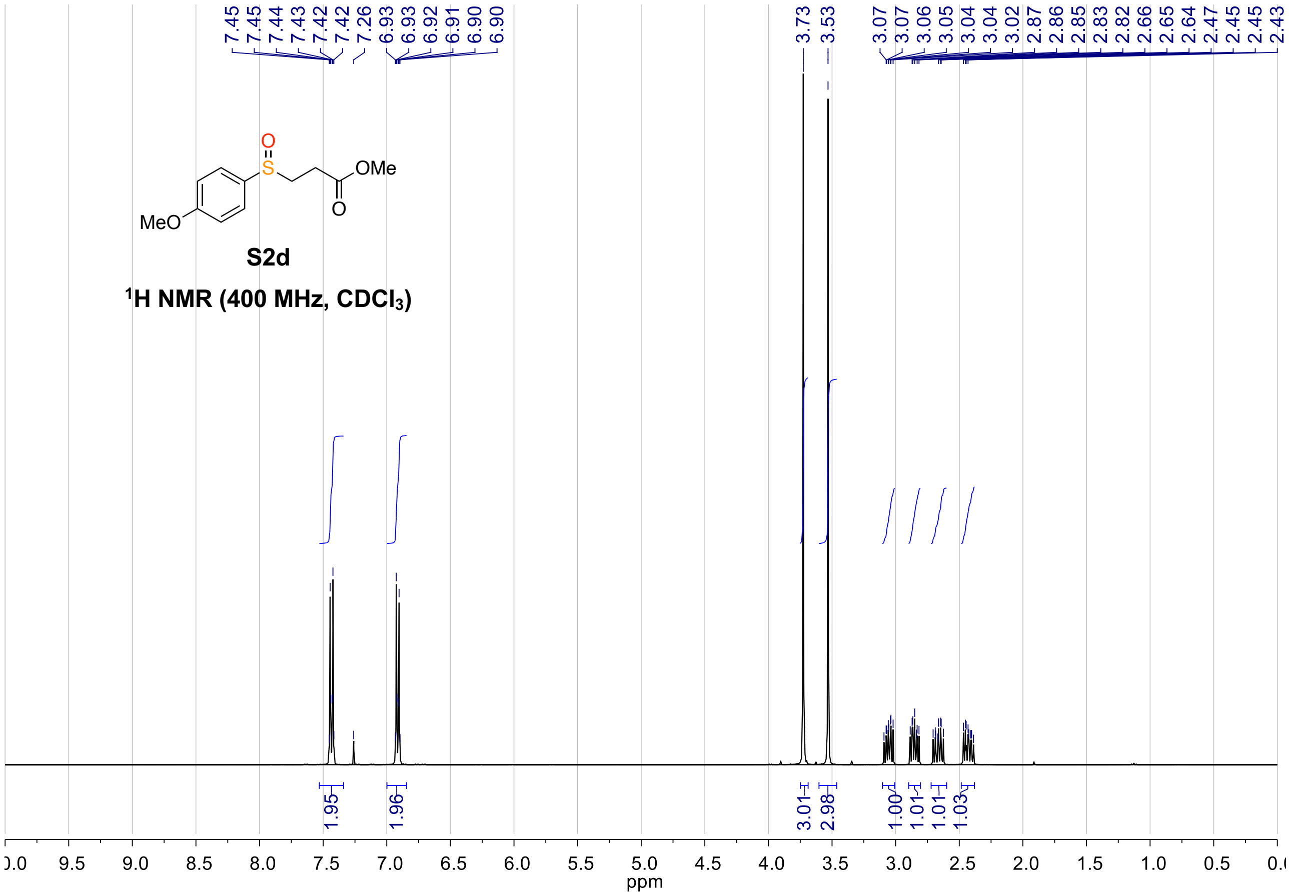

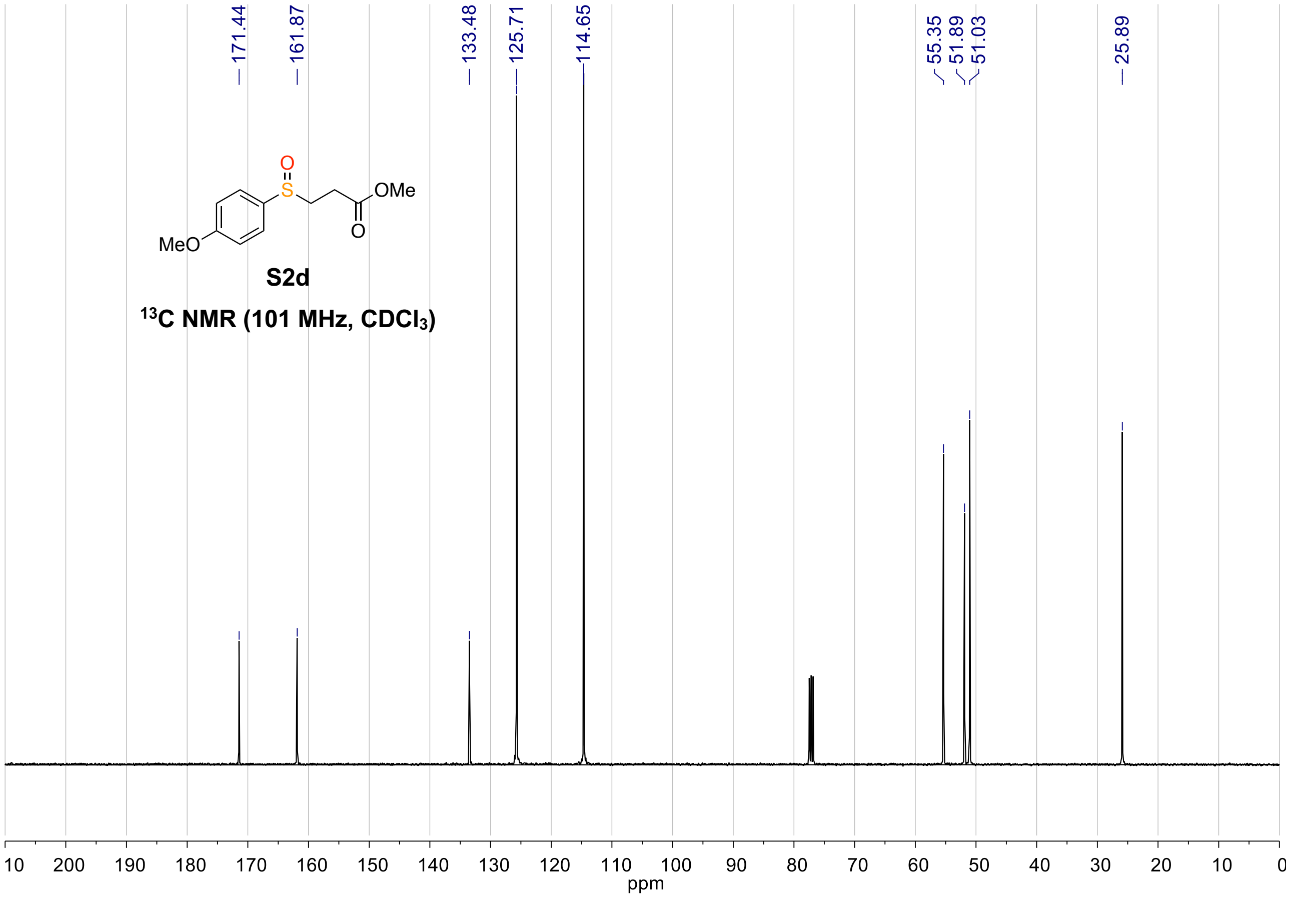

Methyl 3-(pyridin-2-ylsulfinyl)propanoate (S2e)

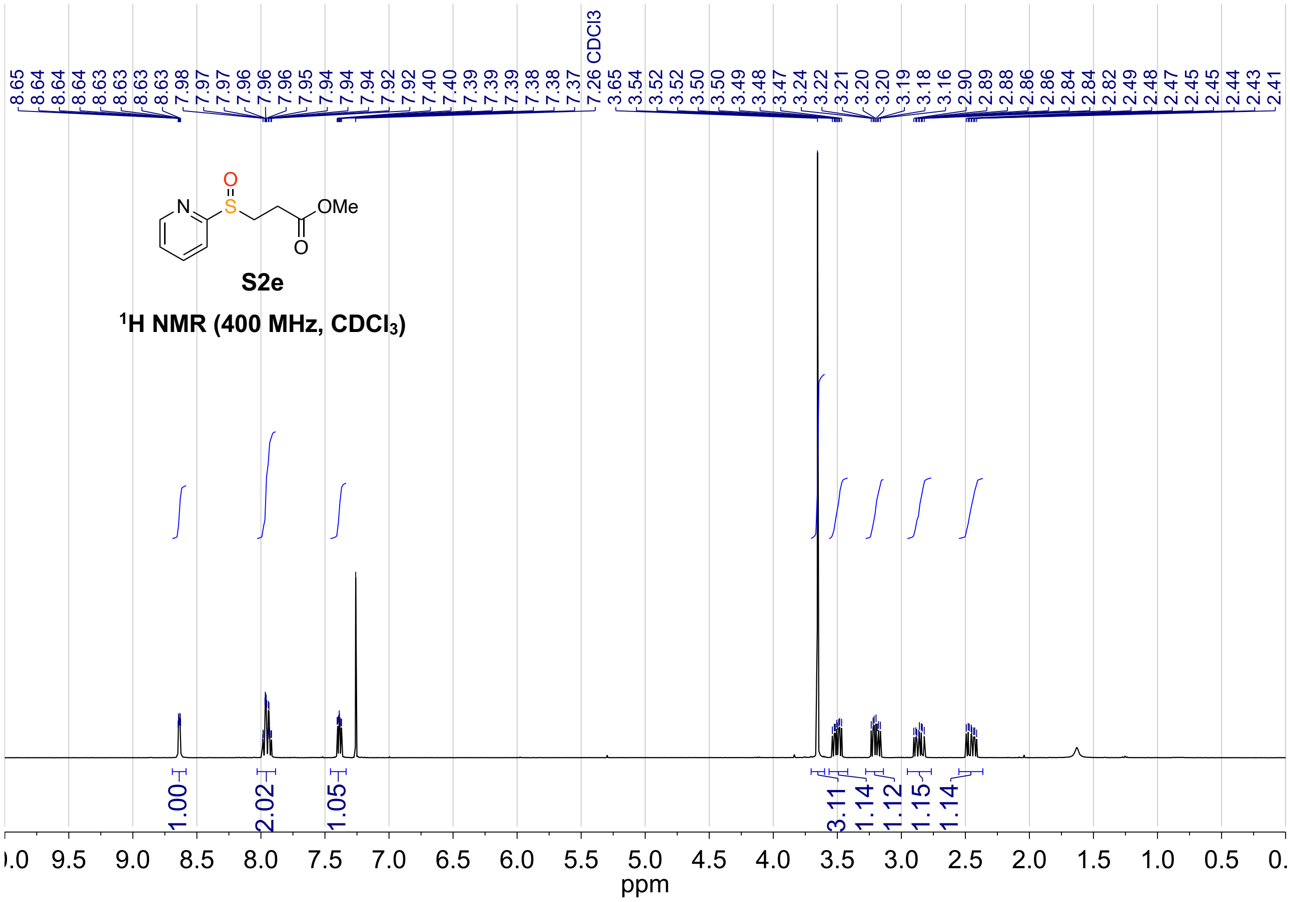

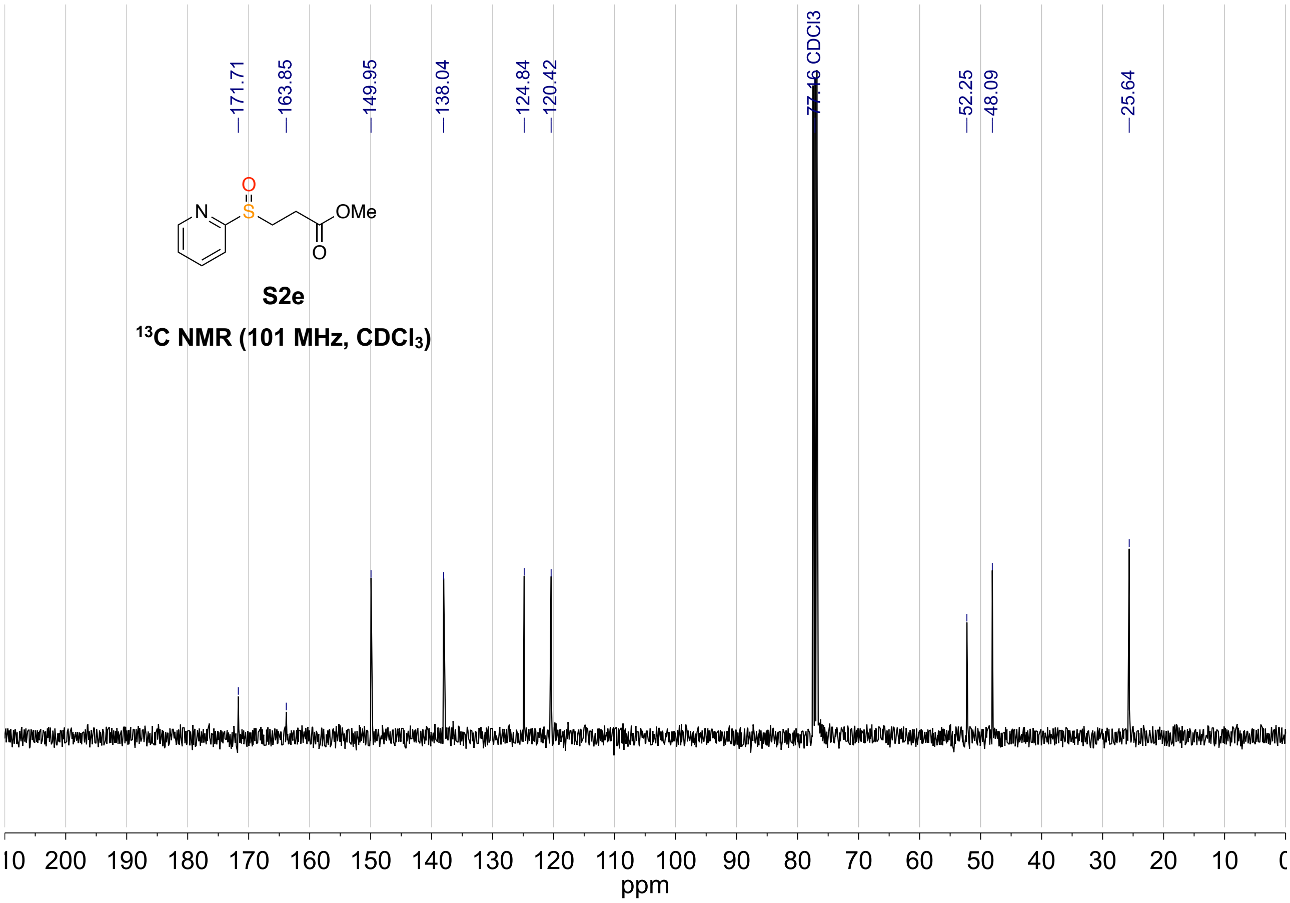

Methyl 3-(isopropylsulfinyl)propanoate (S2f)

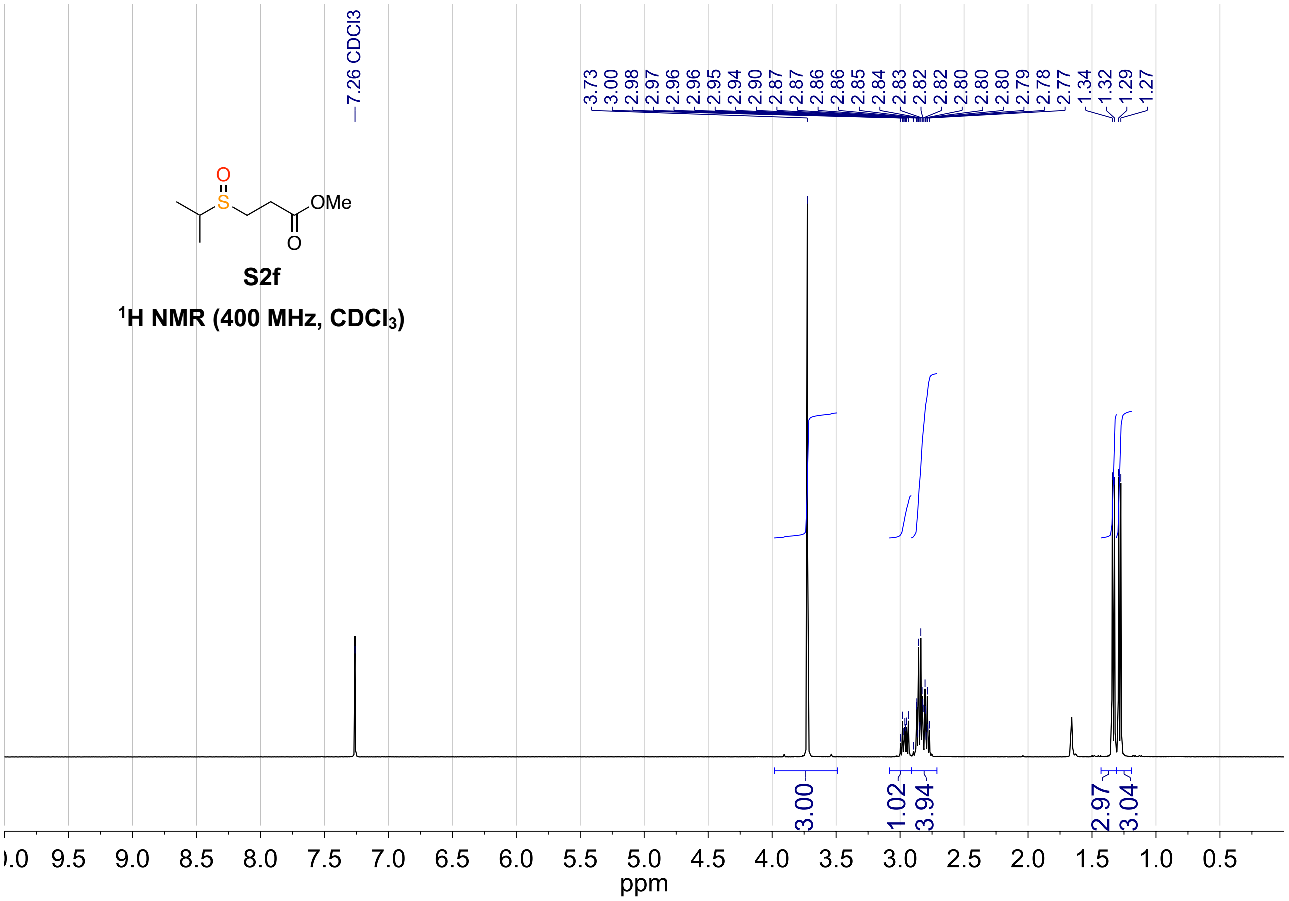

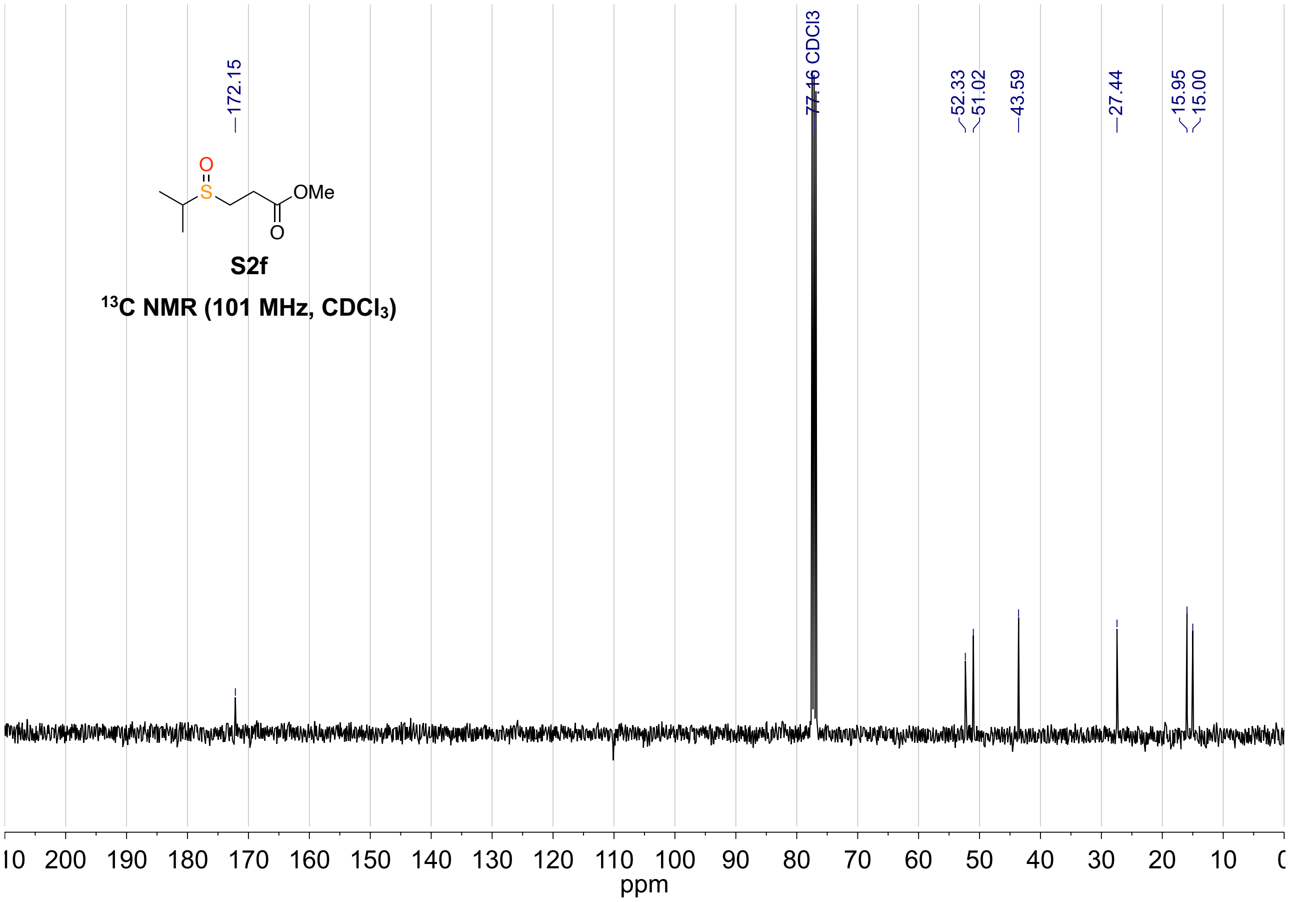

Methyl 3-(methanesulfinyl)propanoate (S2g)

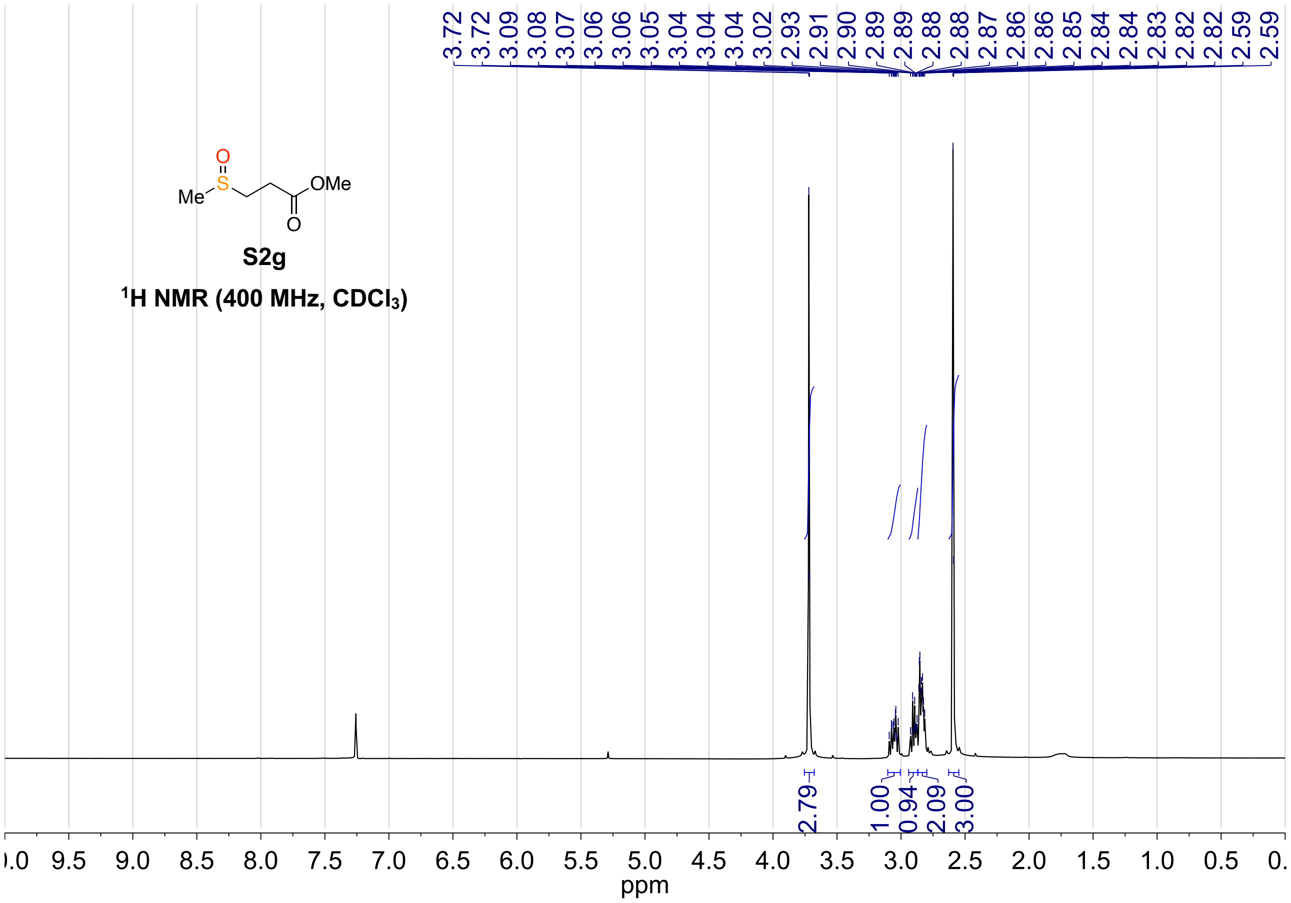

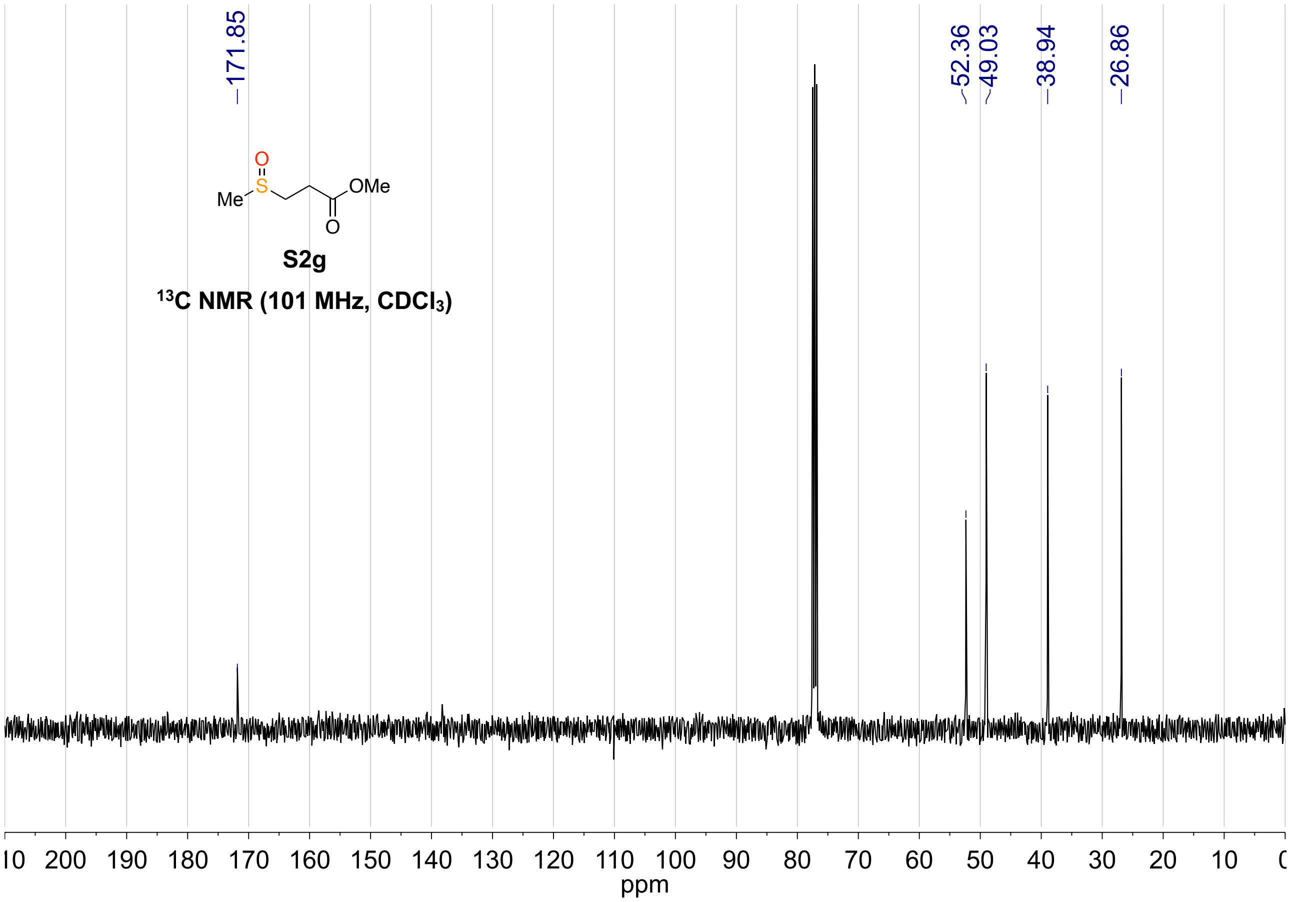

Methyl 3-(N-(tert-butoxycarbonyl)-4-methylphenylsulfonimidoyl)propanoate (S3a)

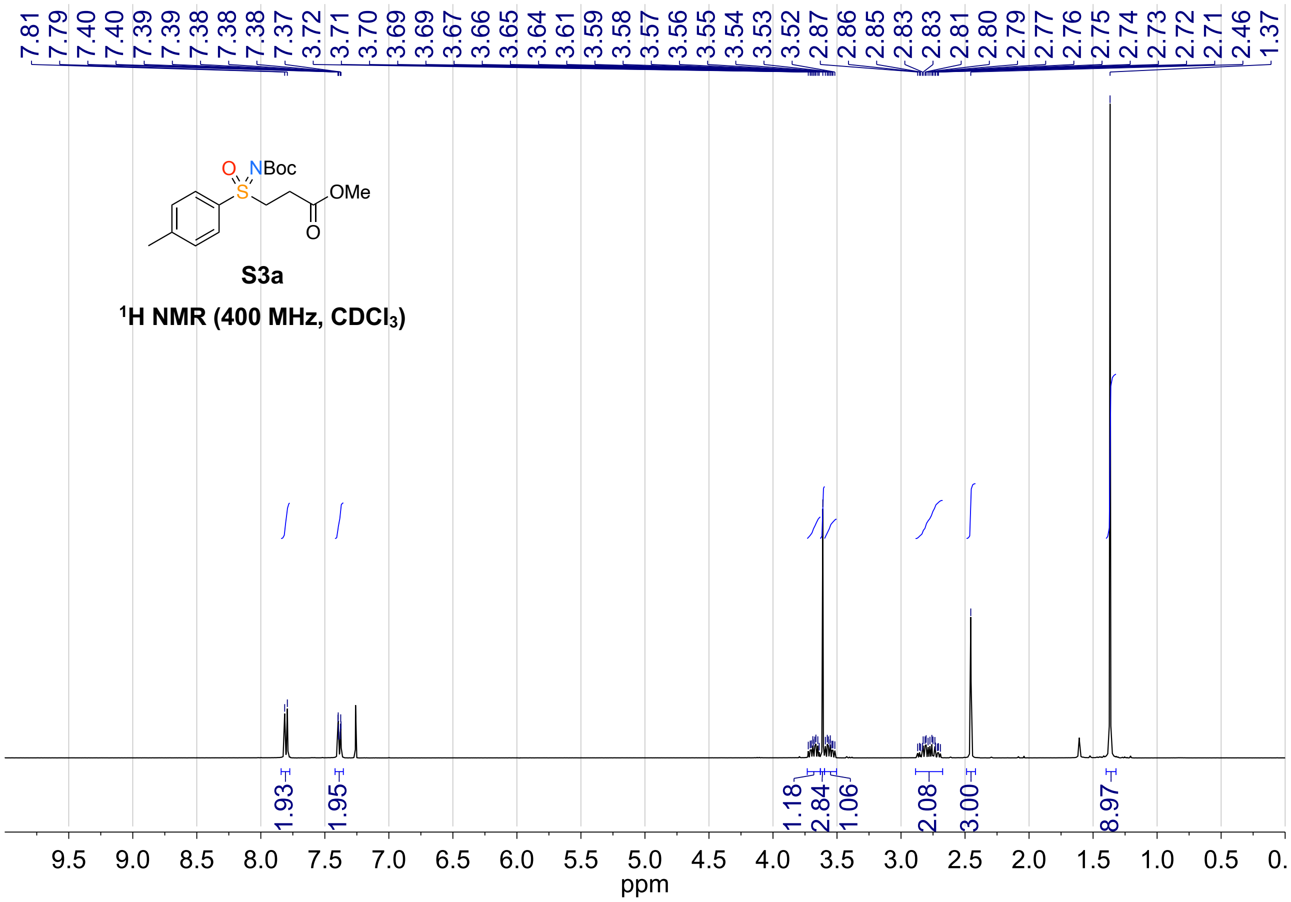

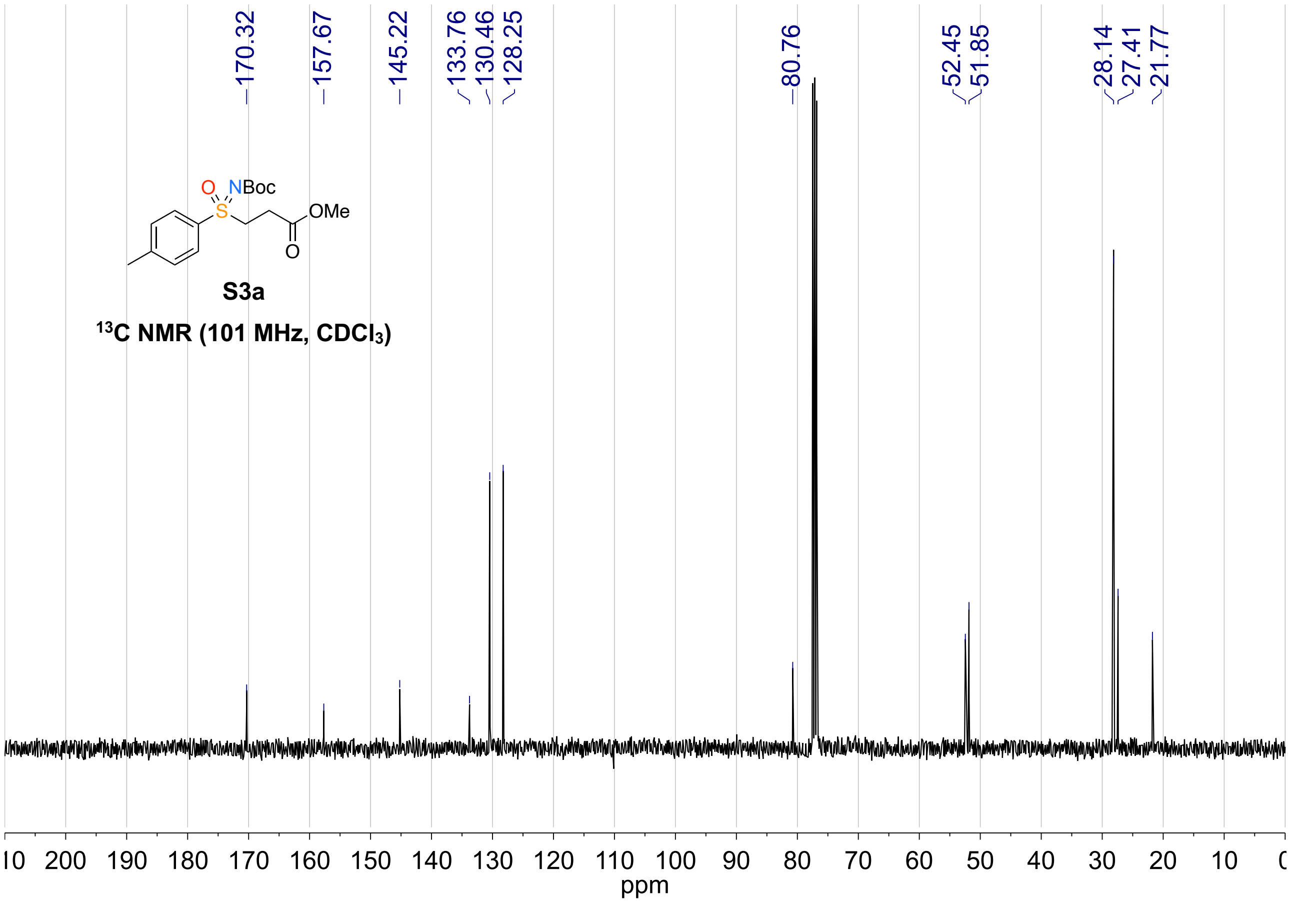

Methyl 3-(*N*-(*tert*-butoxycarbonyl)phenylsulfonimidoyl)propanoate (S3b)

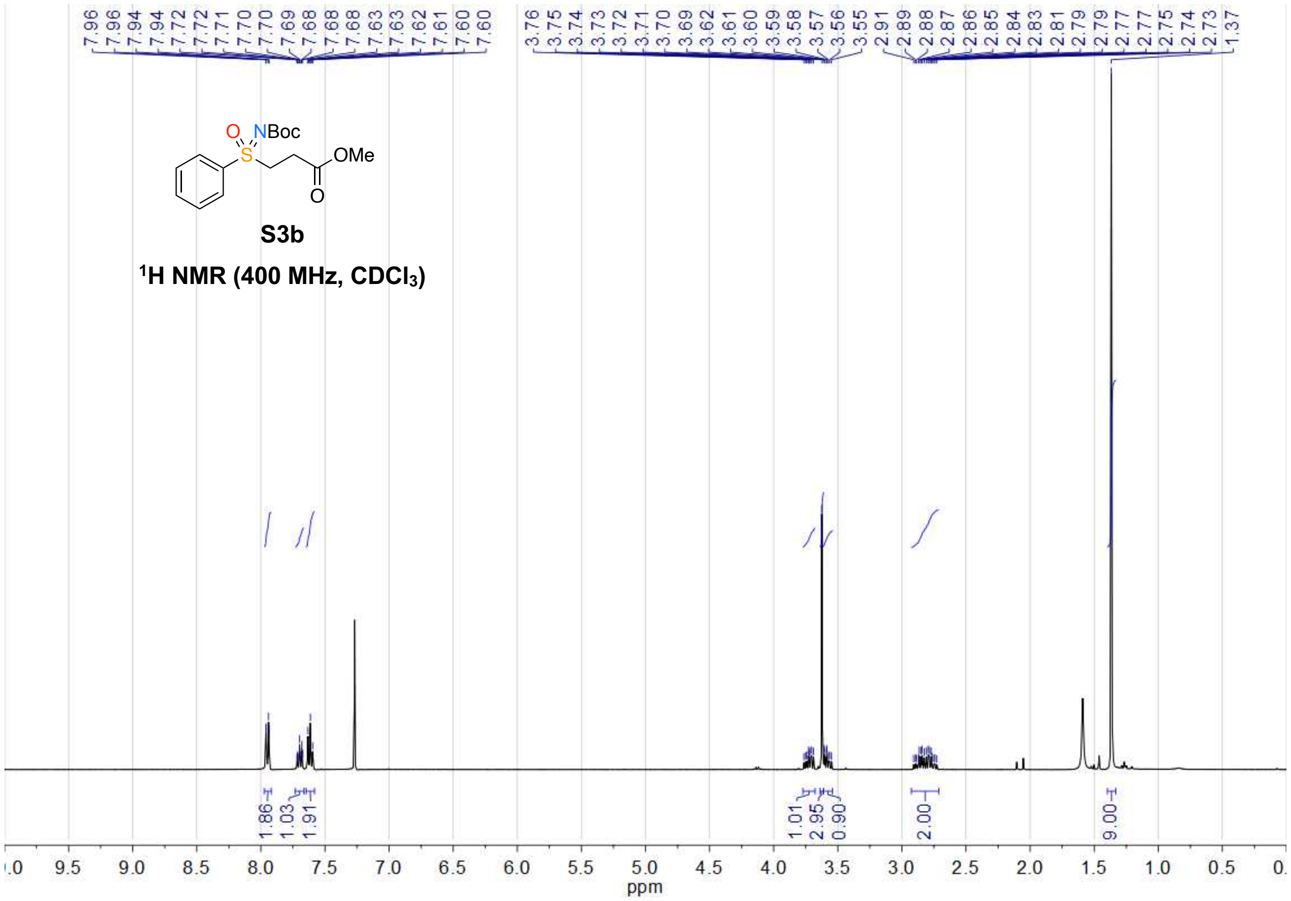

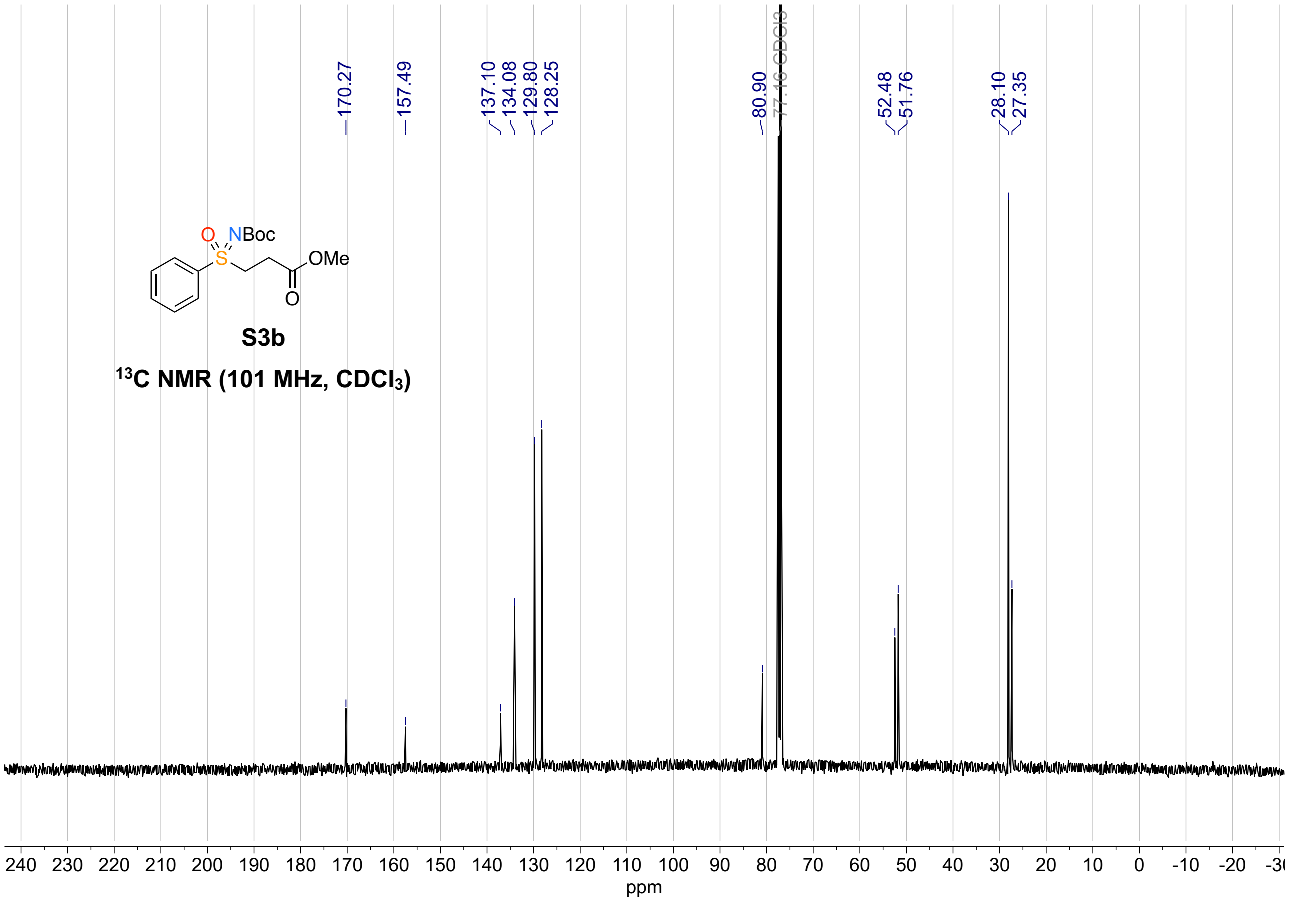

Methyl 3-(*N*-(*tert*-butoxycarbonyl)-4-fluorophenylsulfonimidoyl)propanoate (S3c)

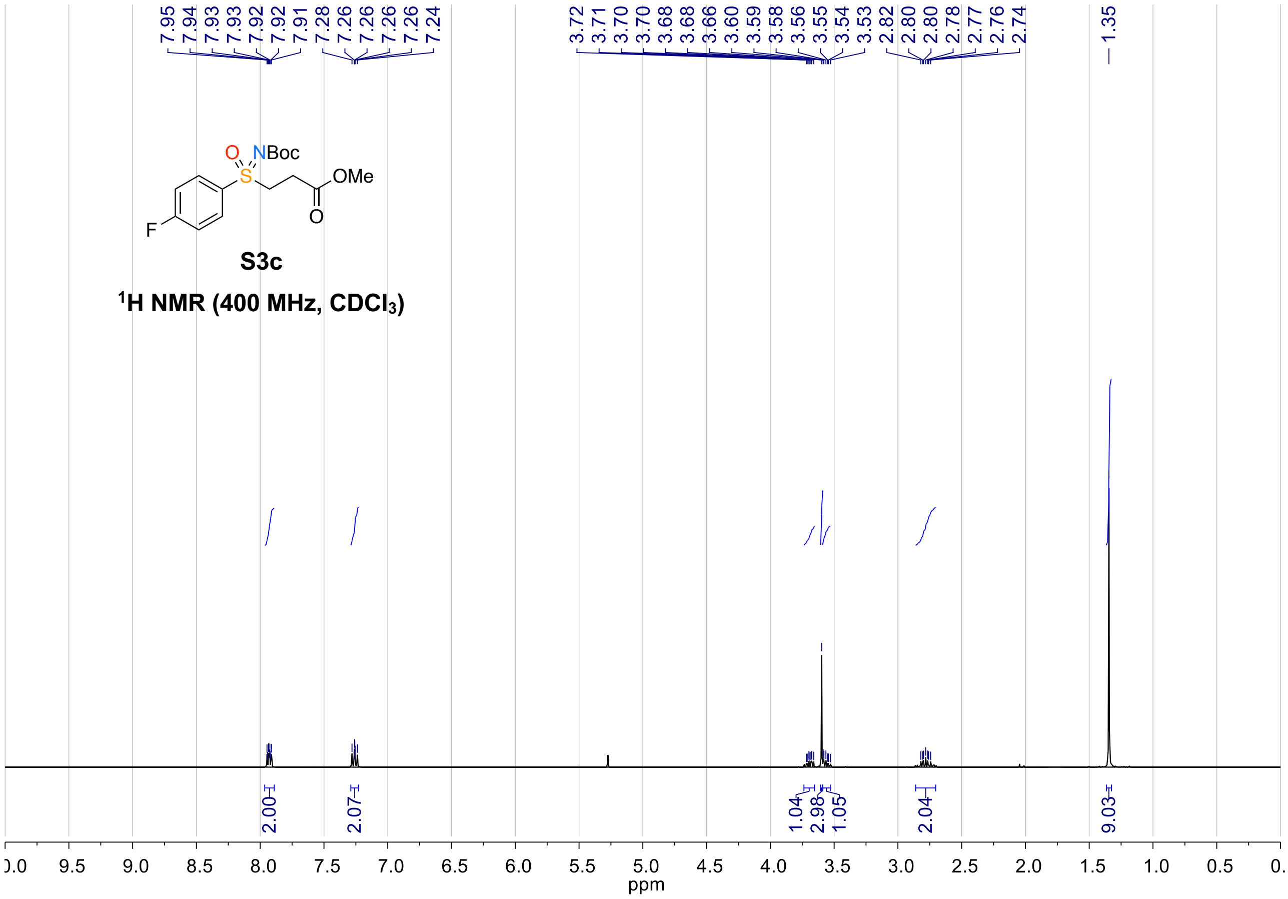

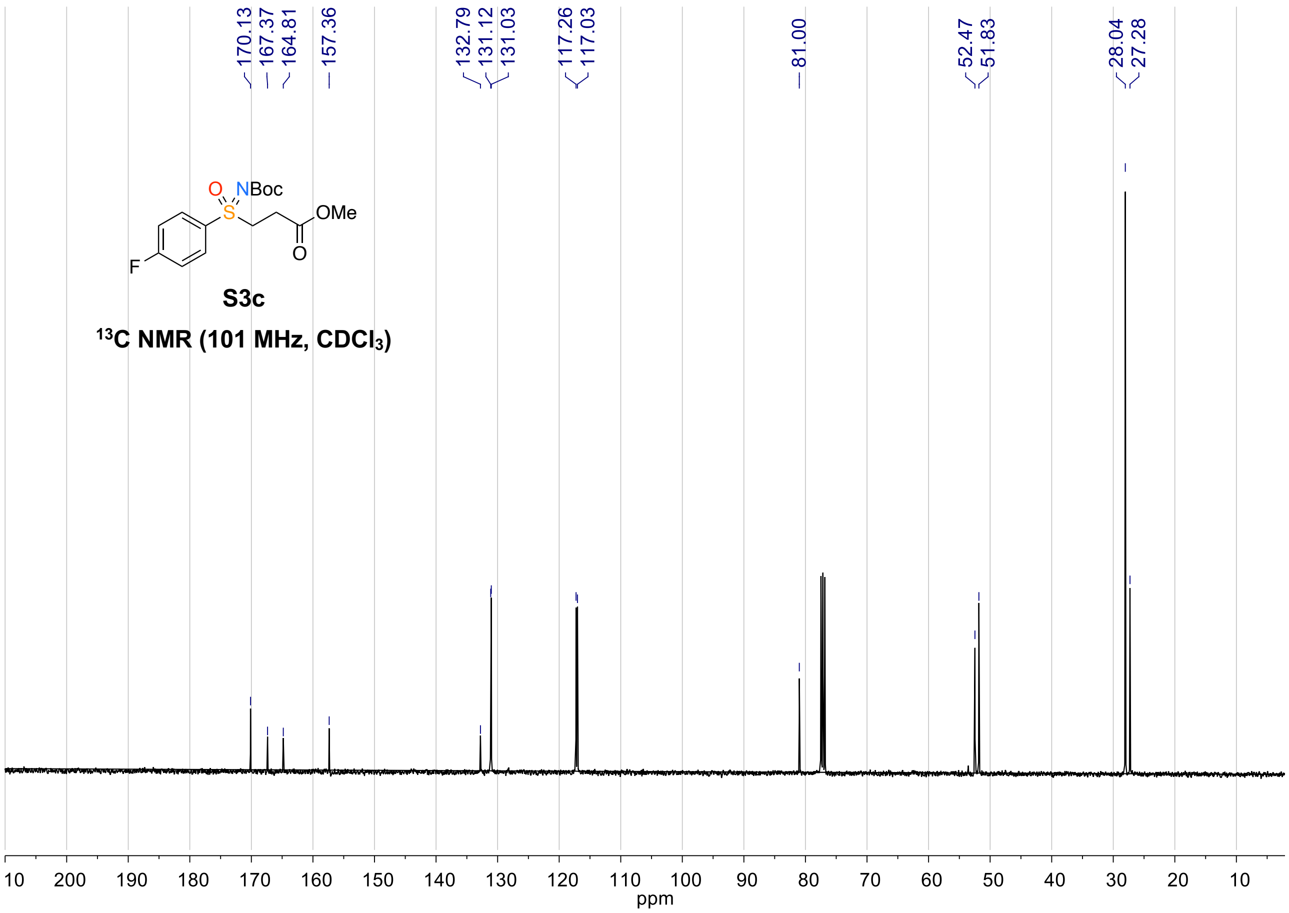

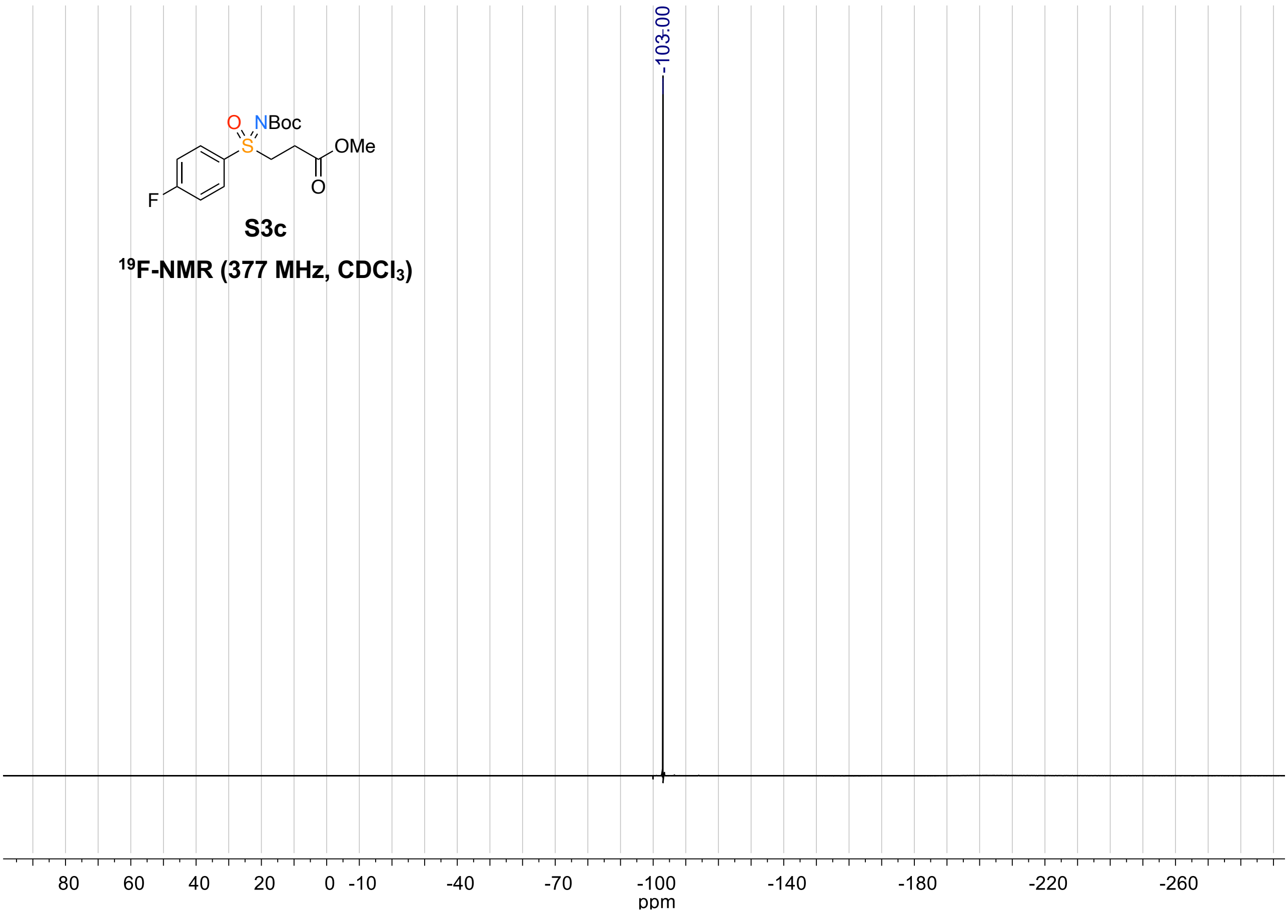
-103.00
O NBoc
S
OMe
O
F
S3c
19F-NMR (377 MHz, CDCl3)
80
60
40
20
0
-10
-40
-70
-100
ppm
-140
-180
-220
-260

Methyl 3-(*N*-(tert-butoxycarbonyl)-4-methoxyphenylsulfonimidoyl)propanoate (S3d)

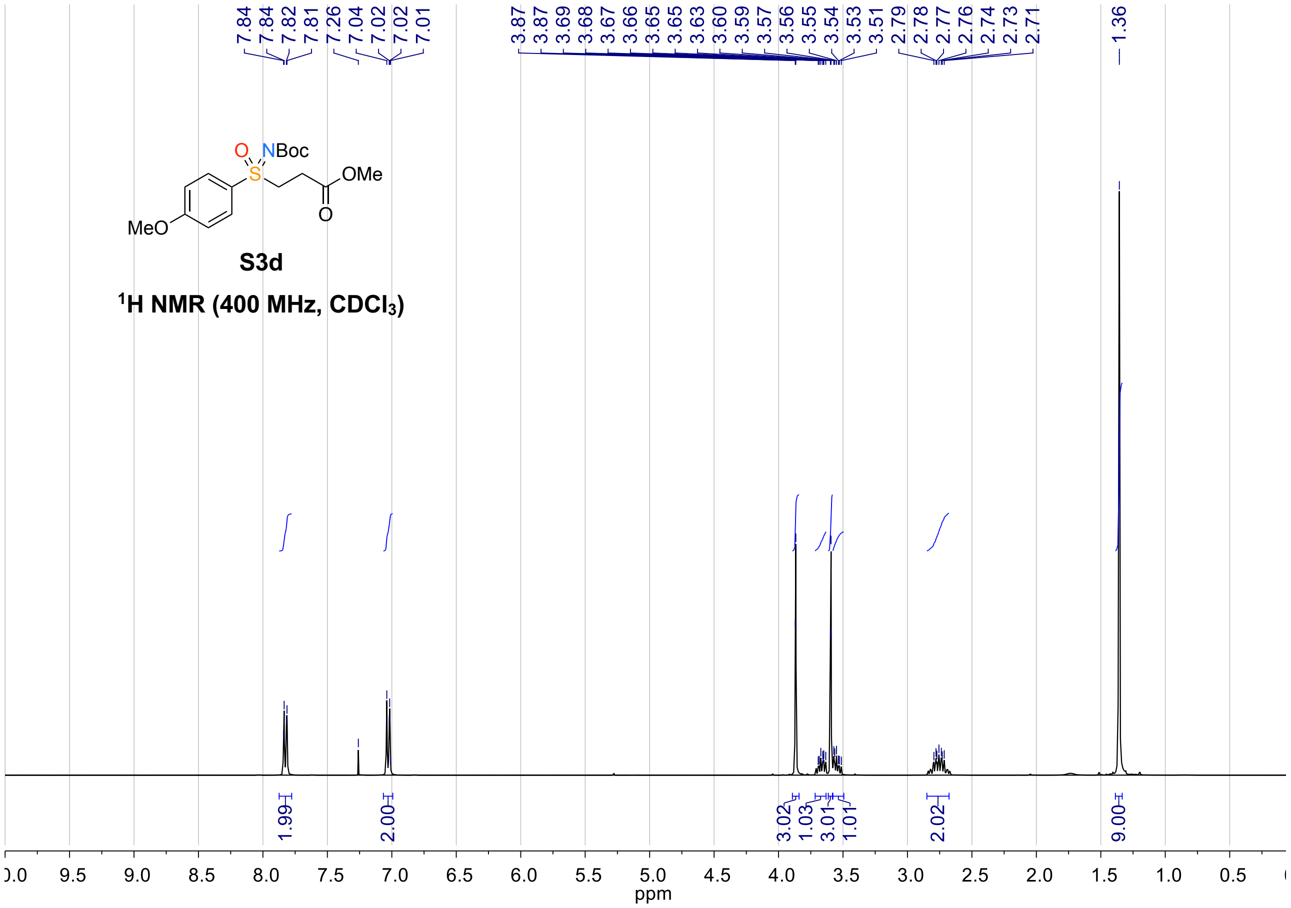

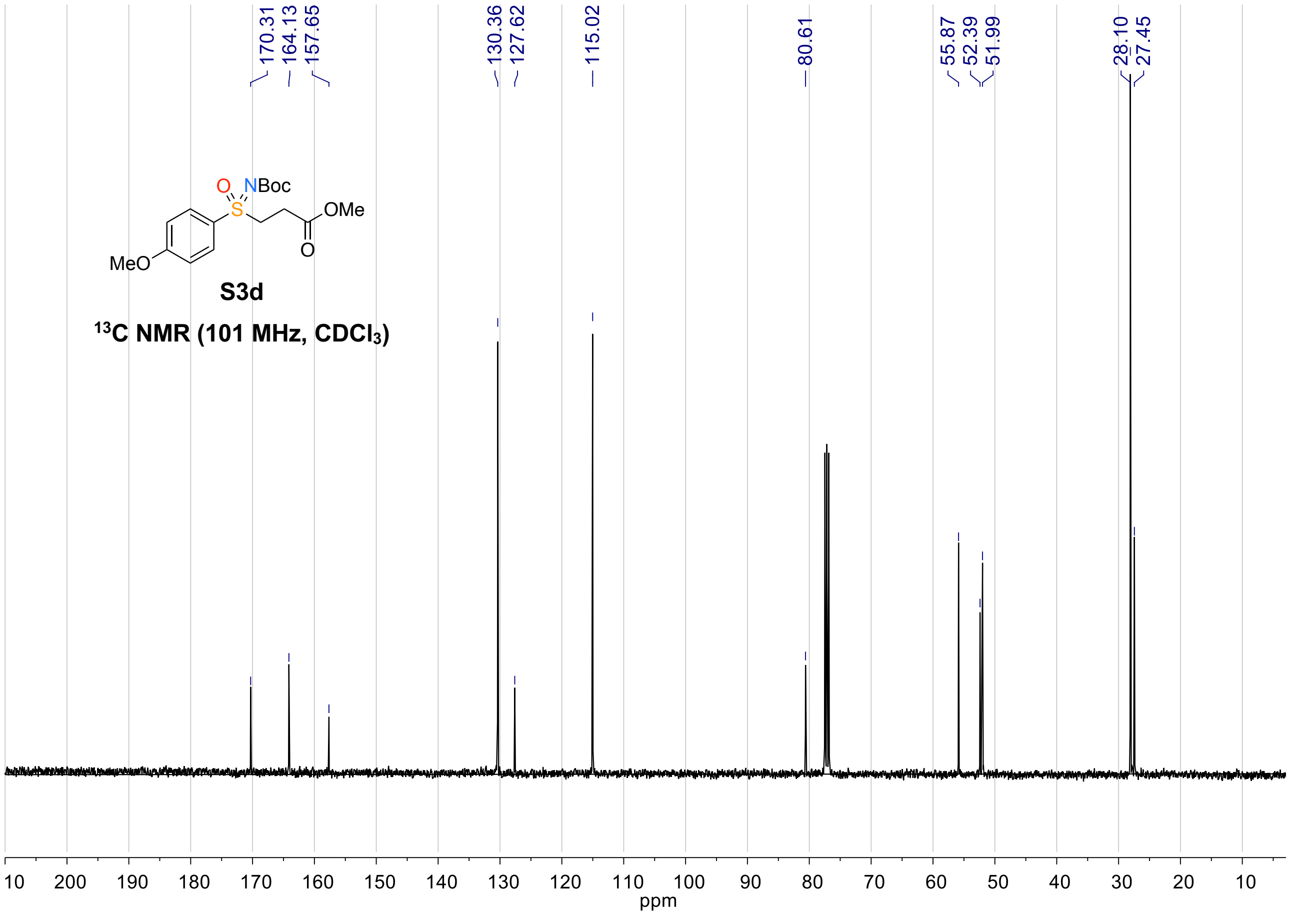

Methyl 3-(*N*-(*tert*-butoxycarbonyl)pyridine-2-sulfonimidoyl)propanoate (S3e)

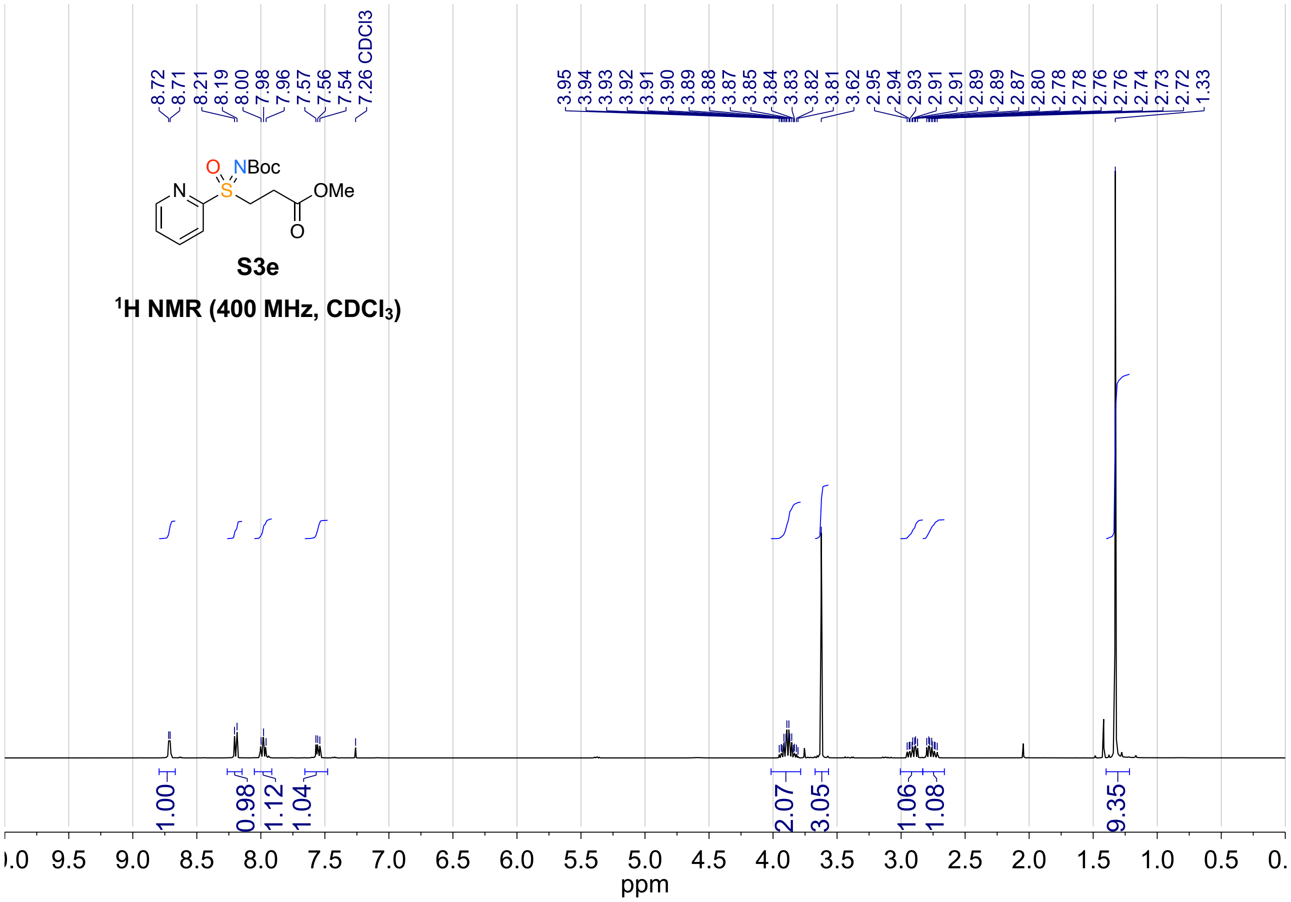

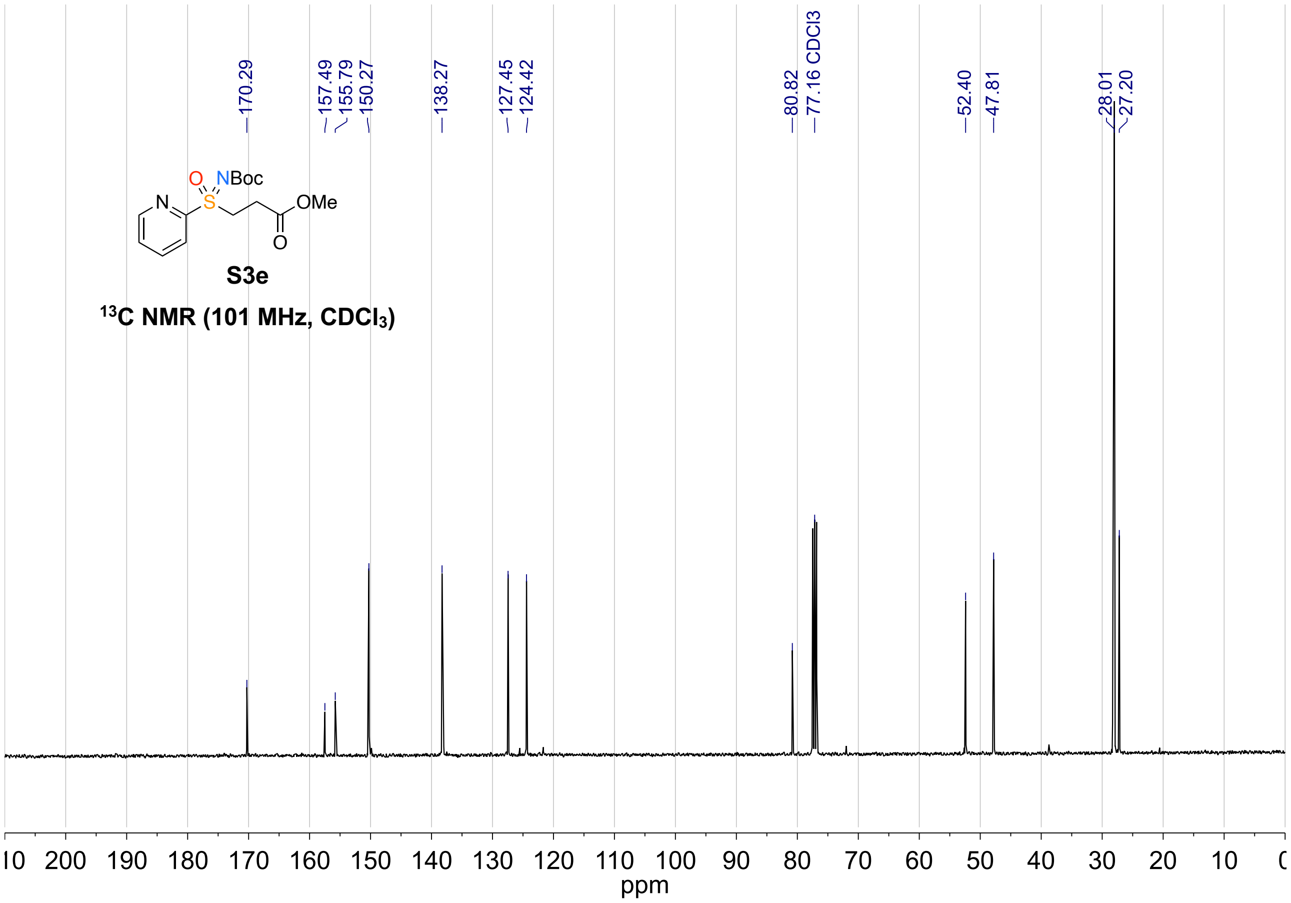

Methyl 3-(*N*-(*tert*-butoxycarbonyl)propan-2-ylsulfonimidoyl)propanoate (S3f)

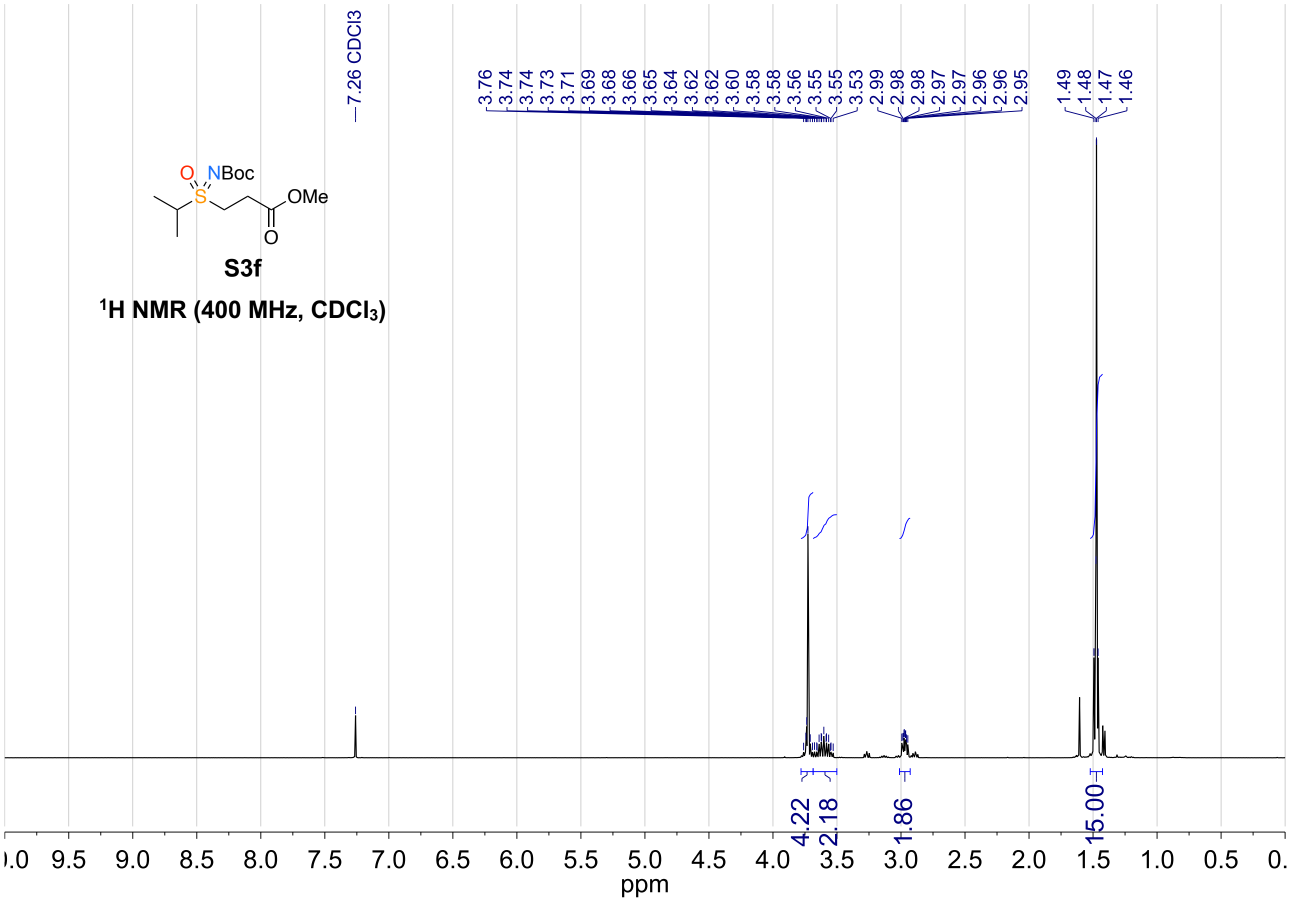

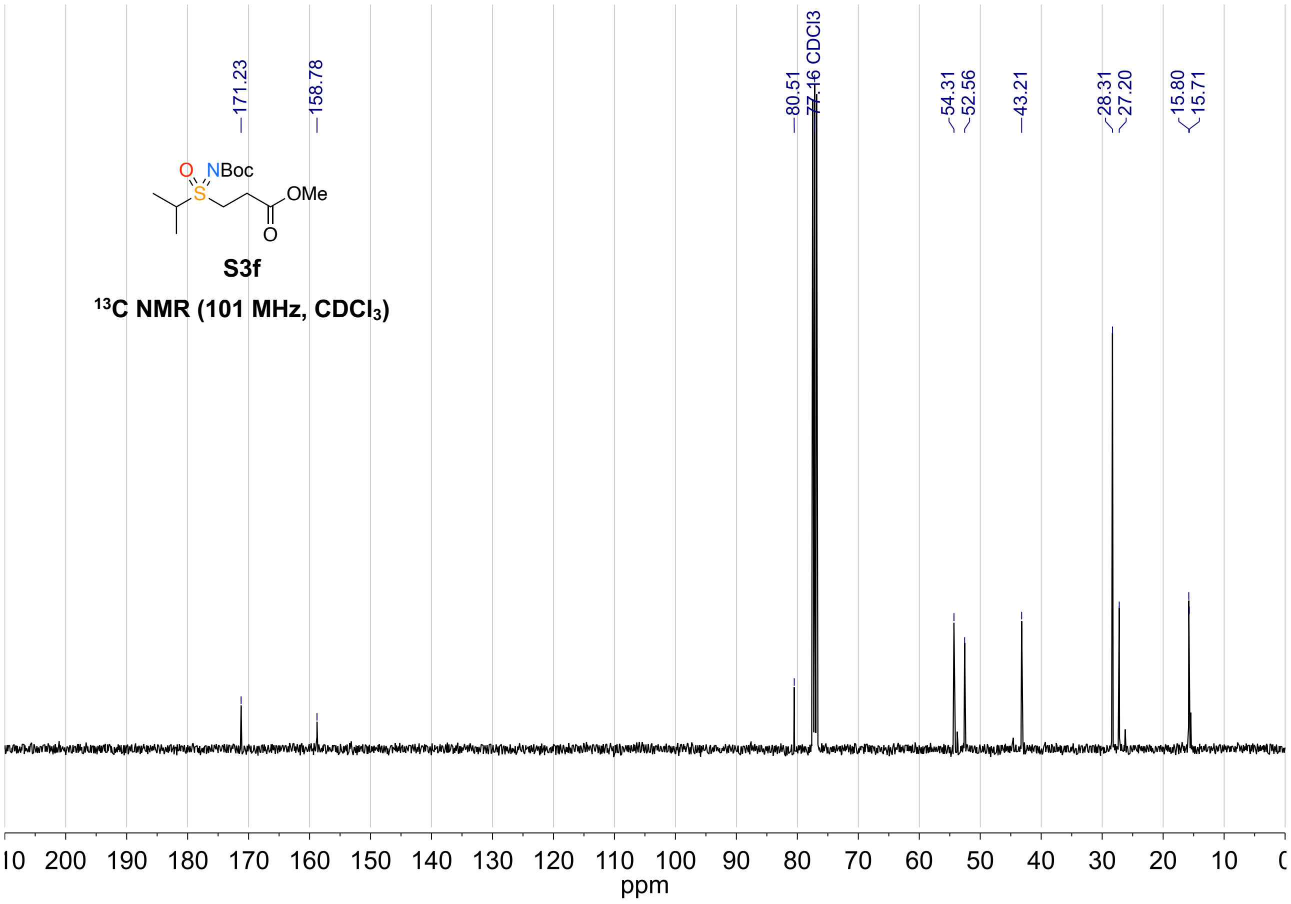

Methyl 3-(*N*-(*tert*-butoxycarbonyl)-*S*-methylsulfonimidoyl)propanoate (S3g)

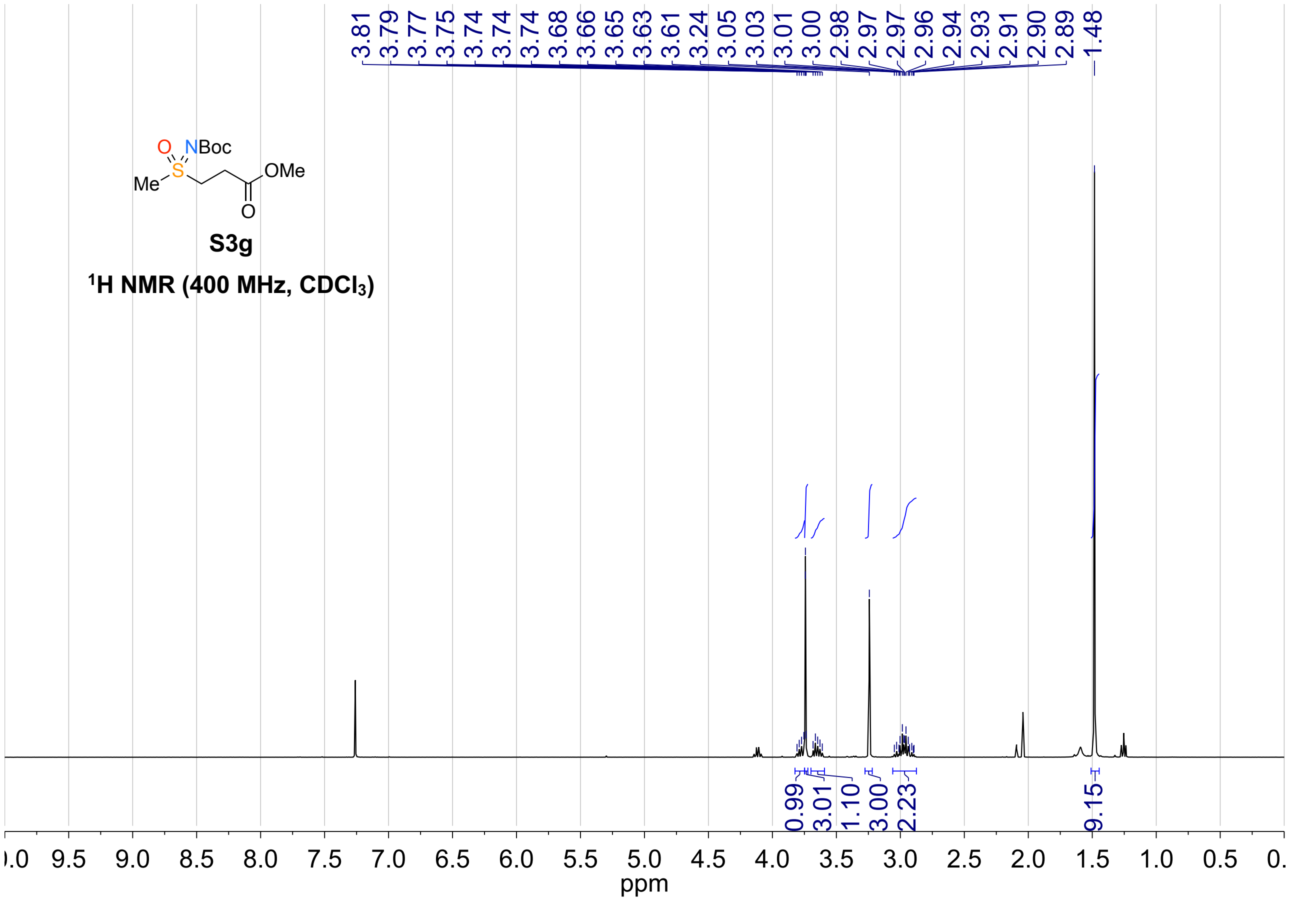

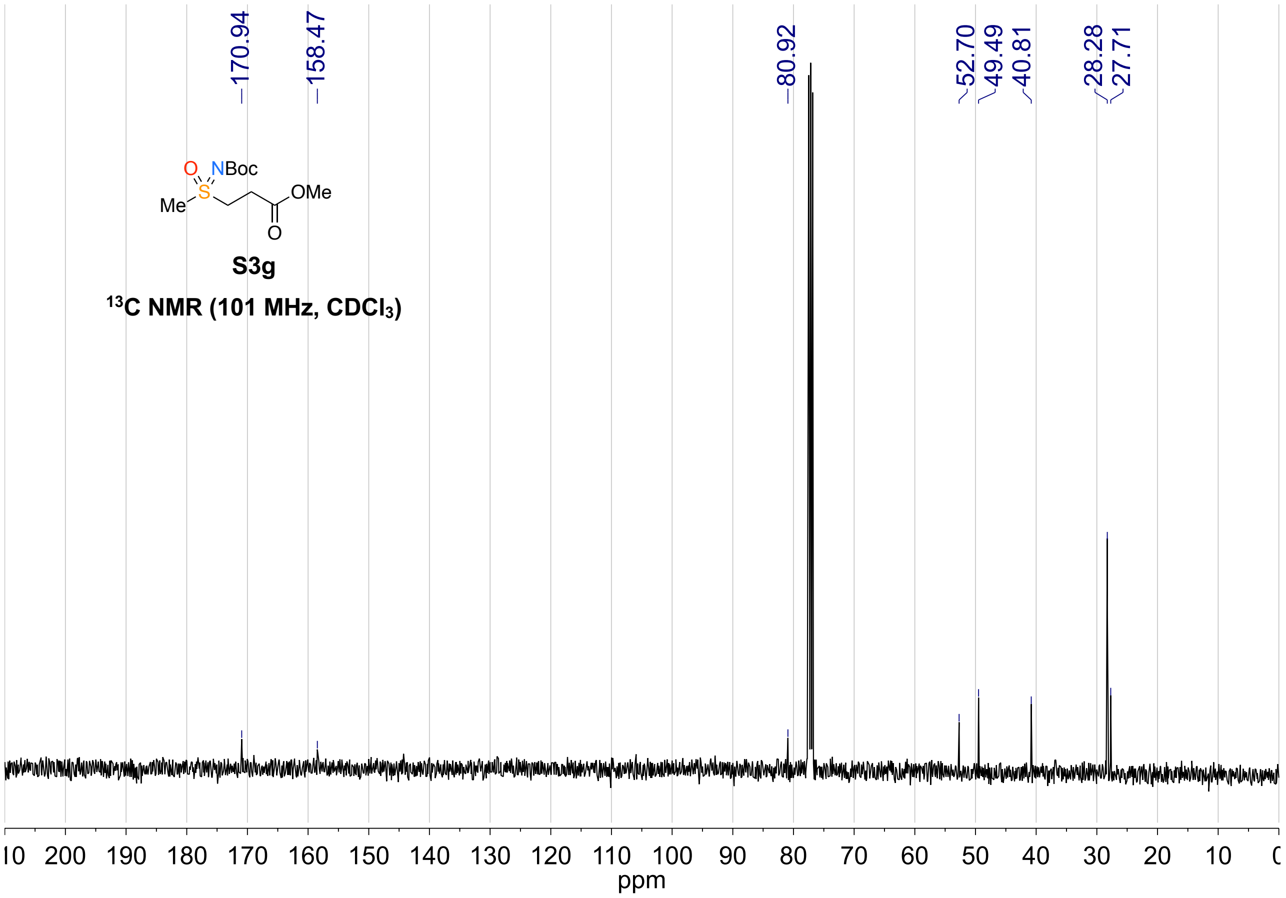

Sodium (*tert*-butoxycarbonyl)(phenylsulfinyl)amide (S4b)

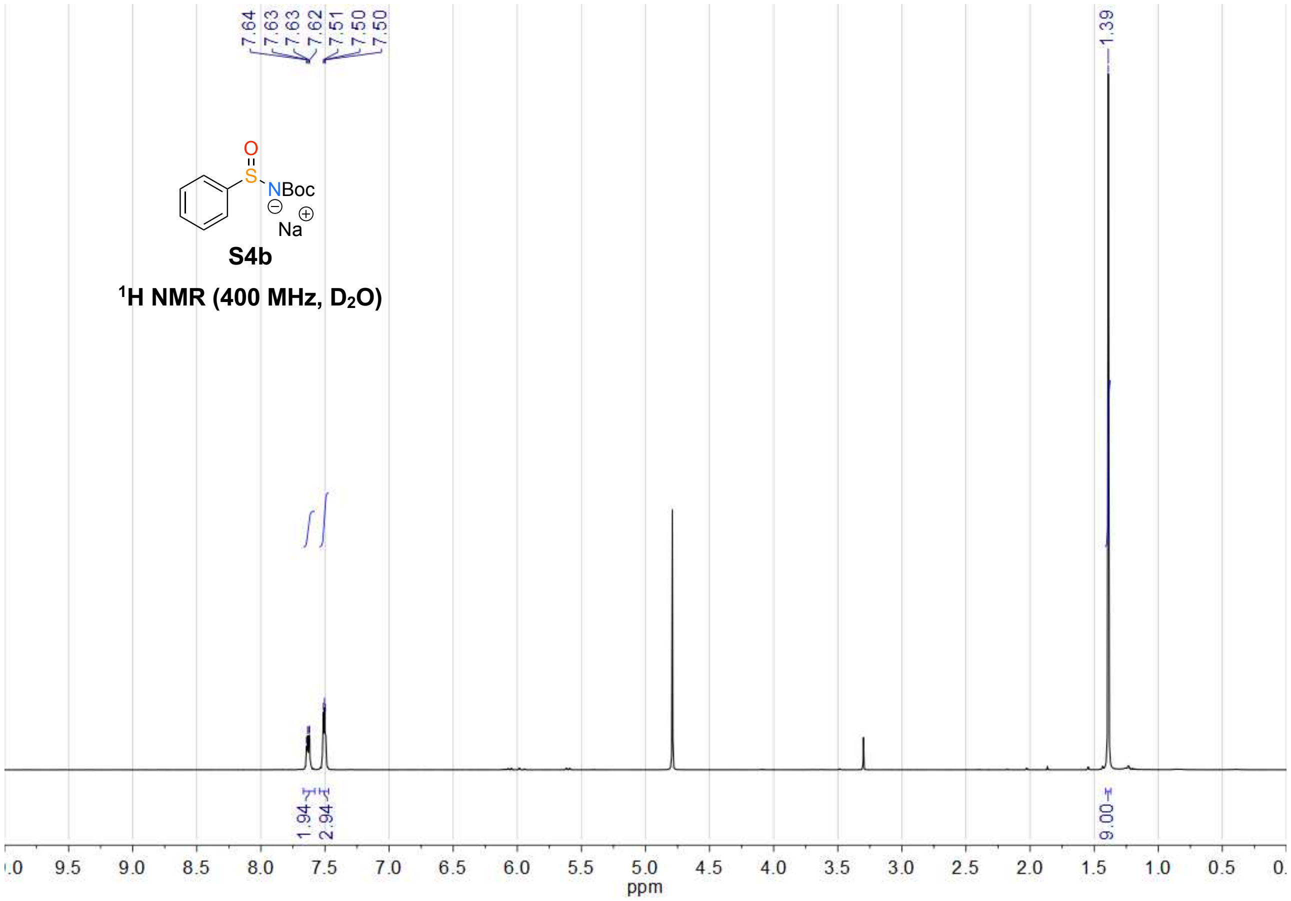

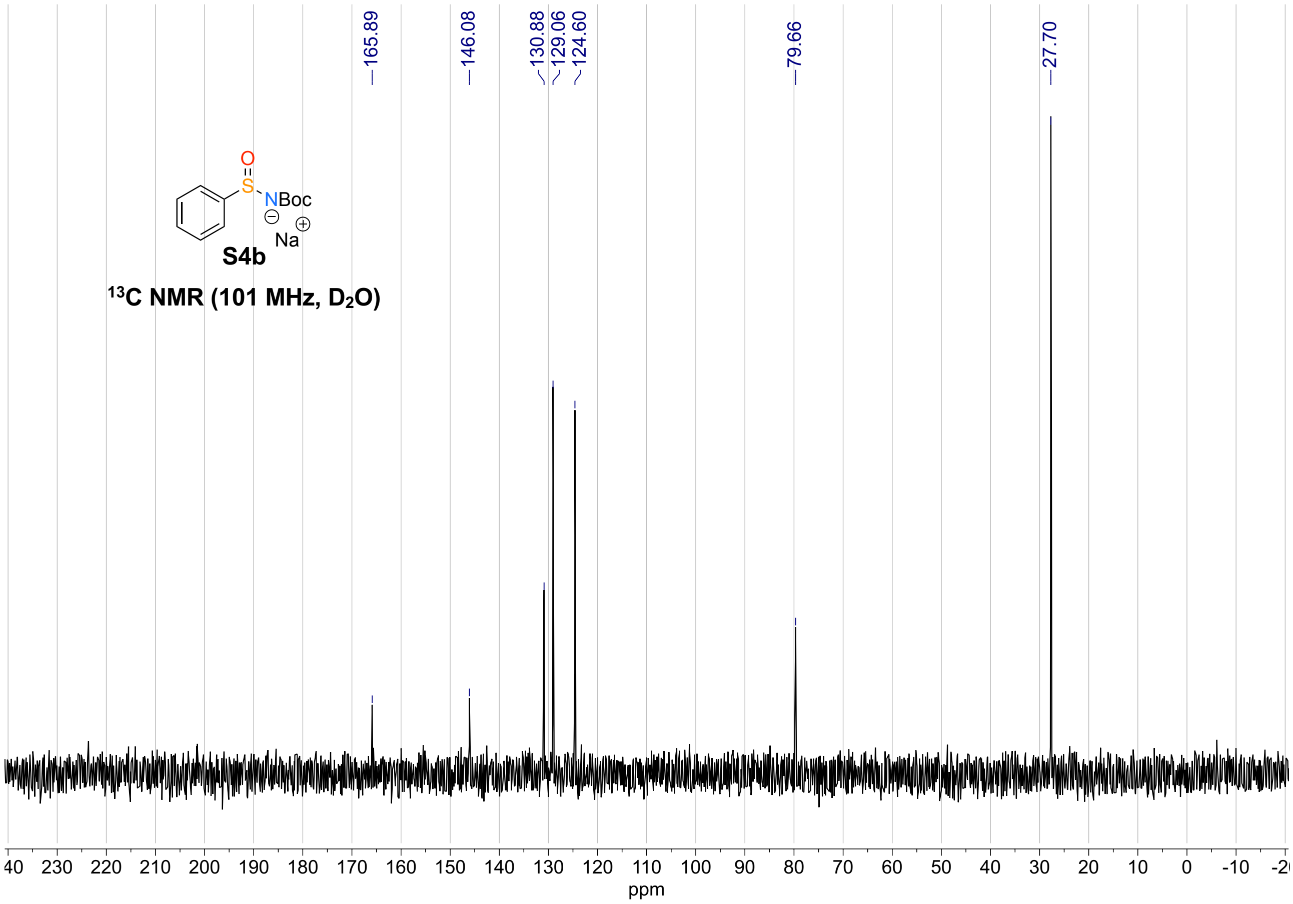

Sodium (*tert*-butoxycarbonyl)((4-fluorophenyl)sulfinyl)amide S4c

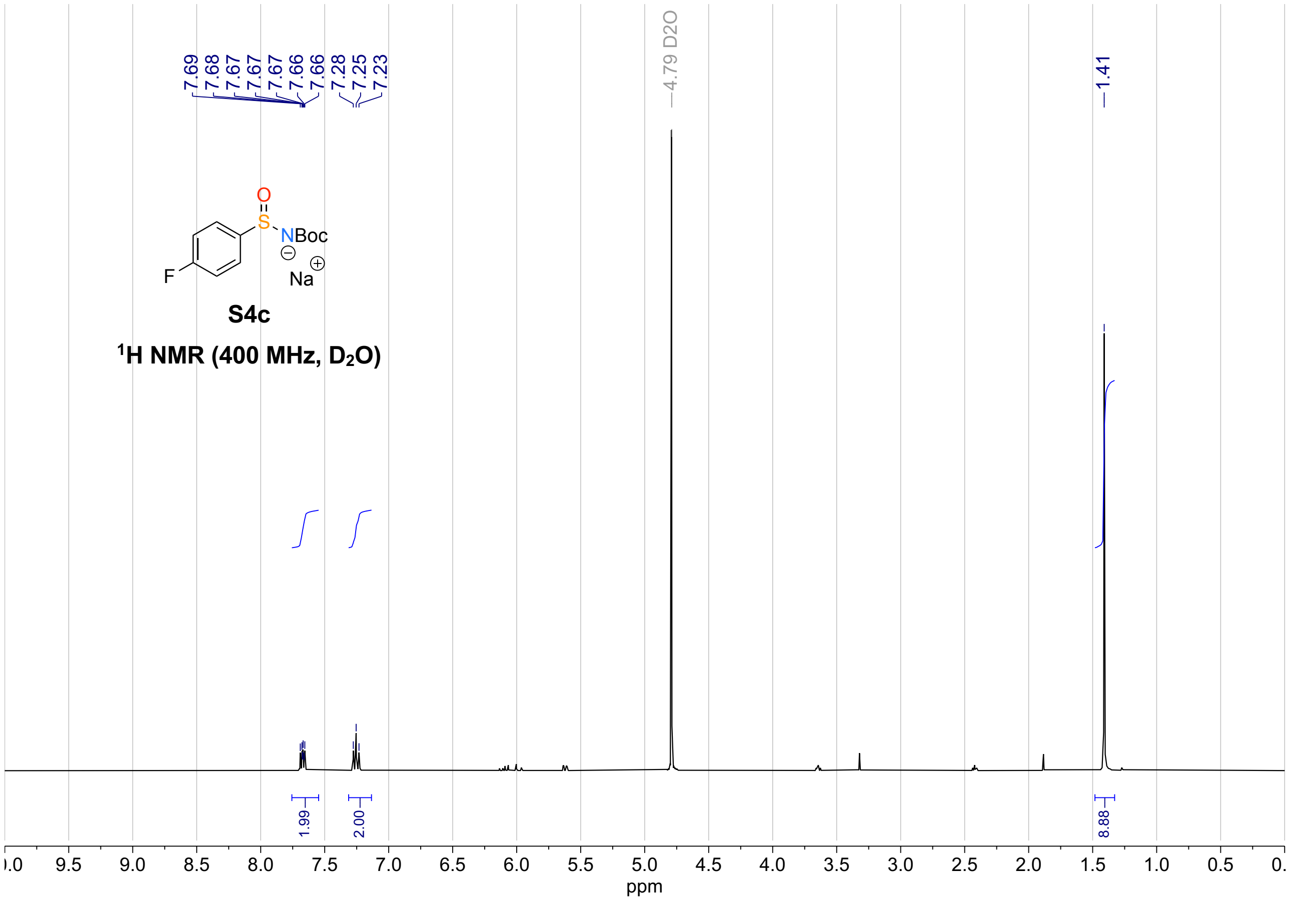

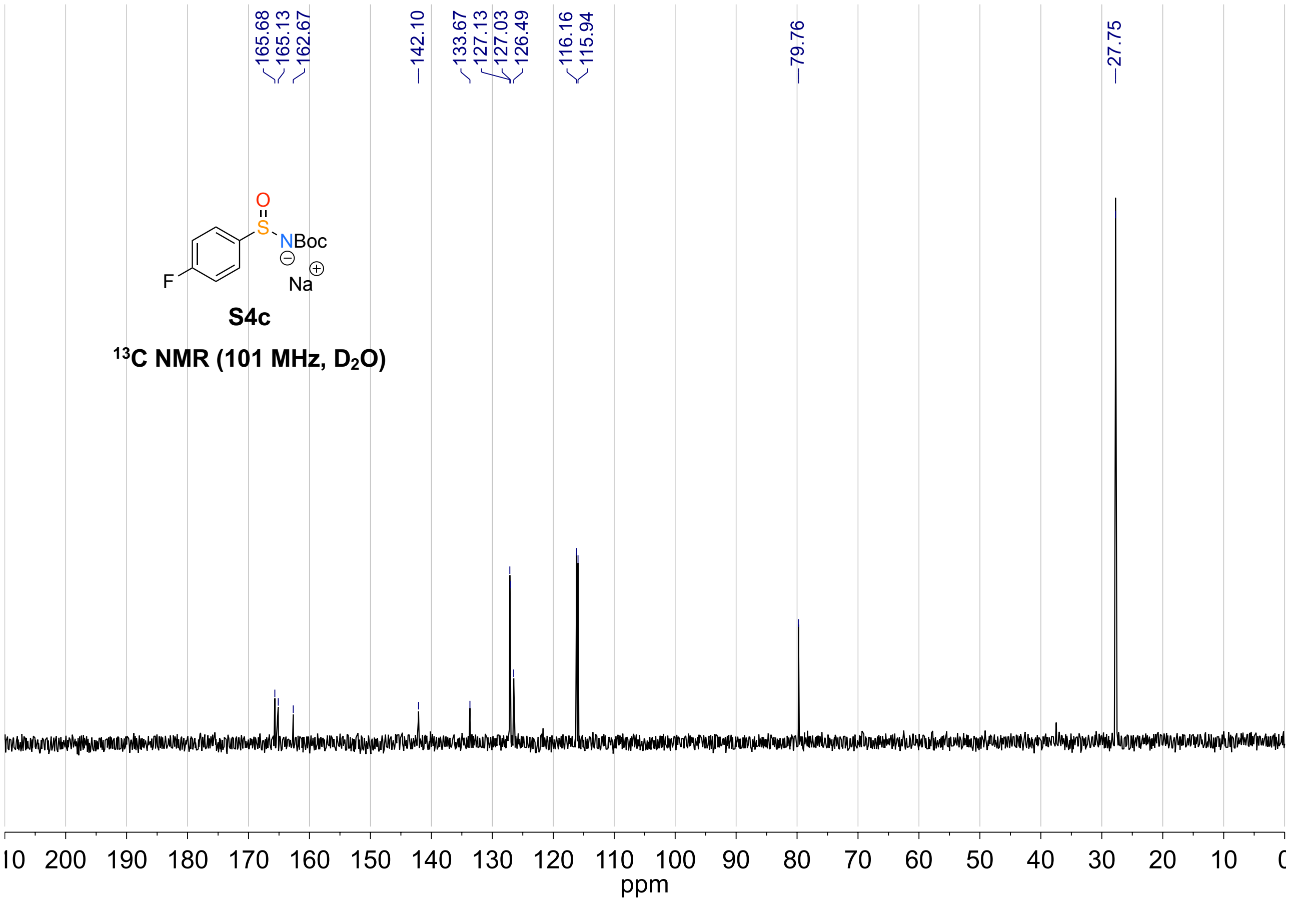

Sodium (*tert*-butoxycarbonyl)((4-methoxyphenyl)sulfinyl)amide (S4d)

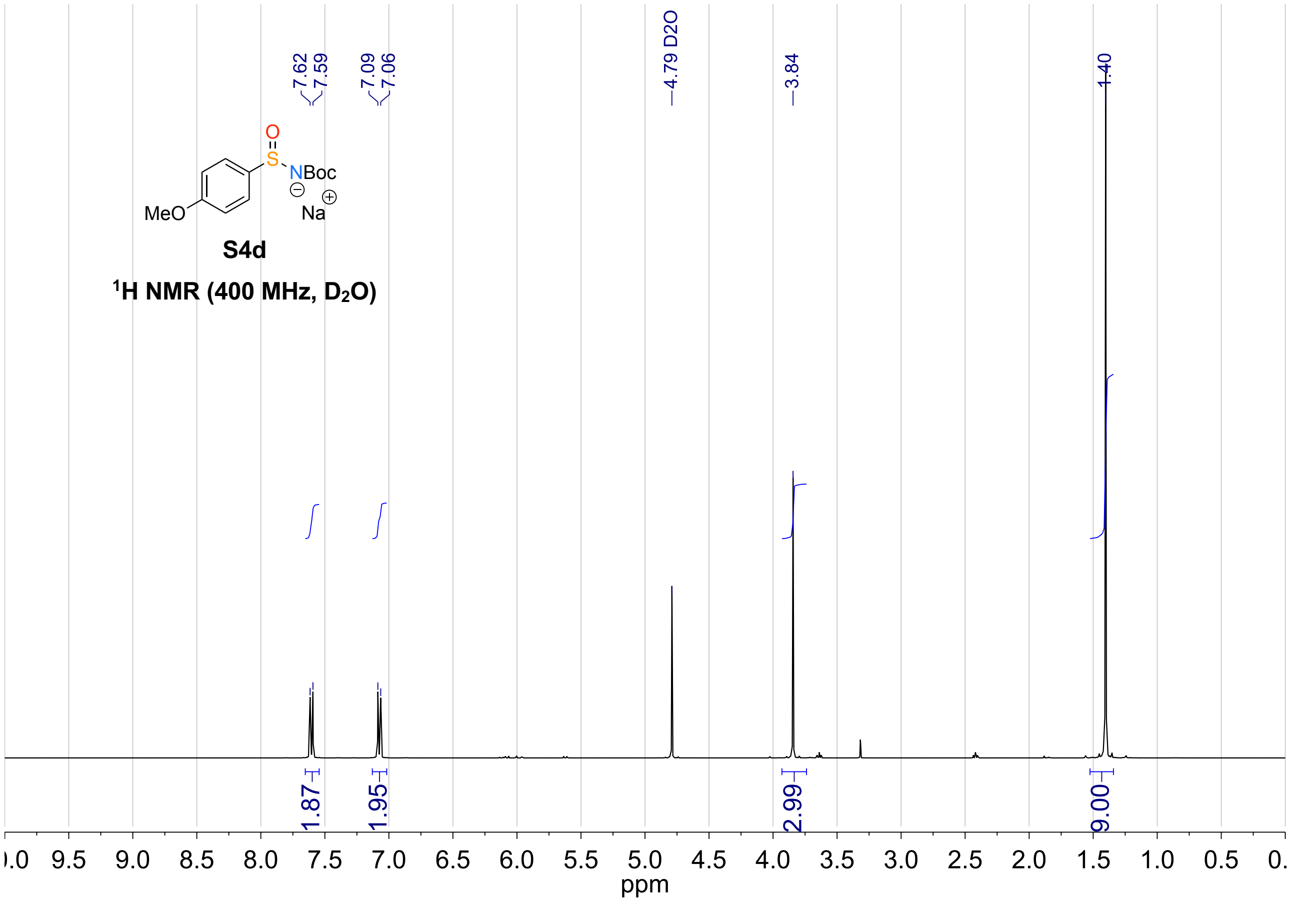

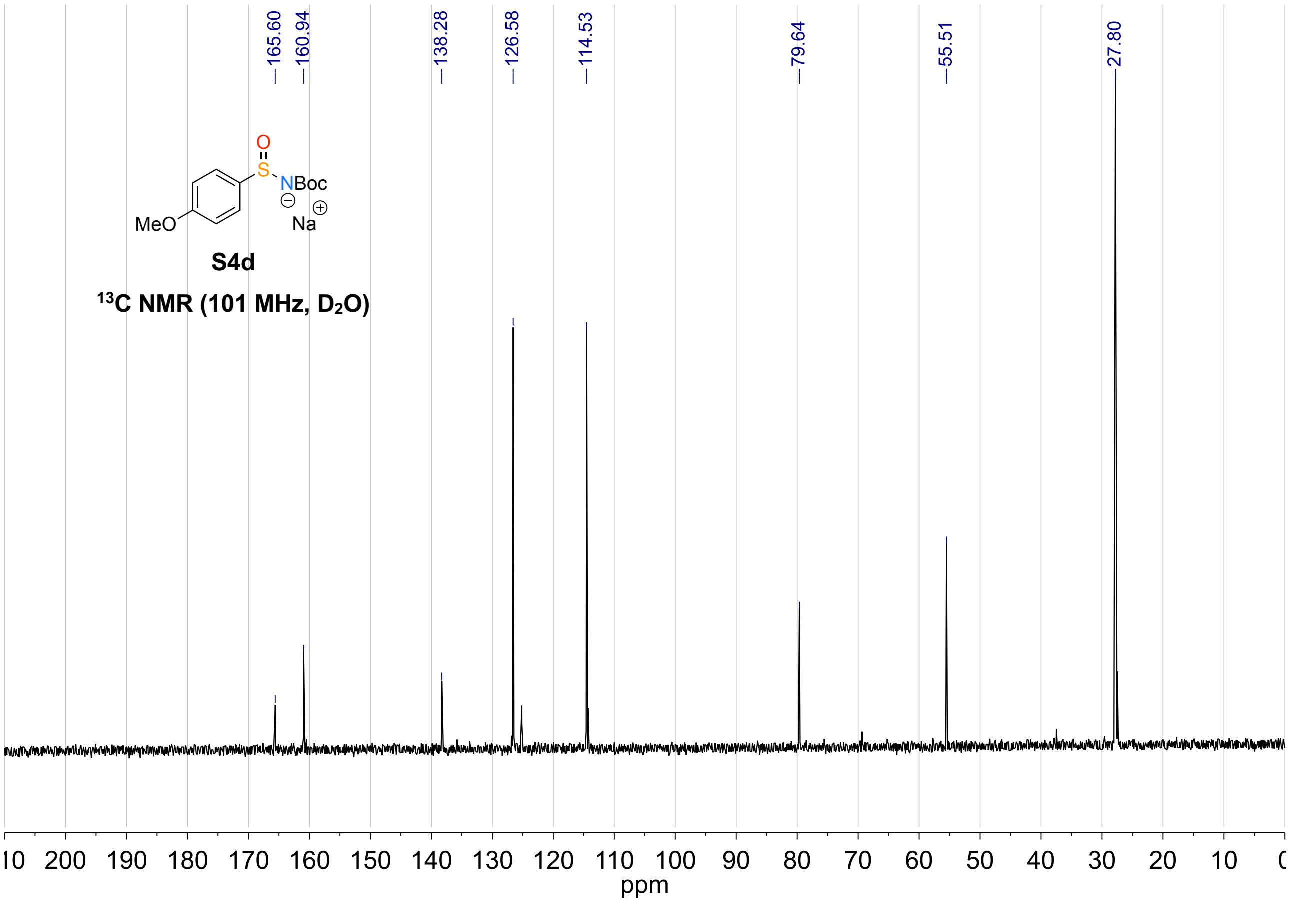

Sodium (*tert*-butoxycarbonyl)(pyridin-2-ylsulfinyl)amide (S4e)

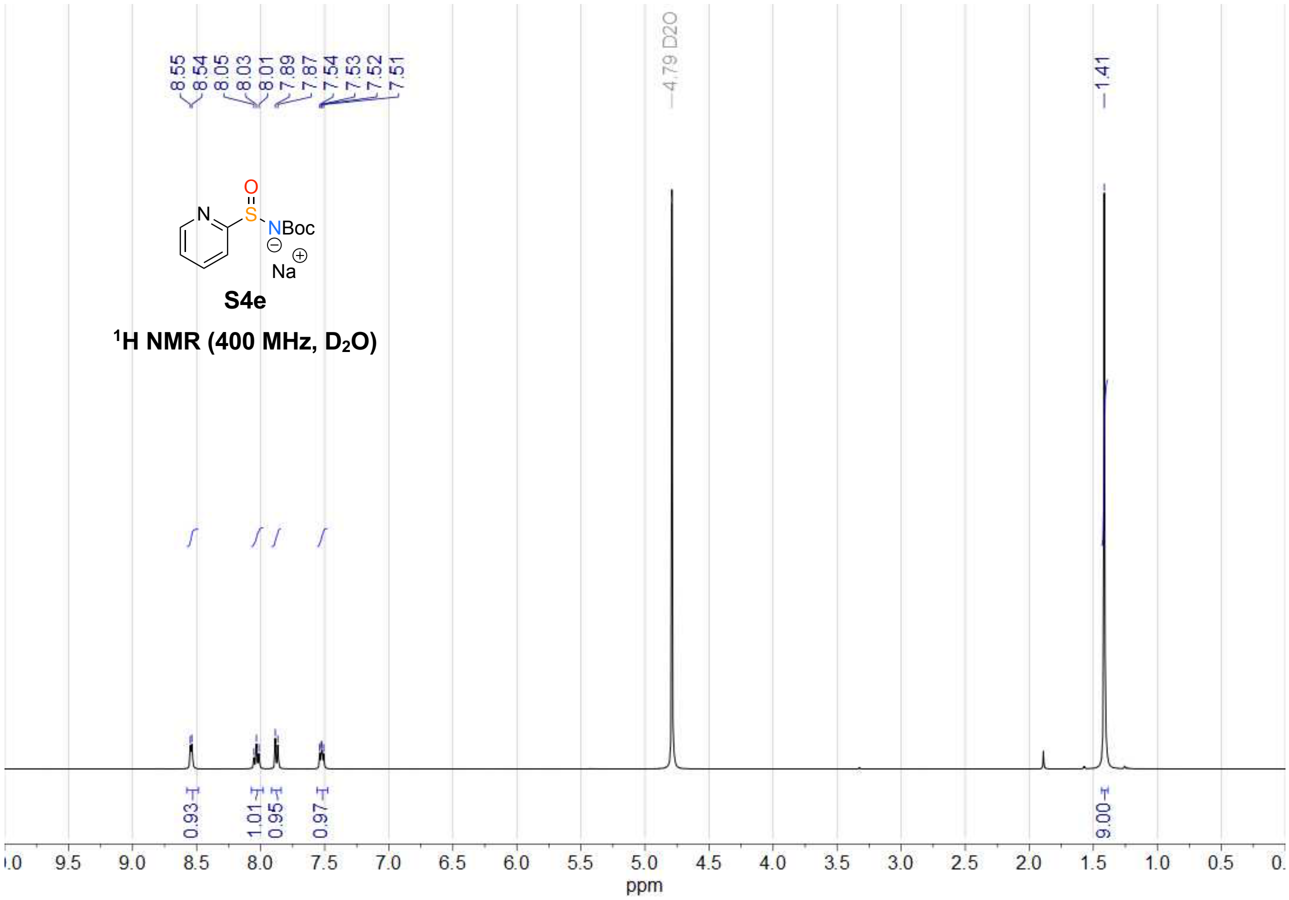

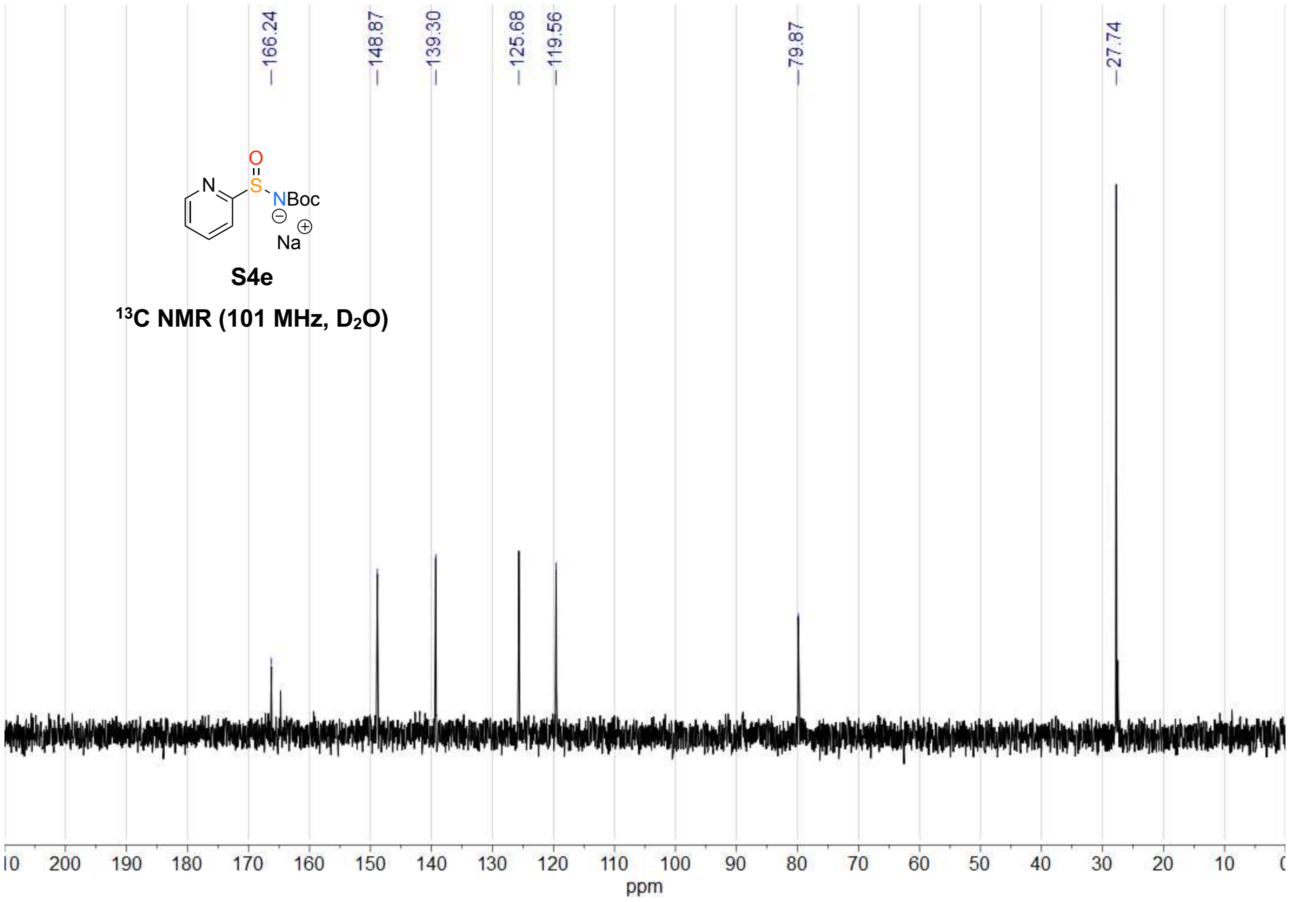

Sodium (*tert*-butoxycarbonyl)(isopropylsulfinyl)amide (S4f)

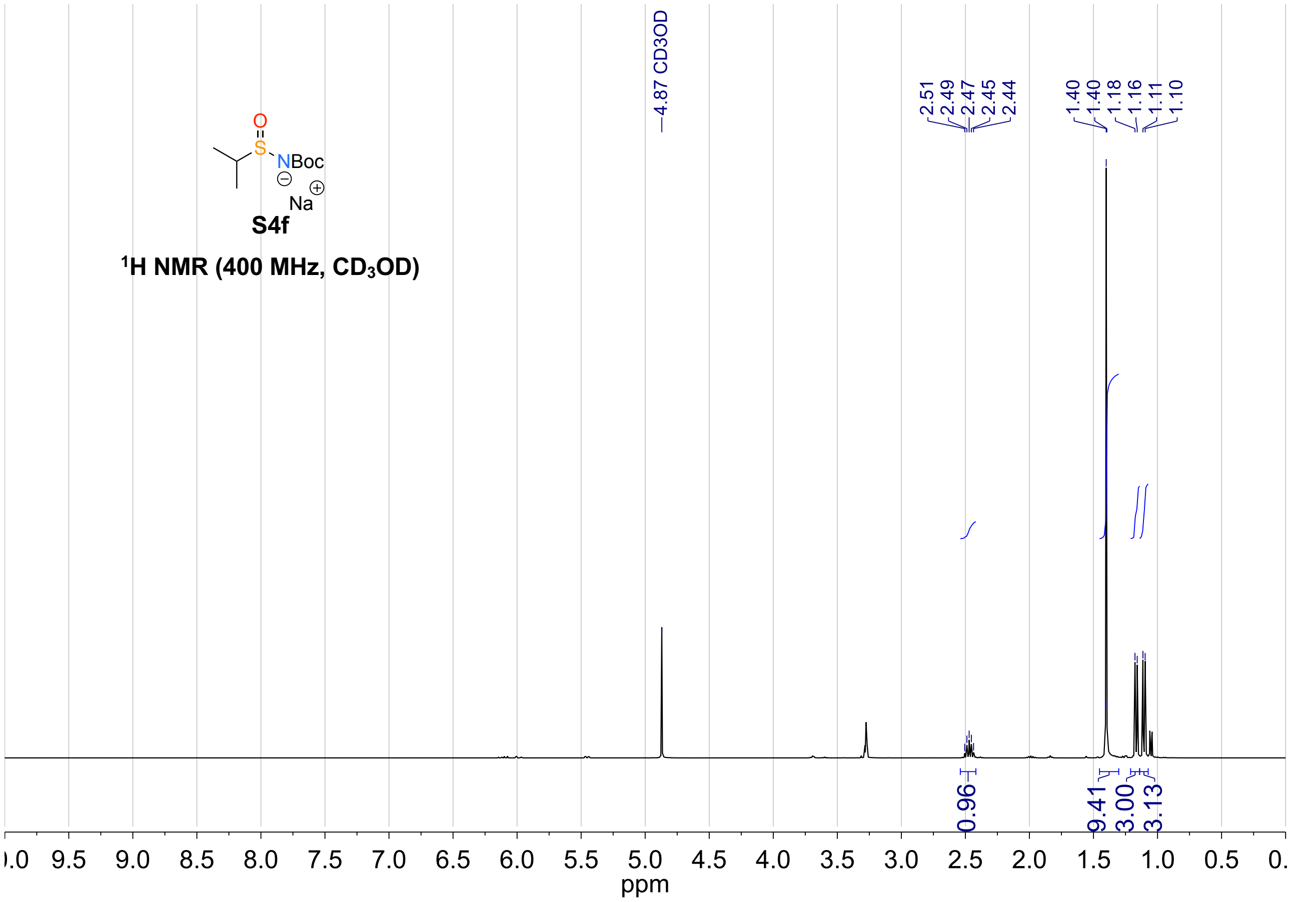

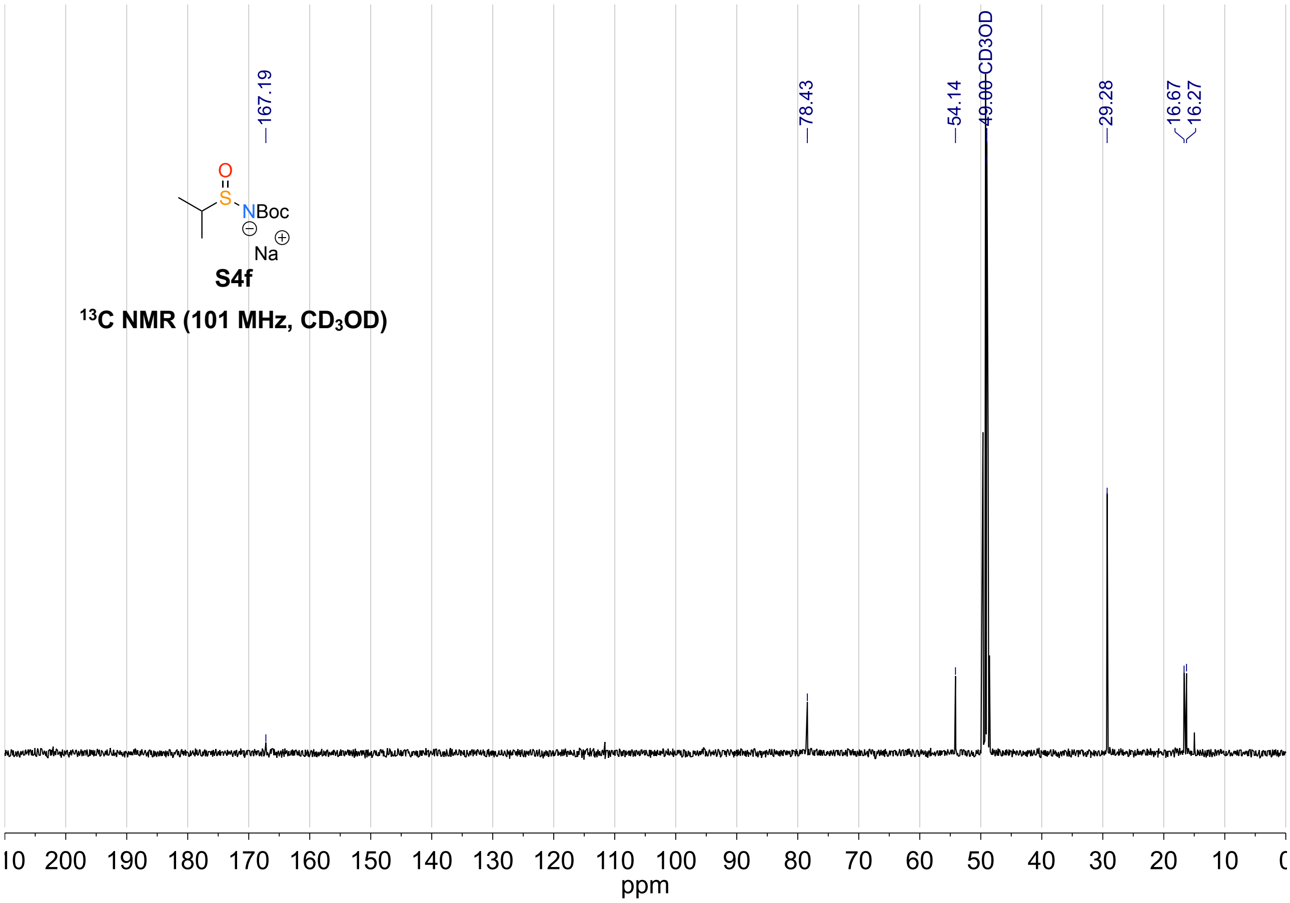

Sodium (*tert*-butoxycarbonyl)(methylsulfinyl)amide (S4g)

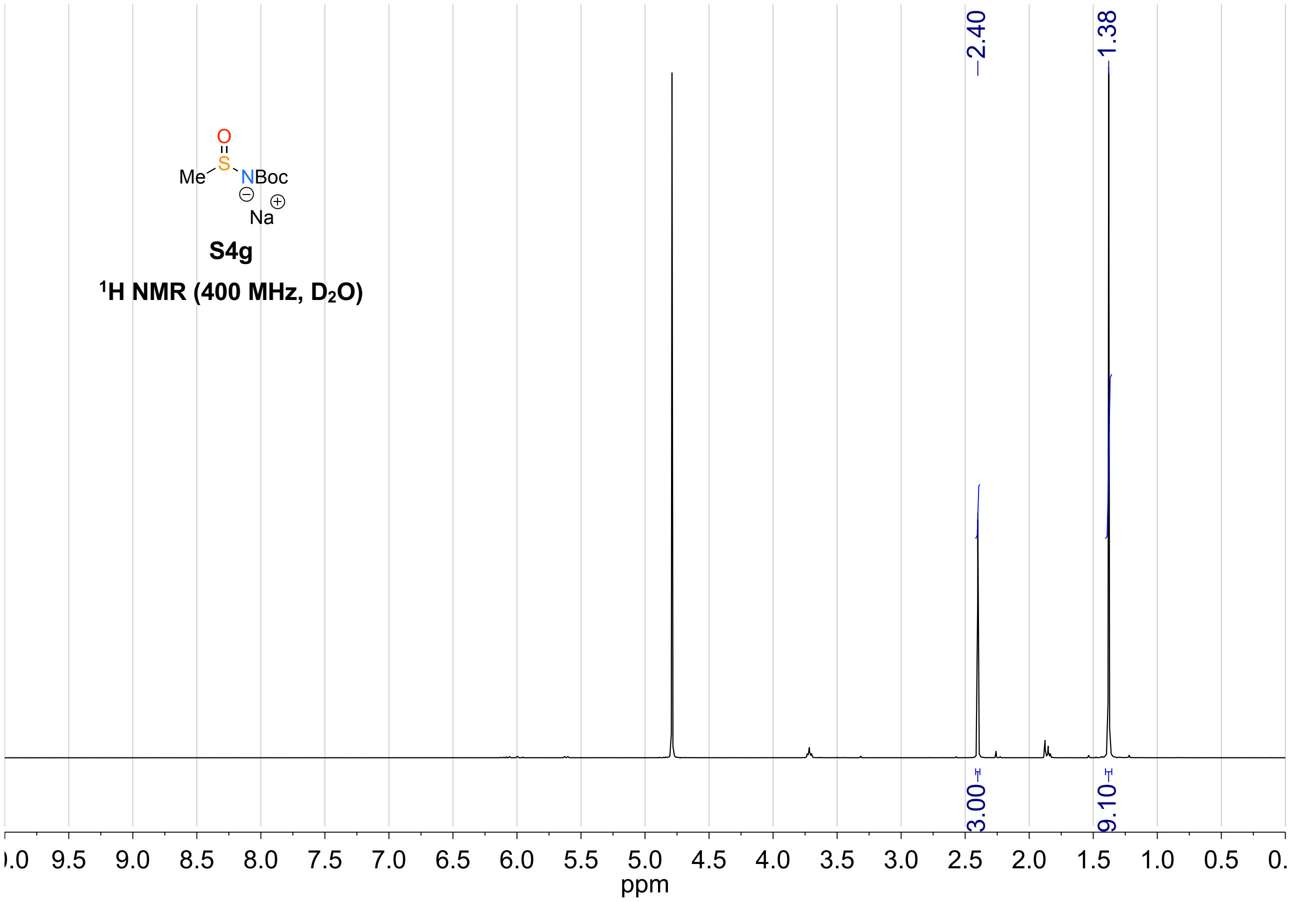

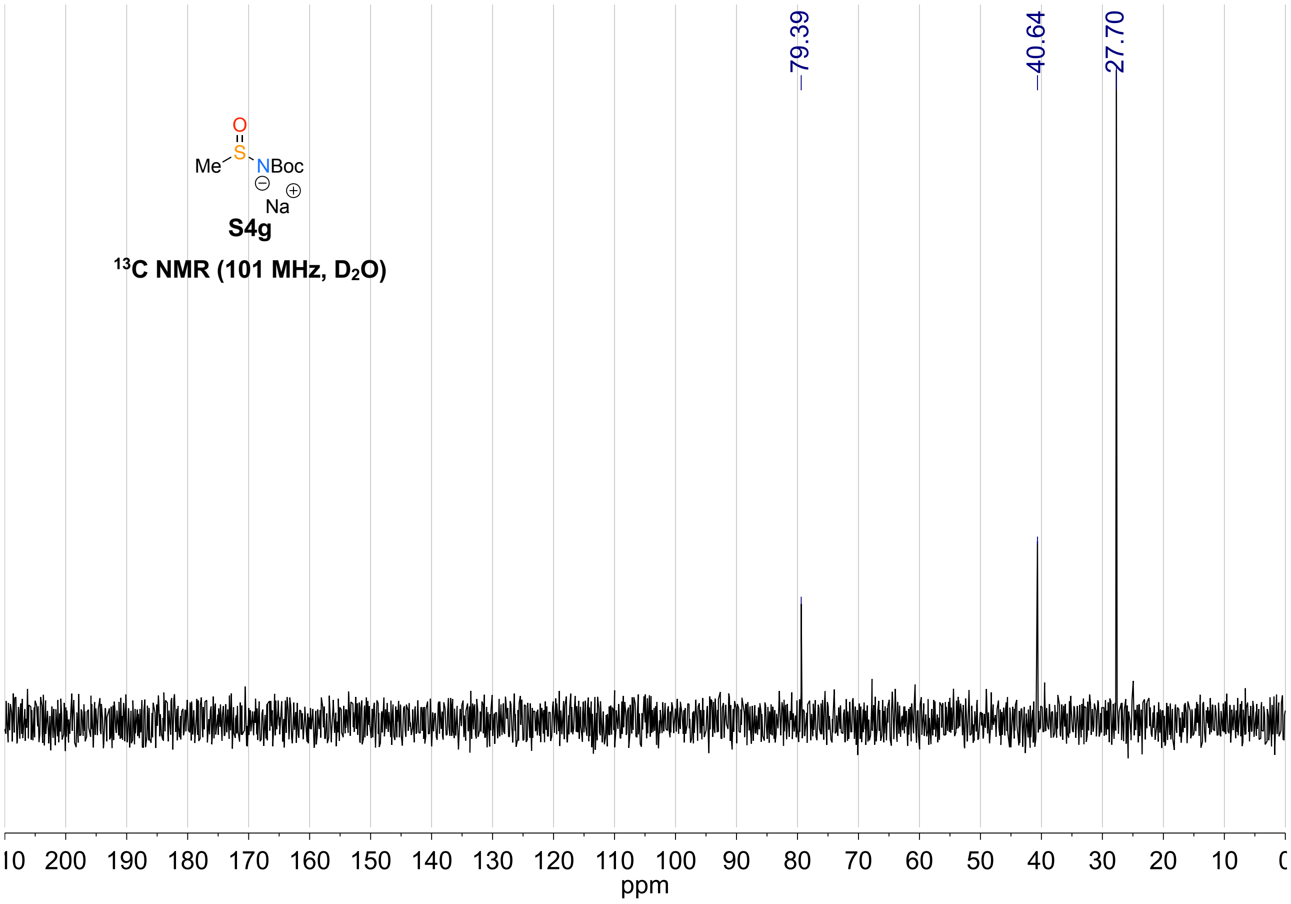

tert-Butyl (fluoro(oxo)(phenyl)-λ^6-sulfaneylidene)carbamate (2c)

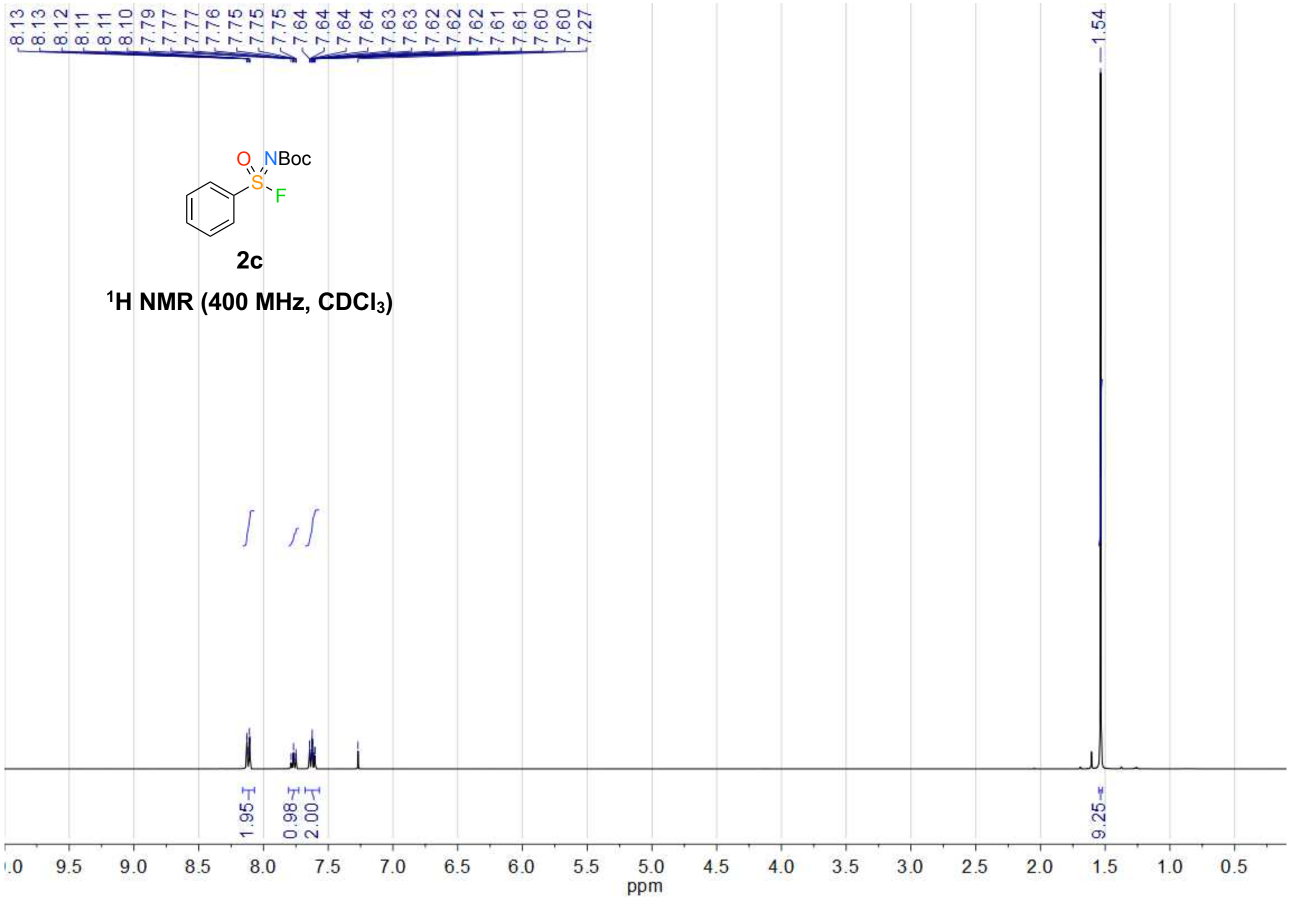

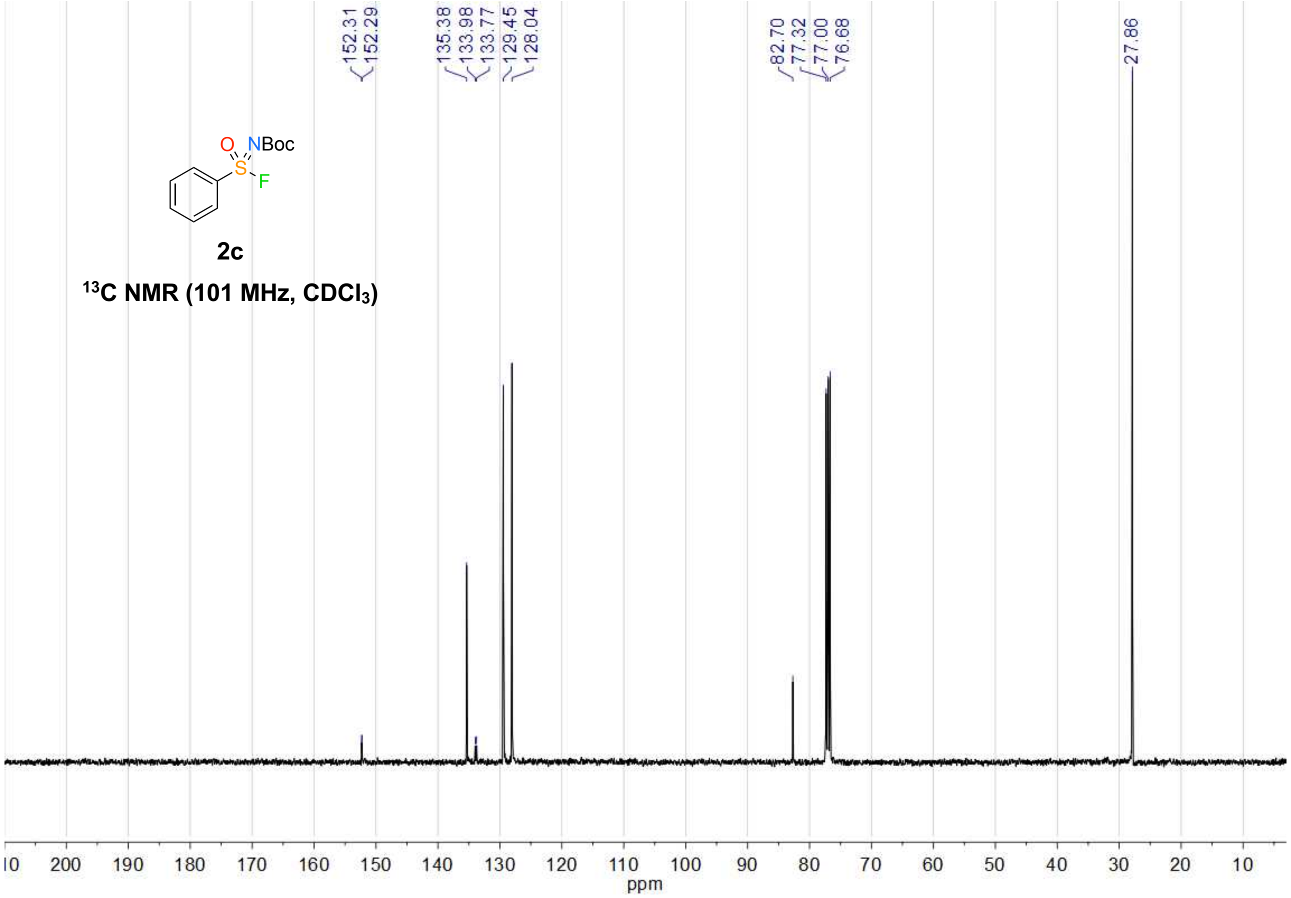

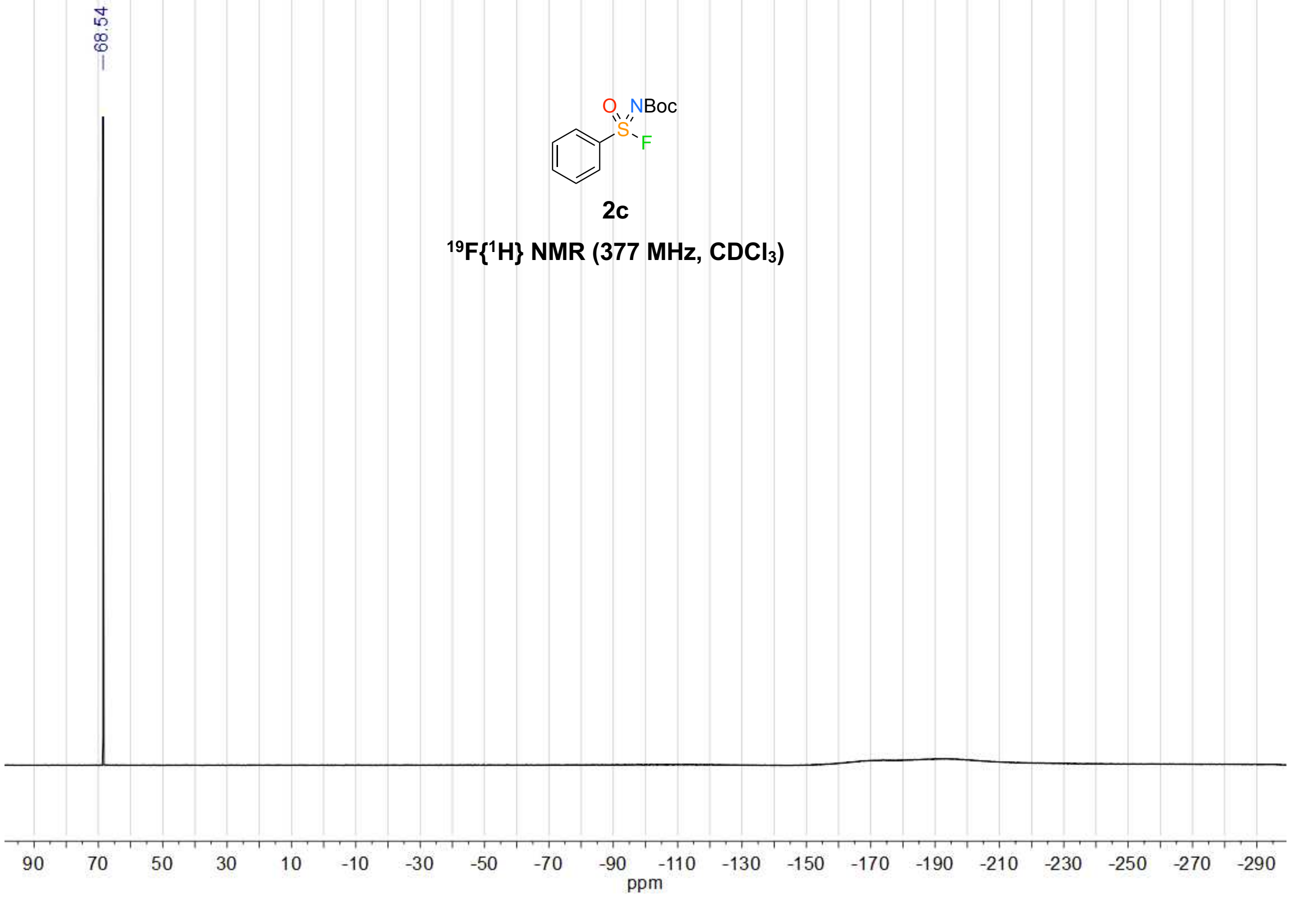
68.54
NBoc
O
S
F
2c
$^{19}F\{^1H\}$ NMR (377 MHz, $CDCl_3$)
90 70 50 30 10 -10 -30 -50 -70 -90 -110 -130 -150 -170 -190 -210 -230 -250 -270 -290
ppm

tert-Butyl (fluoro(4-fluorophenyl)(oxo)-λ⁶-sulfaneylidene)carbamate (2d)

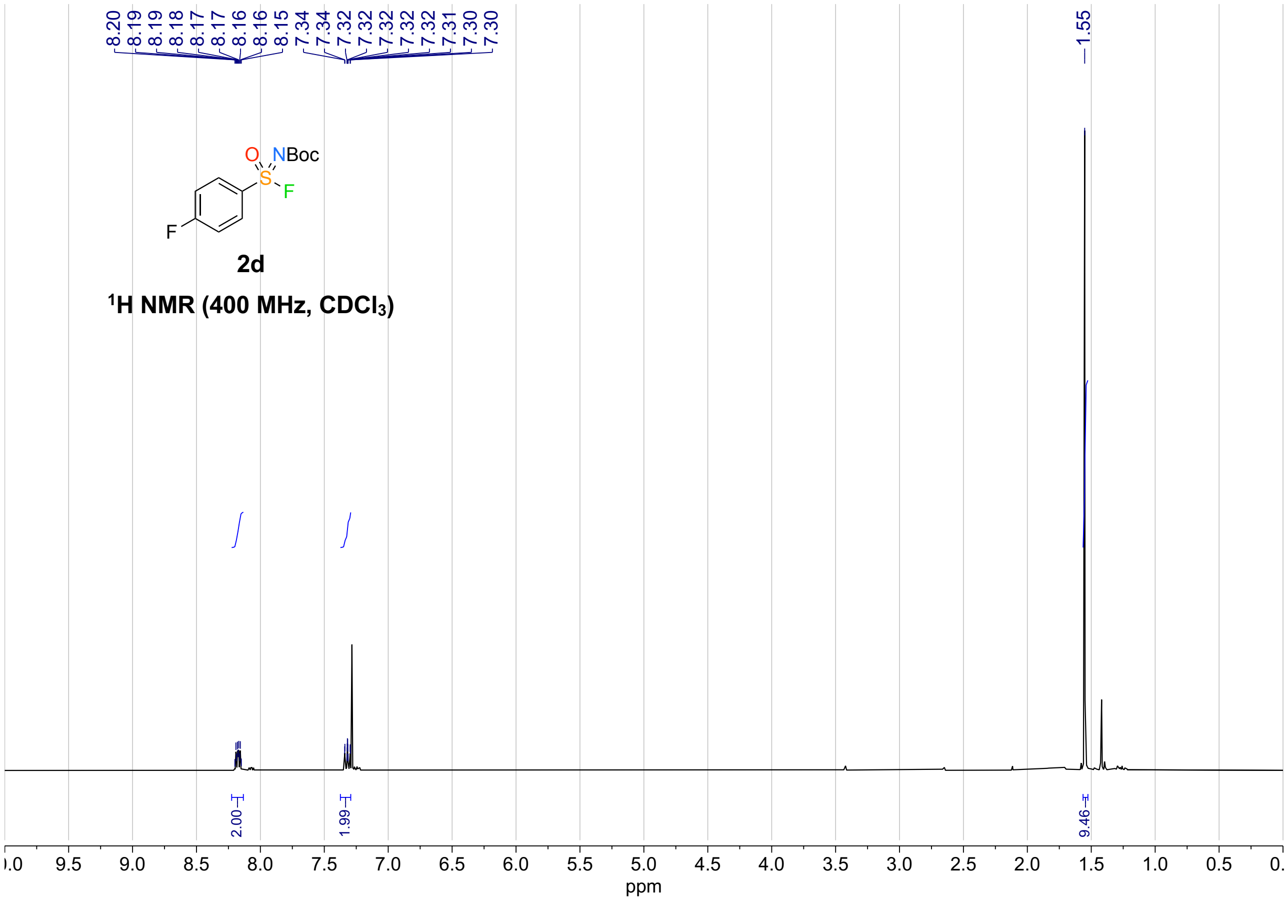

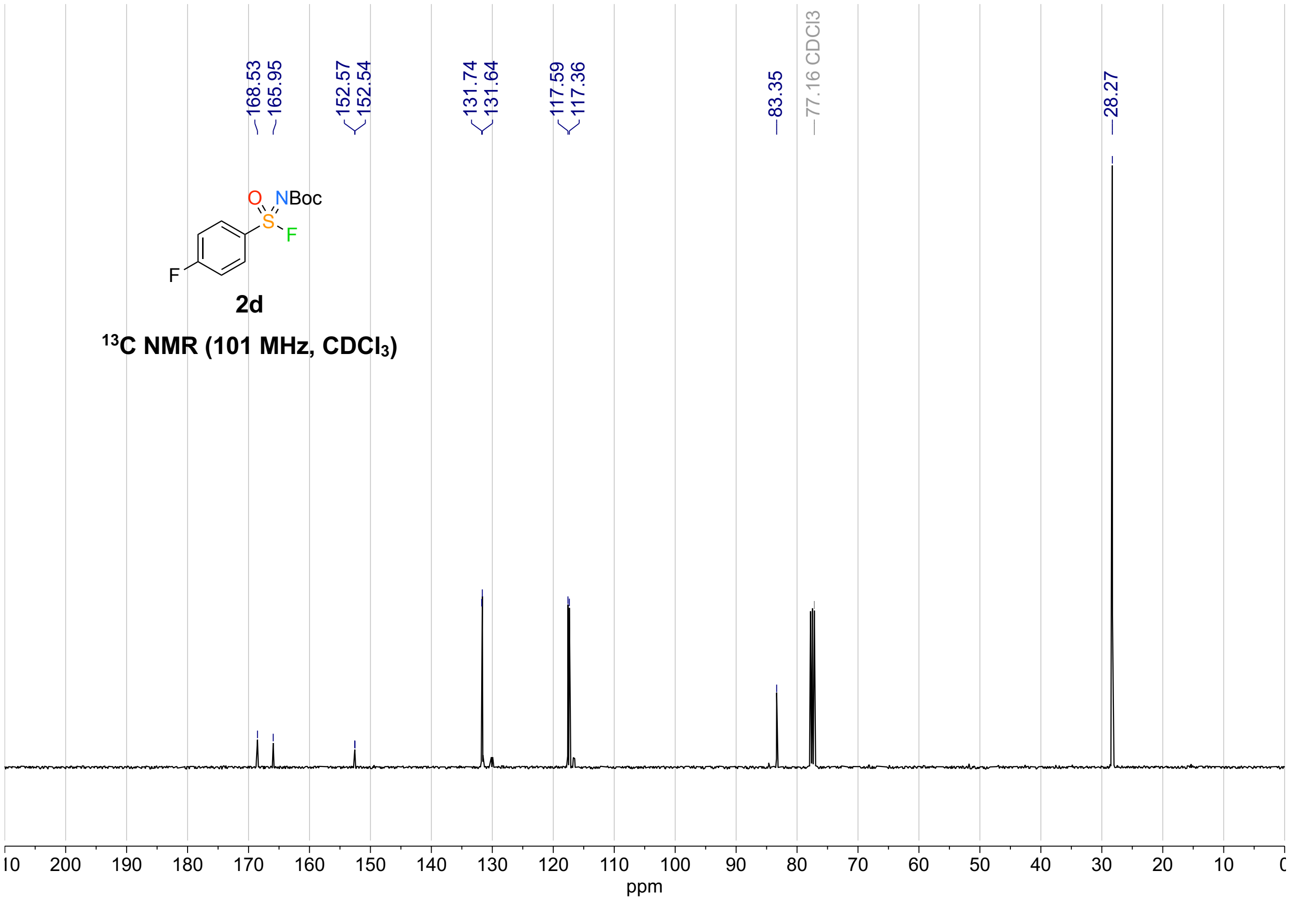

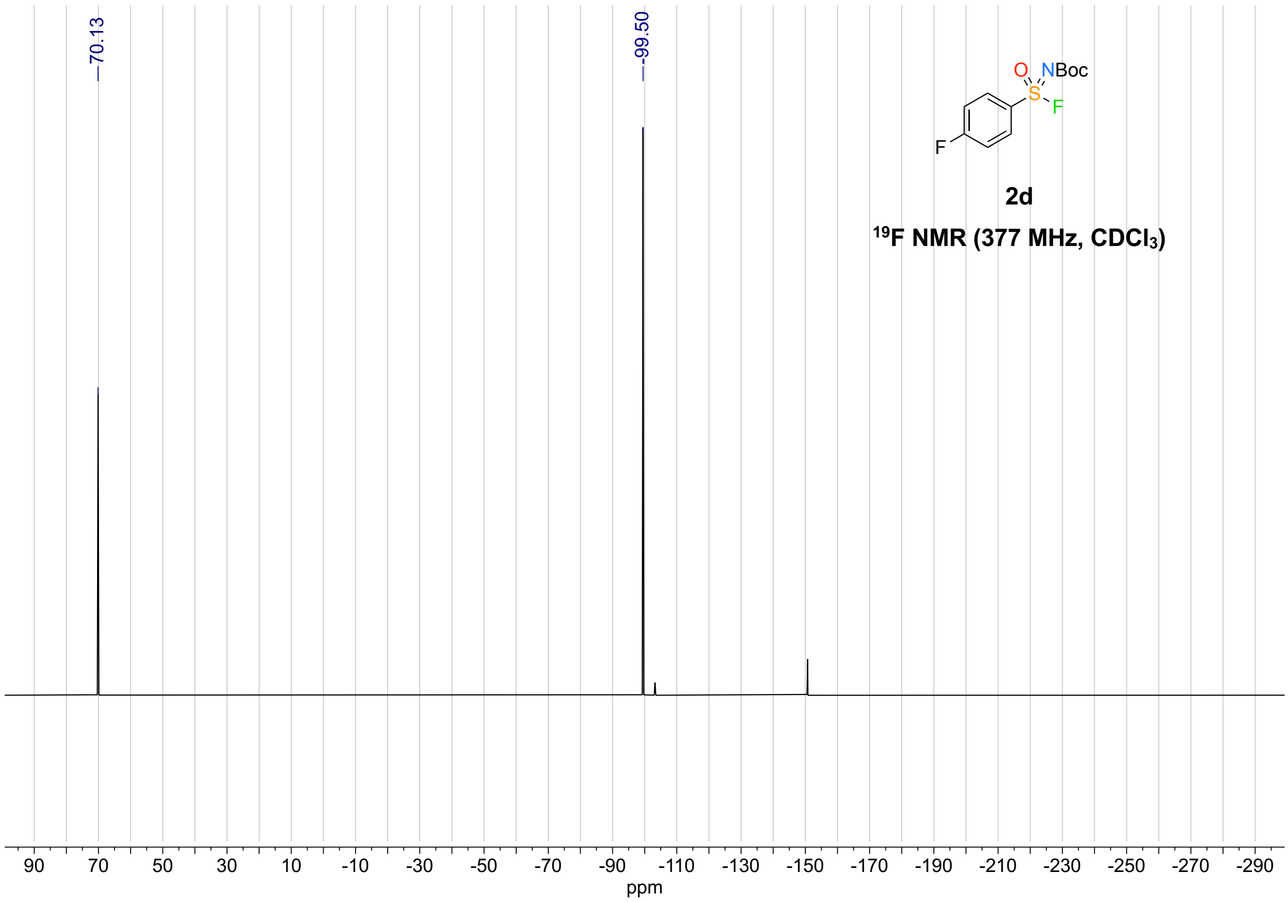

2d

^{19}F NMR (377 MHz, $CDCl_3$)

tert-Butyl (fluoro(4-methoxyphenyl)(oxo)-λ⁶-sulfaneylidene)carbamate (2e)

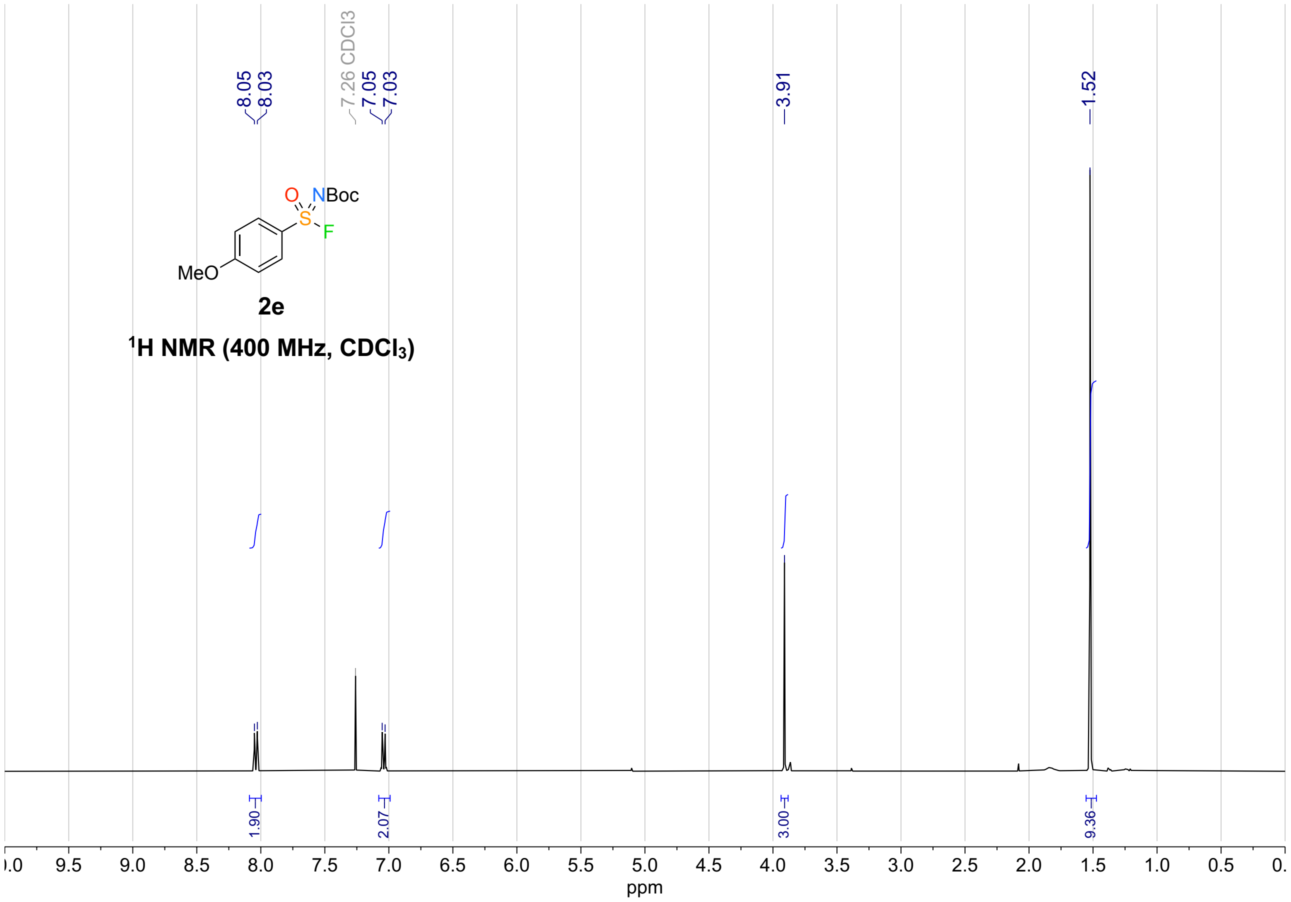

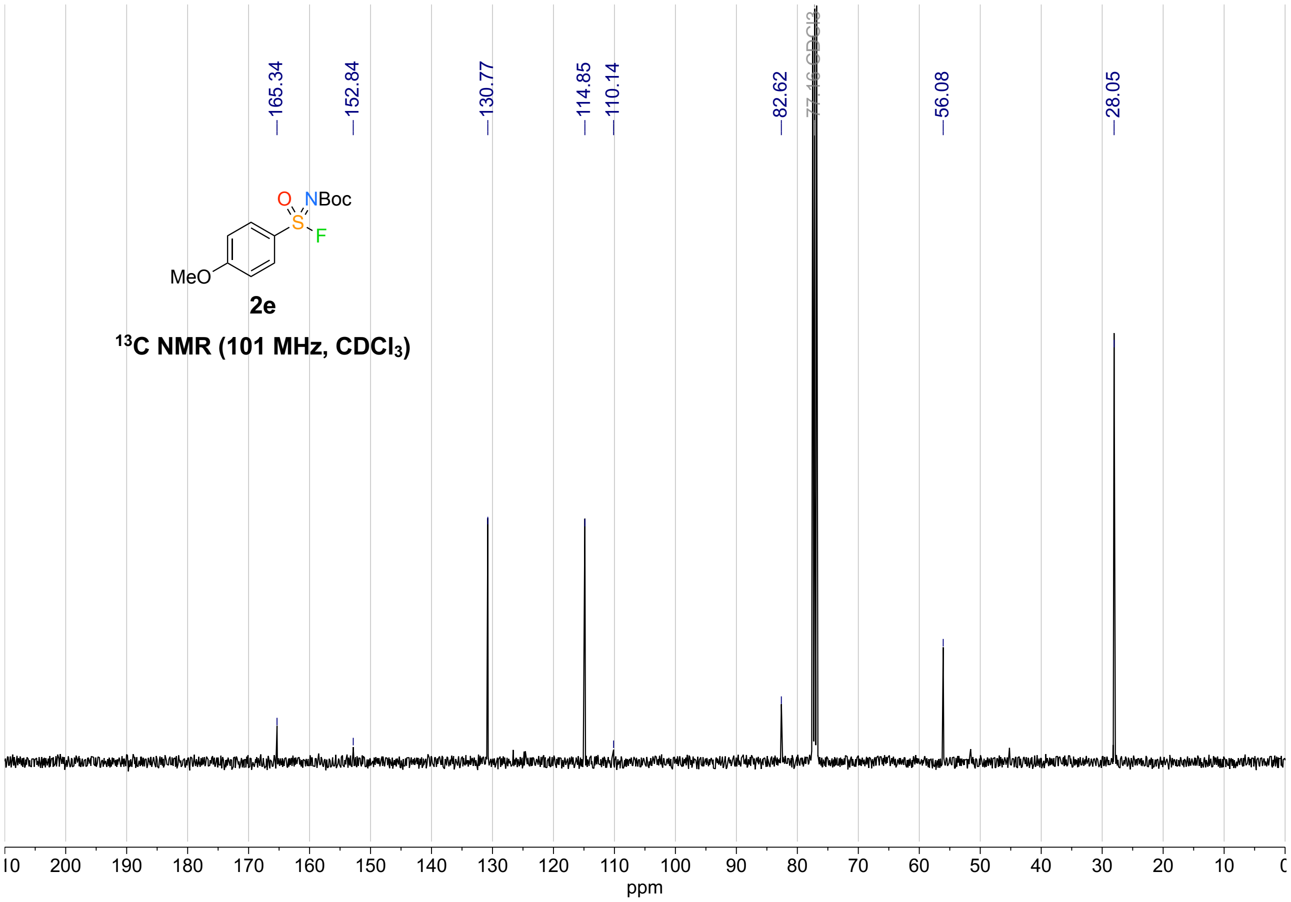

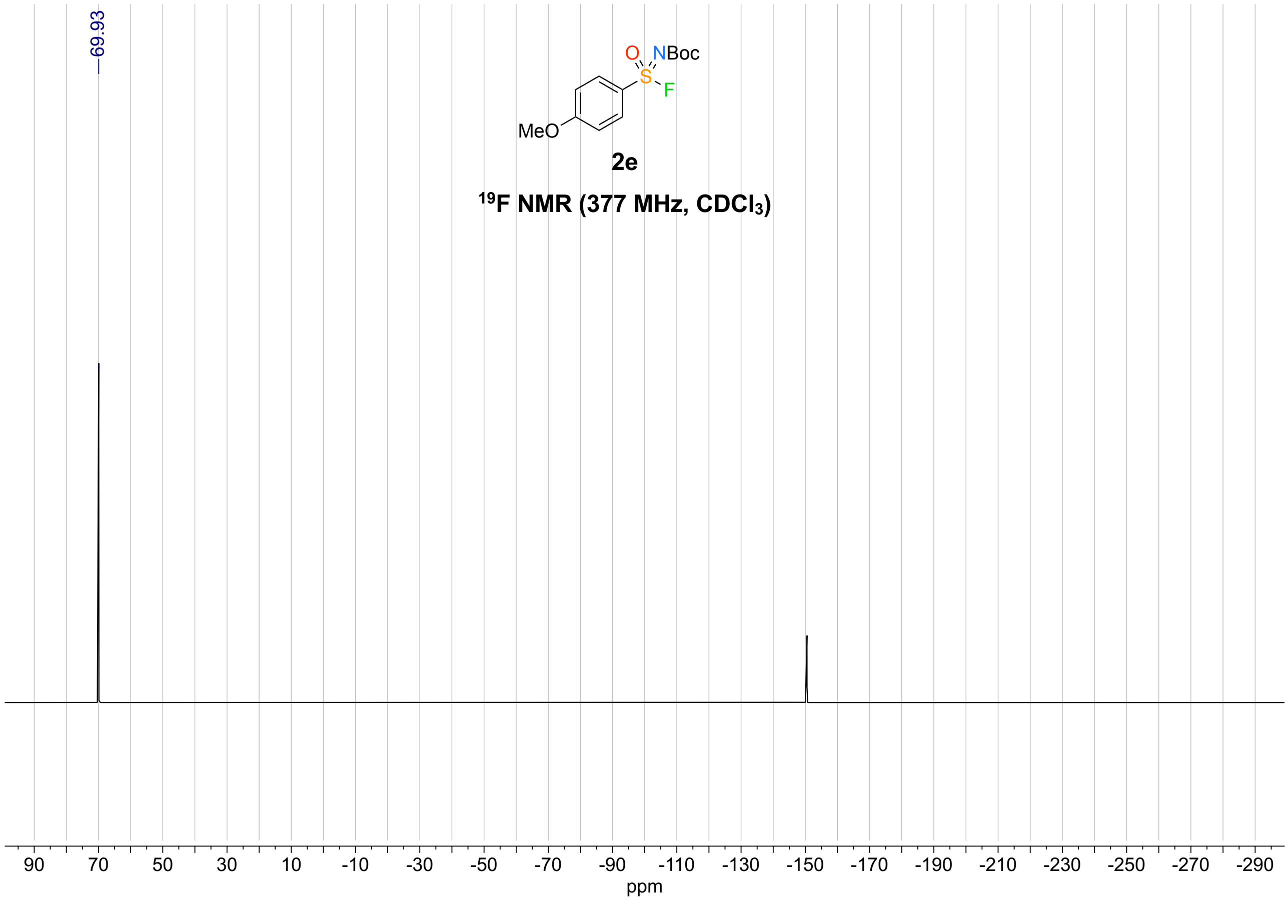
69.93
O
NBoc
S
F
MeO
2e
19F NMR (377 MHz, CDCl3)
90
70
50
30
10
-10
-30
-50
-70
-90
-110
-130
-150
-170
-190
-210
-230
-250
-270
-290
ppm

tert-Butyl (fluoro(oxo)(pyridin-2-yl)-λ⁶-sulfaneylidene)carbamate (2f)

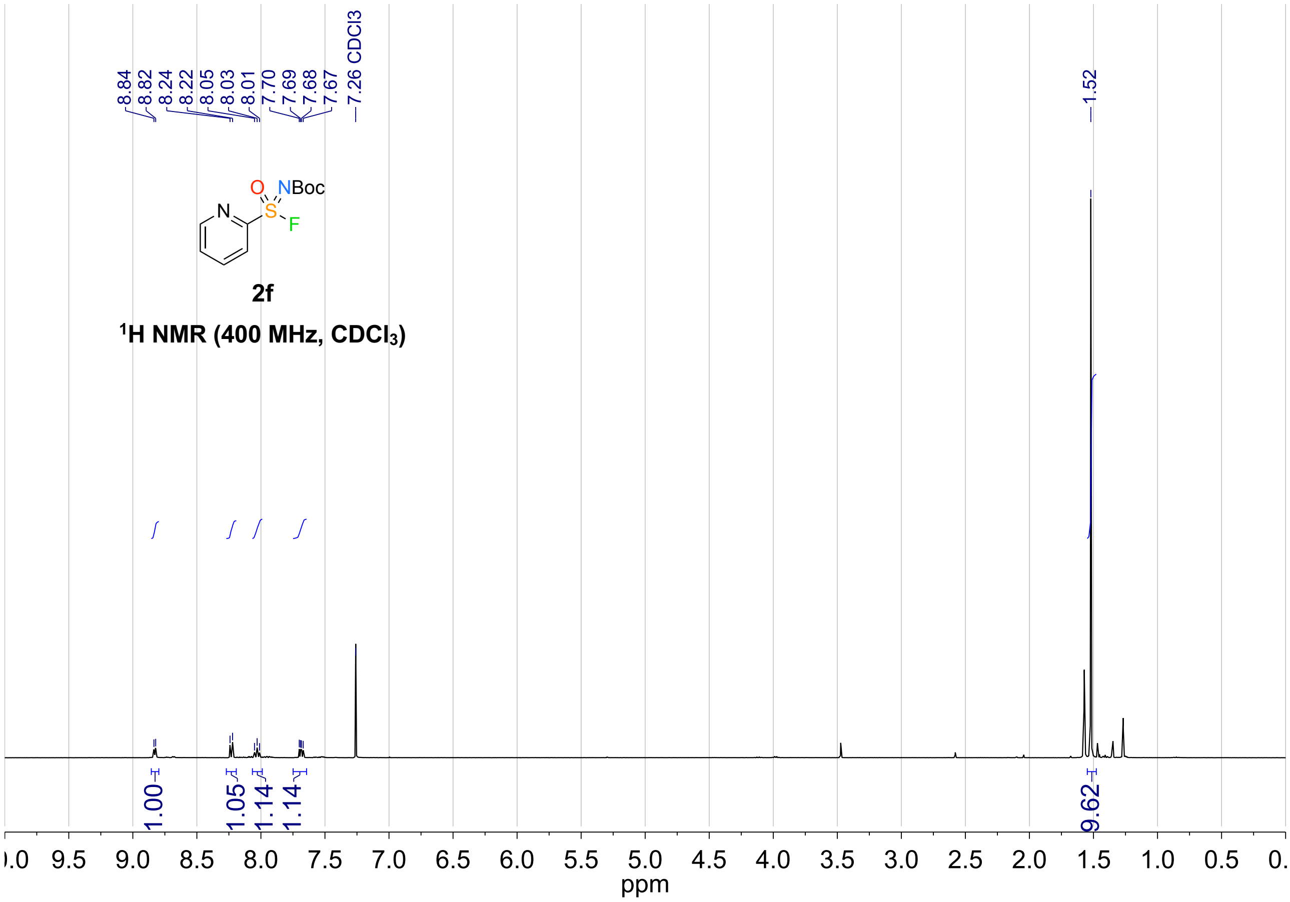

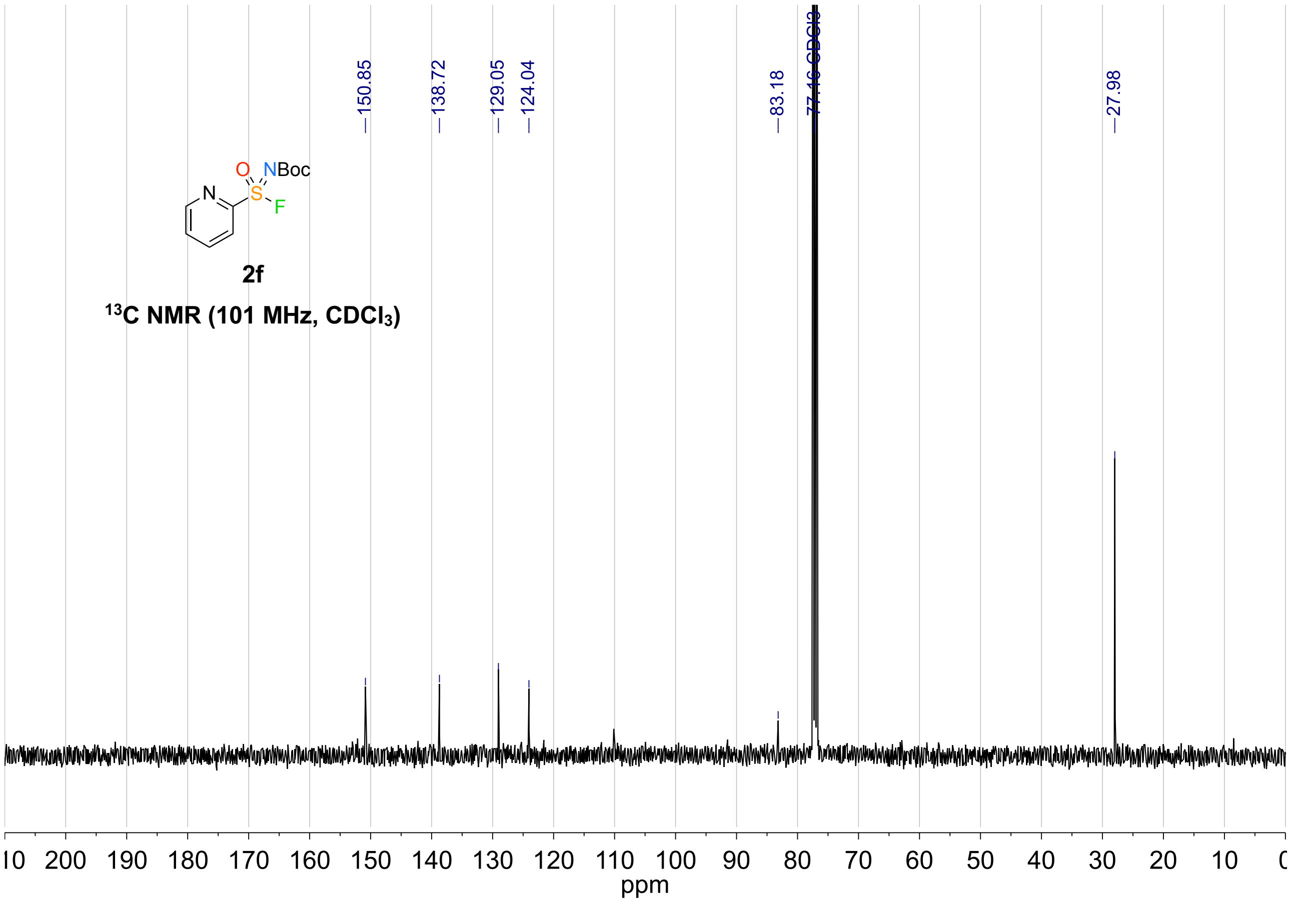

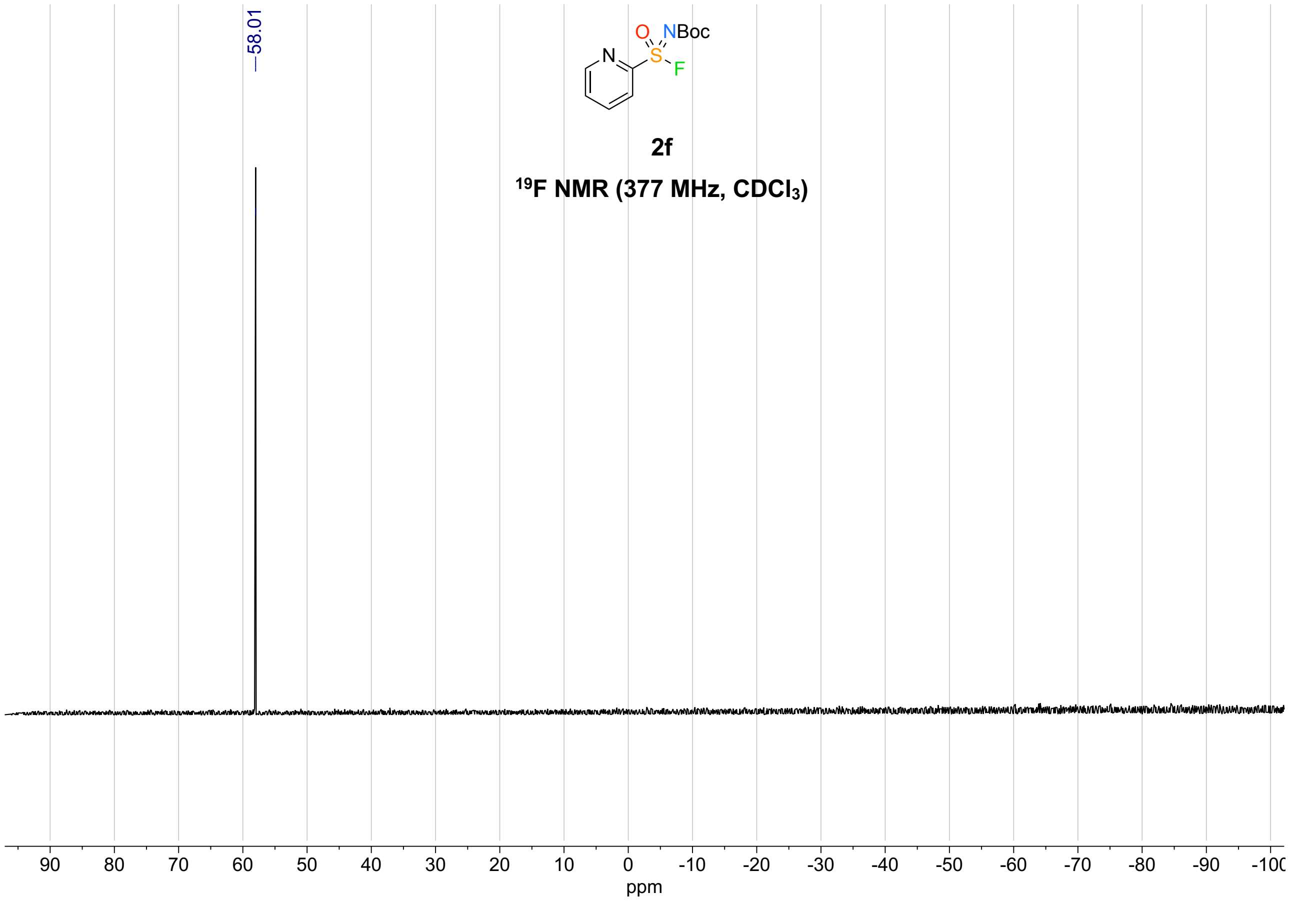

2f

^{19}F NMR (377 MHz, $CDCl_3$)

tert-Butyl (fluoro(isopropyl)(oxo)-λ^6-sulfaneylidene)carbamate (2g)

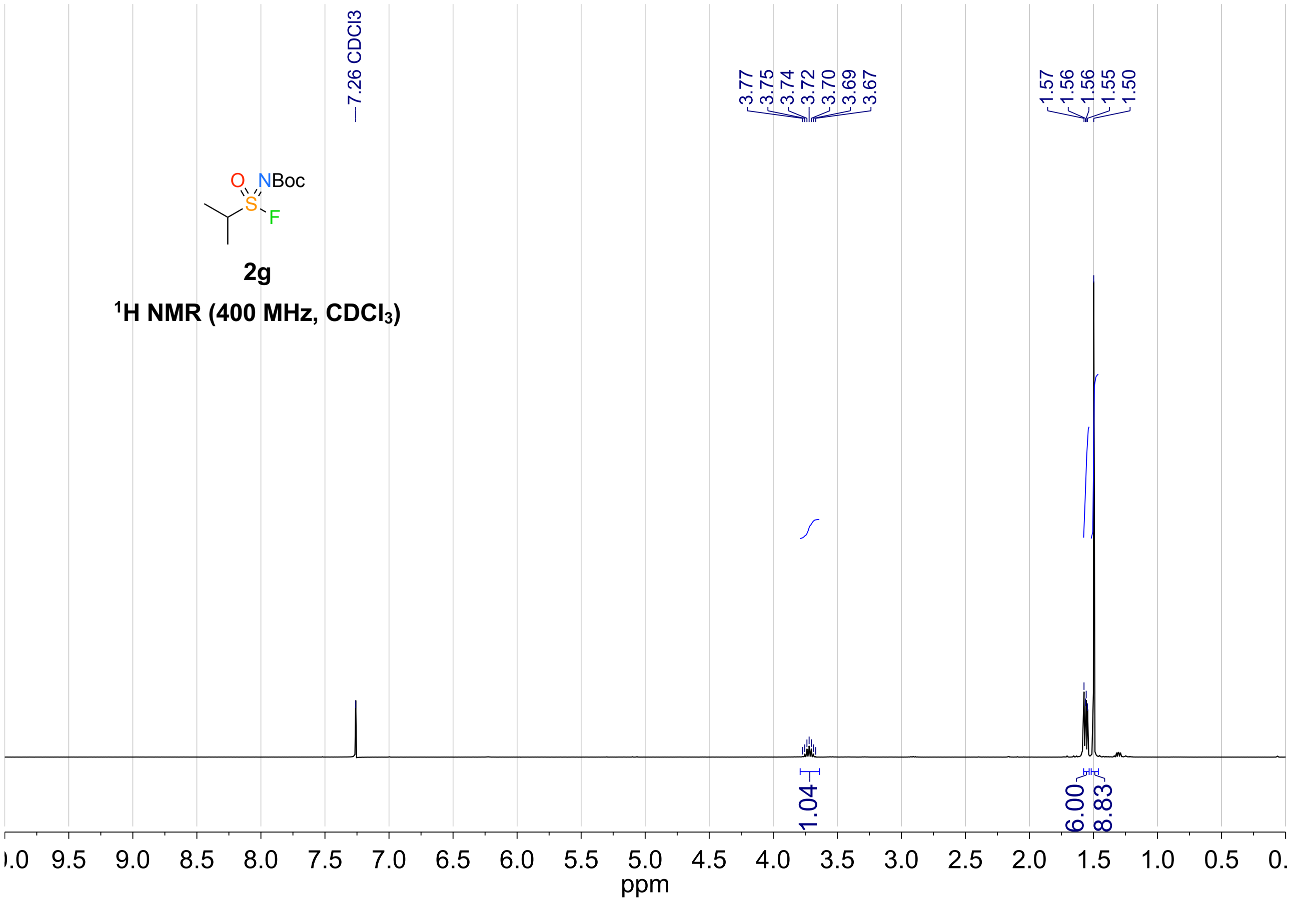

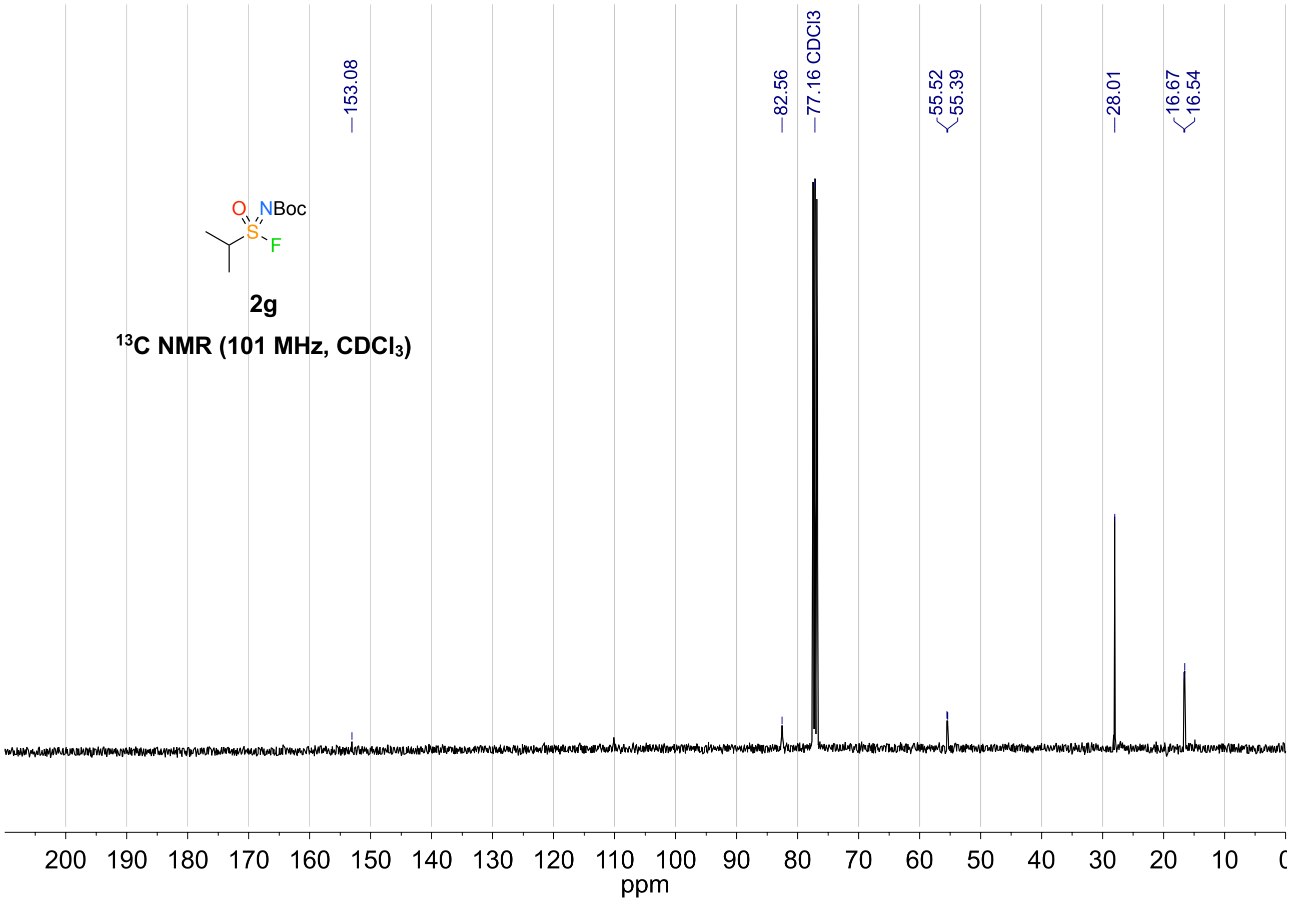

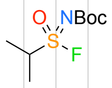

2g

^{19}F NMR (377 MHz, CDCl$_3$)

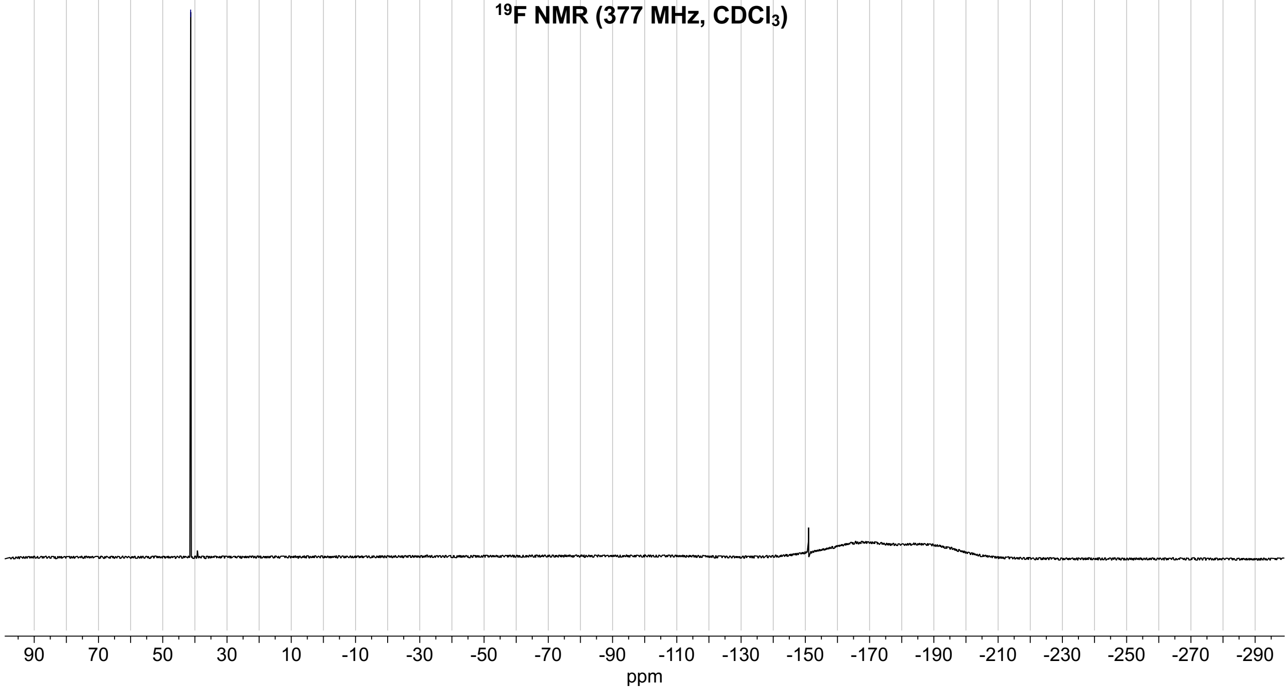

tert-Butyl (fluoro(methyl)(oxo)-λ^6-sulfaneylidene)carbamate (2h)

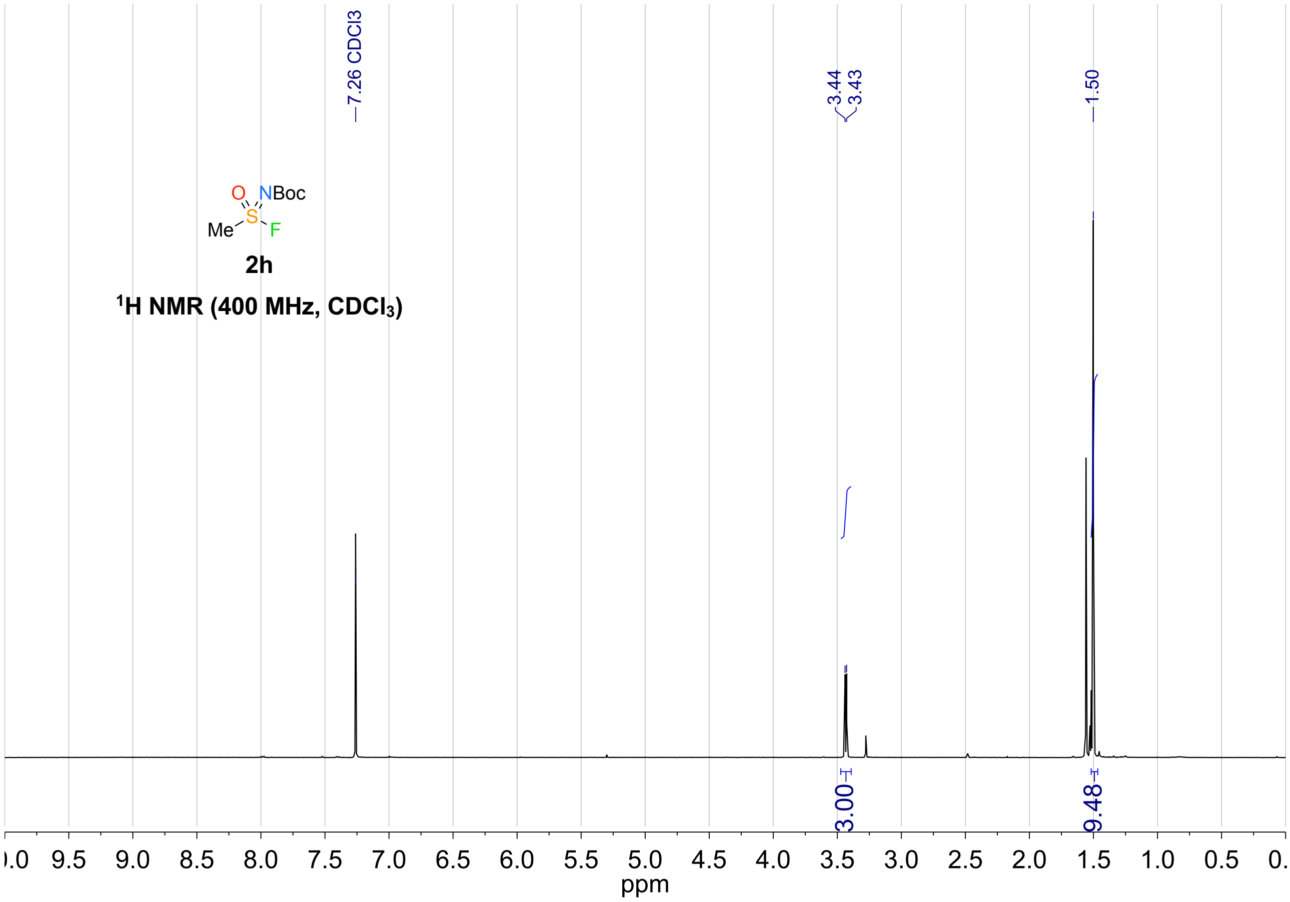

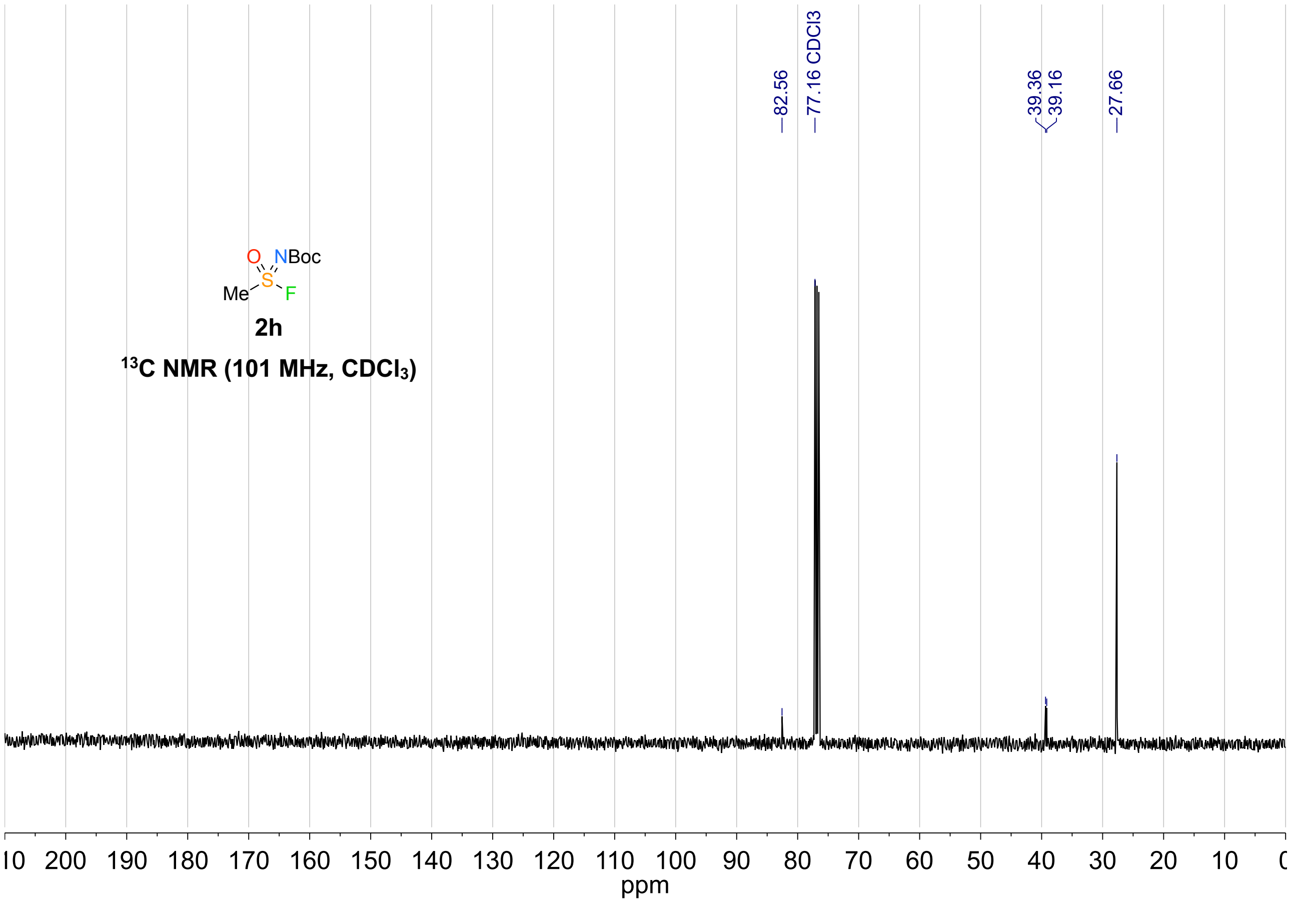

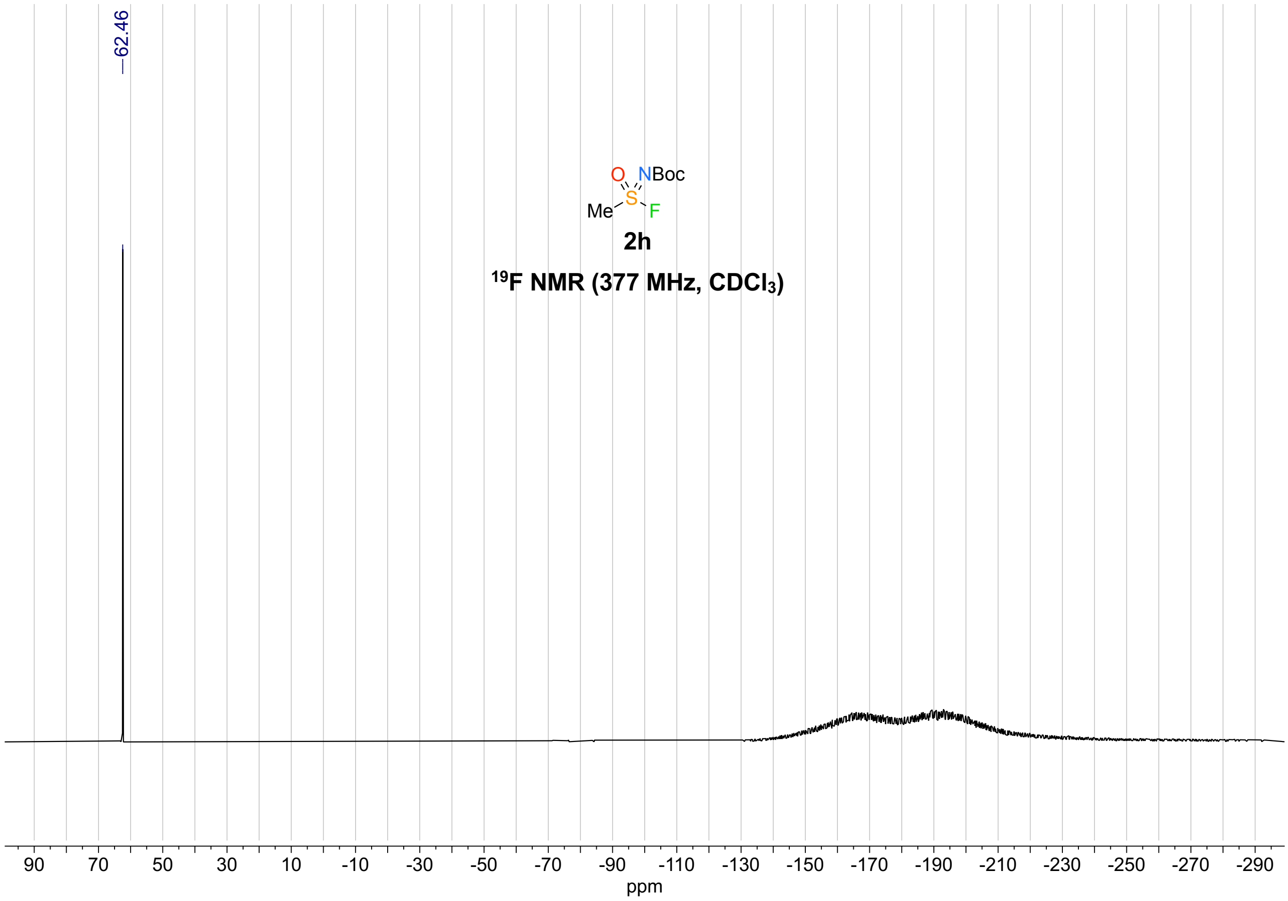

2h

^{19}F NMR (377 MHz, $CDCl_3$)

tert-Butyl (oxo(phenyl)(piperidin-1-yl)-λ⁶-sulfaneylidene)carbamate (3z)

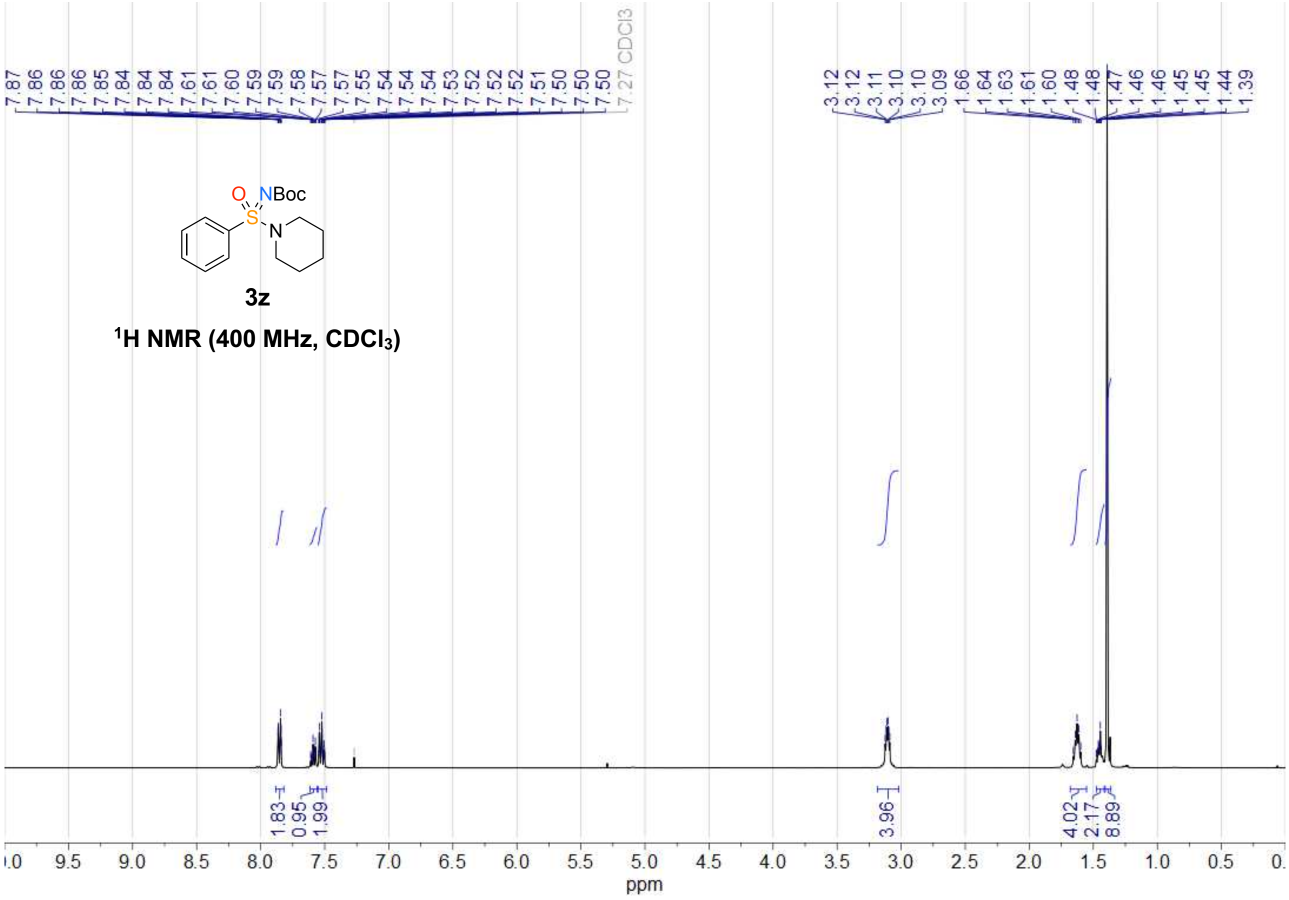

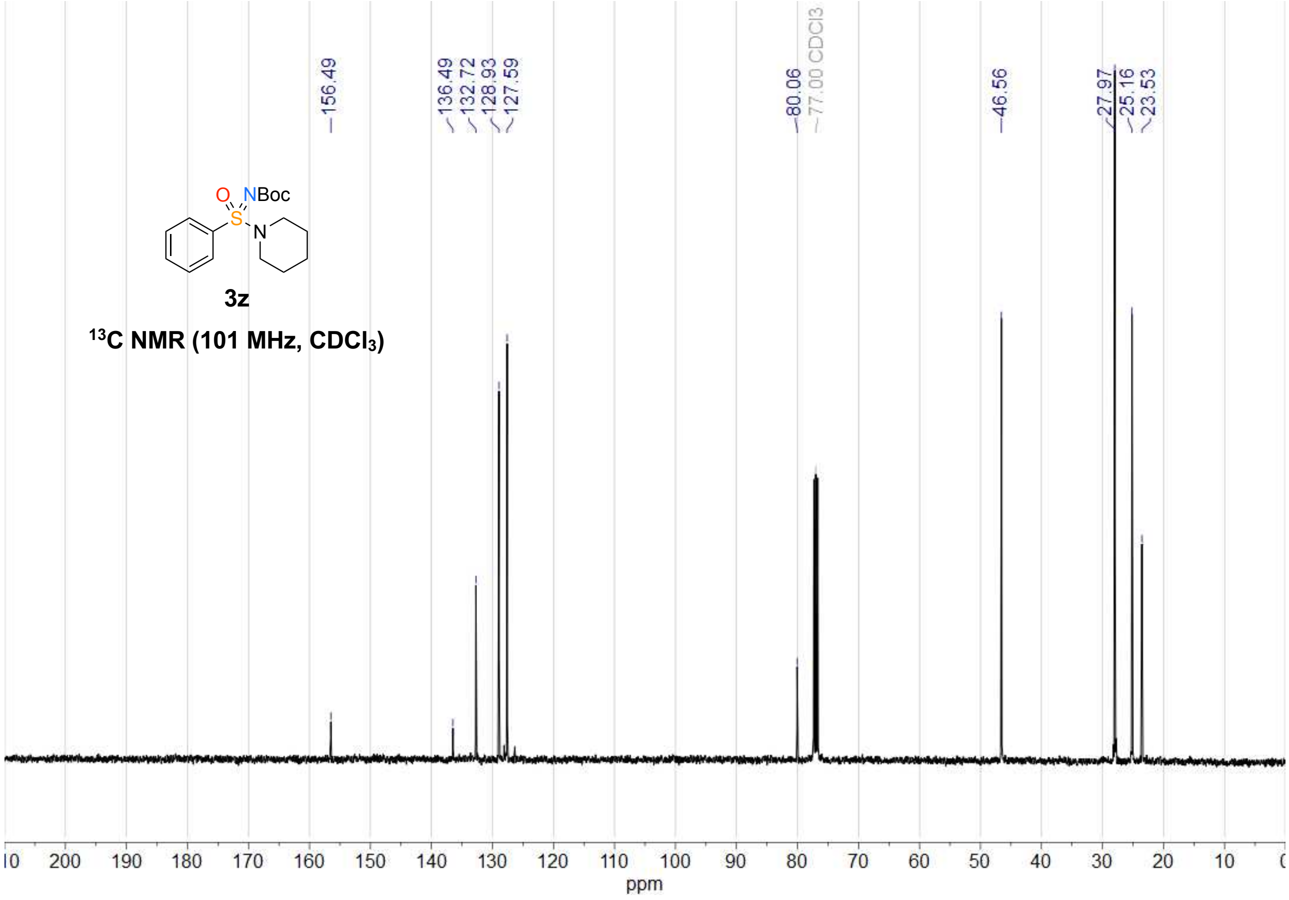

tert-Butyl ((4-fluorophenyl)(oxo)(piperidin-1-yl)-λ⁶-sulfaneylidene)carbamate (3aa)

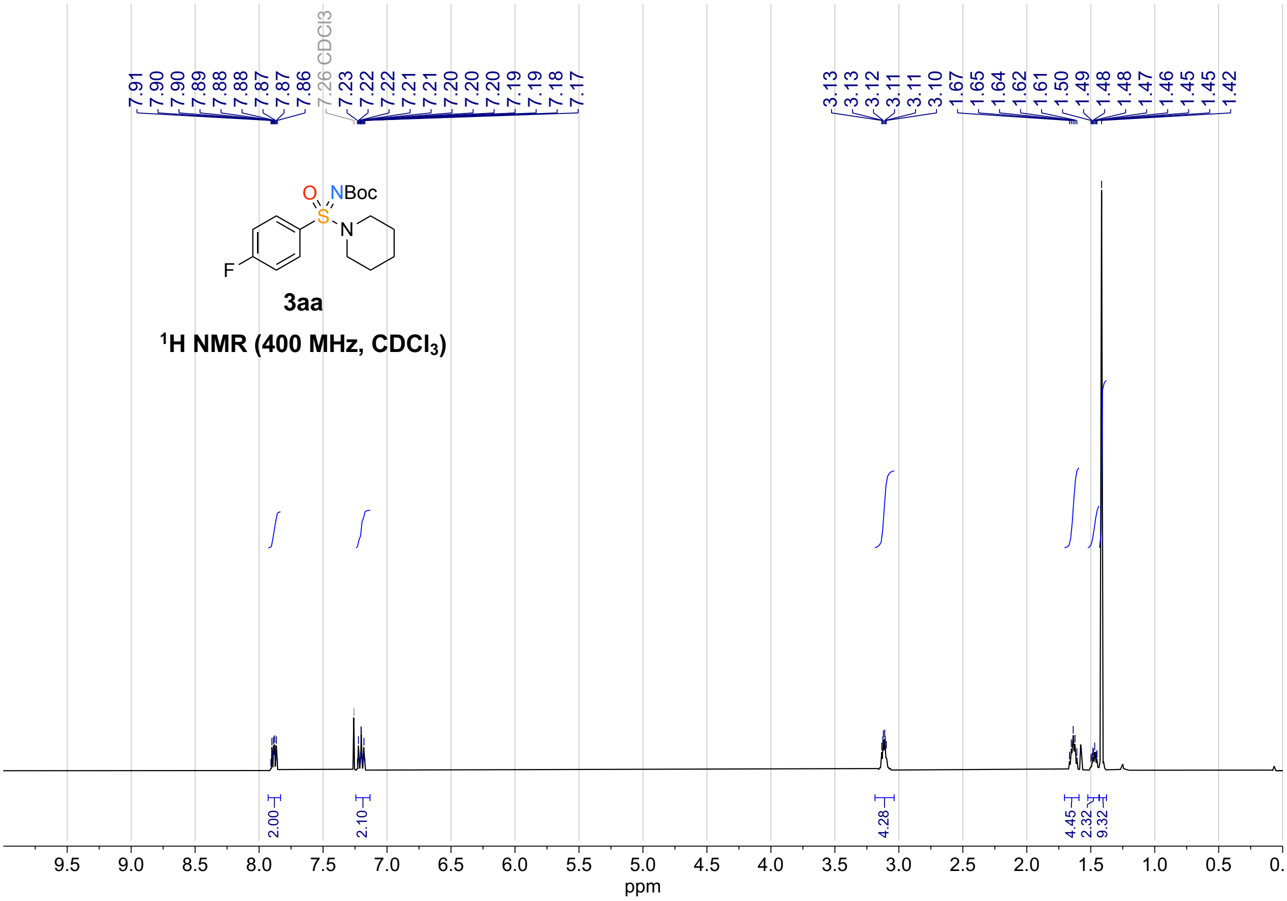

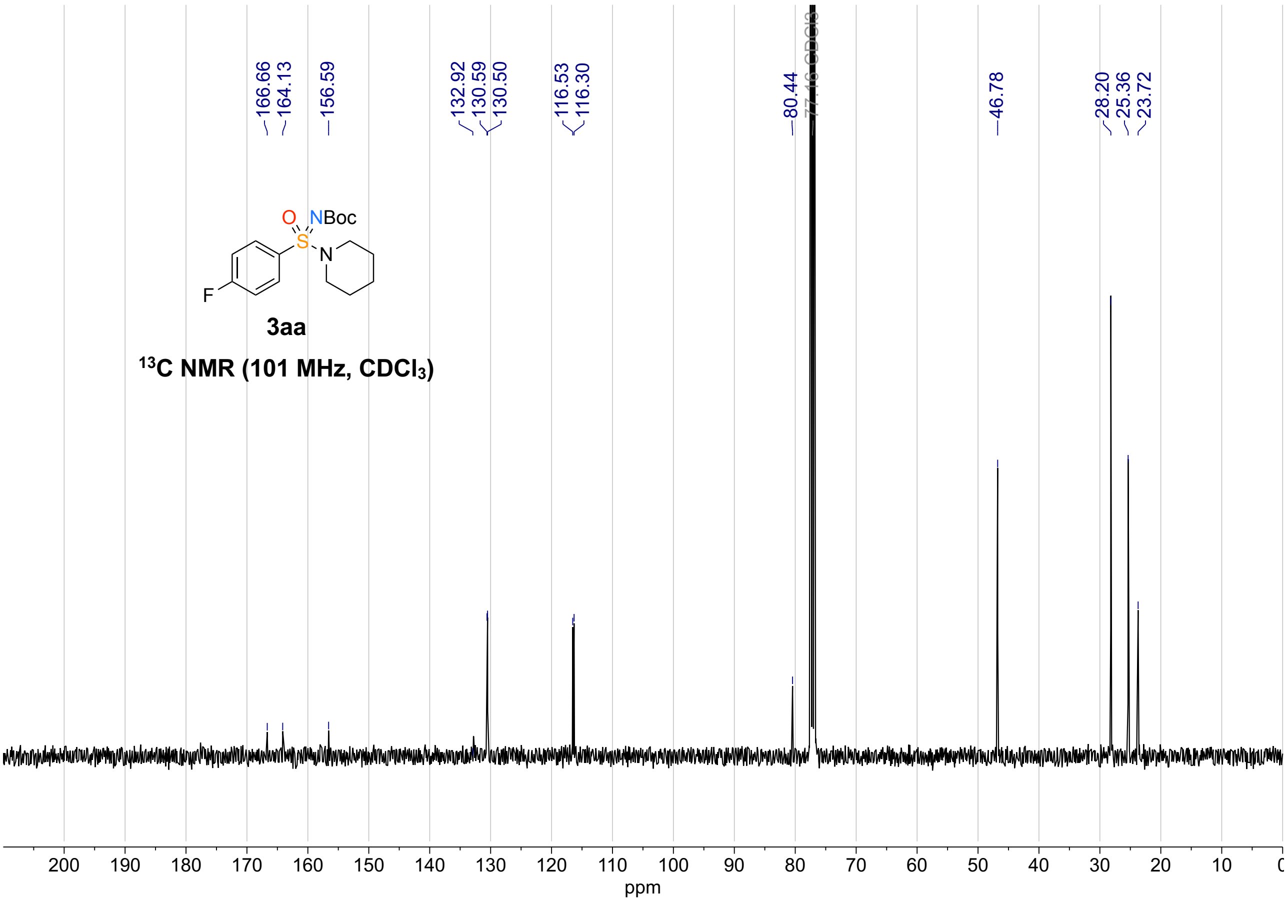

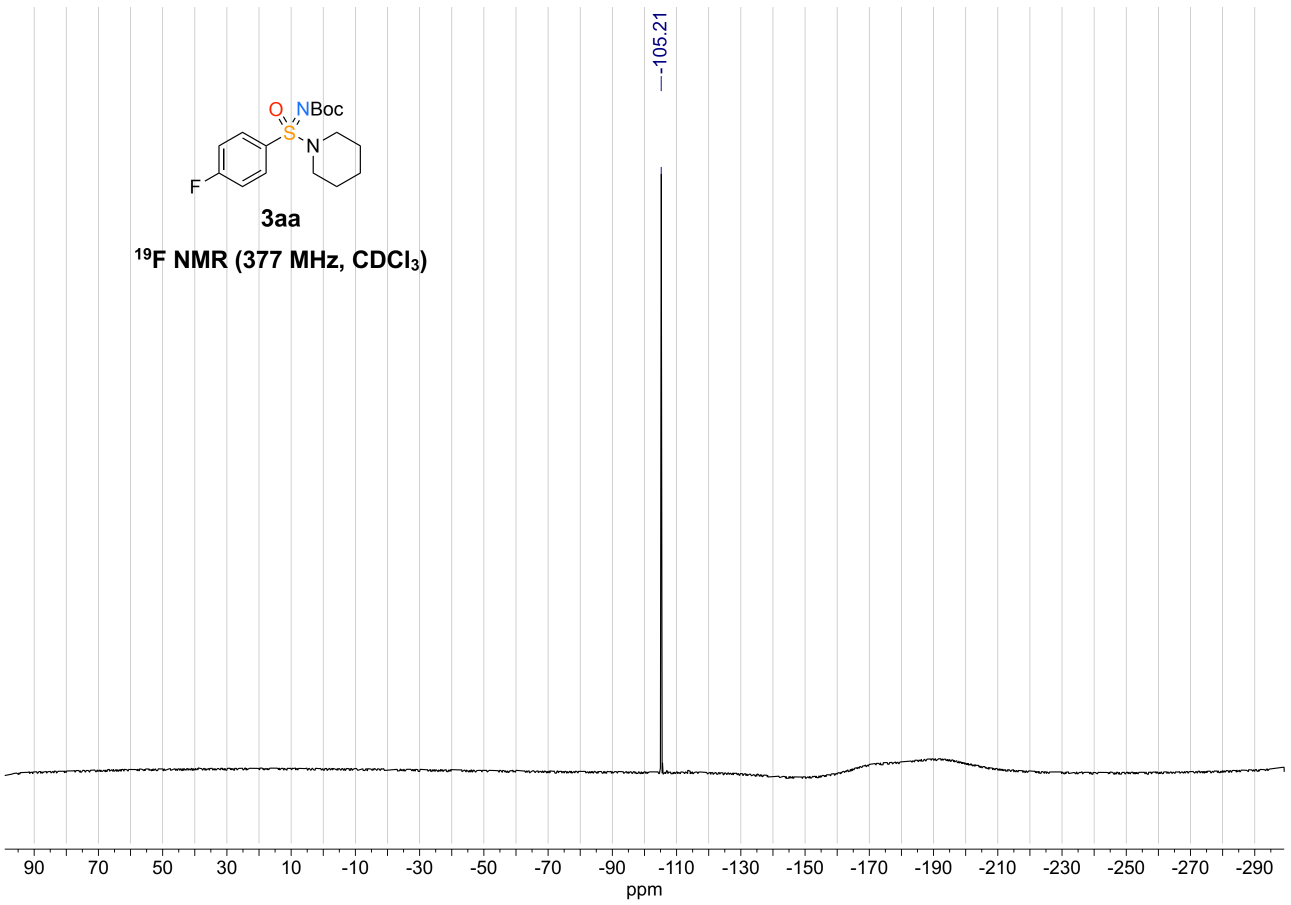

3aa

^{19}F NMR (377 MHz, $CDCl_3$)

tert-Butyl ((4-methoxyphenyl)(oxo)(piperidin-1-yl)-λ^6-sulfaneylidene)carbamate (3ab)

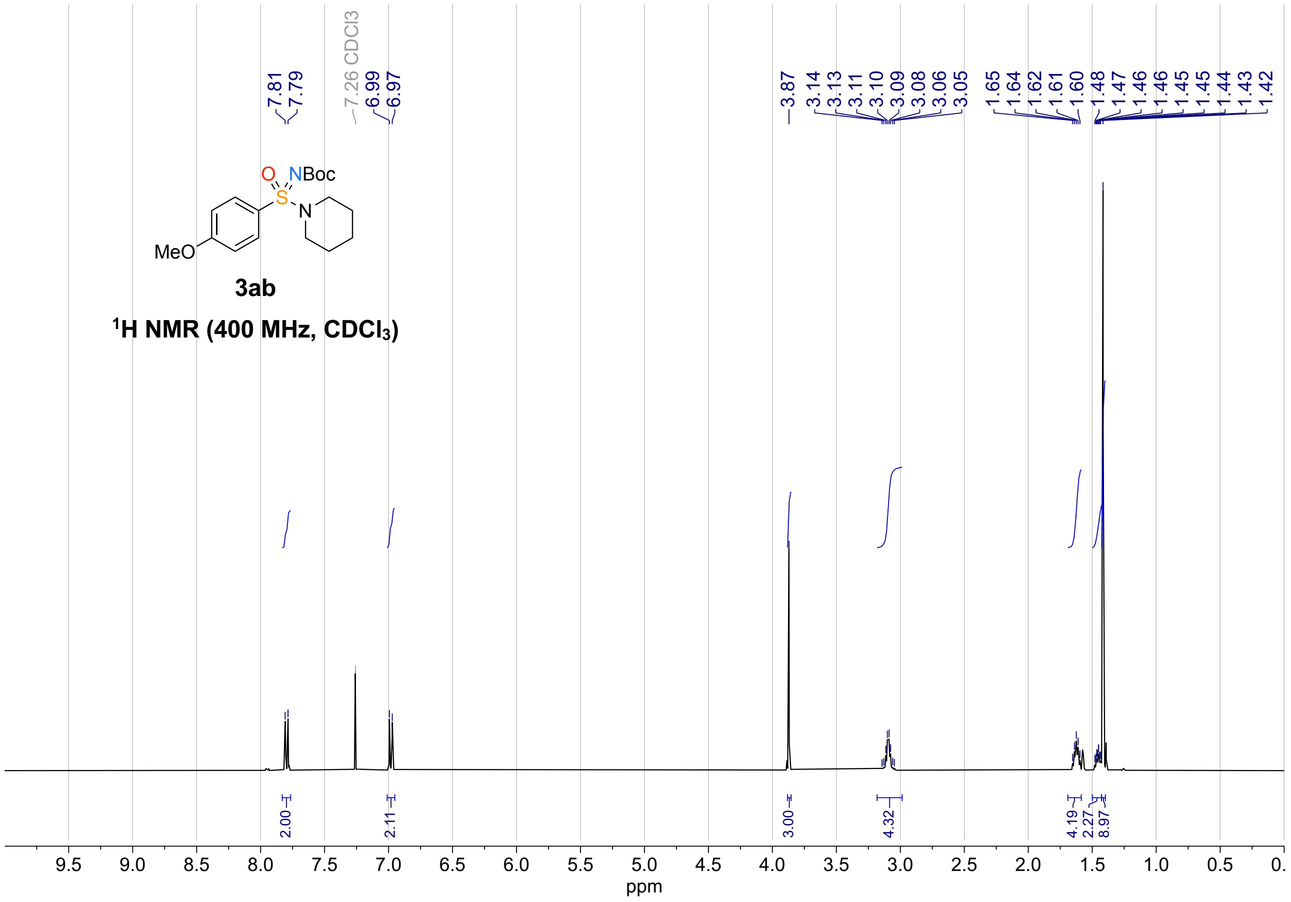

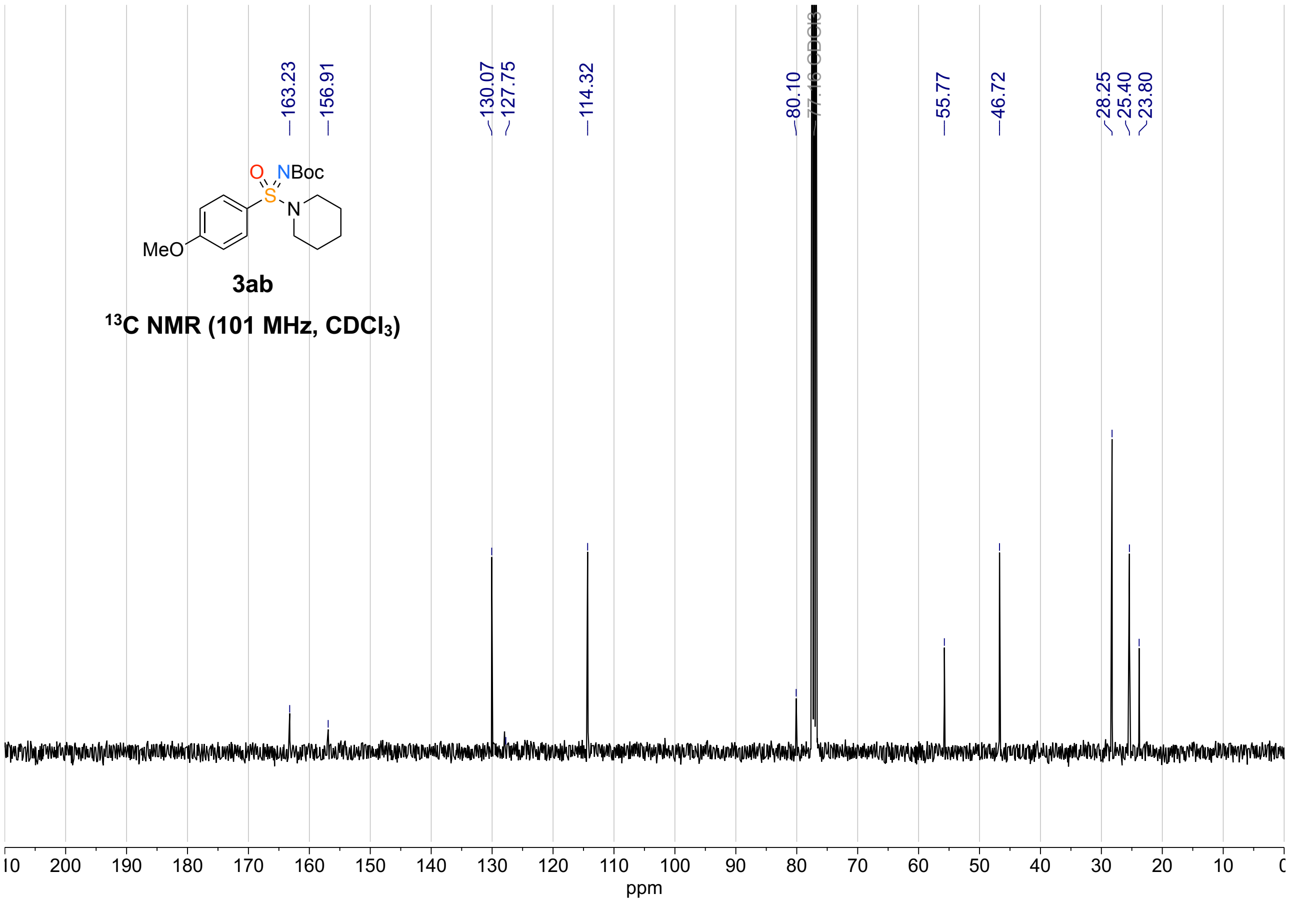

tert-Butyl (oxo(piperidin-1-yl)(pyridin-2-yl)-λ⁶-sulfaneylidene)carbamate (3ac)

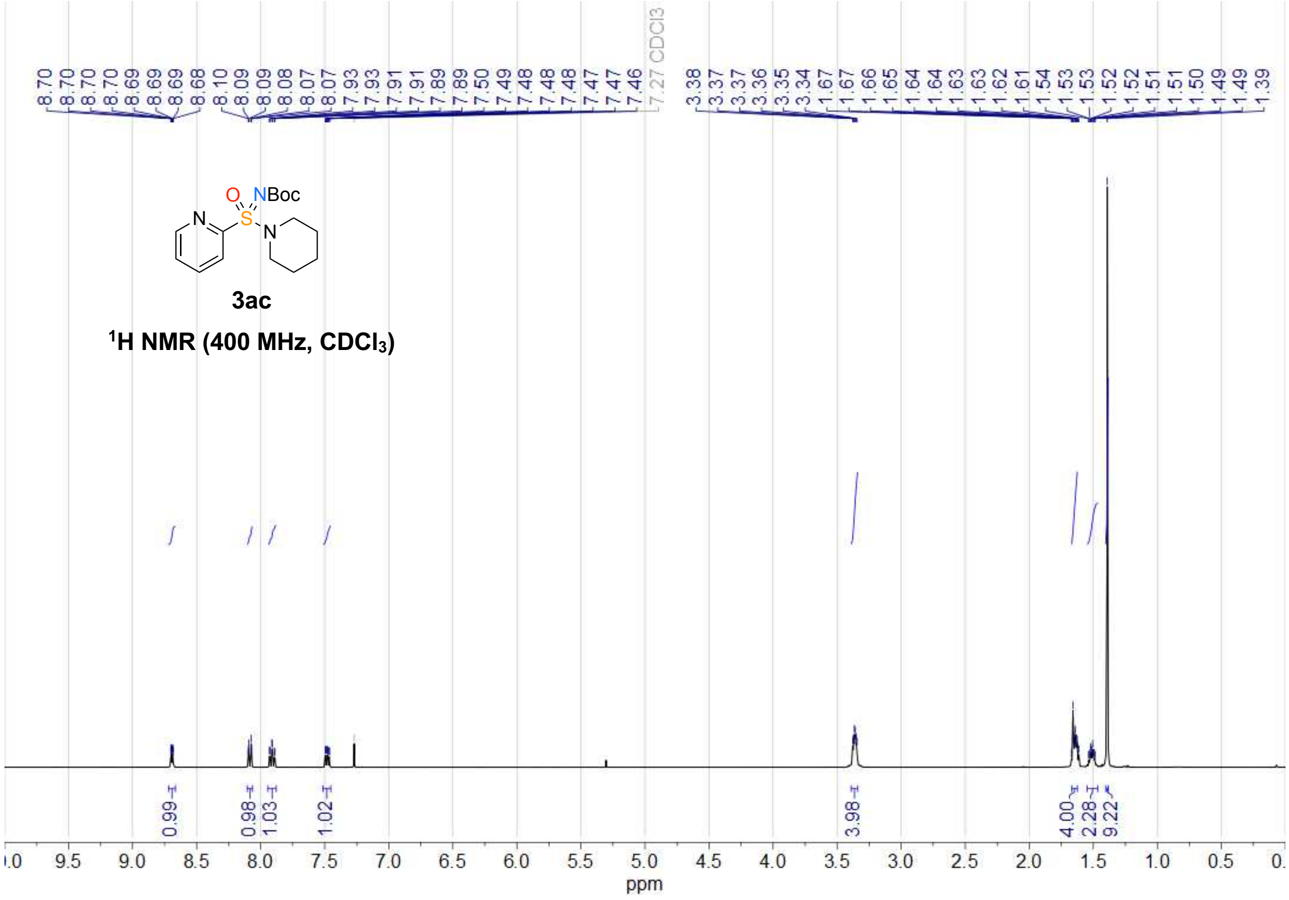

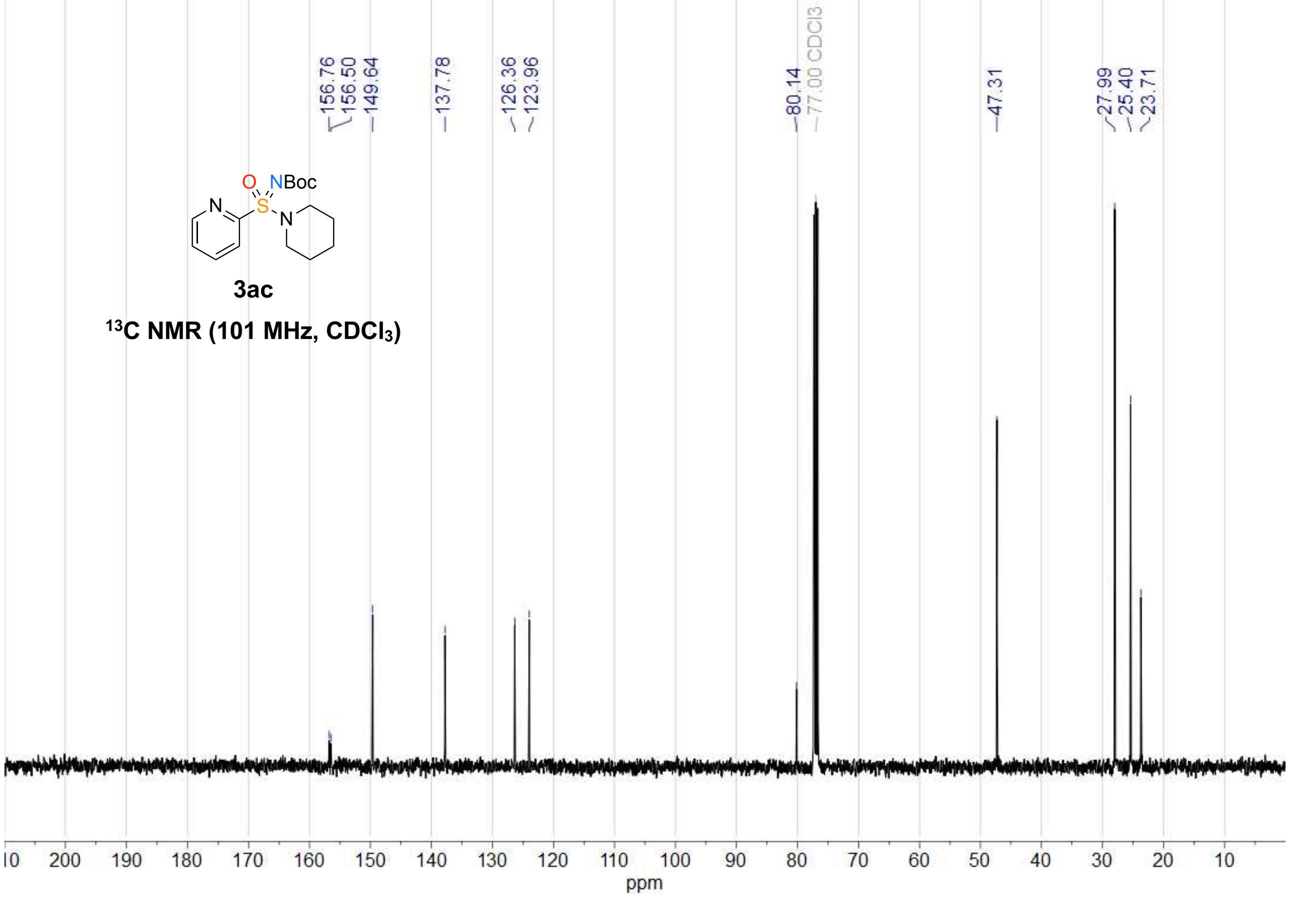

tert-Butyl (isopropyl(oxo)(piperidin-1-yl)-λ^6-sulfaneylidene)carbamate (3ad)

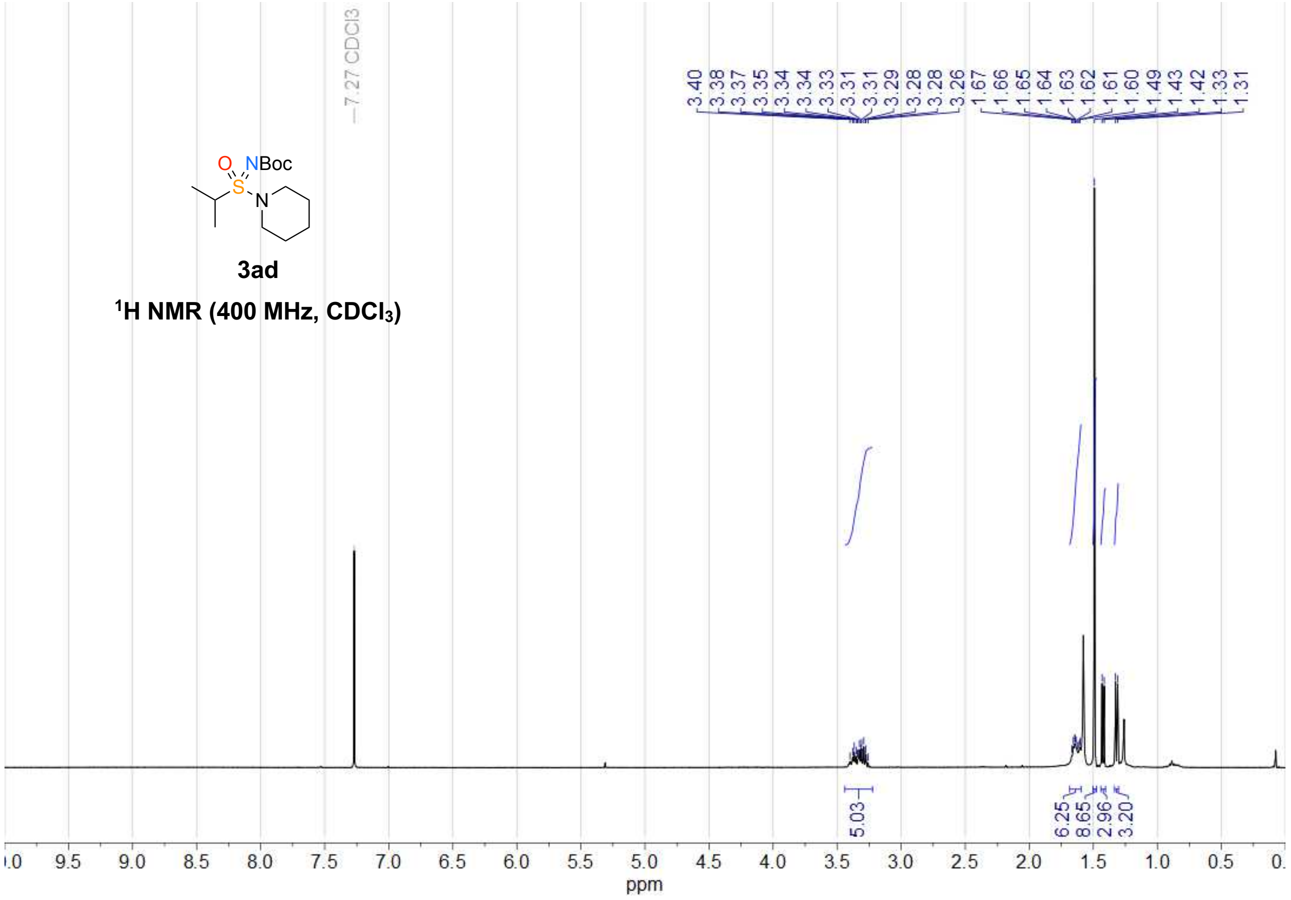

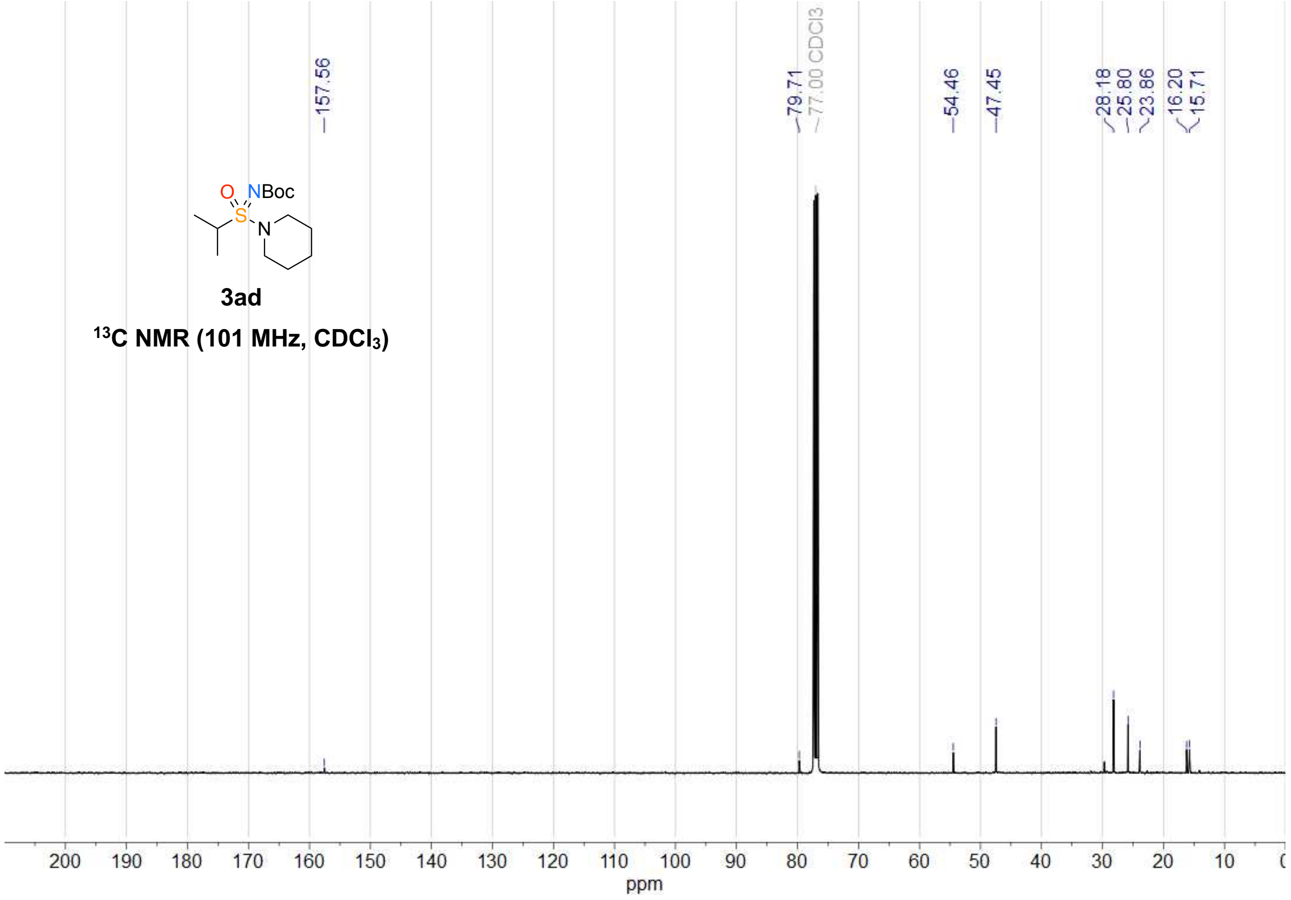

tert-Butyl (methyl(oxo)(piperidin-1-yl)-λ^6-sulfaneylidene)carbamate (3ae)

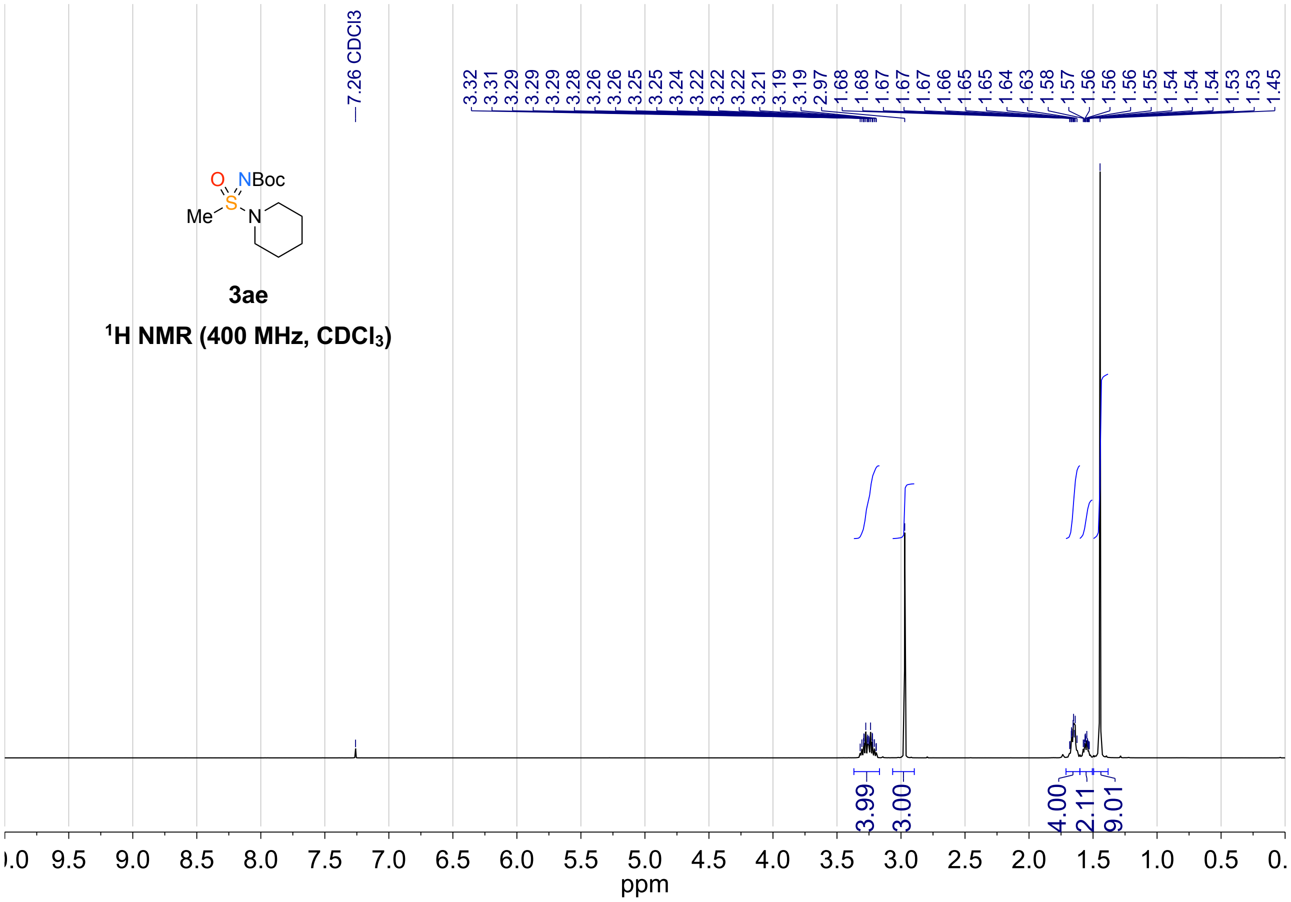

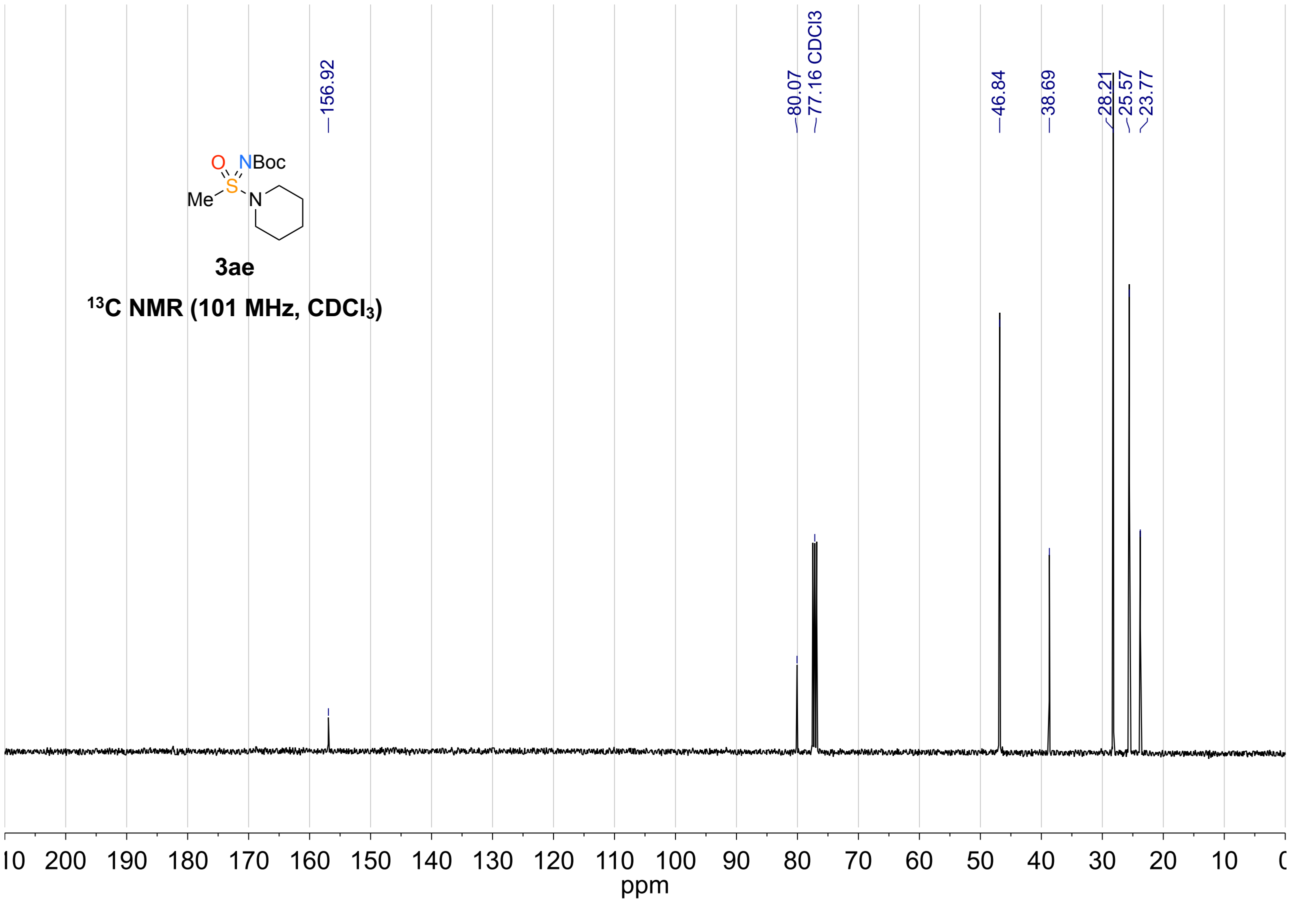

tert-Butyl (methyl(morpholino)(oxo)-λ^6-sulfaneylidene)carbamate (3af)

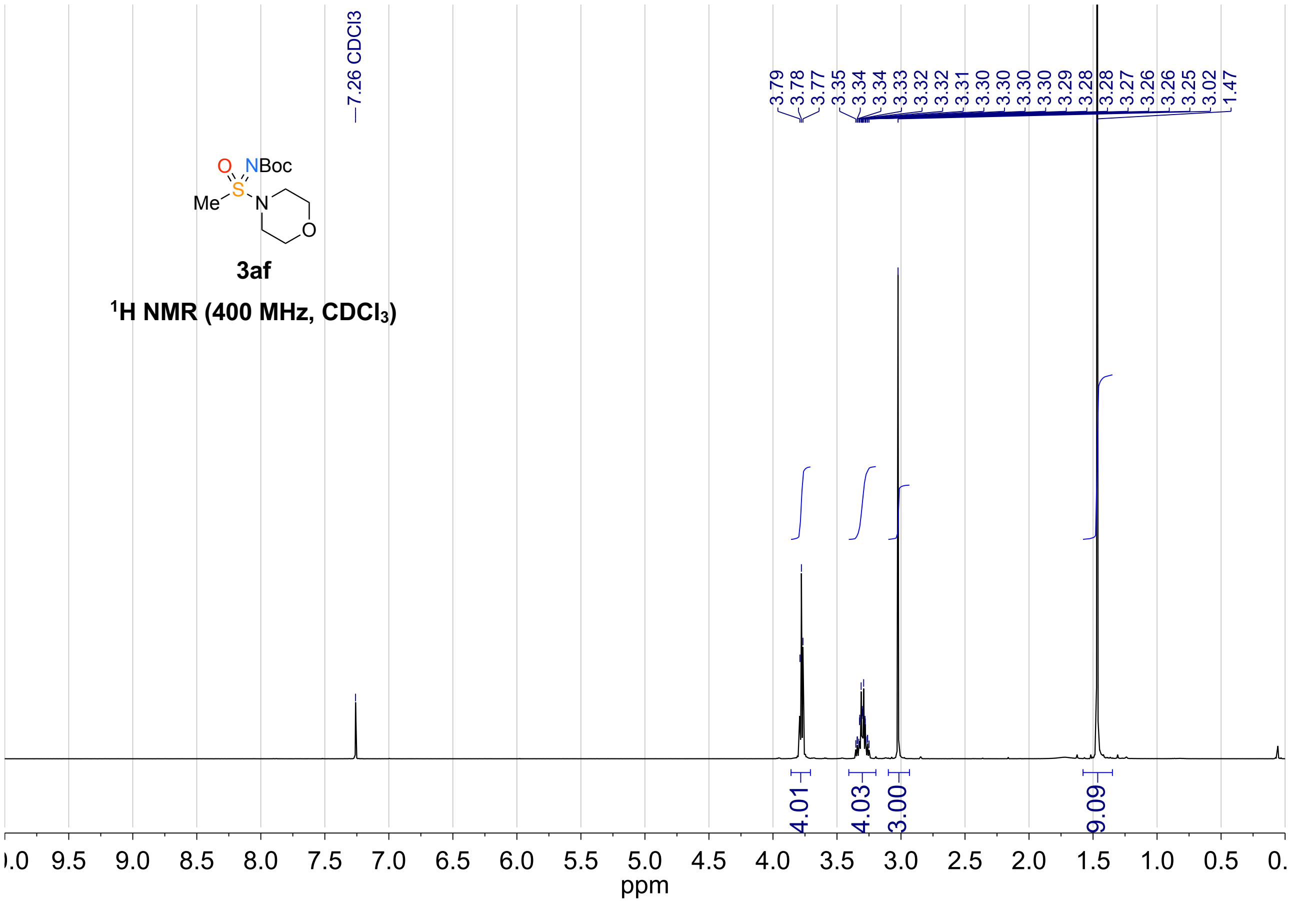

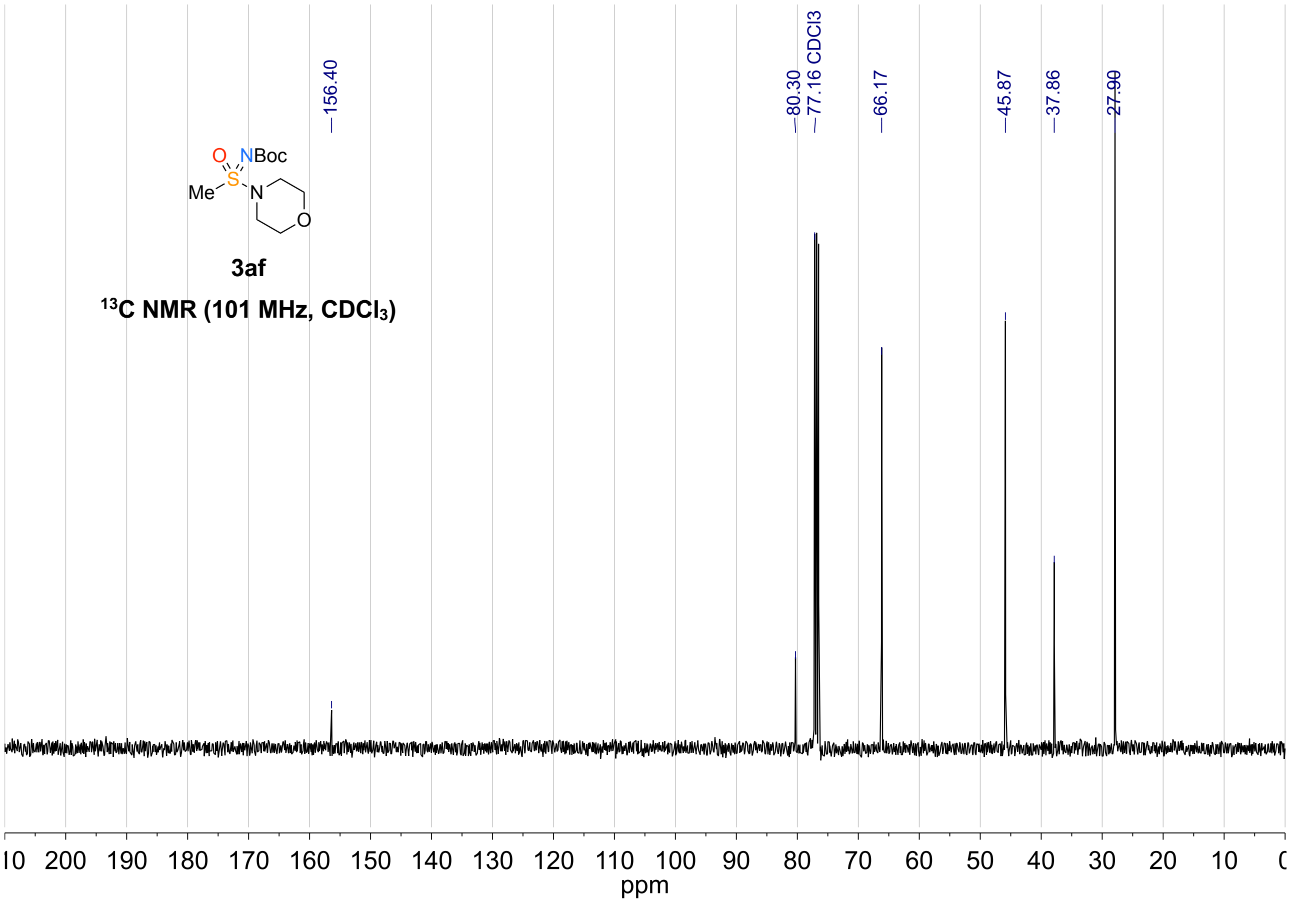

tert-Butyl 4-(N-(tert-butoxycarbonyl)-S-methylsulfonimidoyl)piperazine-1-carboxylate (3ag)

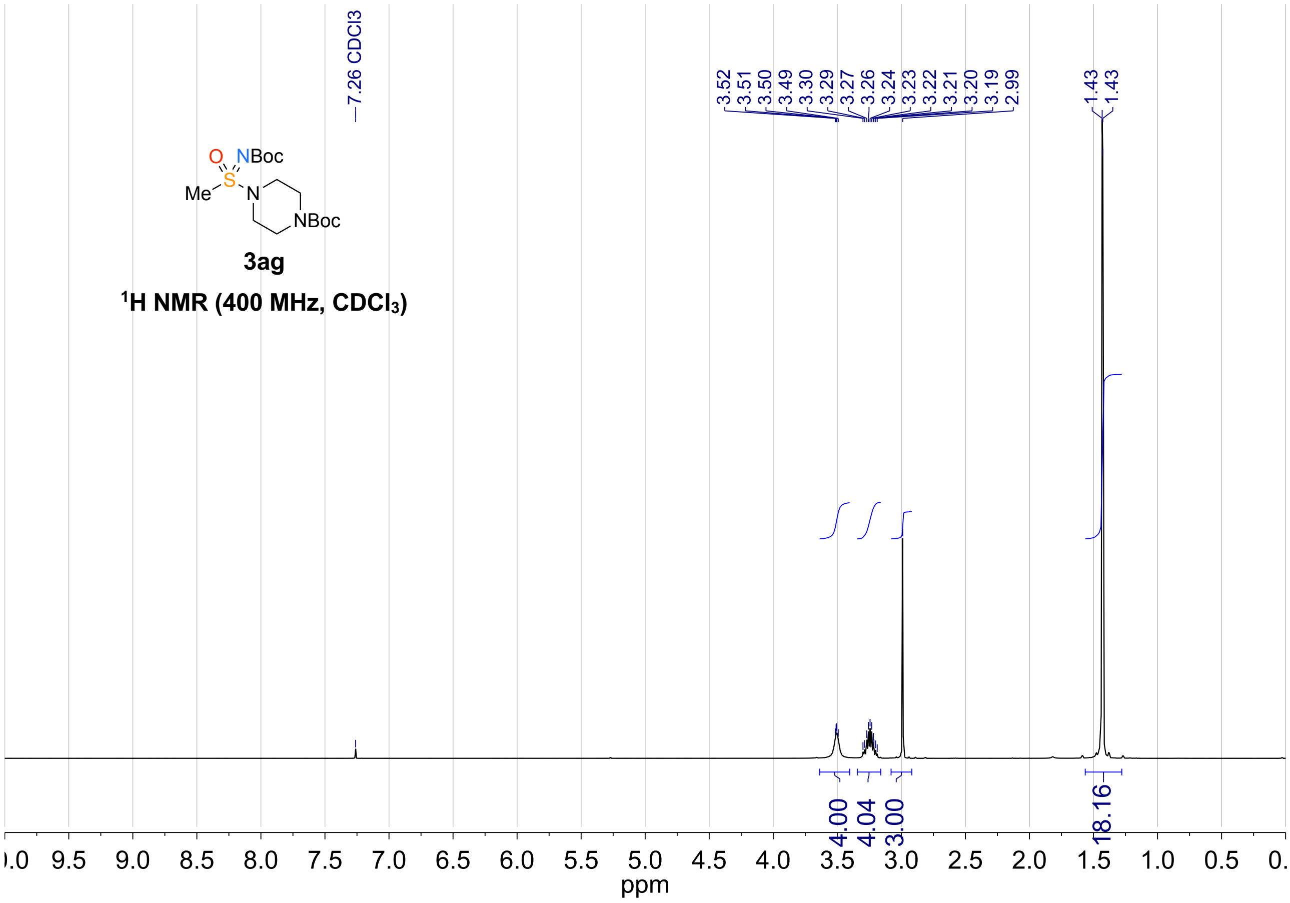

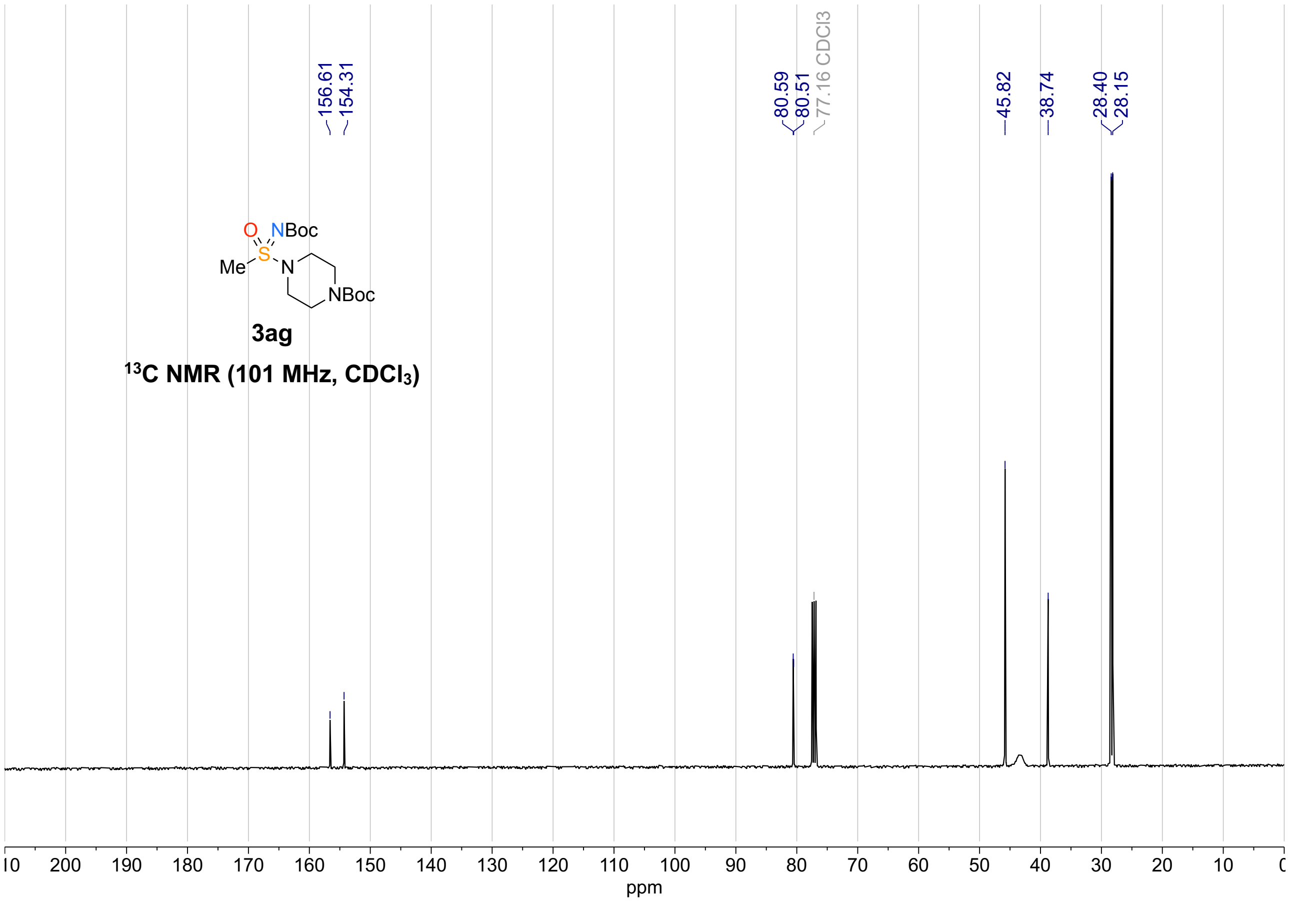

tert-Butyl (((4-((6-methoxyquinolin-8-yl)amino)pentyl)amino)(methyl)(oxo)-λ^6-sulfaneylidene)carbamate (3ah)

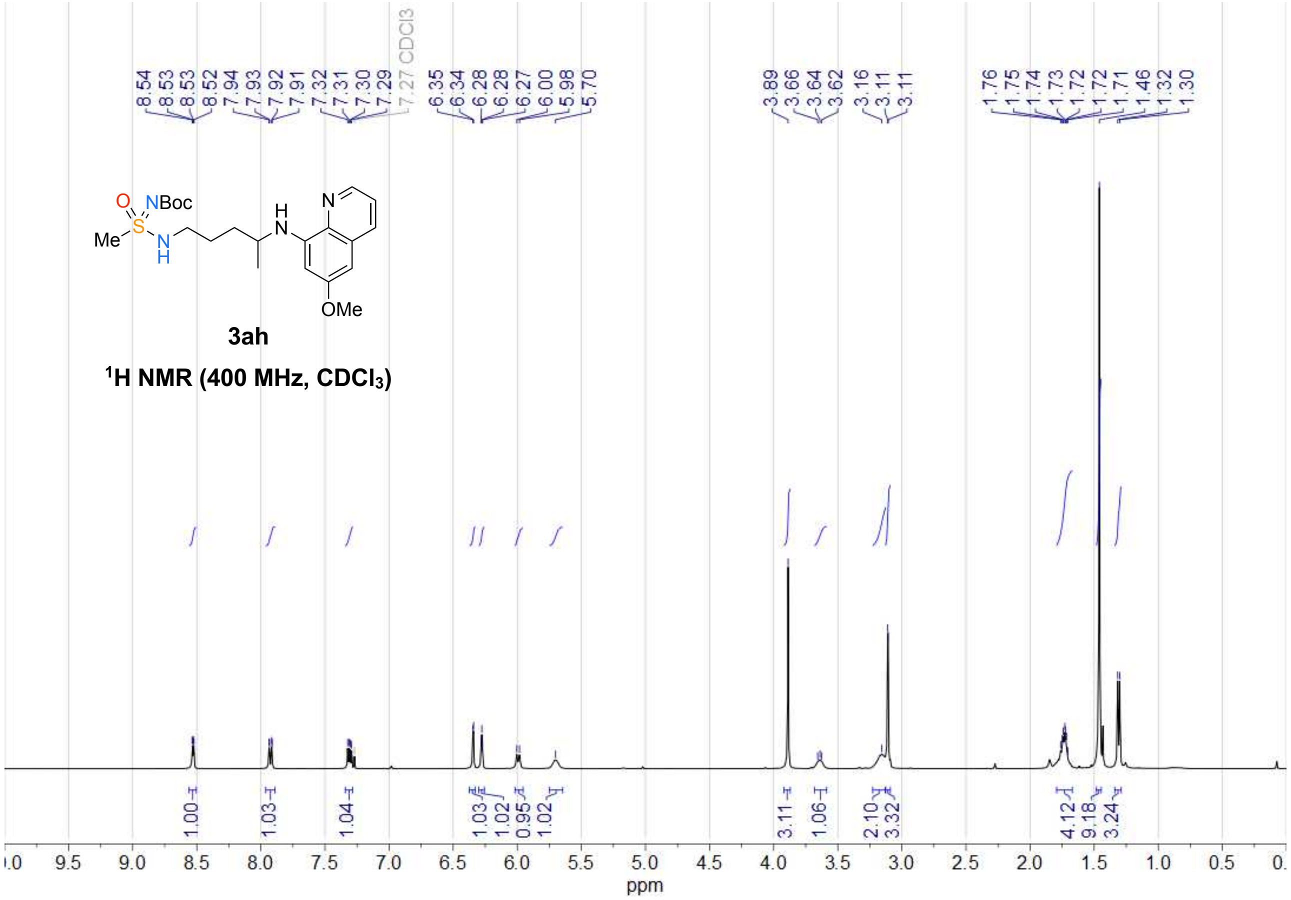

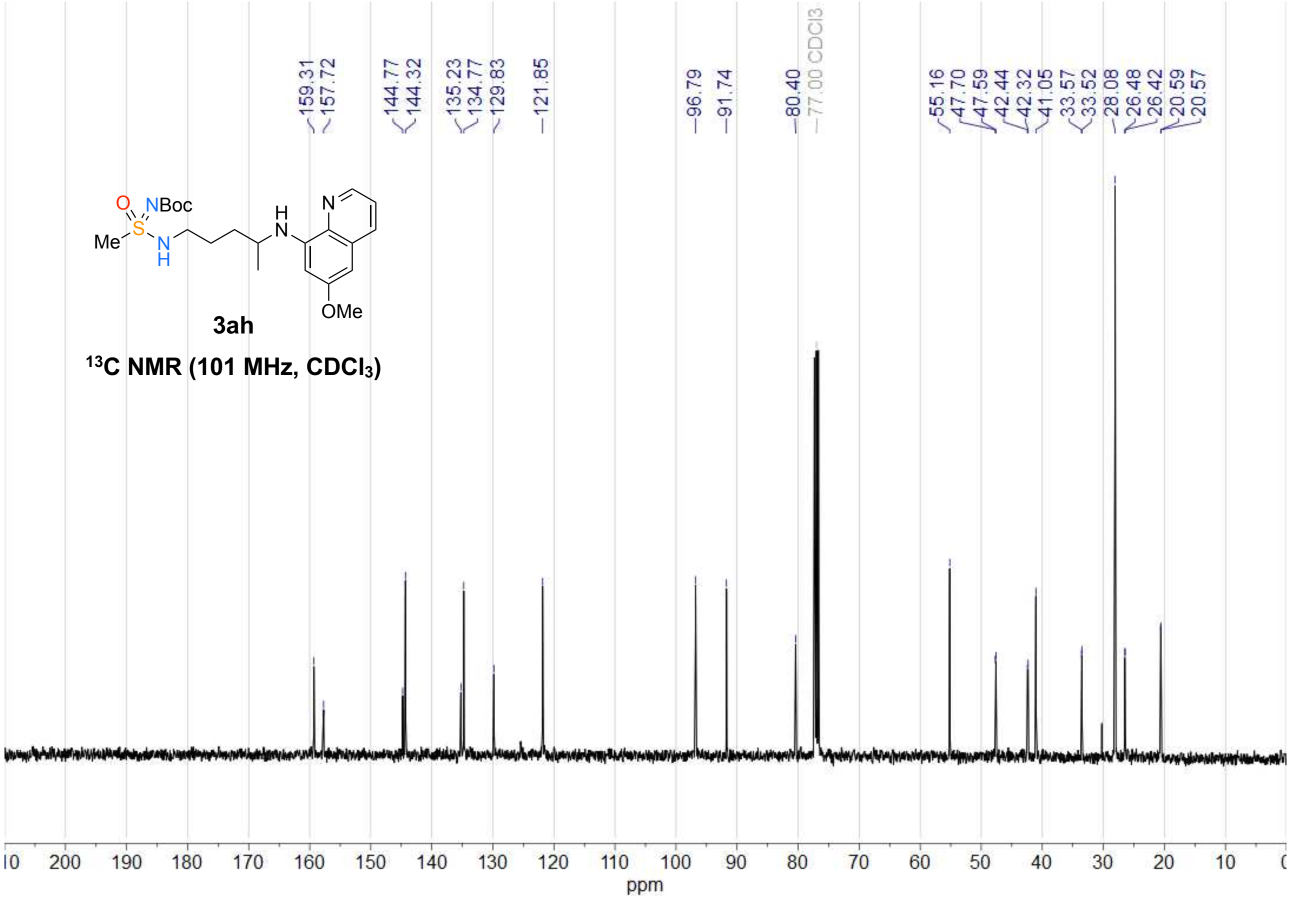

tert-Butyl (((3-(10,11-dihydro-5*H*-dibenzo[*b*,*f*]azepin-5-yl)propyl)(methyl)amino)(methyl)(oxo)-λ⁶-sulfaneylidene)carbamate (3ai)

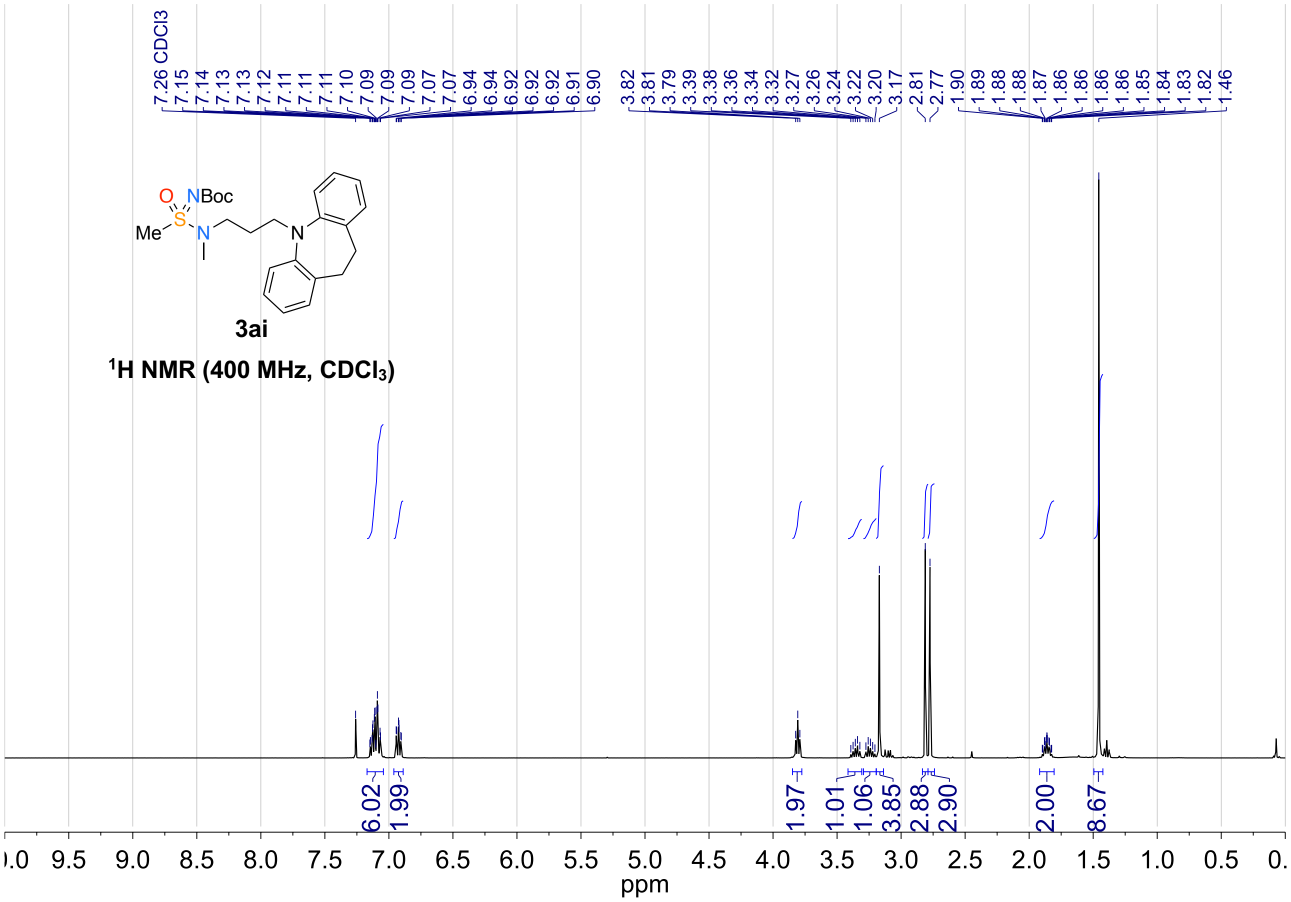

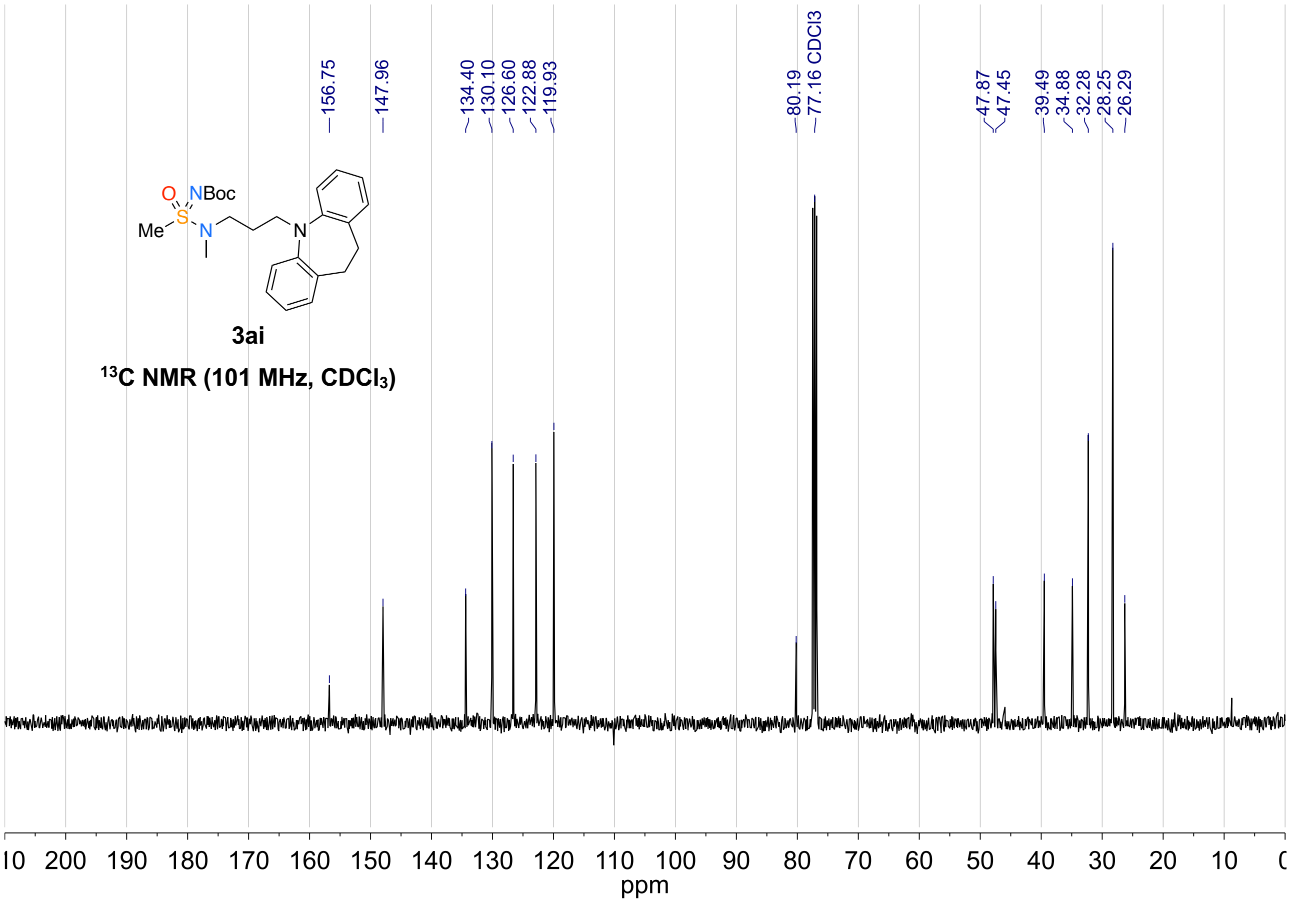

tert-Butyl ((4-(2-chlorodibenzo[b,f][1,4]oxazepin-11-yl)piperazin-1-yl)(methyl)(oxo)-λ^6-sulfaneylidene)carbamate (3aj)

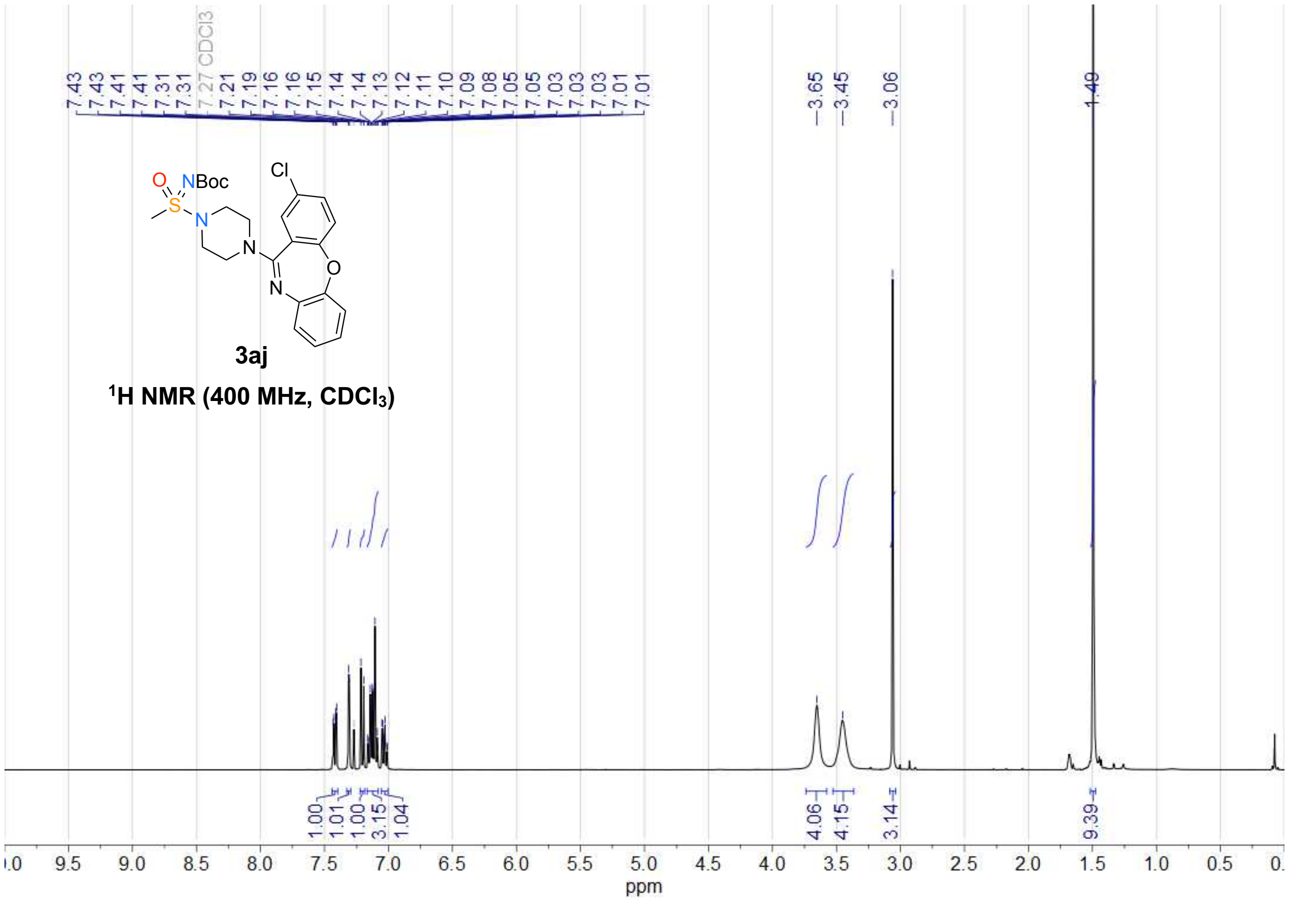

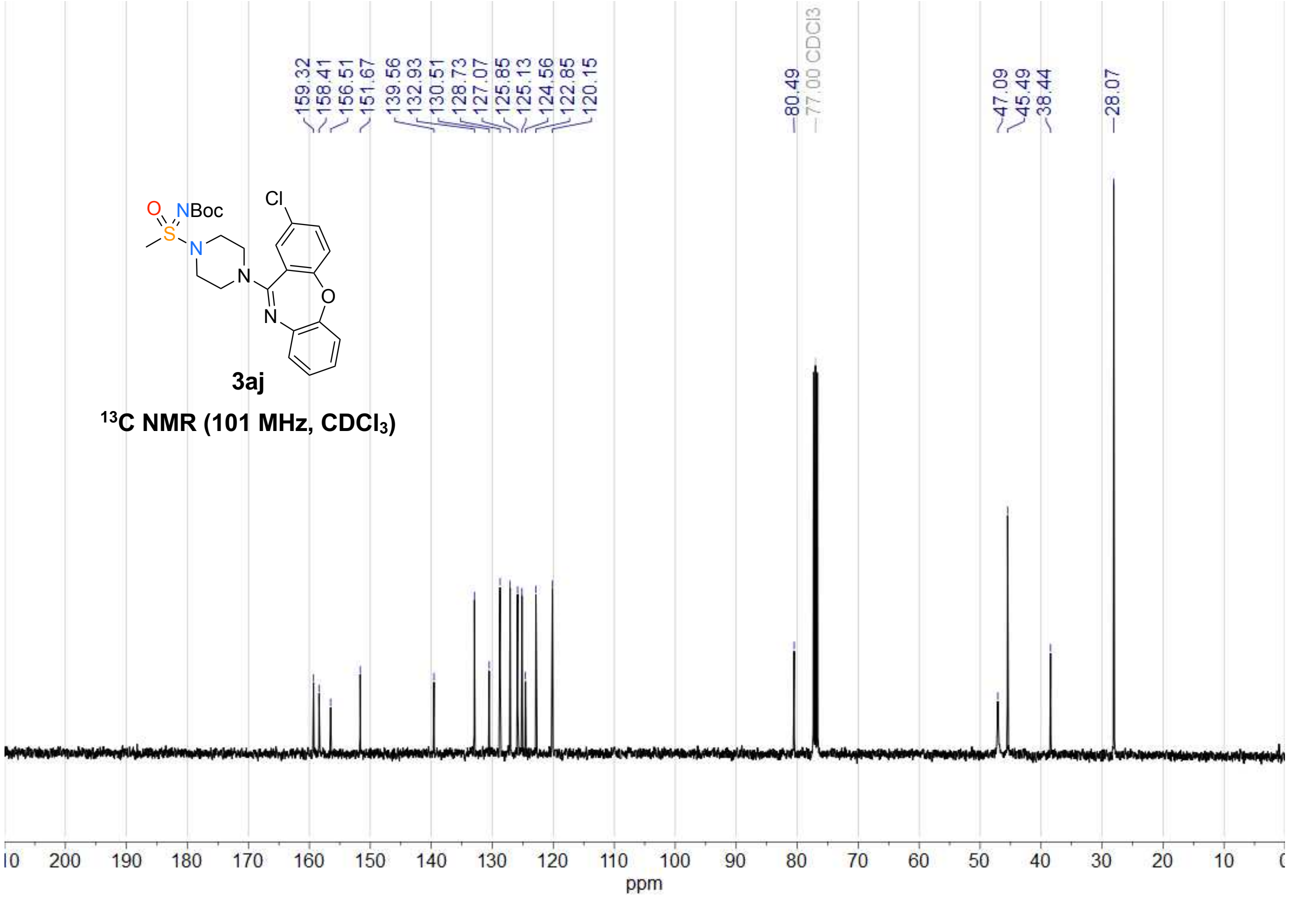

(*R*)-1-(4-Methylphenylsulfonimidoyl)piperidine ((*R*)-11)

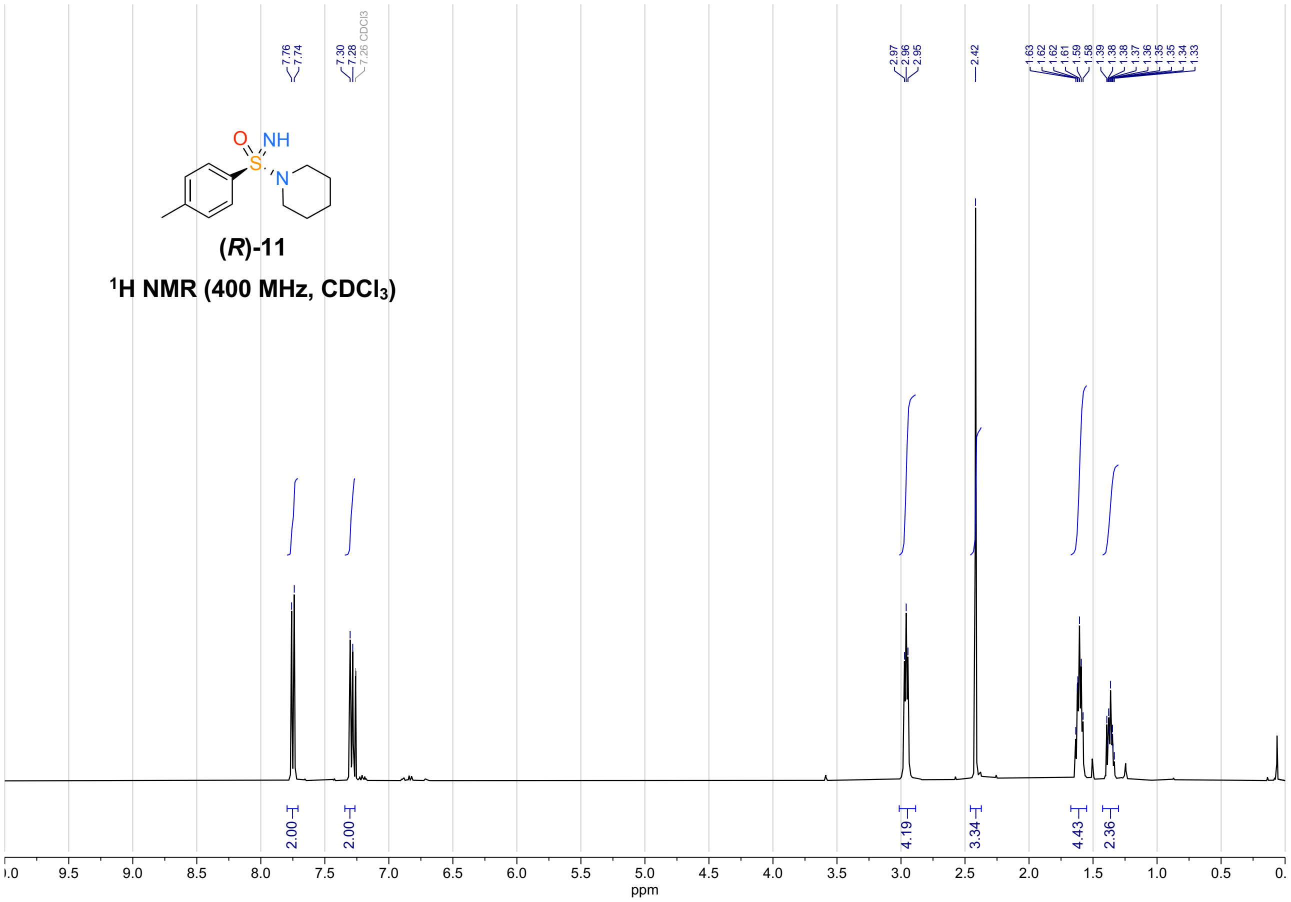

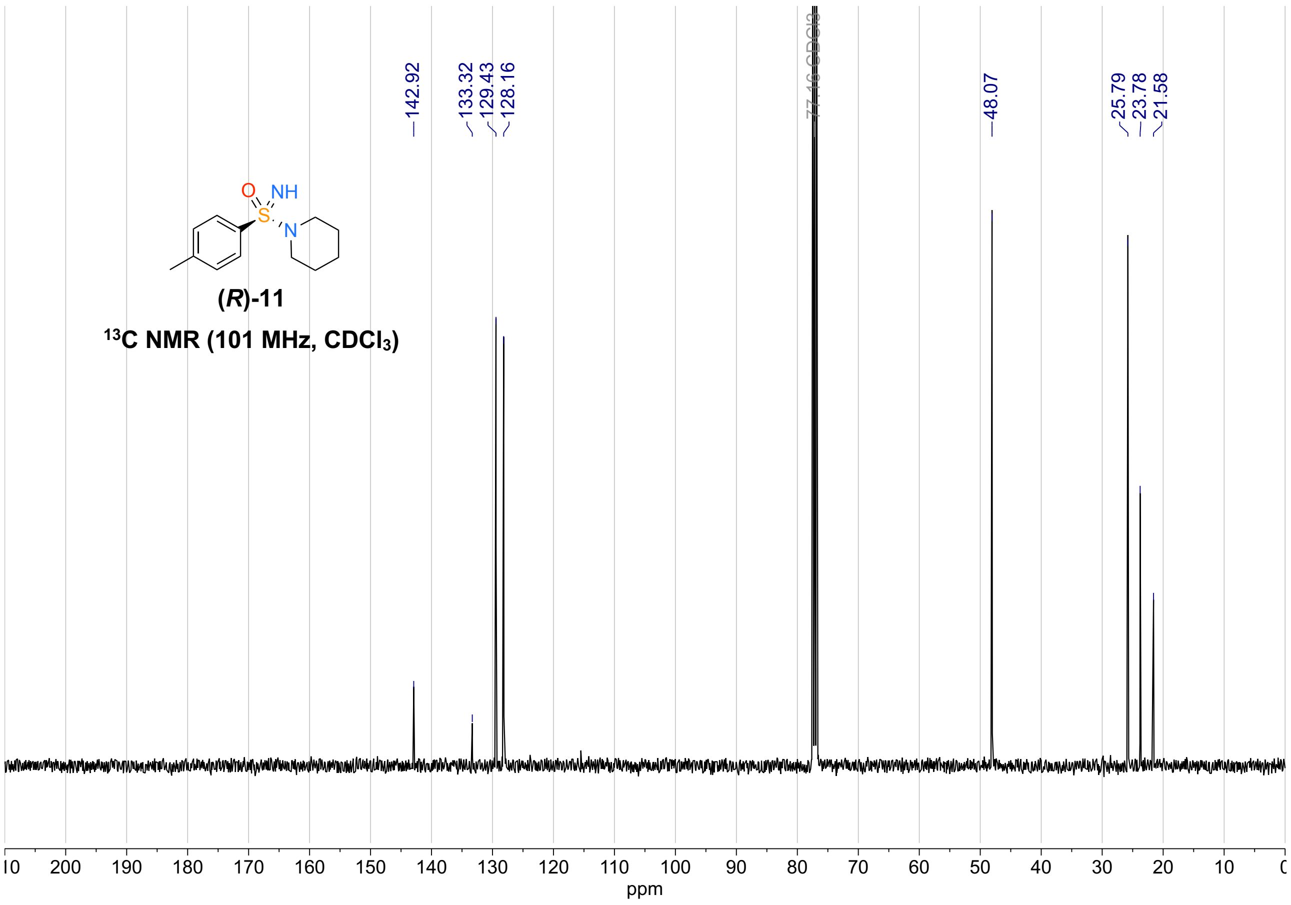

(*R*)-*N'*-Cyclobutyl-4-methylbenzenesulfonimidamide ((*R*)-12)

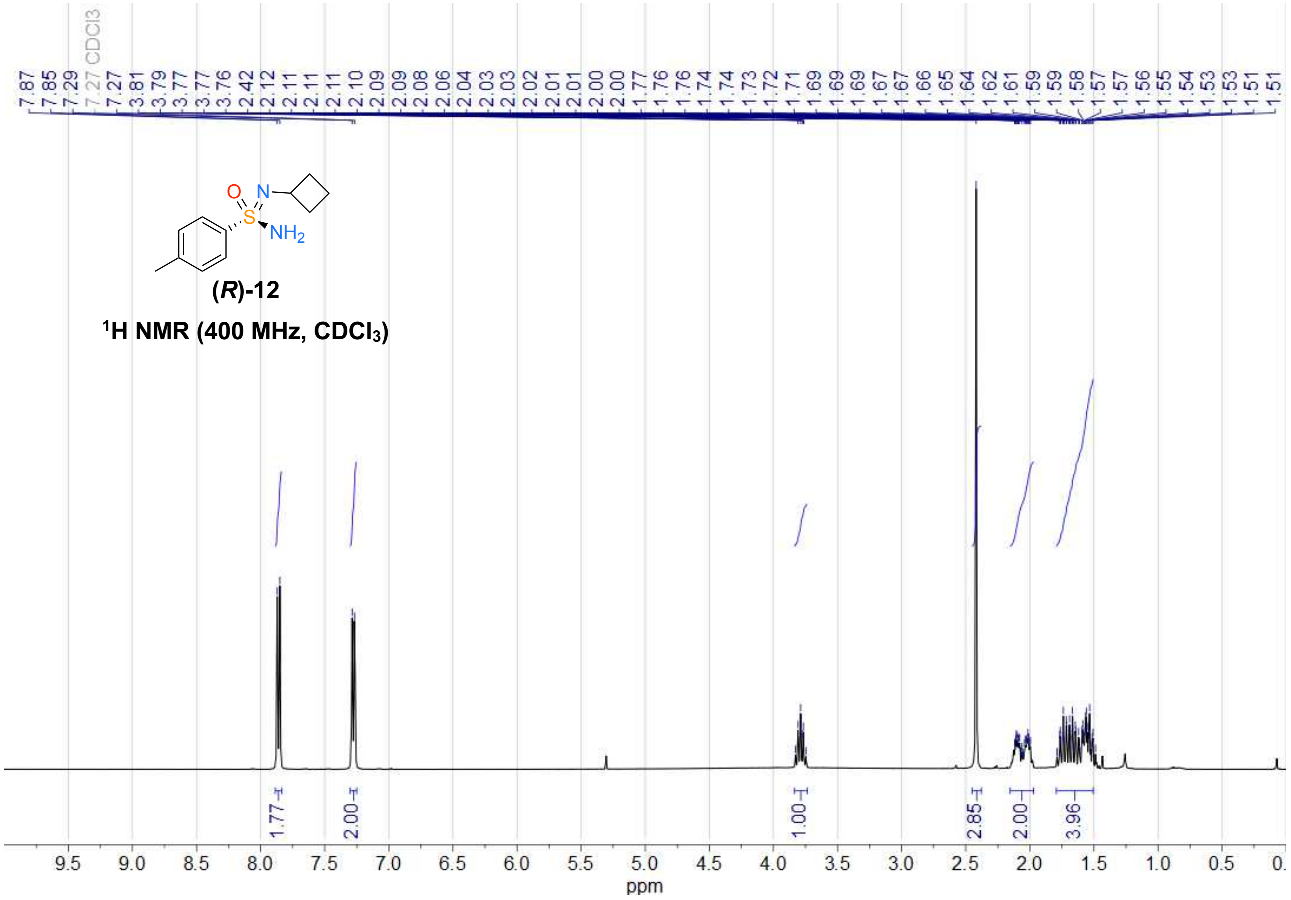

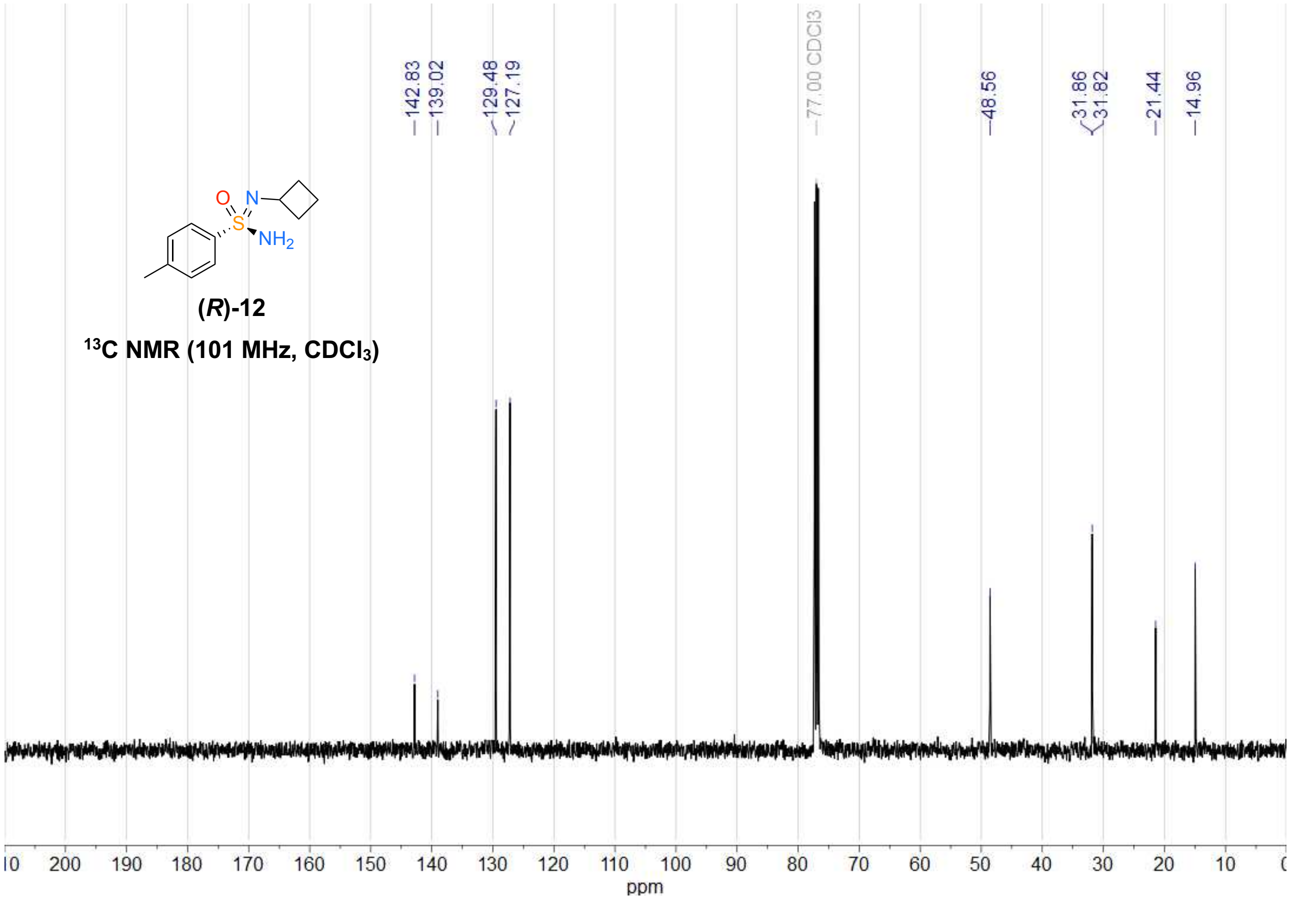

HPLC Data

HPLC Data

***tert*-Butyl (*p*-tolylsulfinyl)carbamate ((*S*)-5)**

Conditions: Chiralpak IB column, 98:2 *n*hexane:*i*PrOH, flow rate: 1 mL min^{-1}, 35 °C, UV detection wavelength: 250 nm.

(*rac*)-5

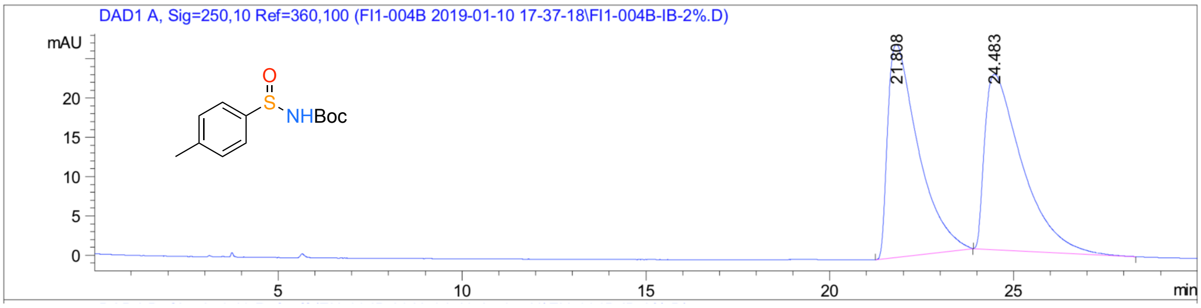

Signal 1: DAD1 A, Sig=250,10 Ref=360,100

Peak #	RetTime [min]	Type	Width [min]	Area [mAU*s]	Height [mAU]	Area %
1	21.808	BB	0.8231	1568.53638	27.10690	49.7337
2	24.483	BB	0.9969	1585.33215	22.31392	50.2663
Totals :				3153.86853	49.42082	

(*S*)-5

$[\alpha]^{21}_{D}$ = +80 (c 0.1, $CHCl_3$).

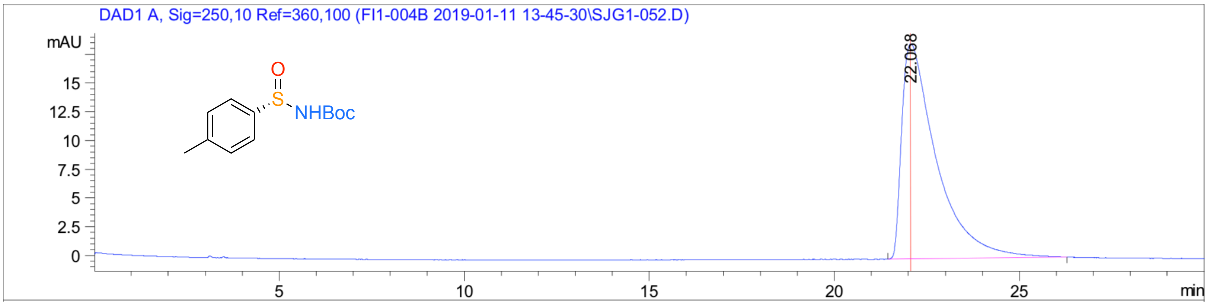

Signal 1: DAD1 A, Sig=250,10 Ref=360,100

Peak #	RetTime [min]	Type	Width [min]	Area [mAU*s]	Height [mAU]	Area %
1	22.068	BB	0.9253	1221.97766	18.72633	100.0000
Totals :				1221.97766	18.72633	

ee > 99%

tert-Butyl (fluoro(oxo)(p-tolyl)-λ^6-sulfaneylidene)carbamate ((R)-2a)

Conditions: Chiralpak IA column, 99:1 *n*hexane:*i*PrOH, flow rate: 1 mL min^{-1}, 35 °C, UV detection wavelength: 250 nm.

(*rac*)-2a

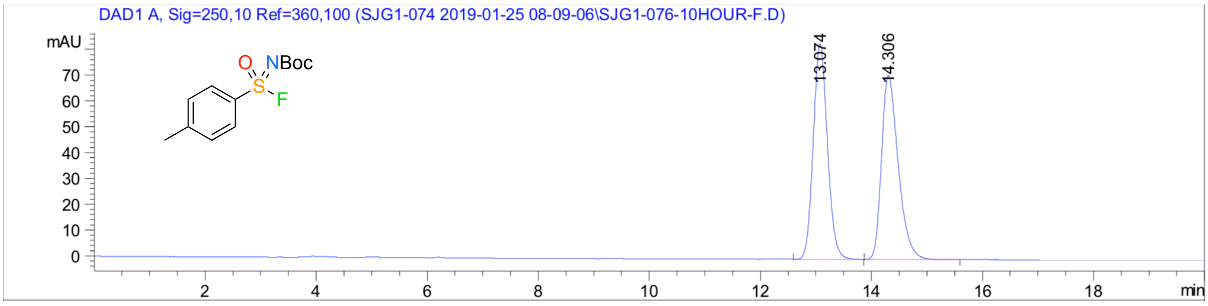

Signal 1: DAD1 A, Sig=250,10 Ref=360,100

Peak #	RetTime [min]	Type	Width [min]	Area [mAU*s]	Height [mAU]	Area %
1	13.074	BB	0.2726	1481.25183	83.62629	49.9140
2	14.306	BB	0.3176	1486.35461	71.18559	50.0860
Totals :				2967.60645	154.81188	

(*R*)-2a

$[\alpha]^{21}_D$ = +9 (c 5.0, $CHCl_3$).

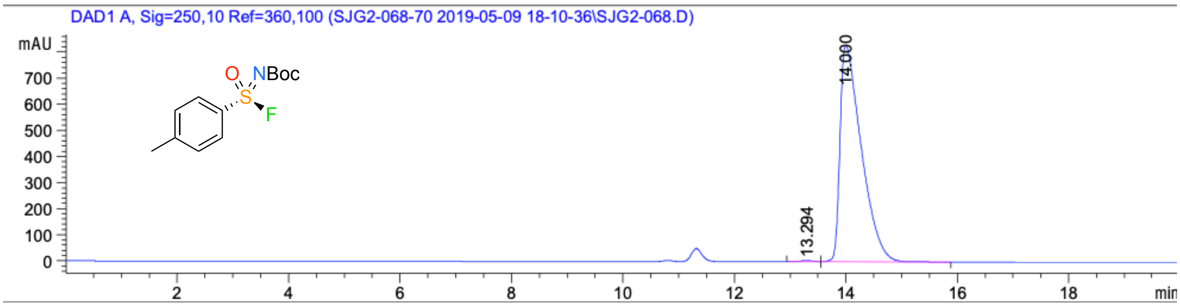

Signal 1: DAD1 A, Sig=250,10 Ref=360,100

Peak #	RetTime [min]	Type	Width [min]	Area [mAU*s]	Height [mAU]	Area %
1	13.294	BV	0.2625	78.58254	4.71449	0.3559
2	14.000	VB	0.3970	2.20035e4	830.72552	99.6441
Totals :				2.20821e4	835.44002	

***ee* > 99%**

tert-Butyl (*R*)-(oxo(piperidin-1-yl)(*p*-tolyl-λ^6-sulfaneylidene)carbamate ((*R*)-3a)

Conditions: Chiralpak IA column, 95:5 *n*hexane:*i*PrOH, flow rate: 1 mL min^{-1}, 35 °C, UV detection wavelength: 270 nm.

(*rac*)-(3a)

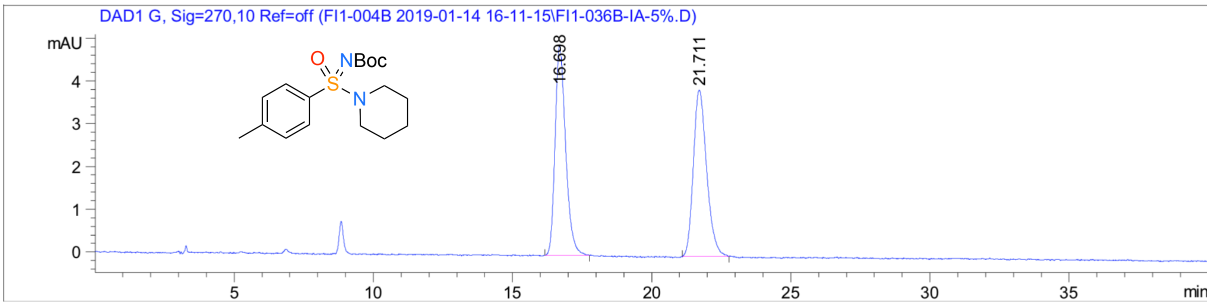

Signal 7: DAD1 G, Sig=270, 10 Ref=off

Peak #	RetTime [min]	Type	Width [min]	Area [mAU*s]	Height [mAU]	Area %
1	16.698	BB	0.3925	130.26352	4.92661	50.1539
2	21.711	BB	0.4862	129.46416	3.89039	49.8461
Totals :				259.72768	8.81699	

(*R*)-(3a)

$[\alpha]^{21}_D$ = -18 (c 0.5, $CHCl_3$).

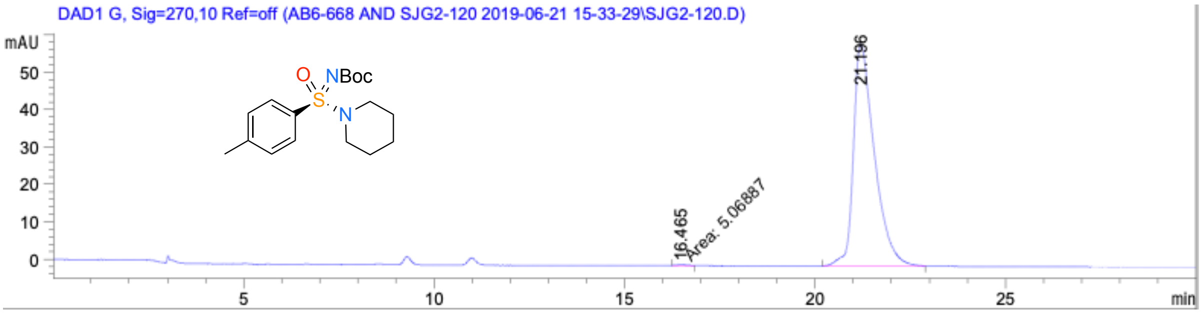

Signal 7: DAD1 G, Sig=270,10 Ref=off

Peak #	RetTime [min]	Type	Width [min]	Area [mAU*s]	Height [mAU]	Area %
1	16.465	MM	0.3392	5.06887	2.49059e-1	0.2228
2	21.196	BB	0.5707	2269.86426	59.28167	99.7772
Totals :				2274.93313	59.53072	

***ee* > 99%**

tert-Butyl (*R*)-((butylamino)(oxo)(*p*-tolyl)-λ^6-sulfaneylidene)carbamate ((*R*)-3b)

Conditions: Chiralpak IA column, 97:3 *n*hexane:*i*PrOH, flow rate: 1 mL min^{-1}, 35 °C, UV detection wavelength: 254 nm.

(*rac*)-(3b)

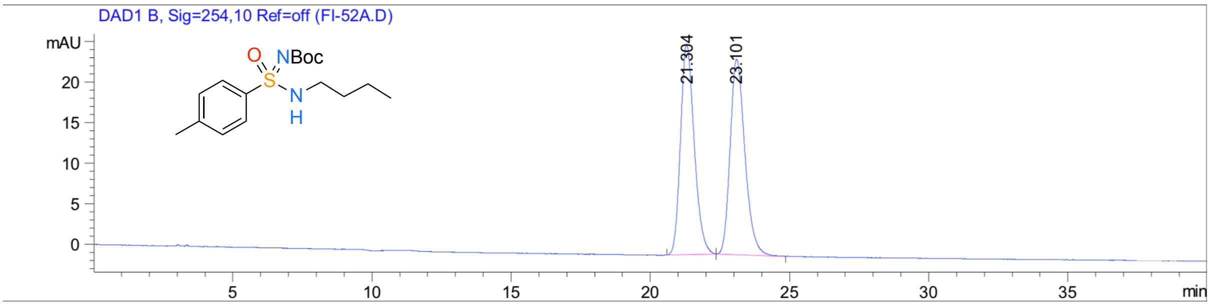

Signal 1: DAD1 B, Sig=254,10 Ref=off

Peak #	RetTime [min]	Type	Width [min]	Area [mAU*s]	Height [mAU]	Area %
1	21.304	BB	0.5260	887.74835	25.88978	49.9281
2	23.101	BB	0.5633	890.30682	24.08071	50.0719
Totals :				1778.05518	49.97049	

(*R*)-(3b)

$[\alpha]^{21}_{D}$ = +42 (c 1.0, $CHCl_3$).

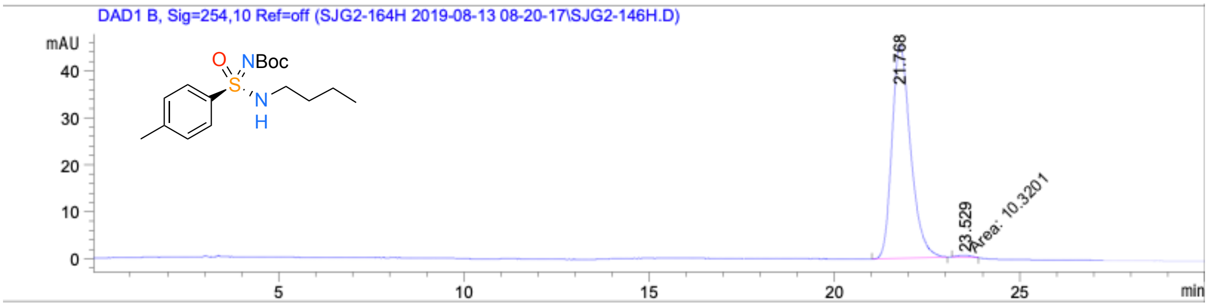

Signal 2: DAD1 B, Sig=254,10 Ref=off

Peak #	RetTime [min]	Type	Width [min]	Area [mAU*s]	Height [mAU]	Area %
1	21.768	BB	0.5266	1584.74768	45.46916	99.3530
2	23.529	MM	0.4161	10.32006	4.13375e-1	0.6470
Totals :				1595.06774	45.88253	

***ee* = 99%**

tert-Butyl (R)-((benzylamino)(oxo)(p-tolyl)-λ^6-sulfaneylidene)carbamate ((R)-3c)

Conditions: Chiralpak IA column, 97:3 *n*hexane:*i*PrOH, flow rate: 1 mL min^{-1}, 35 °C, UV detection wavelength: 250 nm.

(*rac*)-(3c)

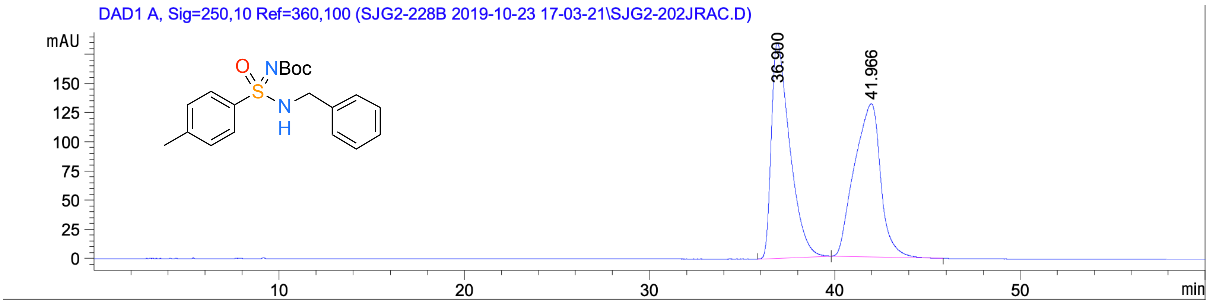

Signal 1: DAD1 A, Sig=250,10 Ref=360,100

Peak #	RetTime [min]	Type	Width [min]	Area [mAU*s]	Height [mAU]	Area %
1	36.900	BB	1.0203	1.27760e4	184.40756	49.7300
2	41.966	BB	1.3526	1.29147e4	131.17830	50.2700
Totals :				2.56908e4	315.58586	

(*R*)-(3c)

$[\alpha]^{23}_{D}$ = +88 (c 0.5, $CHCl_3$).

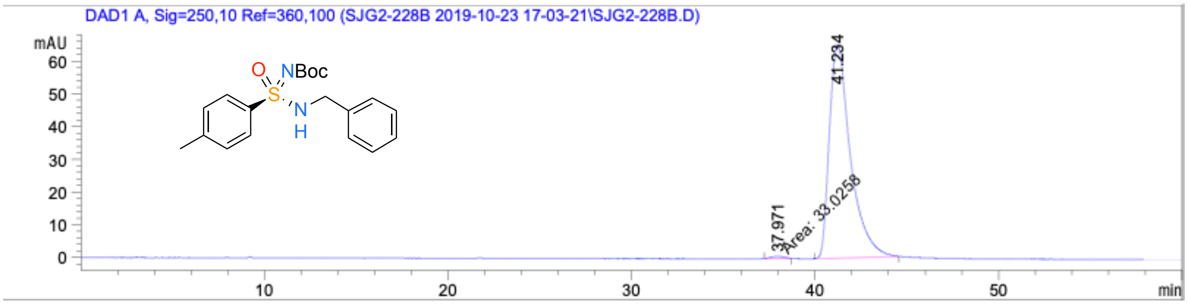

Signal 1: DAD1 A, Sig=250,10 Ref=360,100

Peak #	RetTime [min]	Type	Width [min]	Area [mAU*s]	Height [mAU]	Area %
1	37.971	MM	0.8366	33.02581	6.57922e-1	0.6327
2	41.234	BB	1.1786	5186.88770	65.16691	99.3673
Totals :				5219.91351	65.82483	

***ee* = 99%**

tert-Butyl (_R_)-((allylamino)(oxo)(_p_-tolyl)-λ⁶-sulfaneylidene)carbamate ((_R_)-3d)

Conditions: Chiralpak IA column, 97:3 *n*hexane:*i*PrOH, flow rate: 1 mL min^{-1}, 35 °C, UV detection wavelength: 270 nm.

(_rac_)-(3d)

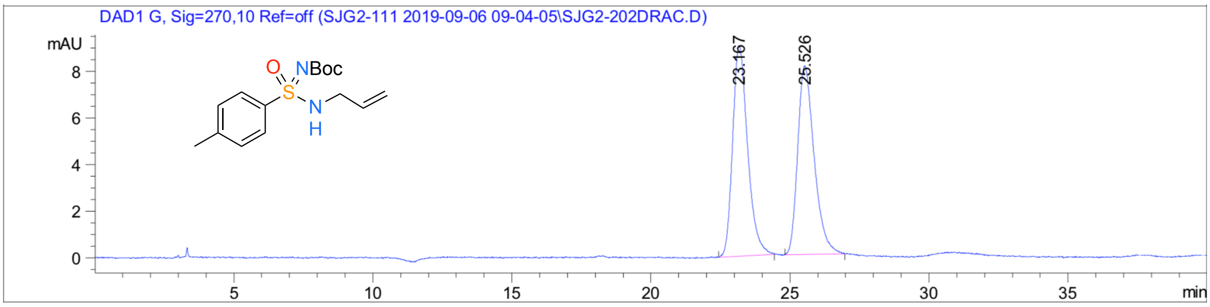

Signal 7: DAD1 G, Sig=270, 10 Ref=off

Peak #	RetTime [min]	Type	Width [min]	Area [mAU*s]	Height [mAU]	Area %
1	23.167	BB	0.5364	338.05084	9.03798	50.3426
2	25.526	BB	0.5708	333.45023	8.09127	49.6574
Totals :				671.50107	17.12926	

(_R_)-(3d)

$[\alpha]^{23}_{D}$ = +40 (c 0.5, $CHCl_3$).

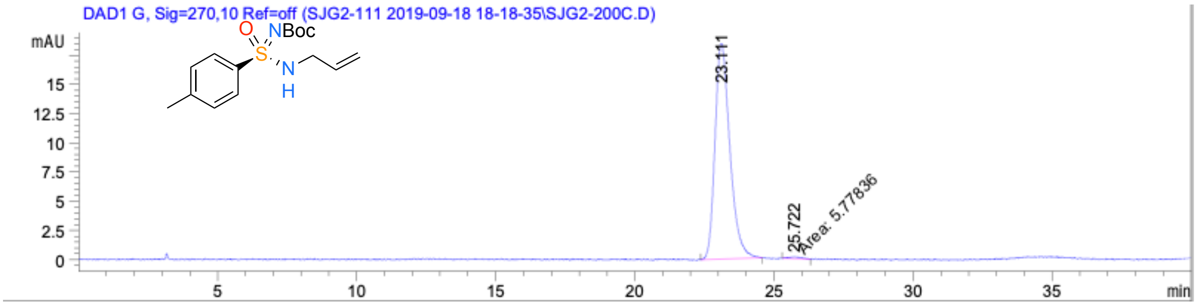

Signal 7: DAD1 G, Sig=270,10 Ref=off

Peak #	RetTime [min]	Type	Width [min]	Area [mAU*s]	Height [mAU]	Area %
1	23.111	BB	0.5258	684.38165	18.49214	99.1628
2	25.722	MM	0.5570	5.77836	1.72910e-1	0.8372
Totals :				690.16001	18.66505	

***ee* = 98%**

tert-Butyl (*R*)-(((cyclopropylmethyl)amino)(oxo)(*p*-tolyl)-λ^6-sulfaneylidene)carbamate ((*R*)-3e)

Conditions: Chiralpak IA column, 97:3 *n*hexane:*i*PrOH, flow rate: 1 mL min^{-1}, 35 °C, UV detection wavelength: 280 nm.

(*rac*)-(3e)

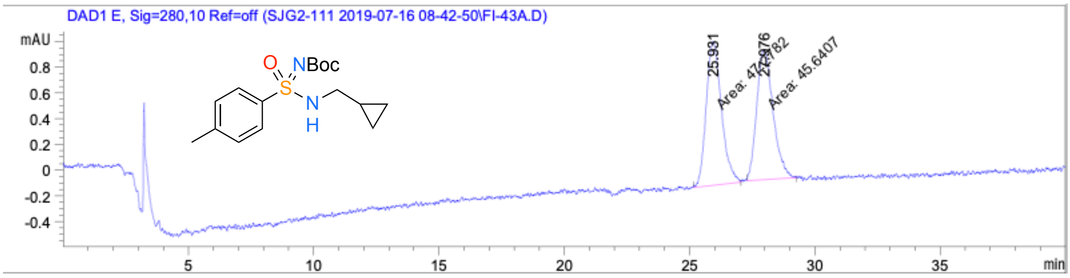

Signal 1: DAD1 A, Sig=250,10 Ref=360,100

Peak #	RetTime [min]	Type	Width [min]	Area [mAU*s]	Height [mAU]	Area %
1	25.929	BB	0.6237	1115.89270	26.91534	50.0552
2	27.963	BB	0.6729	1113.43213	24.46601	49.9448
Totals :				2229.32483	51.38135	

(*R*)-(3e)

$[\alpha]^{21}_D$ = +29 (c 1.0, $CHCl_3$).

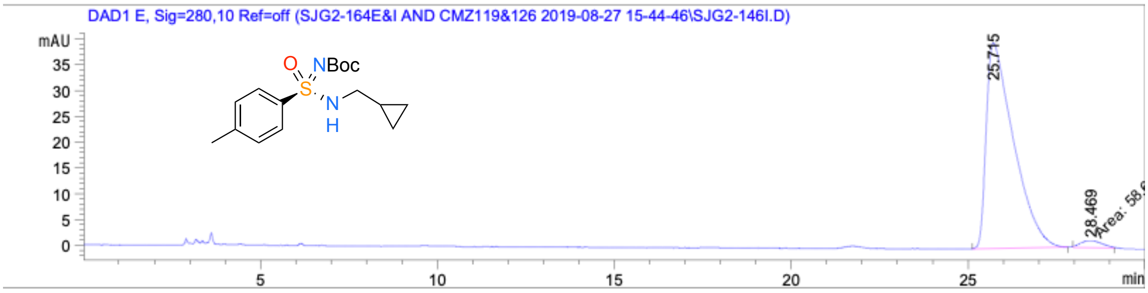

Signal 5: DAD1 E, Sig=280,10 Ref=off

Peak #	RetTime [min]	Type	Width [min]	Area [mAU*s]	Height [mAU]	Area %
1	25.715	BB	0.7456	2133.07471	39.90491	97.3256
2	28.469	MM	0.6996	58.61450	1.39648	2.6744

***ee* = 95%**

tert-Butyl (_R_)-((cyclobutylamino)(oxo)(_p_-tolyl)-λ^6-sulfaneylidene)carbamate ((_R_)-3f)

Conditions: Chiralpak IA column, 95:5 *n*hexane:*i*PrOH, flow rate: 1 mL min^{-1}, 35 °C, UV detection wavelength: 260 nm.

(_rac_)-(3f)

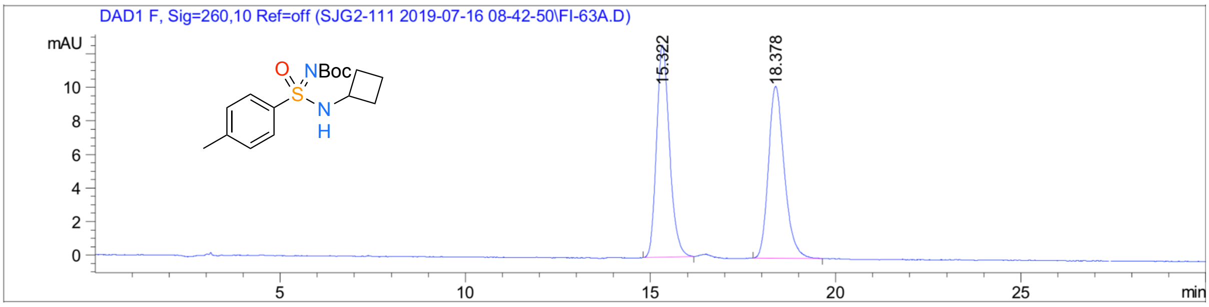

Signal 6: DAD1 F, Sig=260,10 Ref=off

Peak #	RetTime [min]	Type	Width [min]	Area [mAU*s]	Height [mAU]	Area %
1	15.322	BB	0.3697	307.63245	12.64949	50.1173
2	18.378	BB	0.4523	306.19284	10.25480	49.8827
Totals :				613.82529	22.90428	

(_R_)-(3f)

$[\alpha]^{21}_D$ = +48 (c 0.8, $CHCl_3$).

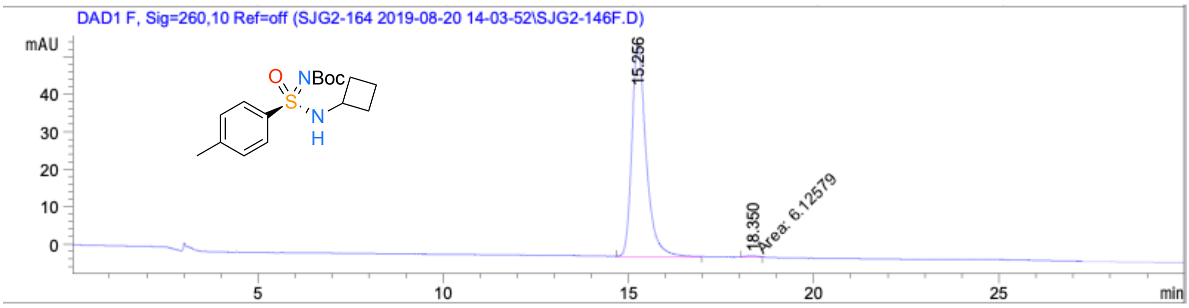

Signal 6: DAD1 F, Sig=260,10 Ref=off

Peak #	RetTime [min]	Type	Width [min]	Area [mAU*s]	Height [mAU]	Area %
1	15.256	BB	0.4059	1500.06885	56.10583	99.5933
2	18.350	MM	0.3520	6.12579	2.90021e-1	0.4067
Totals :				1506.19464	56.39585	

***ee* > 99%**

tert-Butyl (R)-((cyclohexylamino)(oxo)(p-tolyl)-λ⁶-sulfaneylidene)carbamate ((R)-3g)

Conditions: Chiralpak IF column, 95:5 *n*hexane:*i*PrOH, flow rate: 1 mL min^{-1}, 35 °C, UV detection wavelength: 254 nm.

(*rac*)-(3g)

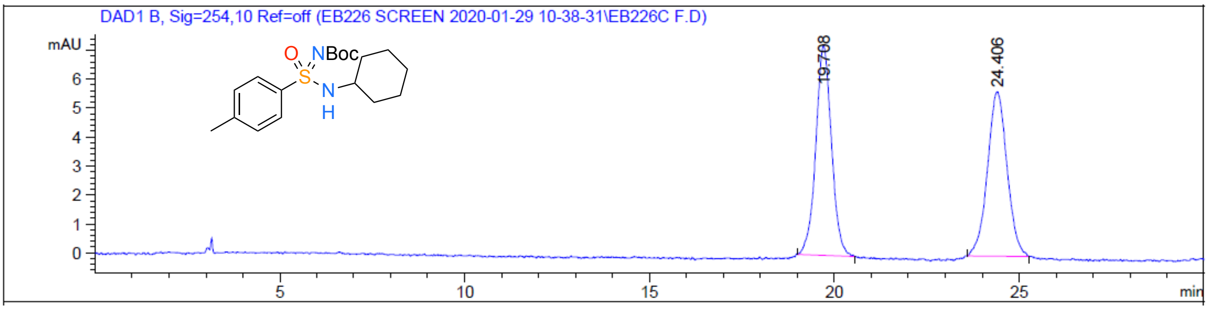

Signal 2: DAD1 B, Sig=254,10 Ref=off

Peak #	RetTime [min]	Type	Width [min]	Area [mAU*s]	Height [mAU]	Area %
1	19.708	BB	0.4301	213.50233	7.23872	50.5042
2	24.406	BB	0.4724	209.23958	5.67202	49.4958
Totals :				422.74191	12.91074	

(*R*)-(3g)

$[\alpha]^{21}_D$ = +42 (c 0.5, $CHCl_3$).

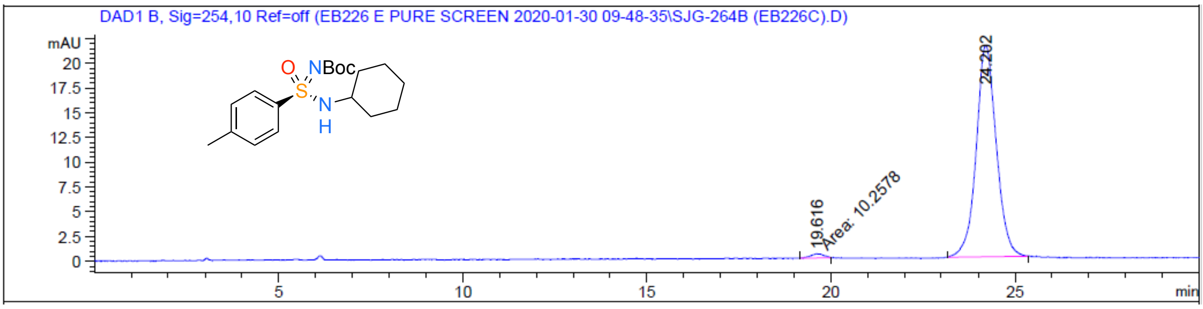

Signal 2: DAD1 B, Sig=254,10 Ref=off

Peak #	RetTime [min]	Type	Width [min]	Area [mAU*s]	Height [mAU]	Area %
1	19.616	MP	0.4110	10.25777	4.15939e-1	1.2543
2	24.202	BB	0.5475	807.56189	21.34123	98.7457
Totals :				817.81966	21.75717	

***ee* = 97%**

tert-Butyl (R)-((dimethylamino)(oxo)(p-tolyl)-λ^6-sulfaneylidene)carbamate ((R)-3h)

Conditions: Chiralpak IA column, 97:3 *n*hexane:*i*PrOH, flow rate: 1 mL min^{-1}, 35 °C, UV detection wavelength: 250 nm.

(*rac*)-(3h)

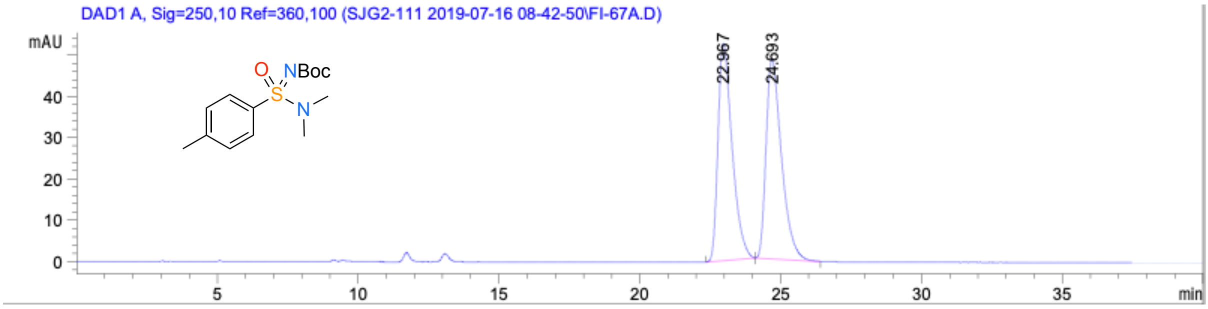

Signal 1: DAD1 A, Sig=250,10 Ref=360,100

Peak #	RetTime [min]	Type	Width [min]	Area [mAU*s]	Height [mAU]	Area %
1	22.967	BB	0.5220	1830.77698	52.61246	49.8946
2	24.693	BB	0.5691	1838.50952	48.41205	50.1054
Totals :				3669.28650	101.02451	

(*R*)-(3h)

$[\alpha]^{21}_D$ = -28 (c 1.0, $CHCl_3$)

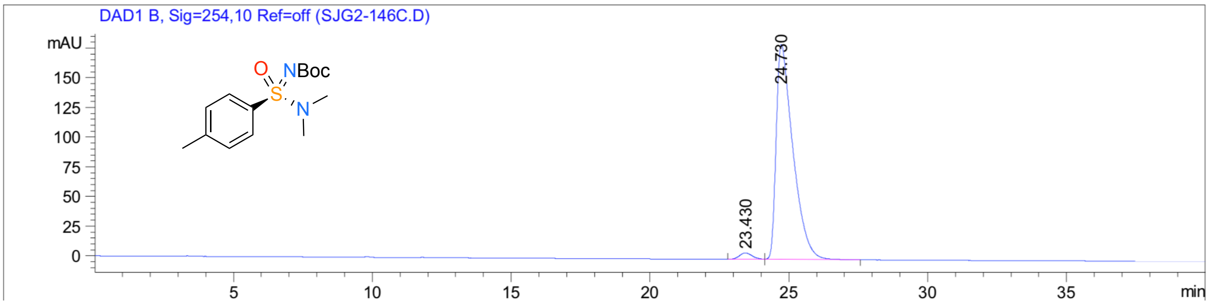

Signal 1: DAD1 B, Sig=254,10 Ref=off

Peak #	Ret Time [min]	Type	Width [min]	Area [mAU*s]	Height [mAU]	Area %
1	23.430	BB	0.4528	167.01021	5.34144	2.1583
2	24.730	BB	0.6141	7570.95605	180.96532	97.8417
Totals :				7737.96626	186.30676	

***ee* = 96%**

tert-Butyl (_R_)-((benzyl(methyl)amino)(oxo)(_p_-tolyl)-λ^6-sulfaneylidene)carbamate ((_R_)-3i)

Conditions: Chiralpak IA column, 95:5 *n*hexane:*i*PrOH, flow rate: 1 mL min^{-1}, 35 °C, UV detection wavelength: 250 nm.

(_rac_)-(3i)

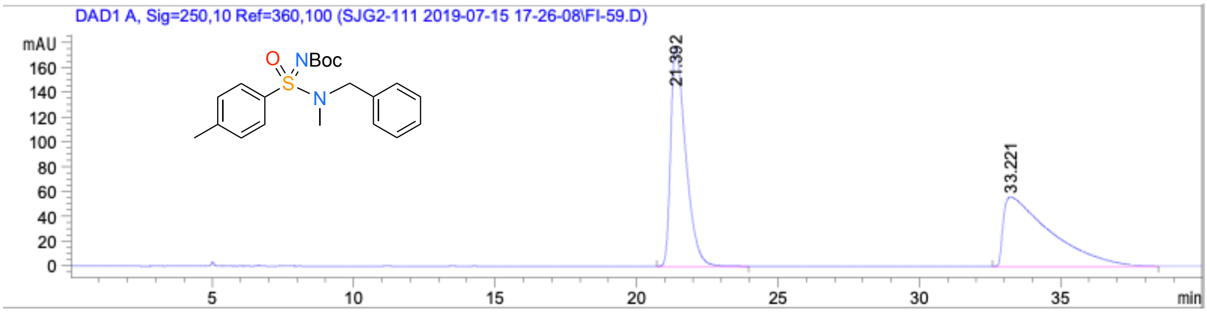

Signal 1: DAD1 A, Sig=250,10 Ref=360,100

Peak #	RetTime [min]	Type	Width [min]	Area [mAU*s]	Height [mAU]	Area %
1	21.392	BB	0.5504	6496.72705	177.73613	50.1472
2	33.221	BB	1.4833	6458.58838	55.92492	49.8528

Totals : 1.29553e4 233.66105

(_R_)-(3i)

$[\alpha]^{23}_D$ = –12 (c 1, $CHCl_3$).

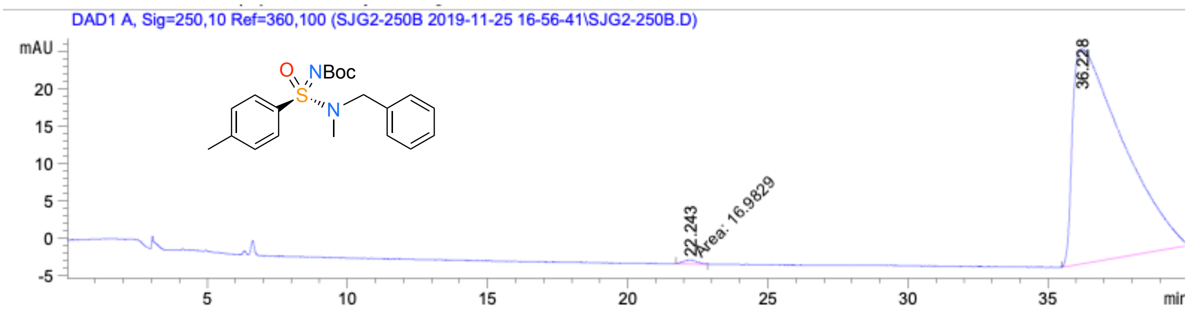

Signal 1: DAD1 A, Sig=250,10 Ref=360,100

Peak #	RetTime [min]	Type	Width [min]	Area [mAU*s]	Height [mAU]	Area %
1	22.243	MM	0.5453	16.98291	5.19109e-1	0.4725
2	36.228	BBA	1.4638	3577.22339	28.78534	99.5275

Totals : 3594.20630 29.30445

***ee* = 99%**

tert-Butyl (R)-((3,4-dihydroisoquinolin-2(1H)-yl)(oxo)(p-tolyl)-λ^6-sulfaneylidene)carbamate ((R)-3j)

Conditions: Chiralpak IA column, 95:5 *n*hexane:*i*PrOH, flow rate: 1 mL min^{-1}, 35 °C, UV detection wavelength: 250 nm.

(*rac*)-(3j)

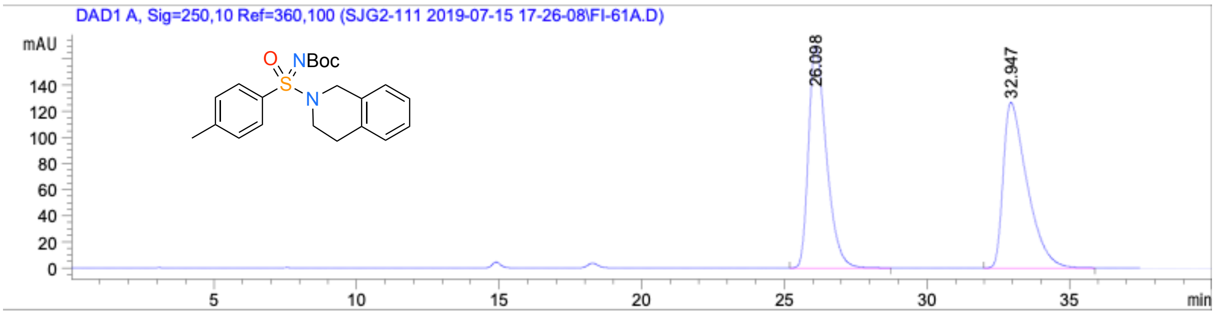

Signal 1: DAD1 A, Sig=250,10 Ref=360,100

Peak #	RetTime [min]	Type	Width [min]	Area [mAU*s]	Height [mAU]	Area %
1	26.098	BB	0.6530	7382.16113	170.54291	50.0445
2	32.947	BB	0.8578	7369.01855	126.83379	49.9555

Totals : 1.47512e4 297.37669

(*R*)-(3j)

$[\alpha]^{23}_D$ = 0 (c 1.0, $CHCl_3$).

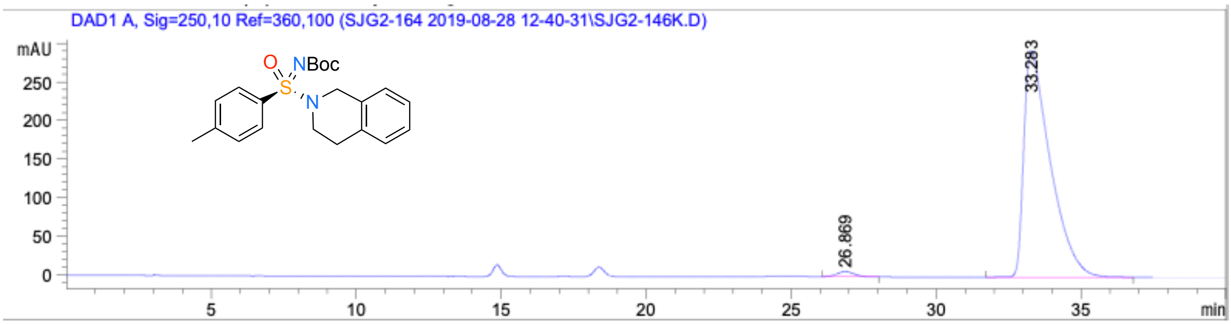

Signal 1: DAD1 A, Sig=250,10 Ref=360,100

Peak #	RetTime [min]	Type	Width [min]	Area [mAU*s]	Height [mAU]	Area %
1	26.869	BB	0.5728	270.96118	6.65785	1.4398
2	33.283	BB	0.9275	1.85491e4	293.42993	98.5602

Totals : 1.88200e4 300.08778

***ee* = 97%**

tert-Butyl (_R_)-(oxo(pyrrolidin-1-yl)(_p_-tolyl)-λ^6-sulfaneylidene)carbamate ((_R_)-3k)

Conditions: Chiralpak IA column, 95:5 *n*hexane:*i*PrOH, flow rate: 1 mL min^{-1}, 35 °C, UV detection wavelength: 260 nm.

(_rac_)-(3k)

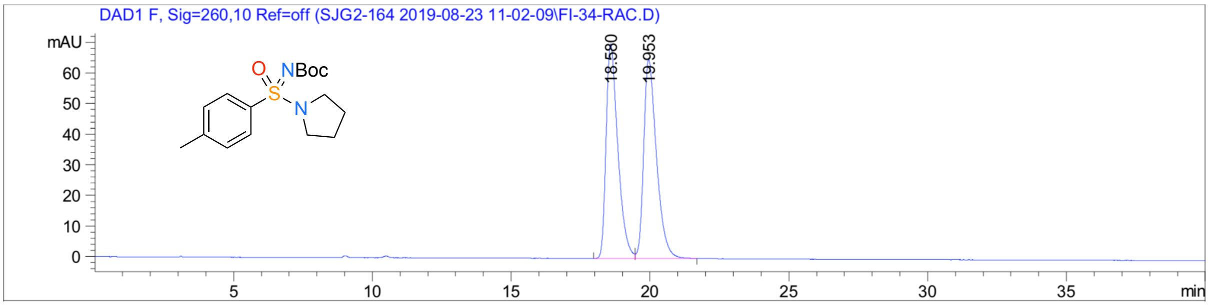

Signal 6: DAD1 F, Sig=260,10 Ref=off

Peak #	RetTime [min]	Type	Width [min]	Area [mAU*s]	Height [mAU]	Area %
1	18.580	BV	0.4467	2058.69604	70.07999	49.6798
2	19.953	VB	0.4841	2085.23169	65.02983	50.3202
Totals :				4143.92773	135.10983	

(_R_)-(3k)

$[\alpha]^{21}_D$ = -9 (c 1.0, $CHCl_3$).

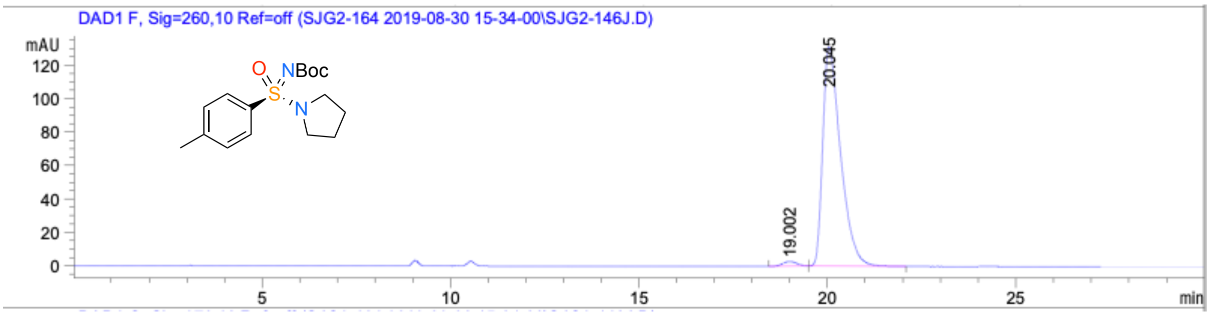

Signal 6: DAD1 F, Sig=260,10 Ref=off

Peak #	RetTime [min]	Type	Width [min]	Area [mAU*s]	Height [mAU]	Area %
1	19.002	BB	0.3669	74.11945	2.85495	1.6924
2	20.045	BB	0.4906	4305.41699	131.28296	98.3076
Totals :				4379.53644	134.13790	

***ee* = 97%**

tert-Butyl (R)-(morpholino(oxo)(p-tolyl)-λ^6-sulfaneylidene)carbamate ((R)-3l)

Conditions: Chiralpak IA column, 95:5 *n*hexane:*i*PrOH, flow rate: 1 mL min^{-1}, 35 °C, UV detection wavelength: 250 nm.

(rac)-(3l)

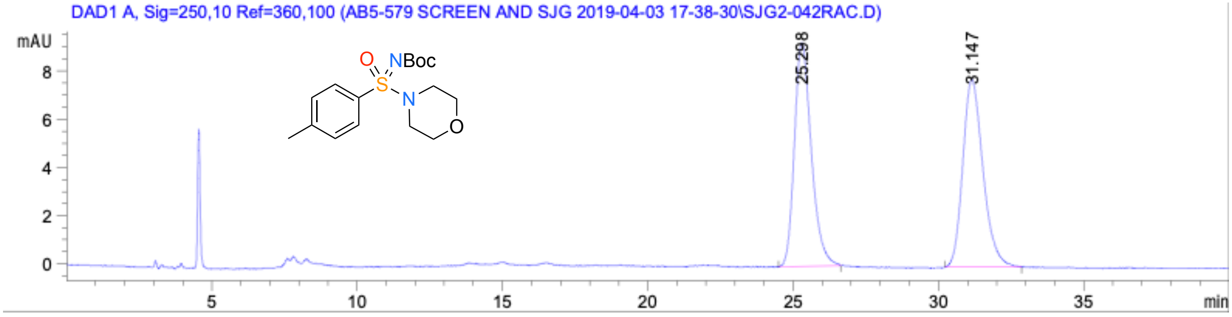

Signal 1: DAD1 A, Sig=250,10 Ref=360,100

Peak #	RetTime [min]	Type	Width [min]	Area [mAU*s]	Height [mAU]	Area %
1	25.298	BB	0.5799	373.90707	9.28350	49.8459
2	31.147	BB	0.6895	376.21826	7.78965	50.1541

Totals : 750.12534 17.07315

(R)-(3l)

[α]$^{23}_D$ = -11 (c 1.0, $CHCl_3$).

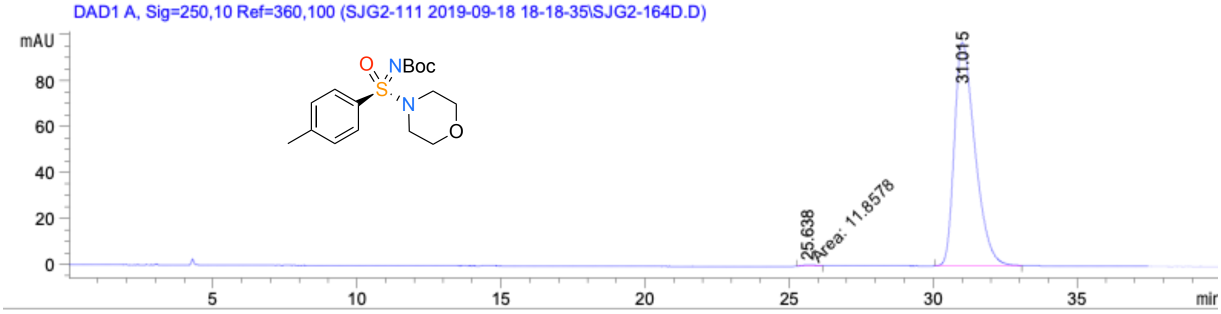

Signal 1: DAD1 A, Sig=250,10 Ref=360,100

Peak #	RetTime [min]	Type	Width [min]	Area [mAU*s]	Height [mAU]	Area %
1	25.638	MM	0.4908	11.85784	4.02664e-1	0.2452
2	31.015	BB	0.7408	4824.73779	97.24724	99.7548

Totals : 4836.59563 97.64990

***ee* > 99%**

tert-Butyl (*R*)-(oxo(4-oxopiperidin-1-yl)(*p*-tolyl)-λ^6-sulfaneylidene)carbamate ((*R*)-3m)

Conditions: Chiralpak IA column, 95:5 *n*hexane:*i*PrOH, flow rate: 1 mL min^{-1}, 35 °C, UV detection wavelength: 254 nm.

(*rac*)-(3m)

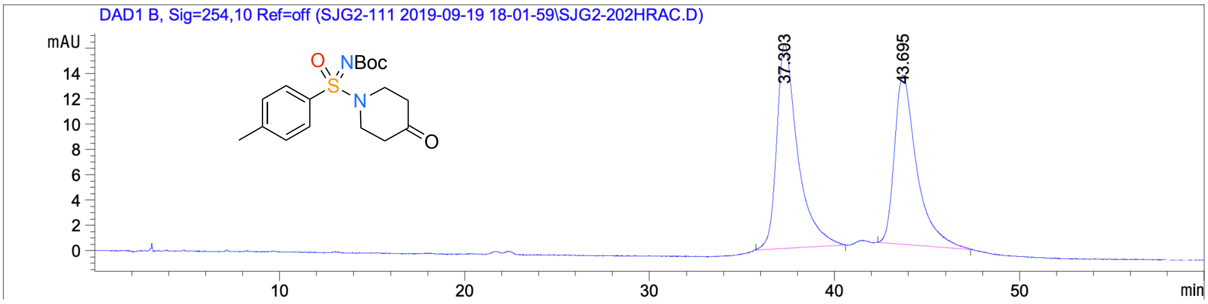

Signal 2: DAD1 B, Sig=254,10 Ref=off

Peak #	RetTime [min]	Type	Width [min]	Area [mAU*s]	Height [mAU]	Area %
1	37.303	BB	0.9859	1279.99353	16.16244	53.3272
2	43.695	BB	1.0028	1120.27039	13.38072	46.6728
Totals :				2400.26392	29.54317	

(*R*)-(3m)

$[\alpha]^{23}_D$ = 0 (c 0.2, $CHCl_3$).

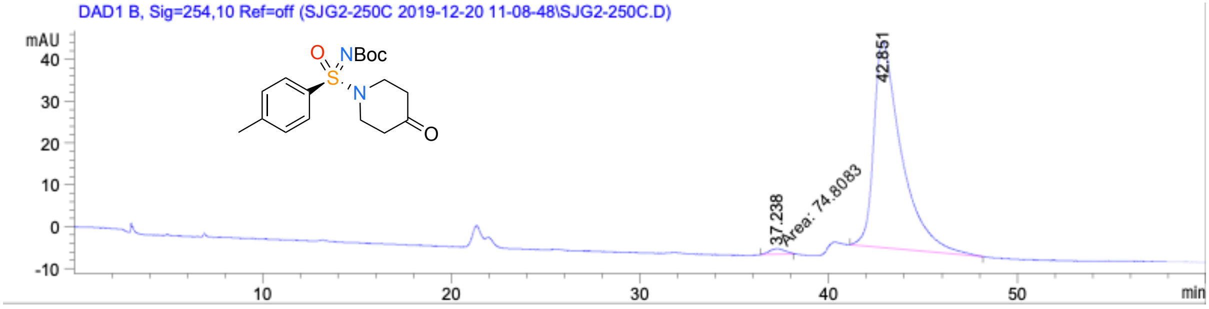

Signal 2: DAD1 B, Sig=254,10 Ref=off

Peak #	RetTime [min]	Type	Width [min]	Area [mAU*s]	Height [mAU]	Area %
1	37.238	MM	0.9614	74.80834	1.29684	1.4650
2	42.851	BB	1.3254	5031.48389	49.26838	98.5350
Totals :				5106.29223	50.56522	

***ee* = 97%**

tert-Butyl (_R_)-((4,4-difluoropiperidin-1-yl)(oxo)(_p_-tolyl)-λ^6-sulfaneylidene)carbamate ((_R_)-3n)

Conditions: Chiralpak IB column, 95:5 *n*hexane:*i*PrOH, flow rate: 1 mL min^{-1}, 35 °C, UV detection wavelength: 250 nm.

(_rac_)-(3n)

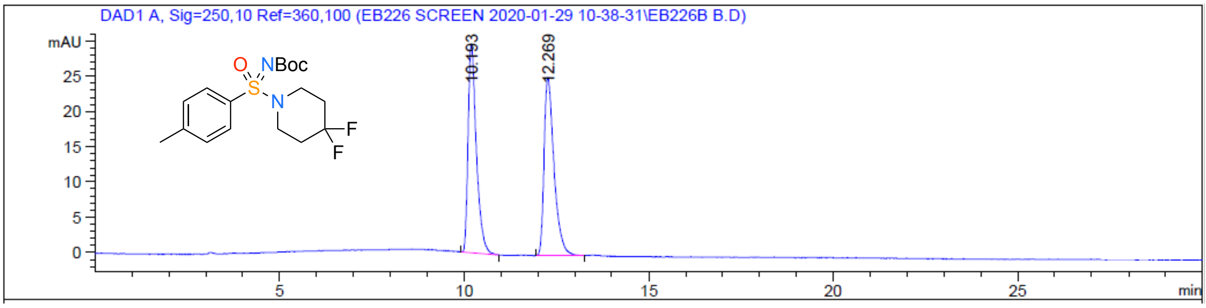

Signal 1: DAD1 A, Sig=250,10 Ref=360,100

Peak #	RetTime [min]	Type	Width [min]	Area [mAU*s]	Height [mAU]	Area %
1	10.193	BB	0.2356	460.88104	29.60700	49.3914
2	12.269	BB	0.2806	472.23984	25.20912	50.6086
Totals :				933.12088	54.81612	

(_R_)-(3n)

$[\alpha]^{23}_D$ = –6 (c 1, $CHCl_3$).

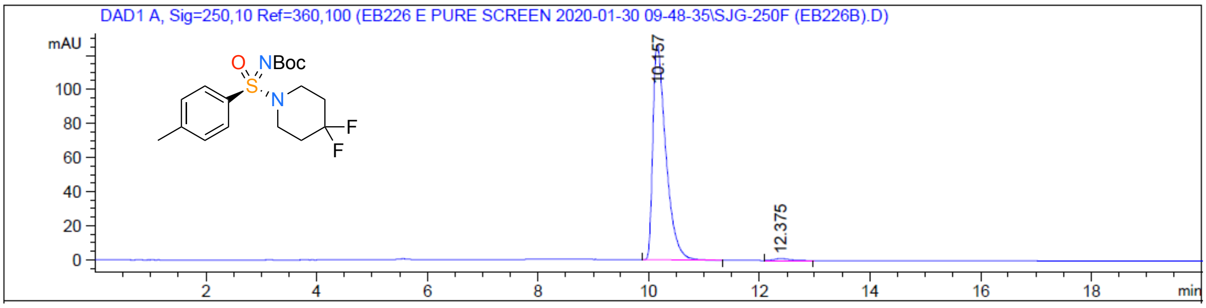

Signal 1: DAD1 A, Sig=250,10 Ref=360,100

Peak #	RetTime [min]	Type	Width [min]	Area [mAU*s]	Height [mAU]	Area %
1	10.157	BB	0.2463	2060.77588	126.33929	98.9847
2	12.375	BB	0.2460	21.13705	1.08157	1.0153
Totals :				2081.91293	127.42087	

***ee* = 98%**

tert-Butyl (_R_)-((4-hydroxypiperidin-1-yl)(oxo)(_p_-tolyl)-λ^6-sulfaneylidene)carbamate ((_R_)-3o)

Conditions: Chiralpak IA column, 85:15 *n*hexane:*i*PrOH, flow rate: 1 mL min^{-1}, 35 °C, UV detection wavelength: 254 nm.

(_rac_)-(3o)

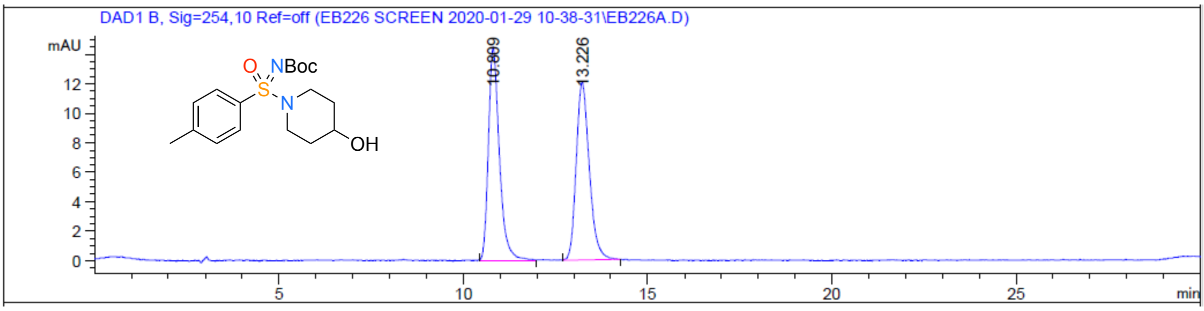

Signal 2: DAD1 B, Sig=254,10 Ref=off

Peak #	RetTime [min]	Type	Width [min]	Area [mAU*s]	Height [mAU]	Area %
1	10.809	BB	0.3085	297.77713	14.44385	50.0692
2	13.226	BB	0.3574	296.95435	12.05329	49.9308
Totals :				594.73148	26.49714	

(_R_)-(3o)

$[\alpha]^{23}_D$ = –4 (c 1, $CHCl_3$).

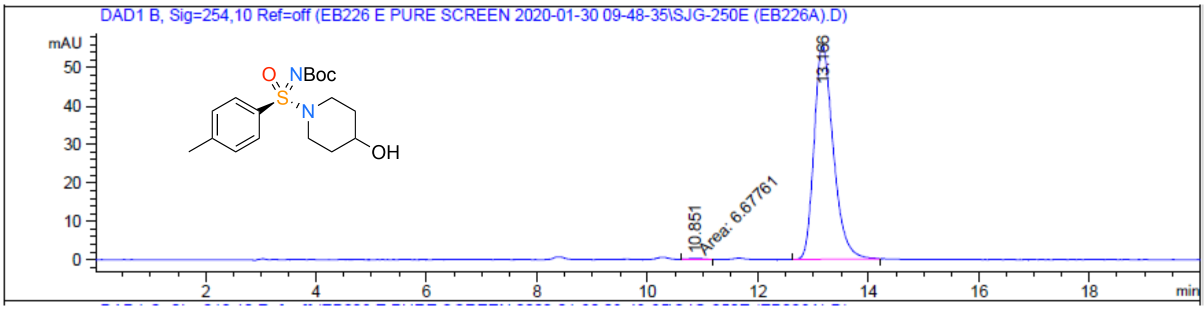

Signal 2: DAD1 B, Sig=254,10 Ref=off

Peak #	RetTime [min]	Type	Width [min]	Area [mAU*s]	Height [mAU]	Area %
1	10.851	MM	0.3270	6.67761	3.40398e-1	0.4953
2	13.166	BB	0.3669	1341.47754	55.71297	99.5047
Totals :				1348.15515	56.05337	

***ee* = 99%**

tert-Butyl (R)-4-(N-(tert-butoxycarbonyl)-4-methylphenylsulfonimidoyl)piperazine-1-carboxylate ((R)-3p)

Conditions: Chiralpak IA column, 95:5 *n*hexane:*i*PrOH, flow rate: 1 mL min^{-1}, 35 °C, UV detection wavelength: 250 nm.

(*rac*)-(3p)

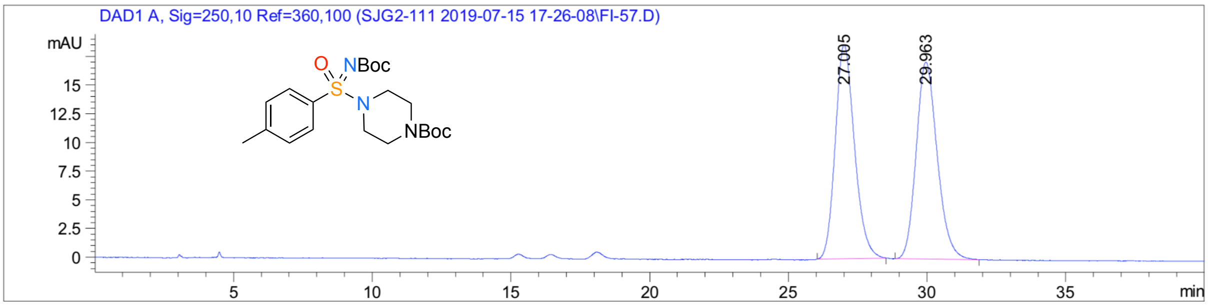

Signal 1: DAD1 A, Sig=250,10 Ref=360,100

Peak #	RetTime [min]	Type	Width [min]	Area [mAU*s]	Height [mAU]	Area %
1	27.005	BB	0.7199	898.88306	18.66262	49.8027
2	29.963	BB	0.7678	906.00513	17.16478	50.1973
Totals :				1804.88818	35.82741	

(*R*)-(3p)

$[\alpha]^{21}_D$ = -6 (c 1.0, $CHCl_3$).

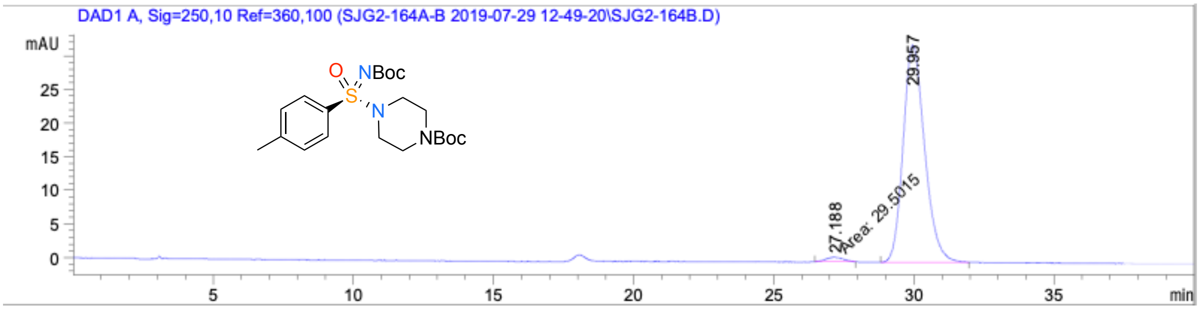

Signal 1: DAD1 A, Sig=250,10 Ref=360,100

Peak #	RetTime [min]	Type	Width [min]	Area [mAU*s]	Height [mAU]	Area %
1	27.188	MM	0.7131	29.50145	6.89538e-1	1.6876
2	29.957	BB	0.8091	1718.63159	32.28175	98.3124
Totals :				1748.13304	32.97128	

ee = 97%

tert-Butyl (R)-(oxo(4-(pyrimidin-2-yl)piperazin-1-yl)(p-tolyl)-λ^6-sulfaneylidene)carbamate ((R)-3q)

Conditions: Chiralpak IB column, 97:3 *n*hexane:*i*PrOH, flow rate: 1 mL min^{-1}, 35 °C, UV detection wavelength: 250 nm.

(*rac*)-(3q)

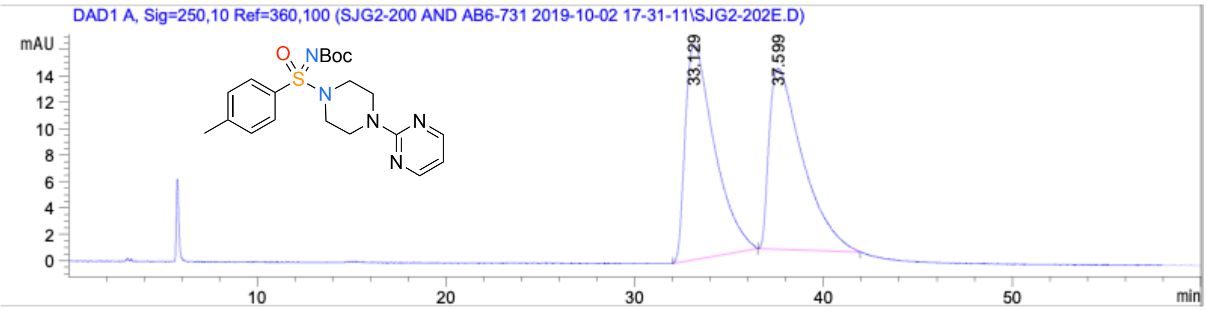

Signal 1: DAD1 A, Sig=250,10 Ref=360,100

Peak #	RetTime [min]	Type	Width [min]	Area [mAU*s]	Height [mAU]	Area %
1	33.129	BB	1.2832	1748.99963	16.28195	50.8502
2	37.599	BB	1.4582	1690.51697	13.71306	49.1498
Totals :				3439.51660	29.99501	

(*R*)-(3q)

$[\alpha]^{23}_D$ = 0 (c 1, $CHCl_3$).

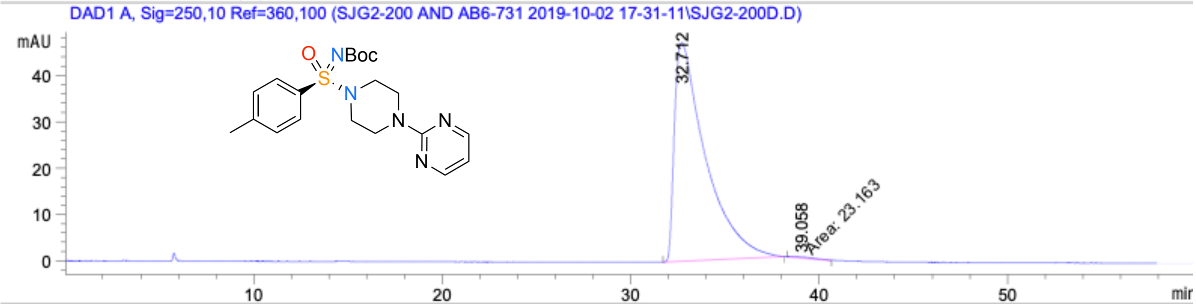

Signal 1: DAD1 A, Sig=250,10 Ref=360,100

Peak #	RetTime [min]	Type	Width [min]	Area [mAU*s]	Height [mAU]	Area %
1	32.712	BB	1.4899	5410.35449	47.17966	99.5737
2	39.058	MM	1.3907	23.16299	2.77596e-1	0.4263
Totals :				5433.51748	47.45725	

***ee* > 99%**

tert-Butyl (_R_)-((4-(6-fluorobenzo[_d_]isoxazol-3-yl)piperidin-1-yl)(oxo)(_p_-tolyl)-λ^6-sulfaneylidene)carbamate ((_R_)-3r)

Conditions: Chiralpak IA column, 90:10 *n*hexane:*i*PrOH, flow rate: 1 mL min^{-1}, 35 °C, UV detection wavelength: 250 nm.

(_rac_)-(3r)

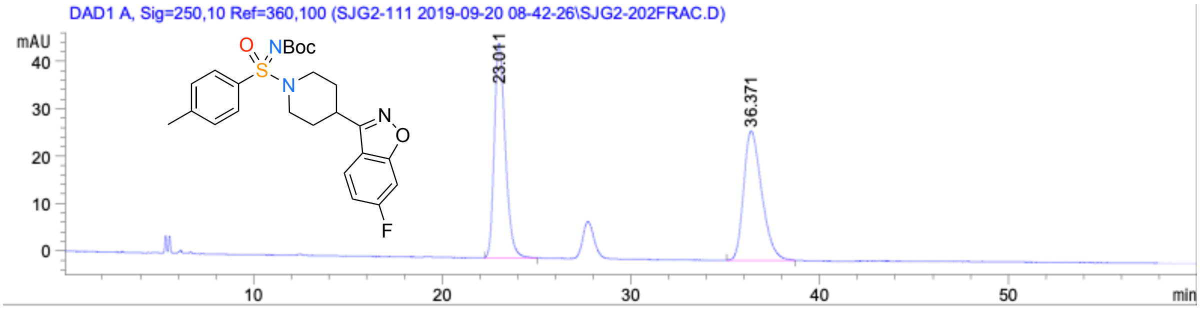

Signal 1: DAD1 A, Sig=250,10 Ref=360,100

Peak #	RetTime [min]	Type	Width [min]	Area [mAU*s]	Height [mAU]	Area %
1	23.011	BB	0.6102	1826.53259	45.32460	50.1203
2	36.371	BB	0.9385	1817.76233	27.38116	49.8797
Totals :				3644.29492	72.70576	

(_R_)-(3r)

[α]$^{23}_{D}$ = +20 (c 0.5, $CHCl_3$).

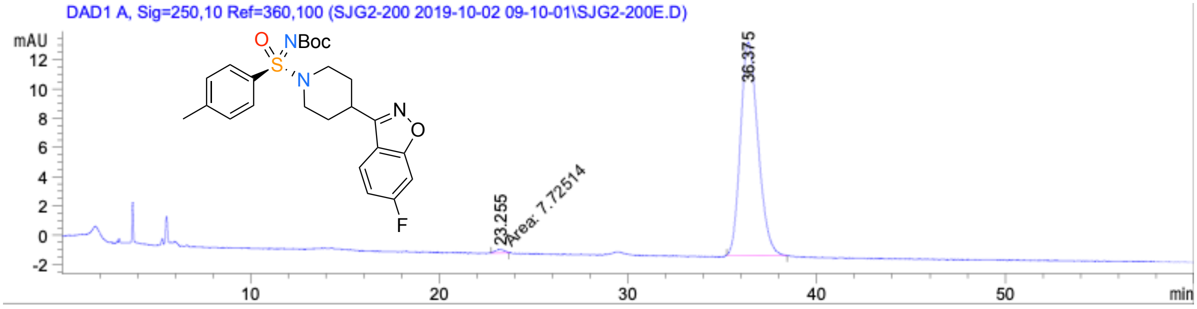

Signal 1: DAD1 A, Sig=250,10 Ref=360,100

Peak #	RetTime [min]	Type	Width [min]	Area [mAU*s]	Height [mAU]	Area %
1	23.255	MM	0.5510	7.72514	2.33659e-1	0.8118
2	36.375	BB	0.8939	943.83289	14.60569	99.1882
Totals :				951.55803	14.83935	

***ee* = 98%**

***tert*-Butyl (*R*)-(((3-(10,11-dihydro-5*H*-dibenzo[*b*,*f*]azepin-5-yl)propyl)(methyl)amino)(oxo)(*p*-tolyl)-λ^6-sulfaneylidene)carbamate ((*R*)-3s)**

Conditions: Chiralpak IA column, 95:5 *n*hexane:*i*PrOH, flow rate: 1 mL min^{-1}, 35 °C, UV detection wavelength: 290 nm.

(*rac*)-(3s)

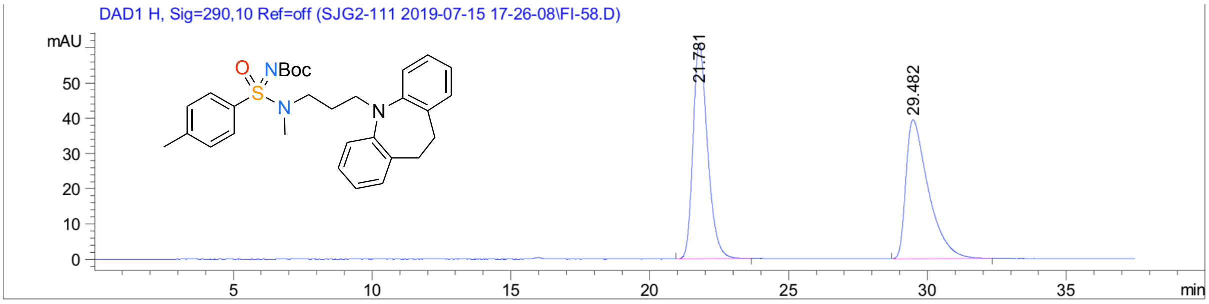

Signal 8: DAD1 H, Sig=290,10 Ref=off

Peak #	RetTime [min]	Type	Width [min]	Area [mAU*s]	Height [mAU]	Area %
1	21.781	BB	0.5561	2224.52295	61.19504	50.0466
2	29.482	BB	0.8284	2220.38013	39.46446	49.9534

Totals : 4444.90308 100.65950

(*R*)-(3s)

$[\alpha]^{23}_{D}$ = +8 (c 1.0, $CHCl_3$).

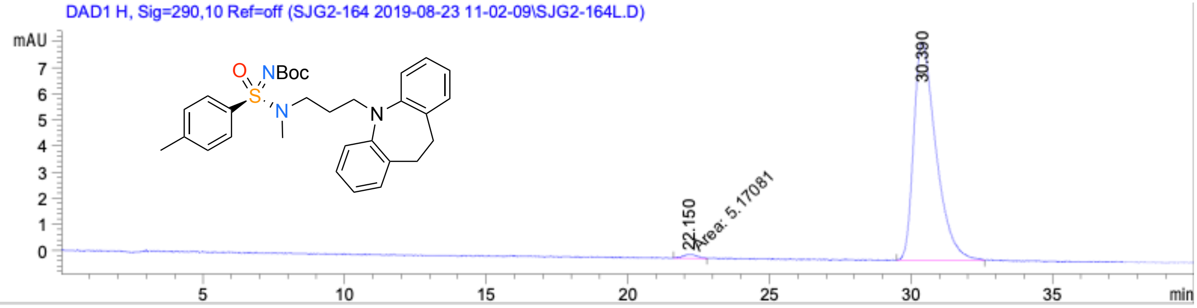

Signal 8: DAD1 H, Sig=290,10 Ref=off

Peak #	RetTime [min]	Type	Width [min]	Area [mAU*s]	Height [mAU]	Area %
1	22.150	MM	0.5800	5.17081	1.48593e-1	1.1202
2	30.390	BB	0.7305	456.44647	8.36694	98.8798

Totals : 461.61729 8.51553

***ee* = 98%**

Methyl 3-((4-bromophenyl)sulfinyl)propanoate ((*S*)-8)

Conditions: Chiralpak IA column, 90:10 *n*hexane:*i*PrOH, flow rate: 1 mL min^{-1}, 35 °C, UV detection wavelength: 250 nm. Retention times: 19 & 21 min.

(*rac*)-8

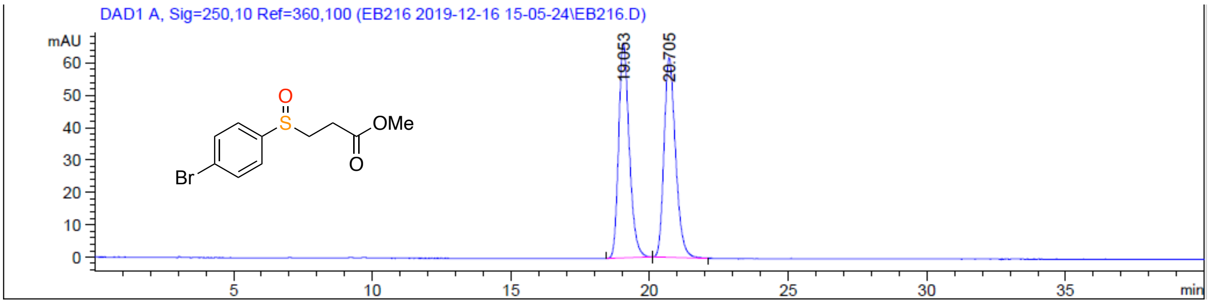

Signal 1: DAD1 A, Sig=250,10 Ref=360,100

Peak #	RetTime [min]	Type	Width [min]	Area [mAU*s]	Height [mAU]	Area %
1	19.053	BB	0.4091	1772.47925	66.46956	49.9404
2	20.705	BB	0.4420	1776.70703	61.69768	50.0596
Totals :				3549.18628	128.16724	

(*S*)-(8)

$[\alpha]^{23}_{D}$ = –98 (c 1.0, $CHCl_3$)

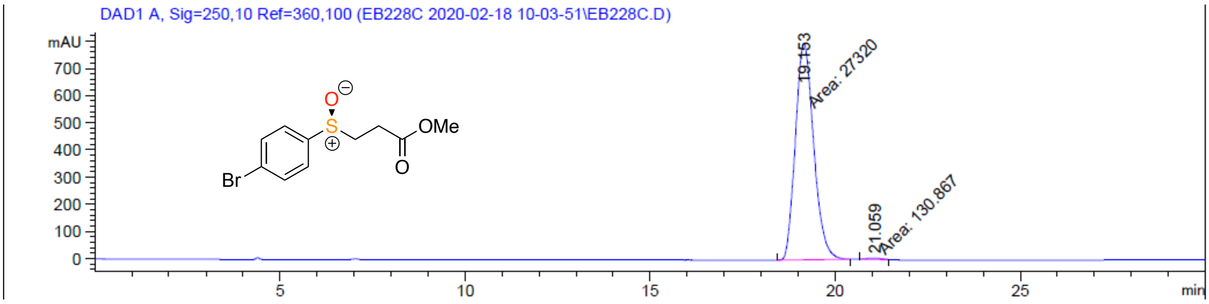

Signal 1: DAD1 A, Sig=250,10 Ref=360,100

Peak #	RetTime [min]	Type	Width [min]	Area [mAU*s]	Height [mAU]	Area %
1	19.153	MM	0.5728	2.73200e4	794.92761	99.5233
2	21.059	MM	0.4381	130.86740	4.97806	0.4767
Totals :				2.74509e4	799.90567	

***ee* = 99%**

Methyl (*S*)-3-(4-bromo-*N*-(*tert*-butoxycarbonyl)phenylsulfonimidoyl)propanoate ((*S*)-9)

Conditions: Chiralpak IA column, 93:7 *n*hexane:*i*PrOH, flow rate: 1 mL min^{-1}, 35 °C, UV detection wavelength: 250 nm.

(*rac*)-9

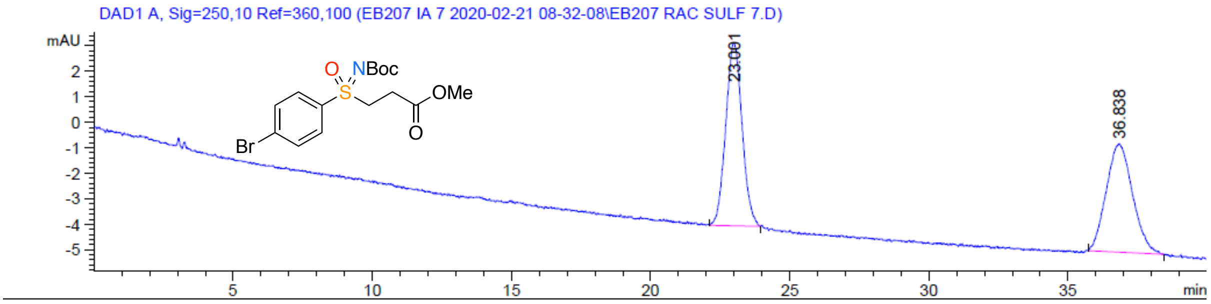

Signal 1: DAD1 A, Sig=250,10 Ref=360,100

Peak #	RetTime [min]	Type	Width [min]	Area [mAU*s]	Height [mAU]	Area %
1	23.001	BB	0.5177	292.65317	7.17716	51.1008
2	36.838	BB	0.7778	280.04456	4.25531	48.8992
Totals :				572.69772	11.43247	

(*S*)-9

$[\alpha]^{23}_{D}$ = +44 (c 1.0, $CHCl_3$)

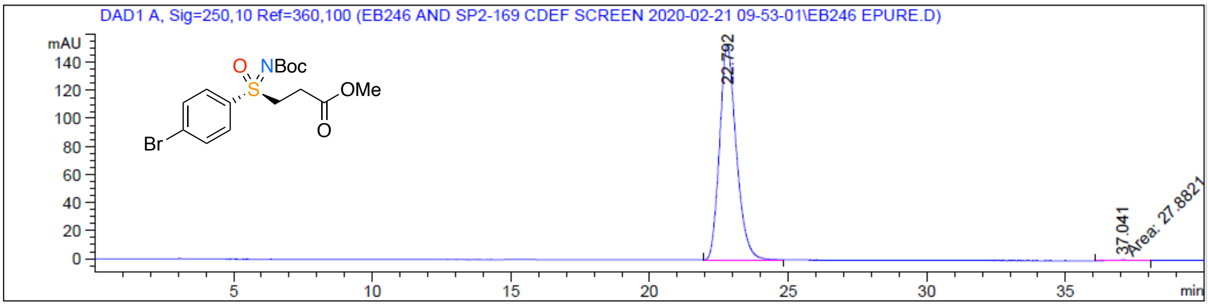

Signal 1: DAD1 A, Sig=250,10 Ref=360,100

Peak #	RetTime [min]	Type	Width [min]	Area [mAU*s]	Height [mAU]	Area %
1	22.792	BB	0.6193	6407.31689	153.39117	99.5667
2	37.041	MM	0.9987	27.88206	4.65299e-1	0.4333
Totals :				6435.19896	153.85647	

***ee* = 99%**

Sodium (*S*)-((4-Bromophenyl)sulfinyl)(*tert*-butoxycarbonyl)amide ((*S*)-1b)

The ee of the sulfinamide salt was tested by reprotonation to the sulfinamide tert-Butyl (S)-((4-bromophenyl)sulfinyl)carbamate. For experimental conditions see experimental data for (S)-1a.

Conditions: Chiralpak IA column, 95:5 *n*hexane:*i*PrOH, flow rate: 1 mL min^{-1}, 35 °C, UV detection wavelength: 250 nm.

(*rac*)-*tert*-Butyl ((4-bromophenyl)sulfinyl)carbamate

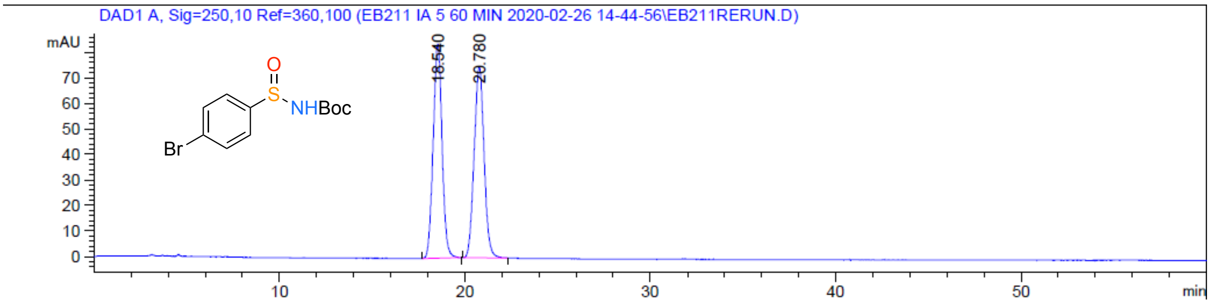

Signal 1: DAD1 A, Sig=250,10 Ref=360,100

Peak #	RetTime [min]	Type	Width [min]	Area [mAU*s]	Height [mAU]	Area %
1	18.540	BB	0.5101	2789.14917	83.83952	50.0487
2	20.780	BB	0.5762	2783.71631	74.80299	49.9513
Totals :				5572.86548	158.64250	

(*S*)-*tert*-Butyl ((4-bromophenyl)sulfinyl)carbamate

$[\alpha]^{23}_{D}$ = +88 (c 1.0, $CDCl_3$)

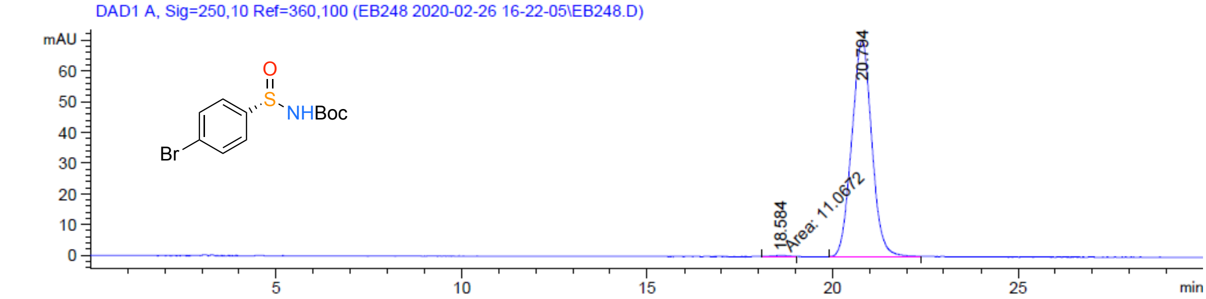

Signal 1: DAD1 A, Sig=250,10 Ref=360,100

Peak #	RetTime [min]	Type	Width [min]	Area [mAU*s]	Height [mAU]	Area %
1	18.584	MM	0.4719	11.06724	3.90911e-1	0.4186
2	20.794	BB	0.5750	2633.01880	70.30122	99.5814
Totals :				2644.08604	70.69213	

***ee* = 99%**

tert-Butyl (*R*)-((4-bromophenyl)fluoro(oxo)-λ^6-sulfaneylidene)carbamate ((*R*)-2b)

Conditions: Chiralpak IA column, 98:2 *n*hexane:*i*PrOH, flow rate: 1 mL min^{-1}, 35 °C, UV detection wavelength: 250 nm.

(*rac*)-2b

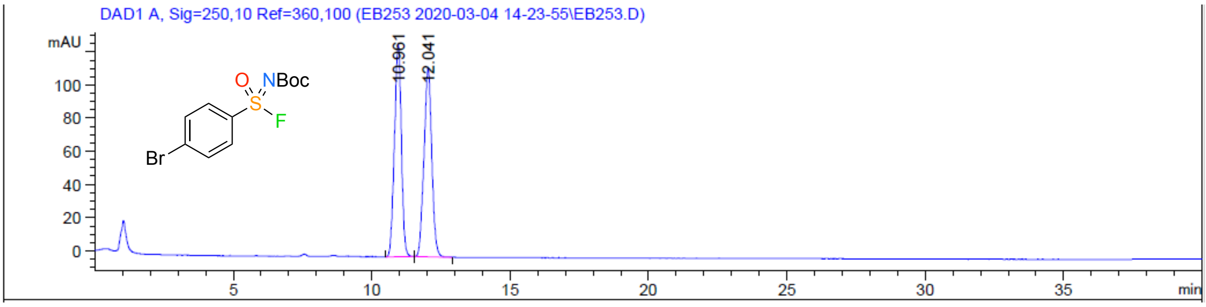

Signal 1: DAD1 A, Sig=250,10 Ref=360,100

Peak #	RetTime [min]	Type	Width [min]	Area [mAU*s]	Height [mAU]	Area %
1	10.961	BB	0.2623	2229.76563	128.59897	50.0398
2	12.041	BB	0.2921	2226.22266	113.86397	49.9602
Totals :				4455.98828	242.46294	

(*S*)-2b

$[\alpha]^{23}_D$ = –15 (c 1.7, $CDCl_3$)

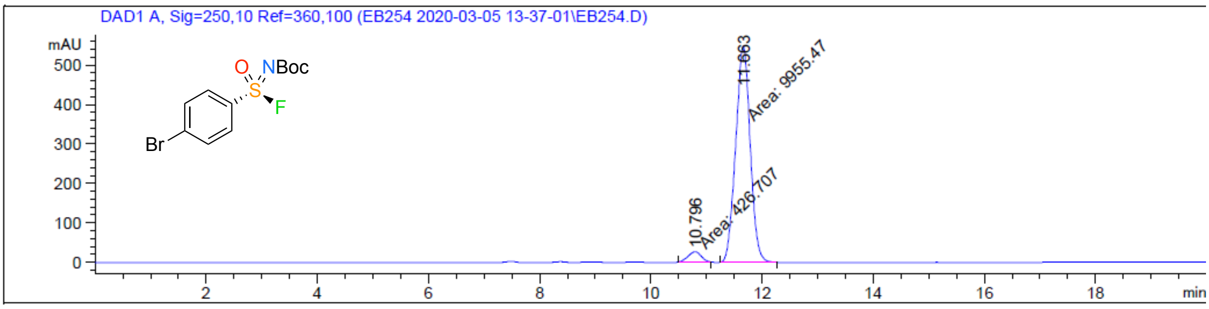

Signal 1: DAD1 A, Sig=250,10 Ref=360,100

Peak #	RetTime [min]	Type	Width [min]	Area [mAU*s]	Height [mAU]	Area %
1	10.796	MM	0.2715	426.70654	26.19217	4.1100
2	11.663	MM	0.3022	9955.47168	549.06299	95.8900
Totals :				1.03822e4	575.25516	

***ee* = 92%**

tert-Butyl (_R_)-((benzylamino)(4-bromophenyl)(oxo)-λ⁶-sulfaneylidene)carbamate ((_R_)-3v)

Conditions: Chiralpak ID column, 90:10 _n_hexane:_i_PrOH, flow rate: 1 mL min^{-1}, 35 °C, UV detection wavelength: 254 nm.

(_rac_)-3v

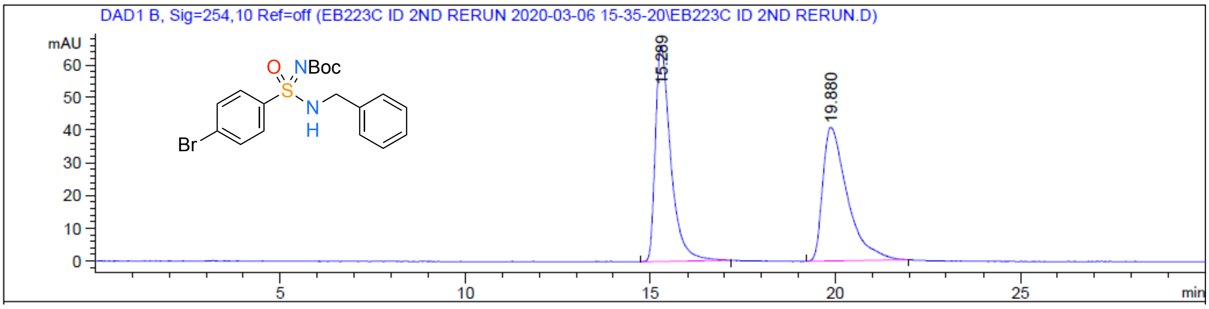


```
Signal 2: DAD1 B, Sig=254,10 Ref=off

Peak RetTime Type  Width     Area      Height     Area
  #   [min]        [min]   [mAU*s]     [mAU]        %
----|-------|----|-------|----------|----------|--------|
   1  15.289 BB    0.4308 1897.99060   66.13738  49.9878
   2  19.880 BB    0.6643 1898.91638   40.85403  50.0122

Totals :                  3796.90698  106.99141
```

(_R_)-3v

$[\alpha]^{23}_{D}$ = +6 (c 0.5, CH_2Cl_2)

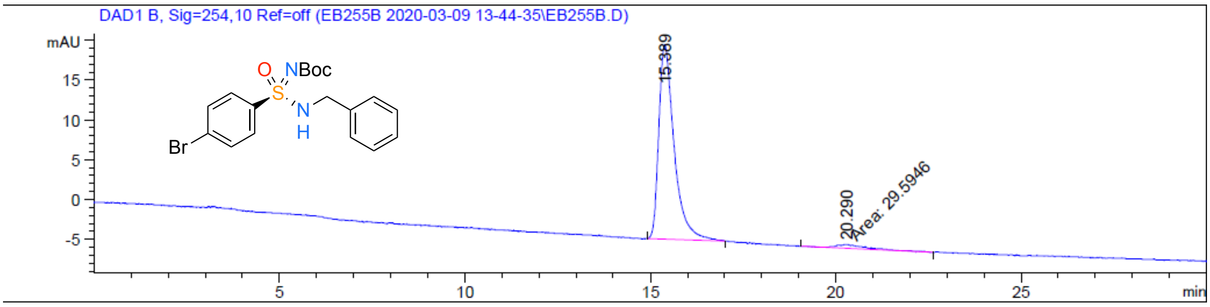


```
Signal 2: DAD1 B, Sig=254,10 Ref=off

Peak RetTime Type  Width     Area      Height     Area
  #   [min]        [min]   [mAU*s]     [mAU]        %
----|-------|----|-------|----------|----------|--------|
   1  15.389 BB    0.4214  703.08716   24.45546  95.9608
   2  20.290 MM    1.0244   29.59464 4.81516e-1   4.0392

Totals :                   732.68180   24.93698
```

***ee* = 92%**

tert-Butyl (R)-((allylamino)(4-bromophenyl)(oxo)-λ^6-sulfaneylidene)carbamate ((R)-3w)

Conditions: Chiralpak IA column, 97:3 *n*hexane:*i*PrOH, flow rate: 1 mL min^{-1}, 35 °C, UV detection wavelength: 250 nm.

(*rac*)-3w

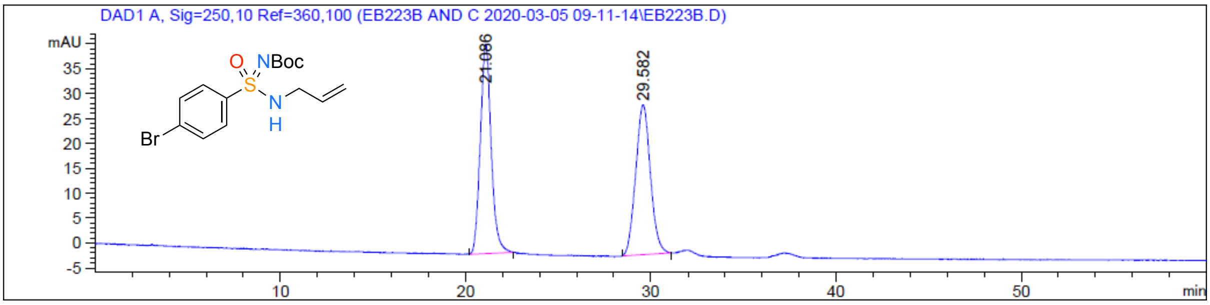

Signal 1: DAD1 A, Sig=250,10 Ref=360,100

Peak #	RetTime [min]	Type	Width [min]	Area [mAU*s]	Height [mAU]	Area %
1	21.086	BB	0.5924	1710.61499	42.06887	50.7446
2	29.582	BB	0.7395	1660.41260	30.10318	49.2554
Totals :				3371.02759	72.17204	

***(R)*-3w**

$[\alpha]^{23}_D$ = +8 (c 0.5, CH_2Cl_2)

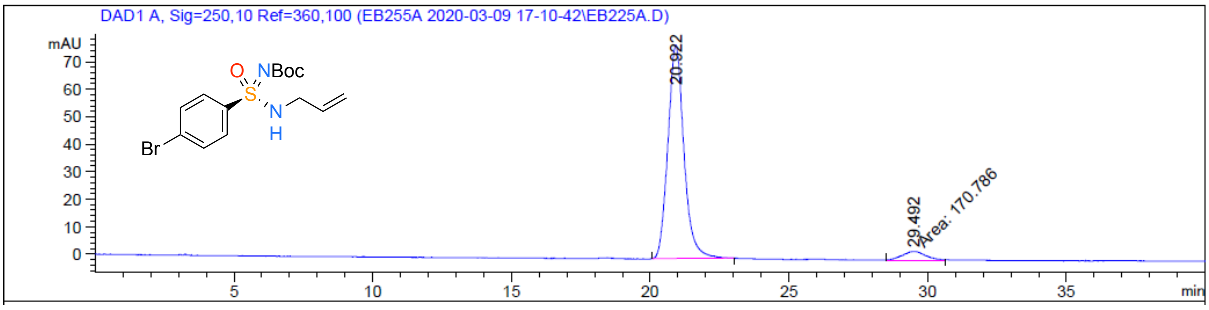

Signal 1: DAD1 A, Sig=250,10 Ref=360,100

Peak #	RetTime [min]	Type	Width [min]	Area [mAU*s]	Height [mAU]	Area %
1	20.922	BB	0.5810	3122.61719	77.67725	94.8143
2	29.492	MM	0.9223	170.78593	3.08615	5.1857
Totals :				3293.40312	80.76340	

***ee* = 90%**

tert-Butyl (R)-((4-bromophenyl)(oxo)(piperidin-1-yl)-λ⁶-sulfaneylidene)carbamate ((R)-3x)

Conditions: Chiralpak IA column, 95:5 *n*hexane:*i*PrOH, flow rate: 1 mL min^{-1}, 35 °C, UV detection wavelength: 250 nm.

(*rac*)-3x

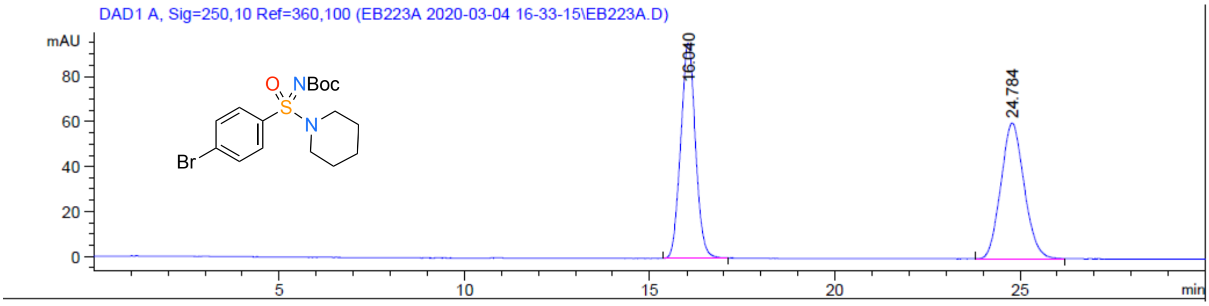

Signal 1: DAD1 A, Sig=250,10 Ref=360,100

Peak #	RetTime [min]	Type	Width [min]	Area [mAU*s]	Height [mAU]	Area %
1	16.040	BB	0.4242	2639.59253	95.57035	50.0593
2	24.784	BB	0.6478	2633.33691	60.26088	49.9407
Totals :				5272.92944	155.83123	

***(R)*-3x**

$[\alpha]^{23}_{D}$ = –8 (c 0.5, CH_2Cl_2)

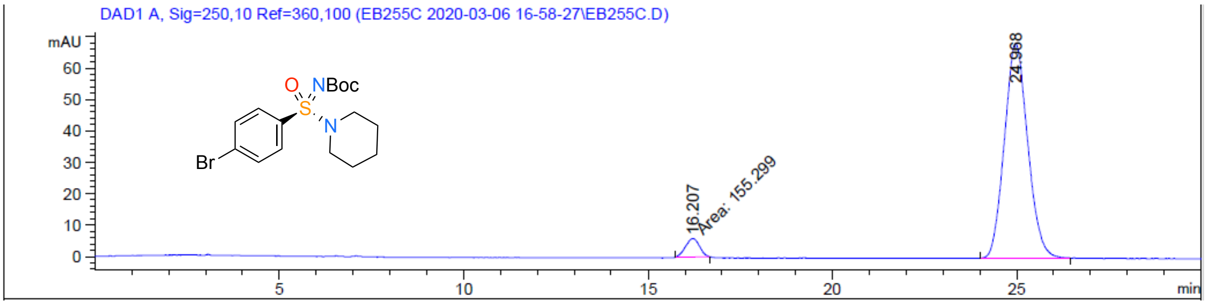

Signal 1: DAD1 A, Sig=250,10 Ref=360,100

Peak #	RetTime [min]	Type	Width [min]	Area [mAU*s]	Height [mAU]	Area %
1	16.207	MM	0.4393	155.29939	5.89220	4.9157
2	24.968	BB	0.6367	3003.94165	68.35349	95.0843
Totals :				3159.24104	74.24569	

***ee* = 90%**

tert-Butyl (_R_)-([1,1'-biphenyl]-4-yl(oxo)(piperidin-1-yl)-λ^6-sulfaneylidene)carbamate ((_R_)-10)

Conditions: Chiralpak IA column, 95:5 _n_hexane:_i_PrOH, flow rate: 1 mL min^{-1}, 35 °C, UV detection wavelength: 250 nm.

(_rac_)-10

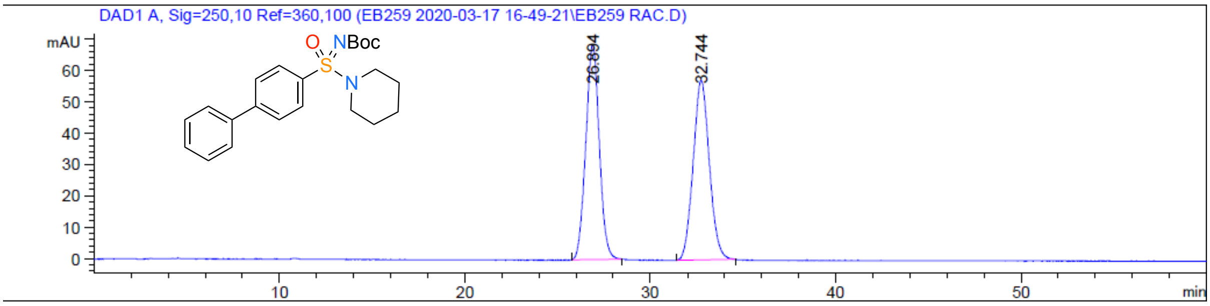

Signal 1: DAD1 A, Sig=250,10 Ref=360,100

Peak #	RetTime [min]	Type	Width [min]	Area [mAU*s]	Height [mAU]	Area %
1	26.894	BB	0.7426	3461.90698	68.61781	49.9489
2	32.744	BB	0.8816	3468.99438	57.19546	50.0511
Totals :				6930.90137	125.81327	

(_R_)-10

$[\alpha]^{23}_D$ = -7 (c 1.0, $CDCl_3$).

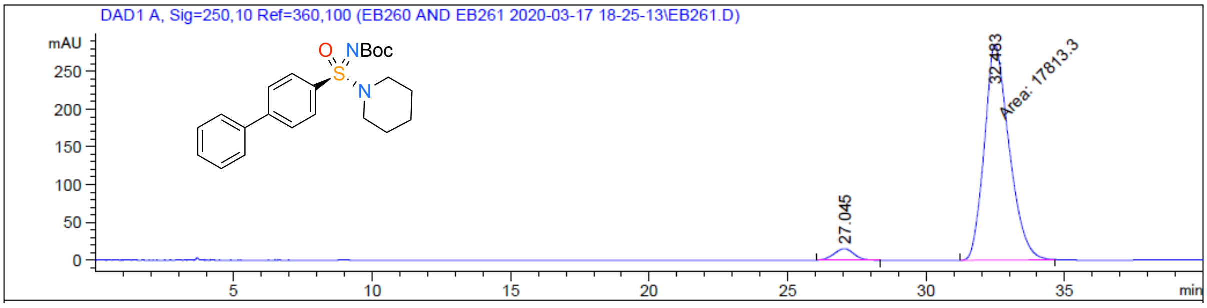

Signal 1: DAD1 A, Sig=250,10 Ref=360,100

Peak #	RetTime [min]	Type	Width [min]	Area [mAU*s]	Height [mAU]	Area %
1	27.045	BB	0.6254	763.23010	15.26192	4.1086
2	32.483	MM	1.0390	1.78133e4	285.74695	95.8914
Totals :				1.85765e4	301.00886	

_ee_ = 92%

1-(4-Methylphenylsulfonimidoyl)piperidine ((*R*)-11)

Conditions: Chiralpak IA column, 95:5 *n*hexane:*i*PrOH, flow rate: 1 mL min^{-1}, 35 °C, UV detection wavelength: 250 nm.

(*rac*)-11

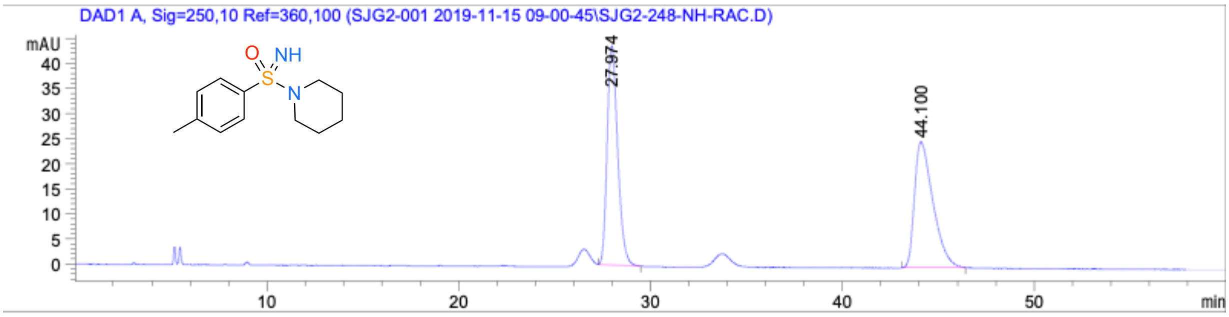

Signal 1: DAD1 A, Sig=250,10 Ref=360,100

Peak #	RetTime [min]	Type	Width [min]	Area [mAU*s]	Height [mAU]	Area %
1	27.974	BB	0.5747	1665.61560	43.70134	49.7831
2	44.100	BB	0.9287	1680.12842	25.17134	50.2169
Totals :				3345.74402	68.87268	

(*R*)-11

$[\alpha]^{23}_D$ = –8 (c 0.5, $CHCl_3$).

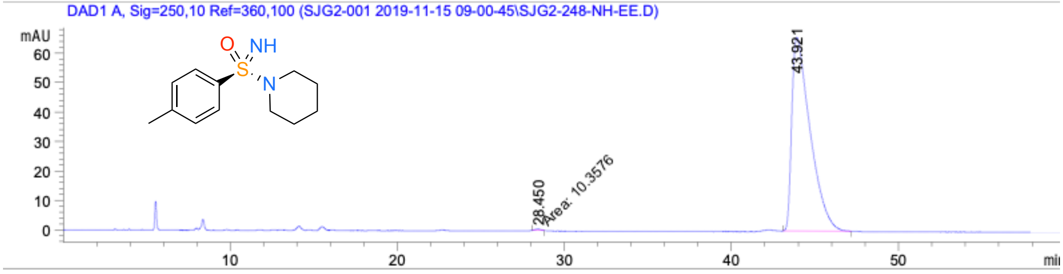

Signal 1: DAD1 A, Sig=250,10 Ref=360,100

Peak #	RetTime [min]	Type	Width [min]	Area [mAU*s]	Height [mAU]	Area %
1	28.456	MM	0.4303	10.69019	4.14060e-1	0.2128
2	43.921	BB	1.0525	5011.75488	65.80835	99.7872
Totals :				5022.44507	66.22241	

***ee* > 99%**

(*R*)-*N′*-Cyclobutyl-4-methylbenzenesulfonimidamide ((*R*)-12)

Conditions: Chiralpak IA column, 85:15 *n*hexane:*i*PrOH, flow rate: 1 mL min^{-1}, 35 °C, UV detection wavelength: 250 nm.

(*rac*)-12

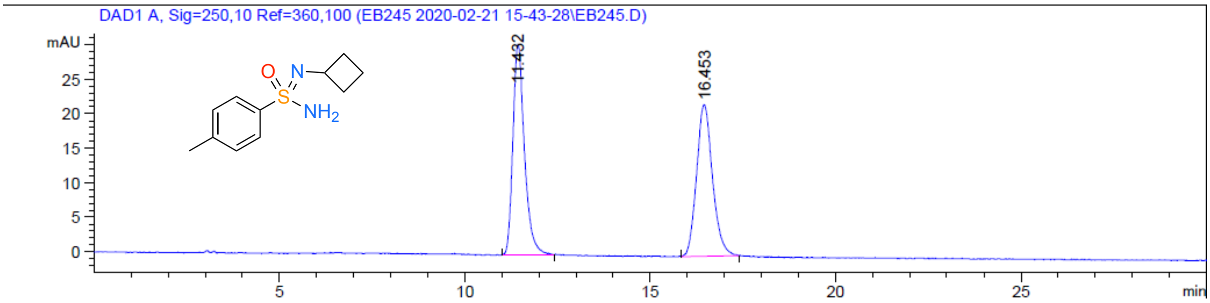

Signal 1: DAD1 A, Sig=250,10 Ref=360,100

Peak #	RetTime [min]	Type	Width [min]	Area [mAU*s]	Height [mAU]	Area %
1	11.432	BB	0.3197	658.97382	30.53941	50.2113
2	16.453	BB	0.4416	653.42694	21.93403	49.7887
Totals :				1312.40076	52.47344	

(*R*)-12

$[\alpha]^{23}_{D}$ = –46 (c 0.13, $CHCl_3$).

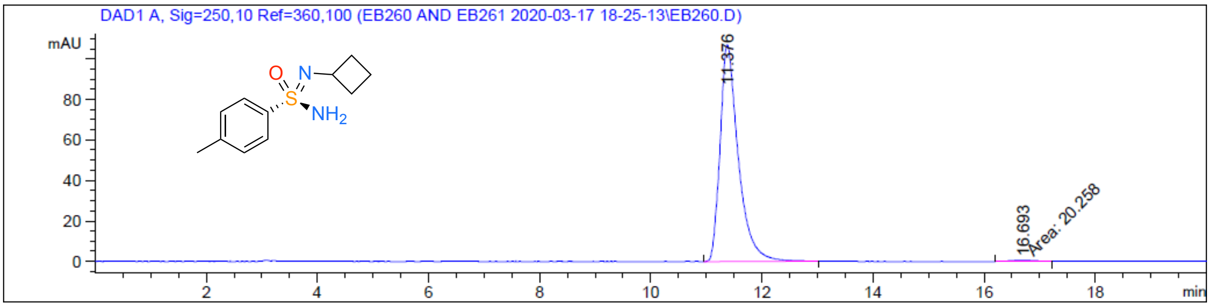

Signal 1: DAD1 A, Sig=250,10 Ref=360,100

Peak #	RetTime [min]	Type	Width [min]	Area [mAU*s]	Height [mAU]	Area %
1	11.376	BB	0.3356	2437.28149	107.06171	99.1757
2	16.693	MM	0.5273	20.25801	6.40252e-1	0.8243
Totals :				2457.53950	107.70197	

***ee* = 98%**
